# The Impact of MicroRNA-223-3p on IL-17 Receptor D Expression in Synovial Cells

**DOI:** 10.1371/journal.pone.0169702

**Published:** 2017-01-05

**Authors:** Nozomu Moriya, Seiji Shibasaki, Miki Karasaki, Tsuyoshi Iwasaki

**Affiliations:** 1 Department of Biopharmaceutics, School of Pharmacy, Hyogo University of Health Sciences, 1-3-6 Minatojima, Chuo-Ku, Kobe, Hyogo, Japan; 2 General Education Center, Hyogo University of Health Sciences, 1-3-6 Minatojima, Chuo-ku, Kobe, Hyogo, Japan; 3 Division of Rheumatology, Department of Internal Medicine, Hyogo College of Medicine, 1-1Mukogawa-cho, Nishinomiya, Hyogo, Japan; 4 Department of Pharmacotherapy, School of Pharmacy, Hyogo University of Health Sciences, 1-3-6 Minatojima, Chuo-ku, Kobe, Hyogo, Japan; Universitat des Saarlandes, GERMANY

## Abstract

**Objective:**

Rheumatoid arthritis (RA) is an autoimmune inflammatory disease affecting joints. Elevated plasma levels of microRNA-223-3p (miR-223-3p) in patients with RA are implicated in the pathogenesis of the disease. This study aimed to analyze the functional role of miR-223-3p in the pathogenesis of RA by overexpressing miR-223-3p in synovial cell lines.

**Methods:**

Arthritis was induced in the RA model of SKG mice by injection of ß-glucan. The histopathologic features of joints were examined using hematoxylin and eosin and immunohistochemical staining. Plasma levels of miRNA were determined by panel real-time PCR analysis. Target genes of the differentially expressed miRNAs in SKG mice were analyzed using miRNA target prediction algorithms. The dual-luciferase reporter system was used to evaluate the relationship between miR-223-3p and IL-17 receptor D (IL-17RD). The activity of miR-223-3p was analyzed by transfection of plasmid vectors overexpressing miR-223-3p into IL-17RD-expressing NIH3T3 and MH7A cell lines. *Il6* and *Il17rd* mRNA expression was analyzed by quantitative real-time PCR. IL-17RD protein expression was analyzed by western blot analysis.

**Results:**

We identified 17 upregulated miRNAs (fold change > 2.0) in plasma of SKG mice injected with ß-glucan relative to untreated SKG mice. *Il17rd* was identified as the candidate target gene of miR-223-3p using five miRNA target prediction algorithms. The transfection of plasmid vectors overexpressing miR-223-3p into NIH3T3 and MH7A cells resulted in the downregulation of *Il17rd* expression and upregulation of *Il6* expression. Expression of miR-223-3p and *Il6* mRNA in MH7A cells was upregulated; however, that of *Il17rd* mRNA was downregulated following TNF-α stimulation. IL-17RD expression in synovial tissues from SKG mice and RA patients was inversely correlated with the severity of arthritis.

**Conclusion:**

This study is the first to demonstrate that miR-223-3p downregulates IL-17RD in both mouse and human cells; miR-223-3p may contribute to the pathogenesis of RA by downregulating the expression of IL-17RD and upregulating that of IL-6 in synovial cells.

## Introduction

MicroRNAs (miRNAs) are short non-coding RNAs that influence messenger RNA (mRNA) processing at the post-transcriptional level [1] via interacting at the 3’-untranslated region (UTR) [2] and inducing translational repression or mRNA degradation, thereby controlling the expression of protein-coding mammalian genes [3, 4]. miRNAs, which are involved in the pathogenesis of a variety of diseases [5], circulate in the blood in a stable form; this property makes these RNAs attractive as biomarkers that enable non-invasive testing [6].

Rheumatoid arthritis (RA) is characterized by synovial cell proliferation, which causes joint destruction [7]. Numerous studies have identified dysregulated miRNAs in blood cells and plasma of patients with RA. However, these results appeared discordant [8–15]. High expression level of miR-223-3p is seen in myeloid cells and upregulation of miR-223-3p is an important element of myeloid cell differentiation [16–18]. miR-223-3p is overexpressed in the synovium and peripheral T cells of patients with RA [12, 19–21]. However, the precise role of miR-223-3p in the pathogenesis of RA is still unknown.

The IL-17 cytokine family consists of six ligands (IL-17A-F), which signal through five receptors (IL-17RA-E) [22, 23]. To date, the ligands for IL-17R members have been identified and the role of IL-17 signaling has been delineated in many inflammatory and autoimmune diseases. However, the ligand for IL-17 receptor D (IL-17RD) and its physiological role is still unknown [24].

In the present study, we evaluated plasma miRNA levels in RA model of SKG mice, and found a significant increase in the plasma levels of miR-223-3p. We additionally report that miR-223-3p targets molecules involved in IL-17RD expression, thereby downregulating IL-17RD levels and that miR-223-3p upregulates IL-6 induction in the IL-17RD expressed synovial cells. Our findings describe novel interplay mechanisms between IL-17R and miRNA families involved in RA.

## Materials and Methods

### Mice

Female SKG mice (7–8 weeks of age) were purchased from CLEA Japan, Inc. (Tokyo, Japan) and maintained under specific pathogen-free conditions in the animal facility of the Hyogo College of Medicine (Nishinomiya, Hyogo, Japan). Animal experiments were performed in accordance with the guidelines of the National Institutes of Health (Bethesda, MD, USA), as specified by the animal care policy of the Hyogo College of Medicine. All experimental procedures were reviewed and approved by the Animal Care and Use Committee of Hyogo College of Medicine (Application number:15–068) [25–27]. Temperature, humidity and light-dark cycle controlled conditions were maintained and allowed food and water and were routinely tested for background pathogens. All efforts were made to prevent animal suffering. In addition, mice numbers were kept as small as necessary for appropriate statistical analyses. During the experiments mice were monitored daily for any signs of pain and distress, and weight loss. To minimize suffering of animal mice were sacrificed by cervical dislocation when statistical analysis of arthritis score data was completed.

### Clinical assessment of SKG arthritis

A single intraperitoneal injection of ß-glucan (45 mg) was performed for the induction of arthritis. Arthritis scores (0, no swelling; 0.1, swelling of one toe joint; 0.5, mild ankle swelling; and 1.0, severe ankle swelling) for all toes and ankles were monitored daily by inspection and the scores for all toes and ankles were totaled for each mouse [26, 27].

### Plasma collection and RNA isolation

Blood was collected from mice into tubes containing EDTA and processed for plasma isolation. To obtain plasma, we centrifuged samples at 1000 × g for 20 min at 4°C and transferred the supernatant into new tubes. Plasma samples were stored at −80°C until use for RNA extraction. Total RNAs, including small RNA molecules, were extracted from 100–200 μL of plasma or the cells using a miRNeasy kit (Qiagen, Tokyo, Japan), according to the manufacturer's instructions.

### Panel real-time PCR analysis

MiRNA expression was analyzed using panel real-time PCR analysis (miRCURY LNA™ microRNA PCR) of pooled plasma from SKG mice injected with ß-glucan (n = 5) and untreated SKG mice (n = 5) with primers for 632 mouse miRNAs and 348 rat miRNAs. All experiments were conducted at CosmoBio Services (Tokyo, Japan). Briefly, 18 μL of RNA was reverse transcribed in a total reaction volume of 90 μL, using the miRCURY LNA™ Universal reverse transcription (RT) microRNA PCR, Polyadenylation and cDNA synthesis kit (Exiqon, Vedbaek, Denmark). Each RT was performed once, including an artificial RNA spike-in (UniSp6). cDNA was diluted 50 × (90 μL of cDNA + 4410 μL of water) and assayed in 10-μL PCR reactions using the miRCURY LNA™ Universal RT microRNA PCR protocol. Each microRNA was assayed once by qPCR using the microRNA Ready-to-Use PCR kit, Mouse&Rat panel I and panel II, V2. The amplification was performed using a LightCycler 480 Real-Time PCR System (Roche, Basel, Switzerland) in 384-well plates. The amplification curves were analyzed using Roche LC software (ver. 1.5), both for determination of the crossing point (Cp), by the 2nd derivative method, and for melting curve analysis. Data were analyzed as follows: raw data was extracted from the LightCycler 480 software. Data was internally calibrated using UniSp3 IPC using GenEx software (ver.5) (Exiqon). Only data with Cp < 37 or at least 3 Cp lower than the value of the negative control was included in the analysis. GenEx software was used for data analysis. All data were normalized to the average Cp detected for the sample (normalized Cp = average Cp [< 37]–target Cp).

### RT reaction

The RT reaction was carried out using two different kits, depending on the type of RNA to be reverse transcribed. For miRNA detection, cDNAs were synthesized using miScript II RT reagent kit (Qiagen). Briefly, 1 μg of total RNA from each sample was resuspended in 20 μl of reaction buffer with 4 μl of 5 × miScript Hispec Buffer and 2 μl of miScript Reverse Transcriptase Mix, and incubated at 37°C for 60 min, at 95°C for 5 min, and then held at 4°C. The cDNAs were stored at −20°C until PCR. For mRNA detection, cDNAs were synthesized using PrimeScript RT reagent Kit (Takara Bio, Shiga, Japan), as described previously [28].

### Quantitative real-time PCR

miRNA or mRNA expression was measured by real-time PCR using the 7500 Fast Real-Time PCR System (Applied Biosystems, Foster City, CA, USA). For measurement of miRNA expression levels, real-time PCR was performed using the miScript SYBR Green PCR kit (Qiagen): 2 μl of cDNA solution was amplified using 12.5 μl of 2 × QuantiTect SYBR Green PCR Master Mix, 2.5 μl of 10 × miScript Universal Primer, 2.5 μl of 10 μM miRNA-specific primers (Table 1), and 5.5 μl of nuclease-free water in a final volume of 25 μl. U6 RNA or miR-328-3p was used as a miRNA internal control in the cell line or plasma, respectively. We used miR-328-3p as miRNA internal control because the levels of miR-328-3p in untreated SKG mice were almost the same as those in ß-glucan-injected SKG mice. The Ct value of miR-328-3p in untreated SKG mice (n = 5) was 30.7 ± 0.85 (means ± S.E.), and that in ß-glucan-injected SKG mice (n = 5) was 30.6 ± 1.20 (S1 Fig). The reaction mixtures were incubated at 95°C for 10 min, followed by 40 cycles of 94°C for 15 s, 55°C for 30 s, and 70°C for 34 s. All experiments were conducted in duplicate using the same sample. The cycle threshold (Ct) values were calculated with the SDS 1.4 software (Applied Biosystems). The specificity of the PCR product was routinely monitored by checking the product melting curves (dissociation curves) for each reaction. The expression level was determined using the delta-delta Ct method. Results were expressed as the relative fold-change of the target miRNA compared with that of a control sample. For measurement of mRNA expression real-time PCR was conducted using gene-specific primers (Table 1) and SYBR Premix Ex TaqII (TaKaRa Bio), as previously described [28]. We determined mRNA expression using the delta-delta Ct method, normalized to that of GAPDH (glyceraldehyde-3-phosphate dehydrogenase) in the same sample.

**Table 1:** Primer for real-time PCR.

Primer	Sequence
mmu-miR-1195	TGAGTTCGAGGCCAGCCTGCTCA
mmu-miR-223-3p	TGTCAGTTTGTCAAATACCCCA
mmu-miR-129-2-3p	AAGCCCTTACCCCAAAAAGCAT
mmu-miR-709	GGAGGCAGAGGCAGGAGGA
mmu-miR-224-5p	TAAGTCACTAGTGGTTCCGTT
mmu-miR-328-3p	CTGGCCCTCTCTGCCCTTCCGT
U6	ACGCAAATTCGTGAAGCGTT
mouse IL17rd for	AACAGCGGACTGCACAACAT
mouse IL17rd rev	GCAAGCGTACTGGCTGATG
human IL-17rd for	ATGCTTGCCATGACCAAGTG
human IL-17rd rev	AGCTCCTCCAGTATTACCCGA
mouse IL6 for	TAGTCCTTCCTACCCCAATTTCC
mouse IL6 rev	TTGGTCCTTAGCCACTCCTTC
human IL6 for	ACTCACCTCTTCAGAACGAATTG
human IL6 rev	CCATCTTTGGAAGGTTCAGGTTG
mouse Gapdh for	AACTTTGGCATTGTGGAAGG
mouse Gapdh rev	GGATGCAGGGATGATGTTCT
human Gapdh for	GGCCTCCAAGGAGTAAGACC
human Gapdh rev	AGGGGAGATTCAGTGTGGTG

### miRNA target gene prediction

In order to identify candidate miRNA target genes, we adopted five commonly used miRNA target prediction algorithms (miRWalk, DIANA-microT, PITA, RNAhybrid, Targetscan), available on the Internet. The prediction methods used by these programs are based on: 1) Watson-Crick base pairing between miRNAs and the 3’UTR of their target sequences, 2) evolutionary conservation of the sequences involved in miRNA and target mRNA binding, 3) the thermodynamic free energy of the miRNA-mRNA duplex, and 4) the binding site accessibility in the secondary structure of the target mRNA. Since each program has its own characteristics, we cross-validated the output of the five programs to identify true positive target mRNAs with greater accuracy [29]. Finally, we extracted the common target genes predicted by all the algorithms.

### Plasmid construction

In order to construct the miR-223-3p overexpression vector (pBA/miR-223-3p), the following oligonucleotides were annealed and inserted into the BamHI/HindIII site of pBAsi-hU6 Neo or Puro vector (TaKaRa Bio): 5’- GATCCGTGTCAGTTTGTCAAATACCCCAGTGTGCTGTCCTGGGGTATTTGACAAACTGACACTTTTTTA-3’ and 5’- AGCTTAAAAAAGTGTCAGTTTGTCAAATACCCCAGGACAGCACACTGGGGTATTTGACAAACTGACACG-3’ (the sequence of miR-223-3p is underlined). The negative control vector (pBA/NC) was similarly constructed using the scrambled oligonucleotide: 5’- GATCCGTAAGGCTATGAAGAGATACGTGTGCTGTCCGTATCTCTTCATAGCCTTACTTTTTTA-3’ and 5’- AGCTTAAAAAAGTAAGGCTATGAAGAGATACGGACAGCACACGTATCTCTTCATAGCCTTACG-3’ (the scrambled sequence is underlined [30]). In order to perform the luciferase assay, the 3’UTR of mouse *IL17rd* (*mIl17rd*) and human *IL17rd* (*hIl17rd*) genes containing miR-223-3p target sequences were amplified from mouse or human genomic DNA by PCR, using the following primers, *mIl17rd*-3’UTR F: 5’-GGACTCGGAAGAGTCTAAGCA-3’, *mIl17rd*-3’UTR R: 5’-TTACAAGAAAACATTTTATTTGATGTAGAA-3’, *hIl17rd*-3’UTR F: 5’-CAAAACGAAAGAGTCTAAGCATTG-3’, and *hIl17rd*-3’UTR R: 5’-TTAAAACAAAACATTTTATTTAATGCAGA-3’. The PCR products (*mIl17rd*; 5900 base pairs, *hIl17rd*; 6411 base pairs) were cloned into the pCR2.1-TOPO vector (TOPO TA Cloning Kit, Invitrogen, Carlsbad, CA, USA). Subsequently, the PCR products were subcloned into the pmirGLO luciferase vector (Promega, Madison, WI, USA), downstream of the firefly luciferase open reading frame, using the restriction endonucleases DraI, and XhoI, to construct a recombinant plasmid called pmG/*mIl17rd* and pmG/*hIl17rd*. The sequences of all plasmids were verified by DNA sequencing.

### Cell culture

HeLa and NIH3T3 cells were purchased from ATCC and maintained in Dulbecco’s Modified Eagle’s Medium (DMEM) (Wako, Osaka, Japan) supplemented with 10% fetal bovine serum, 100 units/ml penicillin, and 100 μg/ml streptomycin. Human MH7A cell line was obtained from Riken (Saitama, Japan) [31]. MH7A was established by transfection with the SV40 T antigen to synovial cells, isolated from intra-articular soft tissue of the knee joints of patients with RA. MH7A cells were maintained in RPMI1640 (Wako) supplemented with 10% fetal bovine serum, 100 units/ml penicillin, and 100 μg/ml streptomycin. All cells were cultured at 37°C and 5% CO2 in air.

### Luciferase assay

In order to evaluate the relationship between miR-223-3p and either *mIl17rd* or *hIl17rd*, the Dual-Luciferase Reporter System (Promega) was used. This approach, which has been widely used in vitro and in vivo, is utilized for the detection of protein–protein interactions as well as the experimental validation of miRNA targets. Herein, a firefly and renilla dual luciferase reporter system was employed to investigate the interaction between miR-223-3p and either *mIl17rd* or *hIl17rd*. One day before transfection, HeLa cells were seeded at 1,000 cells per well into 96-well plates (Thermo Scientific, Rockford, IL, USA). The next day, 20 ng of reporter plasmid, pmG/*mIl17rd* or pmG/*hIl17rd* (both pmG/*hIl17rd* wild type or deleted for the miR-223-3p seed site), was co-transfected with 80 ng of either miR-223-3p overexpression vector (pBA/miR-223-3p) or negative control vector (pBA/NC). Transfection was performed with Lipofectamine 3000 reagent (Invitrogen), according to the manufacturer’s protocol. At 48 h post-transfection, cells were washed with PBS and lysed with 40 μl of Reporter Lysis Buffer (Promega), followed by a freeze-thaw cycle. The lysates were used for luciferase assays. Luciferase activity was measured using Dual-Glo Luciferase Assay System kit (Promega) according the manufacturer’s protocol. Briefly, each lysate was incubated with an equal volume of Dual-Glo Luciferase Reagent. After 10 min at 20–25°C, firefly luminescence was measured at 470 nm using a luminometer (SpectraMax L luminescence microplate reader, Molecular Devices, Sunnyvale, CA, USA). Next, 40 μl of Dual-Glo Stop & Glo Reagent was added to each well and mixed. After incubation for 10 min, renilla luminescence was measured at 470 nm. Luciferase activity was calculated based on the ratio of the activities of firefly and renilla luciferases. All luciferase assay experiments were conducted in triplicate.

### Establishment of stable cell lines

**Fig 1:**
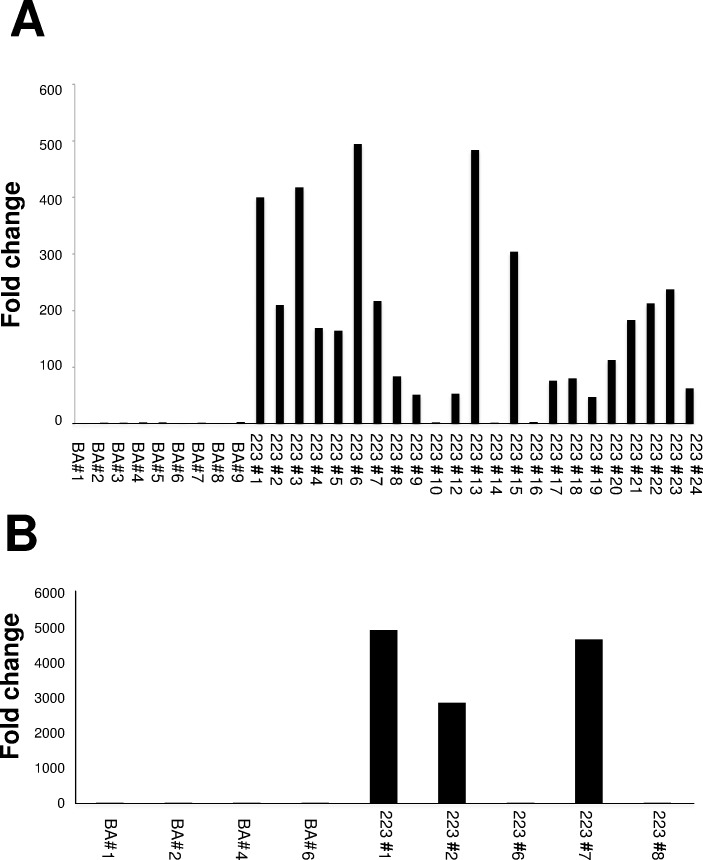
Expression of miR-223-3p in stable transfected cells. NIH3T3 cells (A) or MH7A cells (B) were transfected with pBA/miR-223-3p or pBA/NC and the resulting cells were individually picked for expansion using conventional cloning techniques. miR-223-3p miRNA was determined using real-time PCR. Results were expressed as the relative fold change following transfection with the negative control vector.

For transfection, NIH3T3 cells were grown to 90% confluence and transfected with pBA/miR-223-3p or pBA/NC using Lipofectamine 3000. In order to establish stable cell lines for miRNA overexpression, culture medium was replaced with fresh medium containing 800 μg/ml of G418 at 48 h after transfection. Transfected cells expressing the neomycin resistance gene were capable of survival in the selection media, and non-transfected cells were eliminated. The cells were individually picked for expansion 2 weeks later using conventional cloning techniques. Pools of 23 clones with pBA/miR-223-3p vectors or 9 pBA/NC control vectors were isolated as stable transfectants. Among the 23 clones selected using neomycin, high levels of expression of miR-223-3p miRNA were detected in several clones, such as #1, #3, #6, #13, and #15, using real-time PCR (Fig 1A). As MH7A already exhibited neomycin resistance, pBAsi-hU6 puro, which contains a puromycin resistance gene, was used for transfection; MH7A transfectants were therefore screened using medium containing 0.5 μg/ml of puromycin. Pools of 5 clones with pBA/miR-223-3p vectors or 4 pBA/NC control vectors were isolated as stable transfectants. Among the 5 clones selected using puromycin, high expression levels of miR-223-3p miRNA were detected in several clones, such as #1, #2, and #7, using real-time PCR (Fig 1B). We performed inhibition assay using chemically modified single stranded RNA molecules designated to specifically bind to and inhibit endogenous miRNA molecules (miR-223-3p inhibitor) or scrambles as anti-miRNA inhibitors negative control (Life Technologies, Madrid, Spain).

### Western blot analysis

Western blot analysis was performed using stable NIH3T3 and MH7A transfectant cells harboring pBA/miR-223-3p or pBA/NC vectors. Briefly, cells were lysed in RIPA lysis buffer and protein concentration was determined using Pierce BCA Protein assay kit (Thermo Scientific) with bovine serum albumin as standard. Each sample (20 μg of protein/lane) was separated by 4–15% SDS-PAGE. The separated proteins were transferred from the gel to a PVDF membrane (BioRad Laboratories, Hercules, CA, USA) at 350 mA for 1 h. After blocking for 1 h with 5% nonfat milk in Tris-buffered saline containing 0.05% Tween 20 (TBS-T), membranes were incubated overnight at 4°C with either rabbit anti-mouse IL-17RD, rabbit anti-human IL-17RD (1: 1,000 dilution in TBS-T; R&D, Minneapolis, MN, USA) or rabbit anti-β-actin antibody (1: 2,000 dilution in TBS-T; GeneTex, Irvine, CA, USA). After washing the membranes with TBS-T, goat anti-rabbit IgG-HRP antibody (1: 2,000 dilution in TBS-T; R&D) was added as a secondary antibody, followed by incubation for 1 h. After further washing, signals were detected with ECL western blotting detection reagent (GE Healthcare Japan, Tokyo, Japan) according to the manufacturer’s protocol. Relative densities were analyzed using ImageQuant TL (GE Healthcare Japan). Protein levels were normalized to that of β-actin in the same sample, and results were expressed as a percentage of those of the control.

### Histopathology

SKG mice joints were fixed in 10% formalin, decalcified, embedded in paraffin, sectioned and stained with hematoxylin and eosin. For immunohistochemical staining, cryostat sections of joints were fixed in cold acetone, washed in PBS and depleted of endogenous peroxidase by treatment with 0.3% H_2_O_2_. After blocking nonspecific binding with 10% normal rabbit serum in PBS, the sections were incubated with rabbit anti-mouse IL-6 antibody (Santa Cruz Biotechnology, Santa Cruz, CA, USA), rabbit anti-mouse TNF-α antibody (Santa Cruz), rabbit anti-mouse IL-17 antibody (Santa Cruz), or rabbit anti-mouse IL-17RD antibody (LifeSpan Biosciences, Inc. Seattle, WA, USA) at appropriate dilutions for 1 h at room temperature, washed, incubated with biotinylated goat anti-rabbit IgG, washed again and incubated with avidin-biotinylated horseradish peroxidase complex (ABC) and 3,3'-diaminobenzidine tetrahydrochloride (DAB) (VECTASTAIN Elite ABC Kit; Vector Laboratories, Burlingame, CA, USA) and counterstained with Mayer's hematoxylin. Synovial tissue specimens isolated from patients with RA at the time of arthroscopic biopsy were stained with anti-human IL-17RD antibody (goat IgG, R&D), washed, incubated with biotinylated rabbit anti-goat IgG, washed again, incubated with ABC and DAB and counterstained with Mayer's hematoxylin. All patients gave their informed consent to participate, and the Institutional Medical Ethics Committee approved the study protocol (Application number:1492).

### Statistical analysis

All results are presented as the means ± S.E. The results from the various experimental groups and their corresponding controls were compared using unpaired t-tests. Differences with p-values < 0.05 were considered significant.

## Results

### MiRNA expression profile in the plasma of SKG mice

**Fig 2:**
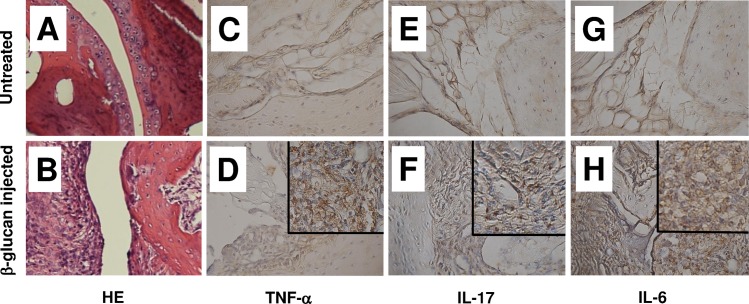
Histopathologic changes of swollen joints in SKG mice injected with ß-glucan. Histopathologic changes of swollen joints 30 days after ß-glucan injection showed severe synovial cell proliferation and infiltration of mononuclear cells with hematoxylin and eosin (HE) staining. (B). These pathologic changes were not observed in untreated SKG mice (A). Immunohistochemical staining showed high expression of TNF-α (D), IL-17 (F), and IL-6 (H) in the synovial tissue of ß-glucan injected SKG mice. In contrast, theses expression was not observed in the synovial tissue of untreated SKG mice (C, E, G). Original magnification, ×200; inset, ×400.

**Fig 3:**
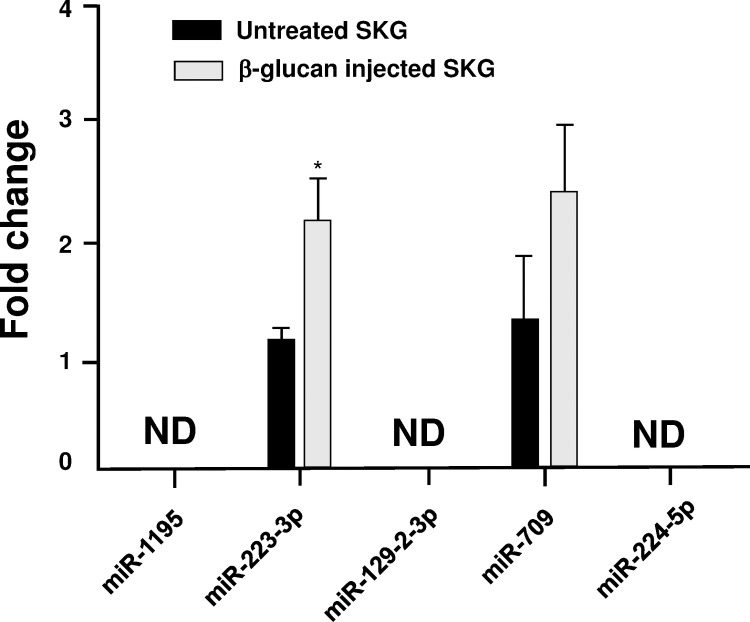
Quantitative real-time PCR analysis of the miRNAs in plasma from SKG mice. Five miRNAs (miR-1195, miR-223-3p, miR-129-2-3p, miR-709, and miR-224-5p), whose levels were found to be significantly elevated in the pooled plasma of ß-glucan-injected SKG mice by panel real-time PCR, were analyzed. Expression values were calculated by normalizing to miR-328-3p levels in each sample, and the expression levels were determined using the delta-delta Ct method. Results were expressed as the relative fold change of the levels of target miRNA compared with that of the control sample. Results are presented as the means ± S.E. for untreated (n = 5) and ß-glucan-injected (n = 5) SKG mice; ND: not determined; *p < 0.05.

SKG mice are RA model which spontaneously develop T cell–mediated chronic autoimmune arthritis [25–27]. Thirty days after injecting with ß-glucan, arthritis was induced in SKG mice (arthritis score 2 ± 0.8) but not in untreated SKG mice (arthritis score 0 ± 0.3). The histopathologic changes of swollen joints in SKG mice injected with ß-glucan by hematoxylin and eosin staining showed proliferation of synovial cells and infiltration of mononuclear cells which has been observed in human RA (Fig 2B) In contrast, these pathological changes were not observed in untreated SKG mice (Fig 2A). Immunohistochemical staining of synovial tissues from SKG mice injected with ß-glucan revealed high expression of TNF-α, IL-17, and IL-6 in synovial cells and infiltrating mononuclear cells (Fig 2D, 2F and 2H). We compared miRNA expression in the pooled plasma from SKG mice injected with ß-glucan (n = 5) with that in untreated SKG mice (n = 5) using panel real-time PCR analysis. We identified 17 upregulated miRNAs (fold change > 2.0) and 61 downregulated miRNAs (fold change < 0.5) in SKG mice injected with ß-glucan relative to untreated SKG mice (S1 Table). The top five upregulated miRNAs (miR-1195, miR-223-3p, miR-129-2-3p, miR-709, and miR-224-5p) were selected for subsequent analysis, which was performed via quantitative real-time PCR analysis of the plasma from individual mice in each group. Quantitative real-time PCR analysis of the plasma showed upregulation of miR-223-3p and miR-709 in the plasma of SKG mice treated with ß-glucan relative to untreated SKG mice. Upregulation of miR-223-3p, but not of miR-709, was statistically significant. We were unable to detect miR-1195, miR-129-2-3p, and miR-224-5p in the plasma of mice in either group (Fig 3).

### Prediction of miRNA target genes

In order to predict the target genes of upregulated miR-223-3p in SKG mice treated with ß-glucan, we used five different miRNA target prediction algorithms (miRWalk, DIANA-microT, PITA, RNAhybrid, Targetscan) and cross-evaluated the output from these algorithms to identify target mRNAs with greater accuracy. The bioinformatics approach identified 1,115 mRNAs as commonly predicted target genes from miR-223-3p by all the algorisms (S2 Table). Among them, we selected *Il17rd* because IL-17 signaling is well-known to be linked to the pathogenesis of inflammatory diseases and autoimmunity.

### IL-17rd is a target gene of miR-223-3p

**Fig 4:**
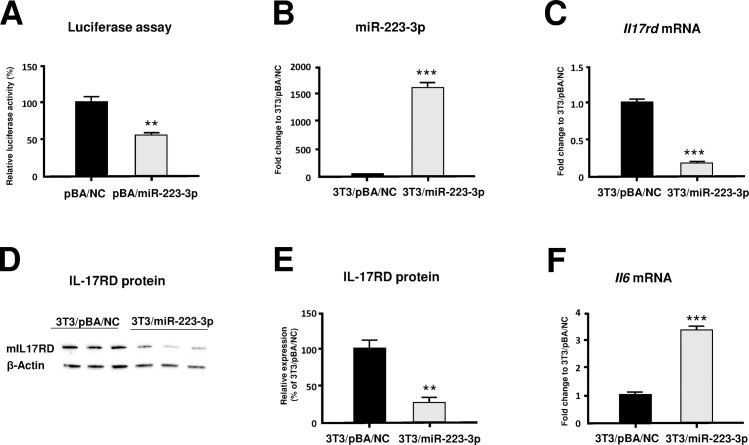
Effects of miR-223-3p overexpression on the mouse cells. (A) Results of luciferase assay. Reporter plasmid and mouse pmG/mIl17rd were transfected into HeLa cells with either miR-223-3p overexpression vector (pBA/miR-223-3p) or negative control vector (pBA/NC). Results were expressed as the relative luciferase activity following transfection of the negative control vector. **p < 0.01. (B) Expression of miR-223-3p in miR-223-3p overexpression NIH3T3 cells. Results were expressed as the relative fold change following transfection of cells with the negative control vector. ***p < 0.001. (C) Expression of *Il17rd* mRNA in miR-223-3p overexpression NIH3T3 cells. Results were expressed as the relative fold change following transfection with the negative control vector. ***p < 0.001. (D) Results of western blot analysis. (E) Relative expression of IL-17RD protein. IL17RD expression was normalized to that of β-actin in the same sample, and results were expressed as a percentage of the expression following transfection with the negative control vector. **p < 0.01. (F) Expression of *Il6* mRNA in miR-223-3p overexpression NIH3T3 cells. Results were expressed as the relative fold change following transfection of cells with the negative control vector. ***p < 0.001. All results are presented as the means ± S.E. for each group (n = 3).

In order to confirm whether miR-223-3p interacts with *Il17rd* mRNA, we used luciferase reporter plasmid vector driven by pmG/mIl17rd. This reporter plasmid vector was transfected into HeLa cells, together with either the miR-223-3p overexpression plasmid vector (pBA/miR-223-3p) or the negative control vector (pBA/NC), to compare the effects of transfection with pBA/miR-223-3p and pBA/NC on luciferase activity. As shown in Fig 4A, transfection with pBA/miR-223-3p significantly inhibited luciferase activity relative to transfection with pBA/NC (Fig 4A). This result indicates that *Il17rd* is a target gene of miR-223-3p.

### MiR-223-3p downregulates IL-17rd expression

To determine the functional role of miR-223-3p in the expression of *Il17rd*, we transduced miR-223-3p overexpression vector into NIH3T3 cells that constitutively expressed IL17RD (Fig 1A). The expression of miR-223-3p was significantly increased in NIH3T3 clone#13 transfected with pBA/miR-223-3p (Fig 4B). In contrast, the expression of *Il17rd* mRNA was significantly decreased in NIH3T3 clone#13 than that in NIH3T3 clone transfected with pBA/NC (Fig 4C). Next, we examined the effect of miR223-3p on IL-17RD expression at the protein level. As shown in Fig 4D, western blot analysis indicated that IL-17RD protein expression in NIH3T3 clone#13 was decreased than that in NIH3T3 clone transfected with pBA/NC (Fig 4D and 4E). These results indicate that miR-223-3p interacts with *Il17rd* mRNA, downregulating Il-17RD protein expression in NIH3T3 cells.

### MiR-223-3p augments IL-6 expression

We next examined the effect of miR-223-3p on IL-6 expression in NIH3T3 cells. Interestingly, NIH3T3 clone#13 induced significant levels of *Il6* mRNA expression (Fig 4F).

### IL-17rd is a target gene of miR-223-3p in humans

**Fig 5:**
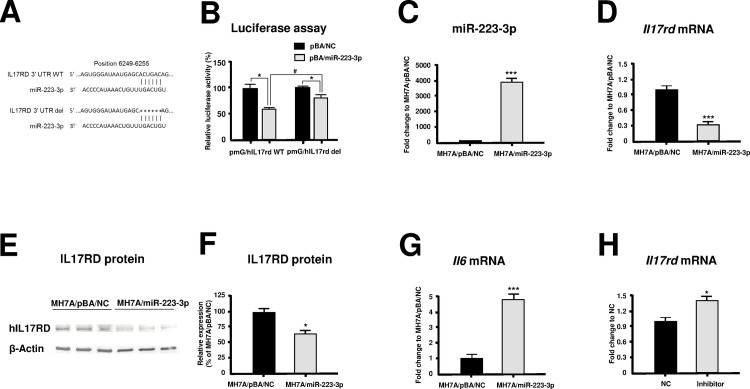
Effects of miR-223-3p overexpression on the human cells. (A) Schematic diagram of the luciferase reporter plasmids including IL-17RD3’UTR wild type (WT) or deleted (del) miR-223-3p binding sequences in the 3’UTR in which six nucleotide forming the seed region of miR-223-3p were deleted. (B) Results of luciferase assay. Either pmG/*hIl17rd* WT or pmG*/hIl17rd* del was transfected into HeLa cells with either miR-223-3p overexpression vector (pBA/miR-223-3p) or negative control vector (pBA/NC). Results were expressed as the relative luciferase activity following transfection of cells with the negative control vector. *p<0.05 as compared with the pBA/NC group, #p<0.05 as compared with the pmG/*hIL17rd* WT group. (C) Results of miR-223-3p expression in MH7A cells. Results were expressed as the relative fold change following transfection of cells with the negative control vector. ***p < 0.001. (D) Results of *Il17rd* mRNA expression in MH7A cells. Results were expressed as the relative fold change following transfection of the negative control vector; ***p < 0.001. (E) Results of western blot analysis. (F) Relative expression of IL-17RD protein. IL17RD expression was normalized to that of β-actin in the same sample, and results were expressed as a percentage of the expression following transfection with the negative control vector. *p < 0.05. (G) Results of *Il6* mRNA expression in MH7A cells. Results were expressed as the relative fold change following transfection with the negative control vector. ***p < 0.001. (H) Effect of miR-223-3p inhibitor on *Il17rd* mRNA expression. Results were expressed as the relative fold change with the negative control (NC). *p < 0.05. All results are presented as the means ± S.E. for each group (n = 3).

The sequence of human miR-223-3p is the same as that of mouse miR-223-3p. In order to determine whether miR-223-3p mediates IL-17RD expression in humans via direct interaction with its 3’UTR, we used bioinformatics software TargetScan to predict the potential binding sequences in the 3’UTR of *hIl17rd*. Then we designed pmirGLO luciferase reporter constructs containing either the wild-type (pmG/*hIl17rd* WT) or deleted (del) miR-223-3p binding sequences in the 3’UTR of *hIl17rd* (pmG/*hIl17rd* del) (Fig 5A). We transfected the luciferase reporter plasmid into HeLa cells, together with either pBA/miR-223-3p or pBA/NC. As shown in Fig 5B, co-transfection with pmG/*hIl17rd* WT and pBA/miR-223-3p showed a significant decrease of the luciferase activity relative to transfection with pBA/NC. By contrast, the inhibitory effect of miR-223-3p on luciferase activity was partially restored by the deletions of binding sequences in the 3’UTR of *hIl17rd* (Fig 5B). To confirm the relationship between miR-223-3p and IL17-RD expression in the synovial cells, we transfected miR-223-3p into the human synovial cell line MH7A (Fig 1B). Transfection with miR-223-3p-expressing clone#7 resulted in increased miR-223-3p mRNA expression (Fig 5C) but decreased *Il17rd* mRNA expression (Fig 5D) in MH7A cells. Next, we examined the effect of miR223-3p on IL-17RD expression at the protein level. As shown in Fig 5E, western blot analysis indicated that IL-17RD protein expression in MH7A clone#7 was decreased than that in MH7A clone transfected with pBA/NC (Fig 5E and 5F). In contrast, transfection with miR-223-3p-expressing clone#7 upregulated *Il6* mRNA expression in these cells (Fig 5G). Next, we examined the effect of miR-223-3p inhibitor on *Il17rd* mRNA expression in MH7A clone#7. As shown in Fig 5H, *Il17rd* mRNA expression was increased by the treatment of miR223-3p inhibitor (Fig 5H). Our results indicated that expression of miR-223-3p was inversely correlated to that of IL-17RD in synovial cells.

### TNF-α induces miR-223-3p and IL-6 expression in the human synovial cell line

**Fig 6:**
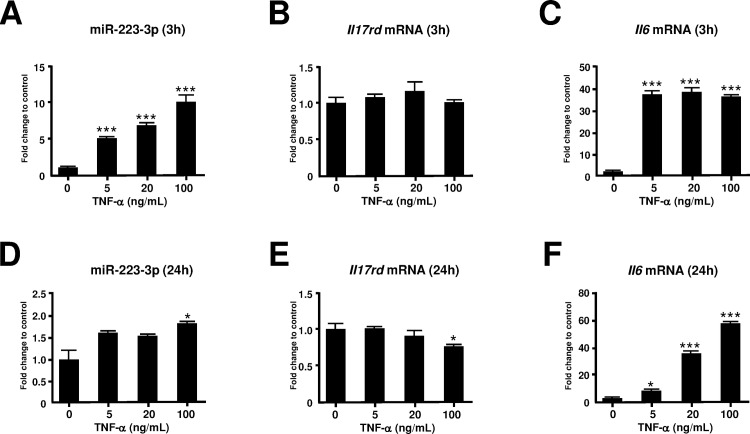
Effect of TNF-α on the expression of miR-223-3p, Il17rd, and Il6 mRNA in MH7A cells. MH7A cells were stimulated with various concentrations of TNF-α and the expression levels of miR-223-3p (A, D) *Il17rd* (B, E), and *Il6* mRNA (C, F) were analyzed after 3 h (A, B, C) and 24 h (D, E, F). Results were expressed as the relative fold change in the expression levels of these RNAs in the untreated control. *p < 0.05, ***p < 0.001. All results are presented as the means ± S.E. for each group (n = 3).

**Fig 7:**
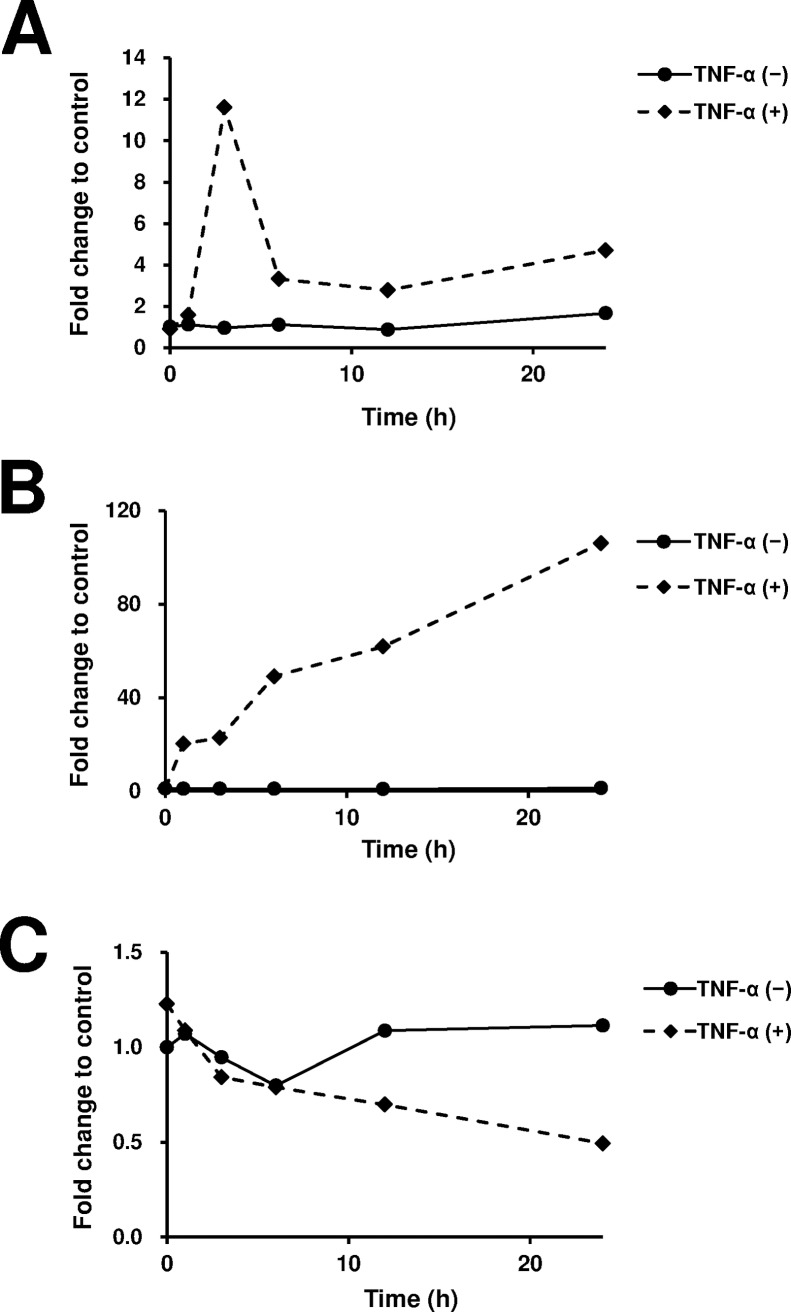
Kinetics of the expression of Il17rd, and Il6 mRNA and miR-223-3p after TNF-α stimulation. MH7A cells were stimulated with TNF-α (100 ng/ml) and the expression levels of miR-223-3p (A), *Il6* (B) and *Il17rd* mRNA (C) in MH7A cells were analyzed after 3, 6, 12 and 24 h. Results were expressed as the relative fold change in the expression levels of these RNAs in the untreated control.

In order to examine the effect of different diose of TNF-α on miR-223-3p expression in synovial cells, we stimulated MH7A cells with TNF-α. The expression of miR-223-3p was significantly augmented at 3 h, but not at 24 h, after TNF-α stimulation (Fig 6A and 6D). *Il6* mRNA expression was augmented at both 3 h and 24 h after TNF-α stimulation (Fig 6C and 6F). No change in *Il17rd* expression was observed at 3 h; however, suppression of *Il17rd* expression occurred at 24 h after TNF-α (100 ng/ml) stimulation (Fig 6B and 6E). We next examined the kinetics of miR-223-3p, *Il6*, and *Il17rd* mRNA expression after TNF-α (100 ng/ml) stimulation. As shown Fig 7, miR-223-3p transiently upregulated at 3h but downregulated at 6, 12, and 24h after TNF-α stimulation. In contrast, *Il-17rd* mRNA was progressively downregulated until 24h after TNF-α stimulation.

### IL-17RD expression inversely correlates to the severity of arthritis

**Fig 8:**
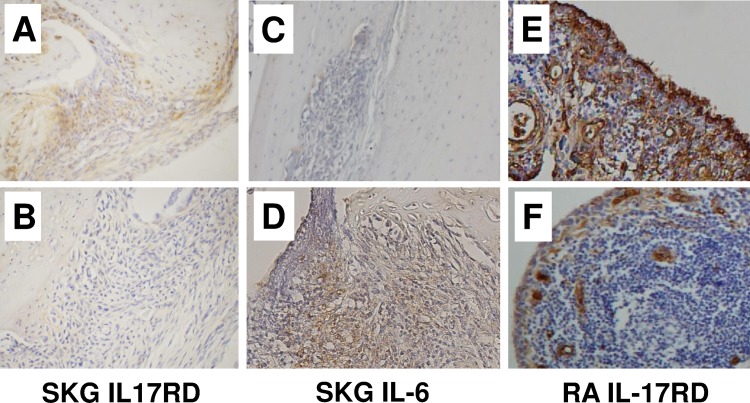
IL-17RD expression in synovial tissues from SKG mice and human RA. The expression of IL-17RD in synovial cells by SKG mice with mild arthritis (arthritis score:0.5) (A) was higher than that by SKG mice with severe arthritis (arthritis score:2.5) (B). The expression of IL-6 in synovial tissues from SKG mice with mild arthritis (C) was lower than that from SKG mice with severe arthritis (D).The expression of IL-17RD in synovial tissues from RA patients with mild arthritis (stage 2) (E) was higher than that from RA patients with severe arthritis (stage 4) (F). Original magnification, ×200.

In order to examine the correlation between IL-17RD expression and the severity of arthritis, we analyzed immunohistochemical staining of IL-17RD in synovial tissues from ß-glucan injected SKG mice and human RA patients. IL-17RD expression in synovial tissues from SKG mice with mild arthritis (arthritis score: 0.5) (Fig 8A) was higher than that from SKG mice with severe arthritis (arthritis score: 2.5) (Fig 8B). In contrast, IL-6 expression in synovial tissues from SKG mice with mild arthritis (Fig 8C) was lower than that from SKG mice with severe arthritis (Fig 8D). In addition, IL-17RD expression in synovial tissues with mild arthritis (stage 2 RA patient) (Fig 8E) was higher than that with severe arthritis (stage 4 RA patient) (Fig 8F), indicating the inverse correlation between IL-17RD expression and the severity of arthritis.

## Discussion

We selected *Il17rd* as a candidate target gene of miR-223-3p by analysis of post-transcriptional miRNA-mRNA interaction networks using miRNA prediction algorithms and demonstrated that miRNA-223-3p downregulated *Il17rd* mRNA expression and IL-17RD protein levels and upregulated *Il6* mRNA expression in both mouse and human synovial cells.

The IL-17 cytokine family includes IL-17A-F, which are predicted to form homo- and heterodimeric interactions that are necessary for signaling. IL-17 is associated with the pathogenesis of numerous autoimmune and inflammatory conditions such as RA, multiple sclerosis, psoriasis, Crohn’s disease, and systemic lupus erythematosus [32, 33]. High levels of IL-17 in the rheumatoid synovium of patients with RA have been reported to promote both inflammation and bone degradation [34]. Type 17 helper (Th17) cells that produce IL-17 induce the release of pro-inflammatory cytokines, such as TNF-α, IL-1β, and IL-6, from cartilage, synovial cells, macrophages, and bone cells. Th17 cells upregulate the production of RANK ligand in osteoblasts, thereby stimulating the activity of matrix metalloproteinases [35, 36]. There are five known IL-17 receptor subunits, namely IL-17RA-E. The *Il17rd* gene encodes a transmembrane protein of the IL-17RD; however, its ligand remains unidentified [37].

IL-17RD was identified as a novel IL-17 receptor-like protein which appears to be a human homologue of a Sef (similar expression of fgf genes) discovered in zebrafish gene [38, 39]. IL-17RD negatively regulates Ras and MAPK signaling induced by FGF and ERK and PI3K signaling induced by tyrosine kinase stimulation [40–45]. We observed an increase in *Il6* mRNA expression, but a decrease in that of *Il17rd*, following transfection of mouse NIH3T3 cell line with the miR-223-3p expression vector. Mellett et al. showed that *Il17rd*-/- mice were more susceptible to Toll-like receptor (TLR)4- and TLR3-induced septic shock, suggesting a novel regulatory network involving the functional interplay of members of the IL-17RD and TLR families. They demonstrated that the intracellular domain of IL-17RD targeted Toll/interleukin-1 receptor adaptor proteins to inhibit TLR downstream signaling [46]. Fuchs et al. showed that IL-17RD is an inhibitor of inflammatory cytokine signaling acting by cytoplasmic sequestration of the NF-kB after IL-1β- and TNF-α-induced IkB degradation [47]. Although the correlations between IL17RD and IL-6 expression are still not known, our observations support these previous findings, confirming that miR-223-3p contributes to functional interplay between IL-17RD, inflammatory cytokines, and TLR families.

The sequence of human miR-223-3p is the same as that of mouse miR-223-3p. In order to confirm the functional role of miR-223-3p in post-transcriptional miRNA-mRNA interaction in human synovial cells, we transfected IL-17RD-expressing human synovial cells (MH7A) with miR-223-3p plasmids, and showed that transfection with these plasmids resulted in decreased *Il17rd* expression and increased *Il6* mRNA expression in MH7A cells. We additionally observed that TNF-α stimulation increased miR-223-3p and *Il6* mRNA levels but decreased *Il17rd* mRNA expression in MH7A cells. miR-223-3p was shown to be overexpressed in synovial tissue of patients with RA [19], and silencing of miR-223-3p expression was found to reduce disease severity in experimental arthritis [48]. miRNA-223-3p was also reported to be a biomarker of disease activity in RA [49]. Our observations support previous reports demonstrating that miR-223-3p is involved in the pathogenesis of RA in humans as well as in animal models [48, 49].

## Conclusions

We observed that plasma levels of miR-223-3p are elevated in RA model of SKG mice, and that miR-223-3p downregulated IL-17RD but upregulated *Il6* expression in mouse NIH3T3 cells. We also observed miR-223-3p downregulated IL-17RD but upregulated *Il6* expression in human MH7A cell, and that TNF-α stimulation increased miR-223-3p and *Il6* mRNA levels but decreased *Il17rd* mRNA expression in MH7A cells. miR-223-3p may contribute the pathogenesis of RA, representing a novel target for the development of therapies against this disease.

## Supporting Information

S1 Fig The Ct value of miR-328-3p in untreated SKG mice and that in ß-glucan-injected SKG mice. The Ct values of miR-328-3p in untreated SKG mice and that in ß-glucan-injected SKG mice were quantified. The Ct value of miR-328-3p in untreated SKG mice was 30.7 ± 0.85 and that in ß-glucan-injected SKG mice was 30.6 ± 1.20. Results are presented as the means ± S.E. for each group (n = 5).
(PDF)

S1 Table Panel real-time PCR analysis. MiRNA expressions in the pooled plasma from SKG mice injected with ß-glucan (n = 5) and untreated SKG mice (n = 5) were analyzed using panel real-time PCR analysis. All data was normalized to the average of assays detected in samples. 17 upregulated miRNAs (fold change >2.0) and 61 downregulated miRNAs (fold change <0.5) comparing to untreated SKG mice are shown.
(PDF)

S2 Table In silico Prediction Results. List of target gene for miR-223-3p from five miRNA target prediction algorithms.
(PDF)
