# Supplementary figures and images for: The Impact of MicroRNA-223-3p on IL-17 Receptor D Expression in Synovial Cells
Source: PLoS One. 2017 Jan 5;12(1):e0169702. doi: 10.1371/journal.pone.0169702 (PMC5215929; doi:10.1371/journal.pone.0169702)

Figure S1

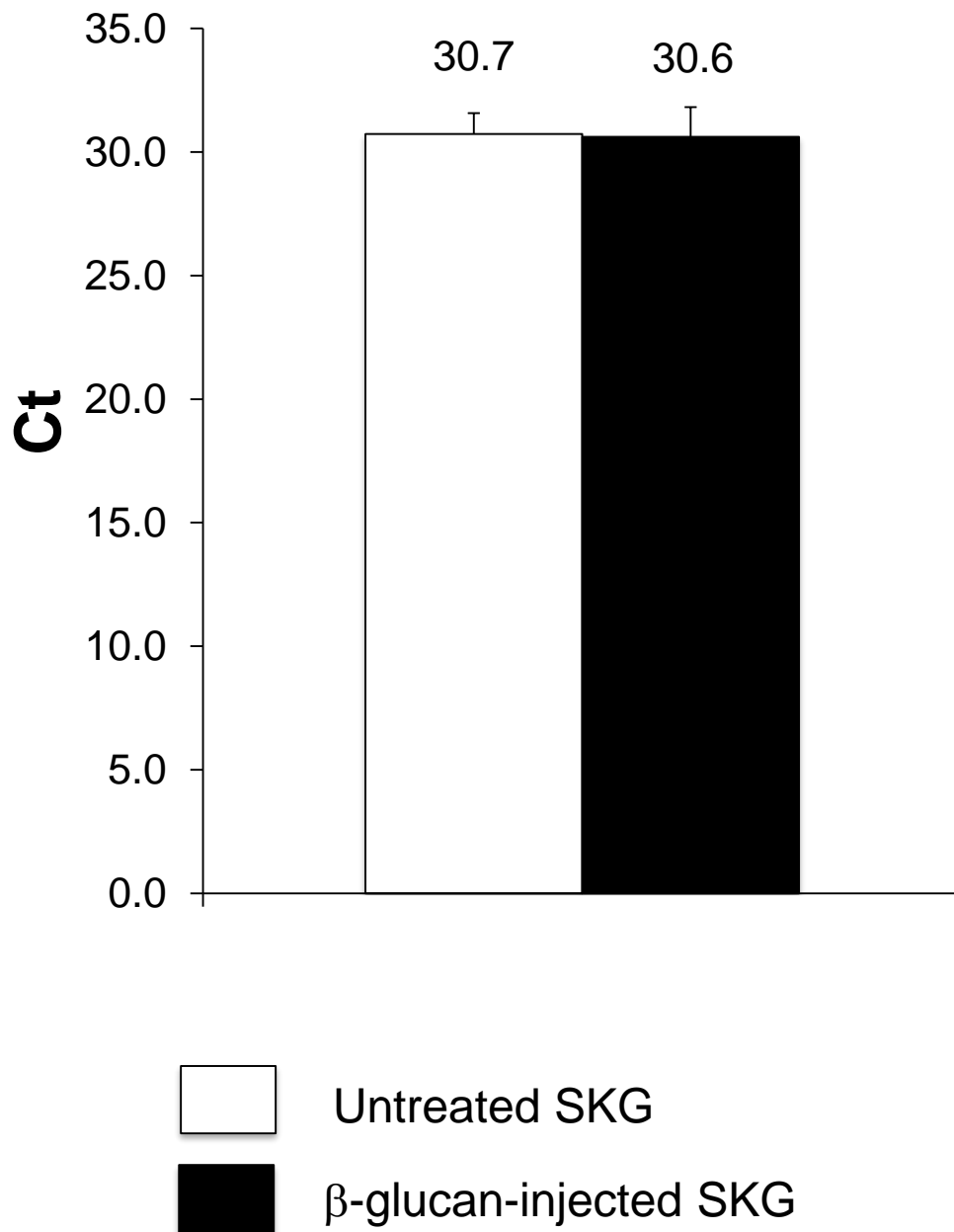

Supplement: S1 Fig — The Ct values of miR-328-3p in untreated SKG mice and that in ß-glucan-injected SKG mice were quantified. The Ct value of miR-328-3p in untreated SKG mice was 30.7 ± 0.85 and that in ß-glucan-injected SKG mice was 30.6 ± 1.20. Results are presented as the means ± S.E. for each group (n = 5). (PDF) [file pone.0169702.s001.pdf]
