# Supplementary material for: The Impact of MicroRNA-223-3p on IL-17 Receptor D Expression in Synovial Cells
Source: PLoS One. 2017 Jan 5;12(1):e0169702. doi: 10.1371/journal.pone.0169702 (PMC5215929; doi:10.1371/journal.pone.0169702)
Supplement: S1 Table — MiRNA expressions in the pooled plasma from SKG mice injected with ß-glucan (n = 5) and untreated SKG mice (n = 5) were analyzed using panel real-time PCR analysis. All data was normalized to the average of assays detected in samples. 17 upregulated miRNAs (fold change >2.0) and 61 downregulated miRNAs (fold change <0.5) comparing to untreated SKG mice are shown. (PDF) [file pone.0169702.s002.pdf]

Table S1. Panel real-time PCR analysis

	Target miRNA	Normalized Cp<br>Untreated	β-glucan injection	Fold change
UP	mmu-miR-1195	-2.91	-0.62	4.89
	mmu-miR-223-3p	8.17	10.22	4.14
	mmu-miR-129-2-3p	-3.09	-1.12	3.92
	mmu-miR-709	0.82	2.71	3.71
	mmu-miR-224-5p	-4.86	-3.18	3.21
	mmu-miR-34b-3p	-1.22	0.36	2.99
	mmu-miR-92b	-3.20	-1.65	2.93
	mmu-miR-342-3p	5.17	6.55	2.60
	mmu-miR-338-5p	-4.26	-2.90	2.57
	mmu-miR-425	-0.56	0.76	2.50
	mmu-miR-362-3p	-0.24	0.90	2.20
	mmu-miR-511-5p	-1.23	-0.10	2.19
	mmu-miR-139-5p	3.61	4.70	2.13
	mmu-miR-202-3p	-3.31	-2.22	2.13
	mmu-miR-23a	7.44	8.50	2.09
	mmu-miR-744	-3.98	-2.94	2.06
	mmu-miR-2137	-0.68	0.32	2.00
DOWN	mmu-miR-324-5p	1.16	0.11	0.48
	mmu-miR-1	4.95	3.90	0.48
	mmu-miR-676	2.82	1.75	0.48
	mmu-miR-300	-2.96	-4.05	0.47
	mmu-miR-29b	0.66	-0.45	0.46
	mmu-miR-486	10.22	9.11	0.46
	mmu-miR-26a	2.24	1.12	0.46
	mmu-miR-34a	-0.44	-1.56	0.46
	mmu-miR-652	1.85	0.71	0.45
	mmu-miR-142-3p	4.12	2.97	0.45
	mmu-miR-214	-1.78	-2.95	0.44
	mmu-miR-200c	1.94	0.76	0.44
	mmu-miR-15b	-1.46	-2.65	0.44
	mmu-let-7b	5.88	4.63	0.42
	mmu-miR-218	0.19	-1.06	0.42
	mmu-miR-143	3.29	2.03	0.42
	mmu-miR-140	3.28	2.02	0.42
	mmu-miR-31	0.49	-0.82	0.40
	mmu-let-7c	3.78	2.46	0.40
	mmu-miR-345-5p	0.32	-1.00	0.40
	mmu-miR-22	-2.83	-4.16	0.40
	mmu-miR-301a	-0.01	-1.37	0.39
	mmu-miR-17	2.33	0.95	0.38
	mmu-miR-222	6.44	5.04	0.38
	mmu-miR-30a	4.00	2.58	0.37
	mmu-miR-30e	1.74	0.32	0.37
	mmu-miR-125b-5p	6.95	5.52	0.37
	mmu-miR-872	-2.46	-3.92	0.36
	mmu-miR-1944	1.11	-0.39	0.35
	mmu-miR-434-5p	-0.73	-2.28	0.34
	mmu-miR-497	1.36	-0.19	0.34
	mmu-miR-106b	-2.27	-3.87	0.33
	mmu-miR-16-1	-1.67	-3.27	0.33
	mmu-miR-101a	4.17	2.55	0.33
	mmu-miR-18a	1.78	0.15	0.32
	mmu-miR-486	0.29	-1.40	0.31
	mmu-miR-93	8.98	7.27	0.31
	mmu-let-7i	3.25	1.51	0.30
	mmu-miR-107	1.98	0.24	0.30
	mmu-miR-331-3p	0.10	-1.73	0.28
	mmu-miR-374	-0.83	-2.66	0.28
	mmu-miR-26b	0.76	-1.10	0.28
	mmu-miR-15a	4.99	3.12	0.27
	mmu-miR-101b	3.41	1.54	0.27
	mmu-miR-19a	1.36	-0.51	0.27
	mmu-miR-20a	7.90	6.00	0.27
	mmu-miR-148b	2.15	0.22	0.26
	mmu-miR-16	10.39	8.44	0.26
	mmu-let-7g	6.04	4.06	0.25
	mmu-miR-365	3.21	1.16	0.24
	mmu-miR-421	1.61	-0.45	0.24
	mmu-miR-181c	-1.63	-3.79	0.22
	mmu-miR-106b	2.13	-0.05	0.22
	mmu-miR-106a	1.23	-1.02	0.21
	mmu-miR-103	3.31	0.97	0.20
	mmu-miR-186	3.71	1.33	0.19
	mmu-miR-144	7.63	5.19	0.18
	mmu-miR-32	0.43	-2.22	0.16
	mmu-miR-451	11.34	8.47	0.14
	mmu-miR-154	0.40	-2.73	0.11
	mmu-miR-199a-5p	-0.20	-3.78	0.08
