# Supplementary material for: The Impact of MicroRNA-223-3p on IL-17 Receptor D Expression in Synovial Cells
Source: PLoS One. 2017 Jan 5;12(1):e0169702. doi: 10.1371/journal.pone.0169702 (PMC5215929; doi:10.1371/journal.pone.0169702)
Supplement: S2 Table — List of target gene for miR-223-3p from five miRNA target prediction algorithms. (PDF) [file pone.0169702.s003.pdf]

Table S2. In silico Prediction Results
(List of target gene for mmu-miR-223-3p from five miRNA target prediction algorithms)

miRNA	EntrezID	Gene	miRWalk	DIANA-microT	PITA	RNA hybrid	Targets can	Total prediction algorithms
mmu-miR-223-3p	171463	Il17rd	1	1	1	1	1	5
mmu-miR-223-3p	11303	Abca1	1	1	1	1	1	5
mmu-miR-223-3p	11352	Abl2	1	1	1	1	1	5
mmu-miR-223-3p	11428	Aco1	1	1	1	1	1	5
mmu-miR-223-3p	11432	Acp2	1	1	1	1	1	5
mmu-miR-223-3p	11480	Acvr2a	1	1	1	1	1	5
mmu-miR-223-3p	11491	Adam17	1	1	1	1	1	5
mmu-miR-223-3p	11551	Adra2a	1	1	1	1	1	5
mmu-miR-223-3p	11593	Aga	1	1	1	1	1	5
mmu-miR-223-3p	11637	Ak2	1	1	1	1	1	5
mmu-miR-223-3p	11658	Alcam	1	1	1	1	1	5
mmu-miR-223-3p	11689	Alox5	1	1	1	1	1	5
mmu-miR-223-3p	11732	Ank	1	1	1	1	1	5
mmu-miR-223-3p	11736	Ankfy1	1	1	1	1	1	5
mmu-miR-223-3p	11758	Prdx6	1	1	1	1	1	5
mmu-miR-223-3p	11789	Apc	1	1	1	1	1	5
mmu-miR-223-3p	11798	Xiap	1	1	1	1	1	5
mmu-miR-223-3p	11799	Birc5	1	1	1	1	1	5
mmu-miR-223-3p	11842	Arf3	1	1	1	1	1	5
mmu-miR-223-3p	11852	Rhob	1	1	1	1	1	5
mmu-miR-223-3p	11877	Arvcf	1	1	1	1	1	5
mmu-miR-223-3p	11906	Zfhx3	1	1	1	1	1	5
mmu-miR-223-3p	11931	Atp1b1	1	1	1	1	1	5
mmu-miR-223-3p	11937	Atp2a1	1	1	1	1	1	5
mmu-miR-223-3p	11972	Atp6v0d1	1	1	1	1	1	5
mmu-miR-223-3p	11990	Atrn	1	1	1	1	1	5
mmu-miR-223-3p	12006	Axin2	1	1	1	1	1	5
mmu-miR-223-3p	12021	Bard1	1	1	1	1	1	5
mmu-miR-223-3p	12033	Bcap29	1	1	1	1	1	5
mmu-miR-223-3p	12038	Bche	1	1	1	1	1	5
mmu-miR-223-3p	12042	Bcl10	1	1	1	1	1	5
mmu-miR-223-3p	12142	Prdm1	1	1	1	1	1	5
mmu-miR-223-3p	12223	Btc	1	1	1	1	1	5
mmu-miR-223-3p	12326	Camk4	1	1	1	1	1	5
mmu-miR-223-3p	12373	Casq2	1	1	1	1	1	5
mmu-miR-223-3p	12374	Casr	1	1	1	1	1	5
mmu-miR-223-3p	12444	Ccnd2	1	1	1	1	1	5
mmu-miR-223-3p	12488	Cd2ap	1	1	1	1	1	5
mmu-miR-223-3p	12515	Cd69	1	1	1	1	1	5
mmu-miR-223-3p	12525	Cd8a	1	1	1	1	1	5
mmu-miR-223-3p	12552	Cdh11	1	1	1	1	1	5
mmu-miR-223-3p	12661	Chl1	1	1	1	1	1	5
mmu-miR-223-3p	12751	Tpp1	1	1	1	1	1	5
mmu-miR-223-3p	12768	Ccr1	1	1	1	1	1	5
mmu-miR-223-3p	12777	Ccr10	1	1	1	1	1	5
mmu-miR-223-3p	12801	Cnr1	1	1	1	1	1	5
mmu-miR-223-3p	12814	Col11a1	1	1	1	1	1	5
mmu-miR-223-3p	12823	Col19a1	1	1	1	1	1	5
mmu-miR-223-3p	12833	Col6a1	1	1	1	1	1	5
mmu-miR-223-3p	12837	Col8a1	1	1	1	1	1	5

mmu-miR-223-3p	12848	Cops2	1	1	1	1	1	5
mmu-miR-223-3p	12890	Cplx2	1	1	1	1	1	5
mmu-miR-223-3p	12950	Hapln1	1	1	1	1	1	5
mmu-miR-223-3p	12953	Cry2	1	1	1	1	1	5
mmu-miR-223-3p	12974	Cs	1	1	1	1	1	5
mmu-miR-223-3p	12994	Csn3	1	1	1	1	1	5
mmu-miR-223-3p	13039	Ctsl	1	1	1	1	1	5
mmu-miR-223-3p	13052	Cxadr	1	1	1	1	1	5
mmu-miR-223-3p	13063	Cycs	1	1	1	1	1	5
mmu-miR-223-3p	13081	Cyp24a1	1	1	1	1	1	5
mmu-miR-223-3p	13090	Cyp2b19	1	1	1	1	1	5
mmu-miR-223-3p	13138	Dag1	1	1	1	1	1	5
mmu-miR-223-3p	13171	Dbt	1	1	1	1	1	5
mmu-miR-223-3p	13196	Asap1	1	1	1	1	1	5
mmu-miR-223-3p	13199	Ddn	1	1	1	1	1	5
mmu-miR-223-3p	13200	Ddost	1	1	1	1	1	5
mmu-miR-223-3p	13445	Cdk2ap1	1	1	1	1	1	5
mmu-miR-223-3p	13481	Dpm2	1	1	1	1	1	5
mmu-miR-223-3p	13510	Dsg1a	1	1	1	1	1	5
mmu-miR-223-3p	13537	Dusp2	1	1	1	1	1	5
mmu-miR-223-3p	13555	E2f1	1	1	1	1	1	5
mmu-miR-223-3p	13589	Mapre1	1	1	1	1	1	5
mmu-miR-223-3p	13592	Ebf2	1	1	1	1	1	5
mmu-miR-223-3p	13605	Ect2	1	1	1	1	1	5
mmu-miR-223-3p	13608	Edar	1	1	1	1	1	5
mmu-miR-223-3p	13615	Edn2	1	1	1	1	1	5
mmu-miR-223-3p	13636	Efna1	1	1	1	1	1	5
mmu-miR-223-3p	13711	Elf5	1	1	1	1	1	5
mmu-miR-223-3p	13712	Elk1	1	1	1	1	1	5
mmu-miR-223-3p	13823	Epb4.1l3	1	1	1	1	1	5
mmu-miR-223-3p	13839	Epha5	1	1	1	1	1	5
mmu-miR-223-3p	14066	F3	1	1	1	1	1	5
mmu-miR-223-3p	14070	F8a	1	1	1	1	1	5
mmu-miR-223-3p	14071	F9	1	1	1	1	1	5
mmu-miR-223-3p	14083	Ptk2	1	1	1	1	1	5
mmu-miR-223-3p	14104	Fasn	1	1	1	1	1	5
mmu-miR-223-3p	14127	Fcer1g	1	1	1	1	1	5
mmu-miR-223-3p	14154	Fem1a	1	1	1	1	1	5
mmu-miR-223-3p	14160	Lgr5	1	1	1	1	1	5
mmu-miR-223-3p	14165	Fgf10	1	1	1	1	1	5
mmu-miR-223-3p	14205	Figf	1	1	1	1	1	5
mmu-miR-223-3p	14235	Foxm1	1	1	1	1	1	5
mmu-miR-223-3p	14262	Fmo3	1	1	1	1	1	5
mmu-miR-223-3p	14270	Srgap2	1	1	1	1	1	5
mmu-miR-223-3p	14347	Fut7	1	1	1	1	1	5
mmu-miR-223-3p	14348	Fut9	1	1	1	1	1	5
mmu-miR-223-3p	14373	G0s2	1	1	1	1	1	5
mmu-miR-223-3p	14378	G6pc2	1	1	1	1	1	5
mmu-miR-223-3p	14405	Gabrg1	1	1	1	1	1	5
mmu-miR-223-3p	14408	Gabrr1	1	1	1	1	1	5
mmu-miR-223-3p	14489	Mtpn	1	1	1	1	1	5
mmu-miR-223-3p	14545	Gdap1	1	1	1	1	1	5
mmu-miR-223-3p	14594	Ggta1	1	1	1	1	1	5
mmu-miR-223-3p	14633	Gli2	1	1	1	1	1	5
mmu-miR-223-3p	14667	Gm2a	1	1	1	1	1	5

mmu-miR-223-3p	14674	Gna13	1	1	1	1	1	5
mmu-miR-223-3p	14732	Gpam	1	1	1	1	1	5
mmu-miR-223-3p	14739	S1pr2	1	1	1	1	1	5
mmu-miR-223-3p	14939	Gzmb	1	1	1	1	1	5
mmu-miR-223-3p	14985	H2-M10.1	1	1	1	1	1	5
mmu-miR-223-3p	15002	H2-Ob	1	1	1	1	1	5
mmu-miR-223-3p	15042	H2-T24	1	1	1	1	1	5
mmu-miR-223-3p	15064	Mr1	1	1	1	1	1	5
mmu-miR-223-3p	15118	Has3	1	1	1	1	1	5
mmu-miR-223-3p	15242	Hhex	1	1	1	1	1	5
mmu-miR-223-3p	15245	Hhip	1	1	1	1	1	5
mmu-miR-223-3p	15257	Hipk1	1	1	1	1	1	5
mmu-miR-223-3p	15378	Hnf4a	1	1	1	1	1	5
mmu-miR-223-3p	15525	Hspa4	1	1	1	1	1	5
mmu-miR-223-3p	15528	Hspe1	1	1	1	1	1	5
mmu-miR-223-3p	15568	Elavl1	1	1	1	1	1	5
mmu-miR-223-3p	15945	Cxcl10	1	1	1	1	1	5
mmu-miR-223-3p	15979	Ifngr1	1	1	1	1	1	5
mmu-miR-223-3p	15985	Cd79b	1	1	1	1	1	5
mmu-miR-223-3p	16001	Igf1r	1	1	1	1	1	5
mmu-miR-223-3p	16011	Igfbp5	1	1	1	1	1	5
mmu-miR-223-3p	16150	Ikbkb	1	1	1	1	1	5
mmu-miR-223-3p	16195	Il6st	1	1	1	1	1	5
mmu-miR-223-3p	16210	Impact	1	1	1	1	1	5
mmu-miR-223-3p	16330	Inpp5b	1	1	1	1	1	5
mmu-miR-223-3p	16333	Ins1	1	1	1	1	1	5
mmu-miR-223-3p	16367	Irs1	1	1	1	1	1	5
mmu-miR-223-3p	16412	Itgb1	1	1	1	1	1	5
mmu-miR-223-3p	16423	Cd47	1	1	1	1	1	5
mmu-miR-223-3p	16438	Itpr1	1	1	1	1	1	5
mmu-miR-223-3p	16456	F11r	1	1	1	1	1	5
mmu-miR-223-3p	16467	Atcay	1	1	1	1	1	5
mmu-miR-223-3p	16490	Kcna2	1	1	1	1	1	5
mmu-miR-223-3p	16513	Kcnj10	1	1	1	1	1	5
mmu-miR-223-3p	16535	Kcnq1	1	1	1	1	1	5
mmu-miR-223-3p	16539	Kcns2	1	1	1	1	1	5
mmu-miR-223-3p	16561	Kif1b	1	1	1	1	1	5
mmu-miR-223-3p	16565	Kif21b	1	1	1	1	1	5
mmu-miR-223-3p	16596	Klf1	1	1	1	1	1	5
mmu-miR-223-3p	16601	Klf9	1	1	1	1	1	5
mmu-miR-223-3p	16646	Kpna1	1	1	1	1	1	5
mmu-miR-223-3p	16651	Sspn	1	1	1	1	1	5
mmu-miR-223-3p	16658	Mafb	1	1	1	1	1	5
mmu-miR-223-3p	16798	Lats1	1	1	1	1	1	5
mmu-miR-223-3p	16842	Lef1	1	1	1	1	1	5
mmu-miR-223-3p	16909	Lmo2	1	1	1	1	1	5
mmu-miR-223-3p	16923	Sh2b3	1	1	1	1	1	5
mmu-miR-223-3p	16969	Zbtb7a	1	1	1	1	1	5
mmu-miR-223-3p	17082	Il1rl1	1	1	1	1	1	5
mmu-miR-223-3p	17112	Tm4sf1	1	1	1	1	1	5
mmu-miR-223-3p	17132	Maf	1	1	1	1	1	5
mmu-miR-223-3p	17181	Matn2	1	1	1	1	1	5
mmu-miR-223-3p	17196	Mbp	1	1	1	1	1	5
mmu-miR-223-3p	17295	Met	1	1	1	1	1	5
mmu-miR-223-3p	17309	Mgat3	1	1	1	1	1	5

mmu-miR-223-3p	17347	Mknk2	1	1	1	1	1	5
mmu-miR-223-3p	17381	Mmp12	1	1	1	1	1	5
mmu-miR-223-3p	17528	Mpz	1	1	1	1	1	5
mmu-miR-223-3p	17532	Mras	1	1	1	1	1	5
mmu-miR-223-3p	17750	Mt2	1	1	1	1	1	5
mmu-miR-223-3p	17755	Map1b	1	1	1	1	1	5
mmu-miR-223-3p	17952	Naip6	1	1	1	1	1	5
mmu-miR-223-3p	17957	Napb	1	1	1	1	1	5
mmu-miR-223-3p	17977	Ncoa1	1	1	1	1	1	5
mmu-miR-223-3p	17979	Ncoa3	1	1	1	1	1	5
mmu-miR-223-3p	18005	Nek2	1	1	1	1	1	5
mmu-miR-223-3p	18014	Neurog1	1	1	1	1	1	5
mmu-miR-223-3p	18015	Nf1	1	1	1	1	1	5
mmu-miR-223-3p	18072	Nhlh2	1	1	1	1	1	5
mmu-miR-223-3p	18080	Nin	1	1	1	1	1	5
mmu-miR-223-3p	18104	Nqo1	1	1	1	1	1	5
mmu-miR-223-3p	18109	Mycn	1	1	1	1	1	5
mmu-miR-223-3p	18117	Emc8	1	1	1	1	1	5
mmu-miR-223-3p	18145	Npc1	1	1	1	1	1	5
mmu-miR-223-3p	18159	Nppc	1	1	1	1	1	5
mmu-miR-223-3p	18186	Nrp1	1	1	1	1	1	5
mmu-miR-223-3p	18189	Nrxn1	1	1	1	1	1	5
mmu-miR-223-3p	18193	Nsd1	1	1	1	1	1	5
mmu-miR-223-3p	18214	Ddr2	1	1	1	1	1	5
mmu-miR-223-3p	18232	Nxph2	1	1	1	1	1	5
mmu-miR-223-3p	18260	Ocln	1	1	1	1	1	5
mmu-miR-223-3p	18378	Omp	1	1	1	1	1	5
mmu-miR-223-3p	18383	Tnfrsf11b	1	1	1	1	1	5
mmu-miR-223-3p	18386	Oprd1	1	1	1	1	1	5
mmu-miR-223-3p	18413	Osm	1	1	1	1	1	5
mmu-miR-223-3p	18417	Cldn11	1	1	1	1	1	5
mmu-miR-223-3p	18472	Pafah1b1	1	1	1	1	1	5
mmu-miR-223-3p	18511	Pax9	1	1	1	1	1	5
mmu-miR-223-3p	18575	Pde1c	1	1	1	1	1	5
mmu-miR-223-3p	18582	Pde6d	1	1	1	1	1	5
mmu-miR-223-3p	18607	Pdpk1	1	1	1	1	1	5
mmu-miR-223-3p	18611	Pea15a	1	1	1	1	1	5
mmu-miR-223-3p	18616	Peg3	1	1	1	1	1	5
mmu-miR-223-3p	18647	Cdk14	1	1	1	1	1	5
mmu-miR-223-3p	18704	Pik3c2a	1	1	1	1	1	5
mmu-miR-223-3p	18710	Pik3r3	1	1	1	1	1	5
mmu-miR-223-3p	18738	Pitpna	1	1	1	1	1	5
mmu-miR-223-3p	18767	Pkia	1	1	1	1	1	5
mmu-miR-223-3p	18771	Pknox1	1	1	1	1	1	5
mmu-miR-223-3p	18782	Pla2g2d	1	1	1	1	1	5
mmu-miR-223-3p	18789	Papola	1	1	1	1	1	5
mmu-miR-223-3p	18813	Pa2g4	1	1	1	1	1	5
mmu-miR-223-3p	18815	Plg	1	1	1	1	1	5
mmu-miR-223-3p	18845	Plxna2	1	1	1	1	1	5
mmu-miR-223-3p	18938	Ppp1r14b	1	1	1	1	1	5
mmu-miR-223-3p	19046	Ppp1cb	1	1	1	1	1	5
mmu-miR-223-3p	19059	Ppp3r2	1	1	1	1	1	5
mmu-miR-223-3p	19087	Prkar2a	1	1	1	1	1	5
mmu-miR-223-3p	19122	Prnp	1	1	1	1	1	5
mmu-miR-223-3p	19225	Ptgs2	1	1	1	1	1	5

mmu-miR-223-3p	19244	Ptp4a2	1	1	1	1	1	5
mmu-miR-223-3p	19246	Ptpn1	1	1	1	1	1	5
mmu-miR-223-3p	19265	Ptprcap	1	1	1	1	1	5
mmu-miR-223-3p	19281	Ptprt	1	1	1	1	1	5
mmu-miR-223-3p	19317	Qk	1	1	1	1	1	5
mmu-miR-223-3p	19325	Rab10	1	1	1	1	1	5
mmu-miR-223-3p	19340	Rab3d	1	1	1	1	1	5
mmu-miR-223-3p	19378	Aldh1a2	1	1	1	1	1	5
mmu-miR-223-3p	19419	Rasgrp1	1	1	1	1	1	5
mmu-miR-223-3p	19645	Rb1	1	1	1	1	1	5
mmu-miR-223-3p	19672	Rcn1	1	1	1	1	1	5
mmu-miR-223-3p	19699	Reln	1	1	1	1	1	5
mmu-miR-223-3p	19708	Dpf2	1	1	1	1	1	5
mmu-miR-223-3p	19735	Rgs2	1	1	1	1	1	5
mmu-miR-223-3p	19736	Rgs4	1	1	1	1	1	5
mmu-miR-223-3p	19766	Ripk1	1	1	1	1	1	5
mmu-miR-223-3p	19775	Xpr1	1	1	1	1	1	5
mmu-miR-223-3p	19822	Rnf4	1	1	1	1	1	5
mmu-miR-223-3p	19885	Rorc	1	1	1	1	1	5
mmu-miR-223-3p	20135	Rrm2	1	1	1	1	1	5
mmu-miR-223-3p	20220	Sap18	1	1	1	1	1	5
mmu-miR-223-3p	20249	Scd1	1	1	1	1	1	5
mmu-miR-223-3p	20265	Scn1a	1	1	1	1	1	5
mmu-miR-223-3p	20269	Scn3a	1	1	1	1	1	5
mmu-miR-223-3p	20272	Scn7a	1	1	1	1	1	5
mmu-miR-223-3p	20280	Scp2	1	1	1	1	1	5
mmu-miR-223-3p	20300	Ccl25	1	1	1	1	1	5
mmu-miR-223-3p	20302	Ccl3	1	1	1	1	1	5
mmu-miR-223-3p	20312	Cx3cl1	1	1	1	1	1	5
mmu-miR-223-3p	20315	Cxcl12	1	1	1	1	1	5
mmu-miR-223-3p	20334	Sec23a	1	1	1	1	1	5
mmu-miR-223-3p	20340	Glg1	1	1	1	1	1	5
mmu-miR-223-3p	20362	Sept8	1	1	1	1	1	5
mmu-miR-223-3p	20437	Siah1a	1	1	1	1	1	5
mmu-miR-223-3p	20451	St8sia3	1	1	1	1	1	5
mmu-miR-223-3p	20538	Slc6a2	1	1	1	1	1	5
mmu-miR-223-3p	20583	Snai2	1	1	1	1	1	5
mmu-miR-223-3p	20585	Hltf	1	1	1	1	1	5
mmu-miR-223-3p	20614	Snap25	1	1	1	1	1	5
mmu-miR-223-3p	20621	Snn	1	1	1	1	1	5
mmu-miR-223-3p	20623	Snrk	1	1	1	1	1	5
mmu-miR-223-3p	20650	Sntb2	1	1	1	1	1	5
mmu-miR-223-3p	20723	Serpinb9	1	1	1	1	1	5
mmu-miR-223-3p	20811	Srms	1	1	1	1	1	5
mmu-miR-223-3p	20817	Srpk2	1	1	1	1	1	5
mmu-miR-223-3p	20822	Trove2	1	1	1	1	1	5
mmu-miR-223-3p	20840	Stac	1	1	1	1	1	5
mmu-miR-223-3p	20844	Stam	1	1	1	1	1	5
mmu-miR-223-3p	20866	Stim1	1	1	1	1	1	5
mmu-miR-223-3p	20893	Bhlhe40	1	1	1	1	1	5
mmu-miR-223-3p	20972	Syngr1	1	1	1	1	1	5
mmu-miR-223-3p	20983	Syt4	1	1	1	1	1	5
mmu-miR-223-3p	21417	Zeb1	1	1	1	1	1	5
mmu-miR-223-3p	21422	Tfcp2	1	1	1	1	1	5
mmu-miR-223-3p	21766	Tex261	1	1	1	1	1	5

mmu-miR-223-3p	21780	Tfam	1	1	1	1	1	5
mmu-miR-223-3p	21814	Tgfbr3	1	1	1	1	1	5
mmu-miR-223-3p	21822	Tgtp1	1	1	1	1	1	5
mmu-miR-223-3p	21841	Tia1	1	1	1	1	1	5
mmu-miR-223-3p	21843	Tial1	1	1	1	1	1	5
mmu-miR-223-3p	21885	Tle1	1	1	1	1	1	5
mmu-miR-223-3p	21898	Tlr4	1	1	1	1	1	5
mmu-miR-223-3p	21912	Tspan7	1	1	1	1	1	5
mmu-miR-223-3p	22030	Traf2	1	1	1	1	1	5
mmu-miR-223-3p	22034	Traf6	1	1	1	1	1	5
mmu-miR-223-3p	22045	Trhr	1	1	1	1	1	5
mmu-miR-223-3p	22065	Trpc3	1	1	1	1	1	5
mmu-miR-223-3p	22095	Tshr	1	1	1	1	1	5
mmu-miR-223-3p	22099	Tsn	1	1	1	1	1	5
mmu-miR-223-3p	22152	Tubb3	1	1	1	1	1	5
mmu-miR-223-3p	22158	Tulp3	1	1	1	1	1	5
mmu-miR-223-3p	22209	Ube2a	1	1	1	1	1	5
mmu-miR-223-3p	22224	Usp10	1	1	1	1	1	5
mmu-miR-223-3p	22253	Unc5c	1	1	1	1	1	5
mmu-miR-223-3p	22278	Usf1	1	1	1	1	1	5
mmu-miR-223-3p	22352	Vim	1	1	1	1	1	5
mmu-miR-223-3p	22402	Wisp1	1	1	1	1	1	5
mmu-miR-223-3p	22445	Xlr3a	1	1	1	1	1	5
mmu-miR-223-3p	22446	Xlr3c	1	1	1	1	1	5
mmu-miR-223-3p	22591	Xpc	1	1	1	1	1	5
mmu-miR-223-3p	22661	Zfp148	1	1	1	1	1	5
mmu-miR-223-3p	22671	Rnf112	1	1	1	1	1	5
mmu-miR-223-3p	22687	Zfp259	1	1	1	1	1	5
mmu-miR-223-3p	22688	Zfp26	1	1	1	1	1	5
mmu-miR-223-3p	22700	Zfp40	1	1	1	1	1	5
mmu-miR-223-3p	22774	Zic4	1	1	1	1	1	5
mmu-miR-223-3p	23797	Akt3	1	1	1	1	1	5
mmu-miR-223-3p	23805	Apc2	1	1	1	1	1	5
mmu-miR-223-3p	23832	Xcr1	1	1	1	1	1	5
mmu-miR-223-3p	23879	Fxr2	1	1	1	1	1	5
mmu-miR-223-3p	23921	Sh2b2	1	1	1	1	1	5
mmu-miR-223-3p	23923	Aadat	1	1	1	1	1	5
mmu-miR-223-3p	23964	Tenm2	1	1	1	1	1	5
mmu-miR-223-3p	23965	Tenm3	1	1	1	1	1	5
mmu-miR-223-3p	23993	Klk7	1	1	1	1	1	5
mmu-miR-223-3p	24000	Ptpn21	1	1	1	1	1	5
mmu-miR-223-3p	24071	Synj2bp	1	1	1	1	1	5
mmu-miR-223-3p	24083	Gm16515	1	1	1	1	1	5
mmu-miR-223-3p	24136	Zeb2	1	1	1	1	1	5
mmu-miR-223-3p	26360	Angptl2	1	1	1	1	1	5
mmu-miR-223-3p	26371	Ciao1	1	1	1	1	1	5
mmu-miR-223-3p	26373	Clcn7	1	1	1	1	1	5
mmu-miR-223-3p	26405	Map3k2	1	1	1	1	1	5
mmu-miR-223-3p	26414	Mapk10	1	1	1	1	1	5
mmu-miR-223-3p	26428	Orc4	1	1	1	1	1	5
mmu-miR-223-3p	26434	Prnd	1	1	1	1	1	5
mmu-miR-223-3p	26443	Psma6	1	1	1	1	1	5
mmu-miR-223-3p	26557	Homer2	1	1	1	1	1	5
mmu-miR-223-3p	26611	Rcn2	1	1	1	1	1	5
mmu-miR-223-3p	26875	Pclo	1	1	1	1	1	5

mmu-miR-223-3p	26909	Exo1	1	1	1	1	1	5
mmu-miR-223-3p	26936	Mprip	1	1	1	1	1	5
mmu-miR-223-3p	26939	Polr3e	1	1	1	1	1	5
mmu-miR-223-3p	26965	Cul1	1	1	1	1	1	5
mmu-miR-223-3p	27055	Fkbp9	1	1	1	1	1	5
mmu-miR-223-3p	27218	Slamf1	1	1	1	1	1	5
mmu-miR-223-3p	27377	Yme1l1	1	1	1	1	1	5
mmu-miR-223-3p	27392	Pign	1	1	1	1	1	5
mmu-miR-223-3p	27801	Zdhhc8	1	1	1	1	1	5
mmu-miR-223-3p	28042	Ept1	1	1	1	1	1	5
mmu-miR-223-3p	29808	Mga	1	1	1	1	1	5
mmu-miR-223-3p	29809	Rabgap1l	1	1	1	1	1	5
mmu-miR-223-3p	29820	Tnfrsf19	1	1	1	1	1	5
mmu-miR-223-3p	29858	Pmm1	1	1	1	1	1	5
mmu-miR-223-3p	29863	Pde7b	1	1	1	1	1	5
mmu-miR-223-3p	29869	Ulk2	1	1	1	1	1	5
mmu-miR-223-3p	30791	Slc39a1	1	1	1	1	1	5
mmu-miR-223-3p	30932	Zfp330	1	1	1	1	1	5
mmu-miR-223-3p	30939	Pttg1	1	1	1	1	1	5
mmu-miR-223-3p	50753	Fbxo8	1	1	1	1	1	5
mmu-miR-223-3p	50785	Hs6st1	1	1	1	1	1	5
mmu-miR-223-3p	50926	Hnrnpdl	1	1	1	1	1	5
mmu-miR-223-3p	51886	Fubp1	1	1	1	1	1	5
mmu-miR-223-3p	52024	Ankrd22	1	1	1	1	1	5
mmu-miR-223-3p	52065	Mfhas1	1	1	1	1	1	5
mmu-miR-223-3p	52120	Hgsnat	1	1	1	1	1	5
mmu-miR-223-3p	52150	Kcnk6	1	1	1	1	1	5
mmu-miR-223-3p	52250	Reep1	1	1	1	1	1	5
mmu-miR-223-3p	52609	Cbx7	1	1	1	1	1	5
mmu-miR-223-3p	52850	Sgsm1	1	1	1	1	1	5
mmu-miR-223-3p	52882	Rgs7bp	1	1	1	1	1	5
mmu-miR-223-3p	52906	Ahi1	1	1	1	1	1	5
mmu-miR-223-3p	53310	Dlg3	1	1	1	1	1	5
mmu-miR-223-3p	53334	Gosr1	1	1	1	1	1	5
mmu-miR-223-3p	53412	Ppp1r3c	1	1	1	1	1	5
mmu-miR-223-3p	53416	Stk39	1	1	1	1	1	5
mmu-miR-223-3p	53422	Ybx2	1	1	1	1	1	5
mmu-miR-223-3p	53601	Pcdh12	1	1	1	1	1	5
mmu-miR-223-3p	53621	Cnot4	1	1	1	1	1	5
mmu-miR-223-3p	53623	Gria3	1	1	1	1	1	5
mmu-miR-223-3p	53945	Slc40a1	1	1	1	1	1	5
mmu-miR-223-3p	54130	Actr1a	1	1	1	1	1	5
mmu-miR-223-3p	54338	Slc23a2	1	1	1	1	1	5
mmu-miR-223-3p	54399	Bet1l	1	1	1	1	1	5
mmu-miR-223-3p	54403	Slc4a4	1	1	1	1	1	5
mmu-miR-223-3p	54418	Fmn2	1	1	1	1	1	5
mmu-miR-223-3p	54447	Asah2	1	1	1	1	1	5
mmu-miR-223-3p	54563	Nup210	1	1	1	1	1	5
mmu-miR-223-3p	54635	Pdgfc	1	1	1	1	1	5
mmu-miR-223-3p	54698	Crtam	1	1	1	1	1	5
mmu-miR-223-3p	54711	Plagl2	1	1	1	1	1	5
mmu-miR-223-3p	55935	Fnbp4	1	1	1	1	1	5
mmu-miR-223-3p	55992	Trim3	1	1	1	1	1	5
mmu-miR-223-3p	56041	Uso1	1	1	1	1	1	5
mmu-miR-223-3p	56050	Cyp39a1	1	1	1	1	1	5

mmu-miR-223-3p	56070	Tcerg1	1	1	1	1	1	5
mmu-miR-223-3p	56072	Lgals12	1	1	1	1	1	5
mmu-miR-223-3p	56093	Pfpl	1	1	1	1	1	5
mmu-miR-223-3p	56095	Ftsj3	1	1	1	1	1	5
mmu-miR-223-3p	56150	Mad2l1	1	1	1	1	1	5
mmu-miR-223-3p	56195	Ptbp2	1	1	1	1	1	5
mmu-miR-223-3p	56196	Tdp2	1	1	1	1	1	5
mmu-miR-223-3p	56217	Mpp5	1	1	1	1	1	5
mmu-miR-223-3p	56248	Ak3	1	1	1	1	1	5
mmu-miR-223-3p	56306	Fam60a	1	1	1	1	1	5
mmu-miR-223-3p	56314	Zfp113	1	1	1	1	1	5
mmu-miR-223-3p	56318	Acpp	1	1	1	1	1	5
mmu-miR-223-3p	56363	Tmeff2	1	1	1	1	1	5
mmu-miR-223-3p	56372	1110004F10Rik	1	1	1	1	1	5
mmu-miR-223-3p	56382	Rab9	1	1	1	1	1	5
mmu-miR-223-3p	56384	Letm1	1	1	1	1	1	5
mmu-miR-223-3p	56403	Syncrip	1	1	1	1	1	5
mmu-miR-223-3p	56484	Foxo3	1	1	1	1	1	5
mmu-miR-223-3p	56485	Slc2a5	1	1	1	1	1	5
mmu-miR-223-3p	56496	Tspan6	1	1	1	1	1	5
mmu-miR-223-3p	56626	Poll	1	1	1	1	1	5
mmu-miR-223-3p	56710	Brinp1	1	1	1	1	1	5
mmu-miR-223-3p	56752	Aldh9a1	1	1	1	1	1	5
mmu-miR-223-3p	56758	Mbnl1	1	1	1	1	1	5
mmu-miR-223-3p	56771	Med20	1	1	1	1	1	5
mmu-miR-223-3p	57745	Zfp112	1	1	1	1	1	5
mmu-miR-223-3p	57784	Bin3	1	1	1	1	1	5
mmu-miR-223-3p	57810	Cdon	1	1	1	1	1	5
mmu-miR-223-3p	57908	Zfp318	1	1	1	1	1	5
mmu-miR-223-3p	58200	Ppp1r1a	1	1	1	1	1	5
mmu-miR-223-3p	58205	Pdcd1lg2	1	1	1	1	1	5
mmu-miR-223-3p	58243	Nap1l5	1	1	1	1	1	5
mmu-miR-223-3p	58244	Stx6	1	1	1	1	1	5
mmu-miR-223-3p	58250	Chst11	1	1	1	1	1	5
mmu-miR-223-3p	58909	Fam13a	1	1	1	1	1	5
mmu-miR-223-3p	58994	Smpd3	1	1	1	1	1	5
mmu-miR-223-3p	59009	Sh3rf1	1	1	1	1	1	5
mmu-miR-223-3p	59012	Moxd1	1	1	1	1	1	5
mmu-miR-223-3p	59035	Carm1	1	1	1	1	1	5
mmu-miR-223-3p	59046	Arpp19	1	1	1	1	1	5
mmu-miR-223-3p	60505	Il21	1	1	1	1	1	5
mmu-miR-223-3p	63953	Dusp10	1	1	1	1	1	5
mmu-miR-223-3p	64138	Ctsz	1	1	1	1	1	5
mmu-miR-223-3p	64658	Mrps25	1	1	1	1	1	5
mmu-miR-223-3p	64705	Dpys	1	1	1	1	1	5
mmu-miR-223-3p	65246	Xpo7	1	1	1	1	1	5
mmu-miR-223-3p	65960	Twsg1	1	1	1	1	1	5
mmu-miR-223-3p	65973	Asph	1	1	1	1	1	5
mmu-miR-223-3p	66067	Gtpbp8	1	1	1	1	1	5
mmu-miR-223-3p	66113	Apoa5	1	1	1	1	1	5
mmu-miR-223-3p	66140	Ska2	1	1	1	1	1	5
mmu-miR-223-3p	66202	1110059G10Rik	1	1	1	1	1	5
mmu-miR-223-3p	66209	Inip	1	1	1	1	1	5
mmu-miR-223-3p	66257	Nicn1	1	1	1	1	1	5
mmu-miR-223-3p	66259	Camk2n1	1	1	1	1	1	5

mmu-miR-223-3p	66261	Tm4sf20	1	1	1	1	1	5
mmu-miR-223-3p	66270	Fam134b	1	1	1	1	1	5
mmu-miR-223-3p	66272	Cox16	1	1	1	1	1	5
mmu-miR-223-3p	66274	Lyrm9	1	1	1	1	1	5
mmu-miR-223-3p	66371	Chmp4c	1	1	1	1	1	5
mmu-miR-223-3p	66412	Arrdc4	1	1	1	1	1	5
mmu-miR-223-3p	66421	2410004B18Rik	1	1	1	1	1	5
mmu-miR-223-3p	66427	Cyb5b	1	1	1	1	1	5
mmu-miR-223-3p	66443	Tnfaip8l1	1	1	1	1	1	5
mmu-miR-223-3p	66480	Rpl15	1	1	1	1	1	5
mmu-miR-223-3p	66511	Chtop	1	1	1	1	1	5
mmu-miR-223-3p	66596	Gtf3a	1	1	1	1	1	5
mmu-miR-223-3p	66601	Tmigd1	1	1	1	1	1	5
mmu-miR-223-3p	66615	Atg4b	1	1	1	1	1	5
mmu-miR-223-3p	66646	Rpe	1	1	1	1	1	5
mmu-miR-223-3p	66689	Klhl28	1	1	1	1	1	5
mmu-miR-223-3p	66717	Ccdc96	1	1	1	1	1	5
mmu-miR-223-3p	66729	Ankrd61	1	1	1	1	1	5
mmu-miR-223-3p	66733	Kcng4	1	1	1	1	1	5
mmu-miR-223-3p	66818	Smim7	1	1	1	1	1	5
mmu-miR-223-3p	66822	Fbxo25	1	1	1	1	1	5
mmu-miR-223-3p	66830	Nacc1	1	1	1	1	1	5
mmu-miR-223-3p	66854	Trim35	1	1	1	1	1	5
mmu-miR-223-3p	66867	Hmg20a	1	1	1	1	1	5
mmu-miR-223-3p	66885	Acadsb	1	1	1	1	1	5
mmu-miR-223-3p	66902	Mtap	1	1	1	1	1	5
mmu-miR-223-3p	66922	Rras2	1	1	1	1	1	5
mmu-miR-223-3p	66935	Cir1	1	1	1	1	1	5
mmu-miR-223-3p	66970	Ssbp2	1	1	1	1	1	5
mmu-miR-223-3p	66978	Luc7l	1	1	1	1	1	5
mmu-miR-223-3p	67006	Cisd2	1	1	1	1	1	5
mmu-miR-223-3p	67008	Yae1d1	1	1	1	1	1	5
mmu-miR-223-3p	67050	Nkap	1	1	1	1	1	5
mmu-miR-223-3p	67065	Polr3d	1	1	1	1	1	5
mmu-miR-223-3p	67071	Rps6ka6	1	1	1	1	1	5
mmu-miR-223-3p	67118	Bfar	1	1	1	1	1	5
mmu-miR-223-3p	67128	Ube2g1	1	1	1	1	1	5
mmu-miR-223-3p	67144	Lrrc40	1	1	1	1	1	5
mmu-miR-223-3p	67149	Nkain1	1	1	1	1	1	5
mmu-miR-223-3p	67151	Psmd9	1	1	1	1	1	5
mmu-miR-223-3p	67203	Nde1	1	1	1	1	1	5
mmu-miR-223-3p	67225	Rnpc3	1	1	1	1	1	5
mmu-miR-223-3p	67230	Zfp329	1	1	1	1	1	5
mmu-miR-223-3p	67269	Agtpbp1	1	1	1	1	1	5
mmu-miR-223-3p	67276	Eri1	1	1	1	1	1	5
mmu-miR-223-3p	67299	Dock7	1	1	1	1	1	5
mmu-miR-223-3p	67302	Zc3h13	1	1	1	1	1	5
mmu-miR-223-3p	67425	Eps8l1	1	1	1	1	1	5
mmu-miR-223-3p	67454	Ikbip	1	1	1	1	1	5
mmu-miR-223-3p	67469	Abhd5	1	1	1	1	1	5
mmu-miR-223-3p	67486	Polr3g	1	1	1	1	1	5
mmu-miR-223-3p	67516	Kctd4	1	1	1	1	1	5
mmu-miR-223-3p	67556	Pigm	1	1	1	1	1	5
mmu-miR-223-3p	67568	Mrfap1	1	1	1	1	1	5
mmu-miR-223-3p	67712	Slc25a37	1	1	1	1	1	5

mmu-miR-223-3p	67819	Derl1	1	1	1	1	1	5
mmu-miR-223-3p	67867	Lrrc28	1	1	1	1	1	5
mmu-miR-223-3p	67886	Camsap2	1	1	1	1	1	5
mmu-miR-223-3p	67890	Ufm1	1	1	1	1	1	5
mmu-miR-223-3p	67911	Zfp169	1	1	1	1	1	5
mmu-miR-223-3p	67952	Tomm20	1	1	1	1	1	5
mmu-miR-223-3p	67972	Atp2b1	1	1	1	1	1	5
mmu-miR-223-3p	68027	Tmem178	1	1	1	1	1	5
mmu-miR-223-3p	68051	Nutf2	1	1	1	1	1	5
mmu-miR-223-3p	68053	Ubxn2b	1	1	1	1	1	5
mmu-miR-223-3p	68117	Apool	1	1	1	1	1	5
mmu-miR-223-3p	68119	Cmtm3	1	1	1	1	1	5
mmu-miR-223-3p	68169	Ndnf	1	1	1	1	1	5
mmu-miR-223-3p	68203	Diras2	1	1	1	1	1	5
mmu-miR-223-3p	68277	2310057M21Rik	1	1	1	1	1	5
mmu-miR-223-3p	68303	Fam114a1	1	1	1	1	1	5
mmu-miR-223-3p	68304	Kdelc2	1	1	1	1	1	5
mmu-miR-223-3p	68420	Ankrd13a	1	1	1	1	1	5
mmu-miR-223-3p	68487	Tmem140	1	1	1	1	1	5
mmu-miR-223-3p	68493	Ndufaf4	1	1	1	1	1	5
mmu-miR-223-3p	68523	Fam96b	1	1	1	1	1	5
mmu-miR-223-3p	68553	Col6a4	1	1	1	1	1	5
mmu-miR-223-3p	68559	Pdrg1	1	1	1	1	1	5
mmu-miR-223-3p	68572	Ict1	1	1	1	1	1	5
mmu-miR-223-3p	68659	Fam198b	1	1	1	1	1	5
mmu-miR-223-3p	68703	Rere	1	1	1	1	1	5
mmu-miR-223-3p	68708	Rabl2	1	1	1	1	1	5
mmu-miR-223-3p	68728	Trp53inp2	1	1	1	1	1	5
mmu-miR-223-3p	68729	Trim37	1	1	1	1	1	5
mmu-miR-223-3p	68810	Nexn	1	1	1	1	1	5
mmu-miR-223-3p	68813	Dock5	1	1	1	1	1	5
mmu-miR-223-3p	68861	1190002N15Rik	1	1	1	1	1	5
mmu-miR-223-3p	68970	Dcaf12	1	1	1	1	1	5
mmu-miR-223-3p	69155	1810030O07Rik	1	1	1	1	1	5
mmu-miR-223-3p	69183	C1qtnf2	1	1	1	1	1	5
mmu-miR-223-3p	69226	Snx24	1	1	1	1	1	5
mmu-miR-223-3p	69227	Selt	1	1	1	1	1	5
mmu-miR-223-3p	69259	Kctd5	1	1	1	1	1	5
mmu-miR-223-3p	69274	Ctdspl	1	1	1	1	1	5
mmu-miR-223-3p	69444	Lyzl6	1	1	1	1	1	5
mmu-miR-223-3p	69540	Klk10	1	1	1	1	1	5
mmu-miR-223-3p	69562	Cdk13	1	1	1	1	1	5
mmu-miR-223-3p	69627	Fam89a	1	1	1	1	1	5
mmu-miR-223-3p	69707	Iqcg	1	1	1	1	1	5
mmu-miR-223-3p	69863	Ttc39b	1	1	1	1	1	5
mmu-miR-223-3p	69900	Mfsd11	1	1	1	1	1	5
mmu-miR-223-3p	69906	Slc25a32	1	1	1	1	1	5
mmu-miR-223-3p	69923	Agk	1	1	1	1	1	5
mmu-miR-223-3p	70024	Mcm10	1	1	1	1	1	5
mmu-miR-223-3p	70052	Prpf4	1	1	1	1	1	5
mmu-miR-223-3p	70061	Sdr9c7	1	1	1	1	1	5
mmu-miR-223-3p	70078	Nol7	1	1	1	1	1	5
mmu-miR-223-3p	70155	Ogfrl1	1	1	1	1	1	5
mmu-miR-223-3p	70291	2510049J12Rik	1	1	1	1	1	5
mmu-miR-223-3p	70316	Ndufab1	1	1	1	1	1	5

mmu-miR-223-3p	70354	Secisbp2l	1	1	1	1	1	5
mmu-miR-223-3p	70361	Lman1	1	1	1	1	1	5
mmu-miR-223-3p	70373	Gpatch2l	1	1	1	1	1	5
mmu-miR-223-3p	70380	Mospd1	1	1	1	1	1	5
mmu-miR-223-3p	70387	Ttc9c	1	1	1	1	1	5
mmu-miR-223-3p	70417	Megf10	1	1	1	1	1	5
mmu-miR-223-3p	70420	2610034B18Rik	1	1	1	1	1	5
mmu-miR-223-3p	70432	Rufy2	1	1	1	1	1	5
mmu-miR-223-3p	70433	Draxin	1	1	1	1	1	5
mmu-miR-223-3p	70560	Wars2	1	1	1	1	1	5
mmu-miR-223-3p	70646	Naa30	1	1	1	1	1	5
mmu-miR-223-3p	70650	Zcchc8	1	1	1	1	1	5
mmu-miR-223-3p	70974	Pgm2l1	1	1	1	1	1	5
mmu-miR-223-3p	71227	Daw1	1	1	1	1	1	5
mmu-miR-223-3p	71302	Arhgap26	1	1	1	1	1	5
mmu-miR-223-3p	71361	Aifm2	1	1	1	1	1	5
mmu-miR-223-3p	71409	Fmnl2	1	1	1	1	1	5
mmu-miR-223-3p	71449	Mettl13	1	1	1	1	1	5
mmu-miR-223-3p	71584	Gdpd2	1	1	1	1	1	5
mmu-miR-223-3p	71773	Ugt2b1	1	1	1	1	1	5
mmu-miR-223-3p	71776	Tha1	1	1	1	1	1	5
mmu-miR-223-3p	71779	March8	1	1	1	1	1	5
mmu-miR-223-3p	71833	Dcaf7	1	1	1	1	1	5
mmu-miR-223-3p	71876	Mlf1ip	1	1	1	1	1	5
mmu-miR-223-3p	71878	Fam83d	1	1	1	1	1	5
mmu-miR-223-3p	71900	Tmem106b	1	1	1	1	1	5
mmu-miR-223-3p	71918	Zcchc24	1	1	1	1	1	5
mmu-miR-223-3p	71929	Tmem123	1	1	1	1	1	5
mmu-miR-223-3p	72055	Slc38a10	1	1	1	1	1	5
mmu-miR-223-3p	72102	Dusp11	1	1	1	1	1	5
mmu-miR-223-3p	72117	Naa50	1	1	1	1	1	5
mmu-miR-223-3p	72129	Pex13	1	1	1	1	1	5
mmu-miR-223-3p	72144	Slc37a3	1	1	1	1	1	5
mmu-miR-223-3p	72160	Tmem163	1	1	1	1	1	5
mmu-miR-223-3p	72168	Aifm3	1	1	1	1	1	5
mmu-miR-223-3p	72175	Mfsd8	1	1	1	1	1	5
mmu-miR-223-3p	72194	Fbxl20	1	1	1	1	1	5
mmu-miR-223-3p	72267	Lrrc8e	1	1	1	1	1	5
mmu-miR-223-3p	72357	2210016L21Rik	1	1	1	1	1	5
mmu-miR-223-3p	72413	Kcnmb2	1	1	1	1	1	5
mmu-miR-223-3p	72477	Tmem87b	1	1	1	1	1	5
mmu-miR-223-3p	72508	Rps6kb1	1	1	1	1	1	5
mmu-miR-223-3p	72543	Mvb12b	1	1	1	1	1	5
mmu-miR-223-3p	72747	Ttc39c	1	1	1	1	1	5
mmu-miR-223-3p	72759	Tmem135	1	1	1	1	1	5
mmu-miR-223-3p	72795	Ttc19	1	1	1	1	1	5
mmu-miR-223-3p	72852	Mblac2	1	1	1	1	1	5
mmu-miR-223-3p	72948	Tppp	1	1	1	1	1	5
mmu-miR-223-3p	72999	Insig2	1	1	1	1	1	5
mmu-miR-223-3p	73010	Gpr22	1	1	1	1	1	5
mmu-miR-223-3p	73068	Fut11	1	1	1	1	1	5
mmu-miR-223-3p	73130	Tmed5	1	1	1	1	1	5
mmu-miR-223-3p	73137	Prrc1	1	1	1	1	1	5
mmu-miR-223-3p	73178	Wasl	1	1	1	1	1	5
mmu-miR-223-3p	73192	Xpot	1	1	1	1	1	5

mmu-miR-223-3p	73379	Dcbld2	1	1	1	1	1	5
mmu-miR-223-3p	73430	1700049G17Rik	1	1	1	1	1	5
mmu-miR-223-3p	73447	Wdr13	1	1	1	1	1	5
mmu-miR-223-3p	73608	Marveld3	1	1	1	1	1	5
mmu-miR-223-3p	73649	Cybrd1	1	1	1	1	1	5
mmu-miR-223-3p	73703	Dppa2	1	1	1	1	1	5
mmu-miR-223-3p	73902	Psmb11	1	1	1	1	1	5
mmu-miR-223-3p	73914	Irak3	1	1	1	1	1	5
mmu-miR-223-3p	73916	Ift57	1	1	1	1	1	5
mmu-miR-223-3p	73940	Hapln2	1	1	1	1	1	5
mmu-miR-223-3p	74020	Cpne4	1	1	1	1	1	5
mmu-miR-223-3p	74055	Plce1	1	1	1	1	1	5
mmu-miR-223-3p	74102	Slc35a5	1	1	1	1	1	5
mmu-miR-223-3p	74108	Parn	1	1	1	1	1	5
mmu-miR-223-3p	74131	Sash3	1	1	1	1	1	5
mmu-miR-223-3p	74165	Fbxl22	1	1	1	1	1	5
mmu-miR-223-3p	74167	Nudt9	1	1	1	1	1	5
mmu-miR-223-3p	74192	Arpc5l	1	1	1	1	1	5
mmu-miR-223-3p	74201	Cep97	1	1	1	1	1	5
mmu-miR-223-3p	74229	Paqr8	1	1	1	1	1	5
mmu-miR-223-3p	74252	Armc1	1	1	1	1	1	5
mmu-miR-223-3p	74365	Lonrf3	1	1	1	1	1	5
mmu-miR-223-3p	74370	Rptor	1	1	1	1	1	5
mmu-miR-223-3p	74427	Eaf1	1	1	1	1	1	5
mmu-miR-223-3p	74438	Clvs1	1	1	1	1	1	5
mmu-miR-223-3p	74442	Sgms2	1	1	1	1	1	5
mmu-miR-223-3p	74492	Kbtbd13	1	1	1	1	1	5
mmu-miR-223-3p	74552	Nipal3	1	1	1	1	1	5
mmu-miR-223-3p	74747	Ddit4	1	1	1	1	1	5
mmu-miR-223-3p	75029	Purg	1	1	1	1	1	5
mmu-miR-223-3p	75064	Zcchc13	1	1	1	1	1	5
mmu-miR-223-3p	75104	Mmd2	1	1	1	1	1	5
mmu-miR-223-3p	75219	Dusp18	1	1	1	1	1	5
mmu-miR-223-3p	75420	Secisbp2	1	1	1	1	1	5
mmu-miR-223-3p	75423	Arl5a	1	1	1	1	1	5
mmu-miR-223-3p	75540	Fpgt	1	1	1	1	1	5
mmu-miR-223-3p	75612	Gns	1	1	1	1	1	5
mmu-miR-223-3p	75616	Smim15	1	1	1	1	1	5
mmu-miR-223-3p	75725	Phf14	1	1	1	1	1	5
mmu-miR-223-3p	75785	Klhl24	1	1	1	1	1	5
mmu-miR-223-3p	75909	Vmp1	1	1	1	1	1	5
mmu-miR-223-3p	75953	Samd7	1	1	1	1	1	5
mmu-miR-223-3p	76044	Ncapg2	1	1	1	1	1	5
mmu-miR-223-3p	76108	Rap2a	1	1	1	1	1	5
mmu-miR-223-3p	76123	Gpsm2	1	1	1	1	1	5
mmu-miR-223-3p	76267	Fads1	1	1	1	1	1	5
mmu-miR-223-3p	76281	Tax1bp3	1	1	1	1	1	5
mmu-miR-223-3p	76338	Rab2b	1	1	1	1	1	5
mmu-miR-223-3p	76367	Trp53rk	1	1	1	1	1	5
mmu-miR-223-3p	76453	Prss23	1	1	1	1	1	5
mmu-miR-223-3p	76459	Car12	1	1	1	1	1	5
mmu-miR-223-3p	76568	Ift46	1	1	1	1	1	5
mmu-miR-223-3p	76582	Ipo11	1	1	1	1	1	5
mmu-miR-223-3p	76626	Msi2	1	1	1	1	1	5
mmu-miR-223-3p	76687	Spcs3	1	1	1	1	1	5

mmu-miR-223-3p	76793	Snip1	1	1	1	1	1	5
mmu-miR-223-3p	76800	Usp42	1	1	1	1	1	5
mmu-miR-223-3p	77037	Mrap	1	1	1	1	1	5
mmu-miR-223-3p	77305	Wdr82	1	1	1	1	1	5
mmu-miR-223-3p	77407	Rab35	1	1	1	1	1	5
mmu-miR-223-3p	77446	Heg1	1	1	1	1	1	5
mmu-miR-223-3p	77480	Kidins220	1	1	1	1	1	5
mmu-miR-223-3p	77579	Myh10	1	1	1	1	1	5
mmu-miR-223-3p	77739	Adamtsl1	1	1	1	1	1	5
mmu-miR-223-3p	77767	Ermn	1	1	1	1	1	5
mmu-miR-223-3p	77781	Epm2aip1	1	1	1	1	1	5
mmu-miR-223-3p	77794	Adamtsl2	1	1	1	1	1	5
mmu-miR-223-3p	77799	Sla2	1	1	1	1	1	5
mmu-miR-223-3p	77963	Hook1	1	1	1	1	1	5
mmu-miR-223-3p	77974	Rdh12	1	1	1	1	1	5
mmu-miR-223-3p	78232	Trappc6b	1	1	1	1	1	5
mmu-miR-223-3p	78388	Mvp	1	1	1	1	1	5
mmu-miR-223-3p	78521	B230219D22Rik	1	1	1	1	1	5
mmu-miR-223-3p	78656	Brd8	1	1	1	1	1	5
mmu-miR-223-3p	78757	Rictor	1	1	1	1	1	5
mmu-miR-223-3p	78787	Usp54	1	1	1	1	1	5
mmu-miR-223-3p	78795	Armc9	1	1	1	1	1	5
mmu-miR-223-3p	78797	Ndor1	1	1	1	1	1	5
mmu-miR-223-3p	78803	Fbxo43	1	1	1	1	1	5
mmu-miR-223-3p	78943	Ern1	1	1	1	1	1	5
mmu-miR-223-3p	79235	Lrat	1	1	1	1	1	5
mmu-miR-223-3p	79554	Gltpd1	1	1	1	1	1	5
mmu-miR-223-3p	80289	Lysmd3	1	1	1	1	1	5
mmu-miR-223-3p	80292	Zxdc	1	1	1	1	1	5
mmu-miR-223-3p	80720	Pbx4	1	1	1	1	1	5
mmu-miR-223-3p	80782	Klrb1b	1	1	1	1	1	5
mmu-miR-223-3p	80883	Ntng1	1	1	1	1	1	5
mmu-miR-223-3p	80892	Zfhx4	1	1	1	1	1	5
mmu-miR-223-3p	80911	Acox3	1	1	1	1	1	5
mmu-miR-223-3p	80985	Trim44	1	1	1	1	1	5
mmu-miR-223-3p	81013	Vmn1r65	1	1	1	1	1	5
mmu-miR-223-3p	81879	Tfcp2l1	1	1	1	1	1	5
mmu-miR-223-3p	81910	Rrbp1	1	1	1	1	1	5
mmu-miR-223-3p	83398	Ndst3	1	1	1	1	1	5
mmu-miR-223-3p	83430	Il23a	1	1	1	1	1	5
mmu-miR-223-3p	83435	Plekha3	1	1	1	1	1	5
mmu-miR-223-3p	83691	Crispld1	1	1	1	1	1	5
mmu-miR-223-3p	83797	Smarcd1	1	1	1	1	1	5
mmu-miR-223-3p	83922	Cep41	1	1	1	1	1	5
mmu-miR-223-3p	83925	Trps1	1	1	1	1	1	5
mmu-miR-223-3p	83946	Phip	1	1	1	1	1	5
mmu-miR-223-3p	83962	Btbd1	1	1	1	1	1	5
mmu-miR-223-3p	83995	Mmp1a	1	1	1	1	1	5
mmu-miR-223-3p	84035	Kremen1	1	1	1	1	1	5
mmu-miR-223-3p	84095	Pi4k2a	1	1	1	1	1	5
mmu-miR-223-3p	84652	Fam126a	1	1	1	1	1	5
mmu-miR-223-3p	93673	Cml2	1	1	1	1	1	5
mmu-miR-223-3p	93681	Zkscan8	1	1	1	1	1	5
mmu-miR-223-3p	93688	Klhl1	1	1	1	1	1	5
mmu-miR-223-3p	93728	Pabpc5	1	1	1	1	1	5

mmu-miR-223-3p	93736	Aff4	1	1	1	1	1	5
mmu-miR-223-3p	93760	Arid1a	1	1	1	1	1	5
mmu-miR-223-3p	93890	Pcdhb19	1	1	1	1	1	5
mmu-miR-223-3p	94109	Csmd1	1	1	1	1	1	5
mmu-miR-223-3p	94180	Acsbg1	1	1	1	1	1	5
mmu-miR-223-3p	94216	Col4a6	1	1	1	1	1	5
mmu-miR-223-3p	94223	Dgcr8	1	1	1	1	1	5
mmu-miR-223-3p	94253	Hecw1	1	1	1	1	1	5
mmu-miR-223-3p	97064	Wwtr1	1	1	1	1	1	5
mmu-miR-223-3p	97487	Cmtm4	1	1	1	1	1	5
mmu-miR-223-3p	97820	4833439L19Rik	1	1	1	1	1	5
mmu-miR-223-3p	98386	Lbr	1	1	1	1	1	5
mmu-miR-223-3p	98417	Cnih4	1	1	1	1	1	5
mmu-miR-223-3p	98711	Rdh10	1	1	1	1	1	5
mmu-miR-223-3p	99003	Qser1	1	1	1	1	1	5
mmu-miR-223-3p	99371	Arfgef2	1	1	1	1	1	5
mmu-miR-223-3p	99512	Wdr47	1	1	1	1	1	5
mmu-miR-223-3p	99887	Tmem56	1	1	1	1	1	5
mmu-miR-223-3p	99929	Tiparp	1	1	1	1	1	5
mmu-miR-223-3p	100061	Lrrc19	1	1	1	1	1	5
mmu-miR-223-3p	100072	Camta1	1	1	1	1	1	5
mmu-miR-223-3p	100129	Gpr153	1	1	1	1	1	5
mmu-miR-223-3p	100198	H6pd	1	1	1	1	1	5
mmu-miR-223-3p	100201	Tmem64	1	1	1	1	1	5
mmu-miR-223-3p	100561	Slc15a4	1	1	1	1	1	5
mmu-miR-223-3p	100710	Pds5b	1	1	1	1	1	5
mmu-miR-223-3p	101118	Tmem168	1	1	1	1	1	5
mmu-miR-223-3p	101490	Inpp5f	1	1	1	1	1	5
mmu-miR-223-3p	101685	Spty2d1	1	1	1	1	1	5
mmu-miR-223-3p	101835	AW146154	1	1	1	1	1	5
mmu-miR-223-3p	102462	Imp3	1	1	1	1	1	5
mmu-miR-223-3p	102580	Alg9	1	1	1	1	1	5
mmu-miR-223-3p	102774	Bbs4	1	1	1	1	1	5
mmu-miR-223-3p	102791	Tcta	1	1	1	1	1	5
mmu-miR-223-3p	103724	Tbc1d10a	1	1	1	1	1	5
mmu-miR-223-3p	104806	Fancm	1	1	1	1	1	5
mmu-miR-223-3p	104885	Tmem179	1	1	1	1	1	5
mmu-miR-223-3p	105171	Arrdc3	1	1	1	1	1	5
mmu-miR-223-3p	105355	Slc17a3	1	1	1	1	1	5
mmu-miR-223-3p	105859	Csdc2	1	1	1	1	1	5
mmu-miR-223-3p	106369	Ypel1	1	1	1	1	1	5
mmu-miR-223-3p	107321	Lpxn	1	1	1	1	1	5
mmu-miR-223-3p	107358	Tm9sf3	1	1	1	1	1	5
mmu-miR-223-3p	107513	Ssr1	1	1	1	1	1	5
mmu-miR-223-3p	107528	Magee1	1	1	1	1	1	5
mmu-miR-223-3p	107585	Dio3	1	1	1	1	1	5
mmu-miR-223-3p	107605	Rdh1	1	1	1	1	1	5
mmu-miR-223-3p	107650	Pi4kb	1	1	1	1	1	5
mmu-miR-223-3p	108123	Napg	1	1	1	1	1	5
mmu-miR-223-3p	108150	Galnt7	1	1	1	1	1	5
mmu-miR-223-3p	108735	Sft2d2	1	1	1	1	1	5
mmu-miR-223-3p	108943	Trmt10a	1	1	1	1	1	5
mmu-miR-223-3p	109108	Slc30a9	1	1	1	1	1	5
mmu-miR-223-3p	109151	Chd9	1	1	1	1	1	5
mmu-miR-223-3p	109246	Tspan9	1	1	1	1	1	5

mmu-miR-223-3p	109333	Pkn2	1	1	1	1	1	5
mmu-miR-223-3p	109672	Cyb5	1	1	1	1	1	5
mmu-miR-223-3p	110052	Dek	1	1	1	1	1	5
mmu-miR-223-3p	110074	Dut	1	1	1	1	1	5
mmu-miR-223-3p	110460	Acat2	1	1	1	1	1	5
mmu-miR-223-3p	110524	Dgkq	1	1	1	1	1	5
mmu-miR-223-3p	110639	Prps2	1	1	1	1	1	5
mmu-miR-223-3p	110696	H2-M10.3	1	1	1	1	1	5
mmu-miR-223-3p	110876	Scn2a1	1	1	1	1	1	5
mmu-miR-223-3p	110886	Gabra5	1	1	1	1	1	5
mmu-miR-223-3p	110948	Hlcs	1	1	1	1	1	5
mmu-miR-223-3p	114615	Elac1	1	1	1	1	1	5
mmu-miR-223-3p	116847	Prelp	1	1	1	1	1	5
mmu-miR-223-3p	116873	Stim2	1	1	1	1	1	5
mmu-miR-223-3p	118446	Gjc3	1	1	1	1	1	5
mmu-miR-223-3p	140486	Igf2bp1	1	1	1	1	1	5
mmu-miR-223-3p	140488	Igf2bp3	1	1	1	1	1	5
mmu-miR-223-3p	140780	Bmp2k	1	1	1	1	1	5
mmu-miR-223-3p	140887	Lnx2	1	1	1	1	1	5
mmu-miR-223-3p	142682	Zcchc14	1	1	1	1	1	5
mmu-miR-223-3p	170439	Elovl6	1	1	1	1	1	5
mmu-miR-223-3p	170625	Snx18	1	1	1	1	1	5
mmu-miR-223-3p	170638	Hpcal4	1	1	1	1	1	5
mmu-miR-223-3p	170656	Krtap21-1	1	1	1	1	1	5
mmu-miR-223-3p	170720	Card14	1	1	1	1	1	5
mmu-miR-223-3p	170736	Parvb	1	1	1	1	1	5
mmu-miR-223-3p	170745	Xpnpep2	1	1	1	1	1	5
mmu-miR-223-3p	170753	Zfp704	1	1	1	1	1	5
mmu-miR-223-3p	170757	Eltd1	1	1	1	1	1	5
mmu-miR-223-3p	170772	Glcci1	1	1	1	1	1	5
mmu-miR-223-3p	170790	Mlc1	1	1	1	1	1	5
mmu-miR-223-3p	171212	Galnt10	1	1	1	1	1	5
mmu-miR-223-3p	171543	Bmf	1	1	1	1	1	5
mmu-miR-223-3p	192198	Lrrc4	1	1	1	1	1	5
mmu-miR-223-3p	192986	Cyb5d2	1	1	1	1	1	5
mmu-miR-223-3p	193796	Kdm4b	1	1	1	1	1	5
mmu-miR-223-3p	194590	Reps2	1	1	1	1	1	5
mmu-miR-223-3p	194952	Jmjd4	1	1	1	1	1	5
mmu-miR-223-3p	195208	Dcdc2a	1	1	1	1	1	5
mmu-miR-223-3p	207259	Zbtb7c	1	1	1	1	1	5
mmu-miR-223-3p	207375	Fam120c	1	1	1	1	1	5
mmu-miR-223-3p	207474	Kctd12b	1	1	1	1	1	5
mmu-miR-223-3p	207592	Tbc1d16	1	1	1	1	1	5
mmu-miR-223-3p	207667	Skor1	1	1	1	1	1	5
mmu-miR-223-3p	207781	C2cd2	1	1	1	1	1	5
mmu-miR-223-3p	207785	Csrnp2	1	1	1	1	1	5
mmu-miR-223-3p	207819	4930539E08Rik	1	1	1	1	1	5
mmu-miR-223-3p	208188	Ghsr	1	1	1	1	1	5
mmu-miR-223-3p	208440	Dip2c	1	1	1	1	1	5
mmu-miR-223-3p	208869	Dock3	1	1	1	1	1	5
mmu-miR-223-3p	208922	Cpeb3	1	1	1	1	1	5
mmu-miR-223-3p	208943	Myo5c	1	1	1	1	1	5
mmu-miR-223-3p	209224	Enox2	1	1	1	1	1	5
mmu-miR-223-3p	209294	Csta	1	1	1	1	1	5
mmu-miR-223-3p	209645	Bend7	1	1	1	1	1	5

mmu-miR-223-3p	210146	Irgq	1	1	1	1	1	5
mmu-miR-223-3p	210711	Mcmbp	1	1	1	1	1	5
mmu-miR-223-3p	210853	Zfp947	1	1	1	1	1	5
mmu-miR-223-3p	210933	Bai3	1	1	1	1	1	5
mmu-miR-223-3p	211232	Cpne9	1	1	1	1	1	5
mmu-miR-223-3p	212070	Clrn3	1	1	1	1	1	5
mmu-miR-223-3p	212307	Mapre2	1	1	1	1	1	5
mmu-miR-223-3p	212439	AA986860	1	1	1	1	1	5
mmu-miR-223-3p	212531	Sh3bgrl2	1	1	1	1	1	5
mmu-miR-223-3p	213391	Rassf4	1	1	1	1	1	5
mmu-miR-223-3p	213582	Map9	1	1	1	1	1	5
mmu-miR-223-3p	213827	Arcn1	1	1	1	1	1	5
mmu-miR-223-3p	214505	Gnptg	1	1	1	1	1	5
mmu-miR-223-3p	214763	Mb21d1	1	1	1	1	1	5
mmu-miR-223-3p	214897	Csnk1g1	1	1	1	1	1	5
mmu-miR-223-3p	215085	Slc35f1	1	1	1	1	1	5
mmu-miR-223-3p	215193	Diexf	1	1	1	1	1	5
mmu-miR-223-3p	215378	Brinp3	1	1	1	1	1	5
mmu-miR-223-3p	215446	Entpd3	1	1	1	1	1	5
mmu-miR-223-3p	215627	Zbtb8b	1	1	1	1	1	5
mmu-miR-223-3p	215654	Cdh12	1	1	1	1	1	5
mmu-miR-223-3p	215798	Gpr126	1	1	1	1	1	5
mmu-miR-223-3p	216119	Ybey	1	1	1	1	1	5
mmu-miR-223-3p	216134	Pdxk	1	1	1	1	1	5
mmu-miR-223-3p	216156	Wdr18	1	1	1	1	1	5
mmu-miR-223-3p	216188	Aldh1l2	1	1	1	1	1	5
mmu-miR-223-3p	216238	Eea1	1	1	1	1	1	5
mmu-miR-223-3p	216543	Cep68	1	1	1	1	1	5
mmu-miR-223-3p	216618	Ccdc104	1	1	1	1	1	5
mmu-miR-223-3p	216799	Nlrp3	1	1	1	1	1	5
mmu-miR-223-3p	216856	Nlgn2	1	1	1	1	1	5
mmu-miR-223-3p	216877	Dhx33	1	1	1	1	1	5
mmu-miR-223-3p	217082	Hlf	1	1	1	1	1	5
mmu-miR-223-3p	217116	Spata20	1	1	1	1	1	5
mmu-miR-223-3p	217480	Dgkb	1	1	1	1	1	5
mmu-miR-223-3p	217588	Mbip	1	1	1	1	1	5
mmu-miR-223-3p	217734	Pomt2	1	1	1	1	1	5
mmu-miR-223-3p	217893	Pacs2	1	1	1	1	1	5
mmu-miR-223-3p	218035	Vps41	1	1	1	1	1	5
mmu-miR-223-3p	218397	Rasa1	1	1	1	1	1	5
mmu-miR-223-3p	218440	Ankrd34b	1	1	1	1	1	5
mmu-miR-223-3p	218543	Srek1	1	1	1	1	1	5
mmu-miR-223-3p	218952	Fermt2	1	1	1	1	1	5
mmu-miR-223-3p	218989	Tmem260	1	1	1	1	1	5
mmu-miR-223-3p	223332	Ranbp3l	1	1	1	1	1	5
mmu-miR-223-3p	223473	Nipal2	1	1	1	1	1	5
mmu-miR-223-3p	223513	Abra	1	1	1	1	1	5
mmu-miR-223-3p	223690	Ankrd54	1	1	1	1	1	5
mmu-miR-223-3p	223696	Tomm22	1	1	1	1	1	5
mmu-miR-223-3p	223722	Mcat	1	1	1	1	1	5
mmu-miR-223-3p	223732	Ldoc1l	1	1	1	1	1	5
mmu-miR-223-3p	223753	Cerk	1	1	1	1	1	5
mmu-miR-223-3p	224022	Slc7a4	1	1	1	1	1	5
mmu-miR-223-3p	224132	Dirc2	1	1	1	1	1	5
mmu-miR-223-3p	224273	Crybg3	1	1	1	1	1	5

mmu-miR-223-3p	224640	Lemd2	1	1	1	1	1	5
mmu-miR-223-3p	224742	Abcf1	1	1	1	1	1	5
mmu-miR-223-3p	224761	H2-M10.5	1	1	1	1	1	5
mmu-miR-223-3p	224796	Clic5	1	1	1	1	1	5
mmu-miR-223-3p	225027	Srsf7	1	1	1	1	1	5
mmu-miR-223-3p	225266	Klhl14	1	1	1	1	1	5
mmu-miR-223-3p	225467	Pggt1b	1	1	1	1	1	5
mmu-miR-223-3p	225471	Ticam2	1	1	1	1	1	5
mmu-miR-223-3p	225523	Cep120	1	1	1	1	1	5
mmu-miR-223-3p	225631	Onecut2	1	1	1	1	1	5
mmu-miR-223-3p	225644	Cplx4	1	1	1	1	1	5
mmu-miR-223-3p	226143	Cyp2c44	1	1	1	1	1	5
mmu-miR-223-3p	226151	Fam178a	1	1	1	1	1	5
mmu-miR-223-3p	226418	Yod1	1	1	1	1	1	5
mmu-miR-223-3p	226421	5430435G22Rik	1	1	1	1	1	5
mmu-miR-223-3p	226432	Ipo9	1	1	1	1	1	5
mmu-miR-223-3p	226470	Zbtb41	1	1	1	1	1	5
mmu-miR-223-3p	226744	Cnst	1	1	1	1	1	5
mmu-miR-223-3p	226757	Wdr26	1	1	1	1	1	5
mmu-miR-223-3p	226777	C130074G19Rik	1	1	1	1	1	5
mmu-miR-223-3p	226841	Vash2	1	1	1	1	1	5
mmu-miR-223-3p	226982	Eif5b	1	1	1	1	1	5
mmu-miR-223-3p	227327	B3gnt7	1	1	1	1	1	5
mmu-miR-223-3p	227399	Ppip5k2	1	1	1	1	1	5
mmu-miR-223-3p	227541	Camk1d	1	1	1	1	1	5
mmu-miR-223-3p	228769	Psmf1	1	1	1	1	1	5
mmu-miR-223-3p	228850	Ralgapb	1	1	1	1	1	5
mmu-miR-223-3p	229214	Qrfpr	1	1	1	1	1	5
mmu-miR-223-3p	229357	Gpr149	1	1	1	1	1	5
mmu-miR-223-3p	229584	Pogz	1	1	1	1	1	5
mmu-miR-223-3p	229782	Slc35a3	1	1	1	1	1	5
mmu-miR-223-3p	229841	Cenpe	1	1	1	1	1	5
mmu-miR-223-3p	229933	Clca5	1	1	1	1	1	5
mmu-miR-223-3p	230376	Haus6	1	1	1	1	1	5
mmu-miR-223-3p	230648	Efcab14	1	1	1	1	1	5
mmu-miR-223-3p	230700	Foxj3	1	1	1	1	1	5
mmu-miR-223-3p	230837	Asap3	1	1	1	1	1	5
mmu-miR-223-3p	230848	Zbtb40	1	1	1	1	1	5
mmu-miR-223-3p	230857	Ece1	1	1	1	1	1	5
mmu-miR-223-3p	231051	Kmt2c	1	1	1	1	1	5
mmu-miR-223-3p	231070	Insig1	1	1	1	1	1	5
mmu-miR-223-3p	231503	Tmem150c	1	1	1	1	1	5
mmu-miR-223-3p	231510	Agpat9	1	1	1	1	1	5
mmu-miR-223-3p	232023	Vopp1	1	1	1	1	1	5
mmu-miR-223-3p	232146	Eva1a	1	1	1	1	1	5
mmu-miR-223-3p	232409	Clec2e	1	1	1	1	1	5
mmu-miR-223-3p	232449	Dera	1	1	1	1	1	5
mmu-miR-223-3p	232539	Klhl42	1	1	1	1	1	5
mmu-miR-223-3p	232906	Grlf1	1	1	1	1	1	5
mmu-miR-223-3p	233011	Itpkc	1	1	1	1	1	5
mmu-miR-223-3p	233033	Samd4b	1	1	1	1	1	5
mmu-miR-223-3p	233115	Dpy19l3	1	1	1	1	1	5
mmu-miR-223-3p	233315	Mtmr10	1	1	1	1	1	5
mmu-miR-223-3p	233490	Crebzf	1	1	1	1	1	5
mmu-miR-223-3p	233724	Tmem41b	1	1	1	1	1	5

mmu-miR-223-3p	233789	Smg1	1	1	1	1	1	5
mmu-miR-223-3p	233875	Ino80e	1	1	1	1	1	5
mmu-miR-223-3p	234404	Nxnl1	1	1	1	1	1	5
mmu-miR-223-3p	234878	BC021891	1	1	1	1	1	5
mmu-miR-223-3p	235050	Zfp810	1	1	1	1	1	5
mmu-miR-223-3p	235330	Ttc12	1	1	1	1	1	5
mmu-miR-223-3p	235344	Sik2	1	1	1	1	1	5
mmu-miR-223-3p	235379	Gldn	1	1	1	1	1	5
mmu-miR-223-3p	235442	Rab8b	1	1	1	1	1	5
mmu-miR-223-3p	235461	Fam63b	1	1	1	1	1	5
mmu-miR-223-3p	235611	Plxnb1	1	1	1	1	1	5
mmu-miR-223-3p	236312	Pyhin1	1	1	1	1	1	5
mmu-miR-223-3p	236781	Gpr119	1	1	1	1	1	5
mmu-miR-223-3p	236792	Mmgt1	1	1	1	1	1	5
mmu-miR-223-3p	237082	Nxt2	1	1	1	1	1	5
mmu-miR-223-3p	237221	Gemin8	1	1	1	1	1	5
mmu-miR-223-3p	237336	Tbpl1	1	1	1	1	1	5
mmu-miR-223-3p	237711	Eml6	1	1	1	1	1	5
mmu-miR-223-3p	237782	Smcr8	1	1	1	1	1	5
mmu-miR-223-3p	238692	Zfp874a	1	1	1	1	1	5
mmu-miR-223-3p	239157	Pnma2	1	1	1	1	1	5
mmu-miR-223-3p	239283	Oxgr1	1	1	1	1	1	5
mmu-miR-223-3p	239420	Csmd3	1	1	1	1	1	5
mmu-miR-223-3p	239447	Colec10	1	1	1	1	1	5
mmu-miR-223-3p	239510	Phf20l1	1	1	1	1	1	5
mmu-miR-223-3p	239739	Lamp3	1	1	1	1	1	5
mmu-miR-223-3p	239796	Mb21d2	1	1	1	1	1	5
mmu-miR-223-3p	239833	Lmln	1	1	1	1	1	5
mmu-miR-223-3p	239857	Cadm2	1	1	1	1	1	5
mmu-miR-223-3p	240665	Ccnj	1	1	1	1	1	5
mmu-miR-223-3p	240880	Scyl3	1	1	1	1	1	5
mmu-miR-223-3p	241070	Gpr1	1	1	1	1	1	5
mmu-miR-223-3p	241075	Plekhm3	1	1	1	1	1	5
mmu-miR-223-3p	241159	Neu4	1	1	1	1	1	5
mmu-miR-223-3p	241263	Gpr158	1	1	1	1	1	5
mmu-miR-223-3p	241447	Cers6	1	1	1	1	1	5
mmu-miR-223-3p	241589	D430041D05Rik	1	1	1	1	1	5
mmu-miR-223-3p	241656	Pak7	1	1	1	1	1	5
mmu-miR-223-3p	241944	D3Ertd254e	1	1	1	1	1	5
mmu-miR-223-3p	242362	Manea	1	1	1	1	1	5
mmu-miR-223-3p	242418	Dcaf10	1	1	1	1	1	5
mmu-miR-223-3p	242505	Rasef	1	1	1	1	1	5
mmu-miR-223-3p	242553	Kank4	1	1	1	1	1	5
mmu-miR-223-3p	242608	Podn	1	1	1	1	1	5
mmu-miR-223-3p	242642	Hpdl	1	1	1	1	1	5
mmu-miR-223-3p	242687	Wasf2	1	1	1	1	1	5
mmu-miR-223-3p	242860	Rsbn1l	1	1	1	1	1	5
mmu-miR-223-3p	242864	Napepld	1	1	1	1	1	5
mmu-miR-223-3p	243084	Tmprss11e	1	1	1	1	1	5
mmu-miR-223-3p	243376	Doxl2	1	1	1	1	1	5
mmu-miR-223-3p	243499	Lrrtm4	1	1	1	1	1	5
mmu-miR-223-3p	243725	Ppp1r9a	1	1	1	1	1	5
mmu-miR-223-3p	243755	Slc13a4	1	1	1	1	1	5
mmu-miR-223-3p	243881	Cyp2b23	1	1	1	1	1	5
mmu-miR-223-3p	243958	Siglecg	1	1	1	1	1	5

mmu-miR-223-3p	244180	E030002O03Rik	1	1	1	1	1	5
mmu-miR-223-3p	244349	Kat6a	1	1	1	1	1	5
mmu-miR-223-3p	244548	Elmod2	1	1	1	1	1	5
mmu-miR-223-3p	244672	Cwf19l2	1	1	1	1	1	5
mmu-miR-223-3p	244891	Scaper	1	1	1	1	1	5
mmu-miR-223-3p	245128	AU018091	1	1	1	1	1	5
mmu-miR-223-3p	245526	Pgr15l	1	1	1	1	1	5
mmu-miR-223-3p	245622	Fam199x	1	1	1	1	1	5
mmu-miR-223-3p	245695	Tceanc	1	1	1	1	1	5
mmu-miR-223-3p	245857	Ssh3	1	1	1	1	1	5
mmu-miR-223-3p	246229	Bivm	1	1	1	1	1	5
mmu-miR-223-3p	246257	Ovca2	1	1	1	1	1	5
mmu-miR-223-3p	252903	Ap1s3	1	1	1	1	1	5
mmu-miR-223-3p	258571	Olfr1033	1	1	1	1	1	5
mmu-miR-223-3p	259051	Olfr658	1	1	1	1	1	5
mmu-miR-223-3p	259104	Olfr613	1	1	1	1	1	5
mmu-miR-223-3p	259302	Srgap3	1	1	1	1	1	5
mmu-miR-223-3p	260315	Nav3	1	1	1	1	1	5
mmu-miR-223-3p	268354	Fam19a2	1	1	1	1	1	5
mmu-miR-223-3p	268379	Abca13	1	1	1	1	1	5
mmu-miR-223-3p	268390	Ahsa2	1	1	1	1	1	5
mmu-miR-223-3p	268396	Sh3pxd2b	1	1	1	1	1	5
mmu-miR-223-3p	268739	Arhgef40	1	1	1	1	1	5
mmu-miR-223-3p	268860	Abat	1	1	1	1	1	5
mmu-miR-223-3p	268902	Robo2	1	1	1	1	1	5
mmu-miR-223-3p	268936	Brpf3	1	1	1	1	1	5
mmu-miR-223-3p	269224	Pask	1	1	1	1	1	5
mmu-miR-223-3p	269233	Fam171a1	1	1	1	1	1	5
mmu-miR-223-3p	269593	Luzp1	1	1	1	1	1	5
mmu-miR-223-3p	269610	Chd5	1	1	1	1	1	5
mmu-miR-223-3p	269702	Mphosph9	1	1	1	1	1	5
mmu-miR-223-3p	269831	Tspan12	1	1	1	1	1	5
mmu-miR-223-3p	270160	Rab39	1	1	1	1	1	5
mmu-miR-223-3p	270624	Spin4	1	1	1	1	1	5
mmu-miR-223-3p	271144	Ankdd1b	1	1	1	1	1	5
mmu-miR-223-3p	271221	5031414D18Rik	1	1	1	1	1	5
mmu-miR-223-3p	271849	Shc4	1	1	1	1	1	5
mmu-miR-223-3p	272027	Tstd2	1	1	1	1	1	5
mmu-miR-223-3p	278279	Tmtc2	1	1	1	1	1	5
mmu-miR-223-3p	279572	Tlr13	1	1	1	1	1	5
mmu-miR-223-3p	319478	Cxxc4	1	1	1	1	1	5
mmu-miR-223-3p	319604	Fam168a	1	1	1	1	1	5
mmu-miR-223-3p	319642	Rab9b	1	1	1	1	1	5
mmu-miR-223-3p	319767	Atp10b	1	1	1	1	1	5
mmu-miR-223-3p	319832	Tmem229a	1	1	1	1	1	5
mmu-miR-223-3p	319880	Tmcc3	1	1	1	1	1	5
mmu-miR-223-3p	319888	Oacyl	1	1	1	1	1	5
mmu-miR-223-3p	319930	Ceacam19	1	1	1	1	1	5
mmu-miR-223-3p	319939	Tns3	1	1	1	1	1	5
mmu-miR-223-3p	319991	Kif6	1	1	1	1	1	5
mmu-miR-223-3p	320311	Rnf152	1	1	1	1	1	5
mmu-miR-223-3p	320343	Lypd6	1	1	1	1	1	5
mmu-miR-223-3p	320460	Vwc2l	1	1	1	1	1	5
mmu-miR-223-3p	320508	Cachd1	1	1	1	1	1	5
mmu-miR-223-3p	320560	Dennd5b	1	1	1	1	1	5

mmu-miR-223-3p	320595	Phf8	1	1	1	1	1	5
mmu-miR-223-3p	320634	Ocrl	1	1	1	1	1	5
mmu-miR-223-3p	320679	Samd12	1	1	1	1	1	5
mmu-miR-223-3p	320769	Prdx6b	1	1	1	1	1	5
mmu-miR-223-3p	327766	Tmem26	1	1	1	1	1	5
mmu-miR-223-3p	327826	Frs2	1	1	1	1	1	5
mmu-miR-223-3p	327957	Scimp	1	1	1	1	1	5
mmu-miR-223-3p	327987	Med13	1	1	1	1	1	5
mmu-miR-223-3p	328110	Prpf39	1	1	1	1	1	5
mmu-miR-223-3p	328232	Gfod1	1	1	1	1	1	5
mmu-miR-223-3p	328833	Treml2	1	1	1	1	1	5
mmu-miR-223-3p	329360	Gm757	1	1	1	1	1	5
mmu-miR-223-3p	329421	Myo3b	1	1	1	1	1	5
mmu-miR-223-3p	329910	Acot11	1	1	1	1	1	5
mmu-miR-223-3p	330502	Zfp82	1	1	1	1	1	5
mmu-miR-223-3p	330662	Dock1	1	1	1	1	1	5
mmu-miR-223-3p	330790	Hapln4	1	1	1	1	1	5
mmu-miR-223-3p	330836	Slc7a6	1	1	1	1	1	5
mmu-miR-223-3p	331401	Thoc2	1	1	1	1	1	5
mmu-miR-223-3p	331623	Bend3	1	1	1	1	1	5
mmu-miR-223-3p	338337	Cog3	1	1	1	1	1	5
mmu-miR-223-3p	338351	Akap17b	1	1	1	1	1	5
mmu-miR-223-3p	338362	Ust	1	1	1	1	1	5
mmu-miR-223-3p	338521	Fa2h	1	1	1	1	1	5
mmu-miR-223-3p	378431	Txlnb	1	1	1	1	1	5
mmu-miR-223-3p	380686	Cnrip1	1	1	1	1	1	5
mmu-miR-223-3p	380840	Lyrm4	1	1	1	1	1	5
mmu-miR-223-3p	380916	Lrch1	1	1	1	1	1	5
mmu-miR-223-3p	381157	Greb1l	1	1	1	1	1	5
mmu-miR-223-3p	381218	4430402I18Rik	1	1	1	1	1	5
mmu-miR-223-3p	381305	Rc3h1	1	1	1	1	1	5
mmu-miR-223-3p	381338	Lonrf2	1	1	1	1	1	5
mmu-miR-223-3p	381379	Med19	1	1	1	1	1	5
mmu-miR-223-3p	381560	Xkr8	1	1	1	1	1	5
mmu-miR-223-3p	381626	Rbm33	1	1	1	1	1	5
mmu-miR-223-3p	381644	Cep135	1	1	1	1	1	5
mmu-miR-223-3p	381695	N4bp2l2	1	1	1	1	1	5
mmu-miR-223-3p	381813	Prmt8	1	1	1	1	1	5
mmu-miR-223-3p	381816	4922502D21Rik	1	1	1	1	1	5
mmu-miR-223-3p	381823	Apold1	1	1	1	1	1	5
mmu-miR-223-3p	381983	Lmtk3	1	1	1	1	1	5
mmu-miR-223-3p	382083	Snx22	1	1	1	1	1	5
mmu-miR-223-3p	382090	4922501C03Rik	1	1	1	1	1	5
mmu-miR-223-3p	382620	Tmed8	1	1	1	1	1	5
mmu-miR-223-3p	385674	Zfp174	1	1	1	1	1	5
mmu-miR-223-3p	396184	Flrt1	1	1	1	1	1	5
mmu-miR-223-3p	399548	Scn4b	1	1	1	1	1	5
mmu-miR-223-3p	407821	Znrf3	1	1	1	1	1	5
mmu-miR-223-3p	408022	Ccdc111	1	1	1	1	1	5
mmu-miR-223-3p	408067	Zfp874b	1	1	1	1	1	5
mmu-miR-223-3p	432530	Adcy1	1	1	1	1	1	5
mmu-miR-223-3p	432572	Specc1	1	1	1	1	1	5
mmu-miR-223-3p	432879	Zbtbd6	1	1	1	1	1	5
mmu-miR-223-3p	433667	Ankrd13c	1	1	1	1	1	5
mmu-miR-223-3p	433938	Mn1	1	1	1	1	1	5

mmu-miR-223-3p	434008	Tmem178b	1	1	1	1	1	5
mmu-miR-223-3p	434203	Slc28a1	1	1	1	1	1	5
mmu-miR-223-3p	434232	Iqck	1	1	1	1	1	5
mmu-miR-223-3p	434396	Gm5615	1	1	1	1	1	5
mmu-miR-223-3p	435766	Tnni3k	1	1	1	1	1	5
mmu-miR-223-3p	494504	Apcdd1	1	1	1	1	1	5
mmu-miR-223-3p	545389	Cep170	1	1	1	1	1	5
mmu-miR-223-3p	545662	B020004J07Rik	1	1	1	1	1	5
mmu-miR-223-3p	545812	Pilrb2	1	1	1	1	1	5
mmu-miR-223-3p	545902	Ptprh	1	1	1	1	1	5
mmu-miR-223-3p	546157	7420426K07Rik	1	1	1	1	1	5
mmu-miR-223-3p	547431	Btnl2	1	1	1	1	1	5
mmu-miR-223-3p	574437	Xlr3b	1	1	1	1	1	5
mmu-miR-223-3p	622320	Kctd21	1	1	1	1	1	5
mmu-miR-223-3p	622434	Arhgef26	1	1	1	1	1	5
mmu-miR-223-3p	622976	Gm6377	1	1	1	1	1	5
mmu-miR-223-3p	624855	Gm6531	1	1	1	1	1	5
mmu-miR-223-3p	654821	Gcnt7	1	1	1	1	1	5
mmu-miR-223-3p	666528	Zfp541	1	1	1	1	1	5
mmu-miR-223-3p	668212	Efr3b	1	1	1	1	1	5
mmu-miR-223-3p	11304	Abca4	1	1	1	0	1	4
mmu-miR-223-3p	11431	Acp1	1	1	0	1	1	4
mmu-miR-223-3p	11488	Adam11	1	1	0	1	1	4
mmu-miR-223-3p	11499	Adam5	1	0	1	1	1	4
mmu-miR-223-3p	11513	Adcy7	1	1	0	1	1	4
mmu-miR-223-3p	11542	Adora3	1	1	0	1	1	4
mmu-miR-223-3p	11569	Aebp2	1	1	0	1	1	4
mmu-miR-223-3p	11610	Agtrap	1	0	1	1	1	4
mmu-miR-223-3p	11625	Ahsg	1	1	1	0	1	4
mmu-miR-223-3p	11694	Alx3	0	1	1	1	1	4
mmu-miR-223-3p	11828	Aqp3	1	1	1	1	0	4
mmu-miR-223-3p	11829	Aqp4	1	0	1	1	1	4
mmu-miR-223-3p	11845	Arf6	1	1	1	1	0	4
mmu-miR-223-3p	11920	Atm	1	0	1	1	1	4
mmu-miR-223-3p	11951	Atp5g1	1	0	1	1	1	4
mmu-miR-223-3p	11977	Atp7a	1	1	0	1	1	4
mmu-miR-223-3p	11988	Slc7a2	1	1	0	1	1	4
mmu-miR-223-3p	11994	Pcdh15	1	0	1	1	1	4
mmu-miR-223-3p	12035	Bcat1	1	1	0	1	1	4
mmu-miR-223-3p	12048	Bcl2l1	1	0	1	1	1	4
mmu-miR-223-3p	12050	Bcl2l2	1	1	1	1	0	4
mmu-miR-223-3p	12123	Hrk	1	0	1	1	1	4
mmu-miR-223-3p	12167	Bmpr1b	1	0	1	1	1	4
mmu-miR-223-3p	12169	Bmx	1	1	1	0	1	4
mmu-miR-223-3p	12180	Smyd1	1	1	0	1	1	4
mmu-miR-223-3p	12183	Bpgm	1	0	1	1	1	4
mmu-miR-223-3p	12212	Chic1	1	1	0	1	1	4
mmu-miR-223-3p	12234	Btrc	1	1	0	1	1	4
mmu-miR-223-3p	12311	Calcr	1	1	0	1	1	4
mmu-miR-223-3p	12313	Calm1	1	0	1	1	1	4
mmu-miR-223-3p	12325	Camk2g	1	1	0	1	1	4
mmu-miR-223-3p	12337	Capn5	1	1	1	1	0	4
mmu-miR-223-3p	12338	Capn6	1	1	1	0	1	4
mmu-miR-223-3p	12361	Cask	1	0	1	1	1	4
mmu-miR-223-3p	12371	Casp9	1	0	1	1	1	4

mmu-miR-223-3p	12400	Cbfb	1	0	1	1	1	4
mmu-miR-223-3p	12418	Cbx4	1	0	1	1	1	4
mmu-miR-223-3p	12419	Cbx5	1	1	0	1	1	4
mmu-miR-223-3p	12462	Cct3	1	0	1	1	1	4
mmu-miR-223-3p	12469	Cct8	1	0	1	1	1	4
mmu-miR-223-3p	12490	Cd34	1	1	0	1	1	4
mmu-miR-223-3p	12495	Entpd1	1	1	1	1	0	4
mmu-miR-223-3p	12499	Entpd5	1	1	0	1	1	4
mmu-miR-223-3p	12503	Cd247	1	0	1	1	1	4
mmu-miR-223-3p	12562	Cdh5	1	1	1	0	1	4
mmu-miR-223-3p	12566	Cdk2	1	1	0	1	1	4
mmu-miR-223-3p	12569	Cdk5r1	1	1	1	1	0	4
mmu-miR-223-3p	12572	Cdk7	1	1	1	1	0	4
mmu-miR-223-3p	12585	Cdr2	1	0	1	1	1	4
mmu-miR-223-3p	12591	Cdx2	1	1	1	1	0	4
mmu-miR-223-3p	12611	Cebpg	1	0	1	1	1	4
mmu-miR-223-3p	12623	Ces1g	1	0	1	1	1	4
mmu-miR-223-3p	12663	Chml	1	0	1	1	1	4
mmu-miR-223-3p	12725	Clcn3	1	1	0	1	1	4
mmu-miR-223-3p	12753	Clock	1	1	1	1	0	4
mmu-miR-223-3p	12766	Cxcr3	1	0	1	1	1	4
mmu-miR-223-3p	12874	Cpd	1	1	1	1	0	4
mmu-miR-223-3p	12894	Cpt1a	1	0	1	1	1	4
mmu-miR-223-3p	12921	Crhr1	1	0	1	1	1	4
mmu-miR-223-3p	12928	Crk	1	1	1	1	0	4
mmu-miR-223-3p	12933	Crmp1	1	0	1	1	1	4
mmu-miR-223-3p	12936	Pcdha4	1	1	0	1	1	4
mmu-miR-223-3p	12942	Pcdha11	1	1	0	1	1	4
mmu-miR-223-3p	12943	Pcdha10	1	1	0	1	1	4
mmu-miR-223-3p	12971	Crym	1	0	1	1	1	4
mmu-miR-223-3p	12982	Csf2ra	1	0	1	1	1	4
mmu-miR-223-3p	12991	Csn2	1	0	1	1	1	4
mmu-miR-223-3p	13046	Celf1	1	1	0	1	1	4
mmu-miR-223-3p	13058	Cybb	1	0	1	1	1	4
mmu-miR-223-3p	13119	Cyp4a14	1	0	1	1	1	4
mmu-miR-223-3p	13131	Dab1	1	1	1	1	0	4
mmu-miR-223-3p	13176	Dcc	1	0	1	1	1	4
mmu-miR-223-3p	13371	Dio2	1	1	1	1	0	4
mmu-miR-223-3p	13383	Dlg1	1	0	1	1	1	4
mmu-miR-223-3p	13400	Dmpk	1	1	0	1	1	4
mmu-miR-223-3p	13421	Dnase1l3	1	1	1	1	0	4
mmu-miR-223-3p	13496	Arid3a	1	1	1	1	0	4
mmu-miR-223-3p	13497	Drp2	1	0	1	1	1	4
mmu-miR-223-3p	13522	Adam28	1	0	1	1	1	4
mmu-miR-223-3p	13527	Dtna	1	0	1	1	1	4
mmu-miR-223-3p	13544	Dvl3	1	0	1	1	1	4
mmu-miR-223-3p	13593	Ebf3	1	1	0	1	1	4
mmu-miR-223-3p	13609	S1pr1	1	1	1	0	1	4
mmu-miR-223-3p	13610	S1pr3	1	1	1	1	0	4
mmu-miR-223-3p	13649	Egfr	1	0	1	1	1	4
mmu-miR-223-3p	13654	Egr2	1	1	1	1	0	4
mmu-miR-223-3p	13690	Eif4g2	1	1	0	1	1	4
mmu-miR-223-3p	13829	Dmtn	1	0	1	1	1	4
mmu-miR-223-3p	13982	Esr1	1	0	1	1	1	4
mmu-miR-223-3p	14007	Celf2	1	1	0	1	1	4

mmu-miR-223-3p	14050	Eya3	1	1	0	1	1	4
mmu-miR-223-3p	14051	Eya4	1	1	1	1	0	4
mmu-miR-223-3p	14055	Ezh1	1	1	1	0	1	4
mmu-miR-223-3p	14084	Faf1	1	0	1	1	1	4
mmu-miR-223-3p	14107	Fat1	0	1	1	1	1	4
mmu-miR-223-3p	14155	Fem1b	1	1	1	1	0	4
mmu-miR-223-3p	14164	Fgf1	1	1	1	1	0	4
mmu-miR-223-3p	14178	Fgf7	1	1	1	1	0	4
mmu-miR-223-3p	14183	Fgfr2	1	1	0	1	1	4
mmu-miR-223-3p	14190	Fgl2	1	0	1	1	1	4
mmu-miR-223-3p	14299	Ncs1	1	1	1	1	0	4
mmu-miR-223-3p	14344	Fut2	1	0	1	1	1	4
mmu-miR-223-3p	14345	Fut4	1	0	1	1	1	4
mmu-miR-223-3p	14389	Gab2	1	1	0	1	1	4
mmu-miR-223-3p	14399	Gabra6	1	1	0	1	1	4
mmu-miR-223-3p	14402	Gabrb3	1	1	0	1	1	4
mmu-miR-223-3p	14406	Gabrg2	1	0	1	1	1	4
mmu-miR-223-3p	14525	Gcsam	1	0	1	1	1	4
mmu-miR-223-3p	14538	Gcnt2	1	1	0	1	1	4
mmu-miR-223-3p	14583	Gfpt1	1	1	0	1	1	4
mmu-miR-223-3p	14593	Ggps1	1	0	1	1	1	4
mmu-miR-223-3p	14595	B4galt1	1	0	1	1	1	4
mmu-miR-223-3p	14672	Gna11	1	0	1	1	1	4
mmu-miR-223-3p	14680	Gnal	1	1	0	1	1	4
mmu-miR-223-3p	14701	Gng12	1	0	1	1	1	4
mmu-miR-223-3p	14725	Lrp2	1	1	1	1	0	4
mmu-miR-223-3p	14745	Lpar1	1	1	0	1	1	4
mmu-miR-223-3p	14758	Gpm6b	1	0	1	1	1	4
mmu-miR-223-3p	14768	Lancl1	1	0	1	1	1	4
mmu-miR-223-3p	14800	Gria2	1	1	0	1	1	4
mmu-miR-223-3p	14807	Grik3	1	0	1	1	1	4
mmu-miR-223-3p	14816	Grm1	1	0	1	1	1	4
mmu-miR-223-3p	14852	Gspt1	1	0	1	1	1	4
mmu-miR-223-3p	14897	Trip12	1	0	1	1	1	4
mmu-miR-223-3p	14917	Gucy2c	1	0	1	1	1	4
mmu-miR-223-3p	14933	Gyk	1	1	0	1	1	4
mmu-miR-223-3p	14957	Hist1h1d	1	0	1	1	1	4
mmu-miR-223-3p	15015	H2-Q4	1	1	0	1	1	4
mmu-miR-223-3p	15194	Htt	1	1	0	1	1	4
mmu-miR-223-3p	15402	Hoxa5	1	1	1	1	0	4
mmu-miR-223-3p	15432	Hoxd12	1	1	1	0	1	4
mmu-miR-223-3p	15460	Hr	1	0	1	1	1	4
mmu-miR-223-3p	15482	Hspa1l	1	0	1	1	1	4
mmu-miR-223-3p	15502	Dnaja1	1	0	1	1	1	4
mmu-miR-223-3p	15529	Sdc2	0	1	1	1	1	4
mmu-miR-223-3p	15550	Htr1a	1	0	1	1	1	4
mmu-miR-223-3p	15567	Slc6a4	1	1	1	1	0	4
mmu-miR-223-3p	15571	Elavl3	1	1	0	1	1	4
mmu-miR-223-3p	15931	Ids	1	1	0	1	1	4
mmu-miR-223-3p	15950	Ifi203	1	1	0	1	1	4
mmu-miR-223-3p	16000	Igf1	1	1	0	1	1	4
mmu-miR-223-3p	16164	Il13ra1	1	1	1	1	0	4
mmu-miR-223-3p	16177	Il1r1	1	1	0	1	1	4
mmu-miR-223-3p	16190	Il4ra	1	0	1	1	1	4
mmu-miR-223-3p	16193	Il6	1	1	1	0	1	4

mmu-miR-223-3p	16211	Kpnb1	1	1	1	1	0	4
mmu-miR-223-3p	16324	Inhbb	1	0	1	1	1	4
mmu-miR-223-3p	16326	Inhbe	1	0	1	1	1	4
mmu-miR-223-3p	16408	Itgal	1	0	1	1	1	4
mmu-miR-223-3p	16413	Itgb1bp1	1	0	1	1	1	4
mmu-miR-223-3p	16420	Itgb6	1	0	1	1	1	4
mmu-miR-223-3p	16440	Itpr3	1	1	1	0	1	4
mmu-miR-223-3p	16443	Itsn1	1	0	1	1	1	4
mmu-miR-223-3p	16509	Kcne1	1	0	1	1	1	4
mmu-miR-223-3p	16510	Kcnh1	1	1	0	1	1	4
mmu-miR-223-3p	16522	Kcnj6	1	0	1	1	1	4
mmu-miR-223-3p	16531	Kcnma1	1	1	0	1	1	4
mmu-miR-223-3p	16553	Kif13a	1	0	1	1	1	4
mmu-miR-223-3p	16560	Kif1a	1	1	0	1	1	4
mmu-miR-223-3p	16562	Kif1c	1	0	1	1	1	4
mmu-miR-223-3p	16571	Kif4	1	1	1	0	1	4
mmu-miR-223-3p	16593	Klc1	1	0	1	1	1	4
mmu-miR-223-3p	16597	Klf12	1	0	1	1	1	4
mmu-miR-223-3p	16648	Kpna3	1	1	0	1	1	4
mmu-miR-223-3p	16653	Kras	1	0	1	1	1	4
mmu-miR-223-3p	16701	Krtap6-2	1	0	1	1	1	4
mmu-miR-223-3p	16764	Aff3	1	0	1	1	1	4
mmu-miR-223-3p	16871	Lhx3	1	0	1	1	1	4
mmu-miR-223-3p	16874	Lhx6	1	1	0	1	1	4
mmu-miR-223-3p	16878	Lif	1	1	0	1	1	4
mmu-miR-223-3p	16880	Lifr	1	1	1	1	0	4
mmu-miR-223-3p	16907	Lmnb2	1	0	1	1	1	4
mmu-miR-223-3p	16975	Lrp8	1	1	0	1	1	4
mmu-miR-223-3p	17059	Klrb1c	1	0	1	1	1	4
mmu-miR-223-3p	17064	Cd93	1	0	1	1	1	4
mmu-miR-223-3p	17069	Ly6e	1	0	1	1	1	4
mmu-miR-223-3p	17125	Smad1	1	0	1	1	1	4
mmu-miR-223-3p	17126	Smad2	1	0	1	1	1	4
mmu-miR-223-3p	17127	Smad3	1	0	1	1	1	4
mmu-miR-223-3p	17156	Man1a2	1	0	1	1	1	4
mmu-miR-223-3p	17164	Mapkapk2	1	1	1	0	1	4
mmu-miR-223-3p	17199	Mc1r	1	1	1	1	0	4
mmu-miR-223-3p	17246	Mdm2	1	0	1	1	1	4
mmu-miR-223-3p	17257	Mecp2	1	1	0	1	1	4
mmu-miR-223-3p	17260	Mef2c	1	1	0	1	1	4
mmu-miR-223-3p	17274	Rab8a	1	1	1	1	0	4
mmu-miR-223-3p	17341	Bhlha15	1	0	1	1	1	4
mmu-miR-223-3p	17342	Mitf	1	1	0	1	1	4
mmu-miR-223-3p	17354	Mllt10	1	0	1	1	1	4
mmu-miR-223-3p	17364	Trpm1	1	0	1	1	1	4
mmu-miR-223-3p	17389	Mmp16	1	1	0	1	1	4
mmu-miR-223-3p	17756	Map2	1	1	0	1	1	4
mmu-miR-223-3p	17758	Map4	1	0	1	1	1	4
mmu-miR-223-3p	17760	Map6	1	0	1	1	1	4
mmu-miR-223-3p	17868	Mybpc3	0	1	1	1	1	4
mmu-miR-223-3p	17869	Myc	1	0	1	1	1	4
mmu-miR-223-3p	17913	Myo1c	1	1	0	1	1	4
mmu-miR-223-3p	17918	Myo5a	1	1	1	1	0	4
mmu-miR-223-3p	17926	Myoc	1	0	1	1	1	4
mmu-miR-223-3p	17951	Naip5	1	0	1	1	1	4

mmu-miR-223-3p	17988	Ndrg1	1	1	1	1	0	4
mmu-miR-223-3p	17999	Nedd4	1	0	1	1	1	4
mmu-miR-223-3p	18000	2-Sep	1	0	1	1	1	4
mmu-miR-223-3p	18027	Nfia	1	1	0	1	1	4
mmu-miR-223-3p	18028	Nfib	1	1	0	1	1	4
mmu-miR-223-3p	18037	Nfkbie	1	0	1	1	1	4
mmu-miR-223-3p	18044	Nfya	1	1	0	1	1	4
mmu-miR-223-3p	18071	Nhlh1	1	0	1	1	1	4
mmu-miR-223-3p	18140	Uhrf1	1	1	0	1	1	4
mmu-miR-223-3p	18181	Nrf1	1	0	1	1	1	4
mmu-miR-223-3p	18188	Nrtn	1	1	1	0	1	4
mmu-miR-223-3p	18191	Nrxn3	1	0	1	1	1	4
mmu-miR-223-3p	18197	Nsg2	1	1	1	1	0	4
mmu-miR-223-3p	18212	Ntrk2	1	0	1	1	1	4
mmu-miR-223-3p	18230	Nxn	1	0	1	1	1	4
mmu-miR-223-3p	18292	Sebox	1	0	1	1	1	4
mmu-miR-223-3p	18293	Ogdh	1	0	1	1	1	4
mmu-miR-223-3p	18475	Pafah1b2	1	0	1	1	1	4
mmu-miR-223-3p	18481	Pak3	1	1	0	1	1	4
mmu-miR-223-3p	18509	Pax7	1	0	1	1	1	4
mmu-miR-223-3p	18526	Pcdh10	1	0	1	1	1	4
mmu-miR-223-3p	18557	Cdk18	1	1	1	1	0	4
mmu-miR-223-3p	18576	Pde3b	1	1	1	0	1	4
mmu-miR-223-3p	18577	Pde4a	1	1	0	1	1	4
mmu-miR-223-3p	18578	Pde4b	1	1	1	1	0	4
mmu-miR-223-3p	18595	Pdgfra	1	1	0	1	1	4
mmu-miR-223-3p	18628	Per3	1	0	1	1	1	4
mmu-miR-223-3p	18667	Pgr	1	0	1	1	1	4
mmu-miR-223-3p	18679	Phka1	1	1	0	1	1	4
mmu-miR-223-3p	18706	Pik3ca	1	1	0	1	1	4
mmu-miR-223-3p	18711	Pikfyve	1	0	1	1	1	4
mmu-miR-223-3p	18718	Pip4k2a	1	1	1	1	0	4
mmu-miR-223-3p	18747	Prkaca	1	0	1	1	1	4
mmu-miR-223-3p	18751	Prkcb	1	0	1	1	1	4
mmu-miR-223-3p	18769	Pkig	1	1	0	1	1	4
mmu-miR-223-3p	18969	Pola2	1	0	1	1	1	4
mmu-miR-223-3p	18986	Pou2f1	1	1	0	1	1	4
mmu-miR-223-3p	19025	Ctsa	1	1	0	1	1	4
mmu-miR-223-3p	19027	Sypl	1	1	0	1	1	4
mmu-miR-223-3p	19088	Prkar2b	1	1	1	0	1	4
mmu-miR-223-3p	19091	Prkg1	1	0	1	1	1	4
mmu-miR-223-3p	19116	Prlr	1	1	0	1	1	4
mmu-miR-223-3p	19201	Pstpip2	1	0	1	1	1	4
mmu-miR-223-3p	19219	Ptger4	1	1	0	1	1	4
mmu-miR-223-3p	19220	Ptgfr	1	0	1	1	1	4
mmu-miR-223-3p	19221	Ptgfrn	1	1	1	1	0	4
mmu-miR-223-3p	19245	Ptp4a3	1	0	1	1	1	4
mmu-miR-223-3p	19252	Dusp1	1	1	1	0	1	4
mmu-miR-223-3p	19263	Ptprb	1	1	0	1	1	4
mmu-miR-223-3p	19267	Ptpre	1	0	1	1	1	4
mmu-miR-223-3p	19291	Purb	1	1	0	1	1	4
mmu-miR-223-3p	19324	Rab1	1	1	1	1	0	4
mmu-miR-223-3p	19326	Rab11b	1	0	1	1	1	4
mmu-miR-223-3p	19334	Rab22a	1	0	1	1	1	4
mmu-miR-223-3p	19345	Rab5c	1	1	1	0	1	4

mmu-miR-223-3p	19646	Rbbp4	1	1	1	1	0	4
mmu-miR-223-3p	19661	Rbp3	1	0	1	1	1	4
mmu-miR-223-3p	19664	Rbpj	1	1	0	1	1	4
mmu-miR-223-3p	19730	Ralgds	1	0	1	1	1	4
mmu-miR-223-3p	20147	Rs1	1	0	1	1	1	4
mmu-miR-223-3p	20181	Rxra	1	0	1	1	1	4
mmu-miR-223-3p	20185	Ncor1	1	1	0	1	1	4
mmu-miR-223-3p	20218	Khdrbs1	1	0	1	1	1	4
mmu-miR-223-3p	20238	Atxn1	1	1	0	1	1	4
mmu-miR-223-3p	20239	Atxn2	1	1	1	0	1	4
mmu-miR-223-3p	20273	Scn8a	1	1	0	1	1	4
mmu-miR-223-3p	20309	Cxcl15	1	1	1	0	1	4
mmu-miR-223-3p	20318	Sdf4	1	0	1	1	1	4
mmu-miR-223-3p	20346	Sema3a	1	0	1	1	1	4
mmu-miR-223-3p	20348	Sema3c	1	1	1	1	0	4
mmu-miR-223-3p	20349	Sema3e	1	0	1	1	1	4
mmu-miR-223-3p	20358	Sema6a	1	1	1	1	0	4
mmu-miR-223-3p	20382	Srsf2	1	1	1	1	0	4
mmu-miR-223-3p	20402	Zfp106	1	0	1	1	1	4
mmu-miR-223-3p	20452	St8sia4	1	1	1	1	0	4
mmu-miR-223-3p	20471	Six1	1	1	1	1	0	4
mmu-miR-223-3p	20513	Slc1a6	1	1	1	0	1	4
mmu-miR-223-3p	20526	Slc2a2	1	0	1	1	1	4
mmu-miR-223-3p	20529	Slc31a1	1	0	1	1	1	4
mmu-miR-223-3p	20603	Sms	1	1	1	1	0	4
mmu-miR-223-3p	20652	Soat1	1	0	1	1	1	4
mmu-miR-223-3p	20658	Son	1	0	1	1	1	4
mmu-miR-223-3p	20662	Sos1	1	1	1	1	0	4
mmu-miR-223-3p	20666	Sox11	1	0	1	1	1	4
mmu-miR-223-3p	20679	Sox6	1	1	0	1	1	4
mmu-miR-223-3p	20680	Sox7	1	0	1	1	1	4
mmu-miR-223-3p	20683	Sp1	1	1	1	1	0	4
mmu-miR-223-3p	20687	Sp3	1	1	0	1	1	4
mmu-miR-223-3p	20713	Serpini1	1	0	1	1	1	4
mmu-miR-223-3p	20725	Serpinb8	0	1	1	1	1	4
mmu-miR-223-3p	20741	Sptb	1	1	1	1	0	4
mmu-miR-223-3p	20742	Sptbn1	1	0	1	1	1	4
mmu-miR-223-3p	20744	Strbp	1	1	0	1	1	4
mmu-miR-223-3p	20779	Src	1	1	0	1	1	4
mmu-miR-223-3p	20821	Trim21	1	1	0	1	1	4
mmu-miR-223-3p	20826	Nhp2l1	1	0	1	1	1	4
mmu-miR-223-3p	20842	Stag1	1	1	1	1	0	4
mmu-miR-223-3p	20848	Stat3	1	1	0	1	1	4
mmu-miR-223-3p	20856	Stc2	1	1	1	0	1	4
mmu-miR-223-3p	20874	Slk	1	1	0	1	1	4
mmu-miR-223-3p	20913	Stxbp4	1	1	0	1	1	4
mmu-miR-223-3p	20970	Sdc3	1	0	1	1	1	4
mmu-miR-223-3p	21349	Tal1	1	0	1	1	1	4
mmu-miR-223-3p	21388	Tbx5	1	1	0	1	1	4
mmu-miR-223-3p	21410	Hnf1b	1	1	1	0	1	4
mmu-miR-223-3p	21418	Tfap2a	1	1	0	1	1	4
mmu-miR-223-3p	21429	Ubtf	1	1	0	1	1	4
mmu-miR-223-3p	21454	Tcp1	1	0	1	1	1	4
mmu-miR-223-3p	21685	Tef	1	1	0	1	1	4
mmu-miR-223-3p	21778	Tex9	1	1	0	1	1	4

mmu-miR-223-3p	21834	Thrb	1	1	0	1	1	4
mmu-miR-223-3p	21873	Tjp2	1	0	1	1	1	4
mmu-miR-223-3p	21888	Tle4	1	1	1	1	0	4
mmu-miR-223-3p	21938	Tnfrsf1b	1	0	1	1	1	4
mmu-miR-223-3p	21949	Tnfsf8	1	1	1	1	0	4
mmu-miR-223-3p	21951	Tnks	1	1	1	1	0	4
mmu-miR-223-3p	21961	Tns1	1	1	0	1	1	4
mmu-miR-223-3p	21975	Top3a	1	0	1	1	1	4
mmu-miR-223-3p	21983	Tpbg	1	1	0	1	1	4
mmu-miR-223-3p	22004	Tpm2	1	1	1	1	0	4
mmu-miR-223-3p	22027	Hsp90b1	1	1	1	0	1	4
mmu-miR-223-3p	22084	Tsc2	1	1	0	1	1	4
mmu-miR-223-3p	22088	Tsg101	1	1	1	1	0	4
mmu-miR-223-3p	22134	Tgoln1	1	1	1	1	0	4
mmu-miR-223-3p	22141	Tub	1	1	1	1	0	4
mmu-miR-223-3p	22156	Tuft1	1	1	1	1	0	4
mmu-miR-223-3p	22160	Twist1	1	0	1	1	1	4
mmu-miR-223-3p	22195	Ube2l3	1	0	1	1	1	4
mmu-miR-223-3p	22196	Ube2i	1	0	1	1	1	4
mmu-miR-223-3p	22210	Ube2b	1	0	1	1	1	4
mmu-miR-223-3p	22230	Ufd1l	1	1	1	1	0	4
mmu-miR-223-3p	22262	Uox	1	1	1	1	0	4
mmu-miR-223-3p	22295	Cdh23	1	1	0	1	1	4
mmu-miR-223-3p	22318	Vamp2	1	1	1	0	1	4
mmu-miR-223-3p	22319	Vamp3	1	1	1	1	0	4
mmu-miR-223-3p	22333	Vdac1	0	1	1	1	1	4
mmu-miR-223-3p	22375	Wars	1	1	0	1	1	4
mmu-miR-223-3p	22422	Wnt7b	1	0	1	1	1	4
mmu-miR-223-3p	22589	Atrx	1	1	1	1	0	4
mmu-miR-223-3p	22629	Ywhah	1	1	1	0	1	4
mmu-miR-223-3p	22631	Ywhaz	1	0	1	1	1	4
mmu-miR-223-3p	22696	Zfp37	1	0	1	1	1	4
mmu-miR-223-3p	22698	Zfp39	1	0	1	1	1	4
mmu-miR-223-3p	22704	Zfp46	1	0	1	1	1	4
mmu-miR-223-3p	22754	Zfp92	1	0	1	1	1	4
mmu-miR-223-3p	22758	Zscan12	1	0	1	1	1	4
mmu-miR-223-3p	22764	Zfx	1	1	0	1	1	4
mmu-miR-223-3p	22767	Zfy1	1	1	1	0	1	4
mmu-miR-223-3p	23792	Adam23	1	0	1	1	1	4
mmu-miR-223-3p	23821	Bace1	1	1	1	1	0	4
mmu-miR-223-3p	23849	Klf6	1	1	1	1	0	4
mmu-miR-223-3p	23856	Dido1	1	0	1	1	1	4
mmu-miR-223-3p	23859	Dlg2	1	1	1	1	0	4
mmu-miR-223-3p	23888	Gpc6	1	1	0	1	1	4
mmu-miR-223-3p	23912	Rhof	1	1	1	0	1	4
mmu-miR-223-3p	23936	Lynx1	1	1	1	1	0	4
mmu-miR-223-3p	23945	Mgll	1	0	1	1	1	4
mmu-miR-223-3p	23984	Pde10a	1	0	1	1	1	4
mmu-miR-223-3p	24086	Tlk2	1	1	0	1	1	4
mmu-miR-223-3p	26377	Dapp1	1	0	1	1	1	4
mmu-miR-223-3p	26381	Esrrg	1	0	1	1	1	4
mmu-miR-223-3p	26383	Fto	1	0	1	1	1	4
mmu-miR-223-3p	26401	Map3k1	1	1	1	1	0	4
mmu-miR-223-3p	26404	Map3k12	1	0	1	1	1	4
mmu-miR-223-3p	26409	Map3k7	1	0	1	1	1	4

mmu-miR-223-3p	26420	Mapk9	1	1	0	1	1	4
mmu-miR-223-3p	26422	Nbea	0	1	1	1	1	4
mmu-miR-223-3p	26450	Rbbp9	1	0	1	1	1	4
mmu-miR-223-3p	26558	Homer3	1	1	0	1	1	4
mmu-miR-223-3p	26559	Hunk	1	0	1	1	1	4
mmu-miR-223-3p	26569	Slc27a4	1	0	1	1	1	4
mmu-miR-223-3p	26934	Racgap1	0	1	1	1	1	4
mmu-miR-223-3p	27041	G3bp1	1	1	1	0	1	4
mmu-miR-223-3p	27081	Zfp275	1	0	1	1	1	4
mmu-miR-223-3p	27215	Azi2	1	0	1	1	1	4
mmu-miR-223-3p	27373	Csnk1e	1	1	1	1	0	4
mmu-miR-223-3p	27382	Tcl1b5	1	1	1	1	0	4
mmu-miR-223-3p	27397	Mrpl17	1	0	1	1	1	4
mmu-miR-223-3p	27406	Abcf3	1	0	1	1	1	4
mmu-miR-223-3p	28000	Prpf19	1	1	0	1	1	4
mmu-miR-223-3p	28109	D10Wsu102e	1	0	1	1	1	4
mmu-miR-223-3p	28248	Slco1a1	1	1	1	0	1	4
mmu-miR-223-3p	29807	Tpk1	1	1	1	0	1	4
mmu-miR-223-3p	29864	Rnf11	1	1	1	0	1	4
mmu-miR-223-3p	30046	Zfp292	1	0	1	1	1	4
mmu-miR-223-3p	30050	Fbxw2	1	0	1	1	1	4
mmu-miR-223-3p	30928	Zbtb18	1	1	0	1	1	4
mmu-miR-223-3p	30934	Tor1b	1	0	1	1	1	4
mmu-miR-223-3p	30936	Slc46a2	1	1	1	0	1	4
mmu-miR-223-3p	30956	Aass	1	1	1	0	1	4
mmu-miR-223-3p	50493	Txnrd1	1	1	0	1	1	4
mmu-miR-223-3p	50523	Lats2	1	1	1	1	0	4
mmu-miR-223-3p	50530	Mfap5	1	0	1	1	1	4
mmu-miR-223-3p	50754	Fbxw7	1	1	0	1	1	4
mmu-miR-223-3p	50766	Crim1	1	1	0	1	1	4
mmu-miR-223-3p	50780	Rgs3	1	0	1	1	1	4
mmu-miR-223-3p	50786	Hs6st2	1	1	0	1	1	4
mmu-miR-223-3p	50793	Orc3	1	0	1	1	1	4
mmu-miR-223-3p	50875	Tmod3	1	0	1	1	1	4
mmu-miR-223-3p	50907	Preb	1	1	1	1	0	4
mmu-miR-223-3p	51902	Rnf24	1	1	1	1	0	4
mmu-miR-223-3p	52028	Bbs1	1	1	1	1	0	4
mmu-miR-223-3p	52705	Krr1	1	1	0	1	1	4
mmu-miR-223-3p	52846	Cnot11	1	1	1	0	1	4
mmu-miR-223-3p	52855	Lair1	1	1	0	1	1	4
mmu-miR-223-3p	53323	Ube2k	1	0	1	1	1	4
mmu-miR-223-3p	53328	Pgrmc1	1	1	1	0	1	4
mmu-miR-223-3p	53330	Vamp4	1	0	1	1	1	4
mmu-miR-223-3p	53357	Pla2g6	1	0	1	1	1	4
mmu-miR-223-3p	53378	Sdcbp	1	1	0	1	1	4
mmu-miR-223-3p	53604	Zpbp	1	1	0	1	1	4
mmu-miR-223-3p	53883	Celsr2	1	1	0	1	1	4
mmu-miR-223-3p	54170	Rragc	1	1	1	1	0	4
mmu-miR-223-3p	54215	Cd160	1	0	1	1	1	4
mmu-miR-223-3p	54371	Chst2	1	1	1	1	0	4
mmu-miR-223-3p	54446	Nfat5	1	1	0	1	1	4
mmu-miR-223-3p	54598	Calcrl	1	1	1	1	0	4
mmu-miR-223-3p	54650	Sfmbt1	1	1	0	1	1	4
mmu-miR-223-3p	54712	Plxnc1	1	1	1	1	0	4
mmu-miR-223-3p	54722	Dfna5	1	0	1	1	1	4

mmu-miR-223-3p	55936	Ctps2	1	0	1	1	1	4
mmu-miR-223-3p	55943	Stx8	1	0	1	1	1	4
mmu-miR-223-3p	55963	Slc1a4	1	0	1	1	1	4
mmu-miR-223-3p	56044	Rala	1	1	1	1	0	4
mmu-miR-223-3p	56046	Uqcc	1	1	1	0	1	4
mmu-miR-223-3p	56077	Dgke	1	1	1	1	0	4
mmu-miR-223-3p	56291	Styx	1	0	1	1	1	4
mmu-miR-223-3p	56307	Metap2	1	0	1	1	1	4
mmu-miR-223-3p	56309	Mycbp	1	1	1	0	1	4
mmu-miR-223-3p	56356	Gltp	1	0	1	1	1	4
mmu-miR-223-3p	56368	Cyb561d2	1	0	1	1	1	4
mmu-miR-223-3p	56376	Pdlim5	1	0	1	1	1	4
mmu-miR-223-3p	56379	Kcnj1	1	0	1	1	1	4
mmu-miR-223-3p	56461	Kcnip3	1	1	0	1	1	4
mmu-miR-223-3p	56468	Socs5	1	1	1	1	0	4
mmu-miR-223-3p	56492	Cldn18	1	1	0	1	1	4
mmu-miR-223-3p	56515	Rnf138	1	1	0	1	1	4
mmu-miR-223-3p	56516	Rbms2	1	1	0	1	1	4
mmu-miR-223-3p	56526	Sept6	1	1	0	1	1	4
mmu-miR-223-3p	56532	Ripk3	1	0	1	1	1	4
mmu-miR-223-3p	56543	Kcnd3	1	0	1	1	1	4
mmu-miR-223-3p	56695	Pnkd	1	0	1	1	1	4
mmu-miR-223-3p	56715	Rabgef1	1	0	1	1	1	4
mmu-miR-223-3p	56716	Mlst8	1	0	1	1	1	4
mmu-miR-223-3p	56736	Rnf14	1	0	1	1	1	4
mmu-miR-223-3p	56745	C1qtnf1	1	0	1	1	1	4
mmu-miR-223-3p	56786	Tmem9b	1	1	1	1	0	4
mmu-miR-223-3p	56839	Lgi1	1	1	1	1	0	4
mmu-miR-223-3p	57230	Sap30bp	1	0	1	1	1	4
mmu-miR-223-3p	57257	Vav3	1	1	0	1	1	4
mmu-miR-223-3p	57261	Brd4	1	1	1	1	0	4
mmu-miR-223-3p	57390	Psors1c2	1	1	1	0	1	4
mmu-miR-223-3p	57748	Jmy	1	1	0	1	1	4
mmu-miR-223-3p	57749	Piwil1	1	1	1	0	1	4
mmu-miR-223-3p	57912	Cdc42se1	1	1	1	1	0	4
mmu-miR-223-3p	57915	Tbc1d1	1	1	1	1	0	4
mmu-miR-223-3p	58172	Sertad2	1	1	0	1	1	4
mmu-miR-223-3p	58193	Extl2	1	0	1	1	1	4
mmu-miR-223-3p	58207	Slc43a3	1	0	1	1	1	4
mmu-miR-223-3p	58220	Pard6b	1	1	0	1	1	4
mmu-miR-223-3p	59026	Huwe1	1	1	1	0	1	4
mmu-miR-223-3p	59027	Nampt	1	1	0	1	1	4
mmu-miR-223-3p	59040	Rhot1	1	0	1	1	1	4
mmu-miR-223-3p	59050	Nsa2	1	1	0	1	1	4
mmu-miR-223-3p	60315	Myg1	1	0	1	1	1	4
mmu-miR-223-3p	60344	Fign	1	0	1	1	1	4
mmu-miR-223-3p	63828	Fn3k	1	0	1	1	1	4
mmu-miR-223-3p	63955	Cables1	1	0	1	1	1	4
mmu-miR-223-3p	63959	Slc29a1	1	0	1	1	1	4
mmu-miR-223-3p	64075	Smoc1	1	0	1	1	1	4
mmu-miR-223-3p	64213	St7	1	1	0	1	1	4
mmu-miR-223-3p	64297	Gprc5b	1	1	1	1	0	4
mmu-miR-223-3p	64339	Fndc4	1	0	1	1	1	4
mmu-miR-223-3p	64602	Ireb2	1	1	0	1	1	4
mmu-miR-223-3p	65086	Lpar3	1	1	1	1	0	4

mmu-miR-223-3p	65115	Bean1	1	1	0	1	1	4
mmu-miR-223-3p	65247	Asb1	1	1	0	1	1	4
mmu-miR-223-3p	65254	Dpysl5	1	0	1	1	1	4
mmu-miR-223-3p	65967	Eefsec	1	1	1	0	1	4
mmu-miR-223-3p	66054	Cndp2	1	0	1	1	1	4
mmu-miR-223-3p	66112	Marc1	1	0	1	1	1	4
mmu-miR-223-3p	66313	Smurf2	1	1	0	1	1	4
mmu-miR-223-3p	66388	Cutc	1	1	0	1	1	4
mmu-miR-223-3p	66419	Mrpl11	1	1	0	1	1	4
mmu-miR-223-3p	66514	Asrgl1	1	1	1	1	0	4
mmu-miR-223-3p	66552	Sppl2a	1	1	0	1	1	4
mmu-miR-223-3p	66570	Cenpm	1	1	0	1	1	4
mmu-miR-223-3p	66634	Mcm8	1	1	1	1	0	4
mmu-miR-223-3p	66663	Uba5	1	1	0	1	1	4
mmu-miR-223-3p	66667	Hspbap1	1	0	1	1	1	4
mmu-miR-223-3p	66684	Tceal8	1	0	1	1	1	4
mmu-miR-223-3p	66711	Sbds	1	0	1	1	1	4
mmu-miR-223-3p	66811	Duoxa2	1	0	1	1	1	4
mmu-miR-223-3p	66817	Tmem170	1	1	1	0	1	4
mmu-miR-223-3p	66849	Ppp1r2	1	0	1	1	1	4
mmu-miR-223-3p	66855	Tcf25	1	0	1	1	1	4
mmu-miR-223-3p	66860	Tanc1	1	1	1	1	0	4
mmu-miR-223-3p	66873	Tril	1	0	1	1	1	4
mmu-miR-223-3p	66899	Fip1l1	1	0	1	1	1	4
mmu-miR-223-3p	66959	Dusp26	1	0	1	1	1	4
mmu-miR-223-3p	66964	Golt1b	1	0	1	1	1	4
mmu-miR-223-3p	66979	Pole4	1	1	1	0	1	4
mmu-miR-223-3p	67040	Ddx17	1	1	0	1	1	4
mmu-miR-223-3p	67059	Ola1	1	1	1	1	0	4
mmu-miR-223-3p	67062	Slc25a53	1	1	0	1	1	4
mmu-miR-223-3p	67074	Mon2	1	1	0	1	1	4
mmu-miR-223-3p	67087	Ctnnbip1	1	0	1	1	1	4
mmu-miR-223-3p	67102	D16Ertd472e	1	0	1	1	1	4
mmu-miR-223-3p	67161	Sclt1	1	1	0	1	1	4
mmu-miR-223-3p	67163	Ccdc47	1	1	1	1	0	4
mmu-miR-223-3p	67229	Prpf18	1	0	1	1	1	4
mmu-miR-223-3p	67254	2900011O08Rik	1	0	1	1	1	4
mmu-miR-223-3p	67288	Srek1ip1	1	1	1	1	0	4
mmu-miR-223-3p	67295	Rab3c	1	1	0	1	1	4
mmu-miR-223-3p	67306	Zc2hc1a	1	0	1	1	1	4
mmu-miR-223-3p	67338	Rffl	1	0	1	1	1	4
mmu-miR-223-3p	67344	Tctex1d1	1	1	0	1	1	4
mmu-miR-223-3p	67370	Zfp606	1	0	1	1	1	4
mmu-miR-223-3p	67382	Brd3	1	1	0	1	1	4
mmu-miR-223-3p	67392	4833420G17Rik	1	1	0	1	1	4
mmu-miR-223-3p	67418	Ppil4	1	1	1	1	0	4
mmu-miR-223-3p	67451	Pkp2	1	0	1	1	1	4
mmu-miR-223-3p	67457	Frmd8	1	1	1	0	1	4
mmu-miR-223-3p	67459	Nvl	1	0	1	1	1	4
mmu-miR-223-3p	67466	Pdcl	1	1	1	0	1	4
mmu-miR-223-3p	67495	Tmem167b	1	0	1	1	1	4
mmu-miR-223-3p	67547	Slc39a8	1	0	1	1	1	4
mmu-miR-223-3p	67573	Loxl4	1	0	1	1	1	4
mmu-miR-223-3p	67588	Rnf41	1	0	1	1	1	4
mmu-miR-223-3p	67731	Fbxo32	1	1	0	1	1	4

mmu-miR-223-3p	67741	4930579F01Rik	1	1	0	1	1	4
mmu-miR-223-3p	67771	Arpc5	1	1	1	1	0	4
mmu-miR-223-3p	67774	Loh12cr1	1	0	1	1	1	4
mmu-miR-223-3p	67784	Plxnd1	1	0	1	1	1	4
mmu-miR-223-3p	67832	Brix1	1	0	1	1	1	4
mmu-miR-223-3p	67873	Mri1	1	1	1	1	0	4
mmu-miR-223-3p	67943	Mesdc2	1	0	1	1	1	4
mmu-miR-223-3p	67963	Npc2	1	0	1	1	1	4
mmu-miR-223-3p	68010	Bambi	1	1	1	1	0	4
mmu-miR-223-3p	68031	Rnf146	1	1	0	1	1	4
mmu-miR-223-3p	68036	Zfp706	1	0	1	1	1	4
mmu-miR-223-3p	68054	Serpina12	1	0	1	1	1	4
mmu-miR-223-3p	68107	Cntd1	1	0	1	1	1	4
mmu-miR-223-3p	68166	Spire1	1	1	0	1	1	4
mmu-miR-223-3p	68366	Tmem129	1	1	1	0	1	4
mmu-miR-223-3p	68428	Steap3	1	1	0	1	1	4
mmu-miR-223-3p	68473	Mob1b	1	0	1	1	1	4
mmu-miR-223-3p	68477	Rmnd5a	1	1	0	1	1	4
mmu-miR-223-3p	68512	Tomm5	1	0	1	1	1	4
mmu-miR-223-3p	68526	Gpr155	1	1	0	1	1	4
mmu-miR-223-3p	68564	Nufip2	1	0	1	1	1	4
mmu-miR-223-3p	68675	Fam172a	1	1	0	1	1	4
mmu-miR-223-3p	68867	Rnf122	1	0	1	1	1	4
mmu-miR-223-3p	68877	Maf1	1	0	1	1	1	4
mmu-miR-223-3p	68904	Abhd13	1	0	1	1	1	4
mmu-miR-223-3p	68968	Cdan1	1	1	1	1	0	4
mmu-miR-223-3p	69035	Zdhhc3	1	0	1	1	1	4
mmu-miR-223-3p	69091	Vps26b	1	1	1	1	0	4
mmu-miR-223-3p	69276	Sec62	1	0	1	1	1	4
mmu-miR-223-3p	69332	Lelp1	1	1	0	1	1	4
mmu-miR-223-3p	69367	Glrx2	1	1	0	1	1	4
mmu-miR-223-3p	69368	Wdfy1	1	1	1	1	0	4
mmu-miR-223-3p	69397	1700019A02Rik	1	1	0	1	1	4
mmu-miR-223-3p	69470	Tmem127	1	1	1	1	0	4
mmu-miR-223-3p	69581	Rhou	1	0	1	1	1	4
mmu-miR-223-3p	69601	Dab2ip	1	1	0	1	1	4
mmu-miR-223-3p	69663	Ddx51	1	0	1	1	1	4
mmu-miR-223-3p	69709	Ptrhd1	1	1	0	1	1	4
mmu-miR-223-3p	69718	Ipmk	1	0	1	1	1	4
mmu-miR-223-3p	69786	Tprkb	1	0	1	1	1	4
mmu-miR-223-3p	69802	Cox11	1	0	1	1	1	4
mmu-miR-223-3p	69807	Trim32	1	1	1	1	0	4
mmu-miR-223-3p	69860	Eif1ad	1	1	1	0	1	4
mmu-miR-223-3p	69888	Cyp2c66	1	1	1	1	0	4
mmu-miR-223-3p	69993	Chn2	1	0	1	1	1	4
mmu-miR-223-3p	70008	Ace2	1	0	1	1	1	4
mmu-miR-223-3p	70118	Srrd	1	1	0	1	1	4
mmu-miR-223-3p	70127	Dpf3	1	1	0	1	1	4
mmu-miR-223-3p	70292	Afap1	1	1	1	1	0	4
mmu-miR-223-3p	70357	Kcnip1	1	0	1	1	1	4
mmu-miR-223-3p	70465	Wdr77	1	0	1	1	1	4
mmu-miR-223-3p	70546	Zdhhc2	1	1	1	1	0	4
mmu-miR-223-3p	70568	Cpne3	1	0	1	1	1	4
mmu-miR-223-3p	70620	Ube2v2	1	0	1	1	1	4
mmu-miR-223-3p	70638	Fam189a1	1	0	1	1	1	4

mmu-miR-223-3p	70640	Dcp2	1	1	0	1	1	4
mmu-miR-223-3p	70757	Ptplb	1	0	1	1	1	4
mmu-miR-223-3p	70804	Pgrmc2	1	1	0	1	1	4
mmu-miR-223-3p	70827	Trak2	1	0	1	1	1	4
mmu-miR-223-3p	70834	Spag9	1	1	0	1	1	4
mmu-miR-223-3p	70891	Spdya	1	1	0	1	1	4
mmu-miR-223-3p	71148	Mier1	1	1	0	1	1	4
mmu-miR-223-3p	71306	Mfap3l	1	0	1	1	1	4
mmu-miR-223-3p	71313	Fsip1	1	0	1	1	1	4
mmu-miR-223-3p	71398	5430427O19Rik	1	1	0	1	1	4
mmu-miR-223-3p	71452	Ankrd40	1	0	1	1	1	4
mmu-miR-223-3p	71529	Kazn	1	1	1	1	0	4
mmu-miR-223-3p	71544	Arhgap42	1	1	0	1	1	4
mmu-miR-223-3p	71586	Ifih1	1	1	0	1	1	4
mmu-miR-223-3p	71591	Zfp251	1	1	1	0	1	4
mmu-miR-223-3p	71592	Pogk	1	0	1	1	1	4
mmu-miR-223-3p	71599	Senp8	1	0	1	1	1	4
mmu-miR-223-3p	71678	Brox	1	1	0	1	1	4
mmu-miR-223-3p	71684	Rbm43	1	0	1	1	1	4
mmu-miR-223-3p	71704	Arhgef3	1	0	1	1	1	4
mmu-miR-223-3p	71721	Fam13c	1	0	1	1	1	4
mmu-miR-223-3p	71743	Coasy	1	1	1	1	0	4
mmu-miR-223-3p	71751	Map3k13	1	0	1	1	1	4
mmu-miR-223-3p	71752	Gtf3c2	1	1	1	0	1	4
mmu-miR-223-3p	71778	Klhl5	1	1	1	0	1	4
mmu-miR-223-3p	71791	Cpa4	1	1	1	1	0	4
mmu-miR-223-3p	71816	Rnf180	1	0	1	1	1	4
mmu-miR-223-3p	71939	Apol6	1	1	0	1	1	4
mmu-miR-223-3p	72003	Synpr	1	0	1	1	1	4
mmu-miR-223-3p	72068	Cnot2	1	1	0	1	1	4
mmu-miR-223-3p	72077	Gcnt3	1	1	1	1	0	4
mmu-miR-223-3p	72123	Ccdc71l	1	1	0	1	1	4
mmu-miR-223-3p	72133	Trub1	1	0	1	1	1	4
mmu-miR-223-3p	72155	Cenpn	1	1	1	0	1	4
mmu-miR-223-3p	72193	Scaf11	1	0	1	1	1	4
mmu-miR-223-3p	72198	Skiv2l2	1	0	1	1	1	4
mmu-miR-223-3p	72301	1810041L15Rik	1	1	0	1	1	4
mmu-miR-223-3p	72318	Cyth4	1	1	1	1	0	4
mmu-miR-223-3p	72344	Usp36	1	1	0	1	1	4
mmu-miR-223-3p	72354	Ttc4	1	0	1	1	1	4
mmu-miR-223-3p	72393	Faim2	1	1	0	1	1	4
mmu-miR-223-3p	72446	Prr5l	1	1	0	1	1	4
mmu-miR-223-3p	72504	Taf4b	1	1	0	1	1	4
mmu-miR-223-3p	72515	Wdr43	1	1	0	1	1	4
mmu-miR-223-3p	72542	Pgam5	1	0	1	1	1	4
mmu-miR-223-3p	72568	Lin9	1	0	1	1	1	4
mmu-miR-223-3p	72667	Zfp444	1	0	1	1	1	4
mmu-miR-223-3p	72685	Dnajc6	1	0	1	1	1	4
mmu-miR-223-3p	72750	Fam117b	1	1	0	1	1	4
mmu-miR-223-3p	72789	Veph1	1	1	0	1	1	4
mmu-miR-223-3p	72805	Zfp839	1	1	0	1	1	4
mmu-miR-223-3p	72925	March1	1	0	1	1	1	4
mmu-miR-223-3p	72949	Ccnt2	1	1	1	0	1	4
mmu-miR-223-3p	72978	Cnih3	1	1	0	1	1	4
mmu-miR-223-3p	72993	Appl1	1	0	1	1	1	4

mmu-miR-223-3p	73181	Nfatc4	1	1	0	1	1	4
mmu-miR-223-3p	73230	Bmper	1	1	1	0	1	4
mmu-miR-223-3p	73246	Rassf6	1	0	1	1	1	4
mmu-miR-223-3p	73385	Fam177a	1	1	0	1	1	4
mmu-miR-223-3p	73419	1700052N19Rik	1	1	1	1	0	4
mmu-miR-223-3p	73473	Iws1	1	1	1	1	0	4
mmu-miR-223-3p	73713	Rbm20	1	1	0	1	1	4
mmu-miR-223-3p	73884	Zdbf2	1	1	0	1	1	4
mmu-miR-223-3p	73945	Otud4	1	0	1	1	1	4
mmu-miR-223-3p	74008	Arsg	1	0	1	1	1	4
mmu-miR-223-3p	74042	4921501E09Rik	1	1	0	1	1	4
mmu-miR-223-3p	74051	Steap2	1	1	0	1	1	4
mmu-miR-223-3p	74107	Cep55	1	0	1	1	1	4
mmu-miR-223-3p	74112	Usp16	1	1	1	1	0	4
mmu-miR-223-3p	74156	Acot12	1	0	1	1	1	4
mmu-miR-223-3p	74180	Muc5b	1	1	1	0	1	4
mmu-miR-223-3p	74205	Acsl3	1	1	0	1	1	4
mmu-miR-223-3p	74315	Rnf145	1	0	1	1	1	4
mmu-miR-223-3p	74330	Dnajc14	1	1	1	1	0	4
mmu-miR-223-3p	74360	Cep57	1	1	1	1	0	4
mmu-miR-223-3p	74386	Rmi1	1	0	1	1	1	4
mmu-miR-223-3p	74451	Pgs1	1	1	1	0	1	4
mmu-miR-223-3p	74470	Cep72	1	1	0	1	1	4
mmu-miR-223-3p	74559	Elovl7	1	1	0	1	1	4
mmu-miR-223-3p	74570	Zkscan1	1	1	0	1	1	4
mmu-miR-223-3p	74591	Abca12	1	1	0	1	1	4
mmu-miR-223-3p	74637	Shpk	1	0	1	1	1	4
mmu-miR-223-3p	74711	Ttll9	1	0	1	1	1	4
mmu-miR-223-3p	74734	Rhoh	1	0	1	1	1	4
mmu-miR-223-3p	74762	Mdga1	1	0	1	1	1	4
mmu-miR-223-3p	75210	Prr3	1	0	1	1	1	4
mmu-miR-223-3p	75296	Fgfr1op	1	1	0	1	1	4
mmu-miR-223-3p	75404	Arhgap36	0	1	1	1	1	4
mmu-miR-223-3p	75424	Zfp820	1	1	1	0	1	4
mmu-miR-223-3p	75530	Lyrm7	1	0	1	1	1	4
mmu-miR-223-3p	75599	Pcdh1	1	1	1	1	0	4
mmu-miR-223-3p	75624	Metap1	1	0	1	1	1	4
mmu-miR-223-3p	75914	Exoc6b	1	1	0	1	1	4
mmu-miR-223-3p	75974	Dock11	1	1	1	1	0	4
mmu-miR-223-3p	76132	Faxc	1	0	1	1	1	4
mmu-miR-223-3p	76179	Usp31	1	1	0	1	1	4
mmu-miR-223-3p	76224	6530409C15Rik	1	1	0	1	1	4
mmu-miR-223-3p	76246	Rtf1	1	1	0	1	1	4
mmu-miR-223-3p	76376	Slc24a2	1	1	0	1	1	4
mmu-miR-223-3p	76498	Paqr4	1	1	1	1	0	4
mmu-miR-223-3p	76499	Clasp2	1	1	0	1	1	4
mmu-miR-223-3p	76559	Atg2b	0	1	1	1	1	4
mmu-miR-223-3p	76688	Arfrp1	1	0	1	1	1	4
mmu-miR-223-3p	76742	Snx27	1	0	1	1	1	4
mmu-miR-223-3p	76768	Alpi	1	0	1	1	1	4
mmu-miR-223-3p	76792	2410131K14Rik	1	0	1	1	1	4
mmu-miR-223-3p	76854	Gper1	1	1	1	0	1	4
mmu-miR-223-3p	76895	Bicd2	1	1	1	1	0	4
mmu-miR-223-3p	76899	Golga1	1	1	1	0	1	4
mmu-miR-223-3p	76980	Ube2ql1	1	1	0	1	1	4

mmu-miR-223-3p	77015	Mpped2	1	1	0	1	1	4
mmu-miR-223-3p	77018	Col25a1	1	1	0	1	1	4
mmu-miR-223-3p	77040	Atg16l1	1	0	1	1	1	4
mmu-miR-223-3p	77044	Arid2	1	0	1	1	1	4
mmu-miR-223-3p	77128	Crebrf	1	1	0	1	1	4
mmu-miR-223-3p	77300	Raph1	1	1	0	1	1	4
mmu-miR-223-3p	77531	Anks1b	1	1	0	1	1	4
mmu-miR-223-3p	77766	Elp4	1	1	1	1	0	4
mmu-miR-223-3p	77889	Lbh	1	1	1	1	0	4
mmu-miR-223-3p	77929	Yipf6	1	0	1	1	1	4
mmu-miR-223-3p	77976	Nuak1	1	1	1	1	0	4
mmu-miR-223-3p	78244	Dnajc21	1	1	0	1	1	4
mmu-miR-223-3p	78248	Armcx1	1	0	1	1	1	4
mmu-miR-223-3p	78255	Ralgps2	1	0	1	1	1	4
mmu-miR-223-3p	78334	Cdk19	1	0	1	1	1	4
mmu-miR-223-3p	78593	Nrip3	1	0	1	1	1	4
mmu-miR-223-3p	78618	Acap2	1	0	1	1	1	4
mmu-miR-223-3p	78825	Desi2	1	1	1	1	0	4
mmu-miR-223-3p	78832	Cacul1	1	1	0	1	1	4
mmu-miR-223-3p	78910	Asb15	1	1	0	1	1	4
mmu-miR-223-3p	78912	Sp2	1	1	0	1	1	4
mmu-miR-223-3p	78935	Saal1	1	1	0	1	1	4
mmu-miR-223-3p	80285	Parp9	1	0	1	1	1	4
mmu-miR-223-3p	80288	Bcl9l	1	1	1	1	0	4
mmu-miR-223-3p	80718	Rab27b	1	1	0	1	1	4
mmu-miR-223-3p	80719	Igsf6	1	1	1	0	1	4
mmu-miR-223-3p	80751	Rnf34	1	1	1	0	1	4
mmu-miR-223-3p	80837	Rhoj	1	0	1	1	1	4
mmu-miR-223-3p	80903	Fgf16	1	1	1	1	0	4
mmu-miR-223-3p	80909	Gatsl2	1	1	1	1	0	4
mmu-miR-223-3p	81702	Ankrd17	1	1	0	1	1	4
mmu-miR-223-3p	83429	Ctns	1	0	1	1	1	4
mmu-miR-223-3p	83436	Plekha2	1	0	1	1	1	4
mmu-miR-223-3p	83453	Chrdl1	0	1	1	1	1	4
mmu-miR-223-3p	83672	Sytl3	1	1	0	1	1	4
mmu-miR-223-3p	93690	Gpr45	1	1	1	1	0	4
mmu-miR-223-3p	93737	Pard6g	1	1	1	0	1	4
mmu-miR-223-3p	93765	Ube2n	1	1	1	1	0	4
mmu-miR-223-3p	93790	Nipa2	1	0	1	1	1	4
mmu-miR-223-3p	93871	Brwd1	1	0	1	1	1	4
mmu-miR-223-3p	93882	Pcdhb11	1	0	1	1	1	4
mmu-miR-223-3p	93896	Glp2r	1	0	1	1	1	4
mmu-miR-223-3p	93960	Nkd1	1	1	0	1	1	4
mmu-miR-223-3p	94045	P2rx5	1	1	1	0	1	4
mmu-miR-223-3p	94088	Trim6	1	1	1	1	0	4
mmu-miR-223-3p	94212	Pag1	1	1	0	1	1	4
mmu-miR-223-3p	98396	Slc41a1	1	1	1	1	0	4
mmu-miR-223-3p	98732	Rab3gap2	1	1	0	1	1	4
mmu-miR-223-3p	98766	Ubac1	1	1	1	1	0	4
mmu-miR-223-3p	98878	Ehd4	1	1	1	1	0	4
mmu-miR-223-3p	98910	Usp6nl	1	1	0	1	1	4
mmu-miR-223-3p	98970	Fibcd1	1	1	1	1	0	4
mmu-miR-223-3p	99138	Stard7	1	1	1	1	0	4
mmu-miR-223-3p	100169	Phactr4	0	1	1	1	1	4
mmu-miR-223-3p	100465	Mob3c	1	0	1	1	1	4

mmu-miR-223-3p	100647	Upk3b	1	0	1	1	1	4
mmu-miR-223-3p	100855	Tbc1d14	1	1	0	1	1	4
mmu-miR-223-3p	101358	Fbxl14	1	0	1	1	1	4
mmu-miR-223-3p	102193	Zdhhc7	1	1	1	1	0	4
mmu-miR-223-3p	102294	Cyp4v3	1	0	1	1	1	4
mmu-miR-223-3p	102502	Pls1	1	1	0	1	1	4
mmu-miR-223-3p	102595	Plekho2	1	0	1	1	1	4
mmu-miR-223-3p	103537	Mbtd1	1	1	1	1	0	4
mmu-miR-223-3p	103677	Smg6	1	1	1	1	0	4
mmu-miR-223-3p	103712	6330403K07Rik	1	0	1	1	1	4
mmu-miR-223-3p	103784	Wdr92	1	0	1	1	1	4
mmu-miR-223-3p	103841	Cuedc1	1	1	0	1	1	4
mmu-miR-223-3p	104215	Rhoq	1	0	1	1	1	4
mmu-miR-223-3p	104401	Pcnxl3	1	1	1	1	0	4
mmu-miR-223-3p	104681	Slc16a6	1	1	0	1	1	4
mmu-miR-223-3p	104709	Pik3r6	1	1	0	1	1	4
mmu-miR-223-3p	104859	Tecpr2	1	1	1	1	0	4
mmu-miR-223-3p	105000	Dnal1	1	0	1	1	1	4
mmu-miR-223-3p	105348	Golm1	1	1	0	1	1	4
mmu-miR-223-3p	105727	Slc38a1	1	0	1	1	1	4
mmu-miR-223-3p	105734	Tigd5	1	0	1	1	1	4
mmu-miR-223-3p	105787	Prkaa1	1	1	1	1	0	4
mmu-miR-223-3p	105853	Mal2	1	0	1	1	1	4
mmu-miR-223-3p	106068	Slc45a4	1	1	0	1	1	4
mmu-miR-223-3p	106583	Scaf8	1	1	0	1	1	4
mmu-miR-223-3p	106585	Ankrd12	1	1	1	1	0	4
mmu-miR-223-3p	106869	Tnfaip8	1	0	1	1	1	4
mmu-miR-223-3p	106947	Slc39a3	1	0	1	1	1	4
mmu-miR-223-3p	107182	Btaf1	1	0	1	1	1	4
mmu-miR-223-3p	107242	AI837181	1	1	0	1	1	4
mmu-miR-223-3p	107568	Wwp1	1	1	1	1	0	4
mmu-miR-223-3p	108037	Shmt2	1	0	1	1	1	4
mmu-miR-223-3p	108083	Pip4k2b	1	1	1	1	0	4
mmu-miR-223-3p	108105	B3gnt5	1	0	1	1	1	4
mmu-miR-223-3p	108116	Slco3a1	1	0	1	1	1	4
mmu-miR-223-3p	108155	Ogt	1	1	1	1	0	4
mmu-miR-223-3p	108655	Foxp1	1	1	0	1	1	4
mmu-miR-223-3p	108657	Rnpepl1	1	1	1	0	1	4
mmu-miR-223-3p	108673	Ccdc86	1	1	1	0	1	4
mmu-miR-223-3p	108705	Pttg1ip	1	1	1	0	1	4
mmu-miR-223-3p	108737	Oxsr1	1	1	0	1	1	4
mmu-miR-223-3p	108829	Jmjd1c	1	1	0	1	1	4
mmu-miR-223-3p	108934	Smim13	1	1	0	1	1	4
mmu-miR-223-3p	108937	Rnf169	1	1	0	1	1	4
mmu-miR-223-3p	108946	Zzz3	1	1	0	1	1	4
mmu-miR-223-3p	109032	Sp110	1	0	1	1	1	4
mmu-miR-223-3p	109154	Mlec	1	1	1	1	0	4
mmu-miR-223-3p	109161	Ube2q2	1	1	1	0	1	4
mmu-miR-223-3p	109168	Atl3	1	0	1	1	1	4
mmu-miR-223-3p	109676	Ank2	1	1	0	1	1	4
mmu-miR-223-3p	109785	Pgm3	1	1	1	1	0	4
mmu-miR-223-3p	109880	Braf	1	0	1	1	1	4
mmu-miR-223-3p	110417	Pigh	1	0	1	1	1	4
mmu-miR-223-3p	110593	Prdm2	1	0	1	1	1	4
mmu-miR-223-3p	110695	Aldh7a1	1	0	1	1	1	4

mmu-miR-223-3p	110835	Chrna5	1	1	0	1	1	4
mmu-miR-223-3p	110893	Slc8a3	1	1	0	1	1	4
mmu-miR-223-3p	110920	Hspa13	1	0	1	1	1	4
mmu-miR-223-3p	114249	Npnt	1	1	0	1	1	4
mmu-miR-223-3p	114565	Zbtb21	1	1	0	1	1	4
mmu-miR-223-3p	114643	Oas1c	1	1	1	1	0	4
mmu-miR-223-3p	114661	Prss28	1	0	1	1	1	4
mmu-miR-223-3p	114715	Spred1	1	1	0	1	1	4
mmu-miR-223-3p	114893	Dcun1d1	1	0	1	1	1	4
mmu-miR-223-3p	116891	Derl2	1	1	1	1	0	4
mmu-miR-223-3p	116939	Pnpla3	1	0	1	1	1	4
mmu-miR-223-3p	117146	Ube3b	1	0	1	1	1	4
mmu-miR-223-3p	117600	Srgap1	1	1	0	1	1	4
mmu-miR-223-3p	118452	Baalc	1	0	1	1	1	4
mmu-miR-223-3p	140484	Pofut1	1	1	0	1	1	4
mmu-miR-223-3p	140491	Ppp1r3a	1	0	1	1	1	4
mmu-miR-223-3p	140493	Kcnn3	1	0	1	1	1	4
mmu-miR-223-3p	140580	Elmo1	1	1	0	1	1	4
mmu-miR-223-3p	140858	Wdr5	1	1	1	0	1	4
mmu-miR-223-3p	142980	Tlr3	1	0	1	1	1	4
mmu-miR-223-3p	170458	Gpha2	1	0	1	1	1	4
mmu-miR-223-3p	170755	Sgk3	1	1	0	1	1	4
mmu-miR-223-3p	171167	Fut10	1	0	1	1	1	4
mmu-miR-223-3p	171233	Vmn1r235	1	0	1	1	1	4
mmu-miR-223-3p	171281	Acot3	1	0	1	1	1	4
mmu-miR-223-3p	171285	Havcr2	1	0	1	1	1	4
mmu-miR-223-3p	171486	Cd99l2	1	0	1	1	1	4
mmu-miR-223-3p	171531	Mlph	1	1	1	1	0	4
mmu-miR-223-3p	192161	Pcdha9	1	1	0	1	1	4
mmu-miR-223-3p	192164	Pcdha12	1	1	0	1	1	4
mmu-miR-223-3p	192216	Tmem47	1	1	0	1	1	4
mmu-miR-223-3p	192236	Hps1	1	0	1	1	1	4
mmu-miR-223-3p	192662	Arhgdia	1	1	1	1	0	4
mmu-miR-223-3p	193385	Fam65b	1	0	1	1	1	4
mmu-miR-223-3p	193740	Hspa1a	1	0	1	1	1	4
mmu-miR-223-3p	194237	Rimkla	1	0	1	1	1	4
mmu-miR-223-3p	194388	Tet3	1	1	0	1	1	4
mmu-miR-223-3p	194908	Pld6	1	0	1	1	1	4
mmu-miR-223-3p	207181	Rbms3	1	1	0	1	1	4
mmu-miR-223-3p	207214	Larp4	1	1	0	1	1	4
mmu-miR-223-3p	207393	Elfn2	1	1	1	0	1	4
mmu-miR-223-3p	207615	Wdr37	1	0	1	1	1	4
mmu-miR-223-3p	207921	Fam228b	1	1	0	1	1	4
mmu-miR-223-3p	207958	Alg11	1	1	0	1	1	4
mmu-miR-223-3p	208092	Chmp6	1	0	1	1	1	4
mmu-miR-223-3p	208292	Zfp871	1	0	1	1	1	4
mmu-miR-223-3p	208650	Cblb	1	1	1	1	0	4
mmu-miR-223-3p	208677	Creb3l3	1	1	1	0	1	4
mmu-miR-223-3p	208748	Prrg3	1	0	1	1	1	4
mmu-miR-223-3p	208846	Daam1	1	1	0	1	1	4
mmu-miR-223-3p	208982	Hmgcll1	1	0	1	1	1	4
mmu-miR-223-3p	209131	Snx30	1	1	1	1	0	4
mmu-miR-223-3p	209176	Ido2	1	1	1	0	1	4
mmu-miR-223-3p	209387	Trim30d	1	1	0	1	1	4
mmu-miR-223-3p	209630	Frmd4a	1	0	1	1	1	4

mmu-miR-223-3p	209683	Ttc28	1	1	0	1	1	4
mmu-miR-223-3p	209773	Dennd2a	1	1	1	0	1	4
mmu-miR-223-3p	210126	Lpp	1	1	0	1	1	4
mmu-miR-223-3p	210162	Zkscan2	1	0	1	1	1	4
mmu-miR-223-3p	210172	Zfp526	1	0	1	1	1	4
mmu-miR-223-3p	211586	Tfdp2	1	0	1	1	1	4
mmu-miR-223-3p	211612	Ptchd1	1	0	1	1	1	4
mmu-miR-223-3p	211712	Pcdh9	1	0	1	1	1	4
mmu-miR-223-3p	211739	Vstm2a	1	1	1	1	0	4
mmu-miR-223-3p	211798	Mfsd9	1	1	1	0	1	4
mmu-miR-223-3p	211922	Dennd6a	1	1	0	1	1	4
mmu-miR-223-3p	211945	Plekhh1	1	1	0	1	1	4
mmu-miR-223-3p	212276	Zfp748	1	0	1	1	1	4
mmu-miR-223-3p	212391	Lcor	1	1	1	1	0	4
mmu-miR-223-3p	212647	Aldh4a1	1	1	1	0	1	4
mmu-miR-223-3p	212943	Fam46a	1	1	0	1	1	4
mmu-miR-223-3p	213006	Mfsd4	1	0	1	1	1	4
mmu-miR-223-3p	213056	Fam126b	1	1	1	1	0	4
mmu-miR-223-3p	213350	Pddc1	1	0	1	1	1	4
mmu-miR-223-3p	213449	Fam46d	1	1	0	1	1	4
mmu-miR-223-3p	213452	Dstyk	1	0	1	1	1	4
mmu-miR-223-3p	213988	Tnrc6b	1	1	0	1	1	4
mmu-miR-223-3p	214058	Megf11	1	0	1	1	1	4
mmu-miR-223-3p	214112	Nipal4	1	0	1	1	1	4
mmu-miR-223-3p	214150	Ago3	1	1	0	1	1	4
mmu-miR-223-3p	214459	Fnbp1l	1	1	0	1	1	4
mmu-miR-223-3p	214469	Fam168b	1	0	1	1	1	4
mmu-miR-223-3p	214523	Tmprss4	1	1	1	1	0	4
mmu-miR-223-3p	214547	She	1	0	1	1	1	4
mmu-miR-223-3p	214575	Tdrd5	1	1	0	1	1	4
mmu-miR-223-3p	214627	Papd5	1	1	0	1	1	4
mmu-miR-223-3p	214944	Mob3b	1	0	1	1	1	4
mmu-miR-223-3p	214952	Rhot2	1	0	1	1	1	4
mmu-miR-223-3p	214968	Sema6d	1	0	1	1	1	4
mmu-miR-223-3p	215008	Vezt	1	1	1	1	0	4
mmu-miR-223-3p	215436	Slc35e3	1	0	1	1	1	4
mmu-miR-223-3p	215474	Sec22c	1	0	1	1	1	4
mmu-miR-223-3p	215708	Fam73a	1	1	1	1	0	4
mmu-miR-223-3p	215789	Phactr2	1	0	1	1	1	4
mmu-miR-223-3p	216049	Zfp365	1	1	1	1	0	4
mmu-miR-223-3p	216233	Socs2	1	1	1	1	0	4
mmu-miR-223-3p	216725	Adamts2	1	1	0	1	1	4
mmu-miR-223-3p	216766	Gemin5	1	0	1	1	1	4
mmu-miR-223-3p	216810	Tom1l2	1	1	0	1	1	4
mmu-miR-223-3p	216963	Git1	1	0	1	1	1	4
mmu-miR-223-3p	216965	Taok1	1	1	1	1	0	4
mmu-miR-223-3p	217026	Heatr6	1	1	1	1	0	4
mmu-miR-223-3p	217143	Gpr179	1	1	1	1	0	4
mmu-miR-223-3p	217344	Rhbdf2	1	0	1	1	1	4
mmu-miR-223-3p	217371	Rab40b	1	0	1	1	1	4
mmu-miR-223-3p	217826	Kcnk13	1	0	1	1	1	4
mmu-miR-223-3p	217830	9030617O03Rik	1	1	1	1	0	4
mmu-miR-223-3p	217869	Eif5	1	1	0	1	1	4
mmu-miR-223-3p	217980	Larp4b	1	0	1	1	1	4
mmu-miR-223-3p	218194	Phactr1	1	1	0	1	1	4

mmu-miR-223-3p	218215	Rnf144b	1	0	1	1	1	4
mmu-miR-223-3p	218294	Cdc14b	1	1	0	1	1	4
mmu-miR-223-3p	218333	BC018507	1	1	0	1	1	4
mmu-miR-223-3p	218460	Wdr41	1	1	1	0	1	4
mmu-miR-223-3p	218476	Gcnt4	1	1	0	1	1	4
mmu-miR-223-3p	218613	Mier3	1	1	1	1	0	4
mmu-miR-223-3p	218772	Rarb	1	0	1	1	1	4
mmu-miR-223-3p	219140	Spata13	1	1	0	1	1	4
mmu-miR-223-3p	219148	Fam167a	1	0	1	1	1	4
mmu-miR-223-3p	219181	Akap11	1	1	0	1	1	4
mmu-miR-223-3p	219228	Pcdh17	1	0	1	1	1	4
mmu-miR-223-3p	223254	Farp1	1	0	1	1	1	4
mmu-miR-223-3p	223601	Fam49b	1	0	1	1	1	4
mmu-miR-223-3p	223739	5031439G07Rik	1	0	1	1	1	4
mmu-miR-223-3p	223773	Zbed4	1	1	1	1	0	4
mmu-miR-223-3p	223838	Adamts20	1	1	0	1	1	4
mmu-miR-223-3p	223922	Atf7	1	0	1	1	1	4
mmu-miR-223-3p	224105	Pak2	1	1	1	1	0	4
mmu-miR-223-3p	224224	Impg2	1	1	1	1	0	4
mmu-miR-223-3p	224648	Uhrf1bp1	1	0	1	1	1	4
mmu-miR-223-3p	224836	Usp49	1	0	1	1	1	4
mmu-miR-223-3p	224840	Treml4	1	0	1	1	1	4
mmu-miR-223-3p	224997	Dlgap1	1	1	0	1	1	4
mmu-miR-223-3p	225215	Rsl24d1	1	0	1	1	1	4
mmu-miR-223-3p	225432	Rbm27	1	1	0	1	1	4
mmu-miR-223-3p	225861	Snx32	1	0	1	1	1	4
mmu-miR-223-3p	225998	Rorb	1	1	0	1	1	4
mmu-miR-223-3p	226026	Smc5	1	1	0	1	1	4
mmu-miR-223-3p	226041	Pgm5	1	0	1	1	1	4
mmu-miR-223-3p	226098	Hectd2	1	0	1	1	1	4
mmu-miR-223-3p	226178	Wbp1l	1	0	1	1	1	4
mmu-miR-223-3p	226304	Npbwr1	1	1	0	1	1	4
mmu-miR-223-3p	226352	Epb4.1l5	1	0	1	1	1	4
mmu-miR-223-3p	226517	Smg7	1	0	1	1	1	4
mmu-miR-223-3p	226525	Rasal2	1	1	0	1	1	4
mmu-miR-223-3p	226610	Fam78b	1	0	1	1	1	4
mmu-miR-223-3p	226641	Atf6	1	0	1	1	1	4
mmu-miR-223-3p	226823	Kctd3	1	0	1	1	1	4
mmu-miR-223-3p	226999	Slc9a2	1	1	1	0	1	4
mmu-miR-223-3p	227120	Plcl1	1	0	1	1	1	4
mmu-miR-223-3p	227195	Ino80d	1	1	0	1	1	4
mmu-miR-223-3p	227334	Usp40	1	1	0	1	1	4
mmu-miR-223-3p	227446	2310035C23Rik	1	1	0	1	1	4
mmu-miR-223-3p	227619	Man1b1	1	1	1	1	0	4
mmu-miR-223-3p	227731	Slc25a25	1	0	1	1	1	4
mmu-miR-223-3p	227800	Rabgap1	1	0	1	1	1	4
mmu-miR-223-3p	227937	Pkp4	1	1	0	1	1	4
mmu-miR-223-3p	228061	Agps	1	1	1	1	0	4
mmu-miR-223-3p	228071	Sestd1	1	1	0	1	1	4
mmu-miR-223-3p	228139	P2rx3	1	0	1	1	1	4
mmu-miR-223-3p	228356	1110051M20Rik	1	0	1	1	1	4
mmu-miR-223-3p	228359	Arhgap1	1	0	1	1	1	4
mmu-miR-223-3p	228564	Frmd5	1	0	1	1	1	4
mmu-miR-223-3p	228829	Phf20	1	1	1	1	0	4
mmu-miR-223-3p	229055	Zbtb10	1	1	0	1	1	4

mmu-miR-223-3p	229285	Spg20	1	1	0	1	1	4
mmu-miR-223-3p	229320	Clrn1	1	1	0	1	1	4
mmu-miR-223-3p	229363	Gmps	1	0	1	1	1	4
mmu-miR-223-3p	229499	Fcrl1	1	0	1	1	1	4
mmu-miR-223-3p	229503	Rrnad1	1	1	0	1	1	4
mmu-miR-223-3p	229517	Slc25a44	1	0	1	1	1	4
mmu-miR-223-3p	229644	Trim45	1	0	1	1	1	4
mmu-miR-223-3p	229675	Rsbn1	1	1	1	1	0	4
mmu-miR-223-3p	229900	Gbp7	1	1	0	1	1	4
mmu-miR-223-3p	230125	Slc25a51	1	0	1	1	1	4
mmu-miR-223-3p	230393	Focad	1	1	1	0	1	4
mmu-miR-223-3p	230696	AU022252	1	1	0	1	1	4
mmu-miR-223-3p	231470	Fras1	1	1	1	1	0	4
mmu-miR-223-3p	231549	Lrrc8d	1	1	0	1	1	4
mmu-miR-223-3p	231600	Chfr	1	0	1	1	1	4
mmu-miR-223-3p	231633	Tmem119	1	0	1	1	1	4
mmu-miR-223-3p	231646	Myo1h	1	1	0	1	1	4
mmu-miR-223-3p	231876	Lmtk2	1	0	1	1	1	4
mmu-miR-223-3p	231912	Katnal1	1	1	0	1	1	4
mmu-miR-223-3p	231986	Jazf1	1	0	1	1	1	4
mmu-miR-223-3p	231997	Fkbp14	1	0	1	1	1	4
mmu-miR-223-3p	232337	Zfp637	1	0	1	1	1	4
mmu-miR-223-3p	232341	Wnk1	1	0	1	1	1	4
mmu-miR-223-3p	232414	Clec9a	1	1	0	1	1	4
mmu-miR-223-3p	232441	Rerg	1	0	1	1	1	4
mmu-miR-223-3p	232855	Zfp772	1	1	1	1	0	4
mmu-miR-223-3p	233064	Wdr62	1	1	0	1	1	4
mmu-miR-223-3p	233103	4931406P16Rik	0	1	1	1	1	4
mmu-miR-223-3p	233107	Kctd15	1	1	1	0	1	4
mmu-miR-223-3p	233168	AI987944	1	0	1	1	1	4
mmu-miR-223-3p	233271	Luzp2	1	0	1	1	1	4
mmu-miR-223-3p	233274	Siglech	1	1	1	0	1	4
mmu-miR-223-3p	233744	Spon1	1	1	0	1	1	4
mmu-miR-223-3p	234130	Dkk4	1	0	1	1	1	4
mmu-miR-223-3p	234138	Tti2	1	1	0	1	1	4
mmu-miR-223-3p	234214	Sorbs2	1	0	1	1	1	4
mmu-miR-223-3p	234353	Psd3	1	1	0	1	1	4
mmu-miR-223-3p	234371	Tmem161a	1	1	1	0	1	4
mmu-miR-223-3p	234582	Ccdc102a	1	0	1	1	1	4
mmu-miR-223-3p	234889	Gucy1a2	1	0	1	1	1	4
mmu-miR-223-3p	235028	Zfp426	1	1	0	1	1	4
mmu-miR-223-3p	235040	Atg4d	1	0	1	1	1	4
mmu-miR-223-3p	235327	Gm4894	1	0	1	1	1	4
mmu-miR-223-3p	235633	Als2cl	1	0	1	1	1	4
mmu-miR-223-3p	235682	Zfp445	1	0	1	1	1	4
mmu-miR-223-3p	236511	Ago1	1	1	0	1	1	4
mmu-miR-223-3p	237397	C2cd4c	1	0	1	1	1	4
mmu-miR-223-3p	237403	Lingo3	1	0	1	1	1	4
mmu-miR-223-3p	237615	Ankrd52	1	0	1	1	1	4
mmu-miR-223-3p	237781	Smcr7	1	0	1	1	1	4
mmu-miR-223-3p	237860	Ssh2	1	1	0	1	1	4
mmu-miR-223-3p	237911	Brip1	1	1	1	1	0	4
mmu-miR-223-3p	237943	Gpatch8	1	1	0	1	1	4
mmu-miR-223-3p	238266	Syt16	1	1	1	0	1	4
mmu-miR-223-3p	238455	Macc1	1	1	0	1	1	4

mmu-miR-223-3p	238871	Pde4d	1	1	0	1	1	4
mmu-miR-223-3p	239099	Homez	1	0	1	1	1	4
mmu-miR-223-3p	239217	Kctd12	1	0	1	1	1	4
mmu-miR-223-3p	239528	Ago2	1	1	0	1	1	4
mmu-miR-223-3p	239606	Slc2a13	1	0	1	1	1	4
mmu-miR-223-3p	239719	Mkl2	1	1	0	1	1	4
mmu-miR-223-3p	239731	Rimbp3	1	1	1	0	1	4
mmu-miR-223-3p	239932	Krtap24-1	1	1	0	1	1	4
mmu-miR-223-3p	240038	Gm4944	1	1	0	1	1	4
mmu-miR-223-3p	240041	Zfp945	1	1	0	1	1	4
mmu-miR-223-3p	240058	Cpne5	1	0	1	1	1	4
mmu-miR-223-3p	240168	Rasgrp3	1	0	1	1	1	4
mmu-miR-223-3p	240354	Malt1	1	0	1	1	1	4
mmu-miR-223-3p	240396	Mex3c	1	1	1	0	1	4
mmu-miR-223-3p	240595	Kcnv2	1	0	1	1	1	4
mmu-miR-223-3p	240726	Slco5a1	1	0	1	1	1	4
mmu-miR-223-3p	240832	Tor1aip2	1	0	1	1	1	4
mmu-miR-223-3p	240873	Tnfsf18	1	1	1	0	1	4
mmu-miR-223-3p	241134	Nyap2	1	0	1	1	1	4
mmu-miR-223-3p	241226	Itga8	1	1	1	1	0	4
mmu-miR-223-3p	241230	St8sia6	1	1	1	0	1	4
mmu-miR-223-3p	241732	Tspyl3	1	1	1	0	1	4
mmu-miR-223-3p	241846	Lsm14b	1	0	1	1	1	4
mmu-miR-223-3p	241915	Phc3	1	1	0	1	1	4
mmu-miR-223-3p	242022	Frem2	1	1	1	1	0	4
mmu-miR-223-3p	242481	Palm2	1	0	1	1	1	4
mmu-miR-223-3p	242506	Frmd3	1	0	1	1	1	4
mmu-miR-223-3p	242523	Dmrta1	1	0	1	1	1	4
mmu-miR-223-3p	242748	Ptchd2	1	0	1	1	1	4
mmu-miR-223-3p	242785	Klhl21	1	0	1	1	1	4
mmu-miR-223-3p	243272	Sbno1	1	1	1	1	0	4
mmu-miR-223-3p	243308	A430033K04Rik	1	1	1	1	0	4
mmu-miR-223-3p	243382	Ppm1k	1	0	1	1	1	4
mmu-miR-223-3p	243621	Iqsec3	1	1	1	1	0	4
mmu-miR-223-3p	243743	Plxna4	1	1	1	1	0	4
mmu-miR-223-3p	243931	Tshz3	1	1	1	0	1	4
mmu-miR-223-3p	244373	Erlin2	1	1	1	1	0	4
mmu-miR-223-3p	244416	Ppp1r3b	1	0	1	1	1	4
mmu-miR-223-3p	244556	Zfp791	1	0	1	1	1	4
mmu-miR-223-3p	244698	Hephl1	1	1	0	1	1	4
mmu-miR-223-3p	244757	Glb1l2	1	1	1	0	1	4
mmu-miR-223-3p	244895	C230081A13Rik	1	1	1	1	0	4
mmu-miR-223-3p	245026	Col6a6	1	0	1	1	1	4
mmu-miR-223-3p	245527	Eda2r	1	0	1	1	1	4
mmu-miR-223-3p	245555	C77370	1	1	1	1	0	4
mmu-miR-223-3p	245615	Kir3dl2	1	0	1	1	1	4
mmu-miR-223-3p	245650	Gucy2f	1	0	1	1	1	4
mmu-miR-223-3p	245671	Klf8	1	0	1	1	1	4
mmu-miR-223-3p	246317	Neto1	1	1	0	1	1	4
mmu-miR-223-3p	258693	Olfr1443	1	1	1	1	0	4
mmu-miR-223-3p	259279	Tubgcp3	1	1	1	1	0	4
mmu-miR-223-3p	267019	Rps15a	1	0	1	1	1	4
mmu-miR-223-3p	268417	Zkscan17	1	0	1	1	1	4
mmu-miR-223-3p	268420	Alkbh5	1	0	1	1	1	4
mmu-miR-223-3p	268564	Zbtb1	1	1	1	1	0	4

mmu-miR-223-3p	268880	Xxylt1	1	0	1	1	1	4
mmu-miR-223-3p	268949	Dpcr1	1	1	1	0	1	4
mmu-miR-223-3p	269016	Sh3rf2	1	0	1	1	1	4
mmu-miR-223-3p	269180	Inpp4a	1	1	0	1	1	4
mmu-miR-223-3p	269473	Lrig2	1	1	1	1	0	4
mmu-miR-223-3p	269615	Plch2	1	0	1	1	1	4
mmu-miR-223-3p	269629	Lhfpl3	1	0	1	1	1	4
mmu-miR-223-3p	269637	Cnpy1	1	0	1	1	1	4
mmu-miR-223-3p	269704	Zfp664	1	1	1	1	0	4
mmu-miR-223-3p	269713	Clip2	1	1	0	1	1	4
mmu-miR-223-3p	269784	Cntn4	1	1	1	1	0	4
mmu-miR-223-3p	269788	Lhfpl4	1	0	1	1	1	4
mmu-miR-223-3p	269870	Zfp446	1	0	1	1	1	4
mmu-miR-223-3p	269994	Gsg1l	1	0	1	1	1	4
mmu-miR-223-3p	270152	Amica1	1	0	1	1	1	4
mmu-miR-223-3p	270192	Rab6b	1	0	1	1	1	4
mmu-miR-223-3p	271842	Rpusd2	1	0	1	1	1	4
mmu-miR-223-3p	279653	Pcdh19	1	1	0	1	1	4
mmu-miR-223-3p	282663	Serpinb1b	1	1	0	1	1	4
mmu-miR-223-3p	319481	Wdr59	1	0	1	1	1	4
mmu-miR-223-3p	319504	Nrcam	1	1	1	1	0	4
mmu-miR-223-3p	319636	Fsd1l	1	0	1	1	1	4
mmu-miR-223-3p	319974	Auts2	1	0	1	1	1	4
mmu-miR-223-3p	320080	Zbtb39	1	1	1	1	0	4
mmu-miR-223-3p	320129	Adrbk2	1	0	1	1	1	4
mmu-miR-223-3p	320150	Zdhhc17	1	0	1	1	1	4
mmu-miR-223-3p	320267	Fubp3	1	1	1	0	1	4
mmu-miR-223-3p	320332	Hist4h4	1	1	0	1	1	4
mmu-miR-223-3p	320495	Ipcef1	1	1	0	1	1	4
mmu-miR-223-3p	320661	D5Ertd579e	1	0	1	1	1	4
mmu-miR-223-3p	320678	Iffo1	1	1	0	1	1	4
mmu-miR-223-3p	320713	Mysm1	1	1	1	1	0	4
mmu-miR-223-3p	320722	A330050F15Rik	1	1	0	1	1	4
mmu-miR-223-3p	320736	Vstm4	1	1	1	0	1	4
mmu-miR-223-3p	320817	Atad2b	1	0	1	1	1	4
mmu-miR-223-3p	320827	C530008M17Rik	1	1	0	1	1	4
mmu-miR-223-3p	320844	Amigo3	1	1	1	0	1	4
mmu-miR-223-3p	320924	Ccbe1	1	0	1	1	1	4
mmu-miR-223-3p	320982	Arl4c	1	0	1	1	1	4
mmu-miR-223-3p	321003	Xpnpep3	1	1	1	1	0	4
mmu-miR-223-3p	321008	6330408A02Rik	1	1	1	0	1	4
mmu-miR-223-3p	321022	Cdv3	1	1	0	1	1	4
mmu-miR-223-3p	328133	Slc39a9	1	1	0	1	1	4
mmu-miR-223-3p	328365	Zmiz1	1	1	0	1	1	4
mmu-miR-223-3p	328829	9830107B12Rik	1	0	1	1	1	4
mmu-miR-223-3p	328949	Mcc	1	1	0	1	1	4
mmu-miR-223-3p	329165	Abi2	1	0	1	1	1	4
mmu-miR-223-3p	329260	Dennd1b	1	0	1	1	1	4
mmu-miR-223-3p	329739	Fam102b	1	1	0	1	1	4
mmu-miR-223-3p	330277	Fam71f1	1	1	1	0	1	4
mmu-miR-223-3p	330361	Gcfc2	1	1	1	1	0	4
mmu-miR-223-3p	330863	Trim67	1	1	0	1	1	4
mmu-miR-223-3p	330908	Opcml	1	1	1	1	0	4
mmu-miR-223-3p	330941	AI593442	1	0	1	1	1	4
mmu-miR-223-3p	338349	Cntln	1	0	1	1	1	4

mmu-miR-223-3p	338354	Zfp780b	1	0	1	1	1	4
mmu-miR-223-3p	338364	Trim65	1	0	1	1	1	4
mmu-miR-223-3p	338368	Fam109b	1	1	1	0	1	4
mmu-miR-223-3p	338371	Endov	1	1	1	1	0	4
mmu-miR-223-3p	353236	Pcdhac1	1	1	0	1	1	4
mmu-miR-223-3p	353237	Pcdhac2	1	1	0	1	1	4
mmu-miR-223-3p	380601	Fastkd5	1	0	1	1	1	4
mmu-miR-223-3p	380684	Nefh	1	1	1	0	1	4
mmu-miR-223-3p	380839	Serpinb1c	1	0	1	1	1	4
mmu-miR-223-3p	380912	Zfp395	1	0	1	1	1	4
mmu-miR-223-3p	381290	Atp2b4	1	1	0	1	1	4
mmu-miR-223-3p	381293	Kif14	1	0	1	1	1	4
mmu-miR-223-3p	381438	Gm5148	1	0	1	1	1	4
mmu-miR-223-3p	381835	Gm1078	1	1	0	1	1	4
mmu-miR-223-3p	382030	Cnep1r1	1	1	1	0	1	4
mmu-miR-223-3p	383619	Aim2	0	1	1	1	1	4
mmu-miR-223-3p	384997	Pglyrp4	1	1	0	1	1	4
mmu-miR-223-3p	385658	Nxpe3	1	1	0	1	1	4
mmu-miR-223-3p	403178	Plcxd1	1	1	0	1	1	4
mmu-miR-223-3p	403187	Opa3	1	0	1	1	1	4
mmu-miR-223-3p	404194	Gfral	1	1	1	0	1	4
mmu-miR-223-3p	432486	Gnptab	1	1	1	1	0	4
mmu-miR-223-3p	432769	Zfp708	1	1	0	1	1	4
mmu-miR-223-3p	432838	Gm5460	1	0	1	1	1	4
mmu-miR-223-3p	432860	B020004C17Rik	1	1	0	1	1	4
mmu-miR-223-3p	433022	Plcxd2	1	1	0	1	1	4
mmu-miR-223-3p	433771	Minos1	1	1	0	1	1	4
mmu-miR-223-3p	436090	Gpr62	1	1	0	1	1	4
mmu-miR-223-3p	448987	Fbxl7	1	0	1	1	1	4
mmu-miR-223-3p	544696	Tbc1d32	1	0	1	1	1	4
mmu-miR-223-3p	544817	Arhgap27	1	0	1	1	1	4
mmu-miR-223-3p	544971	Bdp1	1	0	1	1	1	4
mmu-miR-223-3p	545030	Wdfy4	1	1	0	1	1	4
mmu-miR-223-3p	546071	Mast3	1	1	0	1	1	4
mmu-miR-223-3p	547347	Gm6034	1	1	0	1	1	4
mmu-miR-223-3p	574403	Fam196b	1	0	1	1	1	4
mmu-miR-223-3p	630994	Lce3d	1	1	0	1	1	4
mmu-miR-223-3p	632687	March10	1	0	1	1	1	4
mmu-miR-223-3p	665155	Srp54b	1	0	1	1	1	4
mmu-miR-223-3p	667666	Zfp600	1	1	0	1	1	4
mmu-miR-223-3p	668225	Fignl2	1	1	0	1	1	4
mmu-miR-223-3p	791406	Gm9979	1	1	0	1	1	4
mmu-miR-223-3p	1E+08	Gm13242	1	1	1	1	0	4
mmu-miR-223-3p	1E+08	Gm13247	1	1	0	1	1	4
mmu-miR-223-3p	1E+08	Zfp831	1	0	1	1	1	4
mmu-miR-223-3p	1E+08	Srp54c	1	0	1	1	1	4
mmu-miR-223-3p	1.01E+08	Fam181a	1	1	0	1	1	4
mmu-miR-223-3p	1.01E+08	LOC101056136	1	1	0	1	1	4
mmu-miR-223-3p	11306	Abcb7	1	1	0	1	0	3
mmu-miR-223-3p	11307	Abcg1	1	0	1	1	0	3
mmu-miR-223-3p	11416	Slc33a1	0	1	1	1	0	3
mmu-miR-223-3p	11418	Asic2	1	1	0	1	0	3
mmu-miR-223-3p	11438	Chrna4	1	0	1	1	0	3
mmu-miR-223-3p	11444	Chrnb2	1	0	1	1	0	3
mmu-miR-223-3p	11461	Actb	0	1	1	1	0	3

mmu-miR-223-3p	11479	Acvr1b	1	0	1	1	0	3
mmu-miR-223-3p	11482	Acvrl1	1	0	1	1	0	3
mmu-miR-223-3p	11487	Adam10	1	0	1	1	0	3
mmu-miR-223-3p	11496	Adam22	1	0	1	1	0	3
mmu-miR-223-3p	11512	Adcy6	0	1	1	1	0	3
mmu-miR-223-3p	11516	Adcyap1	0	1	1	1	0	3
mmu-miR-223-3p	11519	Add2	1	0	0	1	1	3
mmu-miR-223-3p	11541	Adora2b	1	0	1	1	0	3
mmu-miR-223-3p	11549	Adra1a	1	0	1	1	0	3
mmu-miR-223-3p	11564	Adsl	1	0	1	1	0	3
mmu-miR-223-3p	11603	Agrn	1	0	1	1	0	3
mmu-miR-223-3p	11608	Agtr1b	1	0	1	1	0	3
mmu-miR-223-3p	11609	Agtr2	1	0	1	1	0	3
mmu-miR-223-3p	11622	Ahr	1	0	1	1	0	3
mmu-miR-223-3p	11639	Ak4	1	0	1	1	0	3
mmu-miR-223-3p	11692	Gfer	1	0	1	1	0	3
mmu-miR-223-3p	11695	Alx4	1	0	0	1	1	3
mmu-miR-223-3p	11720	Mat1a	1	0	1	1	0	3
mmu-miR-223-3p	11750	Anxa7	1	0	0	1	1	3
mmu-miR-223-3p	11778	Ap3s2	1	0	1	1	0	3
mmu-miR-223-3p	11826	Aqp1	0	1	1	1	0	3
mmu-miR-223-3p	11848	Rhoa	0	1	1	1	0	3
mmu-miR-223-3p	11865	Arntl	0	1	1	1	0	3
mmu-miR-223-3p	11876	Artn	0	1	1	1	0	3
mmu-miR-223-3p	11932	Atp1b2	1	0	1	1	0	3
mmu-miR-223-3p	11938	Atp2a2	1	0	0	1	1	3
mmu-miR-223-3p	11945	Atp4b	1	0	1	1	0	3
mmu-miR-223-3p	12013	Bach1	1	0	1	1	0	3
mmu-miR-223-3p	12043	Bcl2	1	0	1	1	0	3
mmu-miR-223-3p	12151	Bmi1	0	1	1	1	0	3
mmu-miR-223-3p	12166	Bmpr1a	1	0	1	1	0	3
mmu-miR-223-3p	12168	Bmpr2	1	0	0	1	1	3
mmu-miR-223-3p	12177	Bnip3l	1	0	1	1	0	3
mmu-miR-223-3p	12217	Bsn	1	0	1	1	0	3
mmu-miR-223-3p	12227	Btg2	1	1	0	1	0	3
mmu-miR-223-3p	12258	Serping1	0	1	1	1	0	3
mmu-miR-223-3p	12267	C3ar1	1	0	1	1	0	3
mmu-miR-223-3p	12307	Calb1	0	1	1	1	0	3
mmu-miR-223-3p	12319	Car8	1	0	1	1	0	3
mmu-miR-223-3p	12364	Casp12	0	1	1	1	0	3
mmu-miR-223-3p	12365	Casp14	0	1	1	1	0	3
mmu-miR-223-3p	12386	Ctnna2	1	0	0	1	1	3
mmu-miR-223-3p	12405	Cbln2	1	0	1	1	0	3
mmu-miR-223-3p	12416	Cbx2	0	1	1	1	0	3
mmu-miR-223-3p	12426	Cckbr	0	1	1	1	0	3
mmu-miR-223-3p	12428	Ccna2	1	0	1	1	0	3
mmu-miR-223-3p	12497	Entpd6	1	0	1	1	0	3
mmu-miR-223-3p	12508	Cd53	1	0	1	1	0	3
mmu-miR-223-3p	12518	Cd79a	1	0	0	1	1	3
mmu-miR-223-3p	12523	Cd84	1	0	1	1	0	3
mmu-miR-223-3p	12540	Cdc42	1	0	1	1	0	3
mmu-miR-223-3p	12545	Cdc7	1	0	1	1	0	3
mmu-miR-223-3p	12549	Arhgap31	1	0	1	1	0	3
mmu-miR-223-3p	12564	Cdh8	1	0	1	1	0	3
mmu-miR-223-3p	12593	Cdyl	1	0	0	1	1	3

mmu-miR-223-3p	12608	Cebpb	0	1	1	1	0	3
mmu-miR-223-3p	12614	Celsr1	0	1	1	1	0	3
mmu-miR-223-3p	12638	Cftr	1	0	1	0	1	3
mmu-miR-223-3p	12672	Chrm4	1	0	1	1	0	3
mmu-miR-223-3p	12675	Chuk	0	1	1	1	0	3
mmu-miR-223-3p	12729	Clns1a	1	0	0	1	1	3
mmu-miR-223-3p	12737	Cldn1	1	0	1	1	0	3
mmu-miR-223-3p	12767	Cxcr4	1	0	1	1	0	3
mmu-miR-223-3p	12769	Ccr9	1	0	1	1	0	3
mmu-miR-223-3p	12826	Col4a1	1	0	1	1	0	3
mmu-miR-223-3p	12828	Col4a3	0	1	1	1	0	3
mmu-miR-223-3p	12829	Col4a4	1	0	1	1	0	3
mmu-miR-223-3p	12889	Cplx1	1	0	1	1	0	3
mmu-miR-223-3p	12912	Creb1	1	0	0	1	1	3
mmu-miR-223-3p	12929	Crkl	1	0	1	1	0	3
mmu-miR-223-3p	12937	Pcdha6	1	0	0	1	1	3
mmu-miR-223-3p	12939	Pcdha7	1	0	0	1	1	3
mmu-miR-223-3p	12941	Pcdha5	1	0	0	1	1	3
mmu-miR-223-3p	12985	Csf3	1	0	1	1	0	3
mmu-miR-223-3p	13004	Ncan	1	1	0	1	0	3
mmu-miR-223-3p	13017	Ctbp2	1	0	1	1	0	3
mmu-miR-223-3p	13034	Ctse	1	0	1	1	0	3
mmu-miR-223-3p	13036	Ctsh	1	0	1	1	0	3
mmu-miR-223-3p	13043	Cttn	1	0	1	1	0	3
mmu-miR-223-3p	13096	Cyp2c37	0	1	1	1	0	3
mmu-miR-223-3p	13116	Cyp46a1	0	1	1	1	0	3
mmu-miR-223-3p	13135	Dad1	1	0	1	1	0	3
mmu-miR-223-3p	13164	Dazl	1	1	0	1	0	3
mmu-miR-223-3p	13175	Dclk1	1	0	1	1	0	3
mmu-miR-223-3p	13193	Dcx	1	1	0	1	0	3
mmu-miR-223-3p	13204	Dhx15	0	1	1	1	0	3
mmu-miR-223-3p	13205	Ddx3x	1	0	1	1	0	3
mmu-miR-223-3p	13244	Degs1	1	0	1	1	0	3
mmu-miR-223-3p	13340	Slc29a2	1	0	1	1	0	3
mmu-miR-223-3p	13356	Dgcr2	1	1	0	1	0	3
mmu-miR-223-3p	13361	Dhfr	1	0	1	1	0	3
mmu-miR-223-3p	13368	Dffb	1	0	1	1	0	3
mmu-miR-223-3p	13392	Dlx2	1	0	1	1	0	3
mmu-miR-223-3p	13405	Dmd	1	1	0	1	0	3
mmu-miR-223-3p	13409	Tmc1	0	1	1	1	0	3
mmu-miR-223-3p	13418	Dnajc1	1	0	0	1	1	3
mmu-miR-223-3p	13429	Dnm1	0	1	1	1	0	3
mmu-miR-223-3p	13435	Dnmt3a	1	0	1	1	0	3
mmu-miR-223-3p	13482	Dpp4	0	1	1	1	0	3
mmu-miR-223-3p	13488	Drd1a	0	1	1	1	0	3
mmu-miR-223-3p	13507	Dsc3	1	0	1	1	0	3
mmu-miR-223-3p	13518	Dst	1	1	0	1	0	3
mmu-miR-223-3p	13524	Adam18	1	0	1	1	0	3
mmu-miR-223-3p	13549	Dyrk1b	0	1	1	1	0	3
mmu-miR-223-3p	13557	E2f3	1	0	1	1	0	3
mmu-miR-223-3p	13607	Eda	1	0	1	1	0	3
mmu-miR-223-3p	13616	Edn3	1	0	1	1	0	3
mmu-miR-223-3p	13661	Ehf	1	0	1	1	0	3
mmu-miR-223-3p	13669	Eif3a	0	1	1	1	0	3
mmu-miR-223-3p	13684	Eif4e	1	0	1	1	0	3

mmu-miR-223-3p	13797	Emx2	1	1	0	1	0	3
mmu-miR-223-3p	13803	Enc1	1	0	1	1	0	3
mmu-miR-223-3p	13809	Enpep	1	0	1	1	0	3
mmu-miR-223-3p	13813	Eomes	1	0	1	1	0	3
mmu-miR-223-3p	13819	Epas1	0	1	1	1	0	3
mmu-miR-223-3p	13841	Epha7	1	0	1	1	0	3
mmu-miR-223-3p	13844	Ephb2	0	1	1	1	0	3
mmu-miR-223-3p	13874	Ereg	1	0	1	1	0	3
mmu-miR-223-3p	14012	Mpzl2	1	0	1	1	0	3
mmu-miR-223-3p	14020	Evi5	1	0	1	1	0	3
mmu-miR-223-3p	14026	Evl	1	0	1	1	0	3
mmu-miR-223-3p	14048	Eya1	1	0	1	1	0	3
mmu-miR-223-3p	14057	Sfxn1	0	1	1	1	0	3
mmu-miR-223-3p	14088	Fancc	1	0	1	1	0	3
mmu-miR-223-3p	14105	Srsf10	1	0	1	1	0	3
mmu-miR-223-3p	14119	Fbn2	1	0	1	1	0	3
mmu-miR-223-3p	14151	Fech	1	0	1	1	0	3
mmu-miR-223-3p	14169	Fgf14	1	1	0	1	0	3
mmu-miR-223-3p	14202	Fhl4	1	0	1	1	0	3
mmu-miR-223-3p	14221	Fjx1	1	0	1	1	0	3
mmu-miR-223-3p	14229	Fkbp5	1	0	1	1	0	3
mmu-miR-223-3p	14230	Fkbp10	1	0	1	1	0	3
mmu-miR-223-3p	14236	Foxn2	0	1	1	1	0	3
mmu-miR-223-3p	14256	Flt3l	0	1	1	1	0	3
mmu-miR-223-3p	14260	Fmn1	1	1	0	1	0	3
mmu-miR-223-3p	14282	Fosb	1	0	1	1	0	3
mmu-miR-223-3p	14313	Fst	0	1	1	1	0	3
mmu-miR-223-3p	14360	Fyn	1	1	0	1	0	3
mmu-miR-223-3p	14362	Fzd1	1	0	1	1	0	3
mmu-miR-223-3p	14365	Fzd3	1	0	0	1	1	3
mmu-miR-223-3p	14366	Fzd4	1	1	1	0	0	3
mmu-miR-223-3p	14369	Fzd7	1	0	1	1	0	3
mmu-miR-223-3p	14390	Gabpa	0	1	1	1	0	3
mmu-miR-223-3p	14396	Gabra3	1	0	1	1	0	3
mmu-miR-223-3p	14397	Gabra4	1	0	1	1	0	3
mmu-miR-223-3p	14415	Gad1	0	1	1	1	0	3
mmu-miR-223-3p	14420	Galc	1	0	1	1	0	3
mmu-miR-223-3p	14422	B4galnt2	0	1	1	1	0	3
mmu-miR-223-3p	14423	Galnt1	1	0	1	1	0	3
mmu-miR-223-3p	14426	Galnt4	1	0	1	1	0	3
mmu-miR-223-3p	14451	Gas1	1	0	1	1	0	3
mmu-miR-223-3p	14465	Gata6	0	1	1	1	0	3
mmu-miR-223-3p	14466	Gba	1	0	0	1	1	3
mmu-miR-223-3p	14528	Gch1	0	1	1	1	0	3
mmu-miR-223-3p	14536	Nr6a1	1	0	1	1	0	3
mmu-miR-223-3p	14555	Gpd1	0	1	1	1	0	3
mmu-miR-223-3p	14567	Gdi1	0	1	1	1	0	3
mmu-miR-223-3p	14569	Gdi2	1	0	1	1	0	3
mmu-miR-223-3p	14616	Gja8	1	0	0	1	1	3
mmu-miR-223-3p	14634	Gli3	0	1	1	1	0	3
mmu-miR-223-3p	14658	Glrb	1	1	0	1	0	3
mmu-miR-223-3p	14696	Gnb4	0	1	1	1	0	3
mmu-miR-223-3p	14697	Gnb5	1	0	0	1	1	3
mmu-miR-223-3p	14706	Gng4	1	1	0	1	0	3
mmu-miR-223-3p	14729	Gp5	1	0	0	1	1	3

mmu-miR-223-3p	14772	Grk4	1	0	1	1	0	3
mmu-miR-223-3p	14783	Grb10	1	0	1	1	0	3
mmu-miR-223-3p	14792	Lpcat3	1	1	0	1	0	3
mmu-miR-223-3p	14799	Gria1	1	0	0	1	1	3
mmu-miR-223-3p	14812	Grin2b	1	0	1	1	0	3
mmu-miR-223-3p	14815	Nr3c1	1	0	1	1	0	3
mmu-miR-223-3p	14924	Magi1	1	1	0	1	0	3
mmu-miR-223-3p	14950	H13	1	0	0	1	1	3
mmu-miR-223-3p	15061	Ifi44l	1	0	1	1	0	3
mmu-miR-223-3p	15081	H3f3b	0	1	1	1	0	3
mmu-miR-223-3p	15117	Has2	0	1	1	1	0	3
mmu-miR-223-3p	15161	Hcfc1	1	0	1	1	0	3
mmu-miR-223-3p	15191	Hdgf	1	0	1	1	0	3
mmu-miR-223-3p	15205	Hes1	0	1	1	1	0	3
mmu-miR-223-3p	15221	Foxd3	1	0	1	1	0	3
mmu-miR-223-3p	15277	Hk2	0	1	1	1	0	3
mmu-miR-223-3p	15360	Hmgcs2	1	1	0	1	0	3
mmu-miR-223-3p	15375	Foxa1	1	0	1	1	0	3
mmu-miR-223-3p	15422	Hoxc13	1	0	1	1	0	3
mmu-miR-223-3p	15424	Hoxc5	0	1	1	1	0	3
mmu-miR-223-3p	15425	Hoxc6	1	0	1	1	0	3
mmu-miR-223-3p	15431	Hoxd11	1	0	0	1	1	3
mmu-miR-223-3p	15490	Hsd17b7	1	0	1	1	0	3
mmu-miR-223-3p	15499	Hsf1	0	1	1	1	0	3
mmu-miR-223-3p	15531	Ndst1	1	0	1	1	0	3
mmu-miR-223-3p	15552	Htr1d	1	0	1	0	1	3
mmu-miR-223-3p	15560	Htr2c	1	0	1	1	0	3
mmu-miR-223-3p	15562	Htr4	1	0	1	1	0	3
mmu-miR-223-3p	15563	Htr5a	1	0	1	1	0	3
mmu-miR-223-3p	15586	Hyal1	1	0	1	0	1	3
mmu-miR-223-3p	15900	Irf8	1	0	1	1	0	3
mmu-miR-223-3p	15958	Ifit2	1	0	1	1	0	3
mmu-miR-223-3p	15975	Ifnar1	1	0	1	1	0	3
mmu-miR-223-3p	16155	Il10rb	1	1	0	1	0	3
mmu-miR-223-3p	16179	Irak1	1	0	1	1	0	3
mmu-miR-223-3p	16182	Il18r1	1	0	1	1	0	3
mmu-miR-223-3p	16184	Il2ra	1	0	1	1	0	3
mmu-miR-223-3p	16197	Il7r	1	0	1	1	0	3
mmu-miR-223-3p	16337	Insr	1	0	1	1	0	3
mmu-miR-223-3p	16362	Irf1	1	0	0	1	1	3
mmu-miR-223-3p	16370	Irs4	1	0	1	1	0	3
mmu-miR-223-3p	16396	Itch	1	0	1	1	0	3
mmu-miR-223-3p	16400	Itga3	1	0	1	1	0	3
mmu-miR-223-3p	16410	Itgav	1	0	1	1	0	3
mmu-miR-223-3p	16431	Itm2a	0	1	1	1	0	3
mmu-miR-223-3p	16439	Itpr2	0	1	0	1	1	3
mmu-miR-223-3p	16449	Jag1	0	1	1	1	0	3
mmu-miR-223-3p	16468	Jarid2	1	0	1	1	0	3
mmu-miR-223-3p	16485	Kcna1	1	0	1	1	0	3
mmu-miR-223-3p	16502	Kcnc1	1	0	1	0	1	3
mmu-miR-223-3p	16508	Kcnd2	1	0	1	1	0	3
mmu-miR-223-3p	16518	Kcnj2	1	0	1	1	0	3
mmu-miR-223-3p	16526	Kcnk2	1	0	1	1	0	3
mmu-miR-223-3p	16558	Kif16b	0	1	1	1	0	3
mmu-miR-223-3p	16568	Kif3a	1	0	1	1	0	3

mmu-miR-223-3p	16570	Kif3c	1	0	1	1	0	3
mmu-miR-223-3p	16574	Kif5c	1	0	1	1	0	3
mmu-miR-223-3p	16590	Kit	1	0	0	1	1	3
mmu-miR-223-3p	16591	Kl	1	1	1	0	0	3
mmu-miR-223-3p	16650	Kpna6	1	0	1	1	0	3
mmu-miR-223-3p	16709	Ktn1	0	1	1	1	0	3
mmu-miR-223-3p	16716	Ky	1	0	1	1	0	3
mmu-miR-223-3p	16765	Stmn1	1	1	0	0	1	3
mmu-miR-223-3p	16785	Rpsa	1	0	1	0	1	3
mmu-miR-223-3p	16846	Lep	1	0	1	1	0	3
mmu-miR-223-3p	16847	Lepr	1	0	1	1	0	3
mmu-miR-223-3p	16859	Lgals9	1	0	0	1	1	3
mmu-miR-223-3p	16876	Lhx9	1	1	0	1	0	3
mmu-miR-223-3p	16948	Lox	1	1	0	1	0	3
mmu-miR-223-3p	16950	Loxl3	1	0	1	1	0	3
mmu-miR-223-3p	16974	Lrp6	0	1	1	1	0	3
mmu-miR-223-3p	16987	Lss	1	0	1	1	0	3
mmu-miR-223-3p	16994	Ltb	0	1	1	1	0	3
mmu-miR-223-3p	17025	Alad	1	0	0	1	1	3
mmu-miR-223-3p	17118	Marcks	1	0	1	1	0	3
mmu-miR-223-3p	17129	Smad5	1	0	1	1	0	3
mmu-miR-223-3p	17173	Ascl2	0	1	1	1	0	3
mmu-miR-223-3p	17191	Mbd2	0	1	1	1	0	3
mmu-miR-223-3p	17192	Mbd3	1	0	1	1	0	3
mmu-miR-223-3p	17207	Mcf2l	1	0	1	1	0	3
mmu-miR-223-3p	17245	Mdm1	1	0	1	1	0	3
mmu-miR-223-3p	17252	Rdh11	1	1	0	1	0	3
mmu-miR-223-3p	17258	Mef2a	1	1	1	0	0	3
mmu-miR-223-3p	17283	Men1	1	0	1	1	0	3
mmu-miR-223-3p	17306	Sypl2	1	0	1	1	0	3
mmu-miR-223-3p	17311	Kitl	0	1	1	1	0	3
mmu-miR-223-3p	17355	Aff1	1	0	0	1	1	3
mmu-miR-223-3p	17380	Mme	1	0	1	1	0	3
mmu-miR-223-3p	17385	Mmp11	1	0	1	1	0	3
mmu-miR-223-3p	17391	Mmp24	1	0	1	1	0	3
mmu-miR-223-3p	17428	Mnt	1	0	1	1	0	3
mmu-miR-223-3p	17535	Mre11a	0	1	1	1	0	3
mmu-miR-223-3p	17686	Msh3	1	0	1	1	0	3
mmu-miR-223-3p	17754	Map1a	0	1	1	1	0	3
mmu-miR-223-3p	17761	Map7	1	0	1	0	1	3
mmu-miR-223-3p	17763	Mtcp1	0	1	1	1	0	3
mmu-miR-223-3p	17764	Mtf1	1	0	1	1	0	3
mmu-miR-223-3p	17769	Mthfr	1	0	1	1	0	3
mmu-miR-223-3p	17827	Mtx1	0	1	1	1	0	3
mmu-miR-223-3p	17846	Commd1	0	1	1	1	0	3
mmu-miR-223-3p	17847	Usp34	1	0	0	1	1	3
mmu-miR-223-3p	17920	Myo6	0	1	1	1	0	3
mmu-miR-223-3p	17936	Nab1	1	0	1	1	0	3
mmu-miR-223-3p	17966	Nbr1	1	0	1	1	0	3
mmu-miR-223-3p	17968	Ncam2	1	0	1	1	0	3
mmu-miR-223-3p	17974	Nck2	0	1	1	1	0	3
mmu-miR-223-3p	17975	Ncl	1	0	1	1	0	3
mmu-miR-223-3p	17986	Ndp	0	1	1	1	0	3
mmu-miR-223-3p	18007	Neo1	1	0	0	1	1	3
mmu-miR-223-3p	18010	Neu1	1	0	0	1	1	3

mmu-miR-223-3p	18016	Nf2	1	1	0	1	0	3
mmu-miR-223-3p	18019	Nfatc2	1	0	1	1	0	3
mmu-miR-223-3p	18029	Nfic	1	0	0	1	1	3
mmu-miR-223-3p	18041	Nfs1	1	0	1	1	0	3
mmu-miR-223-3p	18053	Ngfr	0	1	1	1	0	3
mmu-miR-223-3p	18087	Nktr	1	0	1	1	0	3
mmu-miR-223-3p	18103	Nme2	1	0	0	1	1	3
mmu-miR-223-3p	18105	Nqo2	1	0	1	1	0	3
mmu-miR-223-3p	18106	Cd244	1	0	1	1	0	3
mmu-miR-223-3p	18120	Mrpl49	1	0	1	1	0	3
mmu-miR-223-3p	18129	Notch2	1	0	1	1	0	3
mmu-miR-223-3p	18131	Notch3	1	1	0	1	0	3
mmu-miR-223-3p	18139	Zfml	1	0	1	1	0	3
mmu-miR-223-3p	18164	Nptx1	1	1	0	1	0	3
mmu-miR-223-3p	18167	Npy2r	1	0	1	1	0	3
mmu-miR-223-3p	18176	Nras	1	0	1	1	0	3
mmu-miR-223-3p	18183	Nrg3	1	0	0	1	1	3
mmu-miR-223-3p	18218	Dusp8	1	0	1	1	0	3
mmu-miR-223-3p	18247	Oaz2	1	0	1	1	0	3
mmu-miR-223-3p	18295	Ogn	1	0	1	1	0	3
mmu-miR-223-3p	18415	Hspa4l	0	1	1	1	0	3
mmu-miR-223-3p	18423	Otx1	1	0	1	1	0	3
mmu-miR-223-3p	18430	Oxtr	1	0	1	1	0	3
mmu-miR-223-3p	18439	P2rx7	0	1	1	1	0	3
mmu-miR-223-3p	18451	P4ha1	1	0	1	1	0	3
mmu-miR-223-3p	18457	Bloc1s6	1	0	1	1	0	3
mmu-miR-223-3p	18459	Pabpc2	1	0	0	1	1	3
mmu-miR-223-3p	18479	Pak1	0	1	1	1	0	3
mmu-miR-223-3p	18491	Pappa	1	0	1	1	0	3
mmu-miR-223-3p	18504	Pax2	1	0	0	1	1	3
mmu-miR-223-3p	18514	Pbx1	1	0	1	1	0	3
mmu-miR-223-3p	18516	Pbx3	0	1	1	1	0	3
mmu-miR-223-3p	18519	Kat2b	1	0	1	1	0	3
mmu-miR-223-3p	18573	Pde1a	1	0	1	1	0	3
mmu-miR-223-3p	18596	Pdgfrb	1	0	1	0	1	3
mmu-miR-223-3p	18627	Per2	1	1	0	1	0	3
mmu-miR-223-3p	18632	Pex11b	1	0	0	1	1	3
mmu-miR-223-3p	18671	Abcb1a	1	0	1	1	0	3
mmu-miR-223-3p	18740	Pitx1	0	1	1	1	0	3
mmu-miR-223-3p	18749	Prkacb	1	0	1	1	0	3
mmu-miR-223-3p	18750	Prkca	1	0	1	1	0	3
mmu-miR-223-3p	18754	Prkce	1	0	0	1	1	3
mmu-miR-223-3p	18759	Prkci	1	0	1	1	0	3
mmu-miR-223-3p	18768	Pkib	1	1	0	1	0	3
mmu-miR-223-3p	18776	Prl3b1	1	0	1	1	0	3
mmu-miR-223-3p	18821	Pln	1	0	1	1	0	3
mmu-miR-223-3p	18854	Pml	1	0	0	1	1	3
mmu-miR-223-3p	18973	Pole	0	1	1	1	0	3
mmu-miR-223-3p	18996	Pou4f1	1	0	1	1	0	3
mmu-miR-223-3p	19013	Ppara	1	0	0	1	1	3
mmu-miR-223-3p	19015	Ppard	0	1	1	1	0	3
mmu-miR-223-3p	19018	Scand1	1	0	1	0	1	3
mmu-miR-223-3p	19039	Lgals3bp	1	0	1	1	0	3
mmu-miR-223-3p	19153	Prx	1	0	0	1	1	3
mmu-miR-223-3p	19159	Cyth3	1	0	1	1	0	3

mmu-miR-223-3p	19167	Psma3	1	0	1	1	0	3
mmu-miR-223-3p	19210	Ptdss1	1	0	1	1	0	3
mmu-miR-223-3p	19211	Pten	1	0	1	1	0	3
mmu-miR-223-3p	19214	Ptgdr	1	0	1	1	0	3
mmu-miR-223-3p	19222	Ptgir	1	0	1	0	1	3
mmu-miR-223-3p	19226	Pth	1	0	1	0	1	3
mmu-miR-223-3p	19243	Ptp4a1	1	0	1	1	0	3
mmu-miR-223-3p	19250	Ptpn14	1	0	1	1	0	3
mmu-miR-223-3p	19258	Ptpn4	1	0	1	1	0	3
mmu-miR-223-3p	19271	Ptprj	1	0	1	1	0	3
mmu-miR-223-3p	19344	Rab5b	1	1	0	1	0	3
mmu-miR-223-3p	19353	Rac1	1	0	1	1	0	3
mmu-miR-223-3p	19356	Rad17	1	0	0	1	1	3
mmu-miR-223-3p	19361	Rad51	1	0	1	1	0	3
mmu-miR-223-3p	19363	Rad51b	1	0	1	1	0	3
mmu-miR-223-3p	19647	Rbbp6	1	0	1	0	1	3
mmu-miR-223-3p	19683	Rdh16	0	1	1	1	0	3
mmu-miR-223-3p	19691	Recql	1	0	0	1	1	3
mmu-miR-223-3p	19712	Rest	1	1	0	1	0	3
mmu-miR-223-3p	19727	Rfxank	1	0	0	1	1	3
mmu-miR-223-3p	19739	Rgs9	1	0	0	1	1	3
mmu-miR-223-3p	19765	Ralbp1	1	0	1	1	0	3
mmu-miR-223-3p	19876	Robo1	1	0	1	1	0	3
mmu-miR-223-3p	19879	Slc22a8	1	0	1	1	0	3
mmu-miR-223-3p	19888	Rp1	1	0	0	1	1	3
mmu-miR-223-3p	19889	Rp2h	1	0	1	1	0	3
mmu-miR-223-3p	19893	Rpgr	1	0	0	1	1	3
mmu-miR-223-3p	19934	Rpl22	0	1	1	1	0	3
mmu-miR-223-3p	20019	Polr1a	0	1	1	1	0	3
mmu-miR-223-3p	20203	S100b	1	0	1	1	0	3
mmu-miR-223-3p	20230	Satb1	1	0	1	1	0	3
mmu-miR-223-3p	20250	Scd2	1	0	1	1	0	3
mmu-miR-223-3p	20257	Stmn2	1	0	1	1	0	3
mmu-miR-223-3p	20271	Scn5a	1	1	0	1	0	3
mmu-miR-223-3p	20277	Scnn1b	1	0	0	1	1	3
mmu-miR-223-3p	20308	Ccl9	1	0	1	1	0	3
mmu-miR-223-3p	20320	Nptn	1	0	1	1	0	3
mmu-miR-223-3p	20322	Sord	1	0	1	1	0	3
mmu-miR-223-3p	20324	Sdpr	1	0	1	1	0	3
mmu-miR-223-3p	20356	Sema5a	1	0	1	1	0	3
mmu-miR-223-3p	20361	Sema7a	1	0	1	1	0	3
mmu-miR-223-3p	20383	Srsf3	0	1	1	1	0	3
mmu-miR-223-3p	20391	Sgca	1	0	0	1	1	3
mmu-miR-223-3p	20404	Sh3gl2	0	1	1	1	0	3
mmu-miR-223-3p	20408	Sh3gl3	0	1	1	1	0	3
mmu-miR-223-3p	20411	Sorbs1	1	0	0	1	1	3
mmu-miR-223-3p	20423	Shh	1	0	1	1	0	3
mmu-miR-223-3p	20430	Cyfip1	1	0	1	1	0	3
mmu-miR-223-3p	20446	St6galnac2	1	0	1	1	0	3
mmu-miR-223-3p	20448	St6galnac4	1	0	1	1	0	3
mmu-miR-223-3p	20450	St8sia2	0	1	1	1	0	3
mmu-miR-223-3p	20474	Six4	0	1	1	1	0	3
mmu-miR-223-3p	20492	Slbp	0	1	1	1	0	3
mmu-miR-223-3p	20502	Slc16a2	1	0	1	1	0	3
mmu-miR-223-3p	20511	Slc1a2	1	1	0	1	0	3

mmu-miR-223-3p	20515	Slc20a1	1	0	0	1	1	3
mmu-miR-223-3p	20520	Slc22a5	1	0	1	1	0	3
mmu-miR-223-3p	20539	Slc7a5	1	0	1	0	1	3
mmu-miR-223-3p	20541	Slc8a1	1	0	0	1	1	3
mmu-miR-223-3p	20605	Sstr1	1	0	1	1	0	3
mmu-miR-223-3p	20607	Sstr3	1	0	1	1	0	3
mmu-miR-223-3p	20610	Sumo3	0	1	1	1	0	3
mmu-miR-223-3p	20616	Snap91	1	0	1	1	0	3
mmu-miR-223-3p	20667	Sox12	1	1	0	1	0	3
mmu-miR-223-3p	20678	Sox5	1	1	0	1	0	3
mmu-miR-223-3p	20688	Sp4	1	0	1	1	0	3
mmu-miR-223-3p	20689	Sall3	0	1	1	1	0	3
mmu-miR-223-3p	20707	Serpinb9c	1	0	1	1	0	3
mmu-miR-223-3p	20737	Spn	1	0	1	1	0	3
mmu-miR-223-3p	20745	Spock1	1	0	1	1	0	3
mmu-miR-223-3p	20773	Sptlc2	1	1	0	1	0	3
mmu-miR-223-3p	20815	Srpk1	0	1	1	1	0	3
mmu-miR-223-3p	20818	Srprb	1	0	1	1	0	3
mmu-miR-223-3p	20834	Znrf4	1	0	0	1	1	3
mmu-miR-223-3p	20845	Star	0	1	1	1	0	3
mmu-miR-223-3p	20855	Stc1	0	1	1	1	0	3
mmu-miR-223-3p	20908	Stx3	1	0	0	1	1	3
mmu-miR-223-3p	20963	Syk	0	1	1	1	0	3
mmu-miR-223-3p	20964	Syn1	1	0	1	0	1	3
mmu-miR-223-3p	20979	Syt1	1	0	1	1	0	3
mmu-miR-223-3p	21336	Tacr1	1	0	1	1	0	3
mmu-miR-223-3p	21338	Tacr3	1	0	1	1	0	3
mmu-miR-223-3p	21366	Slc6a6	1	0	1	1	0	3
mmu-miR-223-3p	21367	Cntn2	1	0	1	1	0	3
mmu-miR-223-3p	21372	Tbl1x	1	1	0	1	0	3
mmu-miR-223-3p	21379	Tbrg4	1	1	0	1	0	3
mmu-miR-223-3p	21384	Tbx15	0	1	1	1	0	3
mmu-miR-223-3p	21399	Tcea1	0	1	1	1	0	3
mmu-miR-223-3p	21406	Tcf12	1	0	1	1	0	3
mmu-miR-223-3p	21646	Tcte2	0	1	1	1	0	3
mmu-miR-223-3p	21676	Tead1	1	0	1	1	0	3
mmu-miR-223-3p	21763	Tex2	1	0	1	1	0	3
mmu-miR-223-3p	21788	Tfpi	1	1	0	1	0	3
mmu-miR-223-3p	21802	Tgfa	1	0	1	1	0	3
mmu-miR-223-3p	21808	Tgfb2	1	0	1	1	0	3
mmu-miR-223-3p	21817	Tgm2	1	0	1	1	0	3
mmu-miR-223-3p	21824	Thbd	1	0	1	1	0	3
mmu-miR-223-3p	21844	Tiam1	1	0	1	1	0	3
mmu-miR-223-3p	21858	Timp2	1	0	1	1	0	3
mmu-miR-223-3p	21917	Tmpo	1	0	0	1	1	3
mmu-miR-223-3p	21927	Tnfaip1	0	1	1	1	0	3
mmu-miR-223-3p	21934	Tnfrsf11a	1	0	1	1	0	3
mmu-miR-223-3p	21945	Dedd	1	0	1	1	0	3
mmu-miR-223-3p	21991	Tpi1	1	0	1	0	1	3
mmu-miR-223-3p	22031	Traf3	1	1	0	1	0	3
mmu-miR-223-3p	22032	Traf4	1	0	1	0	1	3
mmu-miR-223-3p	22035	Tnfsf10	1	0	1	1	0	3
mmu-miR-223-3p	22057	Tob1	0	1	1	1	0	3
mmu-miR-223-3p	22066	Trpc4	1	0	0	1	1	3
mmu-miR-223-3p	22115	Tssk2	0	1	1	1	0	3

mmu-miR-223-3p	22138	Ttn	0	1	0	1	1	3
mmu-miR-223-3p	22154	Tubb5	1	1	0	1	0	3
mmu-miR-223-3p	22169	Cmpk2	0	1	1	1	0	3
mmu-miR-223-3p	22171	Tyms	1	0	1	1	0	3
mmu-miR-223-3p	22183	Zrsr1	1	0	1	1	0	3
mmu-miR-223-3p	22194	Ube2e1	1	0	1	1	0	3
mmu-miR-223-3p	22200	Uba3	1	1	0	1	0	3
mmu-miR-223-3p	22214	Ube2h	1	0	0	1	1	3
mmu-miR-223-3p	22217	Usp12	1	0	1	1	0	3
mmu-miR-223-3p	22228	Ucp2	1	0	1	1	0	3
mmu-miR-223-3p	22229	Ucp3	1	1	1	0	0	3
mmu-miR-223-3p	22232	Slc35a2	0	1	1	1	0	3
mmu-miR-223-3p	22238	Ugt2b5	1	0	0	1	1	3
mmu-miR-223-3p	22288	Utrn	0	1	1	1	0	3
mmu-miR-223-3p	22297	Vmn1r45	1	0	1	1	0	3
mmu-miR-223-3p	22329	Vcam1	1	0	1	1	0	3
mmu-miR-223-3p	22344	Vezf1	1	0	1	1	0	3
mmu-miR-223-3p	22354	Vipr1	1	0	1	1	0	3
mmu-miR-223-3p	22390	Wee1	1	0	1	1	0	3
mmu-miR-223-3p	22401	Zmat3	0	1	1	1	0	3
mmu-miR-223-3p	22411	Wnt11	1	0	1	1	0	3
mmu-miR-223-3p	22414	Wnt2b	1	0	1	1	0	3
mmu-miR-223-3p	22437	Xirp1	1	0	0	1	1	3
mmu-miR-223-3p	22599	Slc6a20b	1	1	0	0	1	3
mmu-miR-223-3p	22634	Plagl1	1	1	0	1	0	3
mmu-miR-223-3p	22695	Zfp36	1	0	1	1	0	3
mmu-miR-223-3p	22722	Zfp64	1	1	0	1	0	3
mmu-miR-223-3p	22773	Zic3	1	0	1	1	0	3
mmu-miR-223-3p	22775	Zik1	1	0	1	1	0	3
mmu-miR-223-3p	22779	Ikzf2	1	1	0	1	0	3
mmu-miR-223-3p	22780	Ikzf3	1	0	1	1	0	3
mmu-miR-223-3p	23790	Coro1c	0	1	1	1	0	3
mmu-miR-223-3p	23806	Arih1	0	1	1	1	0	3
mmu-miR-223-3p	23850	Pappa2	1	0	1	1	0	3
mmu-miR-223-3p	23871	Ets1	1	0	0	1	1	3
mmu-miR-223-3p	23887	Ggt5	1	0	1	1	0	3
mmu-miR-223-3p	23893	Grem2	1	0	1	1	0	3
mmu-miR-223-3p	23972	Papss2	1	0	1	1	0	3
mmu-miR-223-3p	24013	Grk1	1	0	1	1	0	3
mmu-miR-223-3p	24050	Sept3	1	0	1	1	0	3
mmu-miR-223-3p	24051	Sgcb	1	0	1	1	0	3
mmu-miR-223-3p	24066	Spry4	1	0	1	1	0	3
mmu-miR-223-3p	24067	Srp54a	1	0	0	1	1	3
mmu-miR-223-3p	24127	Xrn1	1	0	1	1	0	3
mmu-miR-223-3p	24135	Zfp68	1	0	0	1	1	3
mmu-miR-223-3p	26361	Avpr1b	0	1	1	1	0	3
mmu-miR-223-3p	26365	Ceacam1	1	0	0	1	1	3
mmu-miR-223-3p	26367	Ceacam2	1	0	0	1	1	3
mmu-miR-223-3p	26374	Rfwd2	1	0	1	1	0	3
mmu-miR-223-3p	26408	Map3k5	0	1	1	1	0	3
mmu-miR-223-3p	26413	Mapk1	1	0	1	1	0	3
mmu-miR-223-3p	26556	Homer1	1	1	0	1	0	3
mmu-miR-223-3p	26874	Abcd2	1	0	1	1	0	3
mmu-miR-223-3p	26877	B3galt1	1	0	1	1	0	3
mmu-miR-223-3p	26889	Cln8	1	0	1	1	0	3

mmu-miR-223-3p	26896	Med14	1	0	1	1	0	3
mmu-miR-223-3p	26932	Ppp2r5e	1	0	1	1	0	3
mmu-miR-223-3p	26943	Serinc3	0	1	1	1	0	3
mmu-miR-223-3p	27015	Polk	1	1	1	0	0	3
mmu-miR-223-3p	27049	Etv3	1	1	0	1	0	3
mmu-miR-223-3p	27058	Srp9	1	0	1	0	1	3
mmu-miR-223-3p	27059	Sh3d19	1	0	1	1	0	3
mmu-miR-223-3p	27096	Trappc3	1	0	1	1	0	3
mmu-miR-223-3p	27204	Syn3	1	0	0	1	1	3
mmu-miR-223-3p	27217	Mixl1	1	0	1	1	0	3
mmu-miR-223-3p	27223	Trp53bp1	0	1	1	1	0	3
mmu-miR-223-3p	27366	Txnl4a	1	0	1	1	0	3
mmu-miR-223-3p	27368	Tbl2	1	0	1	1	0	3
mmu-miR-223-3p	27380	Tcl1b4	1	0	1	1	0	3
mmu-miR-223-3p	27388	Ptdss2	1	1	0	1	0	3
mmu-miR-223-3p	27395	Mrpl15	1	0	0	1	1	3
mmu-miR-223-3p	27401	Skp2	0	1	1	1	0	3
mmu-miR-223-3p	27418	Mkln1	0	1	1	1	0	3
mmu-miR-223-3p	27494	Amot	1	0	1	1	0	3
mmu-miR-223-3p	27967	Cherp	1	0	1	1	0	3
mmu-miR-223-3p	28018	Ubfd1	1	0	1	1	0	3
mmu-miR-223-3p	28030	Gfm1	1	0	1	1	0	3
mmu-miR-223-3p	28040	D6Wsu163e	1	0	0	1	1	3
mmu-miR-223-3p	28105	Trim36	1	0	0	1	1	3
mmu-miR-223-3p	28199	Dcaf11	1	0	1	1	0	3
mmu-miR-223-3p	28250	Slco1a4	1	0	1	1	0	3
mmu-miR-223-3p	29811	Ndrg2	1	0	1	1	0	3
mmu-miR-223-3p	29812	Ndrg3	1	0	1	1	0	3
mmu-miR-223-3p	29859	Sult4a1	0	0	1	1	1	3
mmu-miR-223-3p	29877	Hdgfrp3	1	0	1	1	0	3
mmu-miR-223-3p	30800	Mmp20	1	0	1	1	0	3
mmu-miR-223-3p	30878	Apln	1	0	1	1	0	3
mmu-miR-223-3p	30925	Slamf6	0	1	1	1	0	3
mmu-miR-223-3p	30940	Usp25	1	0	1	1	0	3
mmu-miR-223-3p	30944	Zfp354c	1	0	1	1	0	3
mmu-miR-223-3p	30945	Rnf19a	1	0	1	0	1	3
mmu-miR-223-3p	50760	Fbxo17	1	0	1	1	0	3
mmu-miR-223-3p	50770	Atp11a	0	1	1	1	0	3
mmu-miR-223-3p	50791	Magi2	1	0	1	1	0	3
mmu-miR-223-3p	50798	Gne	1	1	0	1	0	3
mmu-miR-223-3p	50868	Keap1	1	0	0	1	1	3
mmu-miR-223-3p	50876	Tmod2	1	1	0	1	0	3
mmu-miR-223-3p	50934	Slc7a8	1	0	1	1	0	3
mmu-miR-223-3p	51789	Tnk2	1	0	0	1	1	3
mmu-miR-223-3p	51801	Ramp1	1	0	0	1	1	3
mmu-miR-223-3p	52132	Ccdc97	1	0	1	0	1	3
mmu-miR-223-3p	52187	Rragd	1	0	1	1	0	3
mmu-miR-223-3p	52335	Atxn1l	1	0	1	1	0	3
mmu-miR-223-3p	52348	Vps37a	1	1	0	1	0	3
mmu-miR-223-3p	52392	D1Ertd622e	1	1	0	1	0	3
mmu-miR-223-3p	52430	Echdc2	1	0	1	1	0	3
mmu-miR-223-3p	52432	Ppp2r2d	1	0	0	1	1	3
mmu-miR-223-3p	52477	Angel2	0	1	1	1	0	3
mmu-miR-223-3p	52504	Cenpo	1	0	1	1	0	3
mmu-miR-223-3p	52521	Zfp622	1	1	0	1	0	3

mmu-miR-223-3p	52696	Zwint	1	0	1	1	0	3
mmu-miR-223-3p	52838	Dnlz	1	0	1	1	0	3
mmu-miR-223-3p	52840	Dbndd2	1	0	0	1	1	3
mmu-miR-223-3p	52874	D19Bwg1357e	1	0	1	1	0	3
mmu-miR-223-3p	52892	Sco1	1	0	1	1	0	3
mmu-miR-223-3p	53325	Banp	1	0	0	1	1	3
mmu-miR-223-3p	53414	Bysl	0	1	1	1	0	3
mmu-miR-223-3p	53424	Tsnax	1	0	0	1	1	3
mmu-miR-223-3p	53599	Cd164	1	0	1	1	0	3
mmu-miR-223-3p	53611	Vti1a	0	1	1	1	0	3
mmu-miR-223-3p	53620	Vamp5	0	1	1	1	0	3
mmu-miR-223-3p	53861	Zranb2	1	0	1	1	0	3
mmu-miR-223-3p	53880	Naip7	1	0	0	1	1	3
mmu-miR-223-3p	53881	Slc5a3	1	0	1	1	0	3
mmu-miR-223-3p	53892	Ppm1d	1	1	0	1	0	3
mmu-miR-223-3p	53901	Rcan2	1	0	0	1	1	3
mmu-miR-223-3p	53951	Gpatch11	1	0	1	1	0	3
mmu-miR-223-3p	53978	Lpar2	1	0	1	1	0	3
mmu-miR-223-3p	54122	Uevld	1	0	1	1	0	3
mmu-miR-223-3p	54126	Arhgef7	0	1	1	1	0	3
mmu-miR-223-3p	54151	Cyhr1	1	0	0	1	1	3
mmu-miR-223-3p	54189	Rabep1	1	1	0	1	0	3
mmu-miR-223-3p	54199	Ccrl2	1	0	1	1	0	3
mmu-miR-223-3p	54208	Arl6ip1	1	0	1	1	0	3
mmu-miR-223-3p	54216	Pcdh7	1	0	1	1	0	3
mmu-miR-223-3p	54326	Elovl2	1	0	1	1	0	3
mmu-miR-223-3p	54366	Ctnnal1	1	0	1	1	0	3
mmu-miR-223-3p	54380	Smarcal1	1	0	0	1	1	3
mmu-miR-223-3p	54393	Gabbr1	0	1	1	1	0	3
mmu-miR-223-3p	54450	Il1f5	1	0	1	1	0	3
mmu-miR-223-3p	54486	Hpgds	1	1	0	1	0	3
mmu-miR-223-3p	54604	Pcnx	1	0	1	1	0	3
mmu-miR-223-3p	54608	Abhd2	0	1	1	1	0	3
mmu-miR-223-3p	54616	Extl3	0	1	1	1	0	3
mmu-miR-223-3p	54634	Magix	1	0	1	1	0	3
mmu-miR-223-3p	54648	Ccdc120	1	1	0	1	0	3
mmu-miR-223-3p	54670	Atp8b1	1	0	1	1	0	3
mmu-miR-223-3p	54673	Sh3glb1	1	0	1	1	0	3
mmu-miR-223-3p	55961	Slc13a1	1	0	1	1	0	3
mmu-miR-223-3p	55982	Paxip1	1	0	1	1	0	3
mmu-miR-223-3p	56013	Srcin1	1	0	1	1	0	3
mmu-miR-223-3p	56036	Ccnl2	1	0	1	1	0	3
mmu-miR-223-3p	56043	Akr1e1	1	0	1	1	0	3
mmu-miR-223-3p	56045	Samhd1	1	0	1	1	0	3
mmu-miR-223-3p	56085	Ubqln1	1	1	0	1	0	3
mmu-miR-223-3p	56175	Bace2	1	0	1	1	0	3
mmu-miR-223-3p	56176	Pigp	1	0	1	1	0	3
mmu-miR-223-3p	56177	Olfm1	1	0	0	1	1	3
mmu-miR-223-3p	56205	Ensa	1	0	1	1	0	3
mmu-miR-223-3p	56212	Rhog	0	1	1	1	0	3
mmu-miR-223-3p	56274	Stk3	1	0	1	1	0	3
mmu-miR-223-3p	56284	Mrpl19	1	0	1	1	0	3
mmu-miR-223-3p	56294	Ptpn9	1	0	1	1	0	3
mmu-miR-223-3p	56324	Stam2	1	1	0	1	0	3
mmu-miR-223-3p	56351	Ptges3	1	0	1	1	0	3

mmu-miR-223-3p	56365	Clcnkb	1	0	1	1	0	3
mmu-miR-223-3p	56381	Spen	1	1	1	0	0	3
mmu-miR-223-3p	56386	B4galt6	1	0	1	1	0	3
mmu-miR-223-3p	56399	Akap8	1	0	1	1	0	3
mmu-miR-223-3p	56430	Clip1	1	1	0	1	0	3
mmu-miR-223-3p	56438	Rbx1	1	0	1	1	0	3
mmu-miR-223-3p	56458	Foxo1	1	1	0	1	0	3
mmu-miR-223-3p	56501	Elf4	1	0	1	1	0	3
mmu-miR-223-3p	56542	Ick	1	0	0	1	1	3
mmu-miR-223-3p	56619	Clec4e	1	0	1	1	0	3
mmu-miR-223-3p	56637	Gsk3b	1	0	1	1	0	3
mmu-miR-223-3p	56706	Ccnl1	1	0	1	1	0	3
mmu-miR-223-3p	56707	Zfp111	1	0	1	1	0	3
mmu-miR-223-3p	56726	Sh3bgrl	1	0	1	1	0	3
mmu-miR-223-3p	56738	Mocs1	1	1	0	1	0	3
mmu-miR-223-3p	56741	Igdcc4	1	0	1	1	0	3
mmu-miR-223-3p	56747	Sez6l	1	0	1	1	0	3
mmu-miR-223-3p	56784	Ralgapa1	1	1	0	1	0	3
mmu-miR-223-3p	56788	Scube2	1	0	1	1	0	3
mmu-miR-223-3p	56794	Hacl1	1	0	1	1	0	3
mmu-miR-223-3p	56807	Scamp5	1	0	1	1	0	3
mmu-miR-223-3p	56809	Gmeb1	1	0	0	1	1	3
mmu-miR-223-3p	56811	Dkk2	0	1	1	1	0	3
mmu-miR-223-3p	56847	Aldh1a3	1	0	1	1	0	3
mmu-miR-223-3p	56873	Lmbr1	1	0	1	1	0	3
mmu-miR-223-3p	57138	Slc12a5	1	0	1	1	0	3
mmu-miR-223-3p	57247	Zfp276	1	0	1	1	0	3
mmu-miR-223-3p	57296	Psmd8	0	1	1	0	1	3
mmu-miR-223-3p	57321	Terf2ip	1	0	1	1	0	3
mmu-miR-223-3p	57357	Srd5a3	1	0	1	1	0	3
mmu-miR-223-3p	57440	Ehd3	1	0	1	1	0	3
mmu-miR-223-3p	57443	Fbxo3	1	0	1	1	0	3
mmu-miR-223-3p	57738	Slc15a2	0	0	1	1	1	3
mmu-miR-223-3p	57754	Cend1	0	1	1	1	0	3
mmu-miR-223-3p	57781	Cd200r1	1	0	1	1	0	3
mmu-miR-223-3p	57837	Eral1	0	1	1	1	0	3
mmu-miR-223-3p	57874	Ptplad1	1	0	1	1	0	3
mmu-miR-223-3p	57875	Angptl4	0	1	1	1	0	3
mmu-miR-223-3p	58178	Sorcs1	1	0	1	1	0	3
mmu-miR-223-3p	58180	Hic2	0	1	1	1	0	3
mmu-miR-223-3p	58217	Trem1	1	0	1	1	0	3
mmu-miR-223-3p	58227	Fam184b	1	0	0	1	1	3
mmu-miR-223-3p	58231	Stk4	1	0	1	1	0	3
mmu-miR-223-3p	58235	Pvrl1	0	1	1	1	0	3
mmu-miR-223-3p	58801	Pmaip1	1	0	1	1	0	3
mmu-miR-223-3p	58861	Cysltr1	1	0	1	1	0	3
mmu-miR-223-3p	59001	Pole3	0	1	1	1	0	3
mmu-miR-223-3p	59003	Maea	1	0	1	1	0	3
mmu-miR-223-3p	59013	Hnrnph1	0	1	1	1	0	3
mmu-miR-223-3p	59025	Usp14	1	0	0	1	1	3
mmu-miR-223-3p	59033	Slc4a8	1	0	1	1	0	3
mmu-miR-223-3p	59069	Tpm3	1	0	1	1	0	3
mmu-miR-223-3p	59079	Erbb2ip	1	1	0	1	0	3
mmu-miR-223-3p	60440	Iigp1	1	0	1	1	0	3
mmu-miR-223-3p	60533	Cd274	1	0	1	1	0	3

mmu-miR-223-3p	60599	Trp53inp1	1	0	1	1	0	3
mmu-miR-223-3p	63985	Gmfb	1	0	1	1	0	3
mmu-miR-223-3p	64009	Syne1	0	0	1	1	1	3
mmu-miR-223-3p	64085	Clstn2	1	0	1	1	0	3
mmu-miR-223-3p	64095	Gpr35	1	0	0	1	1	3
mmu-miR-223-3p	64143	Ralb	1	0	1	1	0	3
mmu-miR-223-3p	64176	Sv2b	1	0	0	1	1	3
mmu-miR-223-3p	64290	Foxb1	1	0	1	1	0	3
mmu-miR-223-3p	64378	Gpr88	1	0	1	1	0	3
mmu-miR-223-3p	64898	Lpin2	1	0	1	1	0	3
mmu-miR-223-3p	64930	Tsc1	1	0	1	1	0	3
mmu-miR-223-3p	65020	Zfp110	1	0	1	1	0	3
mmu-miR-223-3p	65102	Nif3l1	1	0	1	1	0	3
mmu-miR-223-3p	66052	Sdhc	1	0	1	1	0	3
mmu-miR-223-3p	66072	Sdhaf2	1	0	1	1	0	3
mmu-miR-223-3p	66073	Txndc12	1	0	1	0	1	3
mmu-miR-223-3p	66074	Tmem167	1	0	1	1	0	3
mmu-miR-223-3p	66075	Chchd3	1	0	1	0	1	3
mmu-miR-223-3p	66146	Tmem57	1	0	1	1	0	3
mmu-miR-223-3p	66190	Acer3	1	0	1	1	0	3
mmu-miR-223-3p	66229	Rpl7l1	1	0	1	1	0	3
mmu-miR-223-3p	66235	Eif1ax	0	1	1	1	0	3
mmu-miR-223-3p	66311	Cenpw	1	0	1	1	0	3
mmu-miR-223-3p	66314	Tpd52l2	1	0	1	0	1	3
mmu-miR-223-3p	66349	Atp5sl	0	1	1	1	0	3
mmu-miR-223-3p	66356	Knop1	1	0	1	1	0	3
mmu-miR-223-3p	66361	Zfand1	1	0	1	1	0	3
mmu-miR-223-3p	66414	Ndufa12	0	1	1	1	0	3
mmu-miR-223-3p	66459	Pyurf	1	0	1	1	0	3
mmu-miR-223-3p	66488	Fam136a	1	0	1	1	0	3
mmu-miR-223-3p	66500	Slc30a7	1	0	0	1	1	3
mmu-miR-223-3p	66510	Rnf181	1	0	1	1	0	3
mmu-miR-223-3p	66522	Pgpep1	1	0	1	1	0	3
mmu-miR-223-3p	66526	Tceanc2	1	0	1	1	0	3
mmu-miR-223-3p	66549	Aggf1	0	1	1	1	0	3
mmu-miR-223-3p	66576	Uqcrh	1	0	1	1	0	3
mmu-miR-223-3p	66585	Snrnp40	1	0	1	1	0	3
mmu-miR-223-3p	66586	Crls1	1	1	0	1	0	3
mmu-miR-223-3p	66588	Cmpk1	0	1	1	1	0	3
mmu-miR-223-3p	66589	Ube2v1	0	1	1	1	0	3
mmu-miR-223-3p	66605	1700017N19Rik	1	0	1	1	0	3
mmu-miR-223-3p	66607	Ms4a4d	1	0	1	1	0	3
mmu-miR-223-3p	66632	Dph6	1	0	1	1	0	3
mmu-miR-223-3p	66648	Tpgs2	1	0	0	1	1	3
mmu-miR-223-3p	66673	Sorcs3	1	0	1	1	0	3
mmu-miR-223-3p	66674	Spryd7	1	1	0	1	0	3
mmu-miR-223-3p	66676	Tmed7	1	1	0	1	0	3
mmu-miR-223-3p	66695	Aspn	1	0	1	1	0	3
mmu-miR-223-3p	66724	Tab3	1	1	0	1	0	3
mmu-miR-223-3p	66755	4933415F23Rik	1	0	1	1	0	3
mmu-miR-223-3p	66756	4933411K20Rik	1	0	1	1	0	3
mmu-miR-223-3p	66771	Gid4	1	0	1	1	0	3
mmu-miR-223-3p	66797	Cntnap2	1	0	0	1	1	3
mmu-miR-223-3p	66816	Thap2	1	0	1	1	0	3
mmu-miR-223-3p	66848	Fuca2	1	0	1	1	0	3

mmu-miR-223-3p	66861	Dnajc10	1	0	1	0	1	3
mmu-miR-223-3p	66864	Clec14a	1	0	1	1	0	3
mmu-miR-223-3p	66866	Nhlrc2	1	0	1	1	0	3
mmu-miR-223-3p	66870	Serbp1	1	1	0	1	0	3
mmu-miR-223-3p	66874	1200014J11Rik	1	1	0	1	0	3
mmu-miR-223-3p	66923	Pbrm1	1	0	1	1	0	3
mmu-miR-223-3p	66934	Dsn1	1	0	1	1	0	3
mmu-miR-223-3p	66962	Swsap1	1	0	0	1	1	3
mmu-miR-223-3p	66967	Edem3	1	0	1	1	0	3
mmu-miR-223-3p	66972	Slc25a23	0	1	1	1	0	3
mmu-miR-223-3p	66999	Med28	1	0	1	1	0	3
mmu-miR-223-3p	67005	Polr3k	1	0	1	1	0	3
mmu-miR-223-3p	67017	Fam210b	1	0	1	1	0	3
mmu-miR-223-3p	67115	Rpl14	1	0	1	1	0	3
mmu-miR-223-3p	67121	Mastl	1	1	0	0	1	3
mmu-miR-223-3p	67154	Mtdh	1	0	1	1	0	3
mmu-miR-223-3p	67164	Lipt2	0	1	1	1	0	3
mmu-miR-223-3p	67171	Dram2	1	1	0	1	0	3
mmu-miR-223-3p	67181	Ctdnep1	1	0	1	1	0	3
mmu-miR-223-3p	67198	Spats2l	1	1	0	1	0	3
mmu-miR-223-3p	67204	Eif2s2	1	0	1	1	0	3
mmu-miR-223-3p	67207	Lsm1	1	0	1	1	0	3
mmu-miR-223-3p	67213	Cmtm6	1	0	1	1	0	3
mmu-miR-223-3p	67245	Peli1	1	0	1	1	0	3
mmu-miR-223-3p	67283	Slc25a19	1	0	1	1	0	3
mmu-miR-223-3p	67379	Dedd2	1	0	1	1	0	3
mmu-miR-223-3p	67433	Ccdc127	1	0	0	1	1	3
mmu-miR-223-3p	67434	Ankrd33b	1	0	1	0	1	3
mmu-miR-223-3p	67453	Slc25a46	0	1	1	1	0	3
mmu-miR-223-3p	67456	Ergic2	1	0	1	1	0	3
mmu-miR-223-3p	67460	Decr1	0	1	1	1	0	3
mmu-miR-223-3p	67468	Mmd	1	1	1	0	0	3
mmu-miR-223-3p	67472	Mtfr1	1	0	1	1	0	3
mmu-miR-223-3p	67474	Snap29	0	1	1	1	0	3
mmu-miR-223-3p	67480	Cwc25	0	1	1	1	0	3
mmu-miR-223-3p	67490	Ufl1	1	0	0	1	1	3
mmu-miR-223-3p	67498	Kcnv1	1	0	1	1	0	3
mmu-miR-223-3p	67501	Ccdc50	1	0	0	1	1	3
mmu-miR-223-3p	67513	2610002J02Rik	1	0	0	1	1	3
mmu-miR-223-3p	67532	Mfap1a	1	0	1	1	0	3
mmu-miR-223-3p	67554	Slc25a30	1	0	1	1	0	3
mmu-miR-223-3p	67581	Tbc1d23	1	0	1	1	0	3
mmu-miR-223-3p	67582	Slc25a26	1	0	1	1	0	3
mmu-miR-223-3p	67607	Zfp788	0	1	1	1	0	3
mmu-miR-223-3p	67615	Ube2r2	1	0	1	1	0	3
mmu-miR-223-3p	67630	Samd8	1	0	1	1	0	3
mmu-miR-223-3p	67665	Dctn4	0	1	1	1	0	3
mmu-miR-223-3p	67693	Hypk	0	1	1	1	0	3
mmu-miR-223-3p	67698	Fam174a	1	0	1	1	0	3
mmu-miR-223-3p	67702	Rnf149	1	0	0	1	1	3
mmu-miR-223-3p	67719	2310057J18Rik	1	0	1	1	0	3
mmu-miR-223-3p	67769	Gpatch2	1	0	1	1	0	3
mmu-miR-223-3p	67792	Rgs8	0	1	1	1	0	3
mmu-miR-223-3p	67803	Limd2	1	0	1	1	0	3
mmu-miR-223-3p	67812	Ubxn4	1	0	1	1	0	3

mmu-miR-223-3p	67834	Idh3a	1	0	1	1	0	3
mmu-miR-223-3p	67840	Mrp63	1	0	1	1	0	3
mmu-miR-223-3p	67864	Yipf4	1	1	0	1	0	3
mmu-miR-223-3p	67878	Tmem33	0	1	1	1	0	3
mmu-miR-223-3p	67881	Mdp1	1	0	1	1	0	3
mmu-miR-223-3p	67897	Rnmt	1	0	1	1	0	3
mmu-miR-223-3p	67912	1600012H06Rik	1	1	0	1	0	3
mmu-miR-223-3p	67946	Spata6	1	0	1	1	0	3
mmu-miR-223-3p	67974	Ccny	1	0	1	1	0	3
mmu-miR-223-3p	67985	Ssxb1	1	0	0	1	1	3
mmu-miR-223-3p	67988	Tmx3	1	0	1	1	0	3
mmu-miR-223-3p	68034	Fam122a	1	0	1	1	0	3
mmu-miR-223-3p	68050	Akirin1	1	0	1	1	0	3
mmu-miR-223-3p	68070	Pdzd2	1	0	1	1	0	3
mmu-miR-223-3p	68089	Arpc4	1	0	0	1	1	3
mmu-miR-223-3p	68145	Etaa1	1	0	1	1	0	3
mmu-miR-223-3p	68151	Wls	1	0	1	1	0	3
mmu-miR-223-3p	68152	Fam133b	1	0	1	1	0	3
mmu-miR-223-3p	68177	Ebpl	1	0	1	1	0	3
mmu-miR-223-3p	68178	Cgnl1	1	0	1	1	0	3
mmu-miR-223-3p	68196	Hsbp1	0	1	1	1	0	3
mmu-miR-223-3p	68229	AI846148	1	0	0	1	1	3
mmu-miR-223-3p	68255	Tmem86b	1	0	1	1	0	3
mmu-miR-223-3p	68259	Ift80	1	0	1	1	0	3
mmu-miR-223-3p	68260	Trmt12	0	1	1	1	0	3
mmu-miR-223-3p	68292	Stt3b	1	0	1	1	0	3
mmu-miR-223-3p	68318	Aph1c	1	0	1	1	0	3
mmu-miR-223-3p	68338	Golt1a	0	1	1	1	0	3
mmu-miR-223-3p	68364	0610030E20Rik	1	0	0	1	1	3
mmu-miR-223-3p	68365	Rab14	1	1	0	1	0	3
mmu-miR-223-3p	68465	Adipor2	1	0	1	1	0	3
mmu-miR-223-3p	68524	Wipf2	1	0	1	1	0	3
mmu-miR-223-3p	68549	Sgol2	0	1	1	1	0	3
mmu-miR-223-3p	68581	Tmed10	1	0	1	1	0	3
mmu-miR-223-3p	68585	Rtn4	1	0	0	1	1	3
mmu-miR-223-3p	68597	Ccdc167	1	0	0	1	1	3
mmu-miR-223-3p	68734	Smek1	1	0	1	1	0	3
mmu-miR-223-3p	68750	Rreb1	1	0	0	1	1	3
mmu-miR-223-3p	68770	Phtf2	0	1	1	1	0	3
mmu-miR-223-3p	68817	Ddi2	1	1	0	1	0	3
mmu-miR-223-3p	68832	1110057K04Rik	0	1	1	1	0	3
mmu-miR-223-3p	68859	Smim1	1	0	0	1	1	3
mmu-miR-223-3p	68889	Ubac2	1	0	1	1	0	3
mmu-miR-223-3p	68926	Ubap2	1	0	1	1	0	3
mmu-miR-223-3p	68942	Chmp2b	1	0	1	1	0	3
mmu-miR-223-3p	68944	Tmco1	1	0	1	1	0	3
mmu-miR-223-3p	68955	Srrm4	1	0	1	1	0	3
mmu-miR-223-3p	68999	Anapc10	1	1	0	1	0	3
mmu-miR-223-3p	69069	1810011H11Rik	1	0	0	1	1	3
mmu-miR-223-3p	69104	March5	1	0	0	1	1	3
mmu-miR-223-3p	69179	Tmem110	0	1	1	1	0	3
mmu-miR-223-3p	69256	Zfp397	1	0	1	1	0	3
mmu-miR-223-3p	69314	Izumo3	0	1	0	1	1	3
mmu-miR-223-3p	69315	1700001L19Rik	1	1	0	1	0	3
mmu-miR-223-3p	69352	Necab1	1	0	1	1	0	3

mmu-miR-223-3p	69371	Smco2	1	0	0	1	1	3
mmu-miR-223-3p	69499	Tsr2	1	0	1	1	0	3
mmu-miR-223-3p	69605	Lnp	1	0	0	1	1	3
mmu-miR-223-3p	69608	Sec24d	1	0	1	1	0	3
mmu-miR-223-3p	69632	Arhgef12	1	0	1	1	0	3
mmu-miR-223-3p	69640	Fam83g	1	0	1	1	0	3
mmu-miR-223-3p	69690	2310057B04Rik	1	0	0	1	1	3
mmu-miR-223-3p	69717	Gm10499	1	0	0	1	1	3
mmu-miR-223-3p	69721	Nkiras1	1	0	1	1	0	3
mmu-miR-223-3p	69726	Smyd3	1	0	1	1	0	3
mmu-miR-223-3p	69773	1810026J23Rik	1	0	1	1	0	3
mmu-miR-223-3p	69780	Smap2	1	0	1	1	0	3
mmu-miR-223-3p	69806	Slc39a11	1	0	1	1	0	3
mmu-miR-223-3p	69821	Mterfd2	0	1	1	1	0	3
mmu-miR-223-3p	69834	Rab43	1	0	0	1	1	3
mmu-miR-223-3p	69852	Tcf23	0	1	1	1	0	3
mmu-miR-223-3p	69861	2010003K11Rik	1	0	1	1	0	3
mmu-miR-223-3p	69908	Rab3b	1	0	1	1	0	3
mmu-miR-223-3p	69976	Galk2	1	0	1	1	0	3
mmu-miR-223-3p	70097	Sash1	1	0	1	1	0	3
mmu-miR-223-3p	70227	Zfp619	0	1	1	1	0	3
mmu-miR-223-3p	70233	Cd2bp2	1	0	1	1	0	3
mmu-miR-223-3p	70296	Tbc1d13	0	1	1	1	0	3
mmu-miR-223-3p	70317	Arl16	1	0	1	1	0	3
mmu-miR-223-3p	70408	Polr3f	1	0	1	1	0	3
mmu-miR-223-3p	70445	Cd248	0	1	1	1	0	3
mmu-miR-223-3p	70451	Dhrs13	0	1	1	1	0	3
mmu-miR-223-3p	70454	Cenpl	1	0	1	1	0	3
mmu-miR-223-3p	70461	Crtc3	1	0	1	1	0	3
mmu-miR-223-3p	70470	Rprd1b	0	1	1	1	0	3
mmu-miR-223-3p	70508	Bbx	1	1	0	1	0	3
mmu-miR-223-3p	70549	Tln2	0	1	1	1	0	3
mmu-miR-223-3p	70591	5730455P16Rik	0	1	1	1	0	3
mmu-miR-223-3p	70617	5730508B09Rik	1	0	1	1	0	3
mmu-miR-223-3p	70699	Nup205	1	0	0	1	1	3
mmu-miR-223-3p	70701	Nipal1	1	0	1	1	0	3
mmu-miR-223-3p	70771	Gpr173	1	0	1	1	0	3
mmu-miR-223-3p	70797	Ankib1	1	1	0	1	0	3
mmu-miR-223-3p	70802	Pwwp2a	1	0	0	1	1	3
mmu-miR-223-3p	70823	Hmgxb4	0	1	1	1	0	3
mmu-miR-223-3p	70918	Nsun7	0	1	1	1	0	3
mmu-miR-223-3p	70925	Cdkn2aip	1	0	1	1	0	3
mmu-miR-223-3p	70952	Poteg	1	0	1	1	0	3
mmu-miR-223-3p	71030	4933403O08Rik	1	0	0	1	1	3
mmu-miR-223-3p	71063	Zfp597	1	1	0	1	0	3
mmu-miR-223-3p	71101	Uvssa	1	0	1	1	0	3
mmu-miR-223-3p	71132	Cabyr	1	0	1	1	0	3
mmu-miR-223-3p	71146	Golga7b	1	0	1	1	0	3
mmu-miR-223-3p	71151	Eri2	0	1	1	0	1	3
mmu-miR-223-3p	71207	Nudt4	1	0	1	1	0	3
mmu-miR-223-3p	71279	Slc29a3	1	0	1	1	0	3
mmu-miR-223-3p	71330	Rcbtb1	1	0	1	1	0	3
mmu-miR-223-3p	71371	Arid5b	0	1	1	1	0	3
mmu-miR-223-3p	71520	Grap	1	0	1	1	0	3
mmu-miR-223-3p	71602	Myo1e	1	0	0	1	1	3

mmu-miR-223-3p	71693	Colec11	1	0	1	1	0	3
mmu-miR-223-3p	71756	Cpn2	1	0	1	1	0	3
mmu-miR-223-3p	71770	Ap2b1	1	1	0	1	0	3
mmu-miR-223-3p	71772	Plbd2	1	0	1	0	1	3
mmu-miR-223-3p	71777	Ing3	1	0	1	1	0	3
mmu-miR-223-3p	71801	Plekhf2	1	0	1	1	0	3
mmu-miR-223-3p	71804	Mtfr2	1	0	1	1	0	3
mmu-miR-223-3p	71819	Kif23	1	0	0	1	1	3
mmu-miR-223-3p	71837	1700003E16Rik	1	0	0	1	1	3
mmu-miR-223-3p	71889	Epn3	0	1	1	1	0	3
mmu-miR-223-3p	71891	Cdadc1	1	0	0	1	1	3
mmu-miR-223-3p	71924	Tube1	1	0	1	1	0	3
mmu-miR-223-3p	71955	Ist1	1	0	1	1	0	3
mmu-miR-223-3p	71956	Rnf135	1	0	1	1	0	3
mmu-miR-223-3p	71974	Prmt3	1	1	0	1	0	3
mmu-miR-223-3p	71988	Esco2	1	0	1	1	0	3
mmu-miR-223-3p	71991	Ercc8	1	1	0	1	0	3
mmu-miR-223-3p	71995	Erv3	1	1	0	1	0	3
mmu-miR-223-3p	71999	Fbxo22	0	1	1	1	0	3
mmu-miR-223-3p	72007	Fndc3b	0	1	1	1	0	3
mmu-miR-223-3p	72020	Zfp654	1	0	1	1	0	3
mmu-miR-223-3p	72022	Slc35f2	1	1	1	0	0	3
mmu-miR-223-3p	72033	Tsc22d2	1	0	1	1	0	3
mmu-miR-223-3p	72065	Rap2c	1	0	1	1	0	3
mmu-miR-223-3p	72085	Osgepl1	0	1	1	1	0	3
mmu-miR-223-3p	72106	Jmjd8	1	1	0	1	0	3
mmu-miR-223-3p	72124	Seh1l	0	1	1	1	0	3
mmu-miR-223-3p	72154	Zfp157	1	0	1	1	0	3
mmu-miR-223-3p	72181	Nsun4	1	0	0	1	1	3
mmu-miR-223-3p	72185	Dbndd1	1	1	0	1	0	3
mmu-miR-223-3p	72190	2510009E07Rik	0	1	1	1	0	3
mmu-miR-223-3p	72201	Otud6b	1	0	1	1	0	3
mmu-miR-223-3p	72244	1600014C10Rik	1	0	0	1	1	3
mmu-miR-223-3p	72258	Kcnk10	1	0	1	1	0	3
mmu-miR-223-3p	72287	Plekhf1	1	0	1	1	0	3
mmu-miR-223-3p	72313	Fryl	1	0	1	1	0	3
mmu-miR-223-3p	72345	Amer1	1	0	1	1	0	3
mmu-miR-223-3p	72349	Dusp3	1	0	0	1	1	3
mmu-miR-223-3p	72350	Zc2hc1c	1	0	1	1	0	3
mmu-miR-223-3p	72461	Prcp	1	0	1	1	0	3
mmu-miR-223-3p	72462	Rrp1b	1	0	0	1	1	3
mmu-miR-223-3p	72472	Slc16a10	1	0	1	1	0	3
mmu-miR-223-3p	72480	Tspyl4	1	0	1	1	0	3
mmu-miR-223-3p	72514	Fgfbp3	1	0	1	1	0	3
mmu-miR-223-3p	72560	Naalad2	1	0	1	1	0	3
mmu-miR-223-3p	72584	Cul4b	1	0	0	1	1	3
mmu-miR-223-3p	72635	Lins	1	0	0	1	1	3
mmu-miR-223-3p	72650	2810006K23Rik	1	0	1	1	0	3
mmu-miR-223-3p	72745	Tmem161b	1	0	1	1	0	3
mmu-miR-223-3p	72776	Sass6	1	0	1	1	0	3
mmu-miR-223-3p	72792	2810459M11Rik	1	1	0	1	0	3
mmu-miR-223-3p	72821	Scn2b	1	0	1	1	0	3
mmu-miR-223-3p	72823	Pard3b	1	0	1	1	0	3
mmu-miR-223-3p	72865	Cxx1c	1	0	1	0	1	3
mmu-miR-223-3p	73122	Tgfbrap1	1	0	1	1	0	3

mmu-miR-223-3p	73242	Atat1	1	0	1	1	0	3
mmu-miR-223-3p	73251	Setd7	1	1	0	1	0	3
mmu-miR-223-3p	73338	Itpripl1	1	1	0	1	0	3
mmu-miR-223-3p	73442	Hspa12a	1	0	1	1	0	3
mmu-miR-223-3p	73469	Rnf38	1	0	0	1	1	3
mmu-miR-223-3p	73680	Zbtb8a	0	1	1	1	0	3
mmu-miR-223-3p	73699	Ppp2r1b	1	0	1	1	0	3
mmu-miR-223-3p	73720	Cst6	1	0	1	1	0	3
mmu-miR-223-3p	73827	Tmem198b	0	1	1	1	0	3
mmu-miR-223-3p	74039	Nfam1	1	0	1	1	0	3
mmu-miR-223-3p	74043	Pex26	1	1	0	1	0	3
mmu-miR-223-3p	74100	Arpp21	1	0	0	1	1	3
mmu-miR-223-3p	74105	Gga2	1	0	1	1	0	3
mmu-miR-223-3p	74117	Actr3	0	1	1	1	0	3
mmu-miR-223-3p	74123	Foxp4	1	0	0	1	1	3
mmu-miR-223-3p	74136	Sec14l1	1	1	0	1	0	3
mmu-miR-223-3p	74153	Uba7	1	1	0	1	0	3
mmu-miR-223-3p	74154	Unkl	1	0	1	1	0	3
mmu-miR-223-3p	74158	Josd1	1	0	1	1	0	3
mmu-miR-223-3p	74164	Nfx1	1	0	1	0	1	3
mmu-miR-223-3p	74186	Ccdc3	1	0	1	1	0	3
mmu-miR-223-3p	74187	Katnb1	0	1	1	1	0	3
mmu-miR-223-3p	74189	Phactr3	1	0	1	1	0	3
mmu-miR-223-3p	74200	2810403A07Rik	1	0	1	1	0	3
mmu-miR-223-3p	74246	Gale	0	1	1	1	0	3
mmu-miR-223-3p	74249	Lrrc2	1	0	1	1	0	3
mmu-miR-223-3p	74286	Tbc1d21	1	0	0	1	1	3
mmu-miR-223-3p	74334	Ranbp10	1	0	1	1	0	3
mmu-miR-223-3p	74352	Zfp84	1	0	1	1	0	3
mmu-miR-223-3p	74388	Dpp8	1	0	1	1	0	3
mmu-miR-223-3p	74411	Ppapdc2	1	0	1	1	0	3
mmu-miR-223-3p	74435	Lrriq3	0	1	1	1	0	3
mmu-miR-223-3p	74450	Pank2	1	0	1	1	0	3
mmu-miR-223-3p	74463	Exoc3l2	1	0	0	1	1	3
mmu-miR-223-3p	74464	Zswim5	0	1	1	1	0	3
mmu-miR-223-3p	74488	Lrrc15	1	0	1	1	0	3
mmu-miR-223-3p	74513	Neto2	1	0	1	1	0	3
mmu-miR-223-3p	74580	Pyroxd2	1	0	1	1	0	3
mmu-miR-223-3p	74645	Fam46c	1	1	0	1	0	3
mmu-miR-223-3p	74694	Tbc1d30	0	1	0	1	1	3
mmu-miR-223-3p	74778	Rrp7a	1	0	1	1	0	3
mmu-miR-223-3p	74781	Wipi2	0	1	1	1	0	3
mmu-miR-223-3p	74963	4930470F04Rik	1	0	0	1	1	3
mmu-miR-223-3p	74998	Rab11fip2	1	0	1	1	0	3
mmu-miR-223-3p	75173	Tex38	1	0	0	1	1	3
mmu-miR-223-3p	75302	Asxl2	1	0	1	1	0	3
mmu-miR-223-3p	75320	Etnk1	1	1	0	1	0	3
mmu-miR-223-3p	75415	Arhgap12	1	0	0	1	1	3
mmu-miR-223-3p	75426	Igfbpl1	1	0	1	1	0	3
mmu-miR-223-3p	75472	1700009P17Rik	0	1	1	1	0	3
mmu-miR-223-3p	75590	Dusp9	1	1	0	1	0	3
mmu-miR-223-3p	75605	Kdm5b	1	0	1	1	0	3
mmu-miR-223-3p	75646	Rai14	0	1	1	1	0	3
mmu-miR-223-3p	75686	Nudt16	1	1	0	1	0	3
mmu-miR-223-3p	75695	Rilpl1	1	0	1	1	0	3

mmu-miR-223-3p	75705	Eif4b	0	1	1	1	0	3
mmu-miR-223-3p	75717	Cul5	1	0	0	1	1	3
mmu-miR-223-3p	75723	Amotl1	1	1	0	1	0	3
mmu-miR-223-3p	75753	Klf17	1	0	1	1	0	3
mmu-miR-223-3p	75767	Rab11fip1	1	0	1	1	0	3
mmu-miR-223-3p	75782	Lca5	1	0	1	1	0	3
mmu-miR-223-3p	75826	Senp2	1	0	0	1	1	3
mmu-miR-223-3p	75901	Dcp1a	1	1	0	1	0	3
mmu-miR-223-3p	75965	Zdhhc20	1	0	1	1	0	3
mmu-miR-223-3p	75991	Slain2	1	0	0	1	1	3
mmu-miR-223-3p	76055	Mgea5	1	0	1	1	0	3
mmu-miR-223-3p	76073	Pcgf5	0	1	1	1	0	3
mmu-miR-223-3p	76080	Ttpal	1	0	0	1	1	3
mmu-miR-223-3p	76088	Dock8	1	0	1	0	1	3
mmu-miR-223-3p	76117	Arhgap15	1	1	0	1	0	3
mmu-miR-223-3p	76251	Ercc6l2	1	0	1	1	0	3
mmu-miR-223-3p	76295	Atp11b	0	1	1	1	0	3
mmu-miR-223-3p	76413	1700016D06Rik	1	0	1	1	0	3
mmu-miR-223-3p	76429	Lhpp	1	0	1	1	0	3
mmu-miR-223-3p	76467	Msrb2	0	1	1	1	0	3
mmu-miR-223-3p	76497	Ppp1r11	0	1	1	1	0	3
mmu-miR-223-3p	76509	1600029D21Rik	1	0	1	1	0	3
mmu-miR-223-3p	76510	Trappc9	1	1	0	1	0	3
mmu-miR-223-3p	76551	Ccdc6	1	0	1	1	0	3
mmu-miR-223-3p	76577	Faf2	0	1	1	1	0	3
mmu-miR-223-3p	76594	Dnajc18	1	0	1	1	0	3
mmu-miR-223-3p	76608	Hectd3	1	0	1	1	0	3
mmu-miR-223-3p	76650	Srxn1	1	0	1	1	0	3
mmu-miR-223-3p	76719	Kansl1	1	0	1	1	0	3
mmu-miR-223-3p	76740	Efr3a	0	1	1	1	0	3
mmu-miR-223-3p	76775	Slc10a7	1	0	1	1	0	3
mmu-miR-223-3p	76788	Klhdc10	1	1	0	1	0	3
mmu-miR-223-3p	76809	Bri3bp	1	0	1	1	0	3
mmu-miR-223-3p	76824	Mtfr1l	1	1	0	1	0	3
mmu-miR-223-3p	76843	Dtl	1	0	1	1	0	3
mmu-miR-223-3p	76850	Ago4	1	0	0	1	1	3
mmu-miR-223-3p	76892	Rnft1	1	1	0	1	0	3
mmu-miR-223-3p	76897	Ralyl	1	0	1	1	0	3
mmu-miR-223-3p	76916	Timmdc1	1	0	1	1	0	3
mmu-miR-223-3p	76943	Psapl1	1	0	1	1	0	3
mmu-miR-223-3p	76982	3110035E14Rik	1	0	1	1	0	3
mmu-miR-223-3p	77035	Kdm8	1	0	1	1	0	3
mmu-miR-223-3p	77041	Arsk	1	0	1	1	0	3
mmu-miR-223-3p	77045	Bcl7a	0	1	1	1	0	3
mmu-miR-223-3p	77053	Sun1	0	1	1	1	0	3
mmu-miR-223-3p	77097	Tanc2	1	0	1	1	0	3
mmu-miR-223-3p	77125	Il33	1	0	1	1	0	3
mmu-miR-223-3p	77318	Ankrd55	1	0	0	1	1	3
mmu-miR-223-3p	77519	Zfp266	1	0	0	1	1	3
mmu-miR-223-3p	77521	Mtus2	0	1	0	1	1	3
mmu-miR-223-3p	77559	Agl	1	0	1	1	0	3
mmu-miR-223-3p	77590	Chst15	1	0	1	1	0	3
mmu-miR-223-3p	77593	Usp45	1	0	1	1	0	3
mmu-miR-223-3p	77629	Sphkap	1	0	1	1	0	3
mmu-miR-223-3p	77644	C330007P06Rik	0	1	1	1	0	3

mmu-miR-223-3p	77697	Mmab	1	0	1	1	0	3
mmu-miR-223-3p	77704	Lcn9	1	0	1	1	0	3
mmu-miR-223-3p	77777	Ulbp1	1	1	0	1	0	3
mmu-miR-223-3p	77809	Lrrc42	1	0	0	1	1	3
mmu-miR-223-3p	77853	Msl2	1	1	0	1	0	3
mmu-miR-223-3p	77864	Ypel2	1	0	1	1	0	3
mmu-miR-223-3p	78088	Sowahb	1	0	1	1	0	3
mmu-miR-223-3p	78339	Ttyh3	1	0	1	1	0	3
mmu-miR-223-3p	78581	Utp23	1	0	1	1	0	3
mmu-miR-223-3p	78655	Eif3j1	1	1	0	1	0	3
mmu-miR-223-3p	78688	Nol3	1	0	1	1	0	3
mmu-miR-223-3p	78749	Filip1l	1	1	0	1	0	3
mmu-miR-223-3p	78755	Fam122b	1	0	1	1	0	3
mmu-miR-223-3p	78771	Mctp1	0	1	1	1	0	3
mmu-miR-223-3p	78808	Stxbp5	1	0	1	1	0	3
mmu-miR-223-3p	78894	Aacs	1	0	1	1	0	3
mmu-miR-223-3p	78908	Igsf3	0	1	1	1	0	3
mmu-miR-223-3p	78921	9130019O22Rik	1	0	1	1	0	3
mmu-miR-223-3p	78926	Gas2l1	1	0	1	1	0	3
mmu-miR-223-3p	79202	Tnfrsf22	1	1	1	0	0	3
mmu-miR-223-3p	79263	Trim39	0	1	1	1	0	3
mmu-miR-223-3p	79264	Krit1	1	0	0	1	1	3
mmu-miR-223-3p	80889	Mesdc1	1	0	1	1	0	3
mmu-miR-223-3p	80902	Zfp202	0	1	1	1	0	3
mmu-miR-223-3p	80912	Pum1	1	0	1	1	0	3
mmu-miR-223-3p	80913	Pum2	1	0	1	1	0	3
mmu-miR-223-3p	80915	Dusp12	0	1	1	1	0	3
mmu-miR-223-3p	81000	Rad54l2	1	0	1	1	0	3
mmu-miR-223-3p	81004	Tbl1xr1	1	0	1	1	0	3
mmu-miR-223-3p	83383	Tfap4	0	1	1	1	0	3
mmu-miR-223-3p	83397	Akap12	1	0	1	1	0	3
mmu-miR-223-3p	83456	Mov10l1	0	1	1	1	0	3
mmu-miR-223-3p	83493	Sacm1l	0	1	1	1	0	3
mmu-miR-223-3p	83565	Pramel3	0	1	1	1	0	3
mmu-miR-223-3p	83602	Gtf2a1	1	0	0	1	1	3
mmu-miR-223-3p	83674	Cnnm1	0	1	1	1	0	3
mmu-miR-223-3p	83771	Tas1r3	0	1	1	1	0	3
mmu-miR-223-3p	83814	Nedd4l	1	0	0	1	1	3
mmu-miR-223-3p	83984	Tssk6	1	0	1	1	0	3
mmu-miR-223-3p	83997	Slmap	1	0	1	1	0	3
mmu-miR-223-3p	89867	Sec16b	1	0	1	1	0	3
mmu-miR-223-3p	93670	Tac4	0	1	1	1	0	3
mmu-miR-223-3p	93683	Glce	1	0	1	1	0	3
mmu-miR-223-3p	93697	Narg2	1	0	1	1	0	3
mmu-miR-223-3p	93703	Pcdhgb6	1	1	0	1	0	3
mmu-miR-223-3p	93706	Pcdhgc3	1	1	0	1	0	3
mmu-miR-223-3p	93707	Pcdhgc4	1	1	0	1	0	3
mmu-miR-223-3p	93708	Pcdhgc5	1	1	0	1	0	3
mmu-miR-223-3p	93711	Pcdhga3	1	1	0	1	0	3
mmu-miR-223-3p	93716	Pcdhga8	1	1	0	1	0	3
mmu-miR-223-3p	93717	Pcdhga9	1	1	0	1	0	3
mmu-miR-223-3p	93723	Pcdhga11	1	1	0	1	0	3
mmu-miR-223-3p	93724	Pcdhga12	1	1	0	1	0	3
mmu-miR-223-3p	93834	Peli2	1	0	1	1	0	3
mmu-miR-223-3p	93836	Rnf111	0	1	1	1	0	3

mmu-miR-223-3p	93842	Igsf9	0	1	1	1	0	3
mmu-miR-223-3p	93843	Pnck	1	0	1	1	0	3
mmu-miR-223-3p	93887	Pcdhb16	1	0	1	1	0	3
mmu-miR-223-3p	93889	Pcdhb18	1	0	1	1	0	3
mmu-miR-223-3p	94044	Bcl2l13	1	0	1	1	0	3
mmu-miR-223-3p	94047	Cecr6	0	1	1	1	0	3
mmu-miR-223-3p	94061	Mrpl1	1	0	1	1	0	3
mmu-miR-223-3p	94090	Trim9	1	0	0	1	1	3
mmu-miR-223-3p	94094	Trim34a	1	0	1	0	1	3
mmu-miR-223-3p	94191	Adarb2	1	0	1	1	0	3
mmu-miR-223-3p	94221	Gopc	1	0	1	1	0	3
mmu-miR-223-3p	94246	Arid4b	1	1	0	1	0	3
mmu-miR-223-3p	94282	Sfxn5	0	1	1	1	0	3
mmu-miR-223-3p	94332	Cadm3	0	1	1	1	0	3
mmu-miR-223-3p	94352	Loxl2	1	0	1	1	0	3
mmu-miR-223-3p	97243	Naa11	0	1	1	1	0	3
mmu-miR-223-3p	98314	D2hgdh	1	0	1	1	0	3
mmu-miR-223-3p	98404	AI597479	1	0	1	1	0	3
mmu-miR-223-3p	98415	Nucks1	1	1	0	1	0	3
mmu-miR-223-3p	98432	Phlpp1	1	1	0	0	1	3
mmu-miR-223-3p	98682	Mfsd6	1	0	1	1	0	3
mmu-miR-223-3p	98741	Kcnb2	1	1	0	1	0	3
mmu-miR-223-3p	98999	Znfx1	0	1	1	1	0	3
mmu-miR-223-3p	99031	Osbpl6	1	0	1	1	0	3
mmu-miR-223-3p	99470	Magi3	1	1	0	1	0	3
mmu-miR-223-3p	99480	Dnttip2	0	1	1	0	1	3
mmu-miR-223-3p	99633	Lphn2	0	1	1	1	0	3
mmu-miR-223-3p	99712	Cept1	1	1	0	1	0	3
mmu-miR-223-3p	99889	Arfip1	0	1	1	0	1	3
mmu-miR-223-3p	100019	Mdn1	1	0	1	1	0	3
mmu-miR-223-3p	100342	Fam46b	1	0	1	1	0	3
mmu-miR-223-3p	100515	Zfp518b	1	0	1	1	0	3
mmu-miR-223-3p	100532	Rell1	1	0	1	1	0	3
mmu-miR-223-3p	100637	N4bp2l1	1	1	0	1	0	3
mmu-miR-223-3p	100727	Ugt2b34	1	0	0	1	1	3
mmu-miR-223-3p	100732	Mapre3	0	1	1	1	0	3
mmu-miR-223-3p	100737	Dcun1d4	1	0	1	1	0	3
mmu-miR-223-3p	100763	Ube3c	1	0	1	1	0	3
mmu-miR-223-3p	100978	Nfxl1	1	0	1	1	0	3
mmu-miR-223-3p	101187	Parp11	1	0	1	1	0	3
mmu-miR-223-3p	101401	Adamts9	1	0	1	1	0	3
mmu-miR-223-3p	101602	AI467606	1	0	1	1	0	3
mmu-miR-223-3p	101612	Grwd1	0	1	1	1	0	3
mmu-miR-223-3p	101867	Rrp8	1	0	1	1	0	3
mmu-miR-223-3p	102058	Exoc8	1	0	1	1	0	3
mmu-miR-223-3p	102098	Arhgef18	1	0	1	1	0	3
mmu-miR-223-3p	102103	Mtus1	1	1	0	1	0	3
mmu-miR-223-3p	102278	Cpne7	1	0	1	1	0	3
mmu-miR-223-3p	102323	Dcun1d2	1	0	0	1	1	3
mmu-miR-223-3p	102442	Dennd4a	0	1	0	1	1	3
mmu-miR-223-3p	103080	Sept10	0	1	1	1	0	3
mmu-miR-223-3p	103806	Maml1	1	0	1	1	0	3
mmu-miR-223-3p	103967	Dnm3	0	1	1	1	0	3
mmu-miR-223-3p	104156	Etv5	1	0	1	1	0	3
mmu-miR-223-3p	104175	Sbk1	1	0	1	1	0	3

mmu-miR-223-3p	104394	E2f4	1	0	0	1	1	3
mmu-miR-223-3p	104886	Rab15	0	1	1	1	0	3
mmu-miR-223-3p	105005	Fam84a	0	1	1	1	0	3
mmu-miR-223-3p	105148	Iars	0	1	1	1	0	3
mmu-miR-223-3p	105377	Ankrd32	1	0	1	0	1	3
mmu-miR-223-3p	105440	Kctd9	1	1	0	1	0	3
mmu-miR-223-3p	105522	Ankrd28	1	1	0	1	0	3
mmu-miR-223-3p	105722	Ano6	1	0	1	1	0	3
mmu-miR-223-3p	105837	Mtbp	1	1	0	1	0	3
mmu-miR-223-3p	106014	Fam19a5	1	0	1	1	0	3
mmu-miR-223-3p	106052	Fbxo4	1	0	0	1	1	3
mmu-miR-223-3p	106064	AW549877	1	0	1	1	0	3
mmu-miR-223-3p	106143	Cggbp1	1	1	0	1	0	3
mmu-miR-223-3p	106572	Rab31	1	0	1	1	0	3
mmu-miR-223-3p	106639	Vmac	0	1	1	1	0	3
mmu-miR-223-3p	106840	Unc119b	1	0	1	1	0	3
mmu-miR-223-3p	106894	Hmgxb3	0	1	0	1	1	3
mmu-miR-223-3p	107065	Lrrtm2	1	0	1	1	0	3
mmu-miR-223-3p	107227	Macrod1	0	1	1	1	0	3
mmu-miR-223-3p	107271	Yars	0	1	1	1	0	3
mmu-miR-223-3p	107587	Osr2	0	1	1	1	0	3
mmu-miR-223-3p	107589	Mylk	1	0	1	1	0	3
mmu-miR-223-3p	107723	Slc12a6	1	0	0	1	1	3
mmu-miR-223-3p	107767	Scamp1	1	0	1	1	0	3
mmu-miR-223-3p	107815	Scml2	0	1	1	1	0	3
mmu-miR-223-3p	107951	Cdk9	1	0	1	1	0	3
mmu-miR-223-3p	108015	Chrnb4	1	0	1	1	0	3
mmu-miR-223-3p	108052	Slc14a1	1	0	1	1	0	3
mmu-miR-223-3p	108097	Prkab2	0	1	1	1	0	3
mmu-miR-223-3p	108154	Adamts6	0	1	1	1	0	3
mmu-miR-223-3p	108653	Rimklb	1	0	1	1	0	3
mmu-miR-223-3p	108670	Epsti1	0	1	1	1	0	3
mmu-miR-223-3p	108682	Gpt2	1	0	1	1	0	3
mmu-miR-223-3p	108686	Ccdc88a	1	0	1	1	0	3
mmu-miR-223-3p	108899	2700081O15Rik	1	0	1	1	0	3
mmu-miR-223-3p	109006	Ciapin1	1	0	1	1	0	3
mmu-miR-223-3p	109050	Fam212b	1	0	1	1	0	3
mmu-miR-223-3p	109136	Mmaa	1	0	1	1	0	3
mmu-miR-223-3p	109241	Mbd5	1	0	0	1	1	3
mmu-miR-223-3p	109264	Me3	1	0	1	1	0	3
mmu-miR-223-3p	109624	Cald1	1	0	1	1	0	3
mmu-miR-223-3p	109658	Txlna	0	1	1	1	0	3
mmu-miR-223-3p	109910	Zfp91	1	0	1	1	0	3
mmu-miR-223-3p	110012	Tpgs1	1	0	1	1	0	3
mmu-miR-223-3p	110157	Raf1	1	0	1	1	0	3
mmu-miR-223-3p	110173	Manba	0	0	1	1	1	3
mmu-miR-223-3p	110213	Tmbim6	1	0	1	1	0	3
mmu-miR-223-3p	110380	Shroom2	1	0	1	1	0	3
mmu-miR-223-3p	110611	Hdlbp	1	0	1	1	0	3
mmu-miR-223-3p	110616	Atxn3	1	0	1	1	0	3
mmu-miR-223-3p	110750	Cse1l	1	0	1	1	0	3
mmu-miR-223-3p	110809	Srsf1	1	0	0	1	1	3
mmu-miR-223-3p	110880	Scn4a	1	0	1	1	0	3
mmu-miR-223-3p	110911	Cds2	0	1	1	1	0	3
mmu-miR-223-3p	111173	Erc1	1	0	1	1	0	3

mmu-miR-223-3p	112422	2610305D13Rik	1	0	1	1	0	3
mmu-miR-223-3p	114142	Foxp2	1	1	0	1	0	3
mmu-miR-223-3p	114255	Dok4	0	1	1	1	0	3
mmu-miR-223-3p	114601	Ehbp1l1	1	0	0	1	1	3
mmu-miR-223-3p	114602	Zmynd10	1	0	1	1	0	3
mmu-miR-223-3p	114741	Supt16	1	1	0	1	0	3
mmu-miR-223-3p	114875	Plcz1	1	1	0	0	1	3
mmu-miR-223-3p	114889	Vsx1	1	0	0	1	1	3
mmu-miR-223-3p	116731	Pcdha1	1	0	0	1	1	3
mmu-miR-223-3p	116848	Baz2a	1	0	1	1	0	3
mmu-miR-223-3p	116905	Dph1	1	0	0	1	1	3
mmu-miR-223-3p	117197	Bloc1s4	1	0	1	1	0	3
mmu-miR-223-3p	117592	B3galt6	1	0	1	1	0	3
mmu-miR-223-3p	118449	Synpo2	1	0	0	1	1	3
mmu-miR-223-3p	140579	Elmo2	1	0	0	1	1	3
mmu-miR-223-3p	140630	Ube4a	1	1	0	1	0	3
mmu-miR-223-3p	170460	Stard5	1	0	1	0	1	3
mmu-miR-223-3p	170484	Nphs2	1	0	1	1	0	3
mmu-miR-223-3p	170639	Olfr78	1	0	1	1	0	3
mmu-miR-223-3p	170643	Kirrel	1	1	0	1	0	3
mmu-miR-223-3p	170719	Oxr1	1	0	1	1	0	3
mmu-miR-223-3p	170737	Znrf1	0	1	1	1	0	3
mmu-miR-223-3p	170740	Zfp287	1	0	1	1	0	3
mmu-miR-223-3p	170822	Usp33	1	1	0	1	0	3
mmu-miR-223-3p	170826	Ppargc1b	0	1	1	1	0	3
mmu-miR-223-3p	170835	Inpp5j	1	0	0	1	1	3
mmu-miR-223-3p	171170	Mbnl3	1	0	1	1	0	3
mmu-miR-223-3p	171210	Acot2	1	1	0	1	0	3
mmu-miR-223-3p	171567	Nme7	1	0	1	0	1	3
mmu-miR-223-3p	192157	Socs7	1	0	1	1	0	3
mmu-miR-223-3p	192163	Pcdha3	1	0	0	1	1	3
mmu-miR-223-3p	192167	Nlgn1	1	0	1	1	0	3
mmu-miR-223-3p	192191	Med9	1	0	1	1	0	3
mmu-miR-223-3p	192196	Luc7l2	1	1	0	1	0	3
mmu-miR-223-3p	192197	Bcas3	1	0	1	1	0	3
mmu-miR-223-3p	192657	Ell2	1	0	1	1	0	3
mmu-miR-223-3p	192678	Rassf3	1	0	1	1	0	3
mmu-miR-223-3p	193003	Pirt	0	1	1	1	0	3
mmu-miR-223-3p	193453	Gm11292	1	0	0	1	1	3
mmu-miR-223-3p	193670	Rnf185	1	0	1	1	0	3
mmu-miR-223-3p	194401	Mical3	1	0	0	1	1	3
mmu-miR-223-3p	195018	Zzef1	1	0	1	1	0	3
mmu-miR-223-3p	195434	Utp14b	1	0	1	1	0	3
mmu-miR-223-3p	195564	Skint3	1	0	1	1	0	3
mmu-miR-223-3p	207212	Arhgef17	1	0	1	1	0	3
mmu-miR-223-3p	207227	Stxbp5l	1	0	0	1	1	3
mmu-miR-223-3p	207425	Wdr11	1	0	1	1	0	3
mmu-miR-223-3p	207798	Gramd1c	1	0	1	1	0	3
mmu-miR-223-3p	207806	Gm608	1	0	1	1	0	3
mmu-miR-223-3p	207839	Galnt6	1	0	1	1	0	3
mmu-miR-223-3p	208117	Aph1b	1	0	1	1	0	3
mmu-miR-223-3p	208618	Etl4	0	1	1	1	0	3
mmu-miR-223-3p	208647	Creb3l2	0	1	1	1	0	3
mmu-miR-223-3p	208715	Hmgcs1	1	1	0	0	1	3
mmu-miR-223-3p	208968	Zfp280c	1	0	1	1	0	3

mmu-miR-223-3p	209032	Zc3hav1l	1	0	1	1	0	3
mmu-miR-223-3p	209091	Ccnb3	1	0	1	1	0	3
mmu-miR-223-3p	209200	Dtx3l	0	1	1	1	0	3
mmu-miR-223-3p	209239	Gan	0	1	1	1	0	3
mmu-miR-223-3p	209334	Gen1	1	0	1	1	0	3
mmu-miR-223-3p	209478	Tbc1d12	1	0	1	1	0	3
mmu-miR-223-3p	209707	Lcorl	1	0	1	1	0	3
mmu-miR-223-3p	209743	AF529169	0	1	1	1	0	3
mmu-miR-223-3p	210035	Tmem194	0	1	1	1	0	3
mmu-miR-223-3p	210274	Shank2	1	1	0	1	0	3
mmu-miR-223-3p	210573	Tmem151b	1	1	0	1	0	3
mmu-miR-223-3p	210757	Themis	1	0	1	1	0	3
mmu-miR-223-3p	210766	Brcc3	1	0	1	1	0	3
mmu-miR-223-3p	210801	Unc5d	1	0	0	1	1	3
mmu-miR-223-3p	210973	Kbtbd2	0	1	1	1	0	3
mmu-miR-223-3p	210998	D15Ertd621e	1	0	1	1	0	3
mmu-miR-223-3p	211064	Alkbh1	1	0	1	1	0	3
mmu-miR-223-3p	211187	Lrtm2	0	1	1	1	0	3
mmu-miR-223-3p	211347	Pank3	1	0	1	1	0	3
mmu-miR-223-3p	211484	Tsga10	1	0	1	1	0	3
mmu-miR-223-3p	211488	Ado	1	0	1	1	0	3
mmu-miR-223-3p	212190	Ubxn10	1	0	1	1	0	3
mmu-miR-223-3p	212281	A530054K11Rik	1	0	1	1	0	3
mmu-miR-223-3p	212514	Spice1	1	0	1	1	0	3
mmu-miR-223-3p	212518	Sprn	1	0	1	1	0	3
mmu-miR-223-3p	212541	Rho	1	0	1	1	0	3
mmu-miR-223-3p	212632	Iffo2	1	0	1	1	0	3
mmu-miR-223-3p	212986	Scfd2	1	0	1	1	0	3
mmu-miR-223-3p	213119	Itga10	0	1	1	1	0	3
mmu-miR-223-3p	213417	Klhdc8a	1	0	1	1	0	3
mmu-miR-223-3p	213464	Rbbp5	1	0	1	0	1	3
mmu-miR-223-3p	213556	Plekhh2	1	0	1	1	0	3
mmu-miR-223-3p	214162	Kmt2a	0	1	1	1	0	3
mmu-miR-223-3p	214240	Disp2	0	1	1	1	0	3
mmu-miR-223-3p	214489	BC003965	1	0	0	1	1	3
mmu-miR-223-3p	214531	Tmprss13	0	1	1	1	0	3
mmu-miR-223-3p	214604	4932411E22Rik	1	0	1	1	0	3
mmu-miR-223-3p	214704	Iqub	0	1	1	1	0	3
mmu-miR-223-3p	214899	Kdm5a	0	1	0	1	1	3
mmu-miR-223-3p	214922	Slc39a2	1	0	1	1	0	3
mmu-miR-223-3p	215015	Fam20b	1	0	1	1	0	3
mmu-miR-223-3p	215113	Slc43a2	1	0	1	1	0	3
mmu-miR-223-3p	215114	Hip1	1	0	1	1	0	3
mmu-miR-223-3p	215257	Il1f9	1	0	0	1	1	3
mmu-miR-223-3p	215418	Csrnp1	0	1	1	1	0	3
mmu-miR-223-3p	215476	Prr14l	1	0	1	1	0	3
mmu-miR-223-3p	215653	Rassf2	1	0	1	1	0	3
mmu-miR-223-3p	215748	Cnksr3	1	1	0	1	0	3
mmu-miR-223-3p	215821	D10Bwg1379e	0	1	1	1	0	3
mmu-miR-223-3p	215928	BC021785	1	0	1	1	0	3
mmu-miR-223-3p	216011	Lrrc20	1	0	1	1	0	3
mmu-miR-223-3p	216225	Slc5a8	1	0	1	1	0	3
mmu-miR-223-3p	216345	Zfc3h1	0	1	1	1	0	3
mmu-miR-223-3p	216578	Papolg	1	0	1	1	0	3
mmu-miR-223-3p	216749	Nmur2	1	0	1	0	1	3

mmu-miR-223-3p	216760	Mfap3	1	0	0	1	1	3
mmu-miR-223-3p	216892	Spns2	1	0	0	1	1	3
mmu-miR-223-3p	216974	Proca1	1	0	1	0	1	3
mmu-miR-223-3p	217038	Mrm1	0	1	1	1	0	3
mmu-miR-223-3p	217124	Ppp1r9b	1	0	1	1	0	3
mmu-miR-223-3p	217232	Cdc27	0	1	1	1	0	3
mmu-miR-223-3p	217265	Abca5	1	0	1	1	0	3
mmu-miR-223-3p	217331	Unk	1	0	1	1	0	3
mmu-miR-223-3p	217351	Tnrc6c	1	0	1	1	0	3
mmu-miR-223-3p	217365	Nploc4	1	0	0	1	1	3
mmu-miR-223-3p	217705	Fam161b	1	0	1	1	0	3
mmu-miR-223-3p	217733	Tmem63c	1	0	1	0	1	3
mmu-miR-223-3p	218214	Kdm1b	0	1	1	1	0	3
mmu-miR-223-3p	218441	Zfyve16	1	0	1	1	0	3
mmu-miR-223-3p	218461	Pde8b	1	0	1	1	0	3
mmu-miR-223-3p	218756	Slc4a7	1	0	1	1	0	3
mmu-miR-223-3p	218811	Sec24c	0	1	1	1	0	3
mmu-miR-223-3p	218975	Mapk1ip1l	1	0	1	1	0	3
mmu-miR-223-3p	219105	Zmym5	1	0	1	1	0	3
mmu-miR-223-3p	219135	Mtmr6	0	1	1	1	0	3
mmu-miR-223-3p	219257	Pcdh20	1	0	1	1	0	3
mmu-miR-223-3p	223272	Itgbl1	1	0	1	1	0	3
mmu-miR-223-3p	224014	Fgd4	1	0	1	1	0	3
mmu-miR-223-3p	224116	Muc20	1	1	0	1	0	3
mmu-miR-223-3p	224139	Golgb1	1	1	0	0	1	3
mmu-miR-223-3p	224250	Cldn25	1	0	1	1	0	3
mmu-miR-223-3p	224530	Acat3	1	0	1	1	0	3
mmu-miR-223-3p	224598	Zfp758	1	0	1	1	0	3
mmu-miR-223-3p	224671	Btbd9	1	1	0	1	0	3
mmu-miR-223-3p	224792	Gpr116	1	0	1	1	0	3
mmu-miR-223-3p	225010	Lclat1	0	1	1	1	0	3
mmu-miR-223-3p	225049	Ttc7	1	0	1	1	0	3
mmu-miR-223-3p	225160	Thoc1	1	0	1	1	0	3
mmu-miR-223-3p	225289	AW554918	0	1	1	1	0	3
mmu-miR-223-3p	225339	Ammecr1l	1	0	1	1	0	3
mmu-miR-223-3p	225583	A730017C20Rik	1	0	1	1	0	3
mmu-miR-223-3p	225600	Pde6a	1	0	1	1	0	3
mmu-miR-223-3p	225845	Pla2g16	1	0	1	1	0	3
mmu-miR-223-3p	225876	Kdm2a	0	1	1	1	0	3
mmu-miR-223-3p	225929	Patl1	0	1	1	1	0	3
mmu-miR-223-3p	226043	Cbwd1	1	0	1	0	1	3
mmu-miR-223-3p	226090	Ermp1	1	0	1	1	0	3
mmu-miR-223-3p	226252	Fam160b1	1	0	1	1	0	3
mmu-miR-223-3p	226409	Zranb3	1	0	1	1	0	3
mmu-miR-223-3p	226518	Nmnat2	1	0	1	1	0	3
mmu-miR-223-3p	226519	Lamc1	0	1	1	1	0	3
mmu-miR-223-3p	226591	Tiprl	1	0	1	1	0	3
mmu-miR-223-3p	226856	Lpgat1	1	0	0	1	1	3
mmu-miR-223-3p	227099	Pms1	1	0	1	1	0	3
mmu-miR-223-3p	227210	Ccnyl1	1	0	1	1	0	3
mmu-miR-223-3p	227525	Dclre1c	1	0	1	0	1	3
mmu-miR-223-3p	227526	Cdnf	1	0	1	1	0	3
mmu-miR-223-3p	227648	Sec16a	1	0	1	1	0	3
mmu-miR-223-3p	227737	Fam129b	1	0	1	1	0	3
mmu-miR-223-3p	227835	Gtdc1	1	0	1	1	0	3

mmu-miR-223-3p	227867	Epc2	1	0	1	1	0	3
mmu-miR-223-3p	227960	Gca	1	0	1	1	0	3
mmu-miR-223-3p	228005	Ppig	1	0	1	1	0	3
mmu-miR-223-3p	228019	Mettl8	1	1	0	1	0	3
mmu-miR-223-3p	228026	Pdk1	1	0	1	1	0	3
mmu-miR-223-3p	228368	Slc35c1	1	0	0	1	1	3
mmu-miR-223-3p	228410	Cstf3	1	0	1	1	0	3
mmu-miR-223-3p	228543	Rhov	1	0	1	1	0	3
mmu-miR-223-3p	228662	Btbd3	1	1	0	1	0	3
mmu-miR-223-3p	228788	Ccm2l	1	0	1	1	0	3
mmu-miR-223-3p	228790	Asxl1	0	1	1	1	0	3
mmu-miR-223-3p	228852	Ppp1r16b	1	0	1	1	0	3
mmu-miR-223-3p	228880	Zmynd8	0	1	1	1	0	3
mmu-miR-223-3p	229279	Hnrnpa3	1	1	0	1	0	3
mmu-miR-223-3p	229445	Ctso	1	0	1	1	0	3
mmu-miR-223-3p	229706	Slc6a17	1	0	1	1	0	3
mmu-miR-223-3p	229791	D3Bwg0562e	1	0	1	1	0	3
mmu-miR-223-3p	229877	Rap1gds1	1	0	0	1	1	3
mmu-miR-223-3p	230073	Ddx58	1	0	1	1	0	3
mmu-miR-223-3p	230088	Fam214b	1	0	1	1	0	3
mmu-miR-223-3p	230157	Tmeff1	1	0	1	1	0	3
mmu-miR-223-3p	230162	Zfp189	1	0	1	0	1	3
mmu-miR-223-3p	230584	Yipf1	1	0	1	1	0	3
mmu-miR-223-3p	230784	Sesn2	0	1	1	1	0	3
mmu-miR-223-3p	230809	Pdik1l	1	0	1	1	0	3
mmu-miR-223-3p	230822	Ncmap	1	0	1	1	0	3
mmu-miR-223-3p	230883	Aadacl3	1	0	1	1	0	3
mmu-miR-223-3p	230917	Tmem201	0	1	1	1	0	3
mmu-miR-223-3p	231207	Cpeb2	0	1	1	1	0	3
mmu-miR-223-3p	231238	Sel1l3	1	0	1	1	0	3
mmu-miR-223-3p	231717	Fam109a	1	0	1	1	0	3
mmu-miR-223-3p	231866	Zfp12	0	1	1	1	0	3
mmu-miR-223-3p	231999	Plekha8	1	0	0	1	1	3
mmu-miR-223-3p	232016	Ccdc129	1	0	1	0	1	3
mmu-miR-223-3p	232035	Ccser1	1	0	0	1	1	3
mmu-miR-223-3p	232157	Mob1a	0	1	1	1	0	3
mmu-miR-223-3p	232164	Paip2b	0	1	1	1	0	3
mmu-miR-223-3p	232174	Cyp26b1	1	0	1	1	0	3
mmu-miR-223-3p	232237	Fgd5	1	0	1	1	0	3
mmu-miR-223-3p	232286	Tmf1	1	0	0	1	1	3
mmu-miR-223-3p	232314	Ppp4r2	0	1	1	1	0	3
mmu-miR-223-3p	232333	Slc6a1	1	0	1	1	0	3
mmu-miR-223-3p	232408	Klrb1f	1	1	0	0	1	3
mmu-miR-223-3p	232533	Stk38l	0	1	1	1	0	3
mmu-miR-223-3p	232878	Zscan22	1	0	1	1	0	3
mmu-miR-223-3p	233187	Lim2	1	0	1	1	0	3
mmu-miR-223-3p	233189	Ctu1	0	1	1	1	0	3
mmu-miR-223-3p	233231	Mrgprb1	1	0	1	1	0	3
mmu-miR-223-3p	233246	Ano5	1	0	1	1	0	3
mmu-miR-223-3p	233332	Adamts17	1	0	1	1	0	3
mmu-miR-223-3p	233489	Picalm	0	1	1	1	0	3
mmu-miR-223-3p	233549	Mogat2	1	0	1	1	0	3
mmu-miR-223-3p	233805	Dcun1d3	1	0	1	1	0	3
mmu-miR-223-3p	233826	Palb2	0	1	1	1	0	3
mmu-miR-223-3p	233833	Tnrc6a	0	1	1	1	0	3

mmu-miR-223-3p	233919	Gpr26	1	0	1	1	0	3
mmu-miR-223-3p	233979	Tpcn2	1	0	1	1	0	3
mmu-miR-223-3p	234023	Arglu1	0	1	1	1	0	3
mmu-miR-223-3p	234135	Whsc1l1	1	0	1	1	0	3
mmu-miR-223-3p	234663	Dync1li2	1	0	1	1	0	3
mmu-miR-223-3p	234673	Ces2e	1	1	0	1	0	3
mmu-miR-223-3p	234724	Tat	1	0	1	1	0	3
mmu-miR-223-3p	234725	Zfp612	1	0	0	1	1	3
mmu-miR-223-3p	234733	Ddx19b	1	1	0	1	0	3
mmu-miR-223-3p	234797	6430548M08Rik	0	1	1	1	0	3
mmu-miR-223-3p	234959	Med17	1	0	1	1	0	3
mmu-miR-223-3p	235041	Kank2	0	1	1	1	0	3
mmu-miR-223-3p	235048	Zfp599	0	1	1	1	0	3
mmu-miR-223-3p	235320	Zbtb16	1	0	1	1	0	3
mmu-miR-223-3p	235339	Dlat	1	1	0	1	0	3
mmu-miR-223-3p	235386	Hykk	1	0	1	1	0	3
mmu-miR-223-3p	235472	Prtg	1	0	1	1	0	3
mmu-miR-223-3p	235504	Slc17a5	1	1	0	1	0	3
mmu-miR-223-3p	235628	Prss42	1	0	1	1	0	3
mmu-miR-223-3p	236576	Spry3	1	0	0	1	1	3
mmu-miR-223-3p	236915	Arhgef9	1	0	1	1	0	3
mmu-miR-223-3p	237052	Tceal1	0	1	1	1	0	3
mmu-miR-223-3p	237073	Rbm41	1	0	0	1	1	3
mmu-miR-223-3p	237107	Gnl3l	1	1	0	1	0	3
mmu-miR-223-3p	237213	Glra2	0	1	1	1	0	3
mmu-miR-223-3p	237253	Lrp11	1	0	1	1	0	3
mmu-miR-223-3p	237459	Cdk17	0	1	1	0	1	3
mmu-miR-223-3p	237858	Tusc5	1	0	1	1	0	3
mmu-miR-223-3p	237898	Usp32	1	0	1	1	0	3
mmu-miR-223-3p	238076	Kcns3	0	1	1	1	0	3
mmu-miR-223-3p	238130	Dock4	0	1	1	1	0	3
mmu-miR-223-3p	238161	Akap6	0	1	1	1	0	3
mmu-miR-223-3p	238276	Akap5	0	1	1	1	0	3
mmu-miR-223-3p	238386	Btbd7	0	1	1	1	0	3
mmu-miR-223-3p	238564	Mylk4	1	0	0	1	1	3
mmu-miR-223-3p	238725	Gpr150	1	0	1	1	0	3
mmu-miR-223-3p	238988	Erc2	1	0	1	1	0	3
mmu-miR-223-3p	239188	Enox1	1	0	1	1	0	3
mmu-miR-223-3p	239555	Smcr7l	1	0	1	1	0	3
mmu-miR-223-3p	239650	AI836003	1	0	1	1	0	3
mmu-miR-223-3p	239667	Dip2b	1	0	1	1	0	3
mmu-miR-223-3p	239852	Zpld1	0	1	1	1	0	3
mmu-miR-223-3p	240055	Neurl1b	1	0	0	1	1	3
mmu-miR-223-3p	240063	Zfp811	1	0	0	1	1	3
mmu-miR-223-3p	240185	9430020K01Rik	1	0	1	1	0	3
mmu-miR-223-3p	240327	Gm4951	1	0	1	1	0	3
mmu-miR-223-3p	240444	Kcng2	1	0	0	1	1	3
mmu-miR-223-3p	240752	Pik3c2b	1	0	0	1	1	3
mmu-miR-223-3p	240843	Brinp2	1	0	1	0	1	3
mmu-miR-223-3p	240869	Zbtb37	1	0	1	1	0	3
mmu-miR-223-3p	241062	Pgap1	1	1	0	1	0	3
mmu-miR-223-3p	241303	Fam78a	0	1	1	1	0	3
mmu-miR-223-3p	241322	Zbtb6	1	0	1	1	0	3
mmu-miR-223-3p	241490	Rbm45	0	1	1	1	0	3
mmu-miR-223-3p	241528	Lrrc55	1	0	1	1	0	3

mmu-miR-223-3p	241556	Tspan18	1	0	1	1	0	3
mmu-miR-223-3p	241636	Tgm6	1	0	1	1	0	3
mmu-miR-223-3p	241638	Lzts3	0	1	1	1	0	3
mmu-miR-223-3p	241639	Fermt1	0	1	1	1	0	3
mmu-miR-223-3p	241688	Dzank1	1	0	1	1	0	3
mmu-miR-223-3p	241694	Ralgapa2	0	1	1	1	0	3
mmu-miR-223-3p	241727	Snph	1	0	1	1	0	3
mmu-miR-223-3p	241919	Slc7a14	1	0	1	1	0	3
mmu-miR-223-3p	242126	Slc22a15	1	0	0	1	1	3
mmu-miR-223-3p	242377	Pm20d2	1	0	0	1	1	3
mmu-miR-223-3p	242406	Rgp1	1	0	1	1	0	3
mmu-miR-223-3p	242474	Tmem245	1	0	1	1	0	3
mmu-miR-223-3p	242521	Klhl9	1	0	1	1	0	3
mmu-miR-223-3p	242570	Raver2	1	0	1	1	0	3
mmu-miR-223-3p	242584	Wdr78	0	1	1	1	0	3
mmu-miR-223-3p	242653	Cldn19	1	0	1	1	0	3
mmu-miR-223-3p	242700	Ifnlr1	1	0	1	1	0	3
mmu-miR-223-3p	242705	E2f2	1	0	1	1	0	3
mmu-miR-223-3p	242721	Klhdc7a	0	1	1	1	0	3
mmu-miR-223-3p	243083	Tmprss11f	1	0	1	1	0	3
mmu-miR-223-3p	243270	Gpr81	1	0	1	1	0	3
mmu-miR-223-3p	243362	Stard13	1	0	1	1	0	3
mmu-miR-223-3p	243371	Lrrc61	1	1	0	1	0	3
mmu-miR-223-3p	243407	C130060K24Rik	1	0	1	1	0	3
mmu-miR-223-3p	243510	Ccdc142	1	0	1	1	0	3
mmu-miR-223-3p	243548	Prickle2	1	1	0	1	0	3
mmu-miR-223-3p	243616	Slc6a11	1	0	1	1	0	3
mmu-miR-223-3p	243833	Zfp128	1	0	1	1	0	3
mmu-miR-223-3p	243853	Fkrp	1	0	1	1	0	3
mmu-miR-223-3p	243912	Hspb6	1	0	1	1	0	3
mmu-miR-223-3p	243923	Rgs9bp	1	0	1	1	0	3
mmu-miR-223-3p	244141	Nars2	1	0	1	1	0	3
mmu-miR-223-3p	244237	Tnfrsf26	1	0	1	1	0	3
mmu-miR-223-3p	244431	Sgcz	1	0	1	1	0	3
mmu-miR-223-3p	244650	Phlpp2	1	0	1	1	0	3
mmu-miR-223-3p	244713	Zfp317	1	0	1	1	0	3
mmu-miR-223-3p	244745	Dpy19l1	1	0	1	1	0	3
mmu-miR-223-3p	244859	Ankk1	1	0	1	1	0	3
mmu-miR-223-3p	244885	Sh2d7	0	1	1	1	0	3
mmu-miR-223-3p	244911	C2cd4a	1	1	0	0	1	3
mmu-miR-223-3p	244923	Klhl31	1	0	1	1	0	3
mmu-miR-223-3p	245174	Zfp937	1	0	0	1	1	3
mmu-miR-223-3p	245269	Nim1	1	1	0	1	0	3
mmu-miR-223-3p	245386	Tmem255a	1	0	1	1	0	3
mmu-miR-223-3p	245424	Gpr101	1	0	1	1	0	3
mmu-miR-223-3p	245446	Slitrk4	1	1	0	1	0	3
mmu-miR-223-3p	245572	Tbx22	1	0	1	1	0	3
mmu-miR-223-3p	245596	Hdx	1	1	0	1	0	3
mmu-miR-223-3p	245616	Kir3dl1	1	1	0	1	0	3
mmu-miR-223-3p	245945	Rbm47	1	0	0	1	1	3
mmu-miR-223-3p	246104	Rhbdl3	0	1	1	1	0	3
mmu-miR-223-3p	246133	Kcne2	1	0	1	0	1	3
mmu-miR-223-3p	246198	Mllt6	0	1	1	1	0	3
mmu-miR-223-3p	246313	Prokr2	1	0	1	1	0	3
mmu-miR-223-3p	252864	Dusp15	1	1	0	1	0	3

mmu-miR-223-3p	252870	Usp7	1	1	0	1	0	3
mmu-miR-223-3p	252966	Cables2	0	1	1	1	0	3
mmu-miR-223-3p	252972	Tpcn1	1	0	1	1	0	3
mmu-miR-223-3p	257632	Nod2	0	1	1	1	0	3
mmu-miR-223-3p	258336	Olfr77	1	0	1	1	0	3
mmu-miR-223-3p	258364	Olfr976	1	1	0	1	0	3
mmu-miR-223-3p	263764	Creg2	1	0	1	1	0	3
mmu-miR-223-3p	263876	Spata2	0	1	1	1	0	3
mmu-miR-223-3p	264064	Cdk8	1	0	1	0	1	3
mmu-miR-223-3p	266690	Cyb5r4	1	0	0	1	1	3
mmu-miR-223-3p	266692	Cpne1	1	1	0	1	0	3
mmu-miR-223-3p	268345	Kcnc2	1	0	1	1	0	3
mmu-miR-223-3p	268527	Greb1	1	0	1	1	0	3
mmu-miR-223-3p	268566	Gphn	1	1	0	1	0	3
mmu-miR-223-3p	268706	Slc38a9	1	0	0	1	1	3
mmu-miR-223-3p	268741	Tox4	1	1	0	1	0	3
mmu-miR-223-3p	268782	Agxt2	0	1	1	1	0	3
mmu-miR-223-3p	268859	Rbfox1	1	0	1	1	0	3
mmu-miR-223-3p	268980	Strn	1	0	1	0	1	3
mmu-miR-223-3p	269019	Stk32a	0	1	1	1	0	3
mmu-miR-223-3p	269037	Ctif	0	1	1	1	0	3
mmu-miR-223-3p	269053	Gpr152	1	0	1	0	1	3
mmu-miR-223-3p	269181	Mgat4a	1	0	1	1	0	3
mmu-miR-223-3p	269344	Ell3	1	0	1	1	0	3
mmu-miR-223-3p	269424	Phf17	1	1	0	1	0	3
mmu-miR-223-3p	269614	Pank4	1	0	1	0	1	3
mmu-miR-223-3p	269639	Zfp512	0	1	1	1	0	3
mmu-miR-223-3p	269642	Nat8l	0	1	1	1	0	3
mmu-miR-223-3p	269695	Rnft2	1	0	0	1	1	3
mmu-miR-223-3p	269774	Aak1	1	0	0	1	1	3
mmu-miR-223-3p	269941	Chsy1	1	0	1	1	0	3
mmu-miR-223-3p	269952	Gdpgp1	1	0	1	1	0	3
mmu-miR-223-3p	269999	Orai3	0	1	1	1	0	3
mmu-miR-223-3p	270066	Slc35e1	1	0	1	1	0	3
mmu-miR-223-3p	270118	Maml2	1	1	0	1	0	3
mmu-miR-223-3p	270201	Klhl18	1	0	1	1	0	3
mmu-miR-223-3p	270210	Zfp651	1	0	0	1	1	3
mmu-miR-223-3p	270711	Fam26d	1	0	1	1	0	3
mmu-miR-223-3p	270802	BC048403	1	0	1	1	0	3
mmu-miR-223-3p	270906	Prr11	1	0	1	1	0	3
mmu-miR-223-3p	271305	Phf21b	1	0	1	1	0	3
mmu-miR-223-3p	271786	Galnt13	1	0	1	1	0	3
mmu-miR-223-3p	272031	E130309F12Rik	1	1	0	1	0	3
mmu-miR-223-3p	272382	Spib	1	0	1	1	0	3
mmu-miR-223-3p	277250	Kdm3b	1	0	1	1	0	3
mmu-miR-223-3p	278097	Armcx6	0	1	1	1	0	3
mmu-miR-223-3p	278174	Ssxb3	1	0	0	1	1	3
mmu-miR-223-3p	278304	Zfp385c	1	0	1	1	0	3
mmu-miR-223-3p	280635	Emilin3	1	0	1	1	0	3
mmu-miR-223-3p	280645	B3gat2	1	0	1	1	0	3
mmu-miR-223-3p	286940	Flnb	0	1	1	1	0	3
mmu-miR-223-3p	319211	Nol4	0	1	1	1	0	3
mmu-miR-223-3p	319263	Pcmtd1	1	0	1	1	0	3
mmu-miR-223-3p	319293	A530099J19Rik	1	0	1	1	0	3
mmu-miR-223-3p	319317	Snhg11	1	0	1	1	0	3

mmu-miR-223-3p	319387	Lphn3	0	1	1	1	0	3
mmu-miR-223-3p	319476	Lrtm1	1	1	0	1	0	3
mmu-miR-223-3p	319508	Syt15	1	0	1	1	0	3
mmu-miR-223-3p	319520	Dusp4	0	1	1	1	0	3
mmu-miR-223-3p	319555	Nwd1	1	0	0	1	1	3
mmu-miR-223-3p	319586	Celf5	1	1	0	1	0	3
mmu-miR-223-3p	319615	Zfp944	1	1	0	1	0	3
mmu-miR-223-3p	319622	Itpripl2	1	0	1	1	0	3
mmu-miR-223-3p	319651	Usp37	1	0	0	1	1	3
mmu-miR-223-3p	319670	Eml5	1	0	1	1	0	3
mmu-miR-223-3p	319710	Frmd6	1	0	1	1	0	3
mmu-miR-223-3p	319734	Cacna2d4	1	0	1	1	0	3
mmu-miR-223-3p	319758	Rfx7	0	1	1	1	0	3
mmu-miR-223-3p	319817	Rc3h2	0	1	1	1	0	3
mmu-miR-223-3p	319924	Apba1	1	1	0	1	0	3
mmu-miR-223-3p	320011	Uggt1	1	0	0	1	1	3
mmu-miR-223-3p	320024	Nceh1	1	0	1	1	0	3
mmu-miR-223-3p	320095	6430550D23Rik	1	0	0	1	1	3
mmu-miR-223-3p	320119	Rps6kc1	1	1	0	1	0	3
mmu-miR-223-3p	320145	Sp8	1	1	0	1	0	3
mmu-miR-223-3p	320158	Zmat4	1	0	1	1	0	3
mmu-miR-223-3p	320184	Lrrc58	0	1	1	1	0	3
mmu-miR-223-3p	320191	Hook3	1	0	1	1	0	3
mmu-miR-223-3p	320209	Ddx11	1	0	1	1	0	3
mmu-miR-223-3p	320213	Senp5	1	0	1	1	0	3
mmu-miR-223-3p	320244	Ttll5	0	1	1	1	0	3
mmu-miR-223-3p	320271	Scai	1	0	1	1	0	3
mmu-miR-223-3p	320354	E130003G02Rik	1	0	0	1	1	3
mmu-miR-223-3p	320360	Ric3	1	1	0	1	0	3
mmu-miR-223-3p	320407	Klri2	1	0	1	1	0	3
mmu-miR-223-3p	320438	Alg6	1	0	1	1	0	3
mmu-miR-223-3p	320472	Ppm1e	1	0	1	1	0	3
mmu-miR-223-3p	320487	Heatr5a	1	0	1	1	0	3
mmu-miR-223-3p	320500	Tmem215	0	1	1	1	0	3
mmu-miR-223-3p	320534	Tmem104	1	0	1	1	0	3
mmu-miR-223-3p	320538	Ubn2	1	0	1	1	0	3
mmu-miR-223-3p	320571	Atp8b5	0	1	1	1	0	3
mmu-miR-223-3p	320633	Zbtb26	1	0	1	1	0	3
mmu-miR-223-3p	320640	Skint4	1	0	1	1	0	3
mmu-miR-223-3p	320664	Cass4	1	0	1	1	0	3
mmu-miR-223-3p	320683	Zfp629	1	0	1	1	0	3
mmu-miR-223-3p	320709	Tmem117	0	1	1	1	0	3
mmu-miR-223-3p	320717	Pptc7	1	0	1	1	0	3
mmu-miR-223-3p	320718	Slc26a9	1	1	1	0	0	3
mmu-miR-223-3p	320727	Ipo8	1	0	1	1	0	3
mmu-miR-223-3p	320772	Mdga2	1	0	1	1	0	3
mmu-miR-223-3p	320782	Tmem154	1	0	1	1	0	3
mmu-miR-223-3p	320799	Zhx3	0	1	1	1	0	3
mmu-miR-223-3p	320878	Mical2	1	1	0	1	0	3
mmu-miR-223-3p	321007	Serac1	1	1	0	1	0	3
mmu-miR-223-3p	326623	Tnfsf15	1	0	0	1	1	3
mmu-miR-223-3p	327814	Ppfia2	1	0	1	1	0	3
mmu-miR-223-3p	328099	Prps1l3	1	0	0	1	1	3
mmu-miR-223-3p	328274	Zfp459	1	0	1	1	0	3
mmu-miR-223-3p	328505	Skint7	1	0	0	1	1	3

mmu-miR-223-3p	328977	Zfp532	0	1	1	1	0	3
mmu-miR-223-3p	329154	Ankrd44	1	0	1	1	0	3
mmu-miR-223-3p	329178	Unc80	1	1	0	1	0	3
mmu-miR-223-3p	329244	Il19	0	1	1	1	0	3
mmu-miR-223-3p	329274	Fam163a	1	0	1	1	0	3
mmu-miR-223-3p	329470	Accs	1	0	1	1	0	3
mmu-miR-223-3p	329540	8430427H17Rik	1	0	1	1	0	3
mmu-miR-223-3p	329628	Fat4	0	1	1	1	0	3
mmu-miR-223-3p	329693	Fcrl5	1	0	1	1	0	3
mmu-miR-223-3p	329828	AI464131	0	1	1	1	0	3
mmu-miR-223-3p	329941	Col8a2	1	0	1	1	0	3
mmu-miR-223-3p	330119	Adamts3	1	0	0	1	1	3
mmu-miR-223-3p	330177	Taok3	1	1	0	1	0	3
mmu-miR-223-3p	330267	Thsd7a	1	1	0	1	0	3
mmu-miR-223-3p	330286	D630045J12Rik	1	0	0	1	1	3
mmu-miR-223-3p	330323	Fam188b	1	0	0	1	1	3
mmu-miR-223-3p	330401	Tmcc1	1	1	0	1	0	3
mmu-miR-223-3p	331188	Zfp781	1	0	0	1	1	3
mmu-miR-223-3p	332937	Tfap2e	1	0	1	1	0	3
mmu-miR-223-3p	333048	Tmem211	1	0	1	1	0	3
mmu-miR-223-3p	333433	Gpd1l	1	0	0	1	1	3
mmu-miR-223-3p	333669	Gm5134	1	0	1	1	0	3
mmu-miR-223-3p	338367	Myo1d	1	0	1	1	0	3
mmu-miR-223-3p	338372	Map3k9	1	0	1	0	1	3
mmu-miR-223-3p	347722	Agap1	1	0	0	1	1	3
mmu-miR-223-3p	353047	Plekhm1	0	1	1	1	0	3
mmu-miR-223-3p	353130	Prss33	1	0	0	1	1	3
mmu-miR-223-3p	353190	Edc3	1	1	0	1	0	3
mmu-miR-223-3p	353234	Pcdha2	1	0	0	1	1	3
mmu-miR-223-3p	353235	Pcdha8	1	0	0	1	1	3
mmu-miR-223-3p	378700	Bpifb3	1	0	1	1	0	3
mmu-miR-223-3p	378702	Serf2	0	1	1	1	0	3
mmu-miR-223-3p	380614	Intu	1	0	1	1	0	3
mmu-miR-223-3p	380694	Ccnjl	1	0	1	1	0	3
mmu-miR-223-3p	380732	Milr1	1	0	0	1	1	3
mmu-miR-223-3p	380959	Alg10b	1	0	1	1	0	3
mmu-miR-223-3p	381022	Kmt2d	1	1	0	1	0	3
mmu-miR-223-3p	381067	Zfp229	1	0	0	1	1	3
mmu-miR-223-3p	381199	Tmem151a	1	0	1	1	0	3
mmu-miR-223-3p	381310	6330403A02Rik	1	0	1	1	0	3
mmu-miR-223-3p	381318	Nsl1	1	0	1	1	0	3
mmu-miR-223-3p	381334	Gal3st2	1	0	1	1	0	3
mmu-miR-223-3p	381694	B3galtl	1	0	1	1	0	3
mmu-miR-223-3p	382018	Unc13a	1	0	0	1	1	3
mmu-miR-223-3p	382034	Gse1	0	1	1	1	0	3
mmu-miR-223-3p	382045	Gpr114	1	0	1	1	0	3
mmu-miR-223-3p	382406	Poc1b	1	0	1	1	0	3
mmu-miR-223-3p	382423	Atxn7l3b	1	0	1	1	0	3
mmu-miR-223-3p	382793	Mtx3	1	1	0	1	0	3
mmu-miR-223-3p	383548	Serpinb3b	1	0	1	1	0	3
mmu-miR-223-3p	383787	Ankrd63	1	0	1	1	0	3
mmu-miR-223-3p	384309	Trim56	1	0	1	1	0	3
mmu-miR-223-3p	385312	Ssxb10	1	0	0	1	1	3
mmu-miR-223-3p	385354	Frmd7	1	1	0	1	0	3
mmu-miR-223-3p	387131	Ssxb9	1	0	0	1	1	3

mmu-miR-223-3p	387285	Hcrtr2	1	0	1	1	0	3
mmu-miR-223-3p	387586	Ssxb5	1	0	0	1	1	3
mmu-miR-223-3p	399558	Flrt2	1	1	0	1	0	3
mmu-miR-223-3p	403395	Clec3a	1	0	0	1	1	3
mmu-miR-223-3p	414801	Itprip	1	0	1	1	0	3
mmu-miR-223-3p	414872	Zyg11b	1	0	1	1	0	3
mmu-miR-223-3p	432450	Nkain2	1	0	1	1	0	3
mmu-miR-223-3p	432628	Mfsd2b	1	0	1	1	0	3
mmu-miR-223-3p	432731	Zscan26	1	0	1	1	0	3
mmu-miR-223-3p	432870	Gm5464	1	0	1	1	0	3
mmu-miR-223-3p	433586	Maml3	1	1	0	1	0	3
mmu-miR-223-3p	433791	Gm13251	1	0	1	1	0	3
mmu-miR-223-3p	433804	Gm13154	1	0	1	1	0	3
mmu-miR-223-3p	433904	Ociad2	1	0	1	1	0	3
mmu-miR-223-3p	433968	Gm5566	1	0	0	1	1	3
mmu-miR-223-3p	434778	Ccdc160	1	0	1	1	0	3
mmu-miR-223-3p	435337	Gm5662	1	0	1	1	0	3
mmu-miR-223-3p	436188	Gm5751	1	0	0	1	1	3
mmu-miR-223-3p	442834	D830031N03Rik	1	0	0	1	1	3
mmu-miR-223-3p	474156	Zbtb9	1	1	0	1	0	3
mmu-miR-223-3p	497097	Xkr4	1	0	1	1	0	3
mmu-miR-223-3p	545156	Kalrn	1	0	0	1	1	3
mmu-miR-223-3p	545384	BC094916	0	1	1	1	0	3
mmu-miR-223-3p	545428	Ccdc141	1	1	0	1	0	3
mmu-miR-223-3p	545471	Zfp345	1	0	1	1	0	3
mmu-miR-223-3p	545677	Gm12888	1	0	1	1	0	3
mmu-miR-223-3p	545814	Smok3a	1	0	0	1	1	3
mmu-miR-223-3p	546118	Ubtfl1	1	0	1	1	0	3
mmu-miR-223-3p	547349	LOC547349	1	0	0	1	1	3
mmu-miR-223-3p	574402	Gpr17	1	0	1	1	0	3
mmu-miR-223-3p	574418	Serinc4	1	0	1	1	0	3
mmu-miR-223-3p	619287	Zcchc16	1	1	0	1	0	3
mmu-miR-223-3p	620583	Gm9804	1	0	0	1	1	3
mmu-miR-223-3p	621832	Gm10349	1	0	0	1	1	3
mmu-miR-223-3p	621976	Tmem170b	1	0	0	1	1	3
mmu-miR-223-3p	623781	Gm14137	1	0	1	1	0	3
mmu-miR-223-3p	624224	Clrn2	1	0	0	1	1	3
mmu-miR-223-3p	626802	Gm14322	1	0	0	1	1	3
mmu-miR-223-3p	629016	Zfp953	1	0	1	1	0	3
mmu-miR-223-3p	631286	LOC631286	1	0	0	1	1	3
mmu-miR-223-3p	665113	Tnik	1	1	0	1	0	3
mmu-miR-223-3p	665563	Mthfd2l	1	0	1	1	0	3
mmu-miR-223-3p	666048	Trabd2b	1	0	1	1	0	3
mmu-miR-223-3p	666938	Bend4	1	0	0	1	1	3
mmu-miR-223-3p	667728	Hist1h2al	1	0	0	1	1	3
mmu-miR-223-3p	667803	C920025E04Rik	1	0	0	1	1	3
mmu-miR-223-3p	668208	Gm13288	1	0	0	1	1	3
mmu-miR-223-3p	670558	H60c	1	0	0	1	1	3
mmu-miR-223-3p	677654	LOC677654	1	0	0	1	1	3
mmu-miR-223-3p	1E+08	Pydc3	1	1	0	1	0	3
mmu-miR-223-3p	1E+08	Dnajc3	1	0	0	1	1	3
mmu-miR-223-3p	1E+08	Gm10767	0	1	0	1	1	3
mmu-miR-223-3p	1E+08	Gm2016	1	0	1	1	0	3
mmu-miR-223-3p	1E+08	Gm9780	1	0	0	1	1	3
mmu-miR-223-3p	1E+08	Plac9b	1	0	0	1	1	3

mmu-miR-223-3p	1E+08	Tgtp2	1	1	0	0	1	3
mmu-miR-223-3p	1E+08	Gm15411	1	0	0	1	1	3
mmu-miR-223-3p	1E+08	Gm12349	1	0	0	1	1	3
mmu-miR-223-3p	1E+08	Gm3065	1	0	0	1	1	3
mmu-miR-223-3p	1E+08	9030025P20Rik	1	0	1	1	0	3
mmu-miR-223-3p	1E+08	Gm3435	1	0	1	1	0	3
mmu-miR-223-3p	1E+08	Nhsl2	1	1	0	1	0	3
mmu-miR-223-3p	1E+08	Rex2	1	1	0	1	0	3
mmu-miR-223-3p	1E+08	Amy2a4	1	0	0	1	1	3
mmu-miR-223-3p	1E+08	Amy2a3	1	0	0	1	1	3
mmu-miR-223-3p	1E+08	Amy2a2	1	0	0	1	1	3
mmu-miR-223-3p	1E+08	LOC100044322	1	0	0	1	1	3
mmu-miR-223-3p	1E+08	LOC100047468	1	0	0	1	1	3
mmu-miR-223-3p	1E+08	LOC100047632	1	0	0	1	1	3
mmu-miR-223-3p	1E+08	LOC100047658	1	0	0	1	1	3
mmu-miR-223-3p	1E+08	Yy2	1	0	1	1	0	3
mmu-miR-223-3p	1E+08	1700047I17Rik2	1	0	0	1	1	3
mmu-miR-223-3p	1E+08	Gm20410	1	0	0	1	1	3
mmu-miR-223-3p	1.01E+08	LOC100502777	1	0	0	1	1	3
mmu-miR-223-3p	1.01E+08	E330021D16Rik	1	0	0	1	1	3
mmu-miR-223-3p	1.01E+08	Gm15070	1	0	0	1	1	3
mmu-miR-223-3p	1.01E+08	LOC100503280	1	0	0	1	1	3
mmu-miR-223-3p	1.01E+08	LOC100503822	1	0	0	1	1	3
mmu-miR-223-3p	1.01E+08	Ccdc149	1	0	0	1	1	3
mmu-miR-223-3p	1.01E+08	Gm20149	1	0	0	1	1	3
mmu-miR-223-3p	1.01E+08	Gm21464	1	0	0	1	1	3
mmu-miR-223-3p	1.01E+08	LOC100862570	1	0	0	1	1	3
mmu-miR-223-3p	1.01E+08	LOC100862584	1	0	0	1	1	3
mmu-miR-223-3p	1.01E+08	LOC100862597	1	0	0	1	1	3
mmu-miR-223-3p	1.01E+08	LOC101055654	1	0	0	1	1	3
mmu-miR-223-3p	1.01E+08	LOC101055656	1	0	0	1	1	3
mmu-miR-223-3p	1.01E+08	LOC101055679	1	0	0	1	1	3
mmu-miR-223-3p	1.01E+08	LOC101055691	1	0	0	1	1	3
mmu-miR-223-3p	1.01E+08	LOC101055738	1	0	0	1	1	3
mmu-miR-223-3p	1.01E+08	LOC101055780	1	0	0	1	1	3
mmu-miR-223-3p	1.01E+08	LOC101055787	1	0	0	1	1	3
mmu-miR-223-3p	1.01E+08	LOC101055811	1	0	0	1	1	3
mmu-miR-223-3p	1.01E+08	LOC101055871	1	0	0	1	1	3
mmu-miR-223-3p	1.01E+08	LOC101055889	1	0	0	1	1	3
mmu-miR-223-3p	1.01E+08	LOC101056016	1	0	0	1	1	3
mmu-miR-223-3p	1.01E+08	LOC101056072	1	0	0	1	1	3
mmu-miR-223-3p	1.01E+08	LOC101056077	1	0	0	1	1	3
mmu-miR-223-3p	1.01E+08	LOC101056086	1	0	0	1	1	3
mmu-miR-223-3p	1.01E+08	LOC101056104	1	0	0	1	1	3
mmu-miR-223-3p	1.01E+08	LOC101056322	1	0	0	1	1	3
mmu-miR-223-3p	1.01E+08	LOC101056392	1	0	0	1	1	3
mmu-miR-223-3p	1.01E+08	LOC101056419	1	0	0	1	1	3
mmu-miR-223-3p	1.01E+08	LOC101056602	1	0	0	1	1	3
mmu-miR-223-3p	11298	Aanat	1	0	0	1	0	2
mmu-miR-223-3p	11302	Aatk	0	0	1	1	0	2
mmu-miR-223-3p	11308	Abi1	1	0	0	1	0	2
mmu-miR-223-3p	11350	Abl1	1	0	0	1	0	2
mmu-miR-223-3p	11440	Chrna6	1	0	1	0	0	2
mmu-miR-223-3p	11448	Chrne	0	1	0	1	0	2
mmu-miR-223-3p	11450	Adipoq	1	0	0	1	0	2

mmu-miR-223-3p	11475	Acta2	0	0	1	1	0	2
mmu-miR-223-3p	11489	Adam12	0	0	1	1	0	2
mmu-miR-223-3p	11502	Adam9	0	0	1	1	0	2
mmu-miR-223-3p	11504	Adamts1	1	0	0	1	0	2
mmu-miR-223-3p	11517	Adcyap1r1	1	0	0	1	0	2
mmu-miR-223-3p	11536	Gpr182	0	1	0	1	0	2
mmu-miR-223-3p	11538	Adnp	0	0	1	1	0	2
mmu-miR-223-3p	11552	Adra2b	1	0	0	1	0	2
mmu-miR-223-3p	11595	Acan	1	0	0	1	0	2
mmu-miR-223-3p	11600	Angpt1	0	0	1	1	0	2
mmu-miR-223-3p	11605	Gla	1	0	0	1	0	2
mmu-miR-223-3p	11624	Ahrr	0	0	1	1	0	2
mmu-miR-223-3p	11630	Aim1	1	0	0	1	0	2
mmu-miR-223-3p	11632	Aip	1	0	0	1	0	2
mmu-miR-223-3p	11641	Akap2	1	0	0	1	0	2
mmu-miR-223-3p	11651	Akt1	0	0	1	1	0	2
mmu-miR-223-3p	11668	Aldh1a1	0	1	0	1	0	2
mmu-miR-223-3p	11671	Aldh3a2	0	0	1	1	0	2
mmu-miR-223-3p	11688	Alox8	0	0	1	1	0	2
mmu-miR-223-3p	11702	Amd1	1	0	0	1	0	2
mmu-miR-223-3p	11717	Ampd3	1	0	0	1	0	2
mmu-miR-223-3p	11735	Ank3	0	1	0	1	0	2
mmu-miR-223-3p	11754	Aoc3	1	0	0	1	0	2
mmu-miR-223-3p	11764	Ap1b1	1	0	0	1	0	2
mmu-miR-223-3p	11765	Ap1g1	1	0	0	1	0	2
mmu-miR-223-3p	11766	Ap1g2	1	1	0	0	0	2
mmu-miR-223-3p	11767	Ap1m1	1	0	0	1	0	2
mmu-miR-223-3p	11769	Ap1s1	0	0	1	1	0	2
mmu-miR-223-3p	11777	Ap3s1	0	0	1	1	0	2
mmu-miR-223-3p	11782	Ap4s1	0	0	1	1	0	2
mmu-miR-223-3p	11787	Apbb2	1	0	0	1	0	2
mmu-miR-223-3p	11790	Speg	0	0	1	1	0	2
mmu-miR-223-3p	11800	Api5	0	0	1	1	0	2
mmu-miR-223-3p	11804	Aplp2	0	1	0	1	0	2
mmu-miR-223-3p	11819	Nr2f2	1	0	0	1	0	2
mmu-miR-223-3p	11836	Araf	1	1	0	0	0	2
mmu-miR-223-3p	11838	Arc	0	0	1	1	0	2
mmu-miR-223-3p	11841	Arf2	0	0	1	1	0	2
mmu-miR-223-3p	11843	Arf4	1	0	1	0	0	2
mmu-miR-223-3p	11856	Arhgap6	0	1	0	1	0	2
mmu-miR-223-3p	11857	Arhgdib	1	0	0	1	0	2
mmu-miR-223-3p	11864	Arnt2	1	0	0	1	0	2
mmu-miR-223-3p	11867	Arpc1b	0	1	0	1	0	2
mmu-miR-223-3p	11878	Arx	1	0	0	1	0	2
mmu-miR-223-3p	11881	Arsb	0	0	1	1	0	2
mmu-miR-223-3p	11883	Arsa	1	0	1	0	0	2
mmu-miR-223-3p	11886	Asah1	1	0	0	1	0	2
mmu-miR-223-3p	11891	Rab27a	1	0	0	1	0	2
mmu-miR-223-3p	11909	Atf2	1	0	0	1	0	2
mmu-miR-223-3p	11910	Atf3	1	0	1	0	0	2
mmu-miR-223-3p	11923	Neurod4	1	0	0	1	0	2
mmu-miR-223-3p	11927	Atox1	1	0	0	1	0	2
mmu-miR-223-3p	11933	Atp1b3	1	0	0	1	0	2
mmu-miR-223-3p	11947	Atp5b	0	0	1	1	0	2
mmu-miR-223-3p	11964	Atp6v1a	0	0	1	1	0	2

mmu-miR-223-3p	11975	Atp6v0a1	0	0	1	1	0	2
mmu-miR-223-3p	11980	Atp8a1	1	0	0	1	0	2
mmu-miR-223-3p	11984	Atp6v0c	0	1	0	1	0	2
mmu-miR-223-3p	11987	Slc7a1	0	0	1	1	0	2
mmu-miR-223-3p	12000	Avpr2	0	0	1	1	0	2
mmu-miR-223-3p	12014	Bach2	1	0	0	1	0	2
mmu-miR-223-3p	12018	Bak1	0	0	1	1	0	2
mmu-miR-223-3p	12032	Bcan	0	0	1	1	0	2
mmu-miR-223-3p	12049	Bcl2l10	0	0	1	1	0	2
mmu-miR-223-3p	12054	Bcl7b	0	0	1	1	0	2
mmu-miR-223-3p	12057	Opn1sw	0	1	0	1	0	2
mmu-miR-223-3p	12062	Bdkrb2	0	0	1	1	0	2
mmu-miR-223-3p	12121	Bicd1	0	1	0	1	0	2
mmu-miR-223-3p	12156	Bmp2	0	0	1	1	0	2
mmu-miR-223-3p	12159	Bmp4	1	0	0	1	0	2
mmu-miR-223-3p	12161	Bmp6	1	0	0	1	0	2
mmu-miR-223-3p	12165	Gdf2	0	0	1	1	0	2
mmu-miR-223-3p	12173	Bnc1	0	0	1	1	0	2
mmu-miR-223-3p	12176	Bnip3	1	0	1	0	0	2
mmu-miR-223-3p	12181	Bop1	0	0	1	1	0	2
mmu-miR-223-3p	12182	Bst1	1	0	0	1	0	2
mmu-miR-223-3p	12192	Zfp36l1	0	0	1	1	0	2
mmu-miR-223-3p	12209	Brs3	1	0	0	1	0	2
mmu-miR-223-3p	12211	Birc6	0	0	1	1	0	2
mmu-miR-223-3p	12226	Btg1	1	0	0	1	0	2
mmu-miR-223-3p	12237	Bub3	0	0	1	1	0	2
mmu-miR-223-3p	12265	Ciita	0	0	1	1	0	2
mmu-miR-223-3p	12283	Cab39	0	0	1	1	0	2
mmu-miR-223-3p	12288	Cacna1c	1	0	0	1	0	2
mmu-miR-223-3p	12290	Cacna1e	0	0	1	1	0	2
mmu-miR-223-3p	12293	Cacna2d1	1	0	0	1	0	2
mmu-miR-223-3p	12296	Cacnb2	1	0	0	1	0	2
mmu-miR-223-3p	12298	Cacnb4	1	0	0	1	0	2
mmu-miR-223-3p	12300	Cacng2	1	0	0	1	0	2
mmu-miR-223-3p	12301	Cacybp	1	0	0	1	0	2
mmu-miR-223-3p	12321	Calu	1	0	0	1	0	2
mmu-miR-223-3p	12323	Camk2b	0	0	1	1	0	2
mmu-miR-223-3p	12340	Capza1	0	1	0	1	0	2
mmu-miR-223-3p	12343	Capza2	1	0	1	0	0	2
mmu-miR-223-3p	12349	Car2	1	0	0	1	0	2
mmu-miR-223-3p	12351	Car4	0	0	1	1	0	2
mmu-miR-223-3p	12366	Casp2	0	0	1	1	0	2
mmu-miR-223-3p	12387	Ctnnb1	0	1	0	1	0	2
mmu-miR-223-3p	12388	Ctnnd1	1	0	0	1	0	2
mmu-miR-223-3p	12389	Cav1	1	0	1	0	0	2
mmu-miR-223-3p	12390	Cav2	1	0	0	1	0	2
mmu-miR-223-3p	12393	Runx2	1	0	0	1	0	2
mmu-miR-223-3p	12394	Runx1	1	0	0	1	0	2
mmu-miR-223-3p	12395	Runx1t1	0	1	0	1	0	2
mmu-miR-223-3p	12396	Cbfa2t2	1	0	0	1	0	2
mmu-miR-223-3p	12398	Cbfa2t3	1	0	0	1	0	2
mmu-miR-223-3p	12399	Runx3	1	0	0	1	0	2
mmu-miR-223-3p	12412	Cbx1	0	0	1	1	0	2
mmu-miR-223-3p	12443	Ccnd1	1	0	0	1	0	2
mmu-miR-223-3p	12449	Ccnf	0	0	1	1	0	2

mmu-miR-223-3p	12453	Ccni	0	0	1	1	0	2
mmu-miR-223-3p	12457	Ccrn4l	1	0	0	1	0	2
mmu-miR-223-3p	12461	Cct2	0	0	1	1	0	2
mmu-miR-223-3p	12477	Ctla4	0	0	1	1	0	2
mmu-miR-223-3p	12478	Cd19	1	0	0	1	0	2
mmu-miR-223-3p	12484	Cd24a	1	0	0	1	0	2
mmu-miR-223-3p	12487	Cd28	1	0	0	1	0	2
mmu-miR-223-3p	12489	Cd33	0	1	0	1	0	2
mmu-miR-223-3p	12520	Cd81	0	0	1	1	0	2
mmu-miR-223-3p	12522	Cd83	0	0	1	1	0	2
mmu-miR-223-3p	12539	Cdc37	0	0	1	1	0	2
mmu-miR-223-3p	12560	Cdh3	0	1	0	1	0	2
mmu-miR-223-3p	12561	Cdh4	0	0	1	1	0	2
mmu-miR-223-3p	12565	Cdh9	0	0	1	1	0	2
mmu-miR-223-3p	12568	Cdk5	0	0	1	1	0	2
mmu-miR-223-3p	12576	Cdkn1b	0	1	0	1	0	2
mmu-miR-223-3p	12580	Cdkn2c	0	0	1	1	0	2
mmu-miR-223-3p	12589	Ift81	0	0	1	1	0	2
mmu-miR-223-3p	12590	Cdx1	1	0	0	1	0	2
mmu-miR-223-3p	12606	Cebpa	1	0	0	1	0	2
mmu-miR-223-3p	12617	Cenpc1	0	0	1	1	0	2
mmu-miR-223-3p	12628	Cfh	1	0	0	1	0	2
mmu-miR-223-3p	12631	Cfl1	1	0	0	1	0	2
mmu-miR-223-3p	12632	Cfl2	1	0	0	1	0	2
mmu-miR-223-3p	12633	Cflar	1	0	0	1	0	2
mmu-miR-223-3p	12642	Ch25h	0	1	0	1	0	2
mmu-miR-223-3p	12643	Chad	0	0	1	1	0	2
mmu-miR-223-3p	12648	Chd1	1	0	0	1	0	2
mmu-miR-223-3p	12660	Chka	1	0	0	1	0	2
mmu-miR-223-3p	12662	Chm	0	0	1	1	0	2
mmu-miR-223-3p	12669	Chrm1	1	0	0	1	0	2
mmu-miR-223-3p	12684	Cideb	0	0	1	1	0	2
mmu-miR-223-3p	12686	Elovl3	0	0	1	1	0	2
mmu-miR-223-3p	12723	Clcn1	0	1	0	1	0	2
mmu-miR-223-3p	12728	Clcn5	0	1	0	1	0	2
mmu-miR-223-3p	12740	Cldn4	0	0	1	1	0	2
mmu-miR-223-3p	12741	Cldn5	1	0	0	1	0	2
mmu-miR-223-3p	12745	Clgn	1	0	0	1	0	2
mmu-miR-223-3p	12748	Clk2	0	1	0	1	0	2
mmu-miR-223-3p	12759	Clu	0	0	1	1	0	2
mmu-miR-223-3p	12763	Cmah	1	0	0	1	0	2
mmu-miR-223-3p	12765	Cxcr2	1	0	0	1	0	2
mmu-miR-223-3p	12774	Ccr5	1	0	0	1	0	2
mmu-miR-223-3p	12785	Cnbp	1	0	0	1	0	2
mmu-miR-223-3p	12788	Cnga1	0	0	1	1	0	2
mmu-miR-223-3p	12790	Cnga3	1	0	0	1	0	2
mmu-miR-223-3p	12794	Cnih2	1	0	0	1	0	2
mmu-miR-223-3p	12797	Cnn1	0	0	1	1	0	2
mmu-miR-223-3p	12798	Cnn2	1	0	0	1	0	2
mmu-miR-223-3p	12799	Cnp	1	0	0	1	0	2
mmu-miR-223-3p	12805	Cntn1	0	0	1	1	0	2
mmu-miR-223-3p	12827	Col4a2	0	0	1	1	0	2
mmu-miR-223-3p	12831	Col5a1	0	0	1	1	0	2
mmu-miR-223-3p	12832	Col5a2	1	0	1	0	0	2
mmu-miR-223-3p	12835	Col6a3	0	1	0	1	0	2

mmu-miR-223-3p	12843	Col1a2	1	0	0	1	0	2
mmu-miR-223-3p	12846	Comt	1	0	0	1	0	2
mmu-miR-223-3p	12847	Copa	0	1	0	1	0	2
mmu-miR-223-3p	12870	Cp	1	0	0	1	0	2
mmu-miR-223-3p	12892	Cpox	1	0	0	1	0	2
mmu-miR-223-3p	12902	Cr2	0	0	1	1	0	2
mmu-miR-223-3p	12904	Crabp2	1	0	0	1	0	2
mmu-miR-223-3p	12908	Crat	0	0	1	1	0	2
mmu-miR-223-3p	12916	Crem	1	0	0	1	0	2
mmu-miR-223-3p	12922	Crhr2	1	0	0	1	0	2
mmu-miR-223-3p	12951	Crx	1	0	0	1	0	2
mmu-miR-223-3p	12972	Cryz	0	0	1	1	0	2
mmu-miR-223-3p	12977	Csf1	1	0	0	1	0	2
mmu-miR-223-3p	12978	Csf1r	0	1	1	0	0	2
mmu-miR-223-3p	12981	Csf2	1	0	0	1	0	2
mmu-miR-223-3p	12986	Csf3r	0	0	1	1	0	2
mmu-miR-223-3p	12995	Csnk2a1	0	0	1	1	0	2
mmu-miR-223-3p	13000	Csnk2a2	0	0	1	1	0	2
mmu-miR-223-3p	13002	Dnajc5	1	0	0	1	0	2
mmu-miR-223-3p	13019	Ctf1	0	0	1	1	0	2
mmu-miR-223-3p	13024	Ctla2a	1	0	0	1	0	2
mmu-miR-223-3p	13025	Ctla2b	1	0	0	1	0	2
mmu-miR-223-3p	13030	Ctsb	0	0	1	1	0	2
mmu-miR-223-3p	13033	Ctsd	0	0	1	1	0	2
mmu-miR-223-3p	13047	Cux1	0	1	0	1	0	2
mmu-miR-223-3p	13048	Cux2	1	1	0	0	0	2
mmu-miR-223-3p	13051	Cx3cr1	0	0	1	1	0	2
mmu-miR-223-3p	13078	Cyp1b1	0	0	1	1	0	2
mmu-miR-223-3p	13109	Cyp2j5	0	0	1	1	0	2
mmu-miR-223-3p	13114	Cyp3a16	0	0	1	1	0	2
mmu-miR-223-3p	13121	Cyp51	0	0	1	1	0	2
mmu-miR-223-3p	13124	Cyp8b1	0	0	1	1	0	2
mmu-miR-223-3p	13132	Dab2	0	1	0	1	0	2
mmu-miR-223-3p	13134	Dach1	1	0	0	1	0	2
mmu-miR-223-3p	13136	Cd55	1	0	1	0	0	2
mmu-miR-223-3p	13143	Dapk2	0	0	1	1	0	2
mmu-miR-223-3p	13168	Dbil5	1	0	0	1	0	2
mmu-miR-223-3p	13170	Dbp	0	0	1	1	0	2
mmu-miR-223-3p	13185	Dscr3	1	0	0	1	0	2
mmu-miR-223-3p	13195	Ddc	0	0	1	1	0	2
mmu-miR-223-3p	13197	Gadd45a	1	0	1	0	0	2
mmu-miR-223-3p	13209	Ddx6	1	0	0	1	0	2
mmu-miR-223-3p	13347	Dffa	0	0	1	1	0	2
mmu-miR-223-3p	13360	Dhcr7	1	0	0	1	0	2
mmu-miR-223-3p	13370	Dio1	1	0	0	1	0	2
mmu-miR-223-3p	13394	Dlx4	1	0	0	1	0	2
mmu-miR-223-3p	13404	Dmc1	0	0	1	1	0	2
mmu-miR-223-3p	13411	Dnah11	0	1	0	1	0	2
mmu-miR-223-3p	13427	Dync1i2	0	0	1	1	0	2
mmu-miR-223-3p	13433	Dnmt1	0	1	0	1	0	2
mmu-miR-223-3p	13434	Trdmt1	0	1	0	1	0	2
mmu-miR-223-3p	13447	Doc2b	1	0	0	1	0	2
mmu-miR-223-3p	13480	Dpm1	0	0	1	1	0	2
mmu-miR-223-3p	13483	Dpp6	0	1	0	1	0	2
mmu-miR-223-3p	13492	Drd5	0	0	1	1	0	2

mmu-miR-223-3p	13494	Drg1	1	0	0	1	0	2
mmu-miR-223-3p	13495	Drg2	0	0	1	1	0	2
mmu-miR-223-3p	13506	Dsc2	0	1	0	1	0	2
mmu-miR-223-3p	13511	Dsg2	0	0	1	1	0	2
mmu-miR-223-3p	13512	Dsg3	1	0	1	0	0	2
mmu-miR-223-3p	13531	Usp17la	0	0	1	1	0	2
mmu-miR-223-3p	13543	Dvl2	0	1	0	1	0	2
mmu-miR-223-3p	13591	Ebf1	0	1	0	1	0	2
mmu-miR-223-3p	13612	Edil3	1	0	0	1	0	2
mmu-miR-223-3p	13614	Edn1	1	0	1	0	0	2
mmu-miR-223-3p	13618	Ednrb	1	0	0	1	0	2
mmu-miR-223-3p	13628	Eef1a2	1	0	0	1	0	2
mmu-miR-223-3p	13631	Eef2k	1	0	0	1	0	2
mmu-miR-223-3p	13637	Efna2	1	0	0	1	0	2
mmu-miR-223-3p	13640	Efna5	0	1	0	1	0	2
mmu-miR-223-3p	13642	Efnb2	0	0	1	1	0	2
mmu-miR-223-3p	13653	Egr1	1	0	0	1	0	2
mmu-miR-223-3p	13656	Egr4	0	0	1	1	0	2
mmu-miR-223-3p	13660	Ehd1	0	0	1	1	0	2
mmu-miR-223-3p	13663	Ei24	1	0	1	0	0	2
mmu-miR-223-3p	13666	Eif2ak3	1	0	1	0	0	2
mmu-miR-223-3p	13682	Eif4a2	0	1	0	1	0	2
mmu-miR-223-3p	13688	Eif4ebp2	0	0	1	1	0	2
mmu-miR-223-3p	13713	Elk3	1	0	0	1	0	2
mmu-miR-223-3p	13728	Mark2	0	1	0	1	0	2
mmu-miR-223-3p	13798	En1	1	0	0	1	0	2
mmu-miR-223-3p	13821	Epb4.1l1	1	0	0	1	0	2
mmu-miR-223-3p	13822	Epb4.1l2	0	1	0	1	0	2
mmu-miR-223-3p	13831	Epc1	1	0	0	1	0	2
mmu-miR-223-3p	13836	Epha2	1	0	0	1	0	2
mmu-miR-223-3p	13838	Epha4	0	1	0	1	0	2
mmu-miR-223-3p	13842	Epha8	0	1	1	0	0	2
mmu-miR-223-3p	13845	Ephb3	0	0	1	1	0	2
mmu-miR-223-3p	13855	Epn2	0	0	1	1	0	2
mmu-miR-223-3p	13857	Epor	0	0	1	1	0	2
mmu-miR-223-3p	13858	Eps15	1	0	0	1	0	2
mmu-miR-223-3p	13860	Eps8	1	0	0	1	0	2
mmu-miR-223-3p	13863	Lcn5	0	0	1	1	0	2
mmu-miR-223-3p	13866	Erbb2	0	0	1	1	0	2
mmu-miR-223-3p	13871	Ercc2	0	0	1	1	0	2
mmu-miR-223-3p	13872	Ercc3	0	0	1	1	0	2
mmu-miR-223-3p	13876	Erg	1	0	0	1	0	2
mmu-miR-223-3p	13909	Ces3b	0	0	1	1	0	2
mmu-miR-223-3p	13929	Amz2	0	0	1	1	0	2
mmu-miR-223-3p	13972	Gnb1l	1	0	0	1	0	2
mmu-miR-223-3p	14009	Etv1	1	0	0	1	0	2
mmu-miR-223-3p	14011	Etv6	0	0	1	1	0	2
mmu-miR-223-3p	14013	Mecom	1	0	0	1	0	2
mmu-miR-223-3p	14025	Bcl11a	1	0	0	1	0	2
mmu-miR-223-3p	14028	Evx1	0	0	1	1	0	2
mmu-miR-223-3p	14042	Ext1	0	1	0	1	0	2
mmu-miR-223-3p	14062	F2r	0	0	1	1	0	2
mmu-miR-223-3p	14063	F2rl1	1	0	0	1	0	2
mmu-miR-223-3p	14064	F2rl2	1	0	0	1	0	2
mmu-miR-223-3p	14067	F5	1	0	0	1	0	2

mmu-miR-223-3p	14077	Fabp3	0	0	1	1	0	2
mmu-miR-223-3p	14082	Fadd	0	0	1	1	0	2
mmu-miR-223-3p	14086	Fscn1	1	0	0	1	0	2
mmu-miR-223-3p	14102	Fas	1	0	0	1	0	2
mmu-miR-223-3p	14114	Fbln1	0	0	1	1	0	2
mmu-miR-223-3p	14115	Fbln2	1	0	0	1	0	2
mmu-miR-223-3p	14126	Ms4a2	1	0	0	1	0	2
mmu-miR-223-3p	14128	Fcer2a	1	0	0	1	0	2
mmu-miR-223-3p	14149	Fdxr	0	0	1	1	0	2
mmu-miR-223-3p	14156	Fen1	0	1	1	0	0	2
mmu-miR-223-3p	14161	Fga	1	0	0	1	0	2
mmu-miR-223-3p	14170	Fgf15	1	0	1	0	0	2
mmu-miR-223-3p	14172	Fgf18	1	0	0	1	0	2
mmu-miR-223-3p	14174	Fgf3	0	1	0	1	0	2
mmu-miR-223-3p	14175	Fgf4	1	0	0	1	0	2
mmu-miR-223-3p	14182	Fgfr1	1	0	0	1	0	2
mmu-miR-223-3p	14184	Fgfr3	0	0	1	1	0	2
mmu-miR-223-3p	14191	Fgr	1	0	1	0	0	2
mmu-miR-223-3p	14198	Fhit	1	0	0	1	0	2
mmu-miR-223-3p	14199	Fhl1	1	0	0	1	0	2
mmu-miR-223-3p	14211	Smc2	0	0	1	1	0	2
mmu-miR-223-3p	14219	Ctgf	1	0	1	0	0	2
mmu-miR-223-3p	14225	Fkbp1a	1	0	0	1	0	2
mmu-miR-223-3p	14228	Fkbp4	1	0	0	1	0	2
mmu-miR-223-3p	14232	Fkbp8	1	1	0	0	0	2
mmu-miR-223-3p	14233	Foxi1	0	0	1	1	0	2
mmu-miR-223-3p	14239	Foxs1	1	0	0	1	0	2
mmu-miR-223-3p	14247	Fli1	1	0	0	1	0	2
mmu-miR-223-3p	14254	Flt1	0	0	1	1	0	2
mmu-miR-223-3p	14265	Fmr1	1	0	0	1	0	2
mmu-miR-223-3p	14269	Fnbp1	0	0	1	1	0	2
mmu-miR-223-3p	14281	Fos	0	1	0	1	0	2
mmu-miR-223-3p	14284	Fosl2	0	0	1	1	0	2
mmu-miR-223-3p	14296	Frat1	1	0	0	1	0	2
mmu-miR-223-3p	14302	Frk	1	0	0	1	0	2
mmu-miR-223-3p	14314	Fstl1	0	0	1	1	0	2
mmu-miR-223-3p	14339	Aktip	0	0	1	1	0	2
mmu-miR-223-3p	14367	Fzd5	1	0	0	1	0	2
mmu-miR-223-3p	14368	Fzd6	0	0	1	1	0	2
mmu-miR-223-3p	14370	Fzd8	1	0	0	0	1	2
mmu-miR-223-3p	14371	Fzd9	0	0	1	1	0	2
mmu-miR-223-3p	14377	G6pc	1	0	0	1	0	2
mmu-miR-223-3p	14388	Gab1	0	0	1	1	0	2
mmu-miR-223-3p	14391	Gabpb1	1	0	0	1	0	2
mmu-miR-223-3p	14394	Gabra1	1	0	0	1	0	2
mmu-miR-223-3p	14401	Gabrb2	0	0	1	1	0	2
mmu-miR-223-3p	14417	Gad2	1	0	0	1	0	2
mmu-miR-223-3p	14425	Galnt3	0	0	1	1	0	2
mmu-miR-223-3p	14450	Gart	1	0	0	1	0	2
mmu-miR-223-3p	14453	Gas2	1	0	0	1	0	2
mmu-miR-223-3p	14457	Gas7	1	0	0	1	0	2
mmu-miR-223-3p	14461	Gata2	1	0	0	1	0	2
mmu-miR-223-3p	14472	Gbx2	1	0	0	1	0	2
mmu-miR-223-3p	14533	Bloc1s1	0	1	0	1	0	2
mmu-miR-223-3p	14537	Gcnt1	1	0	0	1	0	2

mmu-miR-223-3p	14562	Gdf3	1	0	0	1	0	2
mmu-miR-223-3p	14571	Gpd2	1	0	0	1	0	2
mmu-miR-223-3p	14573	Gdnf	1	0	0	1	0	2
mmu-miR-223-3p	14585	Gfra1	0	1	0	1	0	2
mmu-miR-223-3p	14586	Gfra2	0	1	0	1	0	2
mmu-miR-223-3p	14600	Ghr	0	0	1	1	0	2
mmu-miR-223-3p	14605	Tsc22d3	0	1	0	1	0	2
mmu-miR-223-3p	14608	Gpr83	0	0	1	1	0	2
mmu-miR-223-3p	14611	Gja3	1	0	0	1	0	2
mmu-miR-223-3p	14613	Gja5	0	0	1	1	0	2
mmu-miR-223-3p	14615	Gjc1	0	0	1	1	0	2
mmu-miR-223-3p	14619	Gjb2	1	0	0	1	0	2
mmu-miR-223-3p	14628	Ostm1	1	0	0	1	0	2
mmu-miR-223-3p	14629	Gclc	0	0	1	1	0	2
mmu-miR-223-3p	14660	Gls	1	0	0	1	0	2
mmu-miR-223-3p	14677	Gnai1	0	0	1	1	0	2
mmu-miR-223-3p	14681	Gnao1	0	1	0	1	0	2
mmu-miR-223-3p	14682	Gnaq	1	0	0	1	0	2
mmu-miR-223-3p	14694	Gnb2l1	0	0	1	1	0	2
mmu-miR-223-3p	14699	Gngt1	1	0	0	1	0	2
mmu-miR-223-3p	14700	Gng10	1	0	0	1	0	2
mmu-miR-223-3p	14708	Gng7	1	0	0	1	0	2
mmu-miR-223-3p	14712	Gnpat	0	0	1	1	0	2
mmu-miR-223-3p	14718	Got1	0	0	1	1	0	2
mmu-miR-223-3p	14719	Got2	1	0	0	1	0	2
mmu-miR-223-3p	14724	Gp1bb	1	0	0	1	0	2
mmu-miR-223-3p	14748	Gpr3	0	0	1	1	0	2
mmu-miR-223-3p	14751	Gpi1	0	0	1	1	0	2
mmu-miR-223-3p	14755	Pigq	0	0	1	1	0	2
mmu-miR-223-3p	14756	Gpld1	0	0	1	1	0	2
mmu-miR-223-3p	14763	Gpr37	0	0	1	1	0	2
mmu-miR-223-3p	14764	Gpr44	0	0	1	1	0	2
mmu-miR-223-3p	14789	Leprel2	0	0	1	1	0	2
mmu-miR-223-3p	14797	Aes	1	0	0	1	0	2
mmu-miR-223-3p	14802	Gria4	1	0	0	1	0	2
mmu-miR-223-3p	14805	Grik1	1	0	0	1	0	2
mmu-miR-223-3p	14806	Grik2	1	0	0	1	0	2
mmu-miR-223-3p	14809	Grik5	0	1	1	0	0	2
mmu-miR-223-3p	14829	Grpr	1	0	0	1	0	2
mmu-miR-223-3p	14842	Gsx1	0	0	1	1	0	2
mmu-miR-223-3p	14859	Gsta3	1	0	0	1	0	2
mmu-miR-223-3p	14867	Gstm6	0	0	1	1	0	2
mmu-miR-223-3p	14884	Gtf2h1	1	0	0	1	0	2
mmu-miR-223-3p	14904	Gtpbp1	0	1	1	0	0	2
mmu-miR-223-3p	14923	Guk1	0	0	1	1	0	2
mmu-miR-223-3p	14934	Gypa	1	0	1	0	0	2
mmu-miR-223-3p	14936	Gys1	1	0	0	1	0	2
mmu-miR-223-3p	15006	H2-Q1	0	0	1	1	0	2
mmu-miR-223-3p	15040	H2-T23	0	0	1	1	0	2
mmu-miR-223-3p	15043	H2-T3	1	0	0	1	0	2
mmu-miR-223-3p	15078	H3f3a	0	1	0	1	0	2
mmu-miR-223-3p	15101	H60a	1	0	0	1	0	2
mmu-miR-223-3p	15110	Hand1	0	0	1	1	0	2
mmu-miR-223-3p	15112	Hao1	0	0	1	1	0	2
mmu-miR-223-3p	15114	Hap1	1	0	0	1	0	2

mmu-miR-223-3p	15159	Hccs	0	0	1	1	0	2
mmu-miR-223-3p	15160	Serpind1	0	0	1	1	0	2
mmu-miR-223-3p	15166	Hcn2	0	1	1	0	0	2
mmu-miR-223-3p	15183	Hdac3	0	0	1	1	0	2
mmu-miR-223-3p	15193	Hdgfrp2	0	0	1	1	0	2
mmu-miR-223-3p	15199	Hebp1	1	0	1	0	0	2
mmu-miR-223-3p	15201	Hells	0	1	0	1	0	2
mmu-miR-223-3p	15204	Herc2	0	0	1	1	0	2
mmu-miR-223-3p	15206	Hes2	0	0	1	1	0	2
mmu-miR-223-3p	15214	Hey2	0	0	1	1	0	2
mmu-miR-223-3p	15218	Foxn1	1	0	0	1	0	2
mmu-miR-223-3p	15227	Foxf1	1	0	0	1	0	2
mmu-miR-223-3p	15251	Hif1a	1	0	0	1	0	2
mmu-miR-223-3p	15357	Hmgcr	0	0	1	1	0	2
mmu-miR-223-3p	15361	Hmga1	1	0	0	1	0	2
mmu-miR-223-3p	15364	Hmga2	1	0	0	1	0	2
mmu-miR-223-3p	15368	Hmox1	0	0	1	1	0	2
mmu-miR-223-3p	15370	Nr4a1	0	0	1	1	0	2
mmu-miR-223-3p	15374	Hn1	0	0	1	1	0	2
mmu-miR-223-3p	15381	Hnrnpc	1	0	0	1	0	2
mmu-miR-223-3p	15396	Hoxa11	0	0	1	1	0	2
mmu-miR-223-3p	15400	Hoxa3	1	0	0	1	0	2
mmu-miR-223-3p	15404	Hoxa7	0	1	0	1	0	2
mmu-miR-223-3p	15405	Hoxa9	0	1	0	1	0	2
mmu-miR-223-3p	15410	Hoxb3	1	0	0	1	0	2
mmu-miR-223-3p	15412	Hoxb4	0	1	0	1	0	2
mmu-miR-223-3p	15423	Hoxc4	0	0	1	1	0	2
mmu-miR-223-3p	15434	Hoxd3	0	1	0	1	0	2
mmu-miR-223-3p	15436	Hoxd4	0	1	0	1	0	2
mmu-miR-223-3p	15438	Hoxd9	0	0	1	1	0	2
mmu-miR-223-3p	15445	Hpd	0	0	1	1	0	2
mmu-miR-223-3p	15461	Hras1	1	0	0	1	0	2
mmu-miR-223-3p	15465	Hrh1	0	1	0	1	0	2
mmu-miR-223-3p	15469	Prmt1	0	0	1	1	0	2
mmu-miR-223-3p	15478	Hs3st3a1	1	0	0	1	0	2
mmu-miR-223-3p	15493	Hsd3b2	0	0	1	1	0	2
mmu-miR-223-3p	15494	Hsd3b3	0	0	1	1	0	2
mmu-miR-223-3p	15497	Hsd3b6	0	0	1	1	0	2
mmu-miR-223-3p	15511	Hspa1b	1	0	0	1	0	2
mmu-miR-223-3p	15526	Hspa9	0	0	1	1	0	2
mmu-miR-223-3p	15530	Hspg2	1	1	0	0	0	2
mmu-miR-223-3p	15559	Htr2b	1	0	0	1	0	2
mmu-miR-223-3p	15566	Htr7	1	0	0	1	0	2
mmu-miR-223-3p	15569	Elavl2	1	0	0	1	0	2
mmu-miR-223-3p	15572	Elavl4	1	0	0	1	0	2
mmu-miR-223-3p	15874	Iapp	1	0	0	1	0	2
mmu-miR-223-3p	15891	Ibsp	1	0	0	1	0	2
mmu-miR-223-3p	15925	Ide	0	0	1	1	0	2
mmu-miR-223-3p	15932	Idua	1	0	1	0	0	2
mmu-miR-223-3p	15957	Ifit1	0	0	1	1	0	2
mmu-miR-223-3p	15959	Ifit3	1	1	0	0	0	2
mmu-miR-223-3p	15976	Ifnar2	0	0	1	1	0	2
mmu-miR-223-3p	15978	Ifng	0	0	1	1	0	2
mmu-miR-223-3p	16068	Il18bp	0	0	1	1	0	2
mmu-miR-223-3p	16069	Igj	1	0	0	1	0	2

mmu-miR-223-3p	16147	Ihh	0	0	1	1	0	2
mmu-miR-223-3p	16151	Ikbkg	0	0	1	1	0	2
mmu-miR-223-3p	16159	Il12a	0	0	1	1	0	2
mmu-miR-223-3p	16163	Il13	1	0	0	1	0	2
mmu-miR-223-3p	16171	Il17a	1	0	0	1	0	2
mmu-miR-223-3p	16180	Il1rap	1	0	0	1	0	2
mmu-miR-223-3p	16181	Il1rn	1	0	0	1	0	2
mmu-miR-223-3p	16192	Il5ra	0	0	1	1	0	2
mmu-miR-223-3p	16194	Il6ra	0	0	1	1	0	2
mmu-miR-223-3p	16206	Lrig1	1	0	0	1	0	2
mmu-miR-223-3p	16328	Cep250	0	0	1	1	0	2
mmu-miR-223-3p	16331	Inpp5d	1	0	0	1	0	2
mmu-miR-223-3p	16348	Invs	1	0	0	1	0	2
mmu-miR-223-3p	16391	Irf9	0	0	1	1	0	2
mmu-miR-223-3p	16392	Isl1	1	0	0	1	0	2
mmu-miR-223-3p	16398	Itga2	1	0	1	0	0	2
mmu-miR-223-3p	16401	Itga4	1	0	0	1	0	2
mmu-miR-223-3p	16402	Itga5	0	1	1	0	0	2
mmu-miR-223-3p	16403	Itga6	0	0	1	1	0	2
mmu-miR-223-3p	16416	Itgb3	0	0	1	1	0	2
mmu-miR-223-3p	16429	Itln1	0	1	1	0	0	2
mmu-miR-223-3p	16451	Jak1	0	0	1	1	0	2
mmu-miR-223-3p	16469	Jrk	1	0	0	1	0	2
mmu-miR-223-3p	16476	Jun	1	0	0	1	0	2
mmu-miR-223-3p	16480	Jup	1	0	0	1	0	2
mmu-miR-223-3p	16493	Kcna5	0	0	1	1	0	2
mmu-miR-223-3p	16494	Kcna6	1	0	0	1	0	2
mmu-miR-223-3p	16497	Kcnab1	1	0	0	1	0	2
mmu-miR-223-3p	16498	Kcnab2	1	0	0	1	0	2
mmu-miR-223-3p	16500	Kcnb1	0	0	1	1	0	2
mmu-miR-223-3p	16512	Kcnh3	0	0	1	1	0	2
mmu-miR-223-3p	16514	Kcnj11	1	0	0	1	0	2
mmu-miR-223-3p	16515	Kcnj12	1	0	0	1	0	2
mmu-miR-223-3p	16516	Kcnj15	1	0	0	1	0	2
mmu-miR-223-3p	16517	Kcnj16	1	0	0	1	0	2
mmu-miR-223-3p	16520	Kcnj4	0	0	1	1	0	2
mmu-miR-223-3p	16524	Kcnj9	0	0	1	1	0	2
mmu-miR-223-3p	16525	Kcnk1	1	0	0	1	0	2
mmu-miR-223-3p	16527	Kcnk3	0	0	1	1	0	2
mmu-miR-223-3p	16538	Kcns1	0	0	1	1	0	2
mmu-miR-223-3p	16543	Mdfic	1	0	0	1	0	2
mmu-miR-223-3p	16545	Kera	1	0	1	0	0	2
mmu-miR-223-3p	16549	Khsrp	1	0	0	1	0	2
mmu-miR-223-3p	16569	Kif3b	0	0	1	1	0	2
mmu-miR-223-3p	16572	Kif5a	0	0	1	1	0	2
mmu-miR-223-3p	16589	Uhmk1	0	1	0	1	0	2
mmu-miR-223-3p	16634	Klra3	1	0	0	1	0	2
mmu-miR-223-3p	16699	Krtap13	1	0	0	1	0	2
mmu-miR-223-3p	16704	Krtap19-5	0	0	1	1	0	2
mmu-miR-223-3p	16782	Lamc2	1	0	0	1	0	2
mmu-miR-223-3p	16783	Lamp1	1	0	0	1	0	2
mmu-miR-223-3p	16792	Laptm5	1	0	0	1	0	2
mmu-miR-223-3p	16796	Lasp1	0	0	1	1	0	2
mmu-miR-223-3p	16803	Lbp	0	0	1	1	0	2
mmu-miR-223-3p	16821	Lcn4	0	0	1	1	0	2

mmu-miR-223-3p	16833	Ldhc	0	0	1	1	0	2
mmu-miR-223-3p	16835	Ldlr	0	0	1	1	0	2
mmu-miR-223-3p	16872	Lhx4	0	1	0	1	0	2
mmu-miR-223-3p	16886	Limk2	1	0	0	1	0	2
mmu-miR-223-3p	16889	Lipa	1	0	0	1	0	2
mmu-miR-223-3p	16891	Lipg	0	0	1	1	0	2
mmu-miR-223-3p	16956	Lpl	1	0	0	1	0	2
mmu-miR-223-3p	16977	Lrrc23	0	0	1	1	0	2
mmu-miR-223-3p	16978	Lrrfip1	1	0	0	1	0	2
mmu-miR-223-3p	16981	Lrrn3	0	0	1	1	0	2
mmu-miR-223-3p	17057	Klrb1a	1	0	0	1	0	2
mmu-miR-223-3p	17075	Epcam	0	0	1	1	0	2
mmu-miR-223-3p	17116	Mab21l1	1	0	0	1	0	2
mmu-miR-223-3p	17119	Mxd1	0	0	1	1	0	2
mmu-miR-223-3p	17133	Maff	1	0	1	0	0	2
mmu-miR-223-3p	17134	Mafg	0	1	0	1	0	2
mmu-miR-223-3p	17135	Mafk	0	0	1	1	0	2
mmu-miR-223-3p	17138	Magea2	1	0	0	1	0	2
mmu-miR-223-3p	17152	Mak	1	0	0	1	0	2
mmu-miR-223-3p	17161	Maoa	1	0	0	1	0	2
mmu-miR-223-3p	17168	Nprl3	0	1	0	1	0	2
mmu-miR-223-3p	17174	Masp1	0	0	1	1	0	2
mmu-miR-223-3p	17175	Masp2	0	0	1	1	0	2
mmu-miR-223-3p	17182	Matn3	1	0	0	1	0	2
mmu-miR-223-3p	17188	Maz	0	0	1	1	0	2
mmu-miR-223-3p	17189	Mb	0	0	1	1	0	2
mmu-miR-223-3p	17190	Mbd1	1	0	0	1	0	2
mmu-miR-223-3p	17200	Mc2r	0	1	1	0	0	2
mmu-miR-223-3p	17202	Mc4r	0	0	1	1	0	2
mmu-miR-223-3p	17210	Mcl1	1	0	0	1	0	2
mmu-miR-223-3p	17217	Mcm4	0	0	1	1	0	2
mmu-miR-223-3p	17222	Anapc1	1	0	0	1	0	2
mmu-miR-223-3p	17237	Mgrn1	0	0	1	1	0	2
mmu-miR-223-3p	17254	Slc3a2	1	0	0	1	0	2
mmu-miR-223-3p	17268	Meis1	0	1	0	1	0	2
mmu-miR-223-3p	17286	Meox2	1	0	0	1	0	2
mmu-miR-223-3p	17287	Mep1a	0	0	1	1	0	2
mmu-miR-223-3p	17294	Mest	1	0	0	1	0	2
mmu-miR-223-3p	17330	Minpp1	1	0	1	0	0	2
mmu-miR-223-3p	17339	Mip	1	0	0	1	0	2
mmu-miR-223-3p	17344	Pias2	0	0	1	1	0	2
mmu-miR-223-3p	17346	Mknk1	0	0	1	1	0	2
mmu-miR-223-3p	17395	Mmp9	0	0	1	1	0	2
mmu-miR-223-3p	17433	Mobp	1	0	1	0	0	2
mmu-miR-223-3p	17436	Me1	1	0	0	1	0	2
mmu-miR-223-3p	17444	Grap2	0	0	1	1	0	2
mmu-miR-223-3p	17449	Mdh1	0	0	1	1	0	2
mmu-miR-223-3p	17470	Cd200	1	0	0	1	0	2
mmu-miR-223-3p	17472	Gbp4	0	0	1	1	0	2
mmu-miR-223-3p	17475	Mpdz	0	0	1	1	0	2
mmu-miR-223-3p	17476	Mpeg1	0	0	1	1	0	2
mmu-miR-223-3p	17524	Mpp1	0	0	1	1	0	2
mmu-miR-223-3p	17527	Mpv17	0	0	1	1	0	2
mmu-miR-223-3p	17536	Meis2	1	0	0	1	0	2
mmu-miR-223-3p	17684	Cited2	1	0	0	1	0	2

mmu-miR-223-3p	17688	Msh6	0	0	1	1	0	2
mmu-miR-223-3p	17690	Msi1	0	0	1	1	0	2
mmu-miR-223-3p	17691	Sik1	1	0	0	1	0	2
mmu-miR-223-3p	17698	Msn	0	1	0	1	0	2
mmu-miR-223-3p	17713	Grpel1	0	0	1	1	0	2
mmu-miR-223-3p	17714	Grpel2	0	0	1	1	0	2
mmu-miR-223-3p	17762	Mapt	0	1	0	1	0	2
mmu-miR-223-3p	17765	Mtf2	1	0	0	1	0	2
mmu-miR-223-3p	17772	Mtm1	0	0	1	1	0	2
mmu-miR-223-3p	17828	Bloc1s5	0	0	1	1	0	2
mmu-miR-223-3p	17833	Muc5ac	1	0	0	1	0	2
mmu-miR-223-3p	17855	Mvk	1	0	0	1	0	2
mmu-miR-223-3p	17864	Mybl1	0	1	0	1	0	2
mmu-miR-223-3p	17877	Myf5	0	0	1	1	0	2
mmu-miR-223-3p	17886	Myh9	1	0	0	1	0	2
mmu-miR-223-3p	17897	Myl3	0	1	0	1	0	2
mmu-miR-223-3p	17901	Myl1	1	0	0	1	0	2
mmu-miR-223-3p	17909	Myo10	0	1	0	1	0	2
mmu-miR-223-3p	17921	Myo7a	0	0	1	1	0	2
mmu-miR-223-3p	17925	Myo9b	0	0	1	1	0	2
mmu-miR-223-3p	17928	Myog	0	0	1	1	0	2
mmu-miR-223-3p	17933	Myt1l	1	0	0	1	0	2
mmu-miR-223-3p	17940	Naip1	1	0	0	1	0	2
mmu-miR-223-3p	17955	Nap1l4	1	0	0	1	0	2
mmu-miR-223-3p	17961	Nat2	1	0	0	1	0	2
mmu-miR-223-3p	17965	Nbl1	0	0	1	1	0	2
mmu-miR-223-3p	17967	Ncam1	1	0	0	1	0	2
mmu-miR-223-3p	17970	Ncf2	1	0	0	1	0	2
mmu-miR-223-3p	17973	Nck1	0	0	1	1	0	2
mmu-miR-223-3p	18003	Nedd9	1	0	0	1	0	2
mmu-miR-223-3p	18011	Neurl1a	1	0	0	1	0	2
mmu-miR-223-3p	18012	Neurod1	0	0	1	1	0	2
mmu-miR-223-3p	18013	Neurod2	1	0	0	1	0	2
mmu-miR-223-3p	18020	Nfatc2ip	1	0	0	1	0	2
mmu-miR-223-3p	18021	Nfatc3	0	1	0	1	0	2
mmu-miR-223-3p	18025	Nfe2l3	1	0	0	1	0	2
mmu-miR-223-3p	18032	Nfix	1	0	0	1	0	2
mmu-miR-223-3p	18040	Nefm	0	1	0	1	0	2
mmu-miR-223-3p	18073	Nid1	1	0	0	1	0	2
mmu-miR-223-3p	18088	Nkx2-2	0	1	0	1	0	2
mmu-miR-223-3p	18095	Nkx3-1	1	0	0	1	0	2
mmu-miR-223-3p	18111	Nnat	0	1	0	1	0	2
mmu-miR-223-3p	18115	Nnt	0	0	1	1	0	2
mmu-miR-223-3p	18128	Notch1	0	0	1	1	0	2
mmu-miR-223-3p	18133	Nov	1	0	0	1	0	2
mmu-miR-223-3p	18141	Nup50	0	0	1	1	0	2
mmu-miR-223-3p	18155	Pnoc	0	0	1	1	0	2
mmu-miR-223-3p	18163	Ctnnd2	1	0	1	0	0	2
mmu-miR-223-3p	18169	Npy6r	0	0	1	1	0	2
mmu-miR-223-3p	18174	Slc11a2	0	0	1	1	0	2
mmu-miR-223-3p	18185	Nrl	1	0	0	1	0	2
mmu-miR-223-3p	18187	Nrp2	1	0	0	1	0	2
mmu-miR-223-3p	18190	Nrxn2	0	1	0	1	0	2
mmu-miR-223-3p	18208	Ntn1	0	0	1	1	0	2
mmu-miR-223-3p	18213	Ntrk3	1	0	0	1	0	2

mmu-miR-223-3p	18216	Ntsr1	0	1	0	1	0	2
mmu-miR-223-3p	18222	Numb	0	1	0	1	0	2
mmu-miR-223-3p	18226	Nup62	0	0	1	1	0	2
mmu-miR-223-3p	18227	Nr4a2	0	1	0	1	0	2
mmu-miR-223-3p	18231	Nxph1	1	0	0	1	0	2
mmu-miR-223-3p	18286	Odf2	0	0	1	1	0	2
mmu-miR-223-3p	18300	Oit1	0	0	1	1	0	2
mmu-miR-223-3p	18343	Olfr44	0	0	1	1	0	2
mmu-miR-223-3p	18387	Oprk1	1	0	0	1	0	2
mmu-miR-223-3p	18391	Sigmar1	0	0	1	1	0	2
mmu-miR-223-3p	18399	Slc22a6	1	0	0	1	0	2
mmu-miR-223-3p	18408	Slc25a15	0	0	1	1	0	2
mmu-miR-223-3p	18414	Osmr	1	0	0	1	0	2
mmu-miR-223-3p	18424	Otx2	1	0	0	1	0	2
mmu-miR-223-3p	18426	Ovol1	0	0	1	1	0	2
mmu-miR-223-3p	18436	P2rx1	0	0	1	1	0	2
mmu-miR-223-3p	18441	P2ry1	1	0	0	1	0	2
mmu-miR-223-3p	18442	P2ry2	1	0	1	0	0	2
mmu-miR-223-3p	18483	Palm	0	0	1	1	0	2
mmu-miR-223-3p	18505	Pax3	0	0	1	1	0	2
mmu-miR-223-3p	18508	Pax6	0	1	0	1	0	2
mmu-miR-223-3p	18510	Pax8	1	0	1	0	0	2
mmu-miR-223-3p	18536	Pcm1	1	0	0	1	0	2
mmu-miR-223-3p	18537	Pcmt1	1	0	1	0	0	2
mmu-miR-223-3p	18549	Pcsk2	1	0	0	1	0	2
mmu-miR-223-3p	18550	Furin	0	1	0	1	0	2
mmu-miR-223-3p	18552	Pcsk5	1	0	0	1	0	2
mmu-miR-223-3p	18553	Pcsk6	0	1	0	1	0	2
mmu-miR-223-3p	18555	Cdk16	0	0	1	1	0	2
mmu-miR-223-3p	18583	Pde7a	0	1	0	1	0	2
mmu-miR-223-3p	18597	Pdha1	0	0	1	1	0	2
mmu-miR-223-3p	18605	Enpp1	1	1	0	0	0	2
mmu-miR-223-3p	18612	Etv4	0	0	1	1	0	2
mmu-miR-223-3p	18634	Pex7	0	0	1	1	0	2
mmu-miR-223-3p	18640	Pfkfb2	0	0	1	1	0	2
mmu-miR-223-3p	18646	Prf1	0	0	1	1	0	2
mmu-miR-223-3p	18648	Pgam1	0	1	1	0	0	2
mmu-miR-223-3p	18674	Slc25a3	0	1	0	1	0	2
mmu-miR-223-3p	18685	Phtf1	0	1	0	1	0	2
mmu-miR-223-3p	18700	Piga	1	0	0	1	0	2
mmu-miR-223-3p	18705	Pik3c2g	1	0	0	1	0	2
mmu-miR-223-3p	18708	Pik3r1	1	0	0	1	0	2
mmu-miR-223-3p	18716	Pip	0	0	1	1	0	2
mmu-miR-223-3p	18717	Pip5k1c	0	0	1	1	0	2
mmu-miR-223-3p	18762	Prkcz	0	1	0	1	0	2
mmu-miR-223-3p	18764	Pkd2	1	0	0	1	0	2
mmu-miR-223-3p	18772	Pkp1	0	0	1	1	0	2
mmu-miR-223-3p	18777	Lypla1	0	0	1	1	0	2
mmu-miR-223-3p	18779	Pla2r1	1	0	0	1	0	2
mmu-miR-223-3p	18798	Plcb4	1	1	0	0	0	2
mmu-miR-223-3p	18805	Pld1	0	1	0	1	0	2
mmu-miR-223-3p	18822	Plod1	0	0	1	1	0	2
mmu-miR-223-3p	18823	Plp1	0	1	0	1	0	2
mmu-miR-223-3p	18844	Plxna1	1	0	0	1	0	2
mmu-miR-223-3p	18858	Pmp22	1	0	0	1	0	2

mmu-miR-223-3p	18861	Pms2	1	0	0	1	0	2
mmu-miR-223-3p	18933	Prrx1	1	0	0	1	0	2
mmu-miR-223-3p	18968	Pola1	0	0	1	1	0	2
mmu-miR-223-3p	18975	Polg	0	1	0	1	0	2
mmu-miR-223-3p	18984	Por	0	0	1	1	0	2
mmu-miR-223-3p	18985	Pou2af1	0	0	1	1	0	2
mmu-miR-223-3p	18988	Pou2f3	1	0	0	1	0	2
mmu-miR-223-3p	18993	Pou3f3	0	1	1	0	0	2
mmu-miR-223-3p	19009	Pou6f1	0	0	1	1	0	2
mmu-miR-223-3p	19017	Ppargc1a	0	1	0	1	0	2
mmu-miR-223-3p	19052	Ppp2ca	0	1	0	1	0	2
mmu-miR-223-3p	19053	Ppp2cb	0	0	1	1	0	2
mmu-miR-223-3p	19055	Ppp3ca	1	1	0	0	0	2
mmu-miR-223-3p	19056	Ppp3cb	0	0	1	1	0	2
mmu-miR-223-3p	19058	Ppp3r1	0	0	1	1	0	2
mmu-miR-223-3p	19062	Inpp5k	0	0	1	1	0	2
mmu-miR-223-3p	19063	Ppt1	1	0	0	1	0	2
mmu-miR-223-3p	19090	Prkdc	0	1	0	1	0	2
mmu-miR-223-3p	19092	Prkg2	1	0	0	1	0	2
mmu-miR-223-3p	19094	Mapk11	1	0	1	0	0	2
mmu-miR-223-3p	19099	Mapk8ip1	0	1	0	1	0	2
mmu-miR-223-3p	19109	Prl	1	0	0	1	0	2
mmu-miR-223-3p	19125	Prodh	0	0	1	1	0	2
mmu-miR-223-3p	19126	Prom1	1	0	0	1	0	2
mmu-miR-223-3p	19130	Prox1	0	0	1	1	0	2
mmu-miR-223-3p	19133	Prph2	0	0	1	1	0	2
mmu-miR-223-3p	19134	Prpf4b	0	0	1	1	0	2
mmu-miR-223-3p	19143	St14	0	0	1	1	0	2
mmu-miR-223-3p	19192	Psme3	0	0	1	1	0	2
mmu-miR-223-3p	19204	Ptafr	0	0	1	1	0	2
mmu-miR-223-3p	19208	Ptcra	0	0	1	1	0	2
mmu-miR-223-3p	19227	Pthlh	1	0	0	1	0	2
mmu-miR-223-3p	19230	Twf1	1	0	0	1	0	2
mmu-miR-223-3p	19255	Ptpn2	1	0	0	1	0	2
mmu-miR-223-3p	19259	Ptpn5	0	0	1	1	0	2
mmu-miR-223-3p	19266	Ptprd	0	1	0	1	0	2
mmu-miR-223-3p	19270	Ptprg	1	0	0	1	0	2
mmu-miR-223-3p	19273	Ptpru	0	1	0	1	0	2
mmu-miR-223-3p	19277	Ptpro	1	0	0	1	0	2
mmu-miR-223-3p	19279	Ptprr	0	0	1	1	0	2
mmu-miR-223-3p	19283	Ptprz1	0	0	1	1	0	2
mmu-miR-223-3p	19285	Ptrf	0	0	1	1	0	2
mmu-miR-223-3p	19286	Pts	0	0	1	1	0	2
mmu-miR-223-3p	19290	Pura	1	1	0	0	0	2
mmu-miR-223-3p	19305	Pex5	1	0	0	1	0	2
mmu-miR-223-3p	19330	Rab18	1	0	0	1	0	2
mmu-miR-223-3p	19335	Rab23	0	0	1	1	0	2
mmu-miR-223-3p	19338	Rab33b	1	0	0	1	0	2
mmu-miR-223-3p	19346	Rab6a	0	0	1	1	0	2
mmu-miR-223-3p	19347	Dennd5a	0	0	1	1	0	2
mmu-miR-223-3p	19352	Rabggtb	1	0	0	1	0	2
mmu-miR-223-3p	19354	Rac2	0	1	0	1	0	2
mmu-miR-223-3p	19357	Rad21	0	1	0	1	0	2
mmu-miR-223-3p	19359	Rad23b	1	0	0	1	0	2
mmu-miR-223-3p	19364	Rad51d	0	0	1	1	0	2

mmu-miR-223-3p	19376	Rab34	0	0	1	1	0	2
mmu-miR-223-3p	19414	Rasa3	1	0	0	1	0	2
mmu-miR-223-3p	19415	Rasal1	0	0	1	1	0	2
mmu-miR-223-3p	19416	Rasd1	1	0	0	1	0	2
mmu-miR-223-3p	19418	Rasgrf2	1	0	0	1	0	2
mmu-miR-223-3p	19651	Rbl2	0	0	1	1	0	2
mmu-miR-223-3p	19652	Rbm3	0	0	1	1	0	2
mmu-miR-223-3p	19655	Rbmx	1	0	0	1	0	2
mmu-miR-223-3p	19662	Rbp4	0	0	1	1	0	2
mmu-miR-223-3p	19663	Rbpms	0	1	0	1	0	2
mmu-miR-223-3p	19671	Rce1	0	0	1	1	0	2
mmu-miR-223-3p	19679	Pitpnm2	0	0	1	1	0	2
mmu-miR-223-3p	19684	Rdx	0	1	0	1	0	2
mmu-miR-223-3p	19697	Rela	1	0	0	1	0	2
mmu-miR-223-3p	19713	Ret	0	0	1	1	0	2
mmu-miR-223-3p	19714	Rev3l	1	1	0	0	0	2
mmu-miR-223-3p	19720	Trim27	1	0	0	1	0	2
mmu-miR-223-3p	19726	Rfx3	1	0	0	1	0	2
mmu-miR-223-3p	19731	Rgl1	0	0	1	1	0	2
mmu-miR-223-3p	19733	Rgn	1	0	0	1	0	2
mmu-miR-223-3p	19734	Rgs16	1	0	0	1	0	2
mmu-miR-223-3p	19744	Rheb	1	0	0	1	0	2
mmu-miR-223-3p	19762	Rit2	0	0	1	1	0	2
mmu-miR-223-3p	19886	Ros1	0	1	0	1	0	2
mmu-miR-223-3p	19892	Rpe65	0	0	1	1	0	2
mmu-miR-223-3p	19895	Rpia	1	0	0	1	0	2
mmu-miR-223-3p	20014	Rpn2	0	0	1	1	0	2
mmu-miR-223-3p	20018	Polr1d	0	0	1	1	0	2
mmu-miR-223-3p	20021	Polr2c	0	0	1	1	0	2
mmu-miR-223-3p	20024	Sub1	1	0	0	1	0	2
mmu-miR-223-3p	20028	Pdc	1	0	0	1	0	2
mmu-miR-223-3p	20090	Rps29	0	0	1	1	0	2
mmu-miR-223-3p	20111	Rps6ka1	1	0	0	1	0	2
mmu-miR-223-3p	20128	Trim30a	1	0	0	1	0	2
mmu-miR-223-3p	20133	Rrm1	1	0	0	1	0	2
mmu-miR-223-3p	20148	Dhrs3	1	0	0	1	0	2
mmu-miR-223-3p	20182	Rxrb	0	1	0	1	0	2
mmu-miR-223-3p	20186	Nr1h4	1	0	0	1	0	2
mmu-miR-223-3p	20191	Ryr2	1	0	0	1	0	2
mmu-miR-223-3p	20192	Ryr3	1	0	0	1	0	2
mmu-miR-223-3p	20224	Sar1a	1	0	0	1	0	2
mmu-miR-223-3p	20226	Sars	1	0	0	1	0	2
mmu-miR-223-3p	20227	Sart1	0	0	1	1	0	2
mmu-miR-223-3p	20231	Nkx1-2	0	1	1	0	0	2
mmu-miR-223-3p	20274	Scn9a	0	0	1	1	0	2
mmu-miR-223-3p	20286	Zc3h7b	1	0	0	1	0	2
mmu-miR-223-3p	20292	Ccl11	0	0	1	1	0	2
mmu-miR-223-3p	20297	Ccl20	1	0	0	1	0	2
mmu-miR-223-3p	20299	Ccl22	0	0	1	1	0	2
mmu-miR-223-3p	20311	Cxcl5	1	0	0	1	0	2
mmu-miR-223-3p	20333	Sec22b	1	0	0	1	0	2
mmu-miR-223-3p	20338	Sel1l	0	1	0	1	0	2
mmu-miR-223-3p	20351	Sema4a	1	0	0	1	0	2
mmu-miR-223-3p	20359	Sema6b	0	0	1	1	0	2
mmu-miR-223-3p	20365	Serf1	0	0	1	1	0	2

mmu-miR-223-3p	20370	Sez6	0	0	1	1	0	2
mmu-miR-223-3p	20371	Foxp3	0	0	1	1	0	2
mmu-miR-223-3p	20377	Sfrp1	0	0	1	1	0	2
mmu-miR-223-3p	20387	Sftpa1	0	0	1	1	0	2
mmu-miR-223-3p	20392	Sgce	0	1	0	1	0	2
mmu-miR-223-3p	20393	Sgk1	0	0	1	1	0	2
mmu-miR-223-3p	20397	Sgpl1	1	0	0	1	0	2
mmu-miR-223-3p	20405	Sh3gl1	0	0	1	1	0	2
mmu-miR-223-3p	20409	Ostf1	0	1	0	1	0	2
mmu-miR-223-3p	20410	Sorbs3	0	0	1	1	0	2
mmu-miR-223-3p	20429	Shox2	0	1	0	1	0	2
mmu-miR-223-3p	20439	Siah2	0	0	1	1	0	2
mmu-miR-223-3p	20440	St6gal1	0	0	1	1	0	2
mmu-miR-223-3p	20442	St3gal1	0	0	1	1	0	2
mmu-miR-223-3p	20447	St6galnac3	0	0	1	1	0	2
mmu-miR-223-3p	20473	Six3	0	1	0	1	0	2
mmu-miR-223-3p	20476	Six6	1	0	0	1	0	2
mmu-miR-223-3p	20482	Skil	1	0	0	1	0	2
mmu-miR-223-3p	20494	Slc10a2	1	0	0	1	0	2
mmu-miR-223-3p	20495	Slc12a1	0	1	0	1	0	2
mmu-miR-223-3p	20501	Slc16a1	0	0	1	1	0	2
mmu-miR-223-3p	20503	Slc16a7	0	1	1	0	0	2
mmu-miR-223-3p	20510	Slc1a1	0	0	1	1	0	2
mmu-miR-223-3p	20522	Slc23a1	0	1	0	1	0	2
mmu-miR-223-3p	20525	Slc2a1	1	0	0	1	0	2
mmu-miR-223-3p	20528	Slc2a4	1	0	1	0	0	2
mmu-miR-223-3p	20530	Slc31a2	0	1	1	0	0	2
mmu-miR-223-3p	20531	Slc34a2	1	0	0	1	0	2
mmu-miR-223-3p	20556	Slfn2	0	0	1	1	0	2
mmu-miR-223-3p	20587	Smarcb1	0	1	0	1	0	2
mmu-miR-223-3p	20588	Smarcc1	0	0	1	1	0	2
mmu-miR-223-3p	20619	Snap23	0	0	1	1	0	2
mmu-miR-223-3p	20624	Eftud2	0	1	0	1	0	2
mmu-miR-223-3p	20661	Sort1	0	0	1	1	0	2
mmu-miR-223-3p	20664	Sox1	0	1	0	1	0	2
mmu-miR-223-3p	20671	Sox17	0	0	1	1	0	2
mmu-miR-223-3p	20682	Sox9	1	0	0	1	0	2
mmu-miR-223-3p	20716	Serpina3n	0	0	1	1	0	2
mmu-miR-223-3p	20729	Spin1	1	0	0	1	0	2
mmu-miR-223-3p	20747	Spop	0	1	1	0	0	2
mmu-miR-223-3p	20755	Sprr2a1	1	0	0	1	0	2
mmu-miR-223-3p	20763	Sprr2i	0	0	1	1	0	2
mmu-miR-223-3p	20807	Srf	0	0	1	1	0	2
mmu-miR-223-3p	20850	Stat5a	1	0	0	1	0	2
mmu-miR-223-3p	20853	Stau1	0	1	0	1	0	2
mmu-miR-223-3p	20868	Stk10	0	0	1	1	0	2
mmu-miR-223-3p	20871	Aurkc	1	0	0	1	0	2
mmu-miR-223-3p	20887	Sult1a1	0	0	1	1	0	2
mmu-miR-223-3p	20918	Eif1	1	0	0	1	0	2
mmu-miR-223-3p	20932	Surf4	1	0	0	1	0	2
mmu-miR-223-3p	20947	Swap70	0	0	1	1	0	2
mmu-miR-223-3p	20965	Syn2	0	0	1	1	0	2
mmu-miR-223-3p	20977	Syp	0	0	1	1	0	2
mmu-miR-223-3p	20980	Syt2	1	0	1	0	0	2
mmu-miR-223-3p	21345	Tagln	0	1	0	1	0	2

mmu-miR-223-3p	21353	Tank	0	1	0	1	0	2
mmu-miR-223-3p	21354	Tap1	1	0	0	1	0	2
mmu-miR-223-3p	21356	Tapbp	1	0	0	1	0	2
mmu-miR-223-3p	21386	Tbx3	1	0	0	1	0	2
mmu-miR-223-3p	21387	Tbx4	0	1	0	1	0	2
mmu-miR-223-3p	21402	Skp1a	1	0	0	1	0	2
mmu-miR-223-3p	21405	Hnf1a	0	1	0	1	0	2
mmu-miR-223-3p	21411	Tcf20	1	0	0	1	0	2
mmu-miR-223-3p	21412	Tcf21	0	0	1	1	0	2
mmu-miR-223-3p	21416	Tcf7l2	0	0	1	1	0	2
mmu-miR-223-3p	21419	Tfap2b	1	0	0	1	0	2
mmu-miR-223-3p	21420	Tfap2c	1	0	0	1	0	2
mmu-miR-223-3p	21463	Tcp11	1	0	0	1	0	2
mmu-miR-223-3p	21645	Tcte1	0	1	0	1	0	2
mmu-miR-223-3p	21672	Prdx2	0	1	0	1	0	2
mmu-miR-223-3p	21679	Tead4	1	0	0	1	0	2
mmu-miR-223-3p	21684	Tectb	0	0	1	1	0	2
mmu-miR-223-3p	21754	Tesk1	0	1	0	1	0	2
mmu-miR-223-3p	21770	Ppp2r5d	1	0	0	1	0	2
mmu-miR-223-3p	21812	Tgfbr1	1	0	0	1	0	2
mmu-miR-223-3p	21813	Tgfbr2	0	0	1	1	0	2
mmu-miR-223-3p	21825	Thbs1	0	1	0	1	0	2
mmu-miR-223-3p	21832	Thpo	1	0	0	1	0	2
mmu-miR-223-3p	21838	Thy1	0	0	1	1	0	2
mmu-miR-223-3p	21847	Klf10	0	1	0	1	0	2
mmu-miR-223-3p	21853	Timeless	0	0	1	1	0	2
mmu-miR-223-3p	21855	Timm17b	1	0	0	1	0	2
mmu-miR-223-3p	21856	Timm44	0	1	0	1	0	2
mmu-miR-223-3p	21869	Nkx2-1	1	0	0	1	0	2
mmu-miR-223-3p	21871	Atp6v0a2	1	0	0	1	0	2
mmu-miR-223-3p	21872	Tjp1	0	0	1	1	0	2
mmu-miR-223-3p	21881	Tkt	1	0	0	1	0	2
mmu-miR-223-3p	21906	Otop1	0	1	0	1	0	2
mmu-miR-223-3p	21907	Nr2e1	0	0	1	1	0	2
mmu-miR-223-3p	21923	Tnc	1	0	0	1	0	2
mmu-miR-223-3p	21929	Tnfaip3	1	0	0	1	0	2
mmu-miR-223-3p	21930	Tnfaip6	0	0	1	1	0	2
mmu-miR-223-3p	21933	Tnfrsf10b	0	0	1	1	0	2
mmu-miR-223-3p	21940	Cd27	1	0	0	1	0	2
mmu-miR-223-3p	21943	Tnfsf11	0	0	1	1	0	2
mmu-miR-223-3p	21968	Tom1	0	1	0	1	0	2
mmu-miR-223-3p	21969	Top1	1	0	0	1	0	2
mmu-miR-223-3p	21973	Top2a	0	0	1	1	0	2
mmu-miR-223-3p	21981	Ppp1r13b	1	0	0	1	0	2
mmu-miR-223-3p	21984	Tpbpa	1	0	0	1	0	2
mmu-miR-223-3p	21985	Tpd52	1	0	0	1	0	2
mmu-miR-223-3p	21987	Tpd52l1	1	0	0	1	0	2
mmu-miR-223-3p	22003	Tpm1	0	0	1	1	0	2
mmu-miR-223-3p	22019	Tpp2	0	1	0	1	0	2
mmu-miR-223-3p	22026	Nr2c2	1	0	0	1	0	2
mmu-miR-223-3p	22036	Traip	1	0	1	0	0	2
mmu-miR-223-3p	22042	Tfrc	1	0	0	1	0	2
mmu-miR-223-3p	22059	Trp53	0	1	0	1	0	2
mmu-miR-223-3p	22061	Trp63	1	0	0	1	0	2
mmu-miR-223-3p	22067	Trpc5	0	0	1	1	0	2

mmu-miR-223-3p	22068	Trpc6	0	0	1	1	0	2
mmu-miR-223-3p	22121	Rpl13a	0	0	1	1	0	2
mmu-miR-223-3p	22123	Psmd3	0	1	0	1	0	2
mmu-miR-223-3p	22130	Ttf1	1	0	0	1	0	2
mmu-miR-223-3p	22137	Ttk	0	0	1	1	0	2
mmu-miR-223-3p	22142	Tuba1a	0	1	1	0	0	2
mmu-miR-223-3p	22143	Tuba1b	1	0	0	1	0	2
mmu-miR-223-3p	22145	Tuba4a	1	0	0	1	0	2
mmu-miR-223-3p	22174	Tyro3	0	0	1	1	0	2
mmu-miR-223-3p	22201	Uba1	0	0	1	1	0	2
mmu-miR-223-3p	22221	Ubp1	1	0	0	1	0	2
mmu-miR-223-3p	22247	Umps	1	0	0	1	0	2
mmu-miR-223-3p	22248	Unc119	1	0	0	1	0	2
mmu-miR-223-3p	22249	Unc13b	1	0	0	1	0	2
mmu-miR-223-3p	22259	Nr1h3	0	0	1	1	0	2
mmu-miR-223-3p	22276	Uros	1	0	0	1	0	2
mmu-miR-223-3p	22282	Usf2	0	0	1	1	0	2
mmu-miR-223-3p	22289	Kdm6a	0	1	1	0	0	2
mmu-miR-223-3p	22290	Uty	1	0	0	1	0	2
mmu-miR-223-3p	22293	Slc45a2	0	0	1	1	0	2
mmu-miR-223-3p	22301	Vmn2r89	1	0	0	1	0	2
mmu-miR-223-3p	22310	Vmn2r42	0	0	1	1	0	2
mmu-miR-223-3p	22317	Vamp1	1	0	0	1	0	2
mmu-miR-223-3p	22324	Vav1	0	0	1	1	0	2
mmu-miR-223-3p	22327	Vbp1	0	0	1	1	0	2
mmu-miR-223-3p	22339	Vegfa	1	0	0	1	0	2
mmu-miR-223-3p	22343	Lin7c	0	0	1	1	0	2
mmu-miR-223-3p	22346	Vhl	0	0	1	1	0	2
mmu-miR-223-3p	22359	Vldlr	1	0	0	1	0	2
mmu-miR-223-3p	22379	Fmnl3	0	0	1	1	0	2
mmu-miR-223-3p	22380	Wbp4	0	0	1	1	0	2
mmu-miR-223-3p	22384	Eif4h	0	0	1	1	0	2
mmu-miR-223-3p	22385	Baz1b	0	0	1	1	0	2
mmu-miR-223-3p	22393	Wfs1	1	0	0	1	0	2
mmu-miR-223-3p	22416	Wnt3a	0	0	1	1	0	2
mmu-miR-223-3p	22417	Wnt4	1	0	0	1	0	2
mmu-miR-223-3p	22421	Wnt7a	0	0	1	1	0	2
mmu-miR-223-3p	22427	Wrn	1	0	0	1	0	2
mmu-miR-223-3p	22431	Wt1	1	0	0	1	0	2
mmu-miR-223-3p	22439	Xk	0	1	0	1	0	2
mmu-miR-223-3p	22601	Yap1	0	1	0	1	0	2
mmu-miR-223-3p	22612	Yes1	0	0	1	1	0	2
mmu-miR-223-3p	22652	Mkrn3	0	0	1	1	0	2
mmu-miR-223-3p	22668	Sf1	0	1	0	1	0	2
mmu-miR-223-3p	22682	Zfand5	1	0	0	1	0	2
mmu-miR-223-3p	22690	Zfp28	1	0	0	1	0	2
mmu-miR-223-3p	22691	Zscan2	0	0	1	1	0	2
mmu-miR-223-3p	22717	Zfp59	0	0	1	1	0	2
mmu-miR-223-3p	22718	Zfp60	1	0	0	1	0	2
mmu-miR-223-3p	22746	Zfp85-rs1	1	0	0	1	0	2
mmu-miR-223-3p	22750	Zfp9	0	0	1	1	0	2
mmu-miR-223-3p	22763	Zfr	0	1	0	1	0	2
mmu-miR-223-3p	22770	Zhx1	1	0	0	1	0	2
mmu-miR-223-3p	22772	Zic2	1	0	0	1	0	2
mmu-miR-223-3p	22776	Zim1	1	0	1	0	0	2

mmu-miR-223-3p	22781	Ikzf4	0	1	1	0	0	2
mmu-miR-223-3p	22782	Slc30a1	0	0	1	1	0	2
mmu-miR-223-3p	22785	Slc30a4	0	1	0	1	0	2
mmu-miR-223-3p	22791	Dnajc2	1	1	0	0	0	2
mmu-miR-223-3p	23794	Adamts5	1	0	0	1	0	2
mmu-miR-223-3p	23796	Aplnr	1	0	0	1	0	2
mmu-miR-223-3p	23802	Amfr	0	0	1	1	0	2
mmu-miR-223-3p	23807	Arih2	1	0	0	1	0	2
mmu-miR-223-3p	23854	Def8	0	0	1	1	0	2
mmu-miR-223-3p	23876	Fbln5	0	0	1	1	0	2
mmu-miR-223-3p	23881	G3bp2	1	0	0	1	0	2
mmu-miR-223-3p	23885	Gmcl1	1	0	0	1	0	2
mmu-miR-223-3p	23892	Grem1	0	0	1	1	0	2
mmu-miR-223-3p	23897	Hax1	0	0	1	1	0	2
mmu-miR-223-3p	23928	Lamc3	0	0	1	1	0	2
mmu-miR-223-3p	23938	Map2k5	0	0	1	1	0	2
mmu-miR-223-3p	23947	Mid2	1	0	0	1	0	2
mmu-miR-223-3p	23948	Mmp17	1	0	1	0	0	2
mmu-miR-223-3p	23950	Dnajb6	0	0	1	1	0	2
mmu-miR-223-3p	23959	Nt5e	1	0	0	1	0	2
mmu-miR-223-3p	23985	Slc26a4	0	1	1	0	0	2
mmu-miR-223-3p	23988	Pin1	0	0	1	1	0	2
mmu-miR-223-3p	23994	Dazap2	1	0	0	1	0	2
mmu-miR-223-3p	23997	Psmd13	0	1	0	1	0	2
mmu-miR-223-3p	24012	Rgs7	1	0	0	1	0	2
mmu-miR-223-3p	24015	Abce1	1	0	0	1	0	2
mmu-miR-223-3p	24055	Sh3bp2	0	0	1	1	0	2
mmu-miR-223-3p	24056	Sh3bp5	1	0	0	1	0	2
mmu-miR-223-3p	24059	Slco2a1	0	0	1	1	0	2
mmu-miR-223-3p	24060	Slc35a1	0	0	1	1	0	2
mmu-miR-223-3p	24064	Spry2	0	0	1	1	0	2
mmu-miR-223-3p	24099	Tnfsf13b	0	0	1	1	0	2
mmu-miR-223-3p	24109	Ubl3	1	0	0	1	0	2
mmu-miR-223-3p	24115	Best1	0	0	1	1	0	2
mmu-miR-223-3p	24116	Nelfa	0	1	0	1	0	2
mmu-miR-223-3p	24131	Ldb3	1	0	0	1	0	2
mmu-miR-223-3p	26369	Cetn1	1	0	0	1	0	2
mmu-miR-223-3p	26380	Esrrb	1	0	0	1	0	2
mmu-miR-223-3p	26390	Mapkbp1	1	0	0	1	0	2
mmu-miR-223-3p	26397	Map2k3	0	0	1	1	0	2
mmu-miR-223-3p	26398	Map2k4	1	0	0	1	0	2
mmu-miR-223-3p	26416	Mapk14	0	0	1	1	0	2
mmu-miR-223-3p	26419	Mapk8	0	1	0	1	0	2
mmu-miR-223-3p	26423	Nr5a1	1	0	0	1	0	2
mmu-miR-223-3p	26424	Nr5a2	1	0	0	1	0	2
mmu-miR-223-3p	26425	Nubp1	0	1	0	1	0	2
mmu-miR-223-3p	26426	Nubp2	0	0	1	1	0	2
mmu-miR-223-3p	26430	Parg	0	0	1	1	0	2
mmu-miR-223-3p	26432	Plod2	1	0	0	1	0	2
mmu-miR-223-3p	26433	Plod3	0	0	1	1	0	2
mmu-miR-223-3p	26436	Psg16	0	1	1	0	0	2
mmu-miR-223-3p	26456	Sema4g	0	1	0	1	0	2
mmu-miR-223-3p	26457	Slc27a1	0	0	1	1	0	2
mmu-miR-223-3p	26562	Ncdn	1	0	0	1	0	2
mmu-miR-223-3p	26570	Slc7a11	1	0	0	1	0	2

mmu-miR-223-3p	26878	B3galt2	0	0	1	1	0	2
mmu-miR-223-3p	26887	Chst4	1	0	0	1	0	2
mmu-miR-223-3p	26895	Cops7b	0	1	0	1	0	2
mmu-miR-223-3p	26905	Eif2s3x	0	0	1	1	0	2
mmu-miR-223-3p	26913	Gprin1	0	0	1	1	0	2
mmu-miR-223-3p	26919	Zfp346	0	0	1	1	0	2
mmu-miR-223-3p	26920	Cep110	0	0	0	1	1	2
mmu-miR-223-3p	26921	Map4k4	0	0	1	1	0	2
mmu-miR-223-3p	26931	Ppp2r5c	1	0	0	1	0	2
mmu-miR-223-3p	26946	Trpc7	0	0	1	1	0	2
mmu-miR-223-3p	26971	Pla2g2f	0	0	1	1	0	2
mmu-miR-223-3p	26987	Eif4e2	1	0	0	1	0	2
mmu-miR-223-3p	27007	Klrk1	1	0	0	1	0	2
mmu-miR-223-3p	27008	Micall1	0	0	1	1	0	2
mmu-miR-223-3p	27028	Ermap	0	1	0	1	0	2
mmu-miR-223-3p	27029	Sgsh	0	0	1	1	0	2
mmu-miR-223-3p	27045	Nit1	1	0	0	1	0	2
mmu-miR-223-3p	27052	Aoah	0	0	1	1	0	2
mmu-miR-223-3p	27205	Podxl	1	0	0	1	0	2
mmu-miR-223-3p	27220	Cartpt	0	1	0	1	0	2
mmu-miR-223-3p	27221	Chaf1a	0	0	1	1	0	2
mmu-miR-223-3p	27224	Tceb3	0	0	1	1	0	2
mmu-miR-223-3p	27260	Plek2	0	0	1	1	0	2
mmu-miR-223-3p	27273	Pdk4	0	0	1	1	0	2
mmu-miR-223-3p	27275	Nufip1	0	0	1	1	0	2
mmu-miR-223-3p	27276	Plekhb1	0	1	0	1	0	2
mmu-miR-223-3p	27281	Hrasls	0	0	1	1	0	2
mmu-miR-223-3p	27355	Pald1	0	0	1	1	0	2
mmu-miR-223-3p	27356	Insl6	1	0	0	1	0	2
mmu-miR-223-3p	27360	Add3	1	0	0	1	0	2
mmu-miR-223-3p	27364	Srr	0	0	1	1	0	2
mmu-miR-223-3p	27369	Dguok	0	0	1	1	0	2
mmu-miR-223-3p	27379	Tcl1b1	1	0	0	1	0	2
mmu-miR-223-3p	27381	Tcl1b2	1	0	0	1	0	2
mmu-miR-223-3p	27386	Npas3	0	0	1	1	0	2
mmu-miR-223-3p	27389	Dusp13	0	0	1	1	0	2
mmu-miR-223-3p	27399	Ip6k1	1	0	0	1	0	2
mmu-miR-223-3p	27407	Abcf2	0	0	1	1	0	2
mmu-miR-223-3p	27412	Peg12	1	0	0	1	0	2
mmu-miR-223-3p	27416	Abcc5	0	0	1	1	0	2
mmu-miR-223-3p	27428	Shroom3	1	0	0	1	0	2
mmu-miR-223-3p	27528	Nrep	0	1	0	1	0	2
mmu-miR-223-3p	27660	1700088E04Rik	0	0	1	1	0	2
mmu-miR-223-3p	27784	Commd8	0	0	1	1	0	2
mmu-miR-223-3p	27878	Tada1	1	0	1	0	0	2
mmu-miR-223-3p	27973	Vkorc1	0	1	0	1	0	2
mmu-miR-223-3p	27993	Imp4	1	0	0	1	0	2
mmu-miR-223-3p	27999	Fam3c	0	0	1	1	0	2
mmu-miR-223-3p	28028	Mrpl50	0	0	1	1	0	2
mmu-miR-223-3p	28035	Usp39	0	0	1	1	0	2
mmu-miR-223-3p	28071	Twistnb	0	0	1	1	0	2
mmu-miR-223-3p	28075	Desi1	0	0	1	1	0	2
mmu-miR-223-3p	28081	Fam104a	0	0	1	1	0	2
mmu-miR-223-3p	28088	D10Wsu52e	0	1	0	1	0	2
mmu-miR-223-3p	28169	Agpat3	0	0	1	1	0	2

mmu-miR-223-3p	28193	Reep3	1	0	0	1	0	2
mmu-miR-223-3p	28194	Apon	1	0	1	0	0	2
mmu-miR-223-3p	28240	Trpm2	0	0	1	1	0	2
mmu-miR-223-3p	28253	Slco1b2	1	0	0	1	0	2
mmu-miR-223-3p	28254	Slco1a6	0	1	0	1	0	2
mmu-miR-223-3p	29857	Mapk12	0	0	1	1	0	2
mmu-miR-223-3p	29873	Cspg5	0	0	1	1	0	2
mmu-miR-223-3p	29876	Clic4	1	0	0	1	0	2
mmu-miR-223-3p	30049	Scd3	0	0	1	1	0	2
mmu-miR-223-3p	30055	Timm13	0	0	1	1	0	2
mmu-miR-223-3p	30058	Timm8a1	0	0	1	1	0	2
mmu-miR-223-3p	30785	Cttnbp2	0	1	0	1	0	2
mmu-miR-223-3p	30805	Slc22a4	1	0	0	1	0	2
mmu-miR-223-3p	30806	Adamts8	0	1	0	1	0	2
mmu-miR-223-3p	30841	Kdm2b	0	1	0	1	0	2
mmu-miR-223-3p	30843	Fbxl12	1	0	0	1	0	2
mmu-miR-223-3p	30935	Tor3a	1	0	0	1	0	2
mmu-miR-223-3p	30946	Abt1	1	0	0	1	0	2
mmu-miR-223-3p	30952	Cngb3	1	0	0	1	0	2
mmu-miR-223-3p	30955	Pik3cg	0	0	1	1	0	2
mmu-miR-223-3p	30957	Mapk8ip3	0	0	1	1	0	2
mmu-miR-223-3p	30960	Vapa	1	0	0	1	0	2
mmu-miR-223-3p	50490	Nox4	0	0	1	1	0	2
mmu-miR-223-3p	50496	E2f6	1	0	0	1	0	2
mmu-miR-223-3p	50498	Ebi3	0	0	1	1	0	2
mmu-miR-223-3p	50500	Ttpa	0	0	1	1	0	2
mmu-miR-223-3p	50505	Ercc4	1	0	0	1	0	2
mmu-miR-223-3p	50527	Ero1l	1	0	0	1	0	2
mmu-miR-223-3p	50529	Mrps7	0	0	1	1	0	2
mmu-miR-223-3p	50702	Cfhr1	1	0	0	1	0	2
mmu-miR-223-3p	50706	Postn	0	0	1	1	0	2
mmu-miR-223-3p	50758	Fbxl17	1	0	0	1	0	2
mmu-miR-223-3p	50767	Pnpla6	0	1	0	1	0	2
mmu-miR-223-3p	50768	Dlc1	0	0	1	1	0	2
mmu-miR-223-3p	50778	Rgs1	0	0	1	1	0	2
mmu-miR-223-3p	50781	Dkk3	0	0	1	1	0	2
mmu-miR-223-3p	50783	Lsm4	0	0	1	1	0	2
mmu-miR-223-3p	50784	Ppap2c	0	0	1	1	0	2
mmu-miR-223-3p	50794	Klf13	0	1	0	1	0	2
mmu-miR-223-3p	50796	Dmrt1	0	0	1	1	0	2
mmu-miR-223-3p	50850	Spast	1	0	0	1	0	2
mmu-miR-223-3p	50887	Hmgn5	0	0	1	1	0	2
mmu-miR-223-3p	50905	Il17rb	1	0	0	1	0	2
mmu-miR-223-3p	50914	Olig1	0	0	1	1	0	2
mmu-miR-223-3p	50916	Irx4	0	0	1	1	0	2
mmu-miR-223-3p	50917	Galns	0	0	1	1	0	2
mmu-miR-223-3p	50928	Klrg1	0	0	1	1	0	2
mmu-miR-223-3p	50930	Tnfsf14	0	0	1	1	0	2
mmu-miR-223-3p	50997	Mpp2	0	1	0	1	0	2
mmu-miR-223-3p	51786	Cpsf2	1	0	0	1	0	2
mmu-miR-223-3p	51793	Ddah2	0	0	1	1	0	2
mmu-miR-223-3p	51795	Srpx	1	0	0	1	0	2
mmu-miR-223-3p	51799	Rundc3a	1	0	0	1	0	2
mmu-miR-223-3p	51810	Hnrnpu	0	0	1	1	0	2
mmu-miR-223-3p	51811	Clec4f	1	0	0	1	0	2

mmu-miR-223-3p	51813	Ccnc	0	0	1	1	0	2
mmu-miR-223-3p	51885	Tubgcp4	0	1	0	1	0	2
mmu-miR-223-3p	51960	Kctd18	0	1	0	1	0	2
mmu-miR-223-3p	52004	Cdk2ap2	1	0	0	1	0	2
mmu-miR-223-3p	52009	Hn1l	0	0	1	1	0	2
mmu-miR-223-3p	52014	Nus1	1	0	0	1	0	2
mmu-miR-223-3p	52020	Umodl1	1	0	0	0	1	2
mmu-miR-223-3p	52023	Pibf1	1	0	0	1	0	2
mmu-miR-223-3p	52118	Pvr	1	0	0	1	0	2
mmu-miR-223-3p	52123	Agpat5	0	1	0	1	0	2
mmu-miR-223-3p	52202	Rbm34	0	0	1	1	0	2
mmu-miR-223-3p	52323	Klhl7	0	0	1	1	0	2
mmu-miR-223-3p	52377	Rcn3	0	0	1	1	0	2
mmu-miR-223-3p	52398	Sept11	0	0	1	1	0	2
mmu-miR-223-3p	52443	Mrpl48	0	1	0	1	0	2
mmu-miR-223-3p	52463	Tet1	1	0	0	1	0	2
mmu-miR-223-3p	52468	Ctdsp2	1	0	0	1	0	2
mmu-miR-223-3p	52502	Carhsp1	0	0	1	1	0	2
mmu-miR-223-3p	52551	Sgta	0	0	1	1	0	2
mmu-miR-223-3p	52563	Cdc23	0	0	1	1	0	2
mmu-miR-223-3p	52588	Tspan14	0	0	1	1	0	2
mmu-miR-223-3p	52589	Ncald	0	0	1	1	0	2
mmu-miR-223-3p	52615	Suz12	0	0	1	1	0	2
mmu-miR-223-3p	52633	Nit2	1	0	0	1	0	2
mmu-miR-223-3p	52653	Nudcd2	0	0	1	1	0	2
mmu-miR-223-3p	52679	E2f7	0	0	1	1	0	2
mmu-miR-223-3p	52690	Setd3	0	0	1	1	0	2
mmu-miR-223-3p	52712	Zkscan6	1	0	0	1	0	2
mmu-miR-223-3p	52713	Ccdc59	0	0	1	1	0	2
mmu-miR-223-3p	52715	Ccdc43	1	0	0	1	0	2
mmu-miR-223-3p	52815	Ldhd	1	0	0	1	0	2
mmu-miR-223-3p	52830	Pnrc2	0	0	1	1	0	2
mmu-miR-223-3p	52837	Tmx4	0	0	1	1	0	2
mmu-miR-223-3p	53313	Atp2a3	0	0	1	1	0	2
mmu-miR-223-3p	53318	Pdlim3	1	0	0	1	0	2
mmu-miR-223-3p	53319	Nxf1	0	0	1	1	0	2
mmu-miR-223-3p	53320	Folh1	0	0	1	1	0	2
mmu-miR-223-3p	53332	Mtmr1	1	0	0	1	0	2
mmu-miR-223-3p	53374	Chst3	0	0	1	1	0	2
mmu-miR-223-3p	53379	Hnrnpa2b1	1	0	0	1	0	2
mmu-miR-223-3p	53382	Txnl1	1	0	0	1	0	2
mmu-miR-223-3p	53417	Hif3a	0	0	1	1	0	2
mmu-miR-223-3p	53418	B4galt2	0	1	0	1	0	2
mmu-miR-223-3p	53419	Corin	1	0	0	1	0	2
mmu-miR-223-3p	53421	Sec61a1	1	0	0	1	0	2
mmu-miR-223-3p	53605	Nap1l1	1	0	0	1	0	2
mmu-miR-223-3p	53610	Nono	0	1	0	1	0	2
mmu-miR-223-3p	53619	Blcap	0	0	1	1	0	2
mmu-miR-223-3p	53860	Sept9	1	0	0	1	0	2
mmu-miR-223-3p	53869	Rab11a	1	0	0	1	0	2
mmu-miR-223-3p	53871	Pkd2l2	0	1	0	1	0	2
mmu-miR-223-3p	53872	Caprin1	1	0	0	1	0	2
mmu-miR-223-3p	53886	Cdkl2	1	0	0	1	0	2
mmu-miR-223-3p	53890	Sart3	1	0	1	0	0	2
mmu-miR-223-3p	53902	Rcan3	1	0	0	1	0	2

mmu-miR-223-3p	53970	Rfx5	0	1	0	1	0	2
mmu-miR-223-3p	54128	Pmm2	0	0	1	1	0	2
mmu-miR-223-3p	54132	Pdlim1	0	0	1	1	0	2
mmu-miR-223-3p	54139	Irf6	0	0	1	1	0	2
mmu-miR-223-3p	54141	Spag5	1	0	0	1	0	2
mmu-miR-223-3p	54156	Egfl6	0	0	1	1	0	2
mmu-miR-223-3p	54167	Icos	1	0	0	1	0	2
mmu-miR-223-3p	54188	Cpsf4	0	0	1	1	0	2
mmu-miR-223-3p	54201	Zfp316	1	0	0	1	0	2
mmu-miR-223-3p	54214	Golga4	0	0	1	1	0	2
mmu-miR-223-3p	54354	Rassf5	1	0	0	1	0	2
mmu-miR-223-3p	54357	Epb4.1l4b	1	1	0	0	0	2
mmu-miR-223-3p	54367	Zfp326	0	0	1	1	0	2
mmu-miR-223-3p	54383	Phc2	0	1	0	1	0	2
mmu-miR-223-3p	54384	Mtmr7	0	0	1	1	0	2
mmu-miR-223-3p	54391	Rfk	0	0	1	1	0	2
mmu-miR-223-3p	54401	Ywhab	1	0	0	1	0	2
mmu-miR-223-3p	54409	Ramp2	0	1	0	1	0	2
mmu-miR-223-3p	54422	Barhl1	0	0	1	1	0	2
mmu-miR-223-3p	54451	Cpsf3	1	0	0	1	0	2
mmu-miR-223-3p	54473	Tollip	0	0	1	1	0	2
mmu-miR-223-3p	54484	Mkrn1	1	0	0	1	0	2
mmu-miR-223-3p	54607	Socs6	1	0	1	0	0	2
mmu-miR-223-3p	54610	Tbc1d8	0	0	1	1	0	2
mmu-miR-223-3p	54613	St3gal6	0	1	0	1	0	2
mmu-miR-223-3p	54630	Prickle3	0	0	1	1	0	2
mmu-miR-223-3p	54631	Nphs1	0	0	1	1	0	2
mmu-miR-223-3p	54632	Ftsj1	0	0	1	1	0	2
mmu-miR-223-3p	54638	Ccdc22	0	0	1	1	0	2
mmu-miR-223-3p	54667	Atp8b2	0	0	1	1	0	2
mmu-miR-223-3p	54720	Rcan1	1	0	0	1	0	2
mmu-miR-223-3p	54725	Cadm1	1	0	0	1	0	2
mmu-miR-223-3p	55942	Sertad1	0	0	1	1	0	2
mmu-miR-223-3p	55949	Eef1b2	1	0	0	1	0	2
mmu-miR-223-3p	55960	Ebag9	0	0	1	1	0	2
mmu-miR-223-3p	55979	Agpat1	0	0	1	1	0	2
mmu-miR-223-3p	55984	Camkk1	1	0	0	1	0	2
mmu-miR-223-3p	55985	Cxcl13	1	0	0	1	0	2
mmu-miR-223-3p	55991	Panx1	0	1	0	1	0	2
mmu-miR-223-3p	55994	Smad9	0	0	1	1	0	2
mmu-miR-223-3p	56014	Olfr70	1	0	1	0	0	2
mmu-miR-223-3p	56030	Tmem131	1	0	0	1	0	2
mmu-miR-223-3p	56048	Lgals8	1	0	0	1	0	2
mmu-miR-223-3p	56055	Gtpbp2	0	1	0	1	0	2
mmu-miR-223-3p	56057	Btg4	1	0	0	1	0	2
mmu-miR-223-3p	56078	Car5b	1	0	0	1	0	2
mmu-miR-223-3p	56089	Ramp3	0	0	1	1	0	2
mmu-miR-223-3p	56149	Grasp	1	0	0	1	0	2
mmu-miR-223-3p	56185	Hao2	0	0	1	1	0	2
mmu-miR-223-3p	56187	Rabggta	0	1	0	1	0	2
mmu-miR-223-3p	56198	Heyl	1	0	0	1	0	2
mmu-miR-223-3p	56200	Ddx21	0	0	1	1	0	2
mmu-miR-223-3p	56207	Uchl5	1	0	0	1	0	2
mmu-miR-223-3p	56208	Becn1	0	0	1	1	0	2
mmu-miR-223-3p	56214	Scamp4	1	0	0	1	0	2

mmu-miR-223-3p	56216	Stx1b	0	1	0	1	0	2
mmu-miR-223-3p	56224	Tspan5	0	0	1	1	0	2
mmu-miR-223-3p	56228	Ube2j1	0	0	1	1	0	2
mmu-miR-223-3p	56229	Thsd1	0	1	0	1	0	2
mmu-miR-223-3p	56233	Hdac7	1	0	0	1	0	2
mmu-miR-223-3p	56273	Pex14	0	1	0	1	0	2
mmu-miR-223-3p	56295	Higd1a	0	0	1	1	0	2
mmu-miR-223-3p	56298	Atl2	1	0	0	1	0	2
mmu-miR-223-3p	56305	Pitpnb	1	0	0	1	0	2
mmu-miR-223-3p	56320	Dbn1	1	0	1	0	0	2
mmu-miR-223-3p	56322	Timm22	0	0	1	1	0	2
mmu-miR-223-3p	56332	Amotl2	0	0	1	1	0	2
mmu-miR-223-3p	56334	Tmed2	0	1	0	1	0	2
mmu-miR-223-3p	56336	B4galt5	0	0	1	1	0	2
mmu-miR-223-3p	56353	Rybp	0	0	1	1	0	2
mmu-miR-223-3p	56357	Ivd	0	0	1	1	0	2
mmu-miR-223-3p	56358	Copz2	0	0	1	1	0	2
mmu-miR-223-3p	56362	Sult1b1	1	0	0	1	0	2
mmu-miR-223-3p	56364	Zmym3	1	0	0	1	0	2
mmu-miR-223-3p	56374	Tmem59	1	0	0	1	0	2
mmu-miR-223-3p	56375	B4galt4	0	0	1	1	0	2
mmu-miR-223-3p	56389	Stx5a	0	0	1	1	0	2
mmu-miR-223-3p	56395	Tmem115	0	1	1	0	0	2
mmu-miR-223-3p	56398	Chp1	1	0	0	1	0	2
mmu-miR-223-3p	56404	Trip4	1	0	0	1	0	2
mmu-miR-223-3p	56409	Nudt3	1	0	0	1	0	2
mmu-miR-223-3p	56410	Cbln3	1	0	0	1	0	2
mmu-miR-223-3p	56418	Ykt6	1	0	1	0	0	2
mmu-miR-223-3p	56426	Pdcd10	0	1	0	1	0	2
mmu-miR-223-3p	56428	Mtch2	0	0	1	1	0	2
mmu-miR-223-3p	56440	Snx1	0	0	1	1	0	2
mmu-miR-223-3p	56442	Serinc1	1	0	0	1	0	2
mmu-miR-223-3p	56445	Dnaja2	1	0	1	0	0	2
mmu-miR-223-3p	56451	Suclg1	0	0	1	1	0	2
mmu-miR-223-3p	56456	Actl6a	0	0	1	1	0	2
mmu-miR-223-3p	56457	Clptm1	0	0	1	1	0	2
mmu-miR-223-3p	56471	Stmn4	0	0	1	1	0	2
mmu-miR-223-3p	56489	Ikbke	1	0	0	1	0	2
mmu-miR-223-3p	56490	Zbtb20	0	1	0	1	0	2
mmu-miR-223-3p	56491	Vapb	0	0	1	1	0	2
mmu-miR-223-3p	56494	Gosr2	1	0	0	1	0	2
mmu-miR-223-3p	56508	Rapgef4	1	0	0	1	0	2
mmu-miR-223-3p	56522	Papolb	0	1	0	1	0	2
mmu-miR-223-3p	56533	Rgs17	1	0	0	1	0	2
mmu-miR-223-3p	56541	Habp4	0	0	1	1	0	2
mmu-miR-223-3p	56550	Ube2d2a	1	0	1	0	0	2
mmu-miR-223-3p	56629	Dnase2b	0	0	1	1	0	2
mmu-miR-223-3p	56631	Trim17	0	1	0	1	0	2
mmu-miR-223-3p	56635	Prl2a1	0	0	1	1	0	2
mmu-miR-223-3p	56643	Slc15a1	0	0	1	1	0	2
mmu-miR-223-3p	56690	Mlycd	0	0	1	1	0	2
mmu-miR-223-3p	56696	Gpr132	0	0	1	1	0	2
mmu-miR-223-3p	56700	0610031J06Rik	0	1	0	1	0	2
mmu-miR-223-3p	56709	Dnajb12	0	1	0	1	0	2
mmu-miR-223-3p	56711	Plag1	1	0	0	1	0	2

mmu-miR-223-3p	56720	Tdo2	0	0	1	1	0	2
mmu-miR-223-3p	56749	Dhodh	0	0	1	1	0	2
mmu-miR-223-3p	56805	Zbtb33	1	0	0	1	0	2
mmu-miR-223-3p	56808	Cacna2d2	1	0	0	1	0	2
mmu-miR-223-3p	56857	Slc37a2	0	0	1	1	0	2
mmu-miR-223-3p	57246	Tbx20	0	0	1	1	0	2
mmu-miR-223-3p	57259	Tob2	1	0	0	1	0	2
mmu-miR-223-3p	57266	Cxcl14	1	0	0	1	0	2
mmu-miR-223-3p	57275	Lenep	0	0	1	1	0	2
mmu-miR-223-3p	57278	Bcam	0	1	0	1	0	2
mmu-miR-223-3p	57295	Icmt	1	0	0	1	0	2
mmu-miR-223-3p	57316	C1d	1	0	0	1	0	2
mmu-miR-223-3p	57340	Jph3	0	1	1	0	0	2
mmu-miR-223-3p	57342	Parva	1	0	0	1	0	2
mmu-miR-223-3p	57354	Cramp1l	0	0	1	1	0	2
mmu-miR-223-3p	57385	P2ry4	0	0	1	1	0	2
mmu-miR-223-3p	57394	Tmem27	0	0	1	1	0	2
mmu-miR-223-3p	57423	Atp5j2	0	0	1	1	0	2
mmu-miR-223-3p	57434	Xrcc2	0	0	1	1	0	2
mmu-miR-223-3p	57438	March7	0	0	1	1	0	2
mmu-miR-223-3p	57740	Stk32c	0	0	1	1	0	2
mmu-miR-223-3p	57743	Sec61a2	1	0	0	1	0	2
mmu-miR-223-3p	57750	Wdr12	1	0	0	1	0	2
mmu-miR-223-3p	57751	Rnf25	0	0	1	1	0	2
mmu-miR-223-3p	57773	Wdr4	1	0	0	1	0	2
mmu-miR-223-3p	57775	Usp29	0	0	1	1	0	2
mmu-miR-223-3p	57778	Fmnl1	0	0	1	1	0	2
mmu-miR-223-3p	57783	Tnip1	0	0	1	1	0	2
mmu-miR-223-3p	57813	Tk2	0	0	1	1	0	2
mmu-miR-223-3p	57814	Kcne4	0	1	0	1	0	2
mmu-miR-223-3p	57890	Il17re	1	0	0	1	0	2
mmu-miR-223-3p	57896	Krcc1	0	0	1	1	0	2
mmu-miR-223-3p	58176	Rhbg	1	0	0	1	0	2
mmu-miR-223-3p	58182	Prokr1	1	0	0	1	0	2
mmu-miR-223-3p	58185	Rsad2	1	0	0	1	0	2
mmu-miR-223-3p	58188	Vstm2b	1	1	0	0	0	2
mmu-miR-223-3p	58194	Sh3kbp1	1	0	0	1	0	2
mmu-miR-223-3p	58202	Nelfb	0	0	1	1	0	2
mmu-miR-223-3p	58223	Mmp19	1	0	0	1	0	2
mmu-miR-223-3p	58226	Cacna1h	1	0	0	1	0	2
mmu-miR-223-3p	58240	Hs1bp3	0	0	1	1	0	2
mmu-miR-223-3p	58242	Nudt11	0	0	1	1	0	2
mmu-miR-223-3p	58521	Eid1	1	0	0	1	0	2
mmu-miR-223-3p	58799	Crbn	0	1	0	1	0	2
mmu-miR-223-3p	58807	Slco1c1	1	0	0	1	0	2
mmu-miR-223-3p	58809	Rnase4	0	1	0	1	0	2
mmu-miR-223-3p	58860	Adamdec1	1	0	0	1	0	2
mmu-miR-223-3p	58869	Pex5l	1	0	0	1	0	2
mmu-miR-223-3p	58875	Hibadh	0	0	1	1	0	2
mmu-miR-223-3p	58887	Repin1	1	0	0	1	0	2
mmu-miR-223-3p	58911	Sumf1	1	0	1	0	0	2
mmu-miR-223-3p	58998	Pvrl3	0	1	0	1	0	2
mmu-miR-223-3p	59006	Myoz2	0	1	0	1	0	2
mmu-miR-223-3p	59010	Sqrdl	0	1	0	1	0	2
mmu-miR-223-3p	59014	Rrs1	0	0	1	1	0	2

mmu-miR-223-3p	59015	Nup160	0	0	1	1	0	2
mmu-miR-223-3p	59021	Rab2a	0	0	1	1	0	2
mmu-miR-223-3p	59024	Med12	0	0	1	1	0	2
mmu-miR-223-3p	59032	Ppp2r3c	1	0	0	1	0	2
mmu-miR-223-3p	59041	Stk25	0	1	1	0	0	2
mmu-miR-223-3p	59048	C1galt1c1	1	0	0	1	0	2
mmu-miR-223-3p	59057	Zfp191	1	0	0	1	0	2
mmu-miR-223-3p	59058	Bhlhe22	1	0	0	1	0	2
mmu-miR-223-3p	59093	Pcbp3	0	0	1	1	0	2
mmu-miR-223-3p	59126	Nek6	0	0	1	1	0	2
mmu-miR-223-3p	59290	Gpa33	0	0	1	1	0	2
mmu-miR-223-3p	59308	Emcn	1	1	0	0	0	2
mmu-miR-223-3p	60361	Ms4a4b	1	0	0	1	0	2
mmu-miR-223-3p	60363	Cldn15	0	0	1	1	0	2
mmu-miR-223-3p	60365	Rbm8a	1	0	0	1	0	2
mmu-miR-223-3p	60406	Sap30	1	0	0	1	0	2
mmu-miR-223-3p	60409	Trappc4	0	0	1	1	0	2
mmu-miR-223-3p	60411	Cenpk	0	0	1	1	0	2
mmu-miR-223-3p	60504	Il21r	1	0	0	1	0	2
mmu-miR-223-3p	60510	Syt9	0	0	1	1	0	2
mmu-miR-223-3p	60530	Fignl1	0	0	1	1	0	2
mmu-miR-223-3p	60532	Wtap	0	0	1	1	0	2
mmu-miR-223-3p	60596	Gucy1a3	0	0	1	1	0	2
mmu-miR-223-3p	60611	Foxj2	0	0	1	1	0	2
mmu-miR-223-3p	63857	Bcmo1	1	0	0	1	0	2
mmu-miR-223-3p	63859	Impg1	0	0	1	1	0	2
mmu-miR-223-3p	63913	Fam129a	0	1	0	1	0	2
mmu-miR-223-3p	63958	Ube4b	0	1	0	1	0	2
mmu-miR-223-3p	64008	Aqp9	1	0	1	0	0	2
mmu-miR-223-3p	64099	Parvg	1	0	1	0	0	2
mmu-miR-223-3p	64144	Mllt1	1	0	0	1	0	2
mmu-miR-223-3p	64209	Herpud1	1	0	0	1	0	2
mmu-miR-223-3p	64214	Rgs18	1	0	0	1	0	2
mmu-miR-223-3p	64242	Ngb	0	0	1	1	0	2
mmu-miR-223-3p	64292	Ptges	0	0	1	1	0	2
mmu-miR-223-3p	64296	Abhd8	1	0	0	1	0	2
mmu-miR-223-3p	64406	Sp5	0	0	1	1	0	2
mmu-miR-223-3p	64450	Gpr85	0	1	0	1	0	2
mmu-miR-223-3p	64451	Dip2a	0	0	1	1	0	2
mmu-miR-223-3p	64654	Fgf23	1	0	0	1	0	2
mmu-miR-223-3p	64656	Mrps23	0	0	1	1	0	2
mmu-miR-223-3p	64706	Scube1	0	0	1	1	0	2
mmu-miR-223-3p	64929	Scel	0	1	0	1	0	2
mmu-miR-223-3p	64945	Cldn12	0	0	1	1	0	2
mmu-miR-223-3p	65100	Zic5	1	0	0	1	0	2
mmu-miR-223-3p	65107	Lrp10	0	1	0	1	0	2
mmu-miR-223-3p	65112	Pmepa1	0	1	0	1	0	2
mmu-miR-223-3p	65257	Asb3	0	0	1	1	0	2
mmu-miR-223-3p	65964	Zak	0	0	1	1	0	2
mmu-miR-223-3p	65970	Lima1	1	0	0	1	0	2
mmu-miR-223-3p	66082	Abhd6	0	0	1	1	0	2
mmu-miR-223-3p	66086	Fopnl	1	0	0	1	0	2
mmu-miR-223-3p	66101	Ppih	0	0	1	1	0	2
mmu-miR-223-3p	66105	Ube2d3	0	0	1	1	0	2
mmu-miR-223-3p	66126	Elof1	0	1	0	1	0	2

mmu-miR-223-3p	66147	Necap2	0	1	0	1	0	2
mmu-miR-223-3p	66148	Dnajc15	0	0	1	1	0	2
mmu-miR-223-3p	66164	Nip7	1	0	0	1	0	2
mmu-miR-223-3p	66169	Tomm7	1	0	0	1	0	2
mmu-miR-223-3p	66176	Nat9	0	0	1	1	0	2
mmu-miR-223-3p	66183	Sptssb	0	0	1	1	0	2
mmu-miR-223-3p	66185	1110037F02Rik	1	0	0	1	0	2
mmu-miR-223-3p	66193	Pithd1	1	0	0	1	0	2
mmu-miR-223-3p	66200	Commd6	1	0	0	1	0	2
mmu-miR-223-3p	66201	Vta1	1	0	0	1	0	2
mmu-miR-223-3p	66205	Cd302	1	0	0	1	0	2
mmu-miR-223-3p	66212	Sec61b	0	0	1	1	0	2
mmu-miR-223-3p	66226	Trappc2	0	1	0	1	0	2
mmu-miR-223-3p	66237	Atp6v1g2	1	0	0	1	0	2
mmu-miR-223-3p	66240	Kcne1l	0	0	1	1	0	2
mmu-miR-223-3p	66244	Nemf	0	0	1	1	0	2
mmu-miR-223-3p	66251	Arfgap3	0	1	0	1	0	2
mmu-miR-223-3p	66256	Ssr2	0	1	0	1	0	2
mmu-miR-223-3p	66262	Ing5	0	0	1	1	0	2
mmu-miR-223-3p	66294	Fam3a	0	0	1	1	0	2
mmu-miR-223-3p	66306	Fam53c	1	0	0	1	0	2
mmu-miR-223-3p	66315	Senp7	1	0	0	1	0	2
mmu-miR-223-3p	66320	Tmem208	0	1	0	1	0	2
mmu-miR-223-3p	66322	1700011A15Rik	0	0	1	1	0	2
mmu-miR-223-3p	66329	Susd3	0	0	1	1	0	2
mmu-miR-223-3p	66335	Atp6v1c1	1	0	0	1	0	2
mmu-miR-223-3p	66343	Tmem177	0	0	1	1	0	2
mmu-miR-223-3p	66350	Pla2g12a	1	0	0	1	0	2
mmu-miR-223-3p	66352	Blzf1	1	0	0	1	0	2
mmu-miR-223-3p	66365	Ccdc90b	1	0	0	1	0	2
mmu-miR-223-3p	66367	2310022A10Rik	1	0	0	1	0	2
mmu-miR-223-3p	66368	Rtca	0	0	1	1	0	2
mmu-miR-223-3p	66385	Ppp1r7	0	1	0	1	0	2
mmu-miR-223-3p	66397	Sar1b	1	0	1	0	0	2
mmu-miR-223-3p	66408	Aptx	1	0	0	1	0	2
mmu-miR-223-3p	66425	Pcp4l1	1	0	0	1	0	2
mmu-miR-223-3p	66435	Uggt2	0	1	0	1	0	2
mmu-miR-223-3p	66439	2010012O05Rik	0	0	1	1	0	2
mmu-miR-223-3p	66440	Cdc26	0	0	1	1	0	2
mmu-miR-223-3p	66441	Magohb	0	1	0	1	0	2
mmu-miR-223-3p	66442	Spc25	0	0	1	1	0	2
mmu-miR-223-3p	66451	2610528J11Rik	0	1	0	1	0	2
mmu-miR-223-3p	66464	Taf12	0	1	0	1	0	2
mmu-miR-223-3p	66467	Gtf2h5	0	0	1	1	0	2
mmu-miR-223-3p	66468	Ska1	0	0	1	1	0	2
mmu-miR-223-3p	66471	Anp32e	0	0	1	1	0	2
mmu-miR-223-3p	66479	1700029F12Rik	0	1	0	1	0	2
mmu-miR-223-3p	66482	Exoc2	1	0	0	1	0	2
mmu-miR-223-3p	66491	Polr2l	1	0	0	1	0	2
mmu-miR-223-3p	66493	Mrpl51	0	0	1	1	0	2
mmu-miR-223-3p	66538	Rps19bp1	1	0	0	1	0	2
mmu-miR-223-3p	66569	Gdpd1	0	0	1	1	0	2
mmu-miR-223-3p	66573	Dzip1	1	0	0	1	0	2
mmu-miR-223-3p	66578	Mis18a	1	0	0	1	0	2
mmu-miR-223-3p	66593	Diablo	0	0	1	1	0	2

mmu-miR-223-3p	66622	Ubr7	0	0	1	1	0	2
mmu-miR-223-3p	66624	Spcs2	0	0	1	1	0	2
mmu-miR-223-3p	66626	Cdip1	1	0	0	1	0	2
mmu-miR-223-3p	66643	Lix1	1	0	0	1	0	2
mmu-miR-223-3p	66660	Sltm	0	1	0	1	0	2
mmu-miR-223-3p	66661	Srp72	0	0	1	1	0	2
mmu-miR-223-3p	66665	Msantd3	0	0	1	1	0	2
mmu-miR-223-3p	66690	Tmem186	0	0	1	1	0	2
mmu-miR-223-3p	66696	Snx31	0	0	1	1	0	2
mmu-miR-223-3p	66700	Chmp3	0	0	1	1	0	2
mmu-miR-223-3p	66701	Spryd4	0	0	1	1	0	2
mmu-miR-223-3p	66713	Actr2	1	0	0	1	0	2
mmu-miR-223-3p	66714	4921524J17Rik	1	0	1	0	0	2
mmu-miR-223-3p	66743	Rnf220	0	0	1	1	0	2
mmu-miR-223-3p	66753	Erlec1	0	0	1	1	0	2
mmu-miR-223-3p	66765	4933411K16Rik	0	1	0	1	0	2
mmu-miR-223-3p	66775	Ptplad2	0	0	1	1	0	2
mmu-miR-223-3p	66786	Olfr701	0	0	1	1	0	2
mmu-miR-223-3p	66787	Gskip	1	0	0	1	0	2
mmu-miR-223-3p	66795	Atg10	1	0	0	1	0	2
mmu-miR-223-3p	66799	Ube2w	0	0	1	1	0	2
mmu-miR-223-3p	66805	Tspan1	0	0	1	1	0	2
mmu-miR-223-3p	66809	Krt20	1	0	0	1	0	2
mmu-miR-223-3p	66810	Rbm22	1	0	0	1	0	2
mmu-miR-223-3p	66812	Ppcdc	0	0	1	1	0	2
mmu-miR-223-3p	66826	Taz	1	0	0	1	0	2
mmu-miR-223-3p	66827	Ttc1	0	0	1	1	0	2
mmu-miR-223-3p	66839	0610009O20Rik	0	0	1	1	0	2
mmu-miR-223-3p	66844	Ormdl2	1	0	0	1	0	2
mmu-miR-223-3p	66847	Hint3	0	1	0	1	0	2
mmu-miR-223-3p	66863	Lztr1	0	1	0	1	0	2
mmu-miR-223-3p	66875	Swt1	1	0	1	0	0	2
mmu-miR-223-3p	66877	Crnkl1	0	0	1	1	0	2
mmu-miR-223-3p	66878	Riok3	0	0	1	1	0	2
mmu-miR-223-3p	66880	Rsrc1	0	0	1	1	0	2
mmu-miR-223-3p	66889	Rnf128	1	0	0	1	0	2
mmu-miR-223-3p	66897	Naa16	0	0	1	1	0	2
mmu-miR-223-3p	66911	Nudt16l1	1	0	0	1	0	2
mmu-miR-223-3p	66912	Bzw2	1	0	0	1	0	2
mmu-miR-223-3p	66917	Chordc1	1	0	0	1	0	2
mmu-miR-223-3p	66926	Trmt6	1	0	0	1	0	2
mmu-miR-223-3p	66929	Asf1b	0	0	1	1	0	2
mmu-miR-223-3p	66930	Fank1	1	0	0	1	0	2
mmu-miR-223-3p	66932	Rexo1	0	0	1	1	0	2
mmu-miR-223-3p	66939	Aagab	0	0	1	1	0	2
mmu-miR-223-3p	66942	Ddx18	0	0	1	1	0	2
mmu-miR-223-3p	66943	Pqlc1	1	0	0	1	0	2
mmu-miR-223-3p	66948	Acad8	1	0	0	1	0	2
mmu-miR-223-3p	66949	Trim59	0	1	0	1	0	2
mmu-miR-223-3p	66950	Tmem206	0	0	1	1	0	2
mmu-miR-223-3p	66953	Cdca7	0	0	1	1	0	2
mmu-miR-223-3p	66960	Fam188a	0	1	0	1	0	2
mmu-miR-223-3p	66973	Mrps18b	0	0	1	1	0	2
mmu-miR-223-3p	66977	Nuf2	0	0	1	1	0	2
mmu-miR-223-3p	66983	Zfp830	1	0	0	1	0	2

mmu-miR-223-3p	66995	Zcchc18	1	0	0	1	0	2
mmu-miR-223-3p	67011	Mettl6	0	0	1	1	0	2
mmu-miR-223-3p	67014	Mina	0	1	0	1	0	2
mmu-miR-223-3p	67016	Tbc1d2b	0	0	1	1	0	2
mmu-miR-223-3p	67020	Tmem88	1	0	0	1	0	2
mmu-miR-223-3p	67028	2610002M06Rik	1	0	0	1	0	2
mmu-miR-223-3p	67036	Mrpl45	1	0	0	1	0	2
mmu-miR-223-3p	67038	2010109I03Rik	1	0	0	1	0	2
mmu-miR-223-3p	67044	Higd2a	0	0	1	1	0	2
mmu-miR-223-3p	67045	Riok2	1	0	0	1	0	2
mmu-miR-223-3p	67048	Vma21	0	0	1	1	0	2
mmu-miR-223-3p	67064	Chmp1b	0	0	1	1	0	2
mmu-miR-223-3p	67072	Cdc37l1	1	0	0	1	0	2
mmu-miR-223-3p	67075	Magt1	1	0	0	1	0	2
mmu-miR-223-3p	67103	Ptgr1	1	1	0	0	0	2
mmu-miR-223-3p	67111	Naaa	1	0	0	1	0	2
mmu-miR-223-3p	67117	Dynlt3	0	0	1	1	0	2
mmu-miR-223-3p	67120	Ttc14	1	0	0	1	0	2
mmu-miR-223-3p	67126	Atp5e	0	0	1	1	0	2
mmu-miR-223-3p	67131	Acbd4	0	1	0	1	0	2
mmu-miR-223-3p	67136	Kbtbd4	0	0	1	1	0	2
mmu-miR-223-3p	67141	Fbxo5	1	0	0	1	0	2
mmu-miR-223-3p	67143	Ikzf5	1	0	0	1	0	2
mmu-miR-223-3p	67155	Smarca2	1	0	0	1	0	2
mmu-miR-223-3p	67157	2610301B20Rik	0	0	1	1	0	2
mmu-miR-223-3p	67166	Arl8b	0	0	1	1	0	2
mmu-miR-223-3p	67177	Cdt1	1	0	0	1	0	2
mmu-miR-223-3p	67179	Ccdc25	0	0	1	1	0	2
mmu-miR-223-3p	67182	Pdzk1ip1	0	0	1	1	0	2
mmu-miR-223-3p	67200	Ccdc77	1	0	0	1	0	2
mmu-miR-223-3p	67201	Glod4	0	1	0	1	0	2
mmu-miR-223-3p	67228	Dph7	1	0	0	1	0	2
mmu-miR-223-3p	67235	Zkscan14	1	0	0	1	0	2
mmu-miR-223-3p	67241	Smc6	1	0	0	1	0	2
mmu-miR-223-3p	67252	Cap2	0	0	1	1	0	2
mmu-miR-223-3p	67260	Cers4	0	0	1	1	0	2
mmu-miR-223-3p	67285	Cwc27	0	0	1	1	0	2
mmu-miR-223-3p	67287	Parp6	0	0	1	1	0	2
mmu-miR-223-3p	67291	Ccdc137	0	0	1	1	0	2
mmu-miR-223-3p	67333	Stk35	1	0	0	1	0	2
mmu-miR-223-3p	67365	Hdhd1a	0	0	1	1	0	2
mmu-miR-223-3p	67367	Paxbp1	1	0	0	1	0	2
mmu-miR-223-3p	67374	Jam2	1	0	0	1	0	2
mmu-miR-223-3p	67378	Bbs2	0	0	1	1	0	2
mmu-miR-223-3p	67384	Bag4	1	0	0	1	0	2
mmu-miR-223-3p	67393	Cxxc5	1	0	0	1	0	2
mmu-miR-223-3p	67399	Pdlim7	0	1	0	1	0	2
mmu-miR-223-3p	67417	Ears2	1	0	0	1	0	2
mmu-miR-223-3p	67420	Far1	1	0	0	1	0	2
mmu-miR-223-3p	67422	Dhdds	1	0	1	0	0	2
mmu-miR-223-3p	67440	Mtpap	1	0	0	1	0	2
mmu-miR-223-3p	67443	Map1lc3b	1	0	0	1	0	2
mmu-miR-223-3p	67448	Plxdc2	1	0	0	1	0	2
mmu-miR-223-3p	67452	Pnpla8	0	0	1	1	0	2
mmu-miR-223-3p	67463	Poc5	0	0	1	1	0	2

mmu-miR-223-3p	67465	Sf3a1	0	0	1	1	0	2
mmu-miR-223-3p	67467	Gpalpp1	1	0	0	1	0	2
mmu-miR-223-3p	67470	Abcg8	1	0	0	1	0	2
mmu-miR-223-3p	67471	Gpatch1	0	1	0	1	0	2
mmu-miR-223-3p	67493	Mettl16	0	1	0	1	0	2
mmu-miR-223-3p	67510	Tvp23b	0	0	1	1	0	2
mmu-miR-223-3p	67525	Trim62	0	0	1	1	0	2
mmu-miR-223-3p	67529	Fgfr1op2	0	0	1	1	0	2
mmu-miR-223-3p	67533	Ppfibp1	0	0	1	1	0	2
mmu-miR-223-3p	67543	Pabpc6	1	0	0	1	0	2
mmu-miR-223-3p	67544	Fam120b	1	0	0	1	0	2
mmu-miR-223-3p	67561	Wdr48	0	0	1	1	0	2
mmu-miR-223-3p	67569	Mgat4c	0	0	1	1	0	2
mmu-miR-223-3p	67591	Ubl4b	0	0	1	1	0	2
mmu-miR-223-3p	67592	4930524B15Rik	0	1	0	1	0	2
mmu-miR-223-3p	67593	4930519G04Rik	0	0	1	1	0	2
mmu-miR-223-3p	67602	Necap1	0	0	1	1	0	2
mmu-miR-223-3p	67608	Narf	0	0	1	1	0	2
mmu-miR-223-3p	67610	Rspry1	0	0	1	1	0	2
mmu-miR-223-3p	67620	Lrp2bp	1	0	0	1	0	2
mmu-miR-223-3p	67623	Tm7sf3	0	0	1	1	0	2
mmu-miR-223-3p	67636	Lyrm5	0	0	1	1	0	2
mmu-miR-223-3p	67684	Luc7l3	0	0	1	1	0	2
mmu-miR-223-3p	67703	Kirrel3	0	0	1	1	0	2
mmu-miR-223-3p	67704	1810037I17Rik	0	0	1	1	0	2
mmu-miR-223-3p	67709	Reg4	1	0	0	1	0	2
mmu-miR-223-3p	67713	Dnajc19	1	1	0	0	0	2
mmu-miR-223-3p	67715	2010106E10Rik	1	0	0	1	0	2
mmu-miR-223-3p	67725	Nudt13	0	0	1	1	0	2
mmu-miR-223-3p	67727	Stx17	1	0	0	1	0	2
mmu-miR-223-3p	67739	Slc48a1	0	0	1	1	0	2
mmu-miR-223-3p	67747	Ribc2	0	1	0	1	0	2
mmu-miR-223-3p	67749	Mgarp	1	0	0	1	0	2
mmu-miR-223-3p	67760	Slc38a2	0	0	1	1	0	2
mmu-miR-223-3p	67768	N6amt1	0	0	1	1	0	2
mmu-miR-223-3p	67770	Caap1	1	0	1	0	0	2
mmu-miR-223-3p	67772	Chd8	1	0	0	1	0	2
mmu-miR-223-3p	67776	Vwa5a	0	0	1	1	0	2
mmu-miR-223-3p	67781	Ilf2	1	1	0	0	0	2
mmu-miR-223-3p	67785	Zmym4	0	0	1	1	0	2
mmu-miR-223-3p	67790	Rab39b	0	1	0	1	0	2
mmu-miR-223-3p	67800	Dgat2	1	0	0	1	0	2
mmu-miR-223-3p	67801	Pllp	0	0	1	1	0	2
mmu-miR-223-3p	67811	Poldip2	0	0	1	1	0	2
mmu-miR-223-3p	67821	Atp1b4	0	0	1	1	0	2
mmu-miR-223-3p	67830	Rer1	0	0	1	1	0	2
mmu-miR-223-3p	67836	Wdr83	0	0	1	1	0	2
mmu-miR-223-3p	67839	Gpsm1	1	0	0	1	0	2
mmu-miR-223-3p	67854	Slco6b1	0	0	1	1	0	2
mmu-miR-223-3p	67856	Echdc3	0	0	1	1	0	2
mmu-miR-223-3p	67857	Ppp6c	0	0	1	1	0	2
mmu-miR-223-3p	67861	Akr1b10	0	0	1	1	0	2
mmu-miR-223-3p	67863	Slc25a11	1	0	0	1	0	2
mmu-miR-223-3p	67869	Paip2	1	0	0	1	0	2
mmu-miR-223-3p	67871	Mrrf	1	0	0	1	0	2

mmu-miR-223-3p	67888	Tmem100	1	0	0	1	0	2
mmu-miR-223-3p	67889	Rbm18	1	0	0	1	0	2
mmu-miR-223-3p	67914	Coq9	1	0	0	1	0	2
mmu-miR-223-3p	67920	Mak16	1	0	0	1	0	2
mmu-miR-223-3p	67922	Fam32a	0	0	1	1	0	2
mmu-miR-223-3p	67923	Tceb1	0	0	1	1	0	2
mmu-miR-223-3p	67936	Wdr55	1	0	0	1	0	2
mmu-miR-223-3p	67939	Prorsd1	0	0	1	1	0	2
mmu-miR-223-3p	67948	Fbxo28	0	0	1	1	0	2
mmu-miR-223-3p	67949	Mki67ip	0	1	1	0	0	2
mmu-miR-223-3p	67958	U2surp	1	0	0	1	0	2
mmu-miR-223-3p	67979	Atad1	1	0	0	1	0	2
mmu-miR-223-3p	67981	Hormad1	1	0	0	1	0	2
mmu-miR-223-3p	67991	Nacc2	1	0	0	1	0	2
mmu-miR-223-3p	67996	Srsf6	0	0	1	1	0	2
mmu-miR-223-3p	68017	Ftsj2	0	0	1	1	0	2
mmu-miR-223-3p	68018	Col4a3bp	1	0	0	1	0	2
mmu-miR-223-3p	68020	Apopt1	0	1	0	1	0	2
mmu-miR-223-3p	68026	2810417H13Rik	0	0	1	1	0	2
mmu-miR-223-3p	68038	Chid1	0	0	1	1	0	2
mmu-miR-223-3p	68041	Mid1ip1	0	0	1	1	0	2
mmu-miR-223-3p	68044	Chac2	1	0	0	1	0	2
mmu-miR-223-3p	68048	Aen	0	1	0	1	0	2
mmu-miR-223-3p	68075	Lurap1	0	1	0	1	0	2
mmu-miR-223-3p	68092	Ncbp2	1	0	0	1	0	2
mmu-miR-223-3p	68095	Ociad1	0	0	1	1	0	2
mmu-miR-223-3p	68114	Mum1	0	0	1	1	0	2
mmu-miR-223-3p	68127	B230217C12Rik	1	0	0	1	0	2
mmu-miR-223-3p	68133	Gcsh	1	0	0	1	0	2
mmu-miR-223-3p	68134	Upf3b	0	1	0	1	0	2
mmu-miR-223-3p	68137	Kdelr1	0	0	1	1	0	2
mmu-miR-223-3p	68142	Ino80	0	1	0	1	0	2
mmu-miR-223-3p	68146	Arl13b	0	0	1	1	0	2
mmu-miR-223-3p	68149	Otub2	0	1	0	1	0	2
mmu-miR-223-3p	68184	Denr	0	0	1	1	0	2
mmu-miR-223-3p	68185	Coa4	0	1	0	1	0	2
mmu-miR-223-3p	68226	Efcab2	1	0	0	1	0	2
mmu-miR-223-3p	68273	Pomgnt1	0	1	0	1	0	2
mmu-miR-223-3p	68276	Toe1	0	0	1	1	0	2
mmu-miR-223-3p	68281	4930430F08Rik	1	0	0	1	0	2
mmu-miR-223-3p	68283	9530077C05Rik	0	0	1	1	0	2
mmu-miR-223-3p	68291	Mto1	0	1	0	1	0	2
mmu-miR-223-3p	68295	Aar2	0	0	1	1	0	2
mmu-miR-223-3p	68310	Zmym1	1	0	0	1	0	2
mmu-miR-223-3p	68316	Apoo	0	0	1	1	0	2
mmu-miR-223-3p	68348	Serpina1f	1	0	0	1	0	2
mmu-miR-223-3p	68350	Mul1	0	0	1	1	0	2
mmu-miR-223-3p	68421	Lmbrd1	0	0	1	1	0	2
mmu-miR-223-3p	68427	Slc39a13	0	1	0	1	0	2
mmu-miR-223-3p	68431	Fbxl15	1	0	0	1	0	2
mmu-miR-223-3p	68440	Dusp23	0	0	1	1	0	2
mmu-miR-223-3p	68481	Mpzl1	1	0	0	1	0	2
mmu-miR-223-3p	68497	Arel1	0	0	1	1	0	2
mmu-miR-223-3p	68514	Micu2	0	0	1	1	0	2
mmu-miR-223-3p	68519	Eml1	1	0	0	1	0	2

mmu-miR-223-3p	68552	Smim14	1	0	0	1	0	2
mmu-miR-223-3p	68554	1110001A16Rik	1	0	0	1	0	2
mmu-miR-223-3p	68606	Ppm1f	1	0	0	1	0	2
mmu-miR-223-3p	68614	Letmd1	0	0	1	1	0	2
mmu-miR-223-3p	68632	Myct1	1	0	0	1	0	2
mmu-miR-223-3p	68636	Fahd1	1	0	0	1	0	2
mmu-miR-223-3p	68652	Tab2	0	1	1	0	0	2
mmu-miR-223-3p	68666	Svop	0	0	1	1	0	2
mmu-miR-223-3p	68668	Klk5	0	0	1	1	0	2
mmu-miR-223-3p	68671	Pcyt2	0	0	1	1	0	2
mmu-miR-223-3p	68691	Kansl1l	1	0	0	1	0	2
mmu-miR-223-3p	68724	Arl8a	1	0	0	1	0	2
mmu-miR-223-3p	68738	Acss1	1	0	0	1	0	2
mmu-miR-223-3p	68743	Anln	0	1	0	1	0	2
mmu-miR-223-3p	68744	Zfp740	0	0	1	1	0	2
mmu-miR-223-3p	68767	Wash	1	0	0	1	0	2
mmu-miR-223-3p	68776	Taf11	0	0	1	1	0	2
mmu-miR-223-3p	68778	Gucd1	0	0	1	1	0	2
mmu-miR-223-3p	68792	Srpx2	1	0	0	1	0	2
mmu-miR-223-3p	68794	Flnc	0	0	1	1	0	2
mmu-miR-223-3p	68833	Pdcl3	0	0	1	1	0	2
mmu-miR-223-3p	68839	Ankrd46	1	0	1	0	0	2
mmu-miR-223-3p	68846	Rnf208	0	0	1	1	0	2
mmu-miR-223-3p	68854	Asb11	1	0	0	1	0	2
mmu-miR-223-3p	68875	Tmcc2	0	1	0	1	0	2
mmu-miR-223-3p	68910	Zfp467	0	1	0	1	0	2
mmu-miR-223-3p	68915	Vars2	0	0	1	1	0	2
mmu-miR-223-3p	68916	Cdkal1	1	0	0	1	0	2
mmu-miR-223-3p	68927	Ptcd2	0	0	1	1	0	2
mmu-miR-223-3p	68939	Rasl11b	1	0	0	1	0	2
mmu-miR-223-3p	68964	Ctc1	0	1	0	1	0	2
mmu-miR-223-3p	68979	Nol11	0	0	1	1	0	2
mmu-miR-223-3p	69017	Prrt2	1	0	0	1	0	2
mmu-miR-223-3p	69020	Zfp707	0	0	1	1	0	2
mmu-miR-223-3p	69034	Nupr1l	0	1	0	1	0	2
mmu-miR-223-3p	69046	Isca1	1	0	0	1	0	2
mmu-miR-223-3p	69066	1810010H24Rik	1	0	0	1	0	2
mmu-miR-223-3p	69068	1810011O10Rik	0	0	1	1	0	2
mmu-miR-223-3p	69071	Tmem97	0	0	1	1	0	2
mmu-miR-223-3p	69072	Ebna1bp2	1	0	0	1	0	2
mmu-miR-223-3p	69083	Sult1c2	1	0	0	1	0	2
mmu-miR-223-3p	69085	Zcchc9	0	0	1	1	0	2
mmu-miR-223-3p	69090	Ascc1	0	0	1	1	0	2
mmu-miR-223-3p	69097	Trim15	0	0	1	1	0	2
mmu-miR-223-3p	69101	Ydjc	1	0	0	1	0	2
mmu-miR-223-3p	69117	Adh6a	0	1	0	1	0	2
mmu-miR-223-3p	69123	Eci3	0	0	1	1	0	2
mmu-miR-223-3p	69125	Cnot8	1	0	0	1	0	2
mmu-miR-223-3p	69131	Cdk12	1	0	0	1	0	2
mmu-miR-223-3p	69137	Vstm5	0	0	1	1	0	2
mmu-miR-223-3p	69156	Comtd1	1	0	0	1	0	2
mmu-miR-223-3p	69159	Rhebl1	1	0	0	1	0	2
mmu-miR-223-3p	69171	Cnppd1	0	0	1	1	0	2
mmu-miR-223-3p	69178	Snx5	0	0	1	1	0	2
mmu-miR-223-3p	69188	Kmt2e	0	1	0	1	0	2

mmu-miR-223-3p	69215	Sat2	0	0	1	1	0	2
mmu-miR-223-3p	69217	Plekha4	0	1	0	1	0	2
mmu-miR-223-3p	69219	Ddah1	0	1	0	1	0	2
mmu-miR-223-3p	69237	Gtpbp4	0	0	1	1	0	2
mmu-miR-223-3p	69288	Rhobtb1	0	0	1	1	0	2
mmu-miR-223-3p	69309	Slc16a13	0	0	1	1	0	2
mmu-miR-223-3p	69376	Zpbp2	1	0	0	1	0	2
mmu-miR-223-3p	69387	Dnajb13	0	1	0	1	0	2
mmu-miR-223-3p	69399	1700025G04Rik	1	0	0	1	0	2
mmu-miR-223-3p	69456	Commd10	0	0	1	1	0	2
mmu-miR-223-3p	69457	2310005G13Rik	0	0	1	1	0	2
mmu-miR-223-3p	69480	Ttc9	0	0	1	1	0	2
mmu-miR-223-3p	69527	Mrps9	0	0	1	1	0	2
mmu-miR-223-3p	69528	1700030J22Rik	0	0	1	1	0	2
mmu-miR-223-3p	69537	Dnase1l1	0	0	1	1	0	2
mmu-miR-223-3p	69538	Antxr1	1	0	0	1	0	2
mmu-miR-223-3p	69539	Trnp1	0	1	1	0	0	2
mmu-miR-223-3p	69568	Vkorc1l1	1	0	0	1	0	2
mmu-miR-223-3p	69572	Mfsd3	1	0	0	1	0	2
mmu-miR-223-3p	69583	Tnfsf13	1	0	0	1	0	2
mmu-miR-223-3p	69634	Clybl	1	0	1	0	0	2
mmu-miR-223-3p	69655	Cd164l2	0	1	0	1	0	2
mmu-miR-223-3p	69672	Txndc15	0	0	1	1	0	2
mmu-miR-223-3p	69674	Mif4gd	0	0	1	1	0	2
mmu-miR-223-3p	69719	Cad	0	1	1	0	0	2
mmu-miR-223-3p	69727	Usp46	1	0	1	0	0	2
mmu-miR-223-3p	69769	Tnfaip8l2	0	0	1	1	0	2
mmu-miR-223-3p	69865	A1cf	0	1	0	1	0	2
mmu-miR-223-3p	69882	Vwa9	0	1	0	1	0	2
mmu-miR-223-3p	69938	Scrn1	0	1	0	1	0	2
mmu-miR-223-3p	69944	2810021J22Rik	1	0	0	1	0	2
mmu-miR-223-3p	69981	Tmem30a	1	0	0	1	0	2
mmu-miR-223-3p	70012	Cep85	0	0	1	1	0	2
mmu-miR-223-3p	70026	Tspo2	1	0	0	1	0	2
mmu-miR-223-3p	70059	Degs2	0	0	1	1	0	2
mmu-miR-223-3p	70086	Cysltr2	0	0	1	1	0	2
mmu-miR-223-3p	70093	Ube2q1	0	0	1	1	0	2
mmu-miR-223-3p	70101	Cyp4f16	0	1	0	1	0	2
mmu-miR-223-3p	70110	Ifi35	0	1	0	1	0	2
mmu-miR-223-3p	70122	Mllt3	0	1	0	1	0	2
mmu-miR-223-3p	70178	Abhd17c	0	0	1	1	0	2
mmu-miR-223-3p	70209	Tmem143	1	0	0	1	0	2
mmu-miR-223-3p	70238	Rnf168	0	0	1	1	0	2
mmu-miR-223-3p	70297	Gcc2	0	1	1	0	0	2
mmu-miR-223-3p	70310	Plscr3	0	0	1	1	0	2
mmu-miR-223-3p	70333	Cd3eap	1	0	0	1	0	2
mmu-miR-223-3p	70350	Basp1	1	0	0	1	0	2
mmu-miR-223-3p	70375	Ica1l	1	0	0	1	0	2
mmu-miR-223-3p	70381	Tecpr1	1	0	0	1	0	2
mmu-miR-223-3p	70383	Cox10	1	0	0	1	0	2
mmu-miR-223-3p	70385	Spdl1	0	0	1	1	0	2
mmu-miR-223-3p	70422	Ints2	1	0	0	1	0	2
mmu-miR-223-3p	70423	Tspan15	0	0	1	1	0	2
mmu-miR-223-3p	70428	Polr3b	0	0	1	1	0	2
mmu-miR-223-3p	70439	Taf15	0	0	1	1	0	2

mmu-miR-223-3p	70478	Mipep	0	0	1	1	0	2
mmu-miR-223-3p	70481	Pnma1	1	0	0	1	0	2
mmu-miR-223-3p	70503	Ddo	0	0	1	1	0	2
mmu-miR-223-3p	70511	Fam86	0	0	1	1	0	2
mmu-miR-223-3p	70533	Btf3l4	1	0	0	1	0	2
mmu-miR-223-3p	70551	Tmtc4	1	1	0	0	0	2
mmu-miR-223-3p	70556	Slc25a33	1	0	0	1	0	2
mmu-miR-223-3p	70561	Txndc16	1	0	1	0	0	2
mmu-miR-223-3p	70572	Ipo5	0	0	1	1	0	2
mmu-miR-223-3p	70573	Tbccd1	0	0	1	1	0	2
mmu-miR-223-3p	70574	Cpm	1	0	0	1	0	2
mmu-miR-223-3p	70579	Zc3h11a	0	0	1	1	0	2
mmu-miR-223-3p	70599	Ssfa2	0	1	0	1	0	2
mmu-miR-223-3p	70604	Dnajb14	0	1	0	1	0	2
mmu-miR-223-3p	70611	Fbxo33	0	1	0	1	0	2
mmu-miR-223-3p	70615	Ankrd24	0	0	1	1	0	2
mmu-miR-223-3p	70645	Oip5	1	0	1	0	0	2
mmu-miR-223-3p	70661	Sik3	1	0	0	1	0	2
mmu-miR-223-3p	70673	Prdm16	0	0	1	1	0	2
mmu-miR-223-3p	70676	Gulp1	0	1	0	1	0	2
mmu-miR-223-3p	70717	Medag	1	0	0	1	0	2
mmu-miR-223-3p	70727	Rasgef1a	1	0	0	1	0	2
mmu-miR-223-3p	70729	Nos1ap	0	0	1	1	0	2
mmu-miR-223-3p	70737	Cgn	0	1	0	1	0	2
mmu-miR-223-3p	70747	Tspan2	1	0	0	1	0	2
mmu-miR-223-3p	70762	Dclk2	0	0	1	1	0	2
mmu-miR-223-3p	70788	Klhl30	1	0	0	1	0	2
mmu-miR-223-3p	70789	Kynu	0	1	0	1	0	2
mmu-miR-223-3p	70809	Clec2g	0	0	1	1	0	2
mmu-miR-223-3p	70829	Ccdc93	1	0	0	1	0	2
mmu-miR-223-3p	70859	Lrrc63	1	0	0	1	0	2
mmu-miR-223-3p	70861	Akr1cl	0	1	0	1	0	2
mmu-miR-223-3p	70866	Slco6d1	1	0	0	1	0	2
mmu-miR-223-3p	70892	Ttll7	1	0	0	1	0	2
mmu-miR-223-3p	70902	Lpcat2b	0	0	1	1	0	2
mmu-miR-223-3p	70911	Phyhipl	0	1	0	1	0	2
mmu-miR-223-3p	70967	Eva1c	1	0	0	1	0	2
mmu-miR-223-3p	70977	Cabs1	0	1	0	1	0	2
mmu-miR-223-3p	70981	4931423N10Rik	1	0	0	1	0	2
mmu-miR-223-3p	70984	4931406C07Rik	1	0	0	1	0	2
mmu-miR-223-3p	70989	4931429I11Rik	0	0	1	1	0	2
mmu-miR-223-3p	70998	Phf6	1	0	0	1	0	2
mmu-miR-223-3p	71041	Pcgf6	1	0	0	1	0	2
mmu-miR-223-3p	71069	Stox2	1	0	0	1	0	2
mmu-miR-223-3p	71091	Cdkl1	1	0	0	1	0	2
mmu-miR-223-3p	71096	Sntg1	1	0	0	1	0	2
mmu-miR-223-3p	71130	Sh2d6	1	0	0	1	0	2
mmu-miR-223-3p	71131	Zfp689	0	0	1	1	0	2
mmu-miR-223-3p	71147	Oxsm	1	0	0	1	0	2
mmu-miR-223-3p	71183	Clec12b	1	0	0	1	0	2
mmu-miR-223-3p	71213	Cage1	1	0	0	0	1	2
mmu-miR-223-3p	71228	Dlg5	0	1	0	1	0	2
mmu-miR-223-3p	71263	Mro	0	0	1	1	0	2
mmu-miR-223-3p	71281	Apobec4	1	0	1	0	0	2
mmu-miR-223-3p	71369	Krtap16-3	0	0	1	1	0	2

mmu-miR-223-3p	71373	Prr16	1	0	0	1	0	2
mmu-miR-223-3p	71375	Foxn3	1	0	0	1	0	2
mmu-miR-223-3p	71389	Chd6	0	0	1	1	0	2
mmu-miR-223-3p	71435	Arhgap21	0	0	1	1	0	2
mmu-miR-223-3p	71446	Wrb	0	0	1	1	0	2
mmu-miR-223-3p	71448	Tmem80	1	0	0	1	0	2
mmu-miR-223-3p	71481	Alpk1	1	0	0	1	0	2
mmu-miR-223-3p	71517	9030624J02Rik	0	1	0	1	0	2
mmu-miR-223-3p	71566	Clmp	0	0	1	1	0	2
mmu-miR-223-3p	71583	9130008F23Rik	1	0	0	1	0	2
mmu-miR-223-3p	71617	9130011E15Rik	0	0	1	1	0	2
mmu-miR-223-3p	71643	4930422G04Rik	0	1	0	1	0	2
mmu-miR-223-3p	71667	Tmem248	0	0	1	1	0	2
mmu-miR-223-3p	71673	Rnf215	0	0	1	1	0	2
mmu-miR-223-3p	71682	Wdr27	1	0	0	1	0	2
mmu-miR-223-3p	71687	Tmem25	1	0	0	1	0	2
mmu-miR-223-3p	71703	Armcx3	0	0	1	1	0	2
mmu-miR-223-3p	71707	Ubiad1	0	0	1	1	0	2
mmu-miR-223-3p	71709	Syde1	1	0	0	1	0	2
mmu-miR-223-3p	71712	Dram1	1	0	0	1	0	2
mmu-miR-223-3p	71720	Osbpl3	1	0	0	1	0	2
mmu-miR-223-3p	71733	Susd2	1	0	0	1	0	2
mmu-miR-223-3p	71740	Pvrl4	1	0	0	1	0	2
mmu-miR-223-3p	71745	Cul2	1	0	0	1	0	2
mmu-miR-223-3p	71750	R3hdm2	0	1	0	1	0	2
mmu-miR-223-3p	71755	Dhdh	1	0	0	1	0	2
mmu-miR-223-3p	71760	Etnppl	1	0	0	1	0	2
mmu-miR-223-3p	71761	Amdhd1	1	0	0	1	0	2
mmu-miR-223-3p	71764	C2cd2l	0	0	1	1	0	2
mmu-miR-223-3p	71766	Raver1	0	0	1	1	0	2
mmu-miR-223-3p	71769	Bbs10	1	0	0	1	0	2
mmu-miR-223-3p	71782	Ankle2	1	0	0	1	0	2
mmu-miR-223-3p	71793	Ints12	0	0	1	1	0	2
mmu-miR-223-3p	71795	Pitpnc1	1	0	0	1	0	2
mmu-miR-223-3p	71807	Tars2	0	0	1	1	0	2
mmu-miR-223-3p	71810	Ranbp3	0	0	1	1	0	2
mmu-miR-223-3p	71820	Wdr34	0	0	1	1	0	2
mmu-miR-223-3p	71835	Lancl2	0	1	1	0	0	2
mmu-miR-223-3p	71838	Phf7	1	0	0	1	0	2
mmu-miR-223-3p	71853	Pdia6	1	0	0	1	0	2
mmu-miR-223-3p	71864	Fam217a	0	1	0	1	0	2
mmu-miR-223-3p	71869	Serpinb12	0	0	1	1	0	2
mmu-miR-223-3p	71887	Ppm1j	0	0	1	1	0	2
mmu-miR-223-3p	71904	Paqr7	0	1	0	1	0	2
mmu-miR-223-3p	71910	Ppapdc1b	0	0	1	1	0	2
mmu-miR-223-3p	71911	Bdh1	1	0	0	1	0	2
mmu-miR-223-3p	71914	Antxr2	0	0	1	1	0	2
mmu-miR-223-3p	71920	Epgn	0	0	1	1	0	2
mmu-miR-223-3p	71934	Car13	0	0	1	1	0	2
mmu-miR-223-3p	71951	Gpc2	1	0	0	1	0	2
mmu-miR-223-3p	71957	Cpsf3l	0	1	0	1	0	2
mmu-miR-223-3p	71966	Nkiras2	0	0	1	1	0	2
mmu-miR-223-3p	71973	Rbpms2	1	0	0	1	0	2
mmu-miR-223-3p	71989	Rpusd4	1	0	0	1	0	2
mmu-miR-223-3p	71994	Cnn3	0	0	1	1	0	2

mmu-miR-223-3p	72016	1600002H07Rik	0	0	1	1	0	2
mmu-miR-223-3p	72017	Cyb5r1	0	1	0	1	0	2
mmu-miR-223-3p	72018	Fundc1	1	0	0	1	0	2
mmu-miR-223-3p	72023	Cyb561d1	0	1	0	1	0	2
mmu-miR-223-3p	72029	Cnpy3	1	0	0	1	0	2
mmu-miR-223-3p	72043	Sulf2	0	0	1	1	0	2
mmu-miR-223-3p	72046	Urgcp	0	1	0	1	0	2
mmu-miR-223-3p	72056	1810055G02Rik	0	0	1	1	0	2
mmu-miR-223-3p	72058	Igsf5	0	0	1	1	0	2
mmu-miR-223-3p	72074	Anks4b	1	0	0	1	0	2
mmu-miR-223-3p	72082	Cyp2c55	1	0	0	1	0	2
mmu-miR-223-3p	72090	Entpd8	0	0	1	1	0	2
mmu-miR-223-3p	72103	Aplf	1	0	0	1	0	2
mmu-miR-223-3p	72113	Adck1	0	0	1	1	0	2
mmu-miR-223-3p	72114	Zbed3	0	0	1	1	0	2
mmu-miR-223-3p	72119	Tpx2	0	1	0	1	0	2
mmu-miR-223-3p	72121	Dennd2d	1	0	0	1	0	2
mmu-miR-223-3p	72128	2610008E11Rik	0	0	1	1	0	2
mmu-miR-223-3p	72136	Chst14	0	0	1	1	0	2
mmu-miR-223-3p	72139	2610044O15Rik8	1	0	0	1	0	2
mmu-miR-223-3p	72145	Wdfy3	1	0	0	1	0	2
mmu-miR-223-3p	72147	Zbtb46	0	1	0	1	0	2
mmu-miR-223-3p	72149	Strada	0	0	1	1	0	2
mmu-miR-223-3p	72151	Rfc5	0	1	0	1	0	2
mmu-miR-223-3p	72162	Dhx36	1	0	0	1	0	2
mmu-miR-223-3p	72169	Trim29	0	0	1	1	0	2
mmu-miR-223-3p	72199	Mms19	0	1	0	1	0	2
mmu-miR-223-3p	72265	Tram1	0	0	1	1	0	2
mmu-miR-223-3p	72278	Ccpg1	1	0	0	1	0	2
mmu-miR-223-3p	72290	Lsm11	0	0	1	1	0	2
mmu-miR-223-3p	72293	Nkd2	1	0	0	0	1	2
mmu-miR-223-3p	72297	B3gnt3	1	0	0	1	0	2
mmu-miR-223-3p	72320	2510003E04Rik	0	1	0	1	0	2
mmu-miR-223-3p	72323	Asb6	0	0	1	1	0	2
mmu-miR-223-3p	72324	Plxdc1	0	0	1	1	0	2
mmu-miR-223-3p	72355	Cdpf1	0	0	1	1	0	2
mmu-miR-223-3p	72361	Ces2g	0	1	0	1	0	2
mmu-miR-223-3p	72371	2210408I21Rik	0	0	1	1	0	2
mmu-miR-223-3p	72388	Ripk4	0	0	1	1	0	2
mmu-miR-223-3p	72392	Tmem175	0	0	1	1	0	2
mmu-miR-223-3p	72399	Brap	0	0	1	1	0	2
mmu-miR-223-3p	72400	Pinx1	0	0	1	1	0	2
mmu-miR-223-3p	72404	Wdr44	1	0	0	1	0	2
mmu-miR-223-3p	72415	Sgol1	1	0	0	1	0	2
mmu-miR-223-3p	72421	Ttc30b	1	0	0	1	0	2
mmu-miR-223-3p	72425	Katnbl1	0	0	1	1	0	2
mmu-miR-223-3p	72432	Spink5	0	0	1	1	0	2
mmu-miR-223-3p	72486	Rnf219	0	0	1	1	0	2
mmu-miR-223-3p	72502	Cwf19l1	1	0	0	1	0	2
mmu-miR-223-3p	72503	2610507B11Rik	0	0	1	1	0	2
mmu-miR-223-3p	72535	Aldh1b1	0	0	1	1	0	2
mmu-miR-223-3p	72544	Exosc6	1	0	1	0	0	2
mmu-miR-223-3p	72552	Hsdl1	1	0	0	1	0	2
mmu-miR-223-3p	72555	Shisa9	1	0	0	1	0	2
mmu-miR-223-3p	72587	Pan3	0	0	1	1	0	2

mmu-miR-223-3p	72605	Car10	0	0	1	1	0	2
mmu-miR-223-3p	72621	Pdzd11	0	0	1	1	0	2
mmu-miR-223-3p	72630	Hspa12b	0	0	1	1	0	2
mmu-miR-223-3p	72632	Smim18	1	0	0	1	0	2
mmu-miR-223-3p	72640	Mex3a	0	0	1	1	0	2
mmu-miR-223-3p	72654	Ccdc12	1	0	0	1	0	2
mmu-miR-223-3p	72656	Ints8	1	0	0	1	0	2
mmu-miR-223-3p	72674	Adipor1	1	0	0	1	0	2
mmu-miR-223-3p	72692	Hnrnpll	0	0	1	1	0	2
mmu-miR-223-3p	72713	Angptl1	1	0	0	1	0	2
mmu-miR-223-3p	72720	Zfp248	0	0	1	1	0	2
mmu-miR-223-3p	72722	Fam98a	1	0	0	1	0	2
mmu-miR-223-3p	72723	Zfp74	0	0	1	1	0	2
mmu-miR-223-3p	72729	Cdc42se2	0	0	1	1	0	2
mmu-miR-223-3p	72739	Zkscan3	0	1	0	1	0	2
mmu-miR-223-3p	72775	Fance	0	1	0	1	0	2
mmu-miR-223-3p	72780	Rspo3	1	0	0	1	0	2
mmu-miR-223-3p	72826	Fam76b	0	0	1	1	0	2
mmu-miR-223-3p	72828	Ubash3b	0	1	1	0	0	2
mmu-miR-223-3p	72836	Pot1b	0	0	1	1	0	2
mmu-miR-223-3p	72844	Kctd17	1	0	0	1	0	2
mmu-miR-223-3p	72895	Setd5	1	0	0	1	0	2
mmu-miR-223-3p	72899	Macrod2	0	0	1	1	0	2
mmu-miR-223-3p	72947	Phykpl	0	1	0	1	0	2
mmu-miR-223-3p	72958	Zfp493	1	0	0	1	0	2
mmu-miR-223-3p	72981	Prkrir	0	0	1	1	0	2
mmu-miR-223-3p	73024	Emc7	1	0	0	1	0	2
mmu-miR-223-3p	73086	Rps6ka5	1	0	0	1	0	2
mmu-miR-223-3p	73094	Sgip1	1	0	0	1	0	2
mmu-miR-223-3p	73095	Slc25a42	0	0	1	1	0	2
mmu-miR-223-3p	73106	Prss57	1	0	0	1	0	2
mmu-miR-223-3p	73124	Golim4	1	0	1	0	0	2
mmu-miR-223-3p	73132	Slc25a16	0	0	1	1	0	2
mmu-miR-223-3p	73158	Larp1	0	1	0	1	0	2
mmu-miR-223-3p	73173	Pcdh18	0	0	1	1	0	2
mmu-miR-223-3p	73225	Fam118a	0	0	1	1	0	2
mmu-miR-223-3p	73229	3110052M02Rik	1	0	0	1	0	2
mmu-miR-223-3p	73233	Zfp942	1	0	0	1	0	2
mmu-miR-223-3p	73274	Gpbp1	0	1	0	1	0	2
mmu-miR-223-3p	73287	1700040L02Rik	0	0	1	1	0	2
mmu-miR-223-3p	73288	Ccdc132	0	1	0	1	0	2
mmu-miR-223-3p	73296	Rhobtb3	0	0	1	1	0	2
mmu-miR-223-3p	73301	Ttc29	0	0	1	1	0	2
mmu-miR-223-3p	73336	Prss44	0	0	1	1	0	2
mmu-miR-223-3p	73340	Nptxr	1	0	0	1	0	2
mmu-miR-223-3p	73341	Arhgef6	0	0	1	1	0	2
mmu-miR-223-3p	73373	Phospho2	0	0	1	1	0	2
mmu-miR-223-3p	73389	Hbp1	0	0	1	1	0	2
mmu-miR-223-3p	73420	Ccsap	0	0	1	1	0	2
mmu-miR-223-3p	73449	1700066B19Rik	0	0	1	1	0	2
mmu-miR-223-3p	73463	Als2cr11	1	0	0	1	0	2
mmu-miR-223-3p	73467	1700066M21Rik	1	0	0	1	0	2
mmu-miR-223-3p	73481	1700074P13Rik	1	0	1	0	0	2
mmu-miR-223-3p	73532	1700080E11Rik	0	0	1	1	0	2
mmu-miR-223-3p	73545	1700094D03Rik	1	0	0	1	0	2

mmu-miR-223-3p	73569	Vgll3	1	0	0	1	0	2
mmu-miR-223-3p	73635	Ptges3l	0	0	1	1	0	2
mmu-miR-223-3p	73658	Spns1	0	1	0	1	0	2
mmu-miR-223-3p	73660	Cabp4	0	0	1	1	0	2
mmu-miR-223-3p	73666	Thoc3	0	0	1	1	0	2
mmu-miR-223-3p	73668	Ttc21b	0	0	1	1	0	2
mmu-miR-223-3p	73671	Sult6b1	1	0	0	1	0	2
mmu-miR-223-3p	73707	Gucy2g	1	0	0	1	0	2
mmu-miR-223-3p	73712	Dmkn	0	0	1	1	0	2
mmu-miR-223-3p	73724	Mcee	0	0	1	1	0	2
mmu-miR-223-3p	73747	1110034G24Rik	0	1	0	1	0	2
mmu-miR-223-3p	73825	Ppp1r21	1	0	0	1	0	2
mmu-miR-223-3p	73826	Poldip3	1	0	0	1	0	2
mmu-miR-223-3p	73828	Dcaf4	0	0	1	1	0	2
mmu-miR-223-3p	73836	Slc35b2	0	1	0	1	0	2
mmu-miR-223-3p	73844	Ankrd45	1	0	0	1	0	2
mmu-miR-223-3p	73936	Ccdc175	1	0	0	1	0	2
mmu-miR-223-3p	74002	Psd2	0	1	0	1	0	2
mmu-miR-223-3p	74004	Jakmip3	1	0	0	1	0	2
mmu-miR-223-3p	74007	Btbd11	0	1	0	1	0	2
mmu-miR-223-3p	74013	Rftn2	1	0	1	0	0	2
mmu-miR-223-3p	74016	Phf19	0	0	1	1	0	2
mmu-miR-223-3p	74023	Rd3	1	0	0	1	0	2
mmu-miR-223-3p	74025	Nphp3	1	0	0	1	0	2
mmu-miR-223-3p	74048	4632428N05Rik	0	0	1	1	0	2
mmu-miR-223-3p	74075	Syce1	0	1	0	1	0	2
mmu-miR-223-3p	74080	Nmnat3	0	0	1	1	0	2
mmu-miR-223-3p	74081	Cep350	1	0	0	1	0	2
mmu-miR-223-3p	74090	Paqr5	0	0	1	1	0	2
mmu-miR-223-3p	74096	Hvcn1	1	0	0	1	0	2
mmu-miR-223-3p	74111	Rbm19	0	0	1	1	0	2
mmu-miR-223-3p	74121	Acoxl	1	0	0	1	0	2
mmu-miR-223-3p	74122	Tmem43	0	0	1	1	0	2
mmu-miR-223-3p	74125	Armc8	1	0	0	1	0	2
mmu-miR-223-3p	74126	Syvn1	1	0	0	1	0	2
mmu-miR-223-3p	74129	Dmgdh	1	0	0	1	0	2
mmu-miR-223-3p	74134	Cyp2s1	0	0	1	1	0	2
mmu-miR-223-3p	74149	Zfp946	1	0	0	1	0	2
mmu-miR-223-3p	74150	Slc35f5	0	1	0	1	0	2
mmu-miR-223-3p	74152	1300002K09Rik	0	0	1	1	0	2
mmu-miR-223-3p	74178	Stk40	0	0	1	1	0	2
mmu-miR-223-3p	74183	2310042D19Rik	0	0	1	1	0	2
mmu-miR-223-3p	74191	P2ry13	1	0	0	1	0	2
mmu-miR-223-3p	74197	Gtf2e1	1	0	0	1	0	2
mmu-miR-223-3p	74198	Dtx2	0	1	0	1	0	2
mmu-miR-223-3p	74202	Fblim1	1	0	0	1	0	2
mmu-miR-223-3p	74206	Sipa1l3	0	0	1	1	0	2
mmu-miR-223-3p	74211	1700017B05Rik	0	1	0	1	0	2
mmu-miR-223-3p	74243	Slx4ip	1	0	0	1	0	2
mmu-miR-223-3p	74244	Atg7	1	0	0	1	0	2
mmu-miR-223-3p	74245	Ctbs	0	0	1	1	0	2
mmu-miR-223-3p	74254	Gpn1	0	1	0	1	0	2
mmu-miR-223-3p	74255	Smu1	1	0	0	1	0	2
mmu-miR-223-3p	74287	Kcmf1	1	0	0	1	0	2
mmu-miR-223-3p	74297	1700106J16Rik	1	0	0	1	0	2

mmu-miR-223-3p	74302	Mtmr3	1	0	0	1	0	2
mmu-miR-223-3p	74320	Wdr33	1	0	0	1	0	2
mmu-miR-223-3p	74326	Hnrnpr	1	0	0	1	0	2
mmu-miR-223-3p	74340	Ahcyl2	1	0	0	1	0	2
mmu-miR-223-3p	74347	Tldc1	0	0	1	1	0	2
mmu-miR-223-3p	74349	Fam160a2	1	0	0	1	0	2
mmu-miR-223-3p	74374	Clec16a	0	0	1	1	0	2
mmu-miR-223-3p	74385	Ap5m1	0	0	1	1	0	2
mmu-miR-223-3p	74393	Map10	1	0	1	0	0	2
mmu-miR-223-3p	74409	Hyal6	1	0	0	1	0	2
mmu-miR-223-3p	74412	Gle1	0	1	0	1	0	2
mmu-miR-223-3p	74437	4933402E13Rik	1	0	0	1	0	2
mmu-miR-223-3p	74448	Arl13a	0	1	0	1	0	2
mmu-miR-223-3p	74455	Nsun6	0	1	0	1	0	2
mmu-miR-223-3p	74469	Taf7l	1	0	0	1	0	2
mmu-miR-223-3p	74480	Samd4	1	0	0	1	0	2
mmu-miR-223-3p	74504	Fam53a	1	1	0	0	0	2
mmu-miR-223-3p	74521	Ppp4r4	0	0	1	1	0	2
mmu-miR-223-3p	74533	Gzf1	1	0	0	1	0	2
mmu-miR-223-3p	74549	Mau2	1	0	0	1	0	2
mmu-miR-223-3p	74563	Rasgef1c	0	0	1	1	0	2
mmu-miR-223-3p	74569	Ttc17	0	0	1	1	0	2
mmu-miR-223-3p	74577	Glb1l	1	0	0	1	0	2
mmu-miR-223-3p	74585	Sppl3	0	0	1	1	0	2
mmu-miR-223-3p	74603	Cd200r3	1	0	0	1	0	2
mmu-miR-223-3p	74670	Zfp943	1	0	0	1	0	2
mmu-miR-223-3p	74673	Spdyb	0	0	1	1	0	2
mmu-miR-223-3p	74718	Snx16	0	0	1	1	0	2
mmu-miR-223-3p	74737	Pcf11	1	0	0	1	0	2
mmu-miR-223-3p	74748	Slamf8	0	0	1	1	0	2
mmu-miR-223-3p	74754	Dhcr24	0	0	1	1	0	2
mmu-miR-223-3p	74764	Klc4	0	0	1	1	0	2
mmu-miR-223-3p	74769	Pik3cb	0	0	1	1	0	2
mmu-miR-223-3p	74776	Ppa2	0	0	1	1	0	2
mmu-miR-223-3p	74777	Sepn1	1	0	0	1	0	2
mmu-miR-223-3p	74838	Naa15	1	0	0	1	0	2
mmu-miR-223-3p	74855	Fam228a	0	0	1	1	0	2
mmu-miR-223-3p	74868	Tmem65	1	0	0	1	0	2
mmu-miR-223-3p	74919	Slc35f6	1	0	0	1	0	2
mmu-miR-223-3p	75015	4930503B20Rik	1	0	0	1	0	2
mmu-miR-223-3p	75033	Mei4	0	0	1	1	0	2
mmu-miR-223-3p	75079	Zbtb49	0	1	0	1	0	2
mmu-miR-223-3p	75094	4930522N08Rik	1	0	0	1	0	2
mmu-miR-223-3p	75099	Lysmd4	1	0	0	1	0	2
mmu-miR-223-3p	75124	Nxnl2	0	0	1	1	0	2
mmu-miR-223-3p	75137	Rprd2	1	0	0	1	0	2
mmu-miR-223-3p	75185	Samt4	0	0	1	1	0	2
mmu-miR-223-3p	75209	Sv2c	1	0	0	1	0	2
mmu-miR-223-3p	75212	Rnf121	0	1	0	1	0	2
mmu-miR-223-3p	75216	Cep128	1	0	0	1	0	2
mmu-miR-223-3p	75234	Rnf19b	1	0	0	1	0	2
mmu-miR-223-3p	75276	Ppp1r1c	0	0	1	1	0	2
mmu-miR-223-3p	75288	Slc35f4	0	0	1	1	0	2
mmu-miR-223-3p	75292	Prkd3	1	0	0	1	0	2
mmu-miR-223-3p	75329	Atf7ip2	1	0	0	1	0	2

mmu-miR-223-3p	75368	4930558K02Rik	0	1	0	1	0	2
mmu-miR-223-3p	75425	Tti1	1	0	0	1	0	2
mmu-miR-223-3p	75495	Morn5	0	0	1	1	0	2
mmu-miR-223-3p	75497	Fabp12	1	0	0	1	0	2
mmu-miR-223-3p	75541	1700019G17Rik	0	0	1	1	0	2
mmu-miR-223-3p	75553	Zc3h14	1	0	0	1	0	2
mmu-miR-223-3p	75580	Zbtb4	1	0	0	1	0	2
mmu-miR-223-3p	75608	Chmp4b	0	0	1	1	0	2
mmu-miR-223-3p	75619	Fastkd2	1	0	0	1	0	2
mmu-miR-223-3p	75669	Pik3r4	0	0	1	1	0	2
mmu-miR-223-3p	75690	Vsig10l	1	0	0	1	0	2
mmu-miR-223-3p	75706	Krt24	0	0	1	1	0	2
mmu-miR-223-3p	75710	Rbm12	0	1	0	1	0	2
mmu-miR-223-3p	75735	Pank1	1	0	0	1	0	2
mmu-miR-223-3p	75739	Mpp7	0	1	0	1	0	2
mmu-miR-223-3p	75744	Svip	1	0	0	1	0	2
mmu-miR-223-3p	75761	Apol7a	0	0	1	1	0	2
mmu-miR-223-3p	75763	Dcaf17	1	0	0	1	0	2
mmu-miR-223-3p	75764	Slx1b	0	0	1	1	0	2
mmu-miR-223-3p	75769	4833424O15Rik	0	0	1	1	0	2
mmu-miR-223-3p	75770	Brsk2	0	1	0	1	0	2
mmu-miR-223-3p	75786	Ckap5	1	0	1	0	0	2
mmu-miR-223-3p	75796	Cdyl2	1	0	0	1	0	2
mmu-miR-223-3p	75805	Nln	0	0	1	1	0	2
mmu-miR-223-3p	75812	Tasp1	1	0	0	1	0	2
mmu-miR-223-3p	75827	4930542N06Rik	1	0	0	1	0	2
mmu-miR-223-3p	75829	Prame	1	0	0	1	0	2
mmu-miR-223-3p	75841	Rnf139	0	1	0	1	0	2
mmu-miR-223-3p	75869	Arl5b	0	0	1	1	0	2
mmu-miR-223-3p	75985	Rab30	0	0	1	1	0	2
mmu-miR-223-3p	76007	Zmym2	1	0	0	1	0	2
mmu-miR-223-3p	76051	Ganc	0	1	1	0	0	2
mmu-miR-223-3p	76089	Rapgef2	1	0	0	1	0	2
mmu-miR-223-3p	76137	Mcur1	1	0	0	1	0	2
mmu-miR-223-3p	76178	Coa5	0	0	1	1	0	2
mmu-miR-223-3p	76199	Med13l	0	0	1	1	0	2
mmu-miR-223-3p	76206	Gpr165	1	0	0	1	0	2
mmu-miR-223-3p	76219	Arxes1	1	0	0	1	0	2
mmu-miR-223-3p	76233	Dnttip1	0	1	0	1	0	2
mmu-miR-223-3p	76252	Atp6v0e2	0	1	0	1	0	2
mmu-miR-223-3p	76299	Erp44	1	0	0	1	0	2
mmu-miR-223-3p	76302	Pcnp	1	0	0	1	0	2
mmu-miR-223-3p	76303	Osbp	0	0	1	1	0	2
mmu-miR-223-3p	76365	Tbx18	1	0	0	1	0	2
mmu-miR-223-3p	76366	Mtif3	1	0	0	1	0	2
mmu-miR-223-3p	76400	Pbp2	1	0	0	1	0	2
mmu-miR-223-3p	76415	Fam187b	1	0	0	1	0	2
mmu-miR-223-3p	76441	Daam2	0	0	1	1	0	2
mmu-miR-223-3p	76478	Haus8	1	0	0	1	0	2
mmu-miR-223-3p	76479	Smndc1	0	0	1	1	0	2
mmu-miR-223-3p	76484	Kndc1	0	0	1	1	0	2
mmu-miR-223-3p	76524	Cln6	0	0	1	1	0	2
mmu-miR-223-3p	76527	Il34	0	0	1	1	0	2
mmu-miR-223-3p	76566	Fam101b	1	0	0	1	0	2
mmu-miR-223-3p	76574	Mfsd2a	0	0	1	1	0	2

mmu-miR-223-3p	76606	1700034O15Rik	0	1	1	0	0	2
mmu-miR-223-3p	76614	Immt	1	0	0	1	0	2
mmu-miR-223-3p	76629	Wbscr28	0	0	1	1	0	2
mmu-miR-223-3p	76681	Trim12a	1	0	0	1	0	2
mmu-miR-223-3p	76707	Clasp1	0	1	0	1	0	2
mmu-miR-223-3p	76718	Catsperg2	0	1	0	1	0	2
mmu-miR-223-3p	76773	Wdyhv1	0	0	1	1	0	2
mmu-miR-223-3p	76789	Mzt1	1	0	1	0	0	2
mmu-miR-223-3p	76804	Kdm4c	1	0	0	1	0	2
mmu-miR-223-3p	76816	Sdccag8	0	0	1	1	0	2
mmu-miR-223-3p	76820	Fam49a	0	0	1	1	0	2
mmu-miR-223-3p	76829	Dok5	1	0	0	1	0	2
mmu-miR-223-3p	76857	Spopl	0	0	1	1	0	2
mmu-miR-223-3p	76866	Morn1	0	0	1	1	0	2
mmu-miR-223-3p	76898	B3gat1	1	0	0	1	0	2
mmu-miR-223-3p	76900	Ssbp4	1	0	0	1	0	2
mmu-miR-223-3p	76932	Arfip2	0	1	0	1	0	2
mmu-miR-223-3p	76960	Bcas1	0	0	1	1	0	2
mmu-miR-223-3p	76964	2610028H24Rik	0	0	1	1	0	2
mmu-miR-223-3p	76965	Slitrk1	0	1	0	1	0	2
mmu-miR-223-3p	76987	Hdhd2	0	0	1	1	0	2
mmu-miR-223-3p	77011	Ticrr	0	0	1	1	0	2
mmu-miR-223-3p	77031	Slc9a8	1	0	0	1	0	2
mmu-miR-223-3p	77055	Krt76	1	0	0	1	0	2
mmu-miR-223-3p	77106	Tmem181a	1	0	0	1	0	2
mmu-miR-223-3p	77113	Klhl2	0	0	1	1	0	2
mmu-miR-223-3p	77116	Mtmr2	0	1	0	1	0	2
mmu-miR-223-3p	77166	8030423J24Rik	1	0	0	1	0	2
mmu-miR-223-3p	77219	Ptgr2	0	0	1	1	0	2
mmu-miR-223-3p	77252	9430038I01Rik	1	0	0	1	0	2
mmu-miR-223-3p	77264	Zfp142	0	0	1	1	0	2
mmu-miR-223-3p	77371	Sec24a	0	1	0	1	0	2
mmu-miR-223-3p	77397	9530003J23Rik	1	0	1	0	0	2
mmu-miR-223-3p	77569	Limch1	1	0	0	1	0	2
mmu-miR-223-3p	77582	Mboat7	0	1	0	1	0	2
mmu-miR-223-3p	77604	Rbm12b2	0	0	1	1	0	2
mmu-miR-223-3p	77632	Pramef12	0	0	1	1	0	2
mmu-miR-223-3p	77652	Zfp955a	1	0	0	1	0	2
mmu-miR-223-3p	77669	Arhgef38	0	1	1	0	0	2
mmu-miR-223-3p	77674	Defb12	1	0	0	1	0	2
mmu-miR-223-3p	77771	Csrnp3	1	0	0	1	0	2
mmu-miR-223-3p	77877	6030458C11Rik	0	0	1	1	0	2
mmu-miR-223-3p	77938	Fam53b	1	0	0	1	0	2
mmu-miR-223-3p	77980	Sbf1	0	1	0	1	0	2
mmu-miR-223-3p	77996	Cutal	0	0	1	1	0	2
mmu-miR-223-3p	78246	Phf23	1	0	0	1	0	2
mmu-miR-223-3p	78252	Nxpe2	1	0	0	1	0	2
mmu-miR-223-3p	78256	4921539H07Rik	1	0	0	1	0	2
mmu-miR-223-3p	78267	Klhdc8b	0	0	1	1	0	2
mmu-miR-223-3p	78287	Zfyve20	0	0	1	1	0	2
mmu-miR-223-3p	78321	Ankrd23	0	0	1	1	0	2
mmu-miR-223-3p	78473	Skap1	1	0	0	1	0	2
mmu-miR-223-3p	78541	Asb8	0	0	1	1	0	2
mmu-miR-223-3p	78558	Htra3	1	0	0	1	0	2
mmu-miR-223-3p	78560	Gpr124	1	0	0	1	0	2

mmu-miR-223-3p	78651	Lsm6	0	0	1	1	0	2
mmu-miR-223-3p	78709	Spink8	0	0	1	1	0	2
mmu-miR-223-3p	78748	Rassf10	1	0	0	1	0	2
mmu-miR-223-3p	78751	Zc3h6	0	0	1	1	0	2
mmu-miR-223-3p	78753	Lipm	1	0	0	1	0	2
mmu-miR-223-3p	78774	4930529M08Rik	0	0	1	1	0	2
mmu-miR-223-3p	78777	Enthd2	0	0	1	1	0	2
mmu-miR-223-3p	78783	Brpf1	1	0	0	1	0	2
mmu-miR-223-3p	78789	Vsig1	0	0	1	1	0	2
mmu-miR-223-3p	78798	Eml4	1	0	0	1	0	2
mmu-miR-223-3p	78802	Ttc30a1	0	0	1	1	0	2
mmu-miR-223-3p	78806	Stpg1	0	0	1	1	0	2
mmu-miR-223-3p	78830	Slc25a12	0	1	0	1	0	2
mmu-miR-223-3p	78885	Coro7	0	1	0	1	0	2
mmu-miR-223-3p	78889	Wsb1	1	0	0	1	0	2
mmu-miR-223-3p	78913	Ltn1	1	0	0	1	0	2
mmu-miR-223-3p	78920	Dlst	0	1	0	1	0	2
mmu-miR-223-3p	78925	Srd5a1	1	0	0	1	0	2
mmu-miR-223-3p	78929	Polr3h	0	0	1	1	0	2
mmu-miR-223-3p	79201	Tnfrsf23	1	0	0	1	0	2
mmu-miR-223-3p	79221	Hdac9	0	0	1	1	0	2
mmu-miR-223-3p	79233	Zfp319	1	0	1	0	0	2
mmu-miR-223-3p	79362	Bhlhe41	1	0	0	1	0	2
mmu-miR-223-3p	79455	Pdcl2	0	0	1	1	0	2
mmu-miR-223-3p	79456	Recql4	1	0	0	1	0	2
mmu-miR-223-3p	79555	BC005537	0	1	0	1	0	2
mmu-miR-223-3p	79565	Wbscr27	0	0	1	1	0	2
mmu-miR-223-3p	79566	Sh3bp5l	0	0	1	1	0	2
mmu-miR-223-3p	80281	Cttnbp2nl	1	0	0	1	0	2
mmu-miR-223-3p	80286	Tusc3	0	1	0	1	0	2
mmu-miR-223-3p	80287	Apobec3	1	0	0	1	0	2
mmu-miR-223-3p	80290	Gpr146	1	0	0	1	0	2
mmu-miR-223-3p	80334	Kcnip4	0	0	1	1	0	2
mmu-miR-223-3p	80385	Tusc2	0	0	1	1	0	2
mmu-miR-223-3p	80707	Wwox	0	1	0	1	0	2
mmu-miR-223-3p	80732	Mynn	1	0	0	1	0	2
mmu-miR-223-3p	80748	BC004004	0	0	1	1	0	2
mmu-miR-223-3p	80860	Ghdc	0	0	1	1	0	2
mmu-miR-223-3p	80877	Lrba	0	0	1	1	0	2
mmu-miR-223-3p	80879	Slc16a3	1	0	0	1	0	2
mmu-miR-223-3p	80890	Trim2	1	0	0	1	0	2
mmu-miR-223-3p	80908	Abo	0	0	1	1	0	2
mmu-miR-223-3p	80976	Syt13	0	0	1	1	0	2
mmu-miR-223-3p	81015	Vmn1r56	0	1	0	1	0	2
mmu-miR-223-3p	81703	Jdp2	1	0	0	1	0	2
mmu-miR-223-3p	81799	C1qtnf3	0	0	1	1	0	2
mmu-miR-223-3p	81840	Sorcs2	1	0	0	1	0	2
mmu-miR-223-3p	83379	Klb	0	1	0	1	0	2
mmu-miR-223-3p	83410	Cstf2t	0	0	1	1	0	2
mmu-miR-223-3p	83428	Ucn3	1	0	0	1	0	2
mmu-miR-223-3p	83491	Pramel1	1	0	0	1	0	2
mmu-miR-223-3p	83555	Tex13	0	0	1	1	0	2
mmu-miR-223-3p	83603	Elovl4	1	0	1	0	0	2
mmu-miR-223-3p	83671	Sytl2	1	0	0	1	0	2
mmu-miR-223-3p	83679	Pde4dip	0	0	1	1	0	2

mmu-miR-223-3p	83703	Dbr1	0	1	0	1	0	2
mmu-miR-223-3p	83704	Slc12a9	0	0	1	1	0	2
mmu-miR-223-3p	83796	Smarcd2	0	0	1	1	0	2
mmu-miR-223-3p	83813	Tnk1	0	0	1	1	0	2
mmu-miR-223-3p	83921	Tmem2	1	0	0	1	0	2
mmu-miR-223-3p	83945	Dnaja3	0	1	0	1	0	2
mmu-miR-223-3p	93672	Il24	0	0	1	1	0	2
mmu-miR-223-3p	93675	Clec2i	0	0	1	1	0	2
mmu-miR-223-3p	93685	Entpd7	0	0	1	1	0	2
mmu-miR-223-3p	93694	Clec2d	0	0	1	1	0	2
mmu-miR-223-3p	93695	Gpnmb	0	1	0	1	0	2
mmu-miR-223-3p	93699	Pcdhgb1	1	0	0	1	0	2
mmu-miR-223-3p	93700	Pcdhgb2	1	0	0	1	0	2
mmu-miR-223-3p	93701	Pcdhgb4	1	0	0	1	0	2
mmu-miR-223-3p	93702	Pcdhgb5	1	0	0	1	0	2
mmu-miR-223-3p	93704	Pcdhgb7	1	0	0	1	0	2
mmu-miR-223-3p	93705	Pcdhgb8	1	0	0	1	0	2
mmu-miR-223-3p	93709	Pcdhga1	1	0	0	1	0	2
mmu-miR-223-3p	93710	Pcdhga2	1	0	0	1	0	2
mmu-miR-223-3p	93712	Pcdhga4	1	0	0	1	0	2
mmu-miR-223-3p	93713	Pcdhga5	1	0	0	1	0	2
mmu-miR-223-3p	93714	Pcdhga6	1	0	0	1	0	2
mmu-miR-223-3p	93715	Pcdhga7	1	0	0	1	0	2
mmu-miR-223-3p	93722	Pcdhga10	1	0	0	1	0	2
mmu-miR-223-3p	93730	Lztfl1	1	0	0	1	0	2
mmu-miR-223-3p	93734	Mpv17l	0	0	1	1	0	2
mmu-miR-223-3p	93739	Gabarapl2	0	0	1	1	0	2
mmu-miR-223-3p	93742	Pard3	1	0	0	1	0	2
mmu-miR-223-3p	93837	Dach2	1	0	0	1	0	2
mmu-miR-223-3p	93892	Pcdhb21	0	1	0	1	0	2
mmu-miR-223-3p	93898	Cers1	0	0	1	1	0	2
mmu-miR-223-3p	93961	B3galt5	1	0	0	1	0	2
mmu-miR-223-3p	94040	Clmn	1	0	0	1	0	2
mmu-miR-223-3p	94064	Mrpl27	0	0	1	1	0	2
mmu-miR-223-3p	94067	Mrpl43	1	0	0	1	0	2
mmu-miR-223-3p	94071	Clec2h	0	0	1	1	0	2
mmu-miR-223-3p	94091	Trim11	0	1	1	0	0	2
mmu-miR-223-3p	94092	Trim16	0	0	1	1	0	2
mmu-miR-223-3p	94093	Trim33	1	0	0	1	0	2
mmu-miR-223-3p	94112	Med15	1	0	0	1	0	2
mmu-miR-223-3p	94176	Dock2	0	0	1	1	0	2
mmu-miR-223-3p	94184	Pdxdc1	0	0	1	1	0	2
mmu-miR-223-3p	94185	Tnfrsf21	0	0	1	1	0	2
mmu-miR-223-3p	94219	Cnnm2	1	0	0	1	0	2
mmu-miR-223-3p	94220	Cnnm4	0	0	1	1	0	2
mmu-miR-223-3p	94226	S1pr5	0	0	1	1	0	2
mmu-miR-223-3p	94227	Pi15	1	0	0	1	0	2
mmu-miR-223-3p	94229	Slc4a10	0	0	1	1	0	2
mmu-miR-223-3p	94232	Ubqln4	0	1	0	1	0	2
mmu-miR-223-3p	94249	Slc24a3	0	0	1	1	0	2
mmu-miR-223-3p	94280	Sfxn3	0	0	1	1	0	2
mmu-miR-223-3p	94346	Tmem40	0	0	1	1	0	2
mmu-miR-223-3p	96957	Tmem62	0	1	0	1	0	2
mmu-miR-223-3p	97130	C77080	0	0	1	1	0	2
mmu-miR-223-3p	97212	Hadha	0	0	1	1	0	2

mmu-miR-223-3p	97287	Mtmr14	0	1	1	0	0	2
mmu-miR-223-3p	97761	Sgsm2	1	0	0	1	0	2
mmu-miR-223-3p	97884	B3galnt2	0	1	0	1	0	2
mmu-miR-223-3p	97895	Nlrp4f	0	1	1	0	0	2
mmu-miR-223-3p	97998	Deptor	0	1	0	1	0	2
mmu-miR-223-3p	98193	Dcaf8	0	1	0	1	0	2
mmu-miR-223-3p	98258	Txndc9	0	0	1	1	0	2
mmu-miR-223-3p	98267	Stk17b	0	1	1	0	0	2
mmu-miR-223-3p	98303	D630023F18Rik	0	0	1	1	0	2
mmu-miR-223-3p	98388	Chst10	0	0	1	1	0	2
mmu-miR-223-3p	98496	Pid1	0	0	1	1	0	2
mmu-miR-223-3p	98582	Khdc1b	0	0	1	1	0	2
mmu-miR-223-3p	98845	Eps8l2	0	0	1	1	0	2
mmu-miR-223-3p	98870	AI182371	1	0	0	1	0	2
mmu-miR-223-3p	98952	Fam102a	0	0	1	1	0	2
mmu-miR-223-3p	99035	Olah	1	0	1	0	0	2
mmu-miR-223-3p	99045	Mrps26	1	0	0	1	0	2
mmu-miR-223-3p	99169	AU015228	1	0	0	1	0	2
mmu-miR-223-3p	99311	Commd7	0	0	1	1	0	2
mmu-miR-223-3p	99334	Zscan29	1	0	0	1	0	2
mmu-miR-223-3p	99382	Abtb2	0	0	1	1	0	2
mmu-miR-223-3p	99439	Duox1	0	0	1	1	0	2
mmu-miR-223-3p	99526	Usp53	0	0	1	1	0	2
mmu-miR-223-3p	99586	Dpyd	0	0	1	1	0	2
mmu-miR-223-3p	99683	Sec24b	0	1	0	1	0	2
mmu-miR-223-3p	99696	Ankrd50	1	0	0	1	0	2
mmu-miR-223-3p	99899	Ifi44	0	0	1	1	0	2
mmu-miR-223-3p	100017	Ldlrap1	1	0	0	1	0	2
mmu-miR-223-3p	100087	Kti12	1	0	1	0	0	2
mmu-miR-223-3p	100088	Rcc1	0	0	1	1	0	2
mmu-miR-223-3p	100102	Pcsk9	0	0	1	1	0	2
mmu-miR-223-3p	100163	Pafah2	0	0	1	1	0	2
mmu-miR-223-3p	100182	Akna	0	0	1	1	0	2
mmu-miR-223-3p	100213	Rusc2	0	1	0	1	0	2
mmu-miR-223-3p	100226	Stx12	0	0	1	1	0	2
mmu-miR-223-3p	100340	Smpdl3b	0	0	1	1	0	2
mmu-miR-223-3p	100494	Zfand2a	1	0	0	1	0	2
mmu-miR-223-3p	100702	Gbp6	0	0	1	1	0	2
mmu-miR-223-3p	100756	Usp30	0	1	1	0	0	2
mmu-miR-223-3p	100986	Akap9	1	0	0	1	0	2
mmu-miR-223-3p	101095	Zfp282	0	0	1	1	0	2
mmu-miR-223-3p	101113	Snx21	0	1	0	1	0	2
mmu-miR-223-3p	101142	Itfg2	0	0	1	1	0	2
mmu-miR-223-3p	101206	Tada3	1	0	0	1	0	2
mmu-miR-223-3p	101351	Eogt	0	0	1	1	0	2
mmu-miR-223-3p	101476	Plekha1	0	0	1	1	0	2
mmu-miR-223-3p	101489	Ric8	1	0	1	0	0	2
mmu-miR-223-3p	101502	Hsd3b7	1	0	0	1	0	2
mmu-miR-223-3p	101540	Prkd2	0	0	1	1	0	2
mmu-miR-223-3p	101706	Numa1	0	0	1	1	0	2
mmu-miR-223-3p	101772	Ano1	1	0	0	1	0	2
mmu-miR-223-3p	101809	Spred3	1	0	0	1	0	2
mmu-miR-223-3p	102122	Fam192a	0	0	1	1	0	2
mmu-miR-223-3p	102141	Snx25	1	0	0	1	0	2
mmu-miR-223-3p	102247	Agpat6	0	0	1	1	0	2

mmu-miR-223-3p	102334	Ankrd10	1	0	0	1	0	2
mmu-miR-223-3p	102436	Lars2	0	0	1	1	0	2
mmu-miR-223-3p	102448	Xylb	0	1	0	1	0	2
mmu-miR-223-3p	102607	Snx19	0	0	1	1	0	2
mmu-miR-223-3p	102693	Phldb1	1	0	0	1	0	2
mmu-miR-223-3p	102747	Lrrc49	0	1	0	1	0	2
mmu-miR-223-3p	102866	Pls3	0	0	1	1	0	2
mmu-miR-223-3p	103135	Pan2	0	0	1	1	0	2
mmu-miR-223-3p	103136	Pwp1	1	0	0	1	0	2
mmu-miR-223-3p	103140	Gstt3	1	0	0	1	0	2
mmu-miR-223-3p	103161	Apof	0	1	0	1	0	2
mmu-miR-223-3p	103236	Csnk1g2	0	0	1	1	0	2
mmu-miR-223-3p	103268	Cep57l1	1	0	0	1	0	2
mmu-miR-223-3p	103284	Zc3h10	1	0	0	1	0	2
mmu-miR-223-3p	103466	Nt5dc3	1	0	0	1	0	2
mmu-miR-223-3p	103737	Pex12	0	0	1	1	0	2
mmu-miR-223-3p	103963	Rpn1	1	0	0	1	0	2
mmu-miR-223-3p	104009	Qsox1	1	0	0	1	0	2
mmu-miR-223-3p	104010	Cdh22	0	0	1	1	0	2
mmu-miR-223-3p	104015	Synj1	1	0	0	1	0	2
mmu-miR-223-3p	104027	Synpo	0	0	1	1	0	2
mmu-miR-223-3p	104082	Wdr7	1	0	0	1	0	2
mmu-miR-223-3p	104099	Itga9	1	0	0	1	0	2
mmu-miR-223-3p	104174	Gldc	1	0	0	1	0	2
mmu-miR-223-3p	104184	Blmh	0	0	1	1	0	2
mmu-miR-223-3p	104248	Cabin1	0	0	1	1	0	2
mmu-miR-223-3p	104318	Csnk1d	1	0	0	1	0	2
mmu-miR-223-3p	104348	Zfp120	1	0	0	1	0	2
mmu-miR-223-3p	104360	Isl2	0	1	0	1	0	2
mmu-miR-223-3p	104732	4930427A07Rik	0	0	1	1	0	2
mmu-miR-223-3p	104759	Pld4	1	0	1	0	0	2
mmu-miR-223-3p	104799	Vipas39	0	0	1	1	0	2
mmu-miR-223-3p	104816	Aspg	0	0	1	1	0	2
mmu-miR-223-3p	104836	Cbll1	0	0	1	1	0	2
mmu-miR-223-3p	104943	Fam110c	0	0	1	1	0	2
mmu-miR-223-3p	105298	Epdr1	0	0	1	1	0	2
mmu-miR-223-3p	105372	Utp15	0	0	1	1	0	2
mmu-miR-223-3p	105418	E330034G19Rik	0	0	1	1	0	2
mmu-miR-223-3p	105428	Fam149b	0	0	1	1	0	2
mmu-miR-223-3p	105445	Dock9	0	0	1	1	0	2
mmu-miR-223-3p	105504	Exoc5	1	0	0	1	0	2
mmu-miR-223-3p	105513	Chmp7	0	0	1	1	0	2
mmu-miR-223-3p	105559	Mbnl2	1	0	0	1	0	2
mmu-miR-223-3p	105594	Cphx1	1	0	0	1	0	2
mmu-miR-223-3p	105653	Phyhip	0	1	0	1	0	2
mmu-miR-223-3p	105670	Rcbtb2	1	0	0	1	0	2
mmu-miR-223-3p	105689	Mycbp2	0	0	1	1	0	2
mmu-miR-223-3p	105827	Amigo2	1	0	0	1	0	2
mmu-miR-223-3p	105841	Dennd3	0	0	1	1	0	2
mmu-miR-223-3p	105847	Lmf2	0	0	1	1	0	2
mmu-miR-223-3p	106073	Mfsd5	0	0	1	1	0	2
mmu-miR-223-3p	106205	Zc3h7a	0	0	1	1	0	2
mmu-miR-223-3p	106298	Rrn3	1	0	0	1	0	2
mmu-miR-223-3p	106326	Osbpl11	1	0	0	1	0	2
mmu-miR-223-3p	106347	Ildr1	1	0	0	1	0	2

mmu-miR-223-3p	106393	Srl	0	0	1	1	0	2
mmu-miR-223-3p	106504	Stk38	0	0	1	1	0	2
mmu-miR-223-3p	106522	Pkdcc	0	0	1	1	0	2
mmu-miR-223-3p	106722	AU023871	0	0	1	1	0	2
mmu-miR-223-3p	106763	Ttbk1	0	1	0	1	0	2
mmu-miR-223-3p	106794	Dhx57	1	0	0	1	0	2
mmu-miR-223-3p	106795	Tcf19	0	0	1	1	0	2
mmu-miR-223-3p	106861	Abhd3	1	0	1	0	0	2
mmu-miR-223-3p	106931	Kctd1	1	0	0	1	0	2
mmu-miR-223-3p	107029	Me2	1	0	0	1	0	2
mmu-miR-223-3p	107250	Kazald1	0	0	1	1	0	2
mmu-miR-223-3p	107260	Otub1	0	0	1	1	0	2
mmu-miR-223-3p	107272	Psat1	1	0	0	1	0	2
mmu-miR-223-3p	107338	Gbf1	1	0	0	1	0	2
mmu-miR-223-3p	107351	Kank1	0	0	1	1	0	2
mmu-miR-223-3p	107368	Pdzd8	0	0	1	1	0	2
mmu-miR-223-3p	107371	Exoc6	1	0	1	0	0	2
mmu-miR-223-3p	107449	Unc5b	1	0	0	1	0	2
mmu-miR-223-3p	107566	Arl2bp	0	1	0	1	0	2
mmu-miR-223-3p	107656	Krt9	0	1	0	1	0	2
mmu-miR-223-3p	107684	Coro2a	0	0	1	1	0	2
mmu-miR-223-3p	107701	Sf3b4	0	0	1	1	0	2
mmu-miR-223-3p	107733	Mrpl41	0	0	1	1	0	2
mmu-miR-223-3p	107765	Ankrd1	1	0	0	1	0	2
mmu-miR-223-3p	107817	Jmjd6	0	1	0	1	0	2
mmu-miR-223-3p	107823	Whsc1	0	0	1	1	0	2
mmu-miR-223-3p	107889	Gcm2	0	0	1	1	0	2
mmu-miR-223-3p	107932	Chd4	1	0	0	1	0	2
mmu-miR-223-3p	107934	Celsr3	0	0	1	1	0	2
mmu-miR-223-3p	107993	Bfsp2	0	0	1	1	0	2
mmu-miR-223-3p	107999	Gtpbp6	1	0	0	0	1	2
mmu-miR-223-3p	108011	Ap4e1	0	0	1	1	0	2
mmu-miR-223-3p	108012	Ap1s2	0	0	1	1	0	2
mmu-miR-223-3p	108013	Celf4	0	0	1	1	0	2
mmu-miR-223-3p	108030	Lin7a	1	0	0	1	0	2
mmu-miR-223-3p	108043	Chrnb3	1	0	0	1	0	2
mmu-miR-223-3p	108058	Camk2d	0	1	0	1	0	2
mmu-miR-223-3p	108062	Cstf2	1	0	0	1	0	2
mmu-miR-223-3p	108067	Eif2b3	0	0	1	1	0	2
mmu-miR-223-3p	108069	Grm3	0	1	0	1	0	2
mmu-miR-223-3p	108071	Grm5	1	0	0	1	0	2
mmu-miR-223-3p	108073	Grm7	0	0	1	1	0	2
mmu-miR-223-3p	108079	Prkaa2	1	0	0	1	0	2
mmu-miR-223-3p	108089	Rnf144a	1	0	0	1	0	2
mmu-miR-223-3p	108100	Baiap2	0	0	1	1	0	2
mmu-miR-223-3p	108115	Slco4a1	1	0	0	1	0	2
mmu-miR-223-3p	108138	Xrcc4	0	0	1	1	0	2
mmu-miR-223-3p	108151	Sema3d	1	0	0	1	0	2
mmu-miR-223-3p	108159	Ubxn8	0	0	1	1	0	2
mmu-miR-223-3p	108652	Slc35b3	0	0	1	1	0	2
mmu-miR-223-3p	108654	Fam210a	1	0	0	1	0	2
mmu-miR-223-3p	108671	Dnajc9	0	0	1	1	0	2
mmu-miR-223-3p	108672	Zdhhc15	0	1	0	1	0	2
mmu-miR-223-3p	108689	Obfc1	0	0	1	1	0	2
mmu-miR-223-3p	108699	Chn1	0	0	1	1	0	2

mmu-miR-223-3p	108797	Mex3b	0	1	0	1	0	2
mmu-miR-223-3p	108837	Ibtk	1	0	0	1	0	2
mmu-miR-223-3p	108897	Aif1l	0	0	1	1	0	2
mmu-miR-223-3p	108907	Nusap1	1	0	0	1	0	2
mmu-miR-223-3p	108909	Aida	0	1	0	1	0	2
mmu-miR-223-3p	108954	Ppp1r15b	1	0	0	1	0	2
mmu-miR-223-3p	108960	Irak2	1	0	0	1	0	2
mmu-miR-223-3p	108978	4930555G01Rik	0	0	1	1	0	2
mmu-miR-223-3p	109075	Exosc4	1	0	0	1	0	2
mmu-miR-223-3p	109113	Uhrf2	0	0	1	1	0	2
mmu-miR-223-3p	109205	Sobp	1	0	0	1	0	2
mmu-miR-223-3p	109225	Ms4a7	1	0	0	1	0	2
mmu-miR-223-3p	109229	Fam118b	0	0	1	1	0	2
mmu-miR-223-3p	109242	Kif24	0	1	0	1	0	2
mmu-miR-223-3p	109245	Lrrc39	1	0	0	1	0	2
mmu-miR-223-3p	109294	Prex2	1	0	0	1	0	2
mmu-miR-223-3p	109299	C330006A16Rik	1	0	0	1	0	2
mmu-miR-223-3p	109323	C1qtnf7	1	0	0	1	0	2
mmu-miR-223-3p	109331	Rnf20	0	0	1	1	0	2
mmu-miR-223-3p	109552	Sri	1	0	0	1	0	2
mmu-miR-223-3p	109689	Arrb1	1	0	0	1	0	2
mmu-miR-223-3p	109700	Itga1	0	0	1	1	0	2
mmu-miR-223-3p	109754	Cyb5r3	0	0	1	1	0	2
mmu-miR-223-3p	109801	Glo1	0	1	0	1	0	2
mmu-miR-223-3p	109821	F11	0	0	1	1	0	2
mmu-miR-223-3p	109905	Rap1a	0	0	1	1	0	2
mmu-miR-223-3p	110006	Gusb	0	0	1	1	0	2
mmu-miR-223-3p	110078	Pygb	0	0	1	1	0	2
mmu-miR-223-3p	110197	Dgkg	1	0	0	1	0	2
mmu-miR-223-3p	110310	Krt7	0	1	0	1	0	2
mmu-miR-223-3p	110350	Dync2h1	0	1	0	1	0	2
mmu-miR-223-3p	110355	Adrbk1	0	1	0	1	0	2
mmu-miR-223-3p	110385	Pde4c	0	0	1	1	0	2
mmu-miR-223-3p	110521	Hivep1	0	1	0	1	0	2
mmu-miR-223-3p	110532	Adarb1	0	1	0	1	0	2
mmu-miR-223-3p	110595	Timp4	0	1	1	0	0	2
mmu-miR-223-3p	110596	Arhgef28	0	0	1	1	0	2
mmu-miR-223-3p	110606	Fntb	0	1	0	1	0	2
mmu-miR-223-3p	110784	Nr3c2	0	0	1	1	0	2
mmu-miR-223-3p	110796	Tshz1	0	0	1	1	0	2
mmu-miR-223-3p	110821	Pcca	0	0	1	1	0	2
mmu-miR-223-3p	110854	Ppp2r4	0	0	1	1	0	2
mmu-miR-223-3p	110959	Nudt19	1	0	0	1	0	2
mmu-miR-223-3p	111241	Hmga1-rs1	1	0	0	1	0	2
mmu-miR-223-3p	112405	Egln1	1	0	0	1	0	2
mmu-miR-223-3p	112407	Egln3	0	0	1	1	0	2
mmu-miR-223-3p	112415	C030039L03Rik	1	0	0	1	0	2
mmu-miR-223-3p	112419	2010002M12Rik	1	0	0	1	0	2
mmu-miR-223-3p	114128	Laptm4b	0	0	1	1	0	2
mmu-miR-223-3p	114230	Aipl1	1	0	0	1	0	2
mmu-miR-223-3p	114301	Palmd	0	1	0	1	0	2
mmu-miR-223-3p	114479	Slc5a5	0	0	1	1	0	2
mmu-miR-223-3p	114604	Prdm15	1	0	0	1	0	2
mmu-miR-223-3p	114641	Rpl31	1	0	0	1	0	2
mmu-miR-223-3p	114642	Brdt	1	0	0	1	0	2

mmu-miR-223-3p	114654	Ly6g6d	1	0	0	1	0	2
mmu-miR-223-3p	114671	4930444G20Rik	1	0	0	1	0	2
mmu-miR-223-3p	114674	Gtf2ird2	0	0	1	1	0	2
mmu-miR-223-3p	114863	Prosc	0	0	1	1	0	2
mmu-miR-223-3p	114874	Ddhd1	0	1	0	1	0	2
mmu-miR-223-3p	114886	Cygb	0	0	1	1	0	2
mmu-miR-223-3p	116733	Vps4a	0	0	1	1	0	2
mmu-miR-223-3p	116871	Mta3	0	1	0	1	0	2
mmu-miR-223-3p	116914	Slc19a2	1	0	0	1	0	2
mmu-miR-223-3p	117066	Cts3	0	0	1	1	0	2
mmu-miR-223-3p	117147	Acsm1	0	0	1	1	0	2
mmu-miR-223-3p	117149	Tirap	1	0	0	1	0	2
mmu-miR-223-3p	117158	Scgb3a2	1	0	0	1	0	2
mmu-miR-223-3p	117198	Ivns1abp	1	0	0	1	0	2
mmu-miR-223-3p	117591	Slc2a9	1	0	0	1	0	2
mmu-miR-223-3p	118451	Mrps2	1	0	0	1	0	2
mmu-miR-223-3p	140475	Bsnd	0	0	1	1	0	2
mmu-miR-223-3p	140481	Man2a2	0	1	0	1	0	2
mmu-miR-223-3p	140570	Plxnb2	1	0	0	1	0	2
mmu-miR-223-3p	140629	Ubox5	0	0	1	1	0	2
mmu-miR-223-3p	140721	Caskin2	0	0	1	1	0	2
mmu-miR-223-3p	140723	Cacng5	0	0	1	1	0	2
mmu-miR-223-3p	140740	Sec63	0	0	1	1	0	2
mmu-miR-223-3p	140743	Rem2	0	0	1	1	0	2
mmu-miR-223-3p	140765	Tmprss3	1	0	0	1	0	2
mmu-miR-223-3p	140792	Colec12	0	0	1	1	0	2
mmu-miR-223-3p	140810	Ttbk2	1	0	0	1	0	2
mmu-miR-223-3p	140917	Dclre1b	0	1	0	1	0	2
mmu-miR-223-3p	140919	Slc17a6	1	0	0	1	0	2
mmu-miR-223-3p	170441	Slc2a10	1	0	1	0	0	2
mmu-miR-223-3p	170442	Bbox1	1	1	0	0	0	2
mmu-miR-223-3p	170459	Stard4	1	0	0	1	0	2
mmu-miR-223-3p	170644	Ubn1	0	0	1	1	0	2
mmu-miR-223-3p	170676	Peg10	1	0	0	1	0	2
mmu-miR-223-3p	170707	Usp48	0	0	1	1	0	2
mmu-miR-223-3p	170725	Capn8	0	0	1	1	0	2
mmu-miR-223-3p	170728	Rtn4ip1	0	0	1	1	0	2
mmu-miR-223-3p	170742	Sertad3	1	0	0	1	0	2
mmu-miR-223-3p	170749	Mtmr4	1	0	0	1	0	2
mmu-miR-223-3p	170756	Slc8b1	0	0	1	1	0	2
mmu-miR-223-3p	170765	Ripply3	0	0	1	1	0	2
mmu-miR-223-3p	170767	Rfxap	1	0	0	1	0	2
mmu-miR-223-3p	170938	Zfp617	0	0	1	1	0	2
mmu-miR-223-3p	171180	Syt12	1	0	1	0	0	2
mmu-miR-223-3p	171188	Vmn1r32	0	0	1	1	0	2
mmu-miR-223-3p	171211	Edaradd	1	0	0	1	0	2
mmu-miR-223-3p	171405	Slc22a27	1	0	0	1	0	2
mmu-miR-223-3p	192119	Dicer1	1	0	0	1	0	2
mmu-miR-223-3p	192156	Mvd	0	0	1	1	0	2
mmu-miR-223-3p	192160	Casc3	1	0	0	1	0	2
mmu-miR-223-3p	192174	Rwdd4a	1	0	0	1	0	2
mmu-miR-223-3p	192185	Nadk	1	0	0	1	0	2
mmu-miR-223-3p	192193	Edem1	1	0	0	1	0	2
mmu-miR-223-3p	192195	Ash1l	1	0	0	1	0	2
mmu-miR-223-3p	192212	Prom2	0	0	1	1	0	2

mmu-miR-223-3p	192231	Hexim1	1	0	1	0	0	2
mmu-miR-223-3p	192287	Slc25a36	0	0	1	1	0	2
mmu-miR-223-3p	192292	Nrbp1	0	0	1	1	0	2
mmu-miR-223-3p	192651	Zfp286	0	1	0	1	0	2
mmu-miR-223-3p	192654	Pla2g15	1	0	0	1	0	2
mmu-miR-223-3p	192663	Abcg4	0	0	1	1	0	2
mmu-miR-223-3p	192734	Fam211b	0	0	1	1	0	2
mmu-miR-223-3p	192786	Rapgef6	1	0	0	1	0	2
mmu-miR-223-3p	192950	Nacad	0	1	0	1	0	2
mmu-miR-223-3p	192970	Dhrs11	0	1	0	1	0	2
mmu-miR-223-3p	193116	Slu7	0	1	0	1	0	2
mmu-miR-223-3p	193322	Oog1	1	0	0	1	0	2
mmu-miR-223-3p	194227	Gm13023	0	0	1	1	0	2
mmu-miR-223-3p	194268	9930104L06Rik	1	0	1	0	0	2
mmu-miR-223-3p	194309	Vps37d	1	0	0	1	0	2
mmu-miR-223-3p	194352	Trpv5	0	0	1	1	0	2
mmu-miR-223-3p	194597	Tmprss11a	1	0	1	0	0	2
mmu-miR-223-3p	195046	Nlrp1a	1	0	0	1	0	2
mmu-miR-223-3p	195209	Gm22	1	0	0	1	0	2
mmu-miR-223-3p	195359	Trim40	0	0	1	1	0	2
mmu-miR-223-3p	207165	Bptf	0	1	0	1	0	2
mmu-miR-223-3p	207175	Cetn4	1	0	1	0	0	2
mmu-miR-223-3p	207278	Fchsd2	0	1	0	1	0	2
mmu-miR-223-3p	207596	Thsd4	1	0	0	1	0	2
mmu-miR-223-3p	207683	Igsf11	1	0	0	1	0	2
mmu-miR-223-3p	207704	Gtpbp10	0	0	1	1	0	2
mmu-miR-223-3p	207777	Bzrap1	1	0	0	1	0	2
mmu-miR-223-3p	207818	Smagp	0	1	0	1	0	2
mmu-miR-223-3p	207932	Urb1	1	0	0	1	0	2
mmu-miR-223-3p	208043	Setd1b	1	0	0	1	0	2
mmu-miR-223-3p	208084	Pif1	1	0	0	1	0	2
mmu-miR-223-3p	208098	Panx3	1	1	0	0	0	2
mmu-miR-223-3p	208104	Mlxip	1	0	0	1	0	2
mmu-miR-223-3p	208111	9830147E19Rik	1	0	0	1	0	2
mmu-miR-223-3p	208144	Dhx37	0	1	1	0	0	2
mmu-miR-223-3p	208151	Tmem132b	0	1	0	1	0	2
mmu-miR-223-3p	208154	Btla	1	0	0	1	0	2
mmu-miR-223-3p	208158	Map6d1	1	0	0	1	0	2
mmu-miR-223-3p	208166	Gm609	0	0	1	1	0	2
mmu-miR-223-3p	208228	Mob3a	0	0	1	1	0	2
mmu-miR-223-3p	208258	Ankrd33	1	0	0	1	0	2
mmu-miR-223-3p	208263	Tor1aip1	0	1	0	1	0	2
mmu-miR-223-3p	208583	Nek11	0	1	0	1	0	2
mmu-miR-223-3p	208606	Rsrc2	0	1	0	1	0	2
mmu-miR-223-3p	208691	Eif5a2	1	0	0	1	0	2
mmu-miR-223-3p	208727	Hdac4	1	0	0	1	0	2
mmu-miR-223-3p	208768	Sde2	0	0	1	1	0	2
mmu-miR-223-3p	208777	Sned1	0	1	0	1	0	2
mmu-miR-223-3p	208820	Triqk	1	0	0	1	0	2
mmu-miR-223-3p	208836	Fanci	0	1	0	1	0	2
mmu-miR-223-3p	208890	Slc26a7	0	1	0	1	0	2
mmu-miR-223-3p	208908	Ccdc62	1	0	0	1	0	2
mmu-miR-223-3p	209195	Clic6	1	0	0	1	0	2
mmu-miR-223-3p	209354	Eif2b1	1	0	0	1	0	2
mmu-miR-223-3p	209361	Taf3	1	0	0	1	0	2

mmu-miR-223-3p	209378	Itih5	1	0	0	1	0	2
mmu-miR-223-3p	209416	Gpkow	1	0	0	1	0	2
mmu-miR-223-3p	209446	Tfe3	0	1	0	1	0	2
mmu-miR-223-3p	209462	Hace1	1	0	0	1	0	2
mmu-miR-223-3p	209497	Tmem164	0	0	1	1	0	2
mmu-miR-223-3p	209584	Tyw3	0	0	1	1	0	2
mmu-miR-223-3p	209760	Tmc7	1	0	0	1	0	2
mmu-miR-223-3p	209966	Pgbd5	0	0	1	1	0	2
mmu-miR-223-3p	210027	Slc35f3	1	0	0	1	0	2
mmu-miR-223-3p	210094	Iglon5	0	1	0	1	0	2
mmu-miR-223-3p	210105	Zfp719	1	0	0	1	0	2
mmu-miR-223-3p	210108	D130043K22Rik	0	0	1	1	0	2
mmu-miR-223-3p	210135	Zfp180	1	0	1	0	0	2
mmu-miR-223-3p	210293	Dock10	1	0	0	1	0	2
mmu-miR-223-3p	210417	Thsd7b	0	1	0	1	0	2
mmu-miR-223-3p	210529	Mettl14	1	0	0	1	0	2
mmu-miR-223-3p	210940	4931408C20Rik	0	0	0	1	1	2
mmu-miR-223-3p	211228	Lrrc25	0	0	1	1	0	2
mmu-miR-223-3p	211255	Kbtbd7	0	0	1	1	0	2
mmu-miR-223-3p	211401	Mtss1	1	0	0	1	0	2
mmu-miR-223-3p	211446	Exoc3	1	0	0	1	0	2
mmu-miR-223-3p	211480	Kcnj14	0	0	1	1	0	2
mmu-miR-223-3p	211556	Ap1ar	0	0	1	1	0	2
mmu-miR-223-3p	211577	Mrgprf	0	0	1	1	0	2
mmu-miR-223-3p	211770	Trib1	1	0	0	1	0	2
mmu-miR-223-3p	211914	Asap2	1	0	0	1	0	2
mmu-miR-223-3p	211936	Ccdc73	1	0	0	1	0	2
mmu-miR-223-3p	211961	Asxl3	1	0	0	1	0	2
mmu-miR-223-3p	211986	Tmem18	0	0	1	1	0	2
mmu-miR-223-3p	212073	Syne3	1	0	0	1	0	2
mmu-miR-223-3p	212127	Proser1	1	0	0	1	0	2
mmu-miR-223-3p	212139	Cc2d1a	0	0	1	1	0	2
mmu-miR-223-3p	212153	2610015P09Rik	1	0	0	1	0	2
mmu-miR-223-3p	212167	Gsap	1	0	0	1	0	2
mmu-miR-223-3p	212168	Zswim4	1	0	0	1	0	2
mmu-miR-223-3p	212326	Fam149a	0	0	1	1	0	2
mmu-miR-223-3p	212448	9330159F19Rik	1	0	0	1	0	2
mmu-miR-223-3p	212517	Wdr52	0	1	0	1	0	2
mmu-miR-223-3p	212569	Zfp273	1	0	0	1	0	2
mmu-miR-223-3p	212712	Satb2	0	0	1	1	0	2
mmu-miR-223-3p	212862	Chpt1	1	0	0	1	0	2
mmu-miR-223-3p	212937	Tifab	0	0	1	1	0	2
mmu-miR-223-3p	212980	Slc45a3	1	0	0	1	0	2
mmu-miR-223-3p	212998	BC016579	0	0	1	1	0	2
mmu-miR-223-3p	213053	Slc39a14	0	0	1	1	0	2
mmu-miR-223-3p	213084	Cdkl3	1	0	0	1	0	2
mmu-miR-223-3p	213109	Phf3	1	0	0	1	0	2
mmu-miR-223-3p	213121	Ankrd35	0	1	0	1	0	2
mmu-miR-223-3p	213208	Il20rb	1	0	0	1	0	2
mmu-miR-223-3p	213236	Dnd1	0	0	1	1	0	2
mmu-miR-223-3p	213262	Fstl5	0	0	1	1	0	2
mmu-miR-223-3p	213389	Prdm9	1	0	0	1	0	2
mmu-miR-223-3p	213402	Armc2	0	0	1	1	0	2
mmu-miR-223-3p	213438	A630033H20Rik	1	0	0	1	0	2
mmu-miR-223-3p	213469	Lgi3	1	0	0	1	0	2

mmu-miR-223-3p	213499	Fbxo42	1	0	0	1	0	2
mmu-miR-223-3p	213753	Zfp598	0	0	1	1	0	2
mmu-miR-223-3p	213819	Casd1	1	0	0	1	0	2
mmu-miR-223-3p	213945	Col28a1	0	0	1	1	0	2
mmu-miR-223-3p	213990	Agap3	1	0	0	1	0	2
mmu-miR-223-3p	214084	Slc18a2	0	0	1	1	0	2
mmu-miR-223-3p	214133	Tet2	1	0	0	1	0	2
mmu-miR-223-3p	214191	Ttc24	1	0	0	1	0	2
mmu-miR-223-3p	214239	A430105I19Rik	0	0	1	1	0	2
mmu-miR-223-3p	214290	Zcchc6	0	0	1	1	0	2
mmu-miR-223-3p	214321	Gm4787	1	0	0	1	0	2
mmu-miR-223-3p	214663	Slc25a29	1	0	0	1	0	2
mmu-miR-223-3p	214669	L3mbtl2	0	0	1	1	0	2
mmu-miR-223-3p	214812	Zfp609	1	0	0	1	0	2
mmu-miR-223-3p	214854	Neurl3	1	0	1	0	0	2
mmu-miR-223-3p	214855	Arid5a	0	0	1	1	0	2
mmu-miR-223-3p	214917	Fam173a	1	0	0	1	0	2
mmu-miR-223-3p	215280	Wipf1	0	0	1	1	0	2
mmu-miR-223-3p	215335	Slc36a1	0	0	1	1	0	2
mmu-miR-223-3p	215387	Ncaph	0	1	0	1	0	2
mmu-miR-223-3p	215449	Rap1b	0	0	1	1	0	2
mmu-miR-223-3p	215493	A3galt2	1	0	1	0	0	2
mmu-miR-223-3p	215632	Psd4	0	0	1	1	0	2
mmu-miR-223-3p	215690	Nav1	1	0	0	1	0	2
mmu-miR-223-3p	215772	Adgb	0	1	0	1	0	2
mmu-miR-223-3p	215819	Nhsl1	0	0	1	1	0	2
mmu-miR-223-3p	215890	Clvs2	1	0	0	1	0	2
mmu-miR-223-3p	215999	Mcu	1	0	0	1	0	2
mmu-miR-223-3p	216001	Micu1	0	1	0	1	0	2
mmu-miR-223-3p	216021	Stox1	0	1	0	1	0	2
mmu-miR-223-3p	216028	Lrrtm3	1	0	0	1	0	2
mmu-miR-223-3p	216080	Ube2d1	1	0	1	0	0	2
mmu-miR-223-3p	216148	Shc2	0	0	1	1	0	2
mmu-miR-223-3p	216227	Slc17a8	1	0	0	1	0	2
mmu-miR-223-3p	216274	Cep290	1	0	1	0	0	2
mmu-miR-223-3p	216285	Alx1	1	0	0	1	0	2
mmu-miR-223-3p	216343	Tph2	1	0	0	1	0	2
mmu-miR-223-3p	216363	Rab3ip	0	0	1	1	0	2
mmu-miR-223-3p	216393	D930020B18Rik	0	0	1	1	0	2
mmu-miR-223-3p	216439	Agap2	1	0	0	1	0	2
mmu-miR-223-3p	216440	Os9	1	0	0	1	0	2
mmu-miR-223-3p	216456	Gls2	0	1	0	1	0	2
mmu-miR-223-3p	216505	Pik3ip1	0	1	0	1	0	2
mmu-miR-223-3p	216549	Aftph	0	1	0	1	0	2
mmu-miR-223-3p	216551	Lgalsl	0	0	1	1	0	2
mmu-miR-223-3p	216613	Ccdc85a	1	0	0	1	0	2
mmu-miR-223-3p	216622	4931440F15Rik	0	0	1	1	0	2
mmu-miR-223-3p	216705	Clint1	0	0	1	1	0	2
mmu-miR-223-3p	216742	Fnip1	1	0	0	1	0	2
mmu-miR-223-3p	216795	Wnt9a	0	0	1	1	0	2
mmu-miR-223-3p	216805	Flcn	1	0	0	1	0	2
mmu-miR-223-3p	216831	Arhgap44	1	0	0	1	0	2
mmu-miR-223-3p	216848	Chd3	0	1	0	1	0	2
mmu-miR-223-3p	216860	Neurl4	0	0	1	1	0	2
mmu-miR-223-3p	216864	Mgl2	0	0	1	1	0	2

mmu-miR-223-3p	216874	Camta2	0	1	0	1	0	2
mmu-miR-223-3p	216881	Wscd1	0	1	1	0	0	2
mmu-miR-223-3p	216987	Utp6	0	0	1	1	0	2
mmu-miR-223-3p	217057	Ptrh2	1	0	0	1	0	2
mmu-miR-223-3p	217122	Gm11545	0	0	1	1	0	2
mmu-miR-223-3p	217125	Samd14	1	0	0	1	0	2
mmu-miR-223-3p	217127	Kat7	0	0	1	1	0	2
mmu-miR-223-3p	217169	Tns4	1	0	0	1	0	2
mmu-miR-223-3p	217201	Rundc1	0	0	1	1	0	2
mmu-miR-223-3p	217203	Tmem106a	0	0	1	1	0	2
mmu-miR-223-3p	217207	Dhx8	0	0	1	1	0	2
mmu-miR-223-3p	217218	Atxn7l3	1	0	0	1	0	2
mmu-miR-223-3p	217262	Abca9	0	0	1	1	0	2
mmu-miR-223-3p	217303	Cd300a	0	0	1	1	0	2
mmu-miR-223-3p	217304	Cd300lb	1	0	0	1	0	2
mmu-miR-223-3p	217316	Slc16a5	0	0	1	1	0	2
mmu-miR-223-3p	217340	Rnf157	1	0	0	1	0	2
mmu-miR-223-3p	217364	Engase	0	1	0	1	0	2
mmu-miR-223-3p	217378	Dnajc27	0	0	1	1	0	2
mmu-miR-223-3p	217410	Trib2	0	0	1	1	0	2
mmu-miR-223-3p	217449	Trappc12	1	0	0	1	0	2
mmu-miR-223-3p	217473	Ankmy2	0	1	1	0	0	2
mmu-miR-223-3p	217558	G2e3	1	0	0	1	0	2
mmu-miR-223-3p	217593	Slc25a21	0	0	1	1	0	2
mmu-miR-223-3p	217615	Ctage5	0	1	0	1	0	2
mmu-miR-223-3p	217653	Mis18bp1	1	0	0	1	0	2
mmu-miR-223-3p	217664	Mgat2	1	0	0	1	0	2
mmu-miR-223-3p	217666	L2hgdh	0	0	1	1	0	2
mmu-miR-223-3p	217682	Plekhd1	1	0	0	1	0	2
mmu-miR-223-3p	217684	4933426M11Rik	0	0	1	1	0	2
mmu-miR-223-3p	217692	Sipa1l1	0	1	0	1	0	2
mmu-miR-223-3p	217708	Lin52	0	0	1	1	0	2
mmu-miR-223-3p	217721	Mfsd7c	1	0	1	0	0	2
mmu-miR-223-3p	217732	2310044G17Rik	0	0	1	1	0	2
mmu-miR-223-3p	217837	Itpk1	0	0	1	1	0	2
mmu-miR-223-3p	217866	Cdc42bpb	0	0	1	1	0	2
mmu-miR-223-3p	217882	Cep170b	1	0	0	1	0	2
mmu-miR-223-3p	217946	Cdca7l	0	0	1	1	0	2
mmu-miR-223-3p	218030	Pou6f2	0	1	0	1	0	2
mmu-miR-223-3p	218038	Amph	1	0	0	1	0	2
mmu-miR-223-3p	218100	Zfp322a	1	0	0	1	0	2
mmu-miR-223-3p	218210	Nup153	0	0	1	1	0	2
mmu-miR-223-3p	218232	Ptpdc1	0	1	1	0	0	2
mmu-miR-223-3p	218271	B4galt7	0	0	1	1	0	2
mmu-miR-223-3p	218314	Zfp595	1	0	0	1	0	2
mmu-miR-223-3p	218442	Serinc5	0	0	1	1	0	2
mmu-miR-223-3p	218454	Lhfpl2	0	0	1	1	0	2
mmu-miR-223-3p	218503	Fcho2	0	0	1	1	0	2
mmu-miR-223-3p	218544	Sgtb	0	0	1	1	0	2
mmu-miR-223-3p	218630	Ccno	0	0	1	1	0	2
mmu-miR-223-3p	218639	Arl15	0	0	1	1	0	2
mmu-miR-223-3p	218820	Zfp503	0	1	0	1	0	2
mmu-miR-223-3p	218885	Oxnad1	0	0	1	1	0	2
mmu-miR-223-3p	219022	Ttc5	0	0	1	1	0	2
mmu-miR-223-3p	219024	Tmem55b	0	0	1	1	0	2

mmu-miR-223-3p	219094	Khnyn	0	0	1	1	0	2
mmu-miR-223-3p	219149	Xkr6	1	0	0	0	1	2
mmu-miR-223-3p	219151	Scara3	0	0	1	1	0	2
mmu-miR-223-3p	219170	Fam216b	0	0	1	1	0	2
mmu-miR-223-3p	219189	Vwa8	0	1	0	1	0	2
mmu-miR-223-3p	223267	Ggact	1	0	0	1	0	2
mmu-miR-223-3p	223433	Fam105a	0	0	1	1	0	2
mmu-miR-223-3p	223455	March6	1	0	0	1	0	2
mmu-miR-223-3p	223527	Eny2	0	0	1	1	0	2
mmu-miR-223-3p	223593	E430025E21Rik	0	0	1	1	0	2
mmu-miR-223-3p	223648	Ccdc166	1	0	0	1	0	2
mmu-miR-223-3p	223649	Nrbp2	0	0	1	1	0	2
mmu-miR-223-3p	223701	Mkl1	0	0	1	1	0	2
mmu-miR-223-3p	223726	Mpped1	0	0	1	1	0	2
mmu-miR-223-3p	223752	Gramd4	0	0	1	1	0	2
mmu-miR-223-3p	223770	Brd1	0	0	1	1	0	2
mmu-miR-223-3p	223918	Spryd3	1	0	0	1	0	2
mmu-miR-223-3p	223920	Soat2	0	0	1	1	0	2
mmu-miR-223-3p	223970	Rmi2	1	0	0	1	0	2
mmu-miR-223-3p	223978	Cpped1	1	0	1	0	0	2
mmu-miR-223-3p	224020	Pi4ka	0	1	0	1	0	2
mmu-miR-223-3p	224044	Cyp2ab1	1	0	0	1	0	2
mmu-miR-223-3p	224045	Eif2b5	0	0	1	1	0	2
mmu-miR-223-3p	224055	Rtp2	0	0	1	1	0	2
mmu-miR-223-3p	224065	Uts2b	0	0	1	1	0	2
mmu-miR-223-3p	224090	Tmem44	0	0	1	1	0	2
mmu-miR-223-3p	224111	Ubxn7	1	0	0	1	0	2
mmu-miR-223-3p	224129	Adcy5	0	0	1	1	0	2
mmu-miR-223-3p	224170	Dzip3	1	0	0	1	0	2
mmu-miR-223-3p	224344	Rbm11	0	0	1	1	0	2
mmu-miR-223-3p	224454	Zdhhc14	1	0	0	1	0	2
mmu-miR-223-3p	224617	Tbc1d24	0	0	1	1	0	2
mmu-miR-223-3p	224619	Traf7	0	1	0	1	0	2
mmu-miR-223-3p	224630	Bnip1	1	1	0	0	0	2
mmu-miR-223-3p	224703	March2	0	0	1	1	0	2
mmu-miR-223-3p	224794	Enpp4	0	0	1	1	0	2
mmu-miR-223-3p	224807	Tmem63b	0	1	0	1	0	2
mmu-miR-223-3p	224813	Lrrc73	0	0	1	1	0	2
mmu-miR-223-3p	224823	Rrp36	0	0	1	1	0	2
mmu-miR-223-3p	224897	Dpp9	1	0	0	1	0	2
mmu-miR-223-3p	224938	Pja2	1	0	0	1	0	2
mmu-miR-223-3p	225004	BC027072	1	0	1	0	0	2
mmu-miR-223-3p	225028	Map4k3	0	0	1	1	0	2
mmu-miR-223-3p	225115	Svil	0	1	0	1	0	2
mmu-miR-223-3p	225131	Wac	0	0	1	1	0	2
mmu-miR-223-3p	225187	Ankrd29	1	0	0	1	0	2
mmu-miR-223-3p	225207	Zfp521	0	1	0	1	0	2
mmu-miR-223-3p	225283	Rprd1a	0	0	1	1	0	2
mmu-miR-223-3p	225363	Etf1	0	0	1	1	0	2
mmu-miR-223-3p	225724	Mapk4	0	0	1	1	0	2
mmu-miR-223-3p	225743	Rnf165	1	0	0	1	0	2
mmu-miR-223-3p	225888	Suv420h1	0	0	0	1	1	2
mmu-miR-223-3p	225896	Ubxn1	0	0	1	1	0	2
mmu-miR-223-3p	225922	Plac1l	1	0	0	1	0	2
mmu-miR-223-3p	226025	Trpm3	0	0	1	1	0	2

mmu-miR-223-3p	226139	Cox15	1	0	0	1	0	2
mmu-miR-223-3p	226144	Erlin1	0	0	1	1	0	2
mmu-miR-223-3p	226162	Dpcd	0	0	1	1	0	2
mmu-miR-223-3p	226250	Afap1l2	0	0	1	1	0	2
mmu-miR-223-3p	226251	Ablim1	1	0	0	1	0	2
mmu-miR-223-3p	226541	Klhl20	1	0	0	1	0	2
mmu-miR-223-3p	226751	Cdc42bpa	0	1	0	1	0	2
mmu-miR-223-3p	226781	Slc30a10	0	0	1	1	0	2
mmu-miR-223-3p	226830	Smyd2	0	0	1	1	0	2
mmu-miR-223-3p	226844	Mfsd7b	0	0	1	1	0	2
mmu-miR-223-3p	226922	Kcnq5	1	0	0	1	0	2
mmu-miR-223-3p	226971	Plekhb2	0	0	1	1	0	2
mmu-miR-223-3p	226976	Kansl3	1	0	1	0	0	2
mmu-miR-223-3p	226977	Actr1b	0	0	1	1	0	2
mmu-miR-223-3p	227094	Tmem194b	1	0	0	1	0	2
mmu-miR-223-3p	227102	Ormdl1	0	1	0	1	0	2
mmu-miR-223-3p	227154	Stradb	1	1	0	0	0	2
mmu-miR-223-3p	227290	Aamp	0	0	1	1	0	2
mmu-miR-223-3p	227331	Gigyf2	1	0	0	1	0	2
mmu-miR-223-3p	227357	Espnl	0	0	1	1	0	2
mmu-miR-223-3p	227377	Farp2	0	0	1	1	0	2
mmu-miR-223-3p	227394	Slco4c1	0	0	1	1	0	2
mmu-miR-223-3p	227449	Zcchc2	1	0	0	1	0	2
mmu-miR-223-3p	227612	Tor4a	1	0	0	1	0	2
mmu-miR-223-3p	227632	Kcnt1	0	0	1	1	0	2
mmu-miR-223-3p	227656	Rexo4	0	0	1	1	0	2
mmu-miR-223-3p	227659	Slc2a6	0	0	1	1	0	2
mmu-miR-223-3p	227674	Ddx31	0	0	1	1	0	2
mmu-miR-223-3p	227682	Trub2	0	0	1	1	0	2
mmu-miR-223-3p	227693	Zer1	0	0	1	1	0	2
mmu-miR-223-3p	227715	Exosc2	1	0	0	1	0	2
mmu-miR-223-3p	227717	Qrfp	0	0	1	1	0	2
mmu-miR-223-3p	227723	Prrc2b	1	0	0	1	0	2
mmu-miR-223-3p	227801	Dennd1a	0	1	0	1	0	2
mmu-miR-223-3p	227929	Cytip	0	0	1	1	0	2
mmu-miR-223-3p	227933	Ccdc148	1	0	0	1	0	2
mmu-miR-223-3p	228357	Lrp4	0	0	1	1	0	2
mmu-miR-223-3p	228366	Gyltl1b	0	1	0	1	0	2
mmu-miR-223-3p	228598	Ebf4	0	1	0	1	0	2
mmu-miR-223-3p	228602	4930402H24Rik	0	0	1	1	0	2
mmu-miR-223-3p	228714	Csrp2bp	0	0	1	1	0	2
mmu-miR-223-3p	228767	BC052486	1	0	0	1	0	2
mmu-miR-223-3p	228770	Rspo4	1	0	1	0	0	2
mmu-miR-223-3p	228777	Nrsn2	0	0	1	1	0	2
mmu-miR-223-3p	228785	Mylk2	0	0	1	1	0	2
mmu-miR-223-3p	228787	Xkr7	0	0	1	1	0	2
mmu-miR-223-3p	228876	Zfp334	0	0	1	1	0	2
mmu-miR-223-3p	228889	Ddx27	0	1	0	1	0	2
mmu-miR-223-3p	228911	Tshz2	0	0	1	1	0	2
mmu-miR-223-3p	228913	Zfp217	0	0	1	1	0	2
mmu-miR-223-3p	228966	Ppp1r3d	1	0	0	1	0	2
mmu-miR-223-3p	228983	Osbpl2	1	0	0	1	0	2
mmu-miR-223-3p	228998	Arfgap1	1	0	0	1	0	2
mmu-miR-223-3p	229004	Gmeb2	0	0	1	1	0	2
mmu-miR-223-3p	229007	Zgpat	1	0	0	1	0	2

mmu-miR-223-3p	229317	Eif2a	0	0	1	1	0	2
mmu-miR-223-3p	229488	Fam160a1	0	1	0	1	0	2
mmu-miR-223-3p	229504	Isg20l2	0	0	1	1	0	2
mmu-miR-223-3p	229512	Smg5	0	0	1	1	0	2
mmu-miR-223-3p	229521	Syt11	0	0	1	1	0	2
mmu-miR-223-3p	229571	Gm4858	0	0	1	1	0	2
mmu-miR-223-3p	229589	Prune	0	0	1	1	0	2
mmu-miR-223-3p	229593	Golph3l	0	0	1	1	0	2
mmu-miR-223-3p	229595	Adamtsl4	0	0	1	1	0	2
mmu-miR-223-3p	229603	Otud7b	1	0	0	1	0	2
mmu-miR-223-3p	229658	Vangl1	1	0	0	1	0	2
mmu-miR-223-3p	229672	Bcl2l15	1	0	0	1	0	2
mmu-miR-223-3p	229681	St7l	1	0	0	1	0	2
mmu-miR-223-3p	229699	Slc16a4	1	0	0	1	0	2
mmu-miR-223-3p	229709	Ahcyl1	1	0	0	1	0	2
mmu-miR-223-3p	229725	Clcc1	0	0	1	1	0	2
mmu-miR-223-3p	229759	Olfm3	1	0	0	1	0	2
mmu-miR-223-3p	229905	Ccbl2	1	1	0	0	0	2
mmu-miR-223-3p	230099	Car9	0	0	1	1	0	2
mmu-miR-223-3p	230145	Galnt12	1	0	0	1	0	2
mmu-miR-223-3p	230233	Ikbkap	0	0	1	1	0	2
mmu-miR-223-3p	230234	BC026590	0	0	1	1	0	2
mmu-miR-223-3p	230235	Frrs1l	1	0	0	1	0	2
mmu-miR-223-3p	230257	Ptbp3	1	0	0	1	0	2
mmu-miR-223-3p	230259	E130308A19Rik	0	1	0	1	0	2
mmu-miR-223-3p	230279	6330416G13Rik	0	0	1	1	0	2
mmu-miR-223-3p	230316	Megf9	1	1	0	0	0	2
mmu-miR-223-3p	230379	Acer2	0	0	1	1	0	2
mmu-miR-223-3p	230459	Cyp2j13	1	0	0	1	0	2
mmu-miR-223-3p	230514	Leprot	0	0	1	1	0	2
mmu-miR-223-3p	230582	Cyb5rl	0	1	0	1	0	2
mmu-miR-223-3p	230598	Nrd1	1	0	0	1	0	2
mmu-miR-223-3p	230603	Ttc39a	1	0	0	1	0	2
mmu-miR-223-3p	230613	Skint10	1	0	1	0	0	2
mmu-miR-223-3p	230678	Tmem125	1	0	0	1	0	2
mmu-miR-223-3p	230735	Epha10	1	0	0	1	0	2
mmu-miR-223-3p	230753	Thrap3	1	0	0	1	0	2
mmu-miR-223-3p	230757	5730409E04Rik	0	0	1	1	0	2
mmu-miR-223-3p	230761	Zfp362	1	0	0	1	0	2
mmu-miR-223-3p	230767	Iqcc	0	1	0	1	0	2
mmu-miR-223-3p	230770	Tmem39b	0	1	0	1	0	2
mmu-miR-223-3p	230779	Serinc2	0	0	1	1	0	2
mmu-miR-223-3p	230787	Themis2	0	1	0	1	0	2
mmu-miR-223-3p	230810	Slc30a2	1	0	0	0	1	2
mmu-miR-223-3p	230815	Man1c1	0	0	1	1	0	2
mmu-miR-223-3p	230824	Grhl3	0	0	1	1	0	2
mmu-miR-223-3p	230828	Il22ra1	1	0	0	1	0	2
mmu-miR-223-3p	230863	Sh2d5	1	0	0	1	0	2
mmu-miR-223-3p	230908	Tardbp	1	0	0	1	0	2
mmu-miR-223-3p	230936	Phf13	1	0	0	1	0	2
mmu-miR-223-3p	230996	9430015G10Rik	0	0	1	1	0	2
mmu-miR-223-3p	231014	9330182L06Rik	0	1	0	1	0	2
mmu-miR-223-3p	231134	Dok7	0	1	0	1	0	2
mmu-miR-223-3p	231148	Ablim2	0	1	0	1	0	2
mmu-miR-223-3p	231151	Tada2b	1	0	0	1	0	2

mmu-miR-223-3p	231201	AF366264	0	0	1	1	0	2
mmu-miR-223-3p	231225	Tapt1	0	1	0	1	0	2
mmu-miR-223-3p	231279	Guf1	1	0	0	1	0	2
mmu-miR-223-3p	231327	Ppat	0	1	0	1	0	2
mmu-miR-223-3p	231380	Uba6	0	0	1	1	0	2
mmu-miR-223-3p	231382	Tmprss11d	0	0	1	1	0	2
mmu-miR-223-3p	231413	Grsf1	1	0	0	1	0	2
mmu-miR-223-3p	231440	Parm1	0	0	1	1	0	2
mmu-miR-223-3p	231464	Cnot6l	0	1	0	1	0	2
mmu-miR-223-3p	231580	Gak	0	1	0	1	0	2
mmu-miR-223-3p	231668	Vsig10	0	0	1	1	0	2
mmu-miR-223-3p	231672	Fbxw8	1	0	0	1	0	2
mmu-miR-223-3p	231805	Pilra	1	0	0	1	0	2
mmu-miR-223-3p	231821	Adap1	0	0	1	1	0	2
mmu-miR-223-3p	231830	Micall2	0	0	1	1	0	2
mmu-miR-223-3p	231861	Tnrc18	1	0	0	1	0	2
mmu-miR-223-3p	231915	Uspl1	1	0	0	1	0	2
mmu-miR-223-3p	232087	Mat2a	0	0	1	1	0	2
mmu-miR-223-3p	232156	Slc4a5	1	0	0	1	0	2
mmu-miR-223-3p	232210	Hmces	0	0	1	1	0	2
mmu-miR-223-3p	232223	Txnrd3	0	0	1	1	0	2
mmu-miR-223-3p	232227	Iqsec1	1	0	0	1	0	2
mmu-miR-223-3p	232232	Hdac11	1	0	1	0	0	2
mmu-miR-223-3p	232413	Clec12a	0	0	1	1	0	2
mmu-miR-223-3p	232431	Gprc5a	1	0	0	1	0	2
mmu-miR-223-3p	232493	Gys2	1	0	0	1	0	2
mmu-miR-223-3p	232585	Vwde	1	0	0	1	0	2
mmu-miR-223-3p	232748	Fam115c	1	0	0	1	0	2
mmu-miR-223-3p	232934	Mypop	0	1	0	1	0	2
mmu-miR-223-3p	233046	Rasgrp4	0	0	1	1	0	2
mmu-miR-223-3p	233056	Zfp790	0	0	1	1	0	2
mmu-miR-223-3p	233060	Zfp382	0	0	1	1	0	2
mmu-miR-223-3p	233071	Arhgap33	0	1	0	1	0	2
mmu-miR-223-3p	233204	Tbc1d17	0	1	0	1	0	2
mmu-miR-223-3p	233210	Prr12	0	0	1	1	0	2
mmu-miR-223-3p	233222	Mrgpra3	0	0	1	1	0	2
mmu-miR-223-3p	233406	Prc1	0	0	1	1	0	2
mmu-miR-223-3p	233410	Zfp592	0	1	0	1	0	2
mmu-miR-223-3p	233532	Rsf1	0	1	0	1	0	2
mmu-miR-223-3p	233552	Gdpd5	0	0	1	1	0	2
mmu-miR-223-3p	233575	Pgap2	1	0	0	1	0	2
mmu-miR-223-3p	233651	Dchs1	0	1	0	1	0	2
mmu-miR-223-3p	233799	Acsm2	1	0	0	1	0	2
mmu-miR-223-3p	233802	Thumpd1	0	0	1	1	0	2
mmu-miR-223-3p	233812	BC030336	1	0	0	1	0	2
mmu-miR-223-3p	233865	D430042O09Rik	0	0	1	1	0	2
mmu-miR-223-3p	233899	Gm166	1	0	0	1	0	2
mmu-miR-223-3p	233900	Rnf40	0	0	1	1	0	2
mmu-miR-223-3p	233977	Ppfia1	0	1	0	1	0	2
mmu-miR-223-3p	234076	Tmco3	0	0	1	1	0	2
mmu-miR-223-3p	234094	Arhgef10	1	0	0	1	0	2
mmu-miR-223-3p	234267	Gpm6a	0	1	0	1	0	2
mmu-miR-223-3p	234329	Trim60	1	0	0	1	0	2
mmu-miR-223-3p	234358	Zfp930	1	0	0	1	0	2
mmu-miR-223-3p	234366	Gatad2a	1	0	0	1	0	2

mmu-miR-223-3p	234373	Sugp2	0	1	0	1	0	2
mmu-miR-223-3p	234384	Mpv17l2	1	0	0	1	0	2
mmu-miR-223-3p	234515	Inpp4b	0	1	0	1	0	2
mmu-miR-223-3p	234577	Cpne2	0	0	1	1	0	2
mmu-miR-223-3p	234678	D230025D16Rik	1	0	0	1	0	2
mmu-miR-223-3p	234683	Elmo3	1	0	0	1	0	2
mmu-miR-223-3p	234728	Cmtr2	1	0	1	0	0	2
mmu-miR-223-3p	234788	Slc38a8	1	0	0	1	0	2
mmu-miR-223-3p	234814	Mthfsd	1	0	0	1	0	2
mmu-miR-223-3p	234852	Chmp1a	0	0	1	1	0	2
mmu-miR-223-3p	235033	Rdh8	1	0	0	1	0	2
mmu-miR-223-3p	235044	BC018242	0	0	1	1	0	2
mmu-miR-223-3p	235047	Zfp809	1	0	0	1	0	2
mmu-miR-223-3p	235106	Ntm	0	1	0	1	0	2
mmu-miR-223-3p	235132	Zbtb44	1	0	0	1	0	2
mmu-miR-223-3p	235134	Nfrkb	0	1	1	0	0	2
mmu-miR-223-3p	235169	Foxred1	0	0	1	1	0	2
mmu-miR-223-3p	235293	Sc5d	0	0	1	1	0	2
mmu-miR-223-3p	235300	Tmem136	0	0	1	1	0	2
mmu-miR-223-3p	235315	Rnf214	0	0	1	1	0	2
mmu-miR-223-3p	235345	4833427G06Rik	0	0	1	1	0	2
mmu-miR-223-3p	235402	Lingo1	1	0	1	0	0	2
mmu-miR-223-3p	235415	Cplx3	1	0	0	1	0	2
mmu-miR-223-3p	235431	Coro2b	0	0	1	1	0	2
mmu-miR-223-3p	235441	Usp3	0	1	0	1	0	2
mmu-miR-223-3p	235497	Leo1	1	0	0	1	0	2
mmu-miR-223-3p	235542	Ppp2r3a	1	0	0	1	0	2
mmu-miR-223-3p	235567	Dnajc13	1	0	0	1	0	2
mmu-miR-223-3p	235574	Atp2c1	0	0	1	1	0	2
mmu-miR-223-3p	235610	Atrip	0	1	0	1	0	2
mmu-miR-223-3p	235636	Rtp3	1	0	0	1	0	2
mmu-miR-223-3p	235661	Dync1li1	0	1	0	1	0	2
mmu-miR-223-3p	235854	Mrgpra4	0	0	1	1	0	2
mmu-miR-223-3p	235907	Zfp71-rs1	1	0	0	1	0	2
mmu-miR-223-3p	236285	Lancl3	1	0	0	1	0	2
mmu-miR-223-3p	236293	Slc22a29	1	0	1	0	0	2
mmu-miR-223-3p	236366	5730507C01Rik	1	0	0	1	0	2
mmu-miR-223-3p	236573	Gbp9	1	0	0	1	0	2
mmu-miR-223-3p	236790	Ddx26b	0	1	0	1	0	2
mmu-miR-223-3p	236794	Slc9a6	1	0	0	1	0	2
mmu-miR-223-3p	236899	Pcyt1b	0	1	0	1	0	2
mmu-miR-223-3p	236920	Stard8	1	0	1	0	0	2
mmu-miR-223-3p	237010	Klhl4	1	0	0	1	0	2
mmu-miR-223-3p	237178	Ppef1	1	0	0	1	0	2
mmu-miR-223-3p	237320	Aldh8a1	0	0	1	1	0	2
mmu-miR-223-3p	237353	Sh3rf3	0	0	1	1	0	2
mmu-miR-223-3p	237411	Zfp938	1	0	0	1	0	2
mmu-miR-223-3p	237422	Ric8b	1	0	0	1	0	2
mmu-miR-223-3p	237436	Gas2l3	1	0	0	1	0	2
mmu-miR-223-3p	237465	Ccdc38	1	0	0	1	0	2
mmu-miR-223-3p	237542	Osbpl8	1	0	0	1	0	2
mmu-miR-223-3p	237553	Trhde	1	0	0	1	0	2
mmu-miR-223-3p	237759	Col23a1	1	0	0	1	0	2
mmu-miR-223-3p	237761	Sowaha	1	0	1	0	0	2
mmu-miR-223-3p	237775	Zfp867	0	0	1	1	0	2

mmu-miR-223-3p	237847	Rtn4rl1	1	0	0	1	0	2
mmu-miR-223-3p	237886	Slfn9	0	0	1	1	0	2
mmu-miR-223-3p	238317	Elmsan1	1	0	0	1	0	2
mmu-miR-223-3p	238330	Irf2bpl	0	1	1	0	0	2
mmu-miR-223-3p	238384	Slc24a4	0	1	0	1	0	2
mmu-miR-223-3p	238662	Spata31d1b	0	1	0	1	0	2
mmu-miR-223-3p	238673	Zfp367	0	0	1	1	0	2
mmu-miR-223-3p	238683	Spata31d1c	0	0	1	1	0	2
mmu-miR-223-3p	238799	Tnpo1	0	1	0	1	0	2
mmu-miR-223-3p	238880	Actbl2	0	0	1	1	0	2
mmu-miR-223-3p	239037	Lrit1	1	0	0	1	0	2
mmu-miR-223-3p	239102	Zfhx2	1	0	1	0	0	2
mmu-miR-223-3p	239133	Dleu7	0	0	1	1	0	2
mmu-miR-223-3p	239273	Abcc4	1	0	0	1	0	2
mmu-miR-223-3p	239336	Rxfp3	0	0	1	1	0	2
mmu-miR-223-3p	239364	Tspyl5	0	0	1	1	0	2
mmu-miR-223-3p	239554	Foxred2	1	0	0	1	0	2
mmu-miR-223-3p	239556	Cacna1i	1	0	0	1	0	2
mmu-miR-223-3p	239570	Ttc38	0	0	1	1	0	2
mmu-miR-223-3p	239647	Pced1b	1	0	0	1	0	2
mmu-miR-223-3p	239759	Liph	0	0	1	1	0	2
mmu-miR-223-3p	239789	Gmnc	0	0	1	1	0	2
mmu-miR-223-3p	239845	Gpr156	1	0	0	1	0	2
mmu-miR-223-3p	239985	Arid1b	0	0	1	1	0	2
mmu-miR-223-3p	240028	Lnpep	0	1	0	1	0	2
mmu-miR-223-3p	240066	Zfp870	1	0	0	1	0	2
mmu-miR-223-3p	240068	Zfp563	1	0	0	1	0	2
mmu-miR-223-3p	240119	St6gal2	1	0	0	1	0	2
mmu-miR-223-3p	240131	Lrrc30	1	0	0	1	0	2
mmu-miR-223-3p	240283	Dmxl1	1	0	0	1	0	2
mmu-miR-223-3p	240328	F830016B08Rik	1	0	0	1	0	2
mmu-miR-223-3p	240334	Pcyox1l	0	0	1	1	0	2
mmu-miR-223-3p	240427	Setbp1	1	0	0	1	0	2
mmu-miR-223-3p	240476	Zfp407	0	1	0	1	0	2
mmu-miR-223-3p	240514	Ccdc85b	1	0	0	1	0	2
mmu-miR-223-3p	240614	Ranbp6	1	0	0	1	0	2
mmu-miR-223-3p	240633	Lipk	1	0	0	1	0	2
mmu-miR-223-3p	240638	Slc16a12	0	0	1	1	0	2
mmu-miR-223-3p	240690	St18	0	0	1	1	0	2
mmu-miR-223-3p	240725	Sulf1	1	0	0	1	0	2
mmu-miR-223-3p	240754	Lax1	1	0	0	1	0	2
mmu-miR-223-3p	240894	Fmo9	0	0	1	1	0	2
mmu-miR-223-3p	241035	Pkhd1	1	0	0	1	0	2
mmu-miR-223-3p	241196	Serpinb13	1	0	0	1	0	2
mmu-miR-223-3p	241289	Ppp1r26	0	0	1	1	0	2
mmu-miR-223-3p	241296	Lrrc8a	0	0	1	1	0	2
mmu-miR-223-3p	241311	Zbtb34	0	1	0	1	0	2
mmu-miR-223-3p	241324	Crb2	1	0	0	1	0	2
mmu-miR-223-3p	241431	Xirp2	0	0	1	1	0	2
mmu-miR-223-3p	241494	Zfp385b	1	0	0	1	0	2
mmu-miR-223-3p	241514	Zfp804a	0	0	1	1	0	2
mmu-miR-223-3p	241547	Harbi1	0	0	1	1	0	2
mmu-miR-223-3p	241576	Ldlrad3	0	0	1	1	0	2
mmu-miR-223-3p	241612	Slc5a12	1	0	0	1	0	2
mmu-miR-223-3p	241624	Exd1	0	0	1	1	0	2

mmu-miR-223-3p	241627	Wdr76	0	0	1	1	0	2
mmu-miR-223-3p	241794	Kcng1	0	0	1	1	0	2
mmu-miR-223-3p	241877	Slc10a5	0	0	1	1	0	2
mmu-miR-223-3p	242050	Igsf10	1	0	0	1	0	2
mmu-miR-223-3p	242083	Ppm1l	1	0	0	1	0	2
mmu-miR-223-3p	242109	Zfp697	1	0	0	1	0	2
mmu-miR-223-3p	242202	Pde5a	1	0	0	1	0	2
mmu-miR-223-3p	242253	Wdr63	0	0	1	1	0	2
mmu-miR-223-3p	242259	Slc44a5	0	0	1	1	0	2
mmu-miR-223-3p	242316	Gdf6	1	0	0	1	0	2
mmu-miR-223-3p	242341	Atp6v0d2	1	0	1	0	0	2
mmu-miR-223-3p	242409	Tmem8b	0	0	1	1	0	2
mmu-miR-223-3p	242443	Grin3a	1	0	0	1	0	2
mmu-miR-223-3p	242466	Zfp462	1	0	0	1	0	2
mmu-miR-223-3p	242484	D630039A03Rik	1	0	1	0	0	2
mmu-miR-223-3p	242509	Bnc2	1	0	0	1	0	2
mmu-miR-223-3p	242557	Atg4c	1	0	0	1	0	2
mmu-miR-223-3p	242585	Slc35d1	0	0	1	1	0	2
mmu-miR-223-3p	242620	Dmrta2	0	0	1	1	0	2
mmu-miR-223-3p	242669	Adc	0	1	0	1	0	2
mmu-miR-223-3p	242747	Zfp933	0	0	1	1	0	2
mmu-miR-223-3p	242819	Rundc3b	1	0	0	1	0	2
mmu-miR-223-3p	243043	Kctd8	0	0	1	1	0	2
mmu-miR-223-3p	243219	2900026A02Rik	1	0	0	1	0	2
mmu-miR-223-3p	243328	Slc29a4	0	0	1	1	0	2
mmu-miR-223-3p	243339	Tmem130	0	0	1	1	0	2
mmu-miR-223-3p	243372	Zfp775	0	0	1	1	0	2
mmu-miR-223-3p	243374	Gimap8	1	0	0	1	0	2
mmu-miR-223-3p	243377	Svs1	0	0	1	1	0	2
mmu-miR-223-3p	243537	Uroc1	1	0	0	1	0	2
mmu-miR-223-3p	243574	Kbtbd8	1	0	0	1	0	2
mmu-miR-223-3p	243634	Ano2	0	1	1	0	0	2
mmu-miR-223-3p	243653	Clec1a	1	0	0	1	0	2
mmu-miR-223-3p	243753	2010107G12Rik	0	1	1	0	0	2
mmu-miR-223-3p	243780	E330009J07Rik	0	1	0	1	0	2
mmu-miR-223-3p	243834	Zfp324	0	0	1	1	0	2
mmu-miR-223-3p	243867	Fbxo46	0	0	1	1	0	2
mmu-miR-223-3p	243880	Nlrp4a	0	1	0	1	0	2
mmu-miR-223-3p	243911	Kirrel2	1	0	0	1	0	2
mmu-miR-223-3p	243914	Lgi4	1	0	0	1	0	2
mmu-miR-223-3p	243961	Shank1	0	1	0	1	0	2
mmu-miR-223-3p	243963	Zfp473	0	1	0	1	0	2
mmu-miR-223-3p	244049	Mctp2	1	0	0	1	0	2
mmu-miR-223-3p	244059	Chd2	0	1	0	1	0	2
mmu-miR-223-3p	244152	Tsku	0	0	1	1	0	2
mmu-miR-223-3p	244198	Olfml1	1	0	0	1	0	2
mmu-miR-223-3p	244199	Ovch2	0	1	0	1	0	2
mmu-miR-223-3p	244202	Nlrp10	0	0	1	1	0	2
mmu-miR-223-3p	244238	Mrgpre	0	0	1	1	0	2
mmu-miR-223-3p	244310	Dlgap2	1	0	0	1	0	2
mmu-miR-223-3p	244329	Mcph1	0	0	1	1	0	2
mmu-miR-223-3p	244562	Abcc12	0	1	0	1	0	2
mmu-miR-223-3p	244579	Tox3	0	0	1	1	0	2
mmu-miR-223-3p	244585	Rpgrip1l	0	0	1	1	0	2
mmu-miR-223-3p	244654	Mtss1l	0	0	1	1	0	2

mmu-miR-223-3p	244864	Layn	1	0	0	1	0	2
mmu-miR-223-3p	244867	Arhgap20	0	1	0	1	0	2
mmu-miR-223-3p	244871	Zc3h12c	0	1	0	1	0	2
mmu-miR-223-3p	244879	Npat	0	0	1	1	0	2
mmu-miR-223-3p	245038	Dclk3	1	0	0	1	0	2
mmu-miR-223-3p	245049	Myrip	0	0	1	1	0	2
mmu-miR-223-3p	245282	Apol10a	0	0	1	1	0	2
mmu-miR-223-3p	245522	Zc4h2	0	0	1	1	0	2
mmu-miR-223-3p	245525	Hsf3	0	0	1	1	0	2
mmu-miR-223-3p	245537	Nlgn3	0	0	1	1	0	2
mmu-miR-223-3p	245610	Nxf3	1	0	0	1	0	2
mmu-miR-223-3p	245631	Mum1l1	0	0	1	1	0	2
mmu-miR-223-3p	245638	Tbc1d8b	1	0	0	1	0	2
mmu-miR-223-3p	245684	Cnksr2	1	0	0	1	0	2
mmu-miR-223-3p	245880	Wasf3	0	0	1	1	0	2
mmu-miR-223-3p	245886	Ankrd27	1	0	0	1	0	2
mmu-miR-223-3p	245902	Ccdc15	1	0	0	1	0	2
mmu-miR-223-3p	245944	Vps54	1	0	0	1	0	2
mmu-miR-223-3p	246086	Onecut3	1	0	0	1	0	2
mmu-miR-223-3p	246103	Atxn7	0	0	1	1	0	2
mmu-miR-223-3p	246228	Vwa1	0	0	1	1	0	2
mmu-miR-223-3p	246293	Klhl8	0	0	1	1	0	2
mmu-miR-223-3p	246694	Hps5	0	1	0	1	0	2
mmu-miR-223-3p	246710	Rhobtb2	0	0	1	1	0	2
mmu-miR-223-3p	246728	Oas2	1	0	0	1	0	2
mmu-miR-223-3p	246738	Dnajc28	0	1	0	1	0	2
mmu-miR-223-3p	246782	Atpaf2	0	0	1	1	0	2
mmu-miR-223-3p	252829	Obox5	0	0	1	1	0	2
mmu-miR-223-3p	252838	Tox	0	0	1	1	0	2
mmu-miR-223-3p	252973	Grhl2	1	0	0	1	0	2
mmu-miR-223-3p	258019	Olfr212	0	0	1	1	0	2
mmu-miR-223-3p	258330	Olfr1274-ps	1	0	0	1	0	2
mmu-miR-223-3p	258513	Olfr536	1	0	0	1	0	2
mmu-miR-223-3p	258560	Olfr843	1	0	0	1	0	2
mmu-miR-223-3p	259097	Olfr558	0	0	1	1	0	2
mmu-miR-223-3p	259172	Mfrp	0	0	1	1	0	2
mmu-miR-223-3p	260299	Cadm4	1	0	0	1	0	2
mmu-miR-223-3p	260302	Gga3	1	0	0	1	0	2
mmu-miR-223-3p	260305	Nphp4	1	0	0	1	0	2
mmu-miR-223-3p	260408	Prss45	0	0	1	1	0	2
mmu-miR-223-3p	260423	Hist1h3f	0	0	1	1	0	2
mmu-miR-223-3p	263406	Plekhg3	0	0	1	1	0	2
mmu-miR-223-3p	264134	Ttc26	0	1	0	1	0	2
mmu-miR-223-3p	266645	Acmsd	1	0	0	1	0	2
mmu-miR-223-3p	266815	Mill1	1	0	0	1	0	2
mmu-miR-223-3p	268281	Shprh	0	0	1	1	0	2
mmu-miR-223-3p	268288	Samd3	0	0	1	1	0	2
mmu-miR-223-3p	268294	Zbtb24	0	0	1	1	0	2
mmu-miR-223-3p	268297	Scml4	0	0	1	1	0	2
mmu-miR-223-3p	268301	Sowahc	1	0	0	1	0	2
mmu-miR-223-3p	268445	Ankrd13b	0	1	1	0	0	2
mmu-miR-223-3p	268481	Krt222	1	0	0	1	0	2
mmu-miR-223-3p	268490	Lsm12	0	0	1	1	0	2
mmu-miR-223-3p	268491	Gm1564	1	0	0	1	0	2
mmu-miR-223-3p	268670	Zfp759	1	0	0	1	0	2

mmu-miR-223-3p	268697	Ccnb1	1	0	0	1	0	2
mmu-miR-223-3p	268783	Mtmr12	0	0	1	1	0	2
mmu-miR-223-3p	268882	Fbxo45	0	0	1	1	0	2
mmu-miR-223-3p	268932	Caskin1	0	0	1	1	0	2
mmu-miR-223-3p	268996	Ss18	0	1	0	1	0	2
mmu-miR-223-3p	269003	Sap130	0	0	1	1	0	2
mmu-miR-223-3p	269060	Dagla	1	0	0	1	0	2
mmu-miR-223-3p	269109	Dpp10	1	0	0	1	0	2
mmu-miR-223-3p	269132	Colgalt2	0	0	1	1	0	2
mmu-miR-223-3p	269198	Nbeal1	0	0	1	1	0	2
mmu-miR-223-3p	269209	Stk36	0	1	1	0	0	2
mmu-miR-223-3p	269252	Gtf3c4	0	0	1	1	0	2
mmu-miR-223-3p	269254	Setx	1	0	0	1	0	2
mmu-miR-223-3p	269275	Acvr1c	1	0	0	1	0	2
mmu-miR-223-3p	269397	Ss18l1	0	0	1	1	0	2
mmu-miR-223-3p	269401	Znf512b	0	1	0	1	0	2
mmu-miR-223-3p	269437	Plch1	1	0	0	1	0	2
mmu-miR-223-3p	269585	Zscan20	1	0	0	1	0	2
mmu-miR-223-3p	269587	Epb4.1	0	1	0	1	0	2
mmu-miR-223-3p	269604	Gpr157	0	0	1	1	0	2
mmu-miR-223-3p	269623	Rbm48	0	0	1	1	0	2
mmu-miR-223-3p	269717	Orai2	1	0	0	1	0	2
mmu-miR-223-3p	269800	Zfp384	1	0	0	1	0	2
mmu-miR-223-3p	270028	Fam155a	0	1	0	1	0	2
mmu-miR-223-3p	270084	Lpcat2	1	0	0	1	0	2
mmu-miR-223-3p	270086	Ogfod1	1	0	0	1	0	2
mmu-miR-223-3p	270096	Mon1b	1	0	0	1	0	2
mmu-miR-223-3p	270097	Vat1l	1	0	0	1	0	2
mmu-miR-223-3p	270110	Irf2bp2	0	1	0	1	0	2
mmu-miR-223-3p	270120	Fat3	1	0	0	1	0	2
mmu-miR-223-3p	270156	AU019823	0	0	1	1	0	2
mmu-miR-223-3p	270163	Myo9a	1	0	0	1	0	2
mmu-miR-223-3p	270198	Pfkfb4	1	0	1	0	0	2
mmu-miR-223-3p	270627	Taf1	1	0	0	1	0	2
mmu-miR-223-3p	270893	Tmem132e	0	0	1	1	0	2
mmu-miR-223-3p	271036	Catsperb	0	0	1	1	0	2
mmu-miR-223-3p	271047	Serpina3b	0	0	1	1	0	2
mmu-miR-223-3p	271375	Cd200r2	0	1	0	1	0	2
mmu-miR-223-3p	271457	Rab5a	1	0	0	1	0	2
mmu-miR-223-3p	271564	Vps13a	1	0	0	1	0	2
mmu-miR-223-3p	272158	Poln	1	0	1	0	0	2
mmu-miR-223-3p	272359	Irf2bp1	1	0	0	1	0	2
mmu-miR-223-3p	272428	Acsm5	1	0	0	1	0	2
mmu-miR-223-3p	272790	Magee2	1	0	0	1	0	2
mmu-miR-223-3p	276950	Slfn8	1	0	0	1	0	2
mmu-miR-223-3p	276952	Rasl10b	0	0	1	1	0	2
mmu-miR-223-3p	277360	Prex1	0	0	1	1	0	2
mmu-miR-223-3p	277432	Vstm2l	0	0	1	1	0	2
mmu-miR-223-3p	277743	Fam131c	0	0	1	1	0	2
mmu-miR-223-3p	277854	Depdc5	1	0	0	1	0	2
mmu-miR-223-3p	277973	Slc9a5	0	0	1	1	0	2
mmu-miR-223-3p	278180	Vsig4	1	0	0	1	0	2
mmu-miR-223-3p	278679	Apol7b	0	1	0	1	0	2
mmu-miR-223-3p	279185	Gm13083	1	0	0	1	0	2
mmu-miR-223-3p	282619	Sbsn	1	0	0	1	0	2

mmu-miR-223-3p	317717	Sec22a	1	0	0	1	0	2
mmu-miR-223-3p	319162	Hist3h2a	0	0	1	1	0	2
mmu-miR-223-3p	319190	Hist2h2be	0	0	1	1	0	2
mmu-miR-223-3p	319200	Gpr82	1	0	0	1	0	2
mmu-miR-223-3p	319207	Pgbd1	1	0	0	1	0	2
mmu-miR-223-3p	319239	Npsr1	0	0	1	1	0	2
mmu-miR-223-3p	319415	Hs3st5	1	0	0	1	0	2
mmu-miR-223-3p	319455	Pld5	1	0	0	1	0	2
mmu-miR-223-3p	319468	Ppm1h	1	0	0	1	0	2
mmu-miR-223-3p	319475	Zfp672	1	0	0	1	0	2
mmu-miR-223-3p	319513	Pced1a	1	0	0	1	0	2
mmu-miR-223-3p	319535	Zfp182	1	0	0	1	0	2
mmu-miR-223-3p	319552	B230216G23Rik	1	0	0	0	1	2
mmu-miR-223-3p	319594	Hif1an	0	0	1	1	0	2
mmu-miR-223-3p	319713	Ablim3	0	0	1	1	0	2
mmu-miR-223-3p	319719	Simc1	0	0	1	1	0	2
mmu-miR-223-3p	319765	Igf2bp2	1	0	1	0	0	2
mmu-miR-223-3p	319776	Tmem72	0	0	1	1	0	2
mmu-miR-223-3p	319801	9630033F20Rik	1	0	0	1	0	2
mmu-miR-223-3p	319807	3110047P20Rik	1	0	0	1	0	2
mmu-miR-223-3p	319922	Vwc2	0	1	0	1	0	2
mmu-miR-223-3p	319944	Taf2	1	0	0	1	0	2
mmu-miR-223-3p	319945	Flad1	0	0	1	1	0	2
mmu-miR-223-3p	319955	Ercc6	0	0	1	1	0	2
mmu-miR-223-3p	319965	Cc2d1b	0	1	1	0	0	2
mmu-miR-223-3p	319996	Casc4	1	0	0	1	0	2
mmu-miR-223-3p	320040	Rnf222	1	0	0	1	0	2
mmu-miR-223-3p	320100	Relt	1	0	0	1	0	2
mmu-miR-223-3p	320111	Prr18	0	0	1	1	0	2
mmu-miR-223-3p	320127	Dgki	0	0	1	1	0	2
mmu-miR-223-3p	320139	Ptpn7	1	0	0	1	0	2
mmu-miR-223-3p	320148	B430306N03Rik	1	0	0	1	0	2
mmu-miR-223-3p	320181	Fndc7	1	0	0	1	0	2
mmu-miR-223-3p	320183	Msrb3	0	0	1	1	0	2
mmu-miR-223-3p	320207	Pik3r5	0	0	1	1	0	2
mmu-miR-223-3p	320309	1520401A03Rik	1	0	0	1	0	2
mmu-miR-223-3p	320351	Tmem251	0	1	1	0	0	2
mmu-miR-223-3p	320404	Itpkb	0	1	0	1	0	2
mmu-miR-223-3p	320435	Rinl	0	0	1	1	0	2
mmu-miR-223-3p	320492	A830018L16Rik	1	0	0	1	0	2
mmu-miR-223-3p	320541	Slc35e2	0	0	1	1	0	2
mmu-miR-223-3p	320563	Islr2	1	0	0	1	0	2
mmu-miR-223-3p	320609	Strip2	1	0	0	1	0	2
mmu-miR-223-3p	320635	Cyb5r2	1	0	1	0	0	2
mmu-miR-223-3p	320696	Ccdc158	0	1	0	1	0	2
mmu-miR-223-3p	320706	Soga1	1	0	0	1	0	2
mmu-miR-223-3p	320747	Lingo4	0	0	1	1	0	2
mmu-miR-223-3p	320802	Ifitm10	1	0	0	1	0	2
mmu-miR-223-3p	320806	Gfm2	1	0	0	1	0	2
mmu-miR-223-3p	320808	Dcaf5	0	0	1	1	0	2
mmu-miR-223-3p	320840	Negr1	1	0	0	1	0	2
mmu-miR-223-3p	320916	Wscd2	0	0	1	1	0	2
mmu-miR-223-3p	320940	Atp11c	1	0	0	1	0	2
mmu-miR-223-3p	320946	A930035D04Rik	1	0	0	1	0	2
mmu-miR-223-3p	320951	Pisd	0	1	0	1	0	2

mmu-miR-223-3p	320974	Lrrn4	0	0	1	1	0	2
mmu-miR-223-3p	320981	Enpp6	0	0	1	1	0	2
mmu-miR-223-3p	321006	Vprbp	1	1	0	0	0	2
mmu-miR-223-3p	326622	Upf2	0	1	0	1	0	2
mmu-miR-223-3p	327749	Gm5079	1	0	0	1	0	2
mmu-miR-223-3p	327762	Dna2	1	0	0	1	0	2
mmu-miR-223-3p	327958	Pitpnm3	1	0	0	1	0	2
mmu-miR-223-3p	327963	Zfp616	1	0	0	1	0	2
mmu-miR-223-3p	327978	Slfn5	1	0	0	1	0	2
mmu-miR-223-3p	327992	Hsf5	1	0	0	1	0	2
mmu-miR-223-3p	328035	Fads6	0	1	0	1	0	2
mmu-miR-223-3p	328059	Slc7a15	0	0	1	1	0	2
mmu-miR-223-3p	328092	Dtd2	0	0	1	1	0	2
mmu-miR-223-3p	328265	A530001N23Rik	1	0	0	1	0	2
mmu-miR-223-3p	328370	Rft1	1	1	0	0	0	2
mmu-miR-223-3p	328561	Apol10b	0	0	1	1	0	2
mmu-miR-223-3p	328577	7530416G11Rik	1	0	0	1	0	2
mmu-miR-223-3p	328643	Vwa5b2	0	1	0	1	0	2
mmu-miR-223-3p	329002	Zfp236	1	0	0	1	0	2
mmu-miR-223-3p	329003	Zfp516	1	0	0	1	0	2
mmu-miR-223-3p	329152	Hecw2	1	0	0	1	0	2
mmu-miR-223-3p	329430	A330043C09Rik	1	0	0	1	0	2
mmu-miR-223-3p	329506	Ctdspl2	1	0	0	1	0	2
mmu-miR-223-3p	329641	Sertm1	1	0	0	1	0	2
mmu-miR-223-3p	329679	Fnip2	0	1	0	1	0	2
mmu-miR-223-3p	329727	Dennd2c	0	0	1	1	0	2
mmu-miR-223-3p	329731	Fam19a3	1	0	0	1	0	2
mmu-miR-223-3p	329839	Gm829	1	0	0	1	0	2
mmu-miR-223-3p	329872	Frem1	1	0	0	1	0	2
mmu-miR-223-3p	330010	Ttll10	1	1	0	0	0	2
mmu-miR-223-3p	330064	Slc5a6	0	0	1	1	0	2
mmu-miR-223-3p	330096	Shisa3	1	0	0	1	0	2
mmu-miR-223-3p	330222	Sdk1	0	0	1	1	0	2
mmu-miR-223-3p	330228	4933411G11Rik	0	0	1	1	0	2
mmu-miR-223-3p	330260	Pon2	0	0	1	1	0	2
mmu-miR-223-3p	330319	Wipf3	1	0	0	1	0	2
mmu-miR-223-3p	330369	Fbxo41	0	0	1	1	0	2
mmu-miR-223-3p	330409	Cecr2	1	0	0	1	0	2
mmu-miR-223-3p	330450	Far2	1	0	0	1	0	2
mmu-miR-223-3p	330460	Tmem150b	1	0	0	1	0	2
mmu-miR-223-3p	330463	Zfp78	1	0	0	1	0	2
mmu-miR-223-3p	330474	Zc3h4	0	0	1	1	0	2
mmu-miR-223-3p	330577	Fam154b	1	0	0	1	0	2
mmu-miR-223-3p	330627	Trim66	1	0	0	1	0	2
mmu-miR-223-3p	330721	Nek5	1	0	0	1	0	2
mmu-miR-223-3p	330723	Htra4	1	0	0	1	0	2
mmu-miR-223-3p	330788	Zfp866	0	0	1	1	0	2
mmu-miR-223-3p	330812	Rnf150	1	0	0	1	0	2
mmu-miR-223-3p	330814	Lphn1	0	0	1	1	0	2
mmu-miR-223-3p	330914	Arhgap32	1	0	0	1	0	2
mmu-miR-223-3p	330921	Pate2	1	0	1	0	0	2
mmu-miR-223-3p	330951	Gm16130	1	0	0	0	1	2
mmu-miR-223-3p	330959	Snapc5	1	0	0	1	0	2
mmu-miR-223-3p	330998	Ankrd34c	0	0	1	1	0	2
mmu-miR-223-3p	331374	Dgkk	1	0	0	1	0	2

mmu-miR-223-3p	331491	Fnd3c2	1	0	0	1	0	2
mmu-miR-223-3p	331493	Gm5127	0	1	0	1	0	2
mmu-miR-223-3p	331529	Gm5128	0	1	0	1	0	2
mmu-miR-223-3p	331531	AV320801	0	1	0	1	0	2
mmu-miR-223-3p	331537	E230019M04Rik	0	0	1	1	0	2
mmu-miR-223-3p	332397	Nanos1	0	0	1	1	0	2
mmu-miR-223-3p	332578	Lcn10	0	0	1	1	0	2
mmu-miR-223-3p	333329	Cngb1	1	0	0	1	0	2
mmu-miR-223-3p	333564	Fndc3c1	1	0	0	1	0	2
mmu-miR-223-3p	333789	N4bp2	0	1	0	1	0	2
mmu-miR-223-3p	338369	Tmem220	0	0	1	1	0	2
mmu-miR-223-3p	338370	Nalcn	1	0	0	1	0	2
mmu-miR-223-3p	338403	Cndp1	0	1	0	1	0	2
mmu-miR-223-3p	338467	Morc3	0	1	0	1	0	2
mmu-miR-223-3p	338523	Jhdm1d	0	0	1	1	0	2
mmu-miR-223-3p	347708	Dppa1	1	0	0	1	0	2
mmu-miR-223-3p	347712	Pramel7	0	0	1	1	0	2
mmu-miR-223-3p	353170	Txlng	0	0	1	1	0	2
mmu-miR-223-3p	353211	Prune2	1	0	0	1	0	2
mmu-miR-223-3p	353242	Mrpl21	0	1	1	0	0	2
mmu-miR-223-3p	353502	Hcfc1r1	0	1	0	1	0	2
mmu-miR-223-3p	360213	Trim46	1	0	0	1	0	2
mmu-miR-223-3p	368204	Khdc1a	1	0	0	1	0	2
mmu-miR-223-3p	373864	Col27a1	1	0	0	1	0	2
mmu-miR-223-3p	378425	Nlrp12	1	0	0	1	0	2
mmu-miR-223-3p	378430	Nanos2	0	0	1	1	0	2
mmu-miR-223-3p	380669	Lin28b	0	0	1	1	0	2
mmu-miR-223-3p	380683	Sec14l3	1	0	0	1	0	2
mmu-miR-223-3p	380701	Slc47a2	0	0	1	1	0	2
mmu-miR-223-3p	380702	Shisa6	1	0	0	1	0	2
mmu-miR-223-3p	380711	Rap1gap2	0	0	1	1	0	2
mmu-miR-223-3p	380718	Mks1	1	0	0	1	0	2
mmu-miR-223-3p	380752	Tssc1	1	0	0	1	0	2
mmu-miR-223-3p	381113	Cdkl4	0	0	1	1	0	2
mmu-miR-223-3p	381126	Garem	0	0	1	1	0	2
mmu-miR-223-3p	381142	Arl14epl	0	0	1	1	0	2
mmu-miR-223-3p	381148	Prob1	1	0	0	1	0	2
mmu-miR-223-3p	381269	Mreg	0	0	1	1	0	2
mmu-miR-223-3p	381270	March4	1	0	0	1	0	2
mmu-miR-223-3p	381272	A630095N17Rik	1	0	0	1	0	2
mmu-miR-223-3p	381280	Hjurp	1	0	0	1	0	2
mmu-miR-223-3p	381286	Serpinb3c	1	0	1	0	0	2
mmu-miR-223-3p	381287	A530032D15Rik	0	0	1	1	0	2
mmu-miR-223-3p	381353	Gm996	1	0	0	1	0	2
mmu-miR-223-3p	381373	Sp9	1	0	0	1	0	2
mmu-miR-223-3p	381406	2810408M09Rik	0	0	1	1	0	2
mmu-miR-223-3p	381410	Zfp408	0	0	1	1	0	2
mmu-miR-223-3p	381510	Dpy19l4	0	1	0	1	0	2
mmu-miR-223-3p	381511	Pdp1	1	0	0	1	0	2
mmu-miR-223-3p	381536	Gm12789	0	0	1	1	0	2
mmu-miR-223-3p	381538	Mroh7	0	1	0	1	0	2
mmu-miR-223-3p	381546	Ccdc24	0	1	0	1	0	2
mmu-miR-223-3p	381591	L1td1	0	0	1	1	0	2
mmu-miR-223-3p	381622	5031410I06Rik	1	0	0	1	0	2
mmu-miR-223-3p	381686	Kpna7	0	0	1	1	0	2

mmu-miR-223-3p	381759	Wee2	1	0	0	1	0	2
mmu-miR-223-3p	381820	2700089E24Rik	1	0	0	1	0	2
mmu-miR-223-3p	381845	2310014L17Rik	1	0	0	1	0	2
mmu-miR-223-3p	381903	Alg8	0	0	1	1	0	2
mmu-miR-223-3p	381970	Scgb2b2	0	1	0	1	0	2
mmu-miR-223-3p	381974	Mrgprg	1	0	0	1	0	2
mmu-miR-223-3p	381979	Brsk1	1	0	0	1	0	2
mmu-miR-223-3p	381990	Zbtb2	1	0	0	1	0	2
mmu-miR-223-3p	382019	Zfp882	1	0	0	1	0	2
mmu-miR-223-3p	382051	Pdp2	1	0	0	1	0	2
mmu-miR-223-3p	382056	Crtc1	0	0	1	1	0	2
mmu-miR-223-3p	382109	Fbxw26	1	0	0	1	0	2
mmu-miR-223-3p	382111	Susd5	1	0	0	1	0	2
mmu-miR-223-3p	382117	Tcaim	0	1	0	1	0	2
mmu-miR-223-3p	382236	Brwd3	0	1	0	1	0	2
mmu-miR-223-3p	382245	Tmem29	1	0	0	1	0	2
mmu-miR-223-3p	382253	Cdkl5	0	0	1	1	0	2
mmu-miR-223-3p	382571	Kcnf1	1	0	0	1	0	2
mmu-miR-223-3p	382867	Zfp488	0	0	1	1	0	2
mmu-miR-223-3p	382985	Rrm2b	1	0	0	1	0	2
mmu-miR-223-3p	383709	Gm1322	0	0	1	1	0	2
mmu-miR-223-3p	384009	Glipr2	1	0	1	0	0	2
mmu-miR-223-3p	384059	Tlr12	1	0	0	1	0	2
mmu-miR-223-3p	384198	Fam47e	1	0	0	1	0	2
mmu-miR-223-3p	384569	Nova2	1	0	0	1	0	2
mmu-miR-223-3p	384806	Adam20	0	0	1	1	0	2
mmu-miR-223-3p	384817	4930448N21Rik	1	0	0	1	0	2
mmu-miR-223-3p	386463	Cdsn	1	0	0	1	0	2
mmu-miR-223-3p	387314	Tmtc1	1	0	0	1	0	2
mmu-miR-223-3p	387609	Zhx2	0	0	1	1	0	2
mmu-miR-223-3p	399549	H2-M10.6	0	1	1	0	0	2
mmu-miR-223-3p	399568	BC052040	1	0	0	1	0	2
mmu-miR-223-3p	399603	Fam84b	1	0	0	1	0	2
mmu-miR-223-3p	404545	Ano7	1	0	0	1	0	2
mmu-miR-223-3p	404634	H2afy2	1	0	0	1	0	2
mmu-miR-223-3p	404710	Iqgap3	0	0	1	1	0	2
mmu-miR-223-3p	406218	Panx2	0	1	0	1	0	2
mmu-miR-223-3p	407786	Taf9b	0	0	1	1	0	2
mmu-miR-223-3p	407789	BC048644	0	0	1	1	0	2
mmu-miR-223-3p	407800	Ecm2	0	0	1	1	0	2
mmu-miR-223-3p	407812	Zfp941	0	0	1	1	0	2
mmu-miR-223-3p	407814	BC053393	1	0	0	1	0	2
mmu-miR-223-3p	407823	Baz2b	1	0	1	0	0	2
mmu-miR-223-3p	408065	Zfp456	0	1	0	1	0	2
mmu-miR-223-3p	408068	Zfp738	1	0	0	1	0	2
mmu-miR-223-3p	414084	Tnip3	1	0	0	1	0	2
mmu-miR-223-3p	425051	D930019O06Rik	1	0	0	1	0	2
mmu-miR-223-3p	432467	Hnrnph3	1	0	1	0	0	2
mmu-miR-223-3p	432508	Cpsf6	1	0	0	1	0	2
mmu-miR-223-3p	432582	E130309D14Rik	0	0	1	1	0	2
mmu-miR-223-3p	432600	Gm11568	1	0	0	1	0	2
mmu-miR-223-3p	433323	Sgpp2	1	0	0	1	0	2
mmu-miR-223-3p	433375	Creg1	1	0	0	1	0	2
mmu-miR-223-3p	433470	AA467197	1	0	0	1	0	2
mmu-miR-223-3p	433658	Gm15688	1	0	0	1	0	2

mmu-miR-223-3p	434179	Gm5595	1	0	0	1	0	2
mmu-miR-223-3p	434197	Fam169b	1	0	0	1	0	2
mmu-miR-223-3p	434215	Lrrc32	0	0	1	1	0	2
mmu-miR-223-3p	434428	Gm5620	1	0	0	1	0	2
mmu-miR-223-3p	434438	Ccdc36	1	0	0	1	0	2
mmu-miR-223-3p	435273	Krtap1-3	1	0	0	1	0	2
mmu-miR-223-3p	435965	Lrp3	0	0	1	1	0	2
mmu-miR-223-3p	436240	Foxr2	1	0	0	1	0	2
mmu-miR-223-3p	442827	Rab44	0	0	1	1	0	2
mmu-miR-223-3p	449521	Zfp213	0	0	1	1	0	2
mmu-miR-223-3p	503610	Zdhhc18	0	0	1	1	0	2
mmu-miR-223-3p	504193	Npcd	1	0	0	1	0	2
mmu-miR-223-3p	544678	2010015L04Rik	0	0	1	1	0	2
mmu-miR-223-3p	544806	Tmem92	0	0	1	1	0	2
mmu-miR-223-3p	544963	Iqgap2	0	0	1	1	0	2
mmu-miR-223-3p	545291	Hpse2	0	0	1	1	0	2
mmu-miR-223-3p	545366	Cfhr2	1	0	0	1	0	2
mmu-miR-223-3p	545370	Hmcn1	1	0	0	1	0	2
mmu-miR-223-3p	545474	Scrt2	0	1	0	1	0	2
mmu-miR-223-3p	545758	Gm5868	1	0	0	1	0	2
mmu-miR-223-3p	545938	Zfp607	0	0	1	1	0	2
mmu-miR-223-3p	545975	Cers3	1	0	0	1	0	2
mmu-miR-223-3p	546049	C330021F23Rik	0	0	1	1	0	2
mmu-miR-223-3p	546058	AA386476	1	0	0	1	0	2
mmu-miR-223-3p	546123	Gm5916	1	0	0	1	0	2
mmu-miR-223-3p	546134	Gramd2	0	0	1	1	0	2
mmu-miR-223-3p	546144	Wdr72	0	0	1	1	0	2
mmu-miR-223-3p	546336	Prrg1	1	0	0	1	0	2
mmu-miR-223-3p	547253	Parp14	1	0	0	1	0	2
mmu-miR-223-3p	619297	C430049E01Rik	1	0	0	1	0	2
mmu-miR-223-3p	619309	A630081J09Rik	1	0	0	1	0	2
mmu-miR-223-3p	619310	Zfp872	1	0	0	1	0	2
mmu-miR-223-3p	619326	9130409I23Rik	0	0	1	1	0	2
mmu-miR-223-3p	619441	BC096441	1	0	0	1	0	2
mmu-miR-223-3p	619597	Gm6086	1	0	0	1	0	2
mmu-miR-223-3p	619665	Klf14	1	0	0	1	0	2
mmu-miR-223-3p	622665	Ccdc17	0	0	1	1	0	2
mmu-miR-223-3p	622675	Zfp827	0	0	1	1	0	2
mmu-miR-223-3p	624286	Cfhr3	1	0	0	1	0	2
mmu-miR-223-3p	624860	Gm12253	0	0	1	1	0	2
mmu-miR-223-3p	624866	Lekr1	0	0	1	1	0	2
mmu-miR-223-3p	625638	Fam43b	0	0	1	1	0	2
mmu-miR-223-3p	626316	Gm13051	1	0	0	1	0	2
mmu-miR-223-3p	626596	Rgs22	0	1	0	1	0	2
mmu-miR-223-3p	626848	Etohi1	1	0	0	1	0	2
mmu-miR-223-3p	626870	Gm11992	0	0	1	1	0	2
mmu-miR-223-3p	627049	Zfp800	0	0	1	1	0	2
mmu-miR-223-3p	627302	Gm14092	0	0	1	1	0	2
mmu-miR-223-3p	627626	Ptchd4	1	0	0	1	0	2
mmu-miR-223-3p	628813	Gm11437	0	0	1	1	0	2
mmu-miR-223-3p	628919	Gm6934	1	0	0	1	0	2
mmu-miR-223-3p	637093	Gm362	1	0	0	1	0	2
mmu-miR-223-3p	637515	Nlrp1b	0	1	0	1	0	2
mmu-miR-223-3p	641376	Tomm40l	0	0	1	1	0	2
mmu-miR-223-3p	653016	Gm7325	1	0	0	1	0	2

mmu-miR-223-3p	664799	Ctcfl	1	0	0	1	0	2
mmu-miR-223-3p	664804	Gm7347	1	0	0	1	0	2
mmu-miR-223-3p	664883	Nova1	1	0	0	1	0	2
mmu-miR-223-3p	665270	Plb1	1	0	0	1	0	2
mmu-miR-223-3p	665622	Hist1h2br	1	0	0	1	0	2
mmu-miR-223-3p	666040	Gm7903	0	1	0	1	0	2
mmu-miR-223-3p	666173	Vps13b	0	1	0	1	0	2
mmu-miR-223-3p	666185	Gm7969	1	0	0	1	0	2
mmu-miR-223-3p	666348	Apol7e	0	1	0	1	0	2
mmu-miR-223-3p	666468	Atg4a	1	0	0	1	0	2
mmu-miR-223-3p	666794	Rbm24	1	0	0	1	0	2
mmu-miR-223-3p	666806	Gm8300	1	0	0	1	0	2
mmu-miR-223-3p	666921	Gm12886	1	0	0	1	0	2
mmu-miR-223-3p	666926	Gm8369	1	0	0	1	0	2
mmu-miR-223-3p	666927	Gm12887	1	0	0	1	0	2
mmu-miR-223-3p	667742	Piezo2	0	1	0	1	0	2
mmu-miR-223-3p	668166	Zxdb	1	0	0	1	0	2
mmu-miR-223-3p	668303	Kif26a	1	0	0	1	0	2
mmu-miR-223-3p	668359	Gm9125	1	0	0	1	0	2
mmu-miR-223-3p	670880	Gm9507	1	0	0	1	0	2
mmu-miR-223-3p	674321	LOC674321	1	0	0	1	0	2
mmu-miR-223-3p	676710	LOC676710	1	0	0	1	0	2
mmu-miR-223-3p	1E+08	Rsph3b	1	0	0	1	0	2
mmu-miR-223-3p	1E+08	Fam174b	0	1	0	1	0	2
mmu-miR-223-3p	1E+08	Gm10603	1	0	0	1	0	2
mmu-miR-223-3p	1E+08	Gm10465	1	0	0	1	0	2
mmu-miR-223-3p	1E+08	Gm10143	1	0	0	1	0	2
mmu-miR-223-3p	1E+08	Gm2042	1	0	0	1	0	2
mmu-miR-223-3p	1E+08	Gm2046	1	0	0	1	0	2
mmu-miR-223-3p	1E+08	Cphx2	1	0	0	1	0	2
mmu-miR-223-3p	1E+08	Cphx3	1	0	0	1	0	2
mmu-miR-223-3p	1E+08	Gm10436	1	0	0	1	0	2
mmu-miR-223-3p	1E+08	Gm20815	1	0	0	1	0	2
mmu-miR-223-3p	1E+08	Ildr2	1	0	0	1	0	2
mmu-miR-223-3p	1E+08	Gm20819	1	0	0	1	0	2
mmu-miR-223-3p	1E+08	Olfr157	0	0	1	1	0	2
mmu-miR-223-3p	1E+08	2810416G20Rik	1	0	0	1	0	2
mmu-miR-223-3p	1E+08	Fancf	0	0	1	1	0	2
mmu-miR-223-3p	1E+08	Gm15023	0	1	0	1	0	2
mmu-miR-223-3p	1E+08	Tceal7	1	0	0	1	0	2
mmu-miR-223-3p	1E+08	Amd2	1	0	0	1	0	2
mmu-miR-223-3p	1E+08	Gm3604	1	0	0	1	0	2
mmu-miR-223-3p	1E+08	Gm3739	1	0	0	1	0	2
mmu-miR-223-3p	1E+08	Eif3j2	1	0	0	1	0	2
mmu-miR-223-3p	1E+08	Gm9769	1	0	0	1	0	2
mmu-miR-223-3p	1E+08	Gm4583	1	0	0	1	0	2
mmu-miR-223-3p	1E+08	Gm4636	1	0	0	1	0	2
mmu-miR-223-3p	1E+08	Gm4701	1	0	0	1	0	2
mmu-miR-223-3p	1E+08	Gm4718	1	0	0	1	0	2
mmu-miR-223-3p	1E+08	Gm21945	1	0	0	1	0	2
mmu-miR-223-3p	1E+08	Gm10778	1	0	0	1	0	2
mmu-miR-223-3p	1E+08	Sprr2a2	1	0	0	1	0	2
mmu-miR-223-3p	1E+08	Dynlt1a	1	0	0	1	0	2
mmu-miR-223-3p	1E+08	AA987161	1	0	0	1	0	2
mmu-miR-223-3p	1.01E+08	1700021K19Rik	0	0	1	1	0	2

mmu-miR-223-3p	1.01E+08	1700015G11Rik	1	0	0	1	0	2
mmu-miR-223-3p	1.01E+08	Pdzd7	1	0	0	1	0	2
mmu-miR-223-3p	1.01E+08	Klhl3	0	1	0	1	0	2
mmu-miR-223-3p	1.01E+08	Gm19638	1	0	0	1	0	2
mmu-miR-223-3p	1.01E+08	Gm14440	1	0	0	1	0	2
mmu-miR-223-3p	1.01E+08	Gm15867	1	0	0	1	0	2
mmu-miR-223-3p	1.01E+08	Dos	0	1	0	1	0	2
mmu-miR-223-3p	1.01E+08	Atg14	0	1	0	1	0	2
mmu-miR-223-3p	1.01E+08	LOC100504821	1	0	0	1	0	2
mmu-miR-223-3p	1.01E+08	LOC100505242	1	0	0	1	0	2
mmu-miR-223-3p	1.01E+08	LOC100861595	1	0	0	1	0	2
mmu-miR-223-3p	1.01E+08	Gm21064	1	0	0	1	0	2
mmu-miR-223-3p	1.01E+08	Gm21292	1	0	0	1	0	2
mmu-miR-223-3p	1.01E+08	Gm21936	1	0	0	1	0	2
mmu-miR-223-3p	1.01E+08	Gm21685	1	0	0	1	0	2
mmu-miR-223-3p	1.01E+08	LOC100862515	1	0	0	1	0	2
mmu-miR-223-3p	1.01E+08	LOC101055632	1	0	0	1	0	2
mmu-miR-223-3p	1.01E+08	LOC101055644	0	0	0	1	1	2
mmu-miR-223-3p	1.01E+08	LOC101055647	1	0	0	1	0	2
mmu-miR-223-3p	1.01E+08	LOC101055658	1	0	0	1	0	2
mmu-miR-223-3p	1.01E+08	LOC101055678	1	0	0	1	0	2
mmu-miR-223-3p	1.01E+08	LOC101055796	1	0	0	1	0	2
mmu-miR-223-3p	1.01E+08	LOC101055802	1	0	0	1	0	2
mmu-miR-223-3p	1.01E+08	LOC101055922	1	0	0	1	0	2
mmu-miR-223-3p	1.01E+08	LOC101055966	1	0	0	1	0	2
mmu-miR-223-3p	1.01E+08	LOC101055971	1	0	0	1	0	2
mmu-miR-223-3p	1.01E+08	LOC101055977	1	0	0	1	0	2
mmu-miR-223-3p	1.01E+08	LOC101056010	1	0	0	1	0	2
mmu-miR-223-3p	1.01E+08	LOC101056249	1	0	0	1	0	2
mmu-miR-223-3p	1.01E+08	LOC101056336	1	0	0	1	0	2
mmu-miR-223-3p	1.01E+08	LOC101056347	1	0	0	1	0	2
mmu-miR-223-3p	1.01E+08	LOC101056362	1	0	0	1	0	2
mmu-miR-223-3p	1.01E+08	LOC101056519	1	0	0	1	0	2
mmu-miR-223-3p	1.01E+08	LOC101056542	1	0	0	0	1	2
mmu-miR-223-3p	1.01E+08	LOC101056638	1	0	0	1	0	2
mmu-miR-223-3p	1.01E+08	LOC101056654	1	0	0	1	0	2
mmu-miR-223-3p	11305	Abca2	0	0	0	1	0	1
mmu-miR-223-3p	11363	Acadl	0	0	0	1	0	1
mmu-miR-223-3p	11364	Acadm	0	0	0	1	0	1
mmu-miR-223-3p	11419	Asic1	0	0	0	1	0	1
mmu-miR-223-3p	11421	Ace	0	0	0	1	0	1
mmu-miR-223-3p	11426	Macf1	0	0	0	1	0	1
mmu-miR-223-3p	11429	Aco2	0	0	0	1	0	1
mmu-miR-223-3p	11430	Acox1	0	0	0	1	0	1
mmu-miR-223-3p	11433	Acp5	0	0	0	1	0	1
mmu-miR-223-3p	11435	Chrna1	0	0	0	1	0	1
mmu-miR-223-3p	11441	Chrna7	1	0	0	0	0	1
mmu-miR-223-3p	11443	Chrnb1	0	0	0	1	0	1
mmu-miR-223-3p	11449	Chrng	0	0	0	1	0	1
mmu-miR-223-3p	11451	Acrv1	0	0	0	1	0	1
mmu-miR-223-3p	11459	Acta1	0	0	0	1	0	1
mmu-miR-223-3p	11465	Actg1	0	0	0	1	0	1
mmu-miR-223-3p	11470	Actl7a	0	0	0	1	0	1
mmu-miR-223-3p	11477	Acvr1	0	0	0	1	0	1
mmu-miR-223-3p	11486	Ada	0	0	0	1	0	1

mmu-miR-223-3p	11492	Adam19	0	0	0	1	0	1
mmu-miR-223-3p	11500	Adam7	0	0	0	1	0	1
mmu-miR-223-3p	11501	Adam8	0	0	0	1	0	1
mmu-miR-223-3p	11515	Adcy9	0	0	0	1	0	1
mmu-miR-223-3p	11518	Add1	0	0	0	1	0	1
mmu-miR-223-3p	11522	Adh1	0	0	0	1	0	1
mmu-miR-223-3p	11532	Adh5	0	0	0	1	0	1
mmu-miR-223-3p	11534	Adk	0	0	0	1	0	1
mmu-miR-223-3p	11535	Adm	0	0	0	1	0	1
mmu-miR-223-3p	11539	Adora1	0	0	0	1	0	1
mmu-miR-223-3p	11544	Adprh	0	0	0	1	0	1
mmu-miR-223-3p	11545	Parp1	0	0	0	1	0	1
mmu-miR-223-3p	11546	Parp2	0	0	0	1	0	1
mmu-miR-223-3p	11548	Adra1b	0	0	0	1	0	1
mmu-miR-223-3p	11553	Adra2c	0	0	1	0	0	1
mmu-miR-223-3p	11554	Adrb1	0	0	0	1	0	1
mmu-miR-223-3p	11555	Adrb2	0	0	0	1	0	1
mmu-miR-223-3p	11556	Adrb3	0	0	0	1	0	1
mmu-miR-223-3p	11565	Adssl1	0	0	1	0	0	1
mmu-miR-223-3p	11566	Adss	0	0	0	1	0	1
mmu-miR-223-3p	11567	Avil	0	0	0	1	0	1
mmu-miR-223-3p	11568	Aebp1	0	0	0	1	0	1
mmu-miR-223-3p	11572	Crisp3	0	0	0	1	0	1
mmu-miR-223-3p	11576	Afp	0	0	0	1	0	1
mmu-miR-223-3p	11606	Agt	0	0	0	1	0	1
mmu-miR-223-3p	11607	Agtr1a	0	0	0	1	0	1
mmu-miR-223-3p	11614	Nr0b1	0	0	0	1	0	1
mmu-miR-223-3p	11628	Aicda	0	0	0	1	0	1
mmu-miR-223-3p	11634	Aire	0	0	0	1	0	1
mmu-miR-223-3p	11636	Ak1	0	0	0	1	0	1
mmu-miR-223-3p	11640	Akap1	0	0	0	1	0	1
mmu-miR-223-3p	11643	Akap4	0	0	0	1	0	1
mmu-miR-223-3p	11647	Alpl	0	0	0	1	0	1
mmu-miR-223-3p	11650	Alppl2	0	0	0	1	0	1
mmu-miR-223-3p	11652	Akt2	0	0	0	1	0	1
mmu-miR-223-3p	11655	Alas1	0	0	0	1	0	1
mmu-miR-223-3p	11656	Alas2	0	0	0	1	0	1
mmu-miR-223-3p	11657	Alb	0	0	0	1	0	1
mmu-miR-223-3p	11669	Aldh2	0	0	0	1	0	1
mmu-miR-223-3p	11670	Aldh3a1	0	0	0	1	0	1
mmu-miR-223-3p	11676	Aldoc	0	0	0	1	0	1
mmu-miR-223-3p	11682	Alk	0	0	0	1	0	1
mmu-miR-223-3p	11684	Alox12	0	0	0	1	0	1
mmu-miR-223-3p	11727	Ang	0	0	0	1	0	1
mmu-miR-223-3p	11730	Ang3	0	0	0	1	0	1
mmu-miR-223-3p	11733	Ank1	0	0	0	1	0	1
mmu-miR-223-3p	11744	Anxa11	0	0	0	1	0	1
mmu-miR-223-3p	11745	Anxa3	0	0	0	1	0	1
mmu-miR-223-3p	11746	Anxa4	0	0	0	1	0	1
mmu-miR-223-3p	11747	Anxa5	0	0	0	1	0	1
mmu-miR-223-3p	11749	Anxa6	0	0	0	1	0	1
mmu-miR-223-3p	11761	Aox1	0	0	0	1	0	1
mmu-miR-223-3p	11768	Ap1m2	0	0	0	1	0	1
mmu-miR-223-3p	11770	Fabp4	0	0	0	1	0	1
mmu-miR-223-3p	11771	Ap2a1	0	1	0	0	0	1

mmu-miR-223-3p	11772	Ap2a2	0	0	0	1	0	1
mmu-miR-223-3p	11773	Ap2m1	0	0	0	1	0	1
mmu-miR-223-3p	11774	Ap3b1	0	0	0	1	0	1
mmu-miR-223-3p	11775	Ap3b2	0	0	0	1	0	1
mmu-miR-223-3p	11776	Ap3d1	0	0	0	1	0	1
mmu-miR-223-3p	11781	Ap4m1	1	0	0	0	0	1
mmu-miR-223-3p	11783	Apaf1	0	0	0	1	0	1
mmu-miR-223-3p	11785	Apbb1	0	0	0	1	0	1
mmu-miR-223-3p	11793	Atg5	0	0	0	1	0	1
mmu-miR-223-3p	11796	Birc3	0	0	0	1	0	1
mmu-miR-223-3p	11797	Birc2	0	0	0	1	0	1
mmu-miR-223-3p	11806	Apoa1	0	0	0	1	0	1
mmu-miR-223-3p	11807	Apoa2	0	0	0	1	0	1
mmu-miR-223-3p	11810	Apobec1	0	0	0	1	0	1
mmu-miR-223-3p	11811	Apobec2	0	0	0	1	0	1
mmu-miR-223-3p	11815	Apod	0	0	0	1	0	1
mmu-miR-223-3p	11820	App	0	0	0	1	0	1
mmu-miR-223-3p	11821	Aprt	0	0	0	1	0	1
mmu-miR-223-3p	11831	Aqp6	0	0	0	1	0	1
mmu-miR-223-3p	11832	Aqp7	0	0	0	1	0	1
mmu-miR-223-3p	11833	Aqp8	0	0	0	1	0	1
mmu-miR-223-3p	11834	Aqr	0	0	0	1	0	1
mmu-miR-223-3p	11835	Ar	0	0	0	1	0	1
mmu-miR-223-3p	11837	Rplp0	0	0	0	1	0	1
mmu-miR-223-3p	11839	Areg	0	0	0	1	0	1
mmu-miR-223-3p	11840	Arf1	0	0	0	1	0	1
mmu-miR-223-3p	11853	Rhoc	0	0	0	1	0	1
mmu-miR-223-3p	11854	Rhod	0	0	0	1	0	1
mmu-miR-223-3p	11855	Arhgap5	0	0	0	1	0	1
mmu-miR-223-3p	11861	Arl4a	0	0	0	1	0	1
mmu-miR-223-3p	11863	Arnt	0	0	0	1	0	1
mmu-miR-223-3p	11870	Art1	0	0	0	1	0	1
mmu-miR-223-3p	11889	Asgr1	0	0	0	1	0	1
mmu-miR-223-3p	11899	Astn1	0	0	0	1	0	1
mmu-miR-223-3p	11905	Serpinc1	1	0	0	0	0	1
mmu-miR-223-3p	11907	Ate1	0	0	0	1	0	1
mmu-miR-223-3p	11921	Atoh1	1	0	0	0	0	1
mmu-miR-223-3p	11922	Neurod6	0	0	0	1	0	1
mmu-miR-223-3p	11924	Neurog2	0	0	1	0	0	1
mmu-miR-223-3p	11925	Neurog3	0	0	0	1	0	1
mmu-miR-223-3p	11941	Atp2b2	0	0	0	1	0	1
mmu-miR-223-3p	11944	Atp4a	0	1	0	0	0	1
mmu-miR-223-3p	11946	Atp5a1	0	0	0	1	0	1
mmu-miR-223-3p	11949	Atp5c1	0	0	0	1	0	1
mmu-miR-223-3p	11973	Atp6v1e1	0	0	0	1	0	1
mmu-miR-223-3p	11974	Atp6v0e	1	0	0	0	0	1
mmu-miR-223-3p	11979	Atp7b	0	1	0	0	0	1
mmu-miR-223-3p	11991	Hnrnpd	0	0	0	1	0	1
mmu-miR-223-3p	12007	Azgp1	0	0	0	1	0	1
mmu-miR-223-3p	12009	Azi1	0	0	0	1	0	1
mmu-miR-223-3p	12010	B2m	0	0	0	1	0	1
mmu-miR-223-3p	12017	Bag1	0	0	0	1	0	1
mmu-miR-223-3p	12023	Barx2	0	0	0	1	0	1
mmu-miR-223-3p	12029	Bcl6b	0	0	0	1	0	1
mmu-miR-223-3p	12034	Phb2	0	0	0	1	0	1

mmu-miR-223-3p	12040	Bckdhb	0	0	0	1	0	1
mmu-miR-223-3p	12053	Bcl6	0	0	0	1	0	1
mmu-miR-223-3p	12061	Bdkrb1	0	0	0	1	0	1
mmu-miR-223-3p	12064	Bdnf	0	0	0	1	0	1
mmu-miR-223-3p	12091	Glb1	0	0	0	1	0	1
mmu-miR-223-3p	12111	Bgn	0	0	0	1	0	1
mmu-miR-223-3p	12116	Bhmt	0	0	0	1	0	1
mmu-miR-223-3p	12122	Bid	1	0	0	0	0	1
mmu-miR-223-3p	12125	Bcl2l11	0	0	0	1	0	1
mmu-miR-223-3p	12145	Cxcr5	0	0	0	1	0	1
mmu-miR-223-3p	12155	Bmp15	0	0	0	1	0	1
mmu-miR-223-3p	12160	Bmp5	0	1	0	0	0	1
mmu-miR-223-3p	12162	Bmp7	0	0	0	1	0	1
mmu-miR-223-3p	12163	Bmp8a	0	0	0	1	0	1
mmu-miR-223-3p	12164	Bmp8b	0	0	0	1	0	1
mmu-miR-223-3p	12175	Bnip2	0	0	0	1	0	1
mmu-miR-223-3p	12189	Brca1	0	0	0	1	0	1
mmu-miR-223-3p	12190	Brca2	0	0	0	1	0	1
mmu-miR-223-3p	12193	Zfp36l2	0	0	0	1	0	1
mmu-miR-223-3p	12229	Btk	0	0	0	1	0	1
mmu-miR-223-3p	12231	Btn1a1	0	0	0	1	0	1
mmu-miR-223-3p	12235	Bub1	0	0	0	1	0	1
mmu-miR-223-3p	12236	Bub1b	0	0	0	1	0	1
mmu-miR-223-3p	12238	Commd3	0	0	0	1	0	1
mmu-miR-223-3p	12257	Tspo	0	0	0	1	0	1
mmu-miR-223-3p	12259	C1qa	0	0	0	1	0	1
mmu-miR-223-3p	12263	C2	0	0	0	1	0	1
mmu-miR-223-3p	12266	C3	1	0	0	0	0	1
mmu-miR-223-3p	12269	C4bp	0	0	0	1	0	1
mmu-miR-223-3p	12273	C5ar1	0	0	0	1	0	1
mmu-miR-223-3p	12274	C6	0	0	0	1	0	1
mmu-miR-223-3p	12282	Hyou1	0	0	0	1	0	1
mmu-miR-223-3p	12286	Cacna1a	0	0	0	1	0	1
mmu-miR-223-3p	12287	Cacna1b	0	0	0	1	0	1
mmu-miR-223-3p	12289	Cacna1d	0	0	0	1	0	1
mmu-miR-223-3p	12295	Cacnb1	0	0	0	1	0	1
mmu-miR-223-3p	12297	Cacnb3	0	0	0	1	0	1
mmu-miR-223-3p	12304	Pdia4	0	0	0	1	0	1
mmu-miR-223-3p	12305	Ddr1	0	0	0	1	0	1
mmu-miR-223-3p	12308	Calb2	0	0	0	1	0	1
mmu-miR-223-3p	12309	S100g	0	0	0	1	0	1
mmu-miR-223-3p	12310	Calca	0	0	0	1	0	1
mmu-miR-223-3p	12315	Calm3	0	0	0	1	0	1
mmu-miR-223-3p	12317	Calr	0	0	0	1	0	1
mmu-miR-223-3p	12322	Camk2a	0	0	0	1	0	1
mmu-miR-223-3p	12330	Canx	0	0	0	1	0	1
mmu-miR-223-3p	12331	Cap1	0	0	1	0	0	1
mmu-miR-223-3p	12333	Capn1	0	0	0	1	0	1
mmu-miR-223-3p	12334	Capn2	0	0	0	1	0	1
mmu-miR-223-3p	12335	Capn3	0	0	0	1	0	1
mmu-miR-223-3p	12339	Capn7	0	0	0	1	0	1
mmu-miR-223-3p	12345	Capzb	0	0	0	1	0	1
mmu-miR-223-3p	12350	Car3	1	0	0	0	0	1
mmu-miR-223-3p	12353	Car6	0	0	0	1	0	1
mmu-miR-223-3p	12354	Car7	0	0	0	1	0	1

mmu-miR-223-3p	12355	Nr1i3	0	0	0	1	0	1
mmu-miR-223-3p	12359	Cat	0	0	0	1	0	1
mmu-miR-223-3p	12368	Casp6	0	0	0	1	0	1
mmu-miR-223-3p	12369	Casp7	0	0	0	1	0	1
mmu-miR-223-3p	12370	Casp8	0	0	0	1	0	1
mmu-miR-223-3p	12380	Cast	0	0	0	1	0	1
mmu-miR-223-3p	12385	Ctnna1	0	0	0	1	0	1
mmu-miR-223-3p	12402	Cbl	0	0	0	1	0	1
mmu-miR-223-3p	12406	Serpinh1	0	0	0	1	0	1
mmu-miR-223-3p	12409	Cbr2	0	0	0	1	0	1
mmu-miR-223-3p	12411	Cbs	0	0	0	1	0	1
mmu-miR-223-3p	12417	Cbx3	0	0	1	0	0	1
mmu-miR-223-3p	12421	Rb1cc1	0	0	1	0	0	1
mmu-miR-223-3p	12425	Cckar	0	0	0	1	0	1
mmu-miR-223-3p	12427	Ccna1	0	0	0	1	0	1
mmu-miR-223-3p	12442	Ccnb2	0	0	0	1	0	1
mmu-miR-223-3p	12445	Ccnd3	0	0	0	1	0	1
mmu-miR-223-3p	12447	Ccne1	0	0	0	1	0	1
mmu-miR-223-3p	12448	Ccne2	0	0	0	1	0	1
mmu-miR-223-3p	12450	Ccng1	0	0	0	1	0	1
mmu-miR-223-3p	12454	Ccnk	0	0	0	1	0	1
mmu-miR-223-3p	12455	Ccnt1	0	1	0	0	0	1
mmu-miR-223-3p	12464	Cct4	0	0	0	1	0	1
mmu-miR-223-3p	12468	Cct7	0	0	0	1	0	1
mmu-miR-223-3p	12476	Cd151	0	0	0	1	0	1
mmu-miR-223-3p	12479	Cd1d1	0	0	0	1	0	1
mmu-miR-223-3p	12482	Ms4a1	0	0	0	1	0	1
mmu-miR-223-3p	12483	Cd22	0	0	0	1	0	1
mmu-miR-223-3p	12491	Cd36	0	0	0	1	0	1
mmu-miR-223-3p	12492	Scarb2	0	0	0	1	0	1
mmu-miR-223-3p	12493	Cd37	0	0	0	1	0	1
mmu-miR-223-3p	12494	Cd38	0	0	0	1	0	1
mmu-miR-223-3p	12501	Cd3e	0	0	0	1	0	1
mmu-miR-223-3p	12505	Cd44	0	0	0	1	0	1
mmu-miR-223-3p	12506	Cd48	0	0	1	0	0	1
mmu-miR-223-3p	12507	Cd5	0	0	0	1	0	1
mmu-miR-223-3p	12509	Cd59a	0	0	0	1	0	1
mmu-miR-223-3p	12511	Cd6	0	0	0	1	0	1
mmu-miR-223-3p	12516	Cd7	0	0	0	1	0	1
mmu-miR-223-3p	12519	Cd80	0	0	0	1	0	1
mmu-miR-223-3p	12521	Cd82	0	0	0	1	0	1
mmu-miR-223-3p	12527	Cd9	0	0	0	1	0	1
mmu-miR-223-3p	12530	Cdc25a	0	0	0	1	0	1
mmu-miR-223-3p	12531	Cdc25b	0	0	0	1	0	1
mmu-miR-223-3p	12534	Cdk1	0	0	0	1	0	1
mmu-miR-223-3p	12537	Cdk11b	0	0	0	1	0	1
mmu-miR-223-3p	12550	Cdh1	1	0	0	0	0	1
mmu-miR-223-3p	12556	Cdh16	0	0	0	1	0	1
mmu-miR-223-3p	12557	Cdh17	0	0	0	1	0	1
mmu-miR-223-3p	12558	Cdh2	0	0	0	1	0	1
mmu-miR-223-3p	12571	Cdk6	0	0	0	1	0	1
mmu-miR-223-3p	12575	Cdkn1a	0	0	0	1	0	1
mmu-miR-223-3p	12577	Cdkn1c	0	0	0	1	0	1
mmu-miR-223-3p	12578	Cdkn2a	1	0	0	0	0	1
mmu-miR-223-3p	12579	Cdkn2b	1	0	0	0	0	1

mmu-miR-223-3p	12581	Cdkn2d	0	0	0	1	0	1
mmu-miR-223-3p	12583	Cdo1	1	0	0	0	0	1
mmu-miR-223-3p	12592	Cdx4	0	0	0	1	0	1
mmu-miR-223-3p	12607	Cebpz	0	0	0	1	0	1
mmu-miR-223-3p	12609	Cebpd	0	0	0	1	0	1
mmu-miR-223-3p	12615	Cenpa	0	0	0	1	0	1
mmu-miR-223-3p	12622	Cer1	0	0	0	1	0	1
mmu-miR-223-3p	12626	Cetn3	0	0	0	1	0	1
mmu-miR-223-3p	12630	Cfi	0	0	0	1	0	1
mmu-miR-223-3p	12640	Cga	0	0	0	1	0	1
mmu-miR-223-3p	12647	Chat	0	0	0	1	0	1
mmu-miR-223-3p	12649	Chek1	0	0	1	0	0	1
mmu-miR-223-3p	12652	Chga	0	0	0	1	0	1
mmu-miR-223-3p	12653	Chgb	0	0	0	1	0	1
mmu-miR-223-3p	12659	Ovgp1	0	0	0	1	0	1
mmu-miR-223-3p	12677	Vsx2	0	0	0	1	0	1
mmu-miR-223-3p	12695	Inadl	0	0	0	1	0	1
mmu-miR-223-3p	12696	Cirbp	0	0	0	1	0	1
mmu-miR-223-3p	12700	Cish	0	0	0	1	0	1
mmu-miR-223-3p	12702	Socs3	0	0	0	1	0	1
mmu-miR-223-3p	12709	Ckb	0	0	0	1	0	1
mmu-miR-223-3p	12715	Ckm	0	0	0	1	0	1
mmu-miR-223-3p	12733	Clcnka	0	0	0	1	0	1
mmu-miR-223-3p	12738	Cldn2	0	0	0	1	0	1
mmu-miR-223-3p	12750	Clk4	0	0	0	1	0	1
mmu-miR-223-3p	12752	Cln3	0	0	0	1	0	1
mmu-miR-223-3p	12757	Clta	0	0	0	1	0	1
mmu-miR-223-3p	12771	Ccr3	0	0	0	1	0	1
mmu-miR-223-3p	12772	Ccr2	0	0	0	1	0	1
mmu-miR-223-3p	12775	Ccr7	0	0	0	1	0	1
mmu-miR-223-3p	12778	Ackr3	0	0	0	1	0	1
mmu-miR-223-3p	12780	Abcc2	0	0	0	1	0	1
mmu-miR-223-3p	12789	Cnga2	0	0	0	1	0	1
mmu-miR-223-3p	12793	Cnih	0	0	0	1	0	1
mmu-miR-223-3p	12802	Cnr2	0	0	0	1	0	1
mmu-miR-223-3p	12804	Cntfr	0	0	0	1	0	1
mmu-miR-223-3p	12807	Hps3	0	0	0	1	0	1
mmu-miR-223-3p	12808	Cobl	0	0	0	1	0	1
mmu-miR-223-3p	12810	Coch	0	0	0	1	0	1
mmu-miR-223-3p	12812	Coil	0	0	0	1	0	1
mmu-miR-223-3p	12813	Col10a1	0	0	0	1	0	1
mmu-miR-223-3p	12816	Col12a1	0	0	0	1	0	1
mmu-miR-223-3p	12817	Col13a1	0	1	0	0	0	1
mmu-miR-223-3p	12818	Col14a1	0	0	0	1	0	1
mmu-miR-223-3p	12819	Col15a1	0	0	0	1	0	1
mmu-miR-223-3p	12821	Col17a1	0	0	0	1	0	1
mmu-miR-223-3p	12822	Col18a1	0	0	0	1	0	1
mmu-miR-223-3p	12824	Col2a1	0	0	0	1	0	1
mmu-miR-223-3p	12825	Col3a1	0	0	0	1	0	1
mmu-miR-223-3p	12830	Col4a5	0	0	0	1	0	1
mmu-miR-223-3p	12834	Col6a2	0	0	0	1	0	1
mmu-miR-223-3p	12836	Col7a1	0	0	0	1	0	1
mmu-miR-223-3p	12839	Col9a1	0	1	0	0	0	1
mmu-miR-223-3p	12840	Col9a2	0	0	0	1	0	1
mmu-miR-223-3p	12841	Col9a3	0	0	0	1	0	1

mmu-miR-223-3p	12850	Coq7	0	0	0	1	0	1
mmu-miR-223-3p	12856	Cox17	0	1	0	0	0	1
mmu-miR-223-3p	12859	Cox5b	0	0	0	1	0	1
mmu-miR-223-3p	12861	Cox6a1	0	0	0	1	0	1
mmu-miR-223-3p	12862	Cox6a2	0	0	0	1	0	1
mmu-miR-223-3p	12864	Cox6c	0	0	0	1	0	1
mmu-miR-223-3p	12865	Cox7a1	1	0	0	0	0	1
mmu-miR-223-3p	12866	Cox7a2	0	0	0	1	0	1
mmu-miR-223-3p	12868	Cox8a	0	0	0	1	0	1
mmu-miR-223-3p	12877	Cpeb1	0	0	0	1	0	1
mmu-miR-223-3p	12879	Cys1	0	0	0	1	0	1
mmu-miR-223-3p	12891	Cpne6	0	0	0	1	0	1
mmu-miR-223-3p	12896	Cpt2	0	0	0	1	0	1
mmu-miR-223-3p	12905	Cradd	0	0	0	1	0	1
mmu-miR-223-3p	12919	Crhbp	0	0	0	1	0	1
mmu-miR-223-3p	12927	Bcar1	0	0	0	1	0	1
mmu-miR-223-3p	12934	Dpysl2	0	0	0	1	0	1
mmu-miR-223-3p	12944	Crp	0	0	1	0	0	1
mmu-miR-223-3p	12945	Dmbt1	0	0	0	1	0	1
mmu-miR-223-3p	12952	Cry1	0	0	0	1	0	1
mmu-miR-223-3p	12955	Cryab	0	0	0	1	0	1
mmu-miR-223-3p	12959	Cryba4	0	0	0	1	0	1
mmu-miR-223-3p	12966	Crygc	0	0	0	1	0	1
mmu-miR-223-3p	12967	Crygd	0	0	0	1	0	1
mmu-miR-223-3p	12983	Csf2rb	0	0	0	1	0	1
mmu-miR-223-3p	12984	Csf2rb2	0	0	0	1	0	1
mmu-miR-223-3p	12988	Csk	0	0	0	1	0	1
mmu-miR-223-3p	12992	Csn1s2b	0	0	0	1	0	1
mmu-miR-223-3p	12993	Csn1s2a	0	0	0	1	0	1
mmu-miR-223-3p	13003	Vcan	0	0	0	1	0	1
mmu-miR-223-3p	13007	Csrp1	0	0	0	1	0	1
mmu-miR-223-3p	13008	Csrp2	1	0	0	0	0	1
mmu-miR-223-3p	13011	Cst7	0	0	0	1	0	1
mmu-miR-223-3p	13013	Cst9	0	0	0	1	0	1
mmu-miR-223-3p	13014	Cstb	1	0	0	0	0	1
mmu-miR-223-3p	13016	Ctbp1	0	0	0	1	0	1
mmu-miR-223-3p	13018	Ctcf	0	0	0	1	0	1
mmu-miR-223-3p	13026	Pcyt1a	0	0	0	1	0	1
mmu-miR-223-3p	13038	Ctsk	0	0	0	1	0	1
mmu-miR-223-3p	13056	Cyb561	1	0	0	0	0	1
mmu-miR-223-3p	13057	Cyba	0	0	0	1	0	1
mmu-miR-223-3p	13075	Cyp19a1	1	0	0	0	0	1
mmu-miR-223-3p	13076	Cyp1a1	0	0	0	1	0	1
mmu-miR-223-3p	13082	Cyp26a1	0	0	0	1	0	1
mmu-miR-223-3p	13094	Cyp2b9	0	0	0	1	0	1
mmu-miR-223-3p	13097	Cyp2c38	0	0	0	1	0	1
mmu-miR-223-3p	13098	Cyp2c39	0	0	0	1	0	1
mmu-miR-223-3p	13099	Cyp2c40	0	1	0	0	0	1
mmu-miR-223-3p	13106	Cyp2e1	0	0	0	1	0	1
mmu-miR-223-3p	13107	Cyp2f2	0	0	0	1	0	1
mmu-miR-223-3p	13108	Cyp2g1	0	0	0	1	0	1
mmu-miR-223-3p	13110	Cyp2j6	0	0	1	0	0	1
mmu-miR-223-3p	13112	Cyp3a11	0	0	0	1	0	1
mmu-miR-223-3p	13113	Cyp3a13	0	0	0	1	0	1
mmu-miR-223-3p	13115	Cyp27b1	0	0	0	1	0	1

mmu-miR-223-3p	13117	Cyp4a10	0	0	0	1	0	1
mmu-miR-223-3p	13118	Cyp4a12b	0	0	0	1	0	1
mmu-miR-223-3p	13122	Cyp7a1	0	0	0	1	0	1
mmu-miR-223-3p	13142	Dao	0	0	0	1	0	1
mmu-miR-223-3p	13144	Dapk3	0	0	0	1	0	1
mmu-miR-223-3p	13162	Slc6a3	0	0	0	1	0	1
mmu-miR-223-3p	13166	Dbh	0	0	0	1	0	1
mmu-miR-223-3p	13169	Dbnl	0	0	0	1	0	1
mmu-miR-223-3p	13172	Dbx1	0	0	0	1	0	1
mmu-miR-223-3p	13178	Dck	0	0	0	1	0	1
mmu-miR-223-3p	13179	Dcn	0	0	0	1	0	1
mmu-miR-223-3p	13180	Pcbd1	0	0	0	1	0	1
mmu-miR-223-3p	13184	Dcpp1	1	0	0	0	0	1
mmu-miR-223-3p	13206	Ddx4	0	0	0	1	0	1
mmu-miR-223-3p	13207	Ddx5	0	0	0	1	0	1
mmu-miR-223-3p	13215	Defb2	0	0	0	1	0	1
mmu-miR-223-3p	13216	Defa1	0	0	0	1	0	1
mmu-miR-223-3p	13222	Defa-rs2	0	0	0	1	0	1
mmu-miR-223-3p	13223	Defa-rs4	0	0	0	1	0	1
mmu-miR-223-3p	13226	Defa-rs7	0	0	0	1	0	1
mmu-miR-223-3p	13237	Defa3	0	0	0	1	0	1
mmu-miR-223-3p	13239	Defa5	0	0	0	1	0	1
mmu-miR-223-3p	13346	Des	0	0	0	1	0	1
mmu-miR-223-3p	13350	Dgat1	0	0	0	1	0	1
mmu-miR-223-3p	13353	Dgcr6	0	0	0	1	0	1
mmu-miR-223-3p	13367	Diap1	0	1	0	0	0	1
mmu-miR-223-3p	13382	Dld	0	0	0	1	0	1
mmu-miR-223-3p	13384	Mpp3	0	0	0	1	0	1
mmu-miR-223-3p	13386	Dlk1	0	0	0	1	0	1
mmu-miR-223-3p	13389	Dll3	0	0	0	1	0	1
mmu-miR-223-3p	13390	Dlx1	0	0	0	1	0	1
mmu-miR-223-3p	13393	Dlx3	0	0	0	1	0	1
mmu-miR-223-3p	13395	Dlx5	0	0	0	1	0	1
mmu-miR-223-3p	13401	Dmwd	0	0	0	1	0	1
mmu-miR-223-3p	13417	Dnah8	0	0	0	1	0	1
mmu-miR-223-3p	13424	Dync1h1	0	0	0	1	0	1
mmu-miR-223-3p	13430	Dnm2	0	0	0	1	0	1
mmu-miR-223-3p	13436	Dnmt3b	0	0	0	1	0	1
mmu-miR-223-3p	13437	Dnpep	0	0	0	1	0	1
mmu-miR-223-3p	13476	Reep5	0	0	0	1	0	1
mmu-miR-223-3p	13479	Dpep1	0	0	0	1	0	1
mmu-miR-223-3p	13486	Dr1	0	0	0	1	0	1
mmu-miR-223-3p	13491	Drd4	0	0	0	1	0	1
mmu-miR-223-3p	13505	Dsc1	0	0	0	1	0	1
mmu-miR-223-3p	13516	Epyc	0	0	0	1	0	1
mmu-miR-223-3p	13521	Slc26a2	0	0	0	1	0	1
mmu-miR-223-3p	13526	Adam24	0	0	0	1	0	1
mmu-miR-223-3p	13528	Dtnb	0	0	0	1	0	1
mmu-miR-223-3p	13529	Prl8a2	0	0	0	1	0	1
mmu-miR-223-3p	13542	Dvl1	0	0	0	1	0	1
mmu-miR-223-3p	13548	Dyrk1a	0	0	0	1	0	1
mmu-miR-223-3p	13559	E2f5	0	1	0	0	0	1
mmu-miR-223-3p	13599	Ecel1	0	0	0	1	0	1
mmu-miR-223-3p	13602	Sparcl1	0	0	0	1	0	1
mmu-miR-223-3p	13617	Ednra	1	0	0	0	0	1

mmu-miR-223-3p	13619	Phc1	0	0	0	1	0	1
mmu-miR-223-3p	13626	Eed	1	0	0	0	0	1
mmu-miR-223-3p	13627	Eef1a1	0	0	0	1	0	1
mmu-miR-223-3p	13629	Eef2	0	0	0	1	0	1
mmu-miR-223-3p	13639	Efna4	0	0	0	1	0	1
mmu-miR-223-3p	13641	Efnb1	0	0	1	0	0	1
mmu-miR-223-3p	13643	Efnb3	0	0	1	0	0	1
mmu-miR-223-3p	13645	Egf	0	0	0	1	0	1
mmu-miR-223-3p	13650	Rhbdf1	0	0	1	0	0	1
mmu-miR-223-3p	13655	Egr3	0	0	0	1	0	1
mmu-miR-223-3p	13664	Eif1a	0	0	0	1	0	1
mmu-miR-223-3p	13665	Eif2s1	0	0	0	1	0	1
mmu-miR-223-3p	13680	Ddx19a	0	0	0	1	0	1
mmu-miR-223-3p	13681	Eif4a1	0	0	0	1	0	1
mmu-miR-223-3p	13709	Elf1	0	0	0	1	0	1
mmu-miR-223-3p	13710	Elf3	0	0	0	1	0	1
mmu-miR-223-3p	13714	Elk4	0	0	0	1	0	1
mmu-miR-223-3p	13716	Ell	0	0	1	0	0	1
mmu-miR-223-3p	13717	Eln	0	0	0	1	0	1
mmu-miR-223-3p	13722	Aimp1	0	0	0	1	0	1
mmu-miR-223-3p	13723	Emb	0	0	0	1	0	1
mmu-miR-223-3p	13726	Emd	0	0	0	1	0	1
mmu-miR-223-3p	13730	Emp1	0	0	0	1	0	1
mmu-miR-223-3p	13731	Emp2	0	0	0	1	0	1
mmu-miR-223-3p	13733	Emr1	0	0	0	1	0	1
mmu-miR-223-3p	13796	Emx1	0	0	0	1	0	1
mmu-miR-223-3p	13799	En2	0	0	0	1	0	1
mmu-miR-223-3p	13800	Enah	0	0	0	1	0	1
mmu-miR-223-3p	13801	Enam	0	0	0	1	0	1
mmu-miR-223-3p	13805	Eng	0	0	0	1	0	1
mmu-miR-223-3p	13806	Eno1	0	0	0	1	0	1
mmu-miR-223-3p	13807	Eno2	0	0	0	1	0	1
mmu-miR-223-3p	13824	Epb4.1l4a	0	0	0	1	0	1
mmu-miR-223-3p	13828	Epb4.2	0	0	0	1	0	1
mmu-miR-223-3p	13835	Epha1	0	0	0	1	0	1
mmu-miR-223-3p	13837	Epha3	0	0	0	1	0	1
mmu-miR-223-3p	13846	Ephb4	0	0	0	1	0	1
mmu-miR-223-3p	13850	Ephx2	0	0	0	1	0	1
mmu-miR-223-3p	13852	Stx2	0	0	0	1	0	1
mmu-miR-223-3p	13854	Epn1	0	0	0	1	0	1
mmu-miR-223-3p	13859	Eps15l1	0	0	0	1	0	1
mmu-miR-223-3p	13861	Epx	0	0	0	1	0	1
mmu-miR-223-3p	13864	Nr2f6	0	0	0	1	0	1
mmu-miR-223-3p	13865	Nr2f1	0	0	0	1	0	1
mmu-miR-223-3p	13869	Erbb4	0	1	0	0	0	1
mmu-miR-223-3p	13875	Erf	1	0	0	0	0	1
mmu-miR-223-3p	13877	Erh	0	0	0	1	0	1
mmu-miR-223-3p	13885	Esd	0	0	0	1	0	1
mmu-miR-223-3p	13983	Esr2	0	0	0	1	0	1
mmu-miR-223-3p	13990	Smarcad1	0	0	0	1	0	1
mmu-miR-223-3p	13998	Fgd6	0	0	0	1	0	1
mmu-miR-223-3p	14004	Chchd2	0	0	0	1	0	1
mmu-miR-223-3p	14017	Evi2a	0	0	0	1	0	1
mmu-miR-223-3p	14029	Evx2	0	1	0	0	0	1
mmu-miR-223-3p	14030	Ewsr1	0	0	0	1	0	1

mmu-miR-223-3p	14038	Wfdc18	0	0	0	1	0	1
mmu-miR-223-3p	14043	Ext2	0	0	0	1	0	1
mmu-miR-223-3p	14049	Eya2	0	0	0	1	0	1
mmu-miR-223-3p	14058	F10	0	0	0	1	0	1
mmu-miR-223-3p	14065	F2rl3	0	0	0	1	0	1
mmu-miR-223-3p	14068	F7	0	0	0	1	0	1
mmu-miR-223-3p	14073	Faah	0	0	0	1	0	1
mmu-miR-223-3p	14079	Fabp2	0	0	0	1	0	1
mmu-miR-223-3p	14081	Acsl1	0	0	0	1	0	1
mmu-miR-223-3p	14087	Fanca	0	0	0	1	0	1
mmu-miR-223-3p	14103	Fasl	0	0	0	1	0	1
mmu-miR-223-3p	14118	Fbn1	0	0	0	1	0	1
mmu-miR-223-3p	14123	Fbrs	0	0	0	1	0	1
mmu-miR-223-3p	14125	Fcer1a	0	0	0	1	0	1
mmu-miR-223-3p	14129	Fcgr1	0	0	0	1	0	1
mmu-miR-223-3p	14130	Fcgr2b	0	0	0	1	0	1
mmu-miR-223-3p	14131	Fcgr3	0	0	0	1	0	1
mmu-miR-223-3p	14137	Fdft1	0	0	0	1	0	1
mmu-miR-223-3p	14148	Fdx1	0	0	0	1	0	1
mmu-miR-223-3p	14158	Fert2	0	0	0	1	0	1
mmu-miR-223-3p	14163	Fgd1	0	0	0	1	0	1
mmu-miR-223-3p	14167	Fgf12	0	0	0	1	0	1
mmu-miR-223-3p	14180	Fgf9	0	0	0	1	0	1
mmu-miR-223-3p	14186	Fgfr4	0	0	0	1	0	1
mmu-miR-223-3p	14187	Akr1b8	0	0	0	1	0	1
mmu-miR-223-3p	14208	Ppm1g	0	0	0	1	0	1
mmu-miR-223-3p	14218	Sh3pxd2a	0	0	0	1	0	1
mmu-miR-223-3p	14226	Fkbp1b	0	0	0	1	0	1
mmu-miR-223-3p	14234	Foxc2	1	0	0	0	0	1
mmu-miR-223-3p	14241	Foxl1	0	0	0	1	0	1
mmu-miR-223-3p	14245	Lpin1	0	0	0	1	0	1
mmu-miR-223-3p	14246	Flg	0	0	0	1	0	1
mmu-miR-223-3p	14251	Flot1	0	0	0	1	0	1
mmu-miR-223-3p	14252	Flot2	0	0	0	1	0	1
mmu-miR-223-3p	14257	Flt4	0	0	0	1	0	1
mmu-miR-223-3p	14263	Fmo5	0	0	0	1	0	1
mmu-miR-223-3p	14268	Fn1	0	0	0	1	0	1
mmu-miR-223-3p	14272	Fnta	1	0	0	0	0	1
mmu-miR-223-3p	14293	Fpr1	0	0	0	1	0	1
mmu-miR-223-3p	14309	Fshr	0	0	0	1	0	1
mmu-miR-223-3p	14311	Cidec	0	0	0	1	0	1
mmu-miR-223-3p	14312	Brd2	0	0	0	1	0	1
mmu-miR-223-3p	14319	Fth1	0	0	0	1	0	1
mmu-miR-223-3p	14343	Fut1	0	0	0	1	0	1
mmu-miR-223-3p	14357	Dtx1	0	0	0	1	0	1
mmu-miR-223-3p	14359	Fxr1	0	0	0	1	0	1
mmu-miR-223-3p	14375	Xrcc6	1	0	0	0	0	1
mmu-miR-223-3p	14376	Ganab	0	0	0	1	0	1
mmu-miR-223-3p	14381	G6pdx	0	0	0	1	0	1
mmu-miR-223-3p	14385	Slc37a4	0	0	0	1	0	1
mmu-miR-223-3p	14387	Gaa	0	0	0	1	0	1
mmu-miR-223-3p	14400	Gabrb1	0	0	0	1	0	1
mmu-miR-223-3p	14403	Gabrd	1	0	0	0	0	1
mmu-miR-223-3p	14407	Gabrg3	0	1	0	0	0	1
mmu-miR-223-3p	14409	Gabrr2	0	0	0	1	0	1

mmu-miR-223-3p	14421	B4galnt1	0	0	0	1	0	1
mmu-miR-223-3p	14430	Galt	0	0	0	1	0	1
mmu-miR-223-3p	14456	Gas6	0	0	0	1	0	1
mmu-miR-223-3p	14460	Gata1	0	0	0	1	0	1
mmu-miR-223-3p	14462	Gata3	0	0	0	1	0	1
mmu-miR-223-3p	14463	Gata4	0	0	0	1	0	1
mmu-miR-223-3p	14464	Gata5	0	0	0	1	0	1
mmu-miR-223-3p	14467	Gbas	0	0	0	1	0	1
mmu-miR-223-3p	14468	Gbp2b	0	0	0	1	0	1
mmu-miR-223-3p	14469	Gbp2	0	0	0	1	0	1
mmu-miR-223-3p	14473	Gc	0	0	0	1	0	1
mmu-miR-223-3p	14479	Usp15	0	0	0	1	0	1
mmu-miR-223-3p	14527	Gcgr	0	0	0	1	0	1
mmu-miR-223-3p	14531	Gcm1	0	0	0	1	0	1
mmu-miR-223-3p	14534	Kat2a	0	0	0	1	0	1
mmu-miR-223-3p	14544	Gda	0	0	0	1	0	1
mmu-miR-223-3p	14548	Mrps33	0	0	0	1	0	1
mmu-miR-223-3p	14559	Gdf1	0	0	0	1	0	1
mmu-miR-223-3p	14560	Gdf10	0	0	0	1	0	1
mmu-miR-223-3p	14561	Gdf11	0	1	0	0	0	1
mmu-miR-223-3p	14563	Gdf5	0	0	0	1	0	1
mmu-miR-223-3p	14566	Gdf9	0	0	0	1	0	1
mmu-miR-223-3p	14570	Arhgdig	0	0	0	1	0	1
mmu-miR-223-3p	14579	Gem	1	0	0	0	0	1
mmu-miR-223-3p	14580	Gfap	0	0	0	1	0	1
mmu-miR-223-3p	14581	Gfi1	0	0	0	1	0	1
mmu-miR-223-3p	14582	Gfi1b	0	0	0	1	0	1
mmu-miR-223-3p	14587	Gfra3	0	0	0	1	0	1
mmu-miR-223-3p	14588	Gfra4	0	0	0	1	0	1
mmu-miR-223-3p	14590	Ggh	0	1	0	0	0	1
mmu-miR-223-3p	14602	Ghrhr	1	0	0	0	0	1
mmu-miR-223-3p	14609	Gja1	0	0	0	1	0	1
mmu-miR-223-3p	14612	Gja4	0	0	0	1	0	1
mmu-miR-223-3p	14618	Gjb1	0	0	0	1	0	1
mmu-miR-223-3p	14620	Gjb3	0	0	0	1	0	1
mmu-miR-223-3p	14621	Gjb4	0	0	0	1	0	1
mmu-miR-223-3p	14622	Gjb5	0	0	0	1	0	1
mmu-miR-223-3p	14623	Gjb6	0	0	0	1	0	1
mmu-miR-223-3p	14625	Gykl1	0	0	0	1	0	1
mmu-miR-223-3p	14630	Gclm	0	0	0	1	0	1
mmu-miR-223-3p	14632	Gli1	1	0	0	0	0	1
mmu-miR-223-3p	14645	Glul	0	0	0	1	0	1
mmu-miR-223-3p	14651	Hagh	0	0	0	1	0	1
mmu-miR-223-3p	14659	Glrp1	0	0	0	1	0	1
mmu-miR-223-3p	14676	Gna15	0	0	0	1	0	1
mmu-miR-223-3p	14678	Gnai2	0	0	0	1	0	1
mmu-miR-223-3p	14679	Gnai3	0	0	0	1	0	1
mmu-miR-223-3p	14683	Gnas	0	0	0	1	0	1
mmu-miR-223-3p	14685	Gnat1	0	0	0	1	0	1
mmu-miR-223-3p	14686	Gnat2	0	0	0	1	0	1
mmu-miR-223-3p	14687	Gnaz	0	0	0	1	0	1
mmu-miR-223-3p	14688	Gnb1	0	0	0	1	0	1
mmu-miR-223-3p	14693	Gnb2	0	0	0	1	0	1
mmu-miR-223-3p	14702	Gng2	0	0	0	1	0	1
mmu-miR-223-3p	14707	Gng5	0	1	0	0	0	1

mmu-miR-223-3p	14714	Gnrh1	0	0	0	1	0	1
mmu-miR-223-3p	14726	Pdpn	0	0	0	1	0	1
mmu-miR-223-3p	14733	Gpc1	0	0	0	1	0	1
mmu-miR-223-3p	14735	Gpc4	0	0	0	1	0	1
mmu-miR-223-3p	14738	Gpr12	0	0	0	1	0	1
mmu-miR-223-3p	14744	Gpr65	0	0	0	1	0	1
mmu-miR-223-3p	14747	Cmklr1	0	0	1	0	0	1
mmu-miR-223-3p	14760	Gpr19	0	0	0	1	0	1
mmu-miR-223-3p	14765	Gpr50	0	1	0	0	0	1
mmu-miR-223-3p	14766	Gpr56	0	0	0	1	0	1
mmu-miR-223-3p	14775	Gpx1	0	0	0	1	0	1
mmu-miR-223-3p	14778	Gpx3	0	0	0	1	0	1
mmu-miR-223-3p	14780	Gpx5	0	0	0	1	0	1
mmu-miR-223-3p	14782	Gsr	0	0	0	1	0	1
mmu-miR-223-3p	14786	Grb7	0	0	0	1	0	1
mmu-miR-223-3p	14787	Rhpn1	0	0	0	1	0	1
mmu-miR-223-3p	14790	Grcc10	0	0	0	1	0	1
mmu-miR-223-3p	14794	Spsb2	0	0	0	1	0	1
mmu-miR-223-3p	14804	Grid2	0	1	0	0	0	1
mmu-miR-223-3p	14810	Grin1	0	0	0	1	0	1
mmu-miR-223-3p	14814	Grin2d	0	0	0	1	0	1
mmu-miR-223-3p	14824	Grn	1	0	0	0	0	1
mmu-miR-223-3p	14825	Cxcl1	0	0	0	1	0	1
mmu-miR-223-3p	14827	Pdia3	0	0	0	1	0	1
mmu-miR-223-3p	14828	Hspa5	0	0	0	1	0	1
mmu-miR-223-3p	14841	Gsg2	0	0	0	1	0	1
mmu-miR-223-3p	14843	Gsx2	0	0	0	1	0	1
mmu-miR-223-3p	14853	Gspt2	1	0	0	0	0	1
mmu-miR-223-3p	14854	Gss	0	0	0	1	0	1
mmu-miR-223-3p	14857	Gsta1	0	0	0	1	0	1
mmu-miR-223-3p	14858	Gsta2	0	0	0	1	0	1
mmu-miR-223-3p	14862	Gstm1	0	0	0	1	0	1
mmu-miR-223-3p	14863	Gstm2	0	0	0	1	0	1
mmu-miR-223-3p	14864	Gstm3	0	0	0	1	0	1
mmu-miR-223-3p	14865	Gstm4	0	0	0	1	0	1
mmu-miR-223-3p	14873	Gsto1	0	0	0	1	0	1
mmu-miR-223-3p	14874	Gstz1	0	0	0	1	0	1
mmu-miR-223-3p	14886	Gtf2i	0	0	0	1	0	1
mmu-miR-223-3p	14894	Gtl3	0	0	0	1	0	1
mmu-miR-223-3p	14912	Nkx6-2	0	0	0	1	0	1
mmu-miR-223-3p	14913	Guca1a	0	0	0	1	0	1
mmu-miR-223-3p	14915	Guca2a	1	0	0	0	0	1
mmu-miR-223-3p	14918	Gucy2d	0	0	0	1	0	1
mmu-miR-223-3p	14919	Gucy2e	0	0	0	1	0	1
mmu-miR-223-3p	14961	H2-Ab1	0	0	0	1	0	1
mmu-miR-223-3p	14962	Cfb	0	0	0	1	0	1
mmu-miR-223-3p	14964	H2-D1	0	0	0	1	0	1
mmu-miR-223-3p	14969	H2-Eb1	1	0	0	0	0	1
mmu-miR-223-3p	14972	H2-K1	0	0	0	1	0	1
mmu-miR-223-3p	14977	Slc39a7	0	0	0	1	0	1
mmu-miR-223-3p	14979	H2-Ke6	0	0	0	1	0	1
mmu-miR-223-3p	14990	H2-M2	0	0	0	1	0	1
mmu-miR-223-3p	14991	H2-M3	0	0	0	1	0	1
mmu-miR-223-3p	14999	H2-DMb1	0	0	0	1	0	1
mmu-miR-223-3p	15001	H2-Oa	0	0	0	1	0	1

mmu-miR-223-3p	15007	H2-Q10	0	0	0	1	0	1
mmu-miR-223-3p	15018	H2-Q7	0	0	0	1	0	1
mmu-miR-223-3p	15077	Hist2h3c1	0	0	0	1	0	1
mmu-miR-223-3p	15109	Hal	0	0	0	1	0	1
mmu-miR-223-3p	15115	Hars	0	0	0	1	0	1
mmu-miR-223-3p	15116	Has1	0	0	0	1	0	1
mmu-miR-223-3p	15132	Hbb-bh1	0	0	0	1	0	1
mmu-miR-223-3p	15139	Hc	0	0	0	1	0	1
mmu-miR-223-3p	15162	Hck	0	0	0	1	0	1
mmu-miR-223-3p	15163	Hcls1	1	0	0	0	0	1
mmu-miR-223-3p	15165	Hcn1	0	0	0	1	0	1
mmu-miR-223-3p	15168	Hcn3	0	0	0	1	0	1
mmu-miR-223-3p	15171	Hcrt	0	0	0	1	0	1
mmu-miR-223-3p	15182	Hdac2	0	0	0	1	0	1
mmu-miR-223-3p	15185	Hdac6	0	0	0	1	0	1
mmu-miR-223-3p	15192	Hdgfl1	0	0	0	1	0	1
mmu-miR-223-3p	15200	Hbegf	0	0	0	1	0	1
mmu-miR-223-3p	15203	Heph	0	0	0	1	0	1
mmu-miR-223-3p	15207	Hes3	0	0	0	1	0	1
mmu-miR-223-3p	15208	Hes5	0	0	0	1	0	1
mmu-miR-223-3p	15209	Hesx1	0	0	0	1	0	1
mmu-miR-223-3p	15211	Hexa	0	0	0	1	0	1
mmu-miR-223-3p	15212	Hexb	0	0	0	1	0	1
mmu-miR-223-3p	15213	Hey1	0	0	0	1	0	1
mmu-miR-223-3p	15216	Hfe	0	0	0	1	0	1
mmu-miR-223-3p	15220	Foxq1	0	0	0	1	0	1
mmu-miR-223-3p	15223	Foxj1	0	0	0	1	0	1
mmu-miR-223-3p	15228	Foxg1	0	0	0	1	0	1
mmu-miR-223-3p	15229	Foxd1	0	0	0	1	0	1
mmu-miR-223-3p	15239	Hgs	0	0	0	1	0	1
mmu-miR-223-3p	15248	Hic1	0	0	0	1	0	1
mmu-miR-223-3p	15254	Hint1	0	0	0	1	0	1
mmu-miR-223-3p	15258	Hipk2	0	1	0	0	0	1
mmu-miR-223-3p	15259	Hipk3	0	0	0	1	0	1
mmu-miR-223-3p	15270	H2afx	0	0	0	1	0	1
mmu-miR-223-3p	15273	Hivep2	0	0	0	1	0	1
mmu-miR-223-3p	15275	Hk1	0	0	0	1	0	1
mmu-miR-223-3p	15284	Hlx	0	0	0	1	0	1
mmu-miR-223-3p	15289	Hmgb1	0	0	0	1	0	1
mmu-miR-223-3p	15312	Hmgn1	0	0	0	1	0	1
mmu-miR-223-3p	15331	Hmgn2	0	0	0	1	0	1
mmu-miR-223-3p	15353	Hmg20b	0	0	0	1	0	1
mmu-miR-223-3p	15354	Hmgb3	0	0	0	1	0	1
mmu-miR-223-3p	15356	Hmgcl	0	0	0	1	0	1
mmu-miR-223-3p	15371	Hmx1	0	0	0	1	0	1
mmu-miR-223-3p	15372	Hmx2	1	0	0	0	0	1
mmu-miR-223-3p	15376	Foxa2	0	0	0	1	0	1
mmu-miR-223-3p	15377	Foxa3	0	0	0	1	0	1
mmu-miR-223-3p	15379	Onecut1	0	1	0	0	0	1
mmu-miR-223-3p	15382	Hnrnpa1	0	0	0	1	0	1
mmu-miR-223-3p	15384	Hnrnpab	0	0	0	1	0	1
mmu-miR-223-3p	15387	Hnrnpk	0	0	0	1	0	1
mmu-miR-223-3p	15388	Hnrnpl	0	0	0	1	0	1
mmu-miR-223-3p	15394	Hoxa1	0	0	0	1	0	1
mmu-miR-223-3p	15395	Hoxa10	0	0	0	1	0	1

mmu-miR-223-3p	15399	Hoxa2	0	0	0	1	0	1
mmu-miR-223-3p	15401	Hoxa4	0	0	0	1	0	1
mmu-miR-223-3p	15407	Hoxb1	0	0	0	1	0	1
mmu-miR-223-3p	15408	Hoxb13	0	0	0	1	0	1
mmu-miR-223-3p	15413	Hoxb5	0	0	0	1	0	1
mmu-miR-223-3p	15416	Hoxb8	0	0	0	1	0	1
mmu-miR-223-3p	15417	Hoxb9	0	0	0	1	0	1
mmu-miR-223-3p	15426	Hoxc8	0	0	0	1	0	1
mmu-miR-223-3p	15427	Hoxc9	0	0	0	1	0	1
mmu-miR-223-3p	15429	Hoxd1	0	0	0	1	0	1
mmu-miR-223-3p	15430	Hoxd10	0	0	0	1	0	1
mmu-miR-223-3p	15433	Hoxd13	0	0	0	1	0	1
mmu-miR-223-3p	15437	Hoxd8	0	0	0	1	0	1
mmu-miR-223-3p	15441	Hp1bp3	0	0	0	1	0	1
mmu-miR-223-3p	15442	Hpse	0	0	0	1	0	1
mmu-miR-223-3p	15444	Hpca	0	0	0	1	0	1
mmu-miR-223-3p	15446	Hpgd	0	0	0	1	0	1
mmu-miR-223-3p	15451	Hpn	0	0	0	1	0	1
mmu-miR-223-3p	15452	Hprt	0	0	0	1	0	1
mmu-miR-223-3p	15463	Agfg1	0	0	0	1	0	1
mmu-miR-223-3p	15466	Hrh2	0	0	1	0	0	1
mmu-miR-223-3p	15467	Eif2ak1	0	0	0	1	0	1
mmu-miR-223-3p	15468	Prmt2	0	0	0	1	0	1
mmu-miR-223-3p	15473	Hrsp12	0	0	0	1	0	1
mmu-miR-223-3p	15476	Hs3st1	0	0	0	1	0	1
mmu-miR-223-3p	15484	Hsd11b2	0	0	0	1	0	1
mmu-miR-223-3p	15485	Hsd17b1	0	0	0	1	0	1
mmu-miR-223-3p	15487	Hsd17b3	0	0	0	1	0	1
mmu-miR-223-3p	15492	Hsd3b1	1	0	0	0	0	1
mmu-miR-223-3p	15495	Hsd3b4	0	0	0	1	0	1
mmu-miR-223-3p	15496	Hsd3b5	0	0	0	1	0	1
mmu-miR-223-3p	15500	Hsf2	0	0	0	1	0	1
mmu-miR-223-3p	15505	Hsph1	0	0	0	1	0	1
mmu-miR-223-3p	15507	Hspb1	0	0	0	1	0	1
mmu-miR-223-3p	15510	Hspd1	0	0	0	1	0	1
mmu-miR-223-3p	15512	Hspa2	0	0	0	1	0	1
mmu-miR-223-3p	15519	Hsp90aa1	0	0	0	1	0	1
mmu-miR-223-3p	15547	Trmt2a	0	0	0	1	0	1
mmu-miR-223-3p	15557	Htr1f	0	0	0	1	0	1
mmu-miR-223-3p	15558	Htr2a	0	0	0	1	0	1
mmu-miR-223-3p	15561	Htr3a	0	0	0	1	0	1
mmu-miR-223-3p	15565	Htr6	1	0	0	0	0	1
mmu-miR-223-3p	15574	Hus1	0	0	0	1	0	1
mmu-miR-223-3p	15587	Hyal2	0	0	0	1	0	1
mmu-miR-223-3p	15894	Icam1	0	0	0	1	0	1
mmu-miR-223-3p	15896	Icam2	0	0	0	1	0	1
mmu-miR-223-3p	15901	Id1	0	0	0	1	0	1
mmu-miR-223-3p	15902	Id2	0	0	0	1	0	1
mmu-miR-223-3p	15903	Id3	0	0	0	1	0	1
mmu-miR-223-3p	15904	Id4	0	0	0	1	0	1
mmu-miR-223-3p	15926	Idh1	0	0	0	1	0	1
mmu-miR-223-3p	15936	Ier2	0	0	0	1	0	1
mmu-miR-223-3p	15937	Ier3	1	0	0	0	0	1
mmu-miR-223-3p	15939	Ier5	0	0	0	1	0	1
mmu-miR-223-3p	15953	Ifi47	0	0	0	1	0	1

mmu-miR-223-3p	15977	Ifnb1	0	0	0	1	0	1
mmu-miR-223-3p	15980	Ifngr2	0	0	0	1	0	1
mmu-miR-223-3p	15982	Ifrd1	0	0	0	1	0	1
mmu-miR-223-3p	15983	Ifrd2	0	0	0	1	0	1
mmu-miR-223-3p	16002	Igf2	0	0	0	1	0	1
mmu-miR-223-3p	16005	Igfals	0	0	0	1	0	1
mmu-miR-223-3p	16006	Igfbp1	0	0	0	1	0	1
mmu-miR-223-3p	16007	Cyr61	0	0	0	1	0	1
mmu-miR-223-3p	16009	Igfbp3	0	0	0	1	0	1
mmu-miR-223-3p	16010	Igfbp4	0	0	0	1	0	1
mmu-miR-223-3p	16149	Cd74	0	0	0	1	0	1
mmu-miR-223-3p	16154	Il10ra	0	0	0	1	0	1
mmu-miR-223-3p	16156	Il11	0	0	0	1	0	1
mmu-miR-223-3p	16158	Il11ra2	0	0	0	1	0	1
mmu-miR-223-3p	16160	Il12b	0	0	0	1	0	1
mmu-miR-223-3p	16161	Il12rb1	0	0	0	1	0	1
mmu-miR-223-3p	16162	Il12rb2	0	0	0	1	0	1
mmu-miR-223-3p	16169	Il15ra	0	0	0	1	0	1
mmu-miR-223-3p	16170	Il16	0	0	0	1	0	1
mmu-miR-223-3p	16172	Il17ra	0	0	0	1	0	1
mmu-miR-223-3p	16174	Il18rap	0	0	0	1	0	1
mmu-miR-223-3p	16175	Il1a	0	0	0	1	0	1
mmu-miR-223-3p	16176	Il1b	0	0	0	1	0	1
mmu-miR-223-3p	16186	Il2rg	0	0	0	1	0	1
mmu-miR-223-3p	16191	Il5	1	0	0	0	0	1
mmu-miR-223-3p	16199	Il9r	0	0	0	1	0	1
mmu-miR-223-3p	16201	Ilf3	0	0	0	1	0	1
mmu-miR-223-3p	16205	Gimap1	0	0	0	1	0	1
mmu-miR-223-3p	16325	Inhbc	0	0	0	1	0	1
mmu-miR-223-3p	16329	Inpp1	0	0	0	1	0	1
mmu-miR-223-3p	16332	Inppl1	0	0	0	1	0	1
mmu-miR-223-3p	16363	Irf2	0	0	0	1	0	1
mmu-miR-223-3p	16364	Irf4	0	0	0	1	0	1
mmu-miR-223-3p	16371	Irx1	1	0	0	0	0	1
mmu-miR-223-3p	16372	Irx2	0	0	0	1	0	1
mmu-miR-223-3p	16373	Irx3	0	0	0	1	0	1
mmu-miR-223-3p	16407	Itgae	0	0	0	1	0	1
mmu-miR-223-3p	16409	Itgam	0	0	0	1	0	1
mmu-miR-223-3p	16419	Itgb5	0	0	0	1	0	1
mmu-miR-223-3p	16424	Itih1	0	0	0	1	0	1
mmu-miR-223-3p	16426	Itih3	0	0	0	1	0	1
mmu-miR-223-3p	16427	Itih4	0	0	0	1	0	1
mmu-miR-223-3p	16428	Itk	0	0	0	1	0	1
mmu-miR-223-3p	16430	Stt3a	0	0	0	1	0	1
mmu-miR-223-3p	16434	Itpa	0	0	0	1	0	1
mmu-miR-223-3p	16447	Ivl	0	0	1	0	0	1
mmu-miR-223-3p	16450	Jag2	0	0	0	1	0	1
mmu-miR-223-3p	16452	Jak2	0	0	0	1	0	1
mmu-miR-223-3p	16453	Jak3	0	0	0	1	0	1
mmu-miR-223-3p	16475	Ajuba	0	0	0	1	0	1
mmu-miR-223-3p	16477	Junb	0	0	0	1	0	1
mmu-miR-223-3p	16478	Jund	0	0	0	1	0	1
mmu-miR-223-3p	16492	Kcna4	0	0	0	1	0	1
mmu-miR-223-3p	16504	Kcnc3	0	0	0	1	0	1
mmu-miR-223-3p	16506	Kcnd1	0	1	0	0	0	1

mmu-miR-223-3p	16519	Kcnj3	0	1	0	0	0	1
mmu-miR-223-3p	16521	Kcnj5	0	0	0	1	0	1
mmu-miR-223-3p	16529	Kcnk5	0	0	0	1	0	1
mmu-miR-223-3p	16533	Kcnmb1	0	0	0	1	0	1
mmu-miR-223-3p	16536	Kcnq2	0	0	0	1	0	1
mmu-miR-223-3p	16542	Kdr	0	0	0	1	0	1
mmu-miR-223-3p	16548	Khk	0	0	0	1	0	1
mmu-miR-223-3p	16551	Kif11	0	0	0	1	0	1
mmu-miR-223-3p	16552	Kif12	0	0	0	1	0	1
mmu-miR-223-3p	16559	Kif17	0	0	0	1	0	1
mmu-miR-223-3p	16563	Kif2a	0	0	0	1	0	1
mmu-miR-223-3p	16564	Kif21a	0	0	0	1	0	1
mmu-miR-223-3p	16573	Kif5b	0	0	0	1	0	1
mmu-miR-223-3p	16578	Kif9	0	0	0	1	0	1
mmu-miR-223-3p	16579	Kifap3	0	0	0	1	0	1
mmu-miR-223-3p	16581	Kifc2	0	0	0	1	0	1
mmu-miR-223-3p	16582	Kifc3	0	0	0	1	0	1
mmu-miR-223-3p	16588	Kin	0	0	0	1	0	1
mmu-miR-223-3p	16592	Fabp5	0	0	0	1	0	1
mmu-miR-223-3p	16594	Klc2	0	0	0	1	0	1
mmu-miR-223-3p	16599	Klf3	0	0	0	1	0	1
mmu-miR-223-3p	16600	Klf4	0	0	0	1	0	1
mmu-miR-223-3p	16621	Klkb1	0	0	0	1	0	1
mmu-miR-223-3p	16625	Serpina3c	0	0	0	1	0	1
mmu-miR-223-3p	16633	Klra2	0	0	0	1	0	1
mmu-miR-223-3p	16636	Klra5	1	0	0	0	0	1
mmu-miR-223-3p	16639	Klra8	1	0	0	0	0	1
mmu-miR-223-3p	16640	Klra9	1	0	0	0	0	1
mmu-miR-223-3p	16644	Kng1	0	0	0	1	0	1
mmu-miR-223-3p	16656	Hivep3	0	1	0	0	0	1
mmu-miR-223-3p	16661	Krt10	0	0	0	1	0	1
mmu-miR-223-3p	16666	Krt16	0	0	0	1	0	1
mmu-miR-223-3p	16667	Krt17	0	0	0	1	0	1
mmu-miR-223-3p	16670	Krt32	0	0	0	1	0	1
mmu-miR-223-3p	16672	Krt34	0	0	0	1	0	1
mmu-miR-223-3p	16678	Krt1	0	0	0	1	0	1
mmu-miR-223-3p	16679	Krt86	0	0	0	1	0	1
mmu-miR-223-3p	16680	Krt84	0	0	0	1	0	1
mmu-miR-223-3p	16687	Krt6a	0	0	0	1	0	1
mmu-miR-223-3p	16691	Krt8	0	0	0	1	0	1
mmu-miR-223-3p	16693	Krtap11-1	0	0	0	1	0	1
mmu-miR-223-3p	16694	Krtap12-1	0	0	0	1	0	1
mmu-miR-223-3p	16706	Ksr1	0	0	0	1	0	1
mmu-miR-223-3p	16763	Lad1	0	0	0	1	0	1
mmu-miR-223-3p	16770	Lalba	0	0	0	1	0	1
mmu-miR-223-3p	16777	Lamb1	0	0	0	1	0	1
mmu-miR-223-3p	16780	Lamb3	0	0	0	1	0	1
mmu-miR-223-3p	16784	Lamp2	0	0	0	1	0	1
mmu-miR-223-3p	16790	Anpep	0	0	0	1	0	1
mmu-miR-223-3p	16795	Large	0	0	0	1	0	1
mmu-miR-223-3p	16800	Arhgef2	0	0	0	1	0	1
mmu-miR-223-3p	16801	Arhgef1	0	0	0	1	0	1
mmu-miR-223-3p	16818	Lck	0	0	0	1	0	1
mmu-miR-223-3p	16819	Lcn2	0	0	0	1	0	1
mmu-miR-223-3p	16822	Lcp2	0	0	0	1	0	1

mmu-miR-223-3p	16826	Ldb2	0	0	0	1	0	1
mmu-miR-223-3p	16854	Lgals3	0	0	0	1	0	1
mmu-miR-223-3p	16865	Eif2d	0	0	0	1	0	1
mmu-miR-223-3p	16869	Lhx1	0	1	0	0	0	1
mmu-miR-223-3p	16870	Lhx2	0	0	0	1	0	1
mmu-miR-223-3p	16873	Lhx5	0	0	0	1	0	1
mmu-miR-223-3p	16875	Lhx8	0	1	0	0	0	1
mmu-miR-223-3p	16881	Lig1	0	0	0	1	0	1
mmu-miR-223-3p	16882	Lig3	0	0	0	1	0	1
mmu-miR-223-3p	16885	Limk1	0	0	1	0	0	1
mmu-miR-223-3p	16897	Llgl1	0	0	0	1	0	1
mmu-miR-223-3p	16898	Rps2	0	0	0	1	0	1
mmu-miR-223-3p	16905	Lmna	0	0	0	1	0	1
mmu-miR-223-3p	16906	Lmnb1	0	0	0	1	0	1
mmu-miR-223-3p	16911	Lmo4	0	0	0	1	0	1
mmu-miR-223-3p	16913	Psmb8	0	0	0	1	0	1
mmu-miR-223-3p	16918	Mycl	0	0	0	1	0	1
mmu-miR-223-3p	16949	Loxl1	0	0	0	1	0	1
mmu-miR-223-3p	16971	Lrp1	0	0	0	1	0	1
mmu-miR-223-3p	16973	Lrp5	0	1	0	0	0	1
mmu-miR-223-3p	16976	Lrpap1	1	0	0	0	0	1
mmu-miR-223-3p	16979	Lrrn1	0	0	0	1	0	1
mmu-miR-223-3p	16980	Lrrn2	0	0	0	1	0	1
mmu-miR-223-3p	16985	Lsp1	0	0	0	1	0	1
mmu-miR-223-3p	16992	Lta	0	0	0	1	0	1
mmu-miR-223-3p	16993	Lta4h	0	0	0	1	0	1
mmu-miR-223-3p	16997	Ltbp2	0	0	0	1	0	1
mmu-miR-223-3p	16998	Ltbp3	0	0	0	1	0	1
mmu-miR-223-3p	17000	Ltbr	0	0	0	1	0	1
mmu-miR-223-3p	17002	Ltf	0	0	0	1	0	1
mmu-miR-223-3p	17022	Lum	0	0	0	1	0	1
mmu-miR-223-3p	17063	Muc13	0	0	0	1	0	1
mmu-miR-223-3p	17067	Ly6c1	0	0	0	1	0	1
mmu-miR-223-3p	17068	Ly6d	0	0	0	1	0	1
mmu-miR-223-3p	17084	Ly86	0	0	0	1	0	1
mmu-miR-223-3p	17085	Ly9	0	0	0	1	0	1
mmu-miR-223-3p	17089	Lyar	0	0	0	1	0	1
mmu-miR-223-3p	17095	Lyl1	0	0	0	1	0	1
mmu-miR-223-3p	17096	Lyn	0	0	0	1	0	1
mmu-miR-223-3p	17105	Lyz2	0	0	0	1	0	1
mmu-miR-223-3p	17110	Lyz1	0	0	0	1	0	1
mmu-miR-223-3p	17113	M6pr	0	0	0	1	0	1
mmu-miR-223-3p	17117	Amacr	0	0	0	1	0	1
mmu-miR-223-3p	17121	Mxd3	0	0	0	1	0	1
mmu-miR-223-3p	17123	Madcam1	0	0	0	1	0	1
mmu-miR-223-3p	17128	Smad4	1	0	0	0	0	1
mmu-miR-223-3p	17131	Smad7	0	0	0	1	0	1
mmu-miR-223-3p	17137	Magea1	0	0	0	1	0	1
mmu-miR-223-3p	17139	Magea3	0	0	0	1	0	1
mmu-miR-223-3p	17141	Magea5	0	0	0	1	0	1
mmu-miR-223-3p	17142	Magea6	0	0	0	1	0	1
mmu-miR-223-3p	17144	Magea8	0	0	0	1	0	1
mmu-miR-223-3p	17147	Mageb3	1	0	0	0	0	1
mmu-miR-223-3p	17150	Mfap2	0	0	0	1	0	1
mmu-miR-223-3p	17151	Ccndbp1	0	0	0	1	0	1

mmu-miR-223-3p	17153	Mal	0	0	0	1	0	1
mmu-miR-223-3p	17155	Man1a	0	0	0	1	0	1
mmu-miR-223-3p	17158	Man2a1	0	0	0	1	0	1
mmu-miR-223-3p	17160	Man2b2	0	0	0	1	0	1
mmu-miR-223-3p	17169	Mark3	0	0	0	1	0	1
mmu-miR-223-3p	17171	Mas1	0	0	0	1	0	1
mmu-miR-223-3p	17172	Ascl1	0	0	0	1	0	1
mmu-miR-223-3p	17178	Fxyd3	0	0	0	1	0	1
mmu-miR-223-3p	17183	Matn4	0	0	0	1	0	1
mmu-miR-223-3p	17184	Matr3	0	0	0	1	0	1
mmu-miR-223-3p	17187	Max	0	0	0	1	0	1
mmu-miR-223-3p	17193	Mbd4	0	0	0	1	0	1
mmu-miR-223-3p	17195	Mbl2	0	0	0	1	0	1
mmu-miR-223-3p	17201	Mc3r	0	0	0	1	0	1
mmu-miR-223-3p	17218	Mcm5	0	0	0	1	0	1
mmu-miR-223-3p	17235	Smcp	0	1	0	0	0	1
mmu-miR-223-3p	17248	Mdm4	0	0	0	1	0	1
mmu-miR-223-3p	17250	Abcc1	0	0	0	1	0	1
mmu-miR-223-3p	17279	Melk	0	0	0	1	0	1
mmu-miR-223-3p	17281	Fyco1	0	0	0	1	0	1
mmu-miR-223-3p	17285	Meox1	0	0	0	1	0	1
mmu-miR-223-3p	17289	Mertk	0	0	0	1	0	1
mmu-miR-223-3p	17292	Mesp1	0	0	0	1	0	1
mmu-miR-223-3p	17293	Mesp2	0	0	0	1	0	1
mmu-miR-223-3p	17300	Foxc1	0	0	0	1	0	1
mmu-miR-223-3p	17301	Foxd2	0	0	0	1	0	1
mmu-miR-223-3p	17304	Mfge8	0	0	0	1	0	1
mmu-miR-223-3p	17305	Mfng	0	0	0	1	0	1
mmu-miR-223-3p	17308	Mgat1	0	0	0	1	0	1
mmu-miR-223-3p	17312	Clec10a	0	0	0	1	0	1
mmu-miR-223-3p	17313	Mgp	0	0	0	1	0	1
mmu-miR-223-3p	17318	Mid1	0	0	0	1	0	1
mmu-miR-223-3p	17329	Cxcl9	0	0	0	1	0	1
mmu-miR-223-3p	17349	Mlf1	0	0	0	1	0	1
mmu-miR-223-3p	17350	Mlh1	0	0	0	1	0	1
mmu-miR-223-3p	17356	Mllt4	0	1	0	0	0	1
mmu-miR-223-3p	17386	Mmp13	0	0	0	1	0	1
mmu-miR-223-3p	17387	Mmp14	0	0	0	1	0	1
mmu-miR-223-3p	17388	Mmp15	0	0	0	1	0	1
mmu-miR-223-3p	17394	Mmp8	0	0	0	1	0	1
mmu-miR-223-3p	17420	Mnat1	0	0	0	1	0	1
mmu-miR-223-3p	17425	Foxk1	0	0	0	1	0	1
mmu-miR-223-3p	17434	Mocs2	0	0	0	1	0	1
mmu-miR-223-3p	17441	Mog	0	0	0	1	0	1
mmu-miR-223-3p	17448	Mdh2	0	0	0	1	0	1
mmu-miR-223-3p	17463	Psmd7	0	0	0	1	0	1
mmu-miR-223-3p	17474	Clec4d	0	0	0	1	0	1
mmu-miR-223-3p	17480	Mpl	0	0	0	1	0	1
mmu-miR-223-3p	17523	Mpo	0	0	0	1	0	1
mmu-miR-223-3p	17534	Mrc2	0	0	0	1	0	1
mmu-miR-223-3p	17540	Mrvi1	0	0	0	1	0	1
mmu-miR-223-3p	17700	Mstn	0	0	0	1	0	1
mmu-miR-223-3p	17701	Msx1	0	0	0	1	0	1
mmu-miR-223-3p	17702	Msx2	0	0	0	1	0	1
mmu-miR-223-3p	17703	Msx3	0	0	0	1	0	1

mmu-miR-223-3p	17749	Polr2k	0	0	0	1	0	1
mmu-miR-223-3p	17768	Mthfd2	0	0	0	1	0	1
mmu-miR-223-3p	17771	Mtl5	1	0	0	0	0	1
mmu-miR-223-3p	17773	Mtnr1a	0	0	0	1	0	1
mmu-miR-223-3p	17777	Mttp	0	0	0	1	0	1
mmu-miR-223-3p	17826	Fam89b	0	0	0	1	0	1
mmu-miR-223-3p	17829	Muc1	0	0	0	1	0	1
mmu-miR-223-3p	17831	Muc2	0	0	0	1	0	1
mmu-miR-223-3p	17840	Mup1	0	0	0	1	0	1
mmu-miR-223-3p	17841	Mup2	0	0	0	1	0	1
mmu-miR-223-3p	17842	Mup3	0	0	0	1	0	1
mmu-miR-223-3p	17850	Mut	0	0	0	1	0	1
mmu-miR-223-3p	17859	Mxi1	0	0	0	1	0	1
mmu-miR-223-3p	17863	Myb	0	0	0	1	0	1
mmu-miR-223-3p	17865	Mybl2	0	0	0	1	0	1
mmu-miR-223-3p	17870	Mycs	0	0	0	1	0	1
mmu-miR-223-3p	17874	Myd88	0	0	0	1	0	1
mmu-miR-223-3p	17876	Myef2	0	0	0	1	0	1
mmu-miR-223-3p	17878	Myf6	0	0	0	1	0	1
mmu-miR-223-3p	17883	Myh3	0	1	0	0	0	1
mmu-miR-223-3p	17885	Myh8	0	0	0	1	0	1
mmu-miR-223-3p	17907	Mylpf	0	0	0	1	0	1
mmu-miR-223-3p	17910	Myo15	0	0	0	1	0	1
mmu-miR-223-3p	17912	Myo1b	0	0	0	1	0	1
mmu-miR-223-3p	17919	Myo5b	1	0	0	0	0	1
mmu-miR-223-3p	17922	Myo7b	0	0	0	1	0	1
mmu-miR-223-3p	17927	Myod1	0	0	0	1	0	1
mmu-miR-223-3p	17929	Myom1	0	0	0	1	0	1
mmu-miR-223-3p	17930	Myom2	0	0	0	1	0	1
mmu-miR-223-3p	17931	Ppp1r12a	0	0	0	1	0	1
mmu-miR-223-3p	17932	Myt1	0	0	0	1	0	1
mmu-miR-223-3p	17939	Naga	0	0	0	1	0	1
mmu-miR-223-3p	17948	Naip2	0	0	0	1	0	1
mmu-miR-223-3p	17954	Nap1l2	0	0	1	0	0	1
mmu-miR-223-3p	17962	Nat3	0	0	0	1	0	1
mmu-miR-223-3p	17972	Ncf4	0	0	0	1	0	1
mmu-miR-223-3p	17978	Ncoa2	0	0	0	1	0	1
mmu-miR-223-3p	17984	Ndn	0	0	1	0	0	1
mmu-miR-223-3p	17992	Ndufa4	0	0	0	1	0	1
mmu-miR-223-3p	17993	Ndufs4	0	0	0	1	0	1
mmu-miR-223-3p	17997	Nedd1	0	0	0	1	0	1
mmu-miR-223-3p	18004	Nek1	0	1	0	0	0	1
mmu-miR-223-3p	18018	Nfatc1	0	0	0	1	0	1
mmu-miR-223-3p	18023	Nfe2l1	0	0	0	1	0	1
mmu-miR-223-3p	18033	Nfkb1	0	0	0	1	0	1
mmu-miR-223-3p	18035	Nfkbia	0	0	0	1	0	1
mmu-miR-223-3p	18036	Nfkbib	0	0	0	1	0	1
mmu-miR-223-3p	18046	Nfyc	0	0	0	1	0	1
mmu-miR-223-3p	18054	Ngp	0	0	0	1	0	1
mmu-miR-223-3p	18082	Nipsnap1	0	0	0	1	0	1
mmu-miR-223-3p	18089	Nkx2-3	0	0	0	1	0	1
mmu-miR-223-3p	18091	Nkx2-5	0	0	0	1	0	1
mmu-miR-223-3p	18092	Nkx2-6	0	0	0	1	0	1
mmu-miR-223-3p	18096	Nkx6-1	0	1	0	0	0	1
mmu-miR-223-3p	18099	Nlk	0	0	0	1	0	1

mmu-miR-223-3p	18100	Mrpl40	0	0	0	1	0	1
mmu-miR-223-3p	18102	Nme1	0	0	0	1	0	1
mmu-miR-223-3p	18108	Nmt2	0	0	0	1	0	1
mmu-miR-223-3p	18113	Nnmt	0	0	0	1	0	1
mmu-miR-223-3p	18114	Rrp1	0	0	0	1	0	1
mmu-miR-223-3p	18121	Nog	0	0	0	1	0	1
mmu-miR-223-3p	18125	Nos1	0	1	0	0	0	1
mmu-miR-223-3p	18127	Nos3	0	0	0	1	0	1
mmu-miR-223-3p	18130	Ints6	1	0	0	0	0	1
mmu-miR-223-3p	18132	Notch4	0	0	0	1	0	1
mmu-miR-223-3p	18143	Npas2	0	0	0	1	0	1
mmu-miR-223-3p	18146	Npdc1	1	0	0	0	0	1
mmu-miR-223-3p	18148	Npm1	0	0	0	1	0	1
mmu-miR-223-3p	18150	Npm3	0	0	0	1	0	1
mmu-miR-223-3p	18158	Nppb	1	0	0	0	0	1
mmu-miR-223-3p	18160	Npr1	0	0	0	1	0	1
mmu-miR-223-3p	18162	Npr3	0	0	0	1	0	1
mmu-miR-223-3p	18166	Npy1r	0	1	0	0	0	1
mmu-miR-223-3p	18168	Npy5r	0	0	0	1	0	1
mmu-miR-223-3p	18171	Nr1i2	0	0	0	1	0	1
mmu-miR-223-3p	18173	Slc11a1	0	0	0	1	0	1
mmu-miR-223-3p	18175	Nrap	0	0	0	1	0	1
mmu-miR-223-3p	18196	Nsg1	0	0	0	1	0	1
mmu-miR-223-3p	18198	Musk	0	0	0	1	0	1
mmu-miR-223-3p	18201	Nsmaf	0	0	0	1	0	1
mmu-miR-223-3p	18203	Ntan1	0	0	0	1	0	1
mmu-miR-223-3p	18205	Ntf3	0	0	0	1	0	1
mmu-miR-223-3p	18209	Ntn3	0	0	0	1	0	1
mmu-miR-223-3p	18217	Ntsr2	0	0	1	0	0	1
mmu-miR-223-3p	18220	Nucb1	0	0	0	1	0	1
mmu-miR-223-3p	18223	Numbl	0	0	0	1	0	1
mmu-miR-223-3p	18242	Oat	0	0	0	1	0	1
mmu-miR-223-3p	18245	Oaz1	0	0	0	1	0	1
mmu-miR-223-3p	18263	Odc1	0	0	0	1	0	1
mmu-miR-223-3p	18291	Nobox	0	0	0	1	0	1
mmu-miR-223-3p	18302	Oit3	0	0	0	1	0	1
mmu-miR-223-3p	18323	Olfr25	0	0	0	1	0	1
mmu-miR-223-3p	18356	Olfr56	0	0	0	1	0	1
mmu-miR-223-3p	18377	Omg	1	0	0	0	0	1
mmu-miR-223-3p	18389	Oprl1	0	0	0	1	0	1
mmu-miR-223-3p	18390	Oprm1	0	0	0	1	0	1
mmu-miR-223-3p	18392	Orc1	0	0	0	1	0	1
mmu-miR-223-3p	18393	Orc2	0	0	0	1	0	1
mmu-miR-223-3p	18405	Orm1	0	0	0	1	0	1
mmu-miR-223-3p	18406	Orm2	0	0	0	1	0	1
mmu-miR-223-3p	18407	Orm3	0	0	0	1	0	1
mmu-miR-223-3p	18416	Otc	0	0	0	1	0	1
mmu-miR-223-3p	18419	Otog	0	0	0	1	0	1
mmu-miR-223-3p	18422	Ott	0	0	0	1	0	1
mmu-miR-223-3p	18438	P2rx4	0	0	0	1	0	1
mmu-miR-223-3p	18440	P2rx6	0	0	0	1	0	1
mmu-miR-223-3p	18452	P4ha2	0	1	0	0	0	1
mmu-miR-223-3p	18453	P4hb	0	0	0	1	0	1
mmu-miR-223-3p	18477	Prdx1	0	0	0	1	0	1
mmu-miR-223-3p	18478	Pah	0	0	0	1	0	1

mmu-miR-223-3p	18484	Pam	0	0	0	1	0	1
mmu-miR-223-3p	18488	Cntn3	0	0	0	1	0	1
mmu-miR-223-3p	18503	Pax1	1	0	0	0	0	1
mmu-miR-223-3p	18507	Pax5	0	1	0	0	0	1
mmu-miR-223-3p	18515	Pbx2	1	0	0	0	0	1
mmu-miR-223-3p	18518	Igbp1	0	0	0	1	0	1
mmu-miR-223-3p	18521	Pcbp2	0	0	0	1	0	1
mmu-miR-223-3p	18530	Pcdh8	0	0	0	1	0	1
mmu-miR-223-3p	18534	Pck1	0	0	0	1	0	1
mmu-miR-223-3p	18538	Pcna	0	0	0	1	0	1
mmu-miR-223-3p	18541	Pcnt	0	0	0	1	0	1
mmu-miR-223-3p	18545	Pcp2	0	0	0	1	0	1
mmu-miR-223-3p	18548	Pcsk1	1	0	0	0	0	1
mmu-miR-223-3p	18551	Pcsk4	0	0	0	1	0	1
mmu-miR-223-3p	18554	Pcsk7	0	0	0	1	0	1
mmu-miR-223-3p	18559	Pctp	0	0	0	1	0	1
mmu-miR-223-3p	18563	Pcx	0	0	0	1	0	1
mmu-miR-223-3p	18566	Pdcd1	0	0	0	1	0	1
mmu-miR-223-3p	18567	Pdcd2	0	1	0	0	0	1
mmu-miR-223-3p	18569	Pdcd4	0	0	0	1	0	1
mmu-miR-223-3p	18570	Pdcd6	0	0	0	1	0	1
mmu-miR-223-3p	18571	Pdcd6ip	0	0	0	1	0	1
mmu-miR-223-3p	18585	Pde9a	0	0	0	1	0	1
mmu-miR-223-3p	18588	Pde6g	0	0	0	1	0	1
mmu-miR-223-3p	18591	Pdgfb	0	0	0	1	0	1
mmu-miR-223-3p	18598	Pdha2	0	0	0	1	0	1
mmu-miR-223-3p	18599	Padi1	0	0	0	1	0	1
mmu-miR-223-3p	18600	Padi2	0	0	0	1	0	1
mmu-miR-223-3p	18601	Padi3	1	0	0	0	0	1
mmu-miR-223-3p	18602	Padi4	0	0	0	1	0	1
mmu-miR-223-3p	18606	Enpp2	0	0	0	1	0	1
mmu-miR-223-3p	18609	Pdx1	1	0	0	0	0	1
mmu-miR-223-3p	18613	Pecam1	0	0	0	1	0	1
mmu-miR-223-3p	18617	Rhox5	0	0	0	1	0	1
mmu-miR-223-3p	18618	Pemt	0	0	0	1	0	1
mmu-miR-223-3p	18619	Penk	0	0	0	1	0	1
mmu-miR-223-3p	18624	Pepd	0	0	0	1	0	1
mmu-miR-223-3p	18626	Per1	0	0	0	1	0	1
mmu-miR-223-3p	18637	Pfdn2	0	0	0	1	0	1
mmu-miR-223-3p	18639	Pfkfb1	0	0	0	1	0	1
mmu-miR-223-3p	18642	Pfkm	0	0	0	1	0	1
mmu-miR-223-3p	18643	Pfn1	0	1	0	0	0	1
mmu-miR-223-3p	18645	Pfn2	0	0	0	1	0	1
mmu-miR-223-3p	18654	Pgf	0	0	0	1	0	1
mmu-miR-223-3p	18655	Pgk1	0	0	0	1	0	1
mmu-miR-223-3p	18673	Phb	0	0	0	1	0	1
mmu-miR-223-3p	18675	Phex	0	0	0	1	0	1
mmu-miR-223-3p	18693	Pick1	0	0	0	1	0	1
mmu-miR-223-3p	18703	Pigr	0	0	0	1	0	1
mmu-miR-223-3p	18707	Pik3cd	0	0	0	1	0	1
mmu-miR-223-3p	18709	Pik3r2	1	0	0	0	0	1
mmu-miR-223-3p	18712	Pim1	1	0	0	0	0	1
mmu-miR-223-3p	18715	Pim2	0	0	0	1	0	1
mmu-miR-223-3p	18720	Pip5k1a	0	0	0	1	0	1
mmu-miR-223-3p	18722	Pira1	0	0	0	1	0	1

mmu-miR-223-3p	18725	Pira2	0	0	0	1	0	1
mmu-miR-223-3p	18727	Pira4	0	0	0	1	0	1
mmu-miR-223-3p	18729	Pira6	0	0	0	1	0	1
mmu-miR-223-3p	18733	Pirb	0	1	0	0	0	1
mmu-miR-223-3p	18742	Pitx3	0	0	0	1	0	1
mmu-miR-223-3p	18744	Pja1	0	0	0	1	0	1
mmu-miR-223-3p	18746	Pkm	0	0	0	1	0	1
mmu-miR-223-3p	18753	Prkcd	0	0	0	1	0	1
mmu-miR-223-3p	18761	Prkcq	0	0	0	1	0	1
mmu-miR-223-3p	18770	Pklr	0	0	0	1	0	1
mmu-miR-223-3p	18783	Pla2g4a	0	0	0	1	0	1
mmu-miR-223-3p	18784	Pla2g5	0	0	0	1	0	1
mmu-miR-223-3p	18786	Plaa	0	0	0	1	0	1
mmu-miR-223-3p	18787	Serpine1	1	0	0	0	0	1
mmu-miR-223-3p	18792	Plau	0	0	0	1	0	1
mmu-miR-223-3p	18793	Plaur	0	0	0	1	0	1
mmu-miR-223-3p	18795	Plcb1	0	0	0	1	0	1
mmu-miR-223-3p	18797	Plcb3	0	0	0	1	0	1
mmu-miR-223-3p	18799	Plcd1	0	0	0	1	0	1
mmu-miR-223-3p	18802	Plcd4	0	0	0	1	0	1
mmu-miR-223-3p	18803	Plcg1	0	0	0	1	0	1
mmu-miR-223-3p	18806	Pld2	0	0	0	1	0	1
mmu-miR-223-3p	18807	Pld3	0	0	0	1	0	1
mmu-miR-223-3p	18810	Plec	0	0	0	1	0	1
mmu-miR-223-3p	18811	Prl2c2	0	0	0	1	0	1
mmu-miR-223-3p	18812	Prl2c3	0	0	0	1	0	1
mmu-miR-223-3p	18816	Serpinf2	0	0	0	1	0	1
mmu-miR-223-3p	18817	Plk1	0	0	0	1	0	1
mmu-miR-223-3p	18826	Lcp1	0	0	0	1	0	1
mmu-miR-223-3p	18828	Plscr2	0	0	0	1	0	1
mmu-miR-223-3p	18829	Ccl21a	0	0	0	1	0	1
mmu-miR-223-3p	18830	Pltp	0	0	0	1	0	1
mmu-miR-223-3p	18935	Phox2b	0	0	1	0	0	1
mmu-miR-223-3p	18949	Pnn	0	0	0	1	0	1
mmu-miR-223-3p	18950	Pnp	0	0	0	1	0	1
mmu-miR-223-3p	18951	Sept5	0	0	0	1	0	1
mmu-miR-223-3p	18952	Sept4	0	0	0	1	0	1
mmu-miR-223-3p	18971	Pold1	0	0	0	1	0	1
mmu-miR-223-3p	18976	Pomc	0	0	0	1	0	1
mmu-miR-223-3p	18979	Pon1	0	0	0	1	0	1
mmu-miR-223-3p	18983	Cnot7	0	0	0	1	0	1
mmu-miR-223-3p	18987	Pou2f2	0	0	0	1	0	1
mmu-miR-223-3p	18991	Pou3f1	0	0	0	1	0	1
mmu-miR-223-3p	18992	Pou3f2	0	0	0	1	0	1
mmu-miR-223-3p	18997	Pou4f2	0	0	1	0	0	1
mmu-miR-223-3p	18999	Pou5f1	0	0	0	1	0	1
mmu-miR-223-3p	19011	Endou	0	0	0	1	0	1
mmu-miR-223-3p	19012	Ppap2a	0	0	0	1	0	1
mmu-miR-223-3p	19014	Med1	0	0	0	1	0	1
mmu-miR-223-3p	19016	Pparg	0	0	0	1	0	1
mmu-miR-223-3p	19024	Ppfibp2	0	0	0	1	0	1
mmu-miR-223-3p	19038	Ppic	0	0	0	1	0	1
mmu-miR-223-3p	19041	Ppl	0	0	0	1	0	1
mmu-miR-223-3p	19042	Ppm1a	0	0	0	1	0	1
mmu-miR-223-3p	19043	Ppm1b	0	0	0	1	0	1

mmu-miR-223-3p	19044	Ppox	0	0	0	1	0	1
mmu-miR-223-3p	19045	Ppp1ca	0	0	0	1	0	1
mmu-miR-223-3p	19049	Ppp1r1b	0	0	0	1	0	1
mmu-miR-223-3p	19051	Ppp1r17	0	0	0	1	0	1
mmu-miR-223-3p	19054	Ppp2r3d	0	0	0	1	0	1
mmu-miR-223-3p	19065	Npy4r	0	0	0	1	0	1
mmu-miR-223-3p	19069	Nup88	0	1	0	0	0	1
mmu-miR-223-3p	19070	Mob4	0	0	0	1	0	1
mmu-miR-223-3p	19073	Srgn	0	0	0	1	0	1
mmu-miR-223-3p	19074	Prg2	0	0	0	1	0	1
mmu-miR-223-3p	19076	Prim2	0	0	0	1	0	1
mmu-miR-223-3p	19079	Prkab1	0	0	0	1	0	1
mmu-miR-223-3p	19084	Prkar1a	0	0	0	1	0	1
mmu-miR-223-3p	19085	Prkar1b	0	0	0	1	0	1
mmu-miR-223-3p	19089	Prkcsh	0	0	0	1	0	1
mmu-miR-223-3p	19106	Eif2ak2	0	0	0	1	0	1
mmu-miR-223-3p	19108	Prkx	1	0	0	0	0	1
mmu-miR-223-3p	19114	Prl7a2	0	0	0	1	0	1
mmu-miR-223-3p	19124	Procr	0	0	0	1	0	1
mmu-miR-223-3p	19128	Pros1	0	0	0	1	0	1
mmu-miR-223-3p	19139	Prps1	0	0	0	1	0	1
mmu-miR-223-3p	19144	Klk6	0	0	0	1	0	1
mmu-miR-223-3p	19146	Tmprss15	0	0	0	1	0	1
mmu-miR-223-3p	19152	Prtn3	1	0	0	0	0	1
mmu-miR-223-3p	19155	Npepps	0	0	1	0	0	1
mmu-miR-223-3p	19156	Psap	0	0	0	1	0	1
mmu-miR-223-3p	19157	Cyth1	0	0	0	1	0	1
mmu-miR-223-3p	19158	Cyth2	0	0	0	1	0	1
mmu-miR-223-3p	19166	Psma2	0	0	0	1	0	1
mmu-miR-223-3p	19172	Psmb4	0	0	0	1	0	1
mmu-miR-223-3p	19175	Psmb6	0	0	0	1	0	1
mmu-miR-223-3p	19179	Psmc1	0	0	0	1	0	1
mmu-miR-223-3p	19182	Psmc3	0	0	0	1	0	1
mmu-miR-223-3p	19183	Psmc3ip	0	0	0	1	0	1
mmu-miR-223-3p	19186	Psme1	0	0	0	1	0	1
mmu-miR-223-3p	19193	Pipox	0	0	0	1	0	1
mmu-miR-223-3p	19194	Bpifa2	0	0	0	1	0	1
mmu-miR-223-3p	19200	Pstpip1	0	0	0	1	0	1
mmu-miR-223-3p	19205	Ptbp1	0	0	0	1	0	1
mmu-miR-223-3p	19212	Pter	0	0	0	1	0	1
mmu-miR-223-3p	19215	Ptgds	0	0	0	1	0	1
mmu-miR-223-3p	19216	Ptger1	0	1	0	0	0	1
mmu-miR-223-3p	19217	Ptger2	0	0	0	1	0	1
mmu-miR-223-3p	19218	Ptger3	0	0	0	1	0	1
mmu-miR-223-3p	19224	Ptgs1	0	0	0	1	0	1
mmu-miR-223-3p	19228	Pth1r	0	0	0	1	0	1
mmu-miR-223-3p	19229	Ptk2b	0	0	0	1	0	1
mmu-miR-223-3p	19231	Ptma	0	0	0	1	0	1
mmu-miR-223-3p	19240	Tmsb10	0	0	0	1	0	1
mmu-miR-223-3p	19241	Tmsb4x	0	0	0	1	0	1
mmu-miR-223-3p	19247	Ptpn11	0	0	0	1	0	1
mmu-miR-223-3p	19249	Ptpn13	0	0	0	1	0	1
mmu-miR-223-3p	19256	Ptpn20	0	0	1	0	0	1
mmu-miR-223-3p	19260	Ptpn22	1	0	0	0	0	1
mmu-miR-223-3p	19261	Sirpa	0	0	0	1	0	1

mmu-miR-223-3p	19262	Ptpra	0	0	0	1	0	1
mmu-miR-223-3p	19264	Ptprc	0	0	0	1	0	1
mmu-miR-223-3p	19272	Ptprk	0	1	0	0	0	1
mmu-miR-223-3p	19274	Ptprm	0	0	0	1	0	1
mmu-miR-223-3p	19275	Ptprn	0	0	0	1	0	1
mmu-miR-223-3p	19276	Ptprn2	0	0	0	1	0	1
mmu-miR-223-3p	19280	Ptprs	0	0	0	1	0	1
mmu-miR-223-3p	19294	Pvrl2	0	0	0	1	0	1
mmu-miR-223-3p	19298	Pex19	0	0	0	1	0	1
mmu-miR-223-3p	19300	Abcd4	0	0	0	1	0	1
mmu-miR-223-3p	19302	Pex2	0	0	0	1	0	1
mmu-miR-223-3p	19303	Pxn	0	0	0	1	0	1
mmu-miR-223-3p	19328	Rab12	1	0	0	0	0	1
mmu-miR-223-3p	19329	Rab17	0	0	0	1	0	1
mmu-miR-223-3p	19337	Rab33a	1	0	0	0	0	1
mmu-miR-223-3p	19339	Rab3a	0	0	0	1	0	1
mmu-miR-223-3p	19342	Rab4b	0	0	0	1	0	1
mmu-miR-223-3p	19348	Kif20a	0	0	0	1	0	1
mmu-miR-223-3p	19355	Rad1	0	1	0	0	0	1
mmu-miR-223-3p	19358	Rad23a	0	0	0	1	0	1
mmu-miR-223-3p	19360	Rad50	0	1	0	0	0	1
mmu-miR-223-3p	19362	Rad51ap1	0	0	0	1	0	1
mmu-miR-223-3p	19365	Rad52	0	0	0	1	0	1
mmu-miR-223-3p	19366	Rad54l	0	0	0	1	0	1
mmu-miR-223-3p	19367	Rad9a	0	0	0	1	0	1
mmu-miR-223-3p	19368	Raet1a	0	0	0	1	0	1
mmu-miR-223-3p	19369	Raet1b	0	0	0	1	0	1
mmu-miR-223-3p	19370	Raet1c	0	0	0	1	0	1
mmu-miR-223-3p	19373	Rag1	0	0	0	1	0	1
mmu-miR-223-3p	19377	Rai1	0	0	0	1	0	1
mmu-miR-223-3p	19383	Raly	0	0	0	1	0	1
mmu-miR-223-3p	19384	Ran	0	0	0	1	0	1
mmu-miR-223-3p	19386	Ranbp2	0	0	0	1	0	1
mmu-miR-223-3p	19387	Rangap1	0	0	0	1	0	1
mmu-miR-223-3p	19395	Rasgrp2	0	0	0	1	0	1
mmu-miR-223-3p	19401	Rara	0	0	0	1	0	1
mmu-miR-223-3p	19411	Rarg	0	0	0	1	0	1
mmu-miR-223-3p	19417	Rasgrf1	0	0	0	1	0	1
mmu-miR-223-3p	19434	Rax	1	0	0	0	0	1
mmu-miR-223-3p	19649	Robo3	0	0	0	1	0	1
mmu-miR-223-3p	19650	Rbl1	0	0	0	1	0	1
mmu-miR-223-3p	19657	Rbmy	0	0	0	1	0	1
mmu-miR-223-3p	19660	Rbp2	0	1	0	0	0	1
mmu-miR-223-3p	19695	Reg3g	0	0	0	1	0	1
mmu-miR-223-3p	19696	Rel	1	0	0	0	0	1
mmu-miR-223-3p	19698	Relb	0	0	0	1	0	1
mmu-miR-223-3p	19700	Rem1	0	0	0	1	0	1
mmu-miR-223-3p	19701	Ren1	0	0	0	1	0	1
mmu-miR-223-3p	19702	Ren2	0	0	0	1	0	1
mmu-miR-223-3p	19704	Upf1	1	0	0	0	0	1
mmu-miR-223-3p	19711	Resp18	0	0	0	1	0	1
mmu-miR-223-3p	19718	Rfc2	0	0	0	1	0	1
mmu-miR-223-3p	19724	Rfx1	0	1	0	0	0	1
mmu-miR-223-3p	19725	Rfx2	0	0	0	1	0	1
mmu-miR-223-3p	19732	Rgl2	0	0	0	1	0	1

mmu-miR-223-3p	19752	Rnase1	0	0	0	1	0	1
mmu-miR-223-3p	19763	Ring1	0	0	0	1	0	1
mmu-miR-223-3p	19769	Rit1	0	0	0	1	0	1
mmu-miR-223-3p	19771	Rlbp1	0	0	0	1	0	1
mmu-miR-223-3p	19773	Rln1	0	0	0	1	0	1
mmu-miR-223-3p	19820	Rlim	0	0	0	1	0	1
mmu-miR-223-3p	19821	Rnf2	1	0	0	0	0	1
mmu-miR-223-3p	19824	Trim10	0	0	0	1	0	1
mmu-miR-223-3p	19826	Rnps1	0	0	0	1	0	1
mmu-miR-223-3p	19877	Rock1	0	0	0	1	0	1
mmu-miR-223-3p	19878	Rock2	0	0	0	1	0	1
mmu-miR-223-3p	19883	Rora	0	1	0	0	0	1
mmu-miR-223-3p	19894	Rph3a	0	0	0	1	0	1
mmu-miR-223-3p	19933	Rpl21	0	0	0	1	0	1
mmu-miR-223-3p	19943	Rpl28	0	0	0	1	0	1
mmu-miR-223-3p	19981	Rpl37a	0	0	0	1	0	1
mmu-miR-223-3p	19988	Rpl6	0	0	0	1	0	1
mmu-miR-223-3p	20017	Polr1b	0	0	0	1	0	1
mmu-miR-223-3p	20022	Polr2j	0	0	0	1	0	1
mmu-miR-223-3p	20042	Rps12	0	0	0	1	0	1
mmu-miR-223-3p	20054	Rps15	0	0	0	1	0	1
mmu-miR-223-3p	20088	Rps24	0	0	0	1	0	1
mmu-miR-223-3p	20104	Rps6	0	0	0	1	0	1
mmu-miR-223-3p	20112	Rps6ka2	0	0	0	1	0	1
mmu-miR-223-3p	20163	Rsu1	0	0	0	1	0	1
mmu-miR-223-3p	20166	Rtkn	0	0	0	1	0	1
mmu-miR-223-3p	20167	Rtn2	0	0	0	1	0	1
mmu-miR-223-3p	20168	Rtn3	0	0	0	1	0	1
mmu-miR-223-3p	20174	Ruvbl2	0	0	0	1	0	1
mmu-miR-223-3p	20183	Rxrg	0	0	0	1	0	1
mmu-miR-223-3p	20184	Uimc1	0	0	0	1	0	1
mmu-miR-223-3p	20187	Ryk	0	0	0	1	0	1
mmu-miR-223-3p	20190	Ryr1	0	0	0	1	0	1
mmu-miR-223-3p	20194	S100a10	0	0	0	1	0	1
mmu-miR-223-3p	20195	S100a11	0	0	0	1	0	1
mmu-miR-223-3p	20198	S100a4	0	0	0	1	0	1
mmu-miR-223-3p	20199	S100a5	0	0	0	1	0	1
mmu-miR-223-3p	20204	Prrx2	1	0	0	0	0	1
mmu-miR-223-3p	20211	Saa4	0	0	0	1	0	1
mmu-miR-223-3p	20255	Scg3	1	0	0	0	0	1
mmu-miR-223-3p	20256	Clec11a	0	0	0	1	0	1
mmu-miR-223-3p	20259	Scin	0	0	0	1	0	1
mmu-miR-223-3p	20262	Stmn3	0	0	0	1	0	1
mmu-miR-223-3p	20264	Scn10a	0	0	0	1	0	1
mmu-miR-223-3p	20278	Scnn1g	0	0	0	1	0	1
mmu-miR-223-3p	20284	Scrg1	1	0	0	0	0	1
mmu-miR-223-3p	20288	Msr1	0	0	0	1	0	1
mmu-miR-223-3p	20289	Scx	0	0	1	0	0	1
mmu-miR-223-3p	20293	Ccl12	1	0	0	0	0	1
mmu-miR-223-3p	20296	Ccl2	0	0	0	1	0	1
mmu-miR-223-3p	20301	Ccl27a	0	0	0	1	0	1
mmu-miR-223-3p	20303	Ccl4	0	0	0	1	0	1
mmu-miR-223-3p	20305	Ccl6	0	0	0	1	0	1
mmu-miR-223-3p	20306	Ccl7	0	0	0	1	0	1
mmu-miR-223-3p	20307	Ccl8	0	0	0	1	0	1

mmu-miR-223-3p	20310	Cxcl2	1	0	0	0	0	1
mmu-miR-223-3p	20317	Serpinf1	0	0	0	1	0	1
mmu-miR-223-3p	20321	Frrs1	0	0	0	1	0	1
mmu-miR-223-3p	20336	Exoc4	0	0	0	1	0	1
mmu-miR-223-3p	20339	Sele	1	0	0	0	0	1
mmu-miR-223-3p	20341	Selenbp1	0	1	0	0	0	1
mmu-miR-223-3p	20343	Sell	0	0	0	1	0	1
mmu-miR-223-3p	20344	Selp	0	0	0	1	0	1
mmu-miR-223-3p	20345	Selplg	0	0	0	1	0	1
mmu-miR-223-3p	20347	Sema3b	0	0	0	1	0	1
mmu-miR-223-3p	20352	Sema4b	0	0	0	1	0	1
mmu-miR-223-3p	20353	Sema4c	0	0	0	1	0	1
mmu-miR-223-3p	20354	Sema4d	0	0	0	1	0	1
mmu-miR-223-3p	20355	Sema4f	0	0	0	1	0	1
mmu-miR-223-3p	20360	Sema6c	0	0	0	1	0	1
mmu-miR-223-3p	20363	Sepp1	0	0	0	1	0	1
mmu-miR-223-3p	20379	Sfrp4	0	0	0	1	0	1
mmu-miR-223-3p	20384	Srsf5	0	0	0	1	0	1
mmu-miR-223-3p	20394	Scg5	0	0	0	1	0	1
mmu-miR-223-3p	20399	Sh2b1	0	0	0	1	0	1
mmu-miR-223-3p	20400	Sh2d1a	1	0	0	0	0	1
mmu-miR-223-3p	20403	Itsn2	1	0	0	0	0	1
mmu-miR-223-3p	20416	Shc1	0	0	0	1	0	1
mmu-miR-223-3p	20438	Siah1b	0	0	0	1	0	1
mmu-miR-223-3p	20441	St3gal3	0	0	0	1	0	1
mmu-miR-223-3p	20444	St3gal2	0	0	0	1	0	1
mmu-miR-223-3p	20445	St6galnac1	1	0	0	0	0	1
mmu-miR-223-3p	20449	St8sia1	0	0	0	1	0	1
mmu-miR-223-3p	20454	St3gal5	0	0	0	1	0	1
mmu-miR-223-3p	20459	Ptk6	0	0	0	1	0	1
mmu-miR-223-3p	20460	Stil	0	0	0	1	0	1
mmu-miR-223-3p	20462	Tra2b	0	0	0	1	0	1
mmu-miR-223-3p	20463	Cox7a2l	0	0	0	1	0	1
mmu-miR-223-3p	20464	Sim1	0	0	0	1	0	1
mmu-miR-223-3p	20466	Sin3a	0	0	0	1	0	1
mmu-miR-223-3p	20467	Sin3b	0	0	0	1	0	1
mmu-miR-223-3p	20472	Six2	0	0	0	1	0	1
mmu-miR-223-3p	20475	Six5	0	1	0	0	0	1
mmu-miR-223-3p	20479	Vps4b	1	0	0	0	0	1
mmu-miR-223-3p	20480	Clpb	0	0	0	1	0	1
mmu-miR-223-3p	20481	Ski	0	0	0	1	0	1
mmu-miR-223-3p	20491	Sla	0	0	0	1	0	1
mmu-miR-223-3p	20493	Slc10a1	0	0	0	1	0	1
mmu-miR-223-3p	20496	Slc12a2	0	0	0	1	0	1
mmu-miR-223-3p	20497	Slc12a3	0	0	0	1	0	1
mmu-miR-223-3p	20498	Slc12a4	0	0	0	1	0	1
mmu-miR-223-3p	20499	Slc12a7	0	0	0	1	0	1
mmu-miR-223-3p	20500	Slc13a2	0	0	0	1	0	1
mmu-miR-223-3p	20504	Slc17a1	0	0	0	1	0	1
mmu-miR-223-3p	20505	Slc34a1	0	0	0	1	0	1
mmu-miR-223-3p	20508	Slc18a3	0	0	0	1	0	1
mmu-miR-223-3p	20509	Slc19a1	0	1	0	0	0	1
mmu-miR-223-3p	20512	Slc1a3	0	0	0	1	0	1
mmu-miR-223-3p	20514	Slc1a5	0	0	0	1	0	1
mmu-miR-223-3p	20516	Slc20a2	0	0	0	1	0	1

mmu-miR-223-3p	20517	Slc22a1	0	0	0	1	0	1
mmu-miR-223-3p	20518	Slc22a2	0	0	0	1	0	1
mmu-miR-223-3p	20519	Slc22a3	0	0	0	1	0	1
mmu-miR-223-3p	20521	Slc22a12	0	0	0	1	0	1
mmu-miR-223-3p	20523	Slc25a14	0	0	0	1	0	1
mmu-miR-223-3p	20524	Slc25a17	0	0	0	1	0	1
mmu-miR-223-3p	20527	Slc2a3	0	0	0	1	0	1
mmu-miR-223-3p	20533	Slc4a1	0	0	0	1	0	1
mmu-miR-223-3p	20534	Slc4a1ap	0	0	0	1	0	1
mmu-miR-223-3p	20536	Slc4a3	1	0	0	0	0	1
mmu-miR-223-3p	20537	Slc5a1	0	0	0	1	0	1
mmu-miR-223-3p	20540	Slc7a7	0	0	0	1	0	1
mmu-miR-223-3p	20544	Slc9a1	1	0	0	0	0	1
mmu-miR-223-3p	20555	Slfn1	0	0	0	1	0	1
mmu-miR-223-3p	20558	Slfn4	0	0	0	1	0	1
mmu-miR-223-3p	20563	Slit2	0	0	0	1	0	1
mmu-miR-223-3p	20568	Slpi	0	0	0	1	0	1
mmu-miR-223-3p	20586	Smarca4	0	0	0	1	0	1
mmu-miR-223-3p	20589	Ighmbp2	0	0	0	1	0	1
mmu-miR-223-3p	20591	Kdm5c	1	0	0	0	0	1
mmu-miR-223-3p	20592	Kdm5d	0	0	0	1	0	1
mmu-miR-223-3p	20597	Smpd1	0	0	0	1	0	1
mmu-miR-223-3p	20602	Ncor2	0	0	0	1	0	1
mmu-miR-223-3p	20609	Sstr5	0	0	0	1	0	1
mmu-miR-223-3p	20612	Siglec1	0	0	0	1	0	1
mmu-miR-223-3p	20613	Snai1	0	0	0	1	0	1
mmu-miR-223-3p	20615	Snapin	0	0	0	1	0	1
mmu-miR-223-3p	20618	Sncg	0	0	0	1	0	1
mmu-miR-223-3p	20620	Plk2	0	0	0	1	0	1
mmu-miR-223-3p	20637	Snrnp70	0	0	0	1	0	1
mmu-miR-223-3p	20638	Snrpb	0	0	0	1	0	1
mmu-miR-223-3p	20639	Snrpb2	0	0	0	1	0	1
mmu-miR-223-3p	20641	Snrpd1	0	0	0	1	0	1
mmu-miR-223-3p	20646	Snrpn	0	0	0	1	0	1
mmu-miR-223-3p	20648	Snta1	0	0	0	1	0	1
mmu-miR-223-3p	20649	Sntb1	0	0	0	1	0	1
mmu-miR-223-3p	20656	Sod2	0	0	0	1	0	1
mmu-miR-223-3p	20660	Sorl1	0	0	0	1	0	1
mmu-miR-223-3p	20665	Sox10	0	1	0	0	0	1
mmu-miR-223-3p	20669	Sox14	0	0	0	1	0	1
mmu-miR-223-3p	20672	Sox18	0	0	0	1	0	1
mmu-miR-223-3p	20674	Sox2	0	0	0	1	0	1
mmu-miR-223-3p	20675	Sox3	0	0	0	1	0	1
mmu-miR-223-3p	20677	Sox4	0	0	0	1	0	1
mmu-miR-223-3p	20684	Sp100	0	1	0	0	0	1
mmu-miR-223-3p	20690	Spam1	0	0	0	1	0	1
mmu-miR-223-3p	20692	Sparc	0	0	0	1	0	1
mmu-miR-223-3p	20698	Sphk1	0	0	0	1	0	1
mmu-miR-223-3p	20700	Serpina1a	0	0	0	1	0	1
mmu-miR-223-3p	20701	Serpina1b	0	0	0	1	0	1
mmu-miR-223-3p	20702	Serpina1c	0	0	0	1	0	1
mmu-miR-223-3p	20704	Serpina1e	0	0	0	1	0	1
mmu-miR-223-3p	20706	Serpinb9b	0	0	0	1	0	1
mmu-miR-223-3p	20709	Serpinb9f	1	0	0	0	0	1
mmu-miR-223-3p	20710	Serpinb9e	1	0	0	0	0	1

mmu-miR-223-3p	20714	Serpina3k	0	0	0	1	0	1
mmu-miR-223-3p	20715	Serpina3g	0	0	0	1	0	1
mmu-miR-223-3p	20717	Serpina3m	0	0	0	1	0	1
mmu-miR-223-3p	20720	Serpine2	0	0	0	1	0	1
mmu-miR-223-3p	20726	Serpinb9d	0	0	0	1	0	1
mmu-miR-223-3p	20728	Spic	0	0	0	1	0	1
mmu-miR-223-3p	20732	Spint1	0	0	0	1	0	1
mmu-miR-223-3p	20740	Sptan1	0	0	0	1	0	1
mmu-miR-223-3p	20750	Spp1	0	0	0	1	0	1
mmu-miR-223-3p	20751	Spr	0	0	0	1	0	1
mmu-miR-223-3p	20753	Sprr1a	0	0	0	1	0	1
mmu-miR-223-3p	20759	Sprr2e	0	0	0	1	0	1
mmu-miR-223-3p	20765	Sprr2k	0	0	0	1	0	1
mmu-miR-223-3p	20766	Sprr3	0	0	0	1	0	1
mmu-miR-223-3p	20768	Sephs2	0	0	0	1	0	1
mmu-miR-223-3p	20770	Spt1	0	0	0	1	0	1
mmu-miR-223-3p	20771	Mucl1	0	0	0	1	0	1
mmu-miR-223-3p	20776	Tmie	0	0	0	1	0	1
mmu-miR-223-3p	20778	Scarb1	0	0	0	1	0	1
mmu-miR-223-3p	20787	Srebf1	0	0	0	1	0	1
mmu-miR-223-3p	20788	Srebf2	0	0	0	1	0	1
mmu-miR-223-3p	20823	Ssb	0	0	0	1	0	1
mmu-miR-223-3p	20841	Zfp143	0	0	0	1	0	1
mmu-miR-223-3p	20843	Stag2	0	0	0	1	0	1
mmu-miR-223-3p	20846	Stat1	0	0	0	1	0	1
mmu-miR-223-3p	20849	Stat4	0	0	0	1	0	1
mmu-miR-223-3p	20851	Stat5b	0	0	0	1	0	1
mmu-miR-223-3p	20859	Sult2a1	0	0	0	1	0	1
mmu-miR-223-3p	20867	Stip1	0	0	0	1	0	1
mmu-miR-223-3p	20872	Stk16	0	0	0	1	0	1
mmu-miR-223-3p	20873	Plk4	0	0	0	1	0	1
mmu-miR-223-3p	20877	Aurkb	0	0	1	0	0	1
mmu-miR-223-3p	20888	Sult1c1	0	0	0	1	0	1
mmu-miR-223-3p	20890	Wnt8a	0	0	0	1	0	1
mmu-miR-223-3p	20892	Stra13	0	0	0	1	0	1
mmu-miR-223-3p	20897	Stra6	0	0	0	1	0	1
mmu-miR-223-3p	20901	Strap	0	0	0	1	0	1
mmu-miR-223-3p	20907	Stx1a	0	0	0	1	0	1
mmu-miR-223-3p	20909	Stx4a	0	0	0	1	0	1
mmu-miR-223-3p	20910	Stxbp1	0	0	0	1	0	1
mmu-miR-223-3p	20911	Stxbp2	0	0	1	0	0	1
mmu-miR-223-3p	20912	Stxbp3a	1	0	0	0	0	1
mmu-miR-223-3p	20917	Suclg2	0	0	0	1	0	1
mmu-miR-223-3p	20922	Supt4a	0	0	0	1	0	1
mmu-miR-223-3p	20926	Supt6	0	0	0	1	0	1
mmu-miR-223-3p	20928	Abcc9	0	0	0	1	0	1
mmu-miR-223-3p	20931	Surf2	0	0	0	1	0	1
mmu-miR-223-3p	20933	Med22	0	0	0	1	0	1
mmu-miR-223-3p	20937	Suv39h1	0	0	0	1	0	1
mmu-miR-223-3p	20941	Svs4	0	0	0	1	0	1
mmu-miR-223-3p	20955	Vamp7	1	0	0	0	0	1
mmu-miR-223-3p	20957	Sycp1	0	0	0	1	0	1
mmu-miR-223-3p	20969	Sdc1	0	0	1	0	0	1
mmu-miR-223-3p	20974	Syngr3	0	0	0	1	0	1
mmu-miR-223-3p	20975	Synj2	0	0	0	1	0	1

mmu-miR-223-3p	20981	Syt3	0	0	0	1	0	1
mmu-miR-223-3p	21333	Tac1	0	0	0	1	0	1
mmu-miR-223-3p	21334	Tac2	0	0	0	1	0	1
mmu-miR-223-3p	21339	Taf1a	1	0	0	0	0	1
mmu-miR-223-3p	21341	Taf1c	0	0	0	1	0	1
mmu-miR-223-3p	21343	Taf6	0	0	0	1	0	1
mmu-miR-223-3p	21346	Tagln2	0	0	0	1	0	1
mmu-miR-223-3p	21351	Taldo1	0	0	0	1	0	1
mmu-miR-223-3p	21374	Tbp	0	0	0	1	0	1
mmu-miR-223-3p	21375	Tbr1	0	0	0	1	0	1
mmu-miR-223-3p	21385	Tbx2	0	0	0	1	0	1
mmu-miR-223-3p	21389	Tbx6	0	0	0	1	0	1
mmu-miR-223-3p	21390	Tbxa2r	0	0	0	1	0	1
mmu-miR-223-3p	21393	Tcap	0	0	0	1	0	1
mmu-miR-223-3p	21408	Zfp354a	0	0	0	1	0	1
mmu-miR-223-3p	21413	Tcf4	0	0	0	1	0	1
mmu-miR-223-3p	21415	Tcf7l1	0	0	0	1	0	1
mmu-miR-223-3p	21423	Tcf3	0	0	0	1	0	1
mmu-miR-223-3p	21425	Tfeb	0	0	0	1	0	1
mmu-miR-223-3p	21426	Tfec	0	0	0	1	0	1
mmu-miR-223-3p	21428	Mlx	0	0	0	1	0	1
mmu-miR-223-3p	21452	Tcn2	0	0	0	1	0	1
mmu-miR-223-3p	21453	Tcof1	0	0	0	1	0	1
mmu-miR-223-3p	21461	Tcp10a	0	0	0	1	0	1
mmu-miR-223-3p	21462	Tcp10b	0	0	0	1	0	1
mmu-miR-223-3p	21648	Dynlt1b	0	0	0	1	0	1
mmu-miR-223-3p	21652	Phf1	0	0	0	1	0	1
mmu-miR-223-3p	21665	Tdg	0	0	0	1	0	1
mmu-miR-223-3p	21667	Tdgf1	0	0	0	1	0	1
mmu-miR-223-3p	21673	Dntt	0	0	0	1	0	1
mmu-miR-223-3p	21677	Tead2	0	0	1	0	0	1
mmu-miR-223-3p	21678	Tead3	0	0	0	1	0	1
mmu-miR-223-3p	21681	Alyref	0	0	0	1	0	1
mmu-miR-223-3p	21682	Tec	0	0	0	1	0	1
mmu-miR-223-3p	21683	Tecta	0	0	0	1	0	1
mmu-miR-223-3p	21743	Inmt	0	0	0	1	0	1
mmu-miR-223-3p	21750	Terf2	0	0	0	1	0	1
mmu-miR-223-3p	21755	Prss39	0	0	0	1	0	1
mmu-miR-223-3p	21756	Prss40	0	1	0	0	0	1
mmu-miR-223-3p	21761	Morf4l1	0	0	0	1	0	1
mmu-miR-223-3p	21762	Psmd2	0	1	0	0	0	1
mmu-miR-223-3p	21769	Zfand3	0	0	0	1	0	1
mmu-miR-223-3p	21784	Tff1	0	0	0	1	0	1
mmu-miR-223-3p	21786	Tff3	0	0	0	1	0	1
mmu-miR-223-3p	21787	Tfg	0	0	0	1	0	1
mmu-miR-223-3p	21807	Tsc22d1	0	0	0	1	0	1
mmu-miR-223-3p	21810	Tgfbi	0	0	0	1	0	1
mmu-miR-223-3p	21815	Tgif1	1	0	0	0	0	1
mmu-miR-223-3p	21816	Tgm1	0	0	0	1	0	1
mmu-miR-223-3p	21818	Tgm3	0	0	0	1	0	1
mmu-miR-223-3p	21819	Tg	0	1	0	0	0	1
mmu-miR-223-3p	21821	Ift88	0	0	0	1	0	1
mmu-miR-223-3p	21826	Thbs2	0	0	0	1	0	1
mmu-miR-223-3p	21827	Thbs3	0	1	0	0	0	1
mmu-miR-223-3p	21833	Thra	0	0	0	1	0	1

mmu-miR-223-3p	21848	Trim24	0	0	0	1	0	1
mmu-miR-223-3p	21857	Timp1	1	0	0	0	0	1
mmu-miR-223-3p	21859	Timp3	0	0	0	1	0	1
mmu-miR-223-3p	21877	Tk1	0	0	0	1	0	1
mmu-miR-223-3p	21886	Tle2	0	0	0	1	0	1
mmu-miR-223-3p	21887	Tle3	0	0	0	1	0	1
mmu-miR-223-3p	21892	Tll1	0	0	0	1	0	1
mmu-miR-223-3p	21908	Tlx1	0	0	0	1	0	1
mmu-miR-223-3p	21909	Tlx2	0	0	0	1	0	1
mmu-miR-223-3p	21915	Dtymk	0	0	0	1	0	1
mmu-miR-223-3p	21916	Tmod1	0	0	0	1	0	1
mmu-miR-223-3p	21922	Clec3b	0	0	0	1	0	1
mmu-miR-223-3p	21924	Tnnc1	0	0	0	1	0	1
mmu-miR-223-3p	21925	Tnnc2	0	0	0	1	0	1
mmu-miR-223-3p	21926	Tnf	0	0	0	1	0	1
mmu-miR-223-3p	21937	Tnfrsf1a	0	0	0	1	0	1
mmu-miR-223-3p	21939	Cd40	0	0	0	1	0	1
mmu-miR-223-3p	21942	Tnfrsf9	0	0	0	1	0	1
mmu-miR-223-3p	21944	Tnfsf12	0	0	0	1	0	1
mmu-miR-223-3p	21946	Pglyrp1	0	0	0	1	0	1
mmu-miR-223-3p	21947	Cd40lg	0	0	0	1	0	1
mmu-miR-223-3p	21952	Tnni1	0	0	0	1	0	1
mmu-miR-223-3p	21955	Tnnt1	0	0	0	1	0	1
mmu-miR-223-3p	21956	Tnnt2	0	0	0	1	0	1
mmu-miR-223-3p	21958	Tnp1	0	0	0	1	0	1
mmu-miR-223-3p	21960	Tnr	0	0	0	1	0	1
mmu-miR-223-3p	21974	Top2b	0	0	0	1	0	1
mmu-miR-223-3p	21982	Tmem165	0	0	0	1	0	1
mmu-miR-223-3p	21990	Tph1	0	0	0	1	0	1
mmu-miR-223-3p	22018	Tpo	0	0	0	1	0	1
mmu-miR-223-3p	22021	Tpst1	0	0	0	1	0	1
mmu-miR-223-3p	22024	Crisp2	0	0	0	1	0	1
mmu-miR-223-3p	22025	Nr2c1	0	0	0	1	0	1
mmu-miR-223-3p	22029	Traf1	0	0	0	1	0	1
mmu-miR-223-3p	22038	Plscr1	0	0	0	1	0	1
mmu-miR-223-3p	22040	Trex1	0	0	0	1	0	1
mmu-miR-223-3p	22041	Trf	0	0	0	1	0	1
mmu-miR-223-3p	22044	Trh	0	0	1	0	0	1
mmu-miR-223-3p	22062	Trp73	0	0	0	1	0	1
mmu-miR-223-3p	22064	Trpc2	0	1	0	0	0	1
mmu-miR-223-3p	22070	Tpt1	0	0	0	1	0	1
mmu-miR-223-3p	22083	Ctr9	1	0	0	0	0	1
mmu-miR-223-3p	22092	Rsph1	0	0	0	1	0	1
mmu-miR-223-3p	22113	Phlda2	0	0	0	1	0	1
mmu-miR-223-3p	22114	Tssk1	0	0	0	1	0	1
mmu-miR-223-3p	22116	Tsks	0	0	0	1	0	1
mmu-miR-223-3p	22117	Tst	0	0	0	1	0	1
mmu-miR-223-3p	22122	Tsta3	0	0	0	1	0	1
mmu-miR-223-3p	22127	Tsx	1	0	0	0	0	1
mmu-miR-223-3p	22129	Ttc3	0	0	0	1	0	1
mmu-miR-223-3p	22135	Tgoln2	1	0	0	0	0	1
mmu-miR-223-3p	22139	Ttr	1	0	0	0	0	1
mmu-miR-223-3p	22144	Tuba3a	0	0	0	1	0	1
mmu-miR-223-3p	22147	Tuba3b	0	0	0	1	0	1
mmu-miR-223-3p	22151	Tubb2a	0	0	0	1	0	1

mmu-miR-223-3p	22153	Tubb4a	0	0	0	1	0	1
mmu-miR-223-3p	22163	Tnfrsf4	0	0	0	1	0	1
mmu-miR-223-3p	22164	Tnfsf4	0	0	0	1	0	1
mmu-miR-223-3p	22165	Txk	0	0	0	1	0	1
mmu-miR-223-3p	22166	Txn1	0	0	0	1	0	1
mmu-miR-223-3p	22178	Tyrp1	0	0	0	1	0	1
mmu-miR-223-3p	22184	Zrsr2	0	0	0	1	0	1
mmu-miR-223-3p	22185	U2af2	0	0	0	1	0	1
mmu-miR-223-3p	22192	Ube2m	0	0	0	1	0	1
mmu-miR-223-3p	22193	Ube2e3	0	0	0	1	0	1
mmu-miR-223-3p	22202	Uba1y	0	0	0	1	0	1
mmu-miR-223-3p	22215	Ube3a	0	0	0	1	0	1
mmu-miR-223-3p	22222	Ubr1	0	0	0	1	0	1
mmu-miR-223-3p	22227	Ucp1	0	0	0	1	0	1
mmu-miR-223-3p	22234	Ugcg	0	0	0	1	0	1
mmu-miR-223-3p	22236	Ugt1a2	0	0	0	1	0	1
mmu-miR-223-3p	22239	Ugt8a	0	0	0	1	0	1
mmu-miR-223-3p	22241	Ulk1	0	0	0	1	0	1
mmu-miR-223-3p	22242	Umod	0	0	0	1	0	1
mmu-miR-223-3p	22245	Uck1	0	0	0	1	0	1
mmu-miR-223-3p	22256	Ung	0	0	0	1	0	1
mmu-miR-223-3p	22258	Usp4	1	0	0	0	0	1
mmu-miR-223-3p	22260	Nr1h2	0	0	0	1	0	1
mmu-miR-223-3p	22264	Prap1	0	0	0	1	0	1
mmu-miR-223-3p	22269	Upk2	0	0	0	1	0	1
mmu-miR-223-3p	22270	Upk3a	0	0	0	1	0	1
mmu-miR-223-3p	22284	Usp9x	0	0	0	1	0	1
mmu-miR-223-3p	22296	Vmn1r51	0	0	0	1	0	1
mmu-miR-223-3p	22300	Vmn2r123	1	0	0	0	0	1
mmu-miR-223-3p	22307	Vmn2r10	0	1	0	0	0	1
mmu-miR-223-3p	22308	Vmn2r122	1	0	0	0	0	1
mmu-miR-223-3p	22320	Vamp8	0	0	0	1	0	1
mmu-miR-223-3p	22323	Vasp	0	0	0	1	0	1
mmu-miR-223-3p	22325	Vav2	0	0	0	1	0	1
mmu-miR-223-3p	22330	Vcl	0	0	0	1	0	1
mmu-miR-223-3p	22334	Vdac2	0	0	0	1	0	1
mmu-miR-223-3p	22337	Vdr	0	0	0	1	0	1
mmu-miR-223-3p	22340	Vegfb	0	0	0	1	0	1
mmu-miR-223-3p	22341	Vegfc	0	0	0	1	0	1
mmu-miR-223-3p	22342	Lin7b	0	0	0	1	0	1
mmu-miR-223-3p	22349	Vil1	0	0	0	1	0	1
mmu-miR-223-3p	22350	Ezr	0	0	0	1	0	1
mmu-miR-223-3p	22351	Vill	0	0	0	1	0	1
mmu-miR-223-3p	22353	Vip	0	0	0	1	0	1
mmu-miR-223-3p	22361	Vnn1	0	0	0	1	0	1
mmu-miR-223-3p	22365	Vps45	0	0	0	1	0	1
mmu-miR-223-3p	22367	Vrk1	0	0	0	1	0	1
mmu-miR-223-3p	22370	Vtn	0	0	0	1	0	1
mmu-miR-223-3p	22371	Vwf	0	0	0	1	0	1
mmu-miR-223-3p	22376	Was	0	0	0	1	0	1
mmu-miR-223-3p	22378	Wbp2	0	0	0	1	0	1
mmu-miR-223-3p	22381	Wbp5	0	0	0	1	0	1
mmu-miR-223-3p	22388	Wdr1	0	0	0	1	0	1
mmu-miR-223-3p	22404	Wiz	0	0	0	1	0	1
mmu-miR-223-3p	22408	Wnt1	0	0	0	1	0	1

mmu-miR-223-3p	22409	Wnt10a	0	0	0	1	0	1
mmu-miR-223-3p	22412	Wnt9b	0	0	0	1	0	1
mmu-miR-223-3p	22415	Wnt3	0	0	1	0	0	1
mmu-miR-223-3p	22418	Wnt5a	0	0	0	1	0	1
mmu-miR-223-3p	22420	Wnt6	0	0	0	1	0	1
mmu-miR-223-3p	22423	Wnt8b	0	0	0	1	0	1
mmu-miR-223-3p	22428	Dctn6	0	0	0	1	0	1
mmu-miR-223-3p	22433	Xbp1	0	0	0	1	0	1
mmu-miR-223-3p	22436	Xdh	0	0	0	1	0	1
mmu-miR-223-3p	22596	Xrcc5	0	0	0	1	0	1
mmu-miR-223-3p	22598	Slc6a18	0	0	0	1	0	1
mmu-miR-223-3p	22608	Ybx1	1	0	0	0	0	1
mmu-miR-223-3p	22627	Ywhae	0	0	0	1	0	1
mmu-miR-223-3p	22628	Ywhag	0	0	0	1	0	1
mmu-miR-223-3p	22630	Ywhaq	0	0	0	1	0	1
mmu-miR-223-3p	22637	Zap70	0	0	0	1	0	1
mmu-miR-223-3p	22642	Zbtb17	0	0	0	1	0	1
mmu-miR-223-3p	22643	Zfp101	0	0	0	1	0	1
mmu-miR-223-3p	22644	Rnf103	0	0	0	1	0	1
mmu-miR-223-3p	22648	Zfp11	0	0	0	1	0	1
mmu-miR-223-3p	22654	Zfp13	0	0	0	1	0	1
mmu-miR-223-3p	22658	Pcgf2	0	0	0	1	0	1
mmu-miR-223-3p	22673	Zfp185	0	0	0	1	0	1
mmu-miR-223-3p	22678	Zfp2	0	0	0	1	0	1
mmu-miR-223-3p	22685	Zfp239	0	0	0	1	0	1
mmu-miR-223-3p	22689	Zfp27	0	0	0	1	0	1
mmu-miR-223-3p	22693	Zfp30	0	0	0	1	0	1
mmu-miR-223-3p	22694	Zfp35	0	0	0	1	0	1
mmu-miR-223-3p	22701	Zfp41	0	0	0	1	0	1
mmu-miR-223-3p	22710	Zfp52	0	0	0	1	0	1
mmu-miR-223-3p	22715	Zfp57	1	0	0	0	0	1
mmu-miR-223-3p	22720	Zfp62	0	0	0	1	0	1
mmu-miR-223-3p	22724	Zbtb7b	0	0	0	1	0	1
mmu-miR-223-3p	22756	Zfp94	0	0	0	1	0	1
mmu-miR-223-3p	22762	Zfpm2	0	0	0	1	0	1
mmu-miR-223-3p	22768	Zfy2	0	0	0	1	0	1
mmu-miR-223-3p	22771	Zic1	0	0	0	1	0	1
mmu-miR-223-3p	22778	Ikzf1	0	0	0	1	0	1
mmu-miR-223-3p	22789	Zp3r	0	0	0	1	0	1
mmu-miR-223-3p	22793	Zyx	0	0	0	1	0	1
mmu-miR-223-3p	23789	Coro1b	0	0	0	1	0	1
mmu-miR-223-3p	23827	Bpnt1	0	0	0	1	0	1
mmu-miR-223-3p	23828	Bves	0	0	0	1	0	1
mmu-miR-223-3p	23829	C1ql1	0	0	0	1	0	1
mmu-miR-223-3p	23830	Capn10	0	0	0	1	0	1
mmu-miR-223-3p	23834	Cdc6	0	0	0	1	0	1
mmu-miR-223-3p	23837	Cfdp1	0	0	0	1	0	1
mmu-miR-223-3p	23845	Clec5a	0	0	0	1	0	1
mmu-miR-223-3p	23857	Dmtf1	0	0	0	1	0	1
mmu-miR-223-3p	23863	Dand5	1	0	0	0	0	1
mmu-miR-223-3p	23872	Ets2	0	0	1	0	0	1
mmu-miR-223-3p	23873	Faim	0	0	0	1	0	1
mmu-miR-223-3p	23874	Farsb	0	0	0	1	0	1
mmu-miR-223-3p	23877	Fiz1	0	0	0	1	0	1
mmu-miR-223-3p	23880	Fyb	0	0	0	1	0	1

mmu-miR-223-3p	23908	Hs2st1	0	0	0	1	0	1
mmu-miR-223-3p	23917	Impdh1	0	0	0	1	0	1
mmu-miR-223-3p	23920	Insrr	0	0	0	1	0	1
mmu-miR-223-3p	23925	Kel	0	0	0	1	0	1
mmu-miR-223-3p	23942	Mta2	0	0	0	1	0	1
mmu-miR-223-3p	23943	Esyt1	0	0	0	1	0	1
mmu-miR-223-3p	23954	Nek3	0	0	0	1	0	1
mmu-miR-223-3p	23955	Nek4	0	0	0	1	0	1
mmu-miR-223-3p	23956	Neu2	0	0	0	1	0	1
mmu-miR-223-3p	23958	Nr2e3	0	0	0	1	0	1
mmu-miR-223-3p	23960	Oas1g	0	0	0	1	0	1
mmu-miR-223-3p	23963	Tenm1	0	1	0	0	0	1
mmu-miR-223-3p	23966	Tenm4	0	1	0	0	0	1
mmu-miR-223-3p	23967	Osr1	0	0	0	1	0	1
mmu-miR-223-3p	23969	Pacsin1	0	0	0	1	0	1
mmu-miR-223-3p	23970	Pacsin2	0	0	0	1	0	1
mmu-miR-223-3p	23980	Pebp1	0	0	0	1	0	1
mmu-miR-223-3p	23983	Pcbp1	0	0	0	1	0	1
mmu-miR-223-3p	23986	Eci2	0	0	0	1	0	1
mmu-miR-223-3p	23989	Med24	0	1	0	0	0	1
mmu-miR-223-3p	23991	Cib1	0	0	0	1	0	1
mmu-miR-223-3p	23992	Prkra	0	0	0	1	0	1
mmu-miR-223-3p	23996	Psmc4	0	0	0	1	0	1
mmu-miR-223-3p	23999	Twf2	0	0	0	1	0	1
mmu-miR-223-3p	24001	Tiam2	0	0	0	1	0	1
mmu-miR-223-3p	24010	Ik	1	0	0	0	0	1
mmu-miR-223-3p	24017	Rnf13	0	0	0	1	0	1
mmu-miR-223-3p	24018	Rngtt	0	0	0	1	0	1
mmu-miR-223-3p	24044	Scamp2	0	0	0	1	0	1
mmu-miR-223-3p	24046	Scn11a	0	0	0	1	0	1
mmu-miR-223-3p	24052	Sgcd	0	1	0	0	0	1
mmu-miR-223-3p	24053	Sgcg	0	0	0	1	0	1
mmu-miR-223-3p	24058	Sigirr	0	0	0	1	0	1
mmu-miR-223-3p	24061	Smc1a	0	0	0	1	0	1
mmu-miR-223-3p	24063	Spry1	0	0	0	1	0	1
mmu-miR-223-3p	24068	Sra1	0	0	0	1	0	1
mmu-miR-223-3p	24069	Sufu	0	0	0	1	0	1
mmu-miR-223-3p	24070	Mpdu1	0	0	0	1	0	1
mmu-miR-223-3p	24074	Taf7	0	0	1	0	0	1
mmu-miR-223-3p	24075	Taf10	0	1	0	0	0	1
mmu-miR-223-3p	24088	Tlr2	1	0	0	0	0	1
mmu-miR-223-3p	24100	Tpra1	0	0	0	1	0	1
mmu-miR-223-3p	24105	Rbck1	0	0	0	1	0	1
mmu-miR-223-3p	24108	Ubd	0	0	0	1	0	1
mmu-miR-223-3p	24110	Usp18	0	0	0	1	0	1
mmu-miR-223-3p	24111	Uts2	0	0	0	1	0	1
mmu-miR-223-3p	24113	Vax2	0	0	0	1	0	1
mmu-miR-223-3p	24117	Wif1	0	0	0	1	0	1
mmu-miR-223-3p	24128	Xrn2	0	0	0	1	0	1
mmu-miR-223-3p	26358	Aldh1a7	0	0	0	1	0	1
mmu-miR-223-3p	26362	Axl	0	0	0	1	0	1
mmu-miR-223-3p	26364	Cd97	0	0	0	1	0	1
mmu-miR-223-3p	26370	Cetn2	0	0	0	1	0	1
mmu-miR-223-3p	26372	Clcn6	0	0	0	1	0	1
mmu-miR-223-3p	26378	Decr2	0	0	1	0	0	1

mmu-miR-223-3p	26379	Esrra	0	0	0	1	0	1
mmu-miR-223-3p	26382	Fgd2	0	0	0	1	0	1
mmu-miR-223-3p	26384	Gnpda1	0	0	1	0	0	1
mmu-miR-223-3p	26385	Grk6	0	0	0	1	0	1
mmu-miR-223-3p	26386	Hsf4	0	1	0	0	0	1
mmu-miR-223-3p	26395	Map2k1	0	0	0	1	0	1
mmu-miR-223-3p	26396	Map2k2	0	0	0	1	0	1
mmu-miR-223-3p	26399	Map2k6	0	1	0	0	0	1
mmu-miR-223-3p	26400	Map2k7	0	0	0	1	0	1
mmu-miR-223-3p	26403	Map3k11	1	0	0	0	0	1
mmu-miR-223-3p	26407	Map3k4	0	0	0	1	0	1
mmu-miR-223-3p	26427	Creb3l1	0	0	0	1	0	1
mmu-miR-223-3p	26431	Git2	0	0	0	1	0	1
mmu-miR-223-3p	26438	Psg18	0	0	0	1	0	1
mmu-miR-223-3p	26439	Psg19	0	0	0	1	0	1
mmu-miR-223-3p	26440	Psma1	0	0	0	1	0	1
mmu-miR-223-3p	26444	Psma7	0	0	0	1	0	1
mmu-miR-223-3p	26445	Psmb2	0	0	0	1	0	1
mmu-miR-223-3p	26448	Stk30	0	0	0	1	0	1
mmu-miR-223-3p	26451	Rpl27a	0	0	0	1	0	1
mmu-miR-223-3p	26459	Slc27a5	0	0	0	1	0	1
mmu-miR-223-3p	26465	Zfp146	1	0	0	0	0	1
mmu-miR-223-3p	26466	Zfp260	0	0	0	1	0	1
mmu-miR-223-3p	26549	Itgb1bp2	0	0	0	1	0	1
mmu-miR-223-3p	26554	Cul3	0	0	0	1	0	1
mmu-miR-223-3p	26564	Ror2	0	0	0	1	0	1
mmu-miR-223-3p	26565	Pla2g10	0	0	0	1	0	1
mmu-miR-223-3p	26754	Cops5	0	0	0	1	0	1
mmu-miR-223-3p	26757	Dpysl4	0	0	0	1	0	1
mmu-miR-223-3p	26879	B3galnt1	0	0	1	0	0	1
mmu-miR-223-3p	26885	Casp8ap2	0	0	0	1	0	1
mmu-miR-223-3p	26886	Cenph	0	0	0	1	0	1
mmu-miR-223-3p	26888	Clec4a2	0	0	0	1	0	1
mmu-miR-223-3p	26894	Cops7a	0	0	0	1	0	1
mmu-miR-223-3p	26898	Ctsj	0	0	0	1	0	1
mmu-miR-223-3p	26900	Ddx3y	0	0	0	1	0	1
mmu-miR-223-3p	26904	Sh2d1b1	0	0	0	1	0	1
mmu-miR-223-3p	26908	Eif2s3y	1	0	0	0	0	1
mmu-miR-223-3p	26912	Gcat	0	0	0	1	0	1
mmu-miR-223-3p	26914	H2afy	0	0	0	1	0	1
mmu-miR-223-3p	26918	Ern2	0	1	0	0	0	1
mmu-miR-223-3p	26922	Mecr	0	0	0	1	0	1
mmu-miR-223-3p	26927	Foxl2	0	0	0	1	0	1
mmu-miR-223-3p	26938	St6galnac5	1	0	0	0	0	1
mmu-miR-223-3p	26940	Ecsit	0	0	0	1	0	1
mmu-miR-223-3p	26941	Slc9a3r1	0	0	0	1	0	1
mmu-miR-223-3p	26949	Vat1	0	0	0	1	0	1
mmu-miR-223-3p	26950	Vsnl1	0	0	0	1	0	1
mmu-miR-223-3p	26951	Zw10	0	0	0	1	0	1
mmu-miR-223-3p	26968	Islr	0	0	0	1	0	1
mmu-miR-223-3p	26972	Spo11	0	0	0	1	0	1
mmu-miR-223-3p	27027	Tspan32	0	0	0	1	0	1
mmu-miR-223-3p	27047	Omd	0	0	0	1	0	1
mmu-miR-223-3p	27050	Rps3	0	0	1	0	0	1
mmu-miR-223-3p	27054	Sec23b	0	0	0	1	0	1

mmu-miR-223-3p	27056	Irf5	0	0	0	1	0	1
mmu-miR-223-3p	27057	Ncoa4	0	0	0	1	0	1
mmu-miR-223-3p	27060	Tcirg1	0	0	0	1	0	1
mmu-miR-223-3p	27061	Bcap31	0	0	0	1	0	1
mmu-miR-223-3p	27140	Tlx3	0	1	0	0	0	1
mmu-miR-223-3p	27206	Nrk	1	0	0	0	0	1
mmu-miR-223-3p	27214	Dbf4	0	0	0	1	0	1
mmu-miR-223-3p	27219	Sgk2	0	0	0	1	0	1
mmu-miR-223-3p	27222	Atp1a4	0	0	0	1	0	1
mmu-miR-223-3p	27267	Cars	0	0	0	1	0	1
mmu-miR-223-3p	27274	Zfp354b	1	0	0	0	0	1
mmu-miR-223-3p	27277	Golga5	0	0	0	1	0	1
mmu-miR-223-3p	27279	Tnfrsf12a	0	0	0	1	0	1
mmu-miR-223-3p	27280	Phlda3	0	0	0	1	0	1
mmu-miR-223-3p	27354	Nbn	1	0	0	0	0	1
mmu-miR-223-3p	27357	Gyg	0	0	0	1	0	1
mmu-miR-223-3p	27359	Sytl4	0	0	0	1	0	1
mmu-miR-223-3p	27362	Dnajb9	0	0	0	1	0	1
mmu-miR-223-3p	27371	Sh2d2a	0	0	0	1	0	1
mmu-miR-223-3p	27374	Prmt5	0	0	0	1	0	1
mmu-miR-223-3p	27376	Slc25a10	0	0	0	1	0	1
mmu-miR-223-3p	27378	Tcl1b3	1	0	0	0	0	1
mmu-miR-223-3p	27384	Akr1c13	0	0	0	1	0	1
mmu-miR-223-3p	27385	Magel2	0	0	0	1	0	1
mmu-miR-223-3p	27387	Sh2d3c	0	0	0	1	0	1
mmu-miR-223-3p	27390	Mmel1	0	0	0	1	0	1
mmu-miR-223-3p	27402	Pdhx	1	0	0	0	0	1
mmu-miR-223-3p	27403	Abca7	0	0	0	1	0	1
mmu-miR-223-3p	27404	Abca8b	0	0	0	1	0	1
mmu-miR-223-3p	27405	Abcg3	0	0	0	1	0	1
mmu-miR-223-3p	27409	Abcg5	0	0	0	1	0	1
mmu-miR-223-3p	27410	Abca3	0	0	0	1	0	1
mmu-miR-223-3p	27411	Slc14a2	0	0	0	1	0	1
mmu-miR-223-3p	27413	Abcb11	0	0	0	1	0	1
mmu-miR-223-3p	27421	Abcc6	0	0	0	1	0	1
mmu-miR-223-3p	27426	Nagpa	0	0	0	1	0	1
mmu-miR-223-3p	27632	Nelfe	0	0	0	1	0	1
mmu-miR-223-3p	27643	Ubl4	0	0	0	1	0	1
mmu-miR-223-3p	27756	Lsm2	0	0	0	1	0	1
mmu-miR-223-3p	27762	Vwa7	0	0	0	1	0	1
mmu-miR-223-3p	27883	Tango2	0	0	0	1	0	1
mmu-miR-223-3p	27886	Dgcr14	0	0	0	1	0	1
mmu-miR-223-3p	27965	Spg21	0	0	0	1	0	1
mmu-miR-223-3p	27984	Efhd2	0	0	0	1	0	1
mmu-miR-223-3p	28015	Polr2m	0	0	0	1	0	1
mmu-miR-223-3p	28019	Ing4	0	1	0	0	0	1
mmu-miR-223-3p	28077	Med10	0	0	1	0	0	1
mmu-miR-223-3p	28078	Prl5a1	0	0	0	1	0	1
mmu-miR-223-3p	28114	Nsun2	0	0	0	1	0	1
mmu-miR-223-3p	28126	Nop16	0	0	0	1	0	1
mmu-miR-223-3p	28146	Serp1	0	0	0	1	0	1
mmu-miR-223-3p	29806	Limd1	0	0	0	1	0	1
mmu-miR-223-3p	29813	Zfp385a	0	0	0	1	0	1
mmu-miR-223-3p	29815	Bcar3	0	0	0	1	0	1
mmu-miR-223-3p	29816	Hip1r	0	0	0	1	0	1

mmu-miR-223-3p	29817	Igfbp7	0	0	0	1	0	1
mmu-miR-223-3p	29818	Hspb7	0	0	0	1	0	1
mmu-miR-223-3p	29819	Stau2	0	0	0	1	0	1
mmu-miR-223-3p	29845	Olfr155	1	0	0	0	0	1
mmu-miR-223-3p	29856	Smtn	0	0	0	1	0	1
mmu-miR-223-3p	29865	Cabp5	0	0	0	1	0	1
mmu-miR-223-3p	29867	Cabp1	0	0	0	1	0	1
mmu-miR-223-3p	29870	Gtse1	0	0	0	1	0	1
mmu-miR-223-3p	29871	Scmh1	0	0	0	1	0	1
mmu-miR-223-3p	29875	Iqgap1	0	0	0	1	0	1
mmu-miR-223-3p	30044	Opn4	0	0	0	1	0	1
mmu-miR-223-3p	30045	Dnajc12	0	0	0	1	0	1
mmu-miR-223-3p	30051	Spdef	0	0	0	1	0	1
mmu-miR-223-3p	30052	Pcsk1n	0	0	0	1	0	1
mmu-miR-223-3p	30057	Timm8b	0	0	0	1	0	1
mmu-miR-223-3p	30059	Timm10	0	0	0	1	0	1
mmu-miR-223-3p	30060	Mfi2	0	0	0	1	0	1
mmu-miR-223-3p	30838	Fbxw4	0	0	0	1	0	1
mmu-miR-223-3p	30839	Fbxw5	0	0	0	1	0	1
mmu-miR-223-3p	30853	Mlf2	0	0	0	1	0	1
mmu-miR-223-3p	30926	Glrx3	0	0	0	1	0	1
mmu-miR-223-3p	30927	Snai3	0	0	0	1	0	1
mmu-miR-223-3p	30930	Vps26a	0	0	0	1	0	1
mmu-miR-223-3p	30933	Tor2a	0	0	0	1	0	1
mmu-miR-223-3p	30937	Lmcd1	0	0	0	1	0	1
mmu-miR-223-3p	30938	Fgd3	0	0	0	1	0	1
mmu-miR-223-3p	30941	Usp21	0	0	0	1	0	1
mmu-miR-223-3p	30942	Hnf4g	0	0	0	1	0	1
mmu-miR-223-3p	30948	Bin1	0	0	0	1	0	1
mmu-miR-223-3p	30949	Lcmt1	0	0	0	1	0	1
mmu-miR-223-3p	30951	Cbx8	0	0	0	1	0	1
mmu-miR-223-3p	30953	Schip1	0	0	0	1	0	1
mmu-miR-223-3p	50492	Thop1	0	0	0	1	0	1
mmu-miR-223-3p	50497	Hspa14	0	1	0	0	0	1
mmu-miR-223-3p	50501	Prok2	0	0	0	1	0	1
mmu-miR-223-3p	50524	Sall2	0	0	0	1	0	1
mmu-miR-223-3p	50525	Spag6	0	0	0	1	0	1
mmu-miR-223-3p	50540	Igbp1b	0	0	0	1	0	1
mmu-miR-223-3p	50708	Hist1h1c	0	0	0	1	0	1
mmu-miR-223-3p	50709	Hist1h1e	0	0	0	1	0	1
mmu-miR-223-3p	50720	Sacs	0	0	0	1	0	1
mmu-miR-223-3p	50721	Sirt6	0	0	0	1	0	1
mmu-miR-223-3p	50723	Icosl	0	0	0	1	0	1
mmu-miR-223-3p	50724	Sap30l	0	0	0	1	0	1
mmu-miR-223-3p	50762	Fbxo6	0	0	0	1	0	1
mmu-miR-223-3p	50765	Tfr2	0	0	0	1	0	1
mmu-miR-223-3p	50771	Atp9b	0	0	0	1	0	1
mmu-miR-223-3p	50772	Mapk6	0	0	0	1	0	1
mmu-miR-223-3p	50775	Krtap5-4	0	0	0	1	0	1
mmu-miR-223-3p	50779	Rgs6	0	0	0	1	0	1
mmu-miR-223-3p	50788	Fbxl8	0	0	0	1	0	1
mmu-miR-223-3p	50789	Fbxl3	0	0	0	1	0	1
mmu-miR-223-3p	50790	Acsl4	0	0	0	1	0	1
mmu-miR-223-3p	50797	Copb2	0	0	0	1	0	1
mmu-miR-223-3p	50799	Slc25a13	0	0	0	1	0	1

mmu-miR-223-3p	50849	Rnf10	0	0	0	1	0	1
mmu-miR-223-3p	50873	Park2	0	0	0	1	0	1
mmu-miR-223-3p	50880	Scly	0	0	0	1	0	1
mmu-miR-223-3p	50883	Chek2	0	0	0	1	0	1
mmu-miR-223-3p	50908	C1s	0	0	0	1	0	1
mmu-miR-223-3p	50909	C1ra	0	0	0	1	0	1
mmu-miR-223-3p	50911	Exosc9	0	0	0	1	0	1
mmu-miR-223-3p	50913	Olig2	0	0	0	1	0	1
mmu-miR-223-3p	50915	Grb14	1	0	0	0	0	1
mmu-miR-223-3p	50918	Myadm	0	0	0	1	0	1
mmu-miR-223-3p	50927	Nasp	0	0	0	1	0	1
mmu-miR-223-3p	50929	Il22	0	0	0	1	0	1
mmu-miR-223-3p	50931	Il27ra	0	0	0	1	0	1
mmu-miR-223-3p	50932	Mink1	0	0	0	1	0	1
mmu-miR-223-3p	50935	St6galnac6	0	0	0	1	0	1
mmu-miR-223-3p	50996	Pdcd7	0	0	0	1	0	1
mmu-miR-223-3p	51791	Rgs14	0	0	0	1	0	1
mmu-miR-223-3p	51792	Ppp2r1a	0	0	0	1	0	1
mmu-miR-223-3p	51796	Srrm1	0	0	0	1	0	1
mmu-miR-223-3p	51797	Ctps	0	0	0	1	0	1
mmu-miR-223-3p	51800	Bok	0	0	0	1	0	1
mmu-miR-223-3p	51812	Mcrs1	0	0	0	1	0	1
mmu-miR-223-3p	51944	Knstrn	0	0	0	1	0	1
mmu-miR-223-3p	52036	Ppp6r3	0	0	0	1	0	1
mmu-miR-223-3p	52055	Rab11fip5	0	0	0	1	0	1
mmu-miR-223-3p	52064	Coq5	0	0	1	0	0	1
mmu-miR-223-3p	52174	Tmem222	0	0	0	1	0	1
mmu-miR-223-3p	52184	Odf2l	0	0	0	1	0	1
mmu-miR-223-3p	52206	Anapc4	0	1	0	0	0	1
mmu-miR-223-3p	52231	Ankzf1	0	0	0	1	0	1
mmu-miR-223-3p	52245	Commd2	0	0	0	1	0	1
mmu-miR-223-3p	52331	Stbd1	0	0	0	1	0	1
mmu-miR-223-3p	52372	D6Ertd527e	0	0	0	1	0	1
mmu-miR-223-3p	52389	Gpr123	0	0	0	1	0	1
mmu-miR-223-3p	52397	Zfp644	0	0	0	1	0	1
mmu-miR-223-3p	52428	Rhpn2	0	0	1	0	0	1
mmu-miR-223-3p	52440	Tax1bp1	0	0	0	1	0	1
mmu-miR-223-3p	52466	Slc46a1	0	0	1	0	0	1
mmu-miR-223-3p	52469	Coa3	1	0	0	0	0	1
mmu-miR-223-3p	52530	Nhp2	0	0	0	1	0	1
mmu-miR-223-3p	52538	Acaa2	1	0	0	0	0	1
mmu-miR-223-3p	52552	Parp8	0	0	0	1	0	1
mmu-miR-223-3p	52635	Esyt2	0	0	0	1	0	1
mmu-miR-223-3p	52639	Wipi1	0	0	0	1	0	1
mmu-miR-223-3p	52662	Ldlrad4	0	0	0	1	0	1
mmu-miR-223-3p	52665	Echdc1	0	0	0	1	0	1
mmu-miR-223-3p	52666	Arhgef25	0	0	0	1	0	1
mmu-miR-223-3p	52670	Cpsf4l	0	0	0	1	0	1
mmu-miR-223-3p	52685	Cd300lg	0	0	0	1	0	1
mmu-miR-223-3p	52686	Mettl2	0	0	0	1	0	1
mmu-miR-223-3p	52700	Txndc17	0	0	0	1	0	1
mmu-miR-223-3p	52708	Zfp410	0	0	0	1	0	1
mmu-miR-223-3p	52710	Slc52a2	0	0	0	1	0	1
mmu-miR-223-3p	52717	Anapc16	0	0	0	1	0	1
mmu-miR-223-3p	52808	Tspyl2	1	0	0	0	0	1

mmu-miR-223-3p	52822	Rufy3	0	0	0	1	0	1
mmu-miR-223-3p	52829	Lurap1l	0	1	0	0	0	1
mmu-miR-223-3p	52857	Gramd1a	0	0	0	1	0	1
mmu-miR-223-3p	52858	Cdipt	0	0	0	1	0	1
mmu-miR-223-3p	52897	Rbfox3	0	0	0	1	0	1
mmu-miR-223-3p	52915	Zmiz2	0	0	0	1	0	1
mmu-miR-223-3p	53312	Nub1	0	0	0	1	0	1
mmu-miR-223-3p	53314	Batf	1	0	0	0	0	1
mmu-miR-223-3p	53315	Sult1d1	0	0	0	1	0	1
mmu-miR-223-3p	53317	Plrg1	0	0	0	1	0	1
mmu-miR-223-3p	53321	Cntnap1	0	0	0	1	0	1
mmu-miR-223-3p	53322	Nucb2	0	0	0	1	0	1
mmu-miR-223-3p	53324	Nptx2	0	1	0	0	0	1
mmu-miR-223-3p	53376	Usp2	0	0	0	1	0	1
mmu-miR-223-3p	53380	Psmd10	0	0	0	1	0	1
mmu-miR-223-3p	53413	Exoc7	0	0	0	1	0	1
mmu-miR-223-3p	53598	Dctn3	0	0	0	1	0	1
mmu-miR-223-3p	53600	Timm23	1	0	0	0	0	1
mmu-miR-223-3p	53602	Hpcal1	0	0	0	1	0	1
mmu-miR-223-3p	53603	Tslp	0	0	0	1	0	1
mmu-miR-223-3p	53614	Reck	0	0	0	1	0	1
mmu-miR-223-3p	53618	Fut8	0	0	0	1	0	1
mmu-miR-223-3p	53625	B3gnt2	0	0	0	1	0	1
mmu-miR-223-3p	53626	Insm1	0	0	0	1	0	1
mmu-miR-223-3p	53791	Tlr5	0	0	0	1	0	1
mmu-miR-223-3p	53858	Rwdd2b	0	0	0	1	0	1
mmu-miR-223-3p	53859	Map3k14	0	0	0	1	0	1
mmu-miR-223-3p	53867	Col5a3	0	0	0	1	0	1
mmu-miR-223-3p	53885	Nphp1	0	0	0	1	0	1
mmu-miR-223-3p	53893	Nudt5	0	0	0	1	0	1
mmu-miR-223-3p	53895	Clpp	1	0	0	0	0	1
mmu-miR-223-3p	53896	Slc7a10	0	0	0	1	0	1
mmu-miR-223-3p	53972	Ngef	0	0	0	1	0	1
mmu-miR-223-3p	53973	Cyp3a41a	0	0	0	1	0	1
mmu-miR-223-3p	54004	Diap2	0	0	0	1	0	1
mmu-miR-223-3p	54124	Cks1b	0	0	0	1	0	1
mmu-miR-223-3p	54125	Polm	0	0	0	1	0	1
mmu-miR-223-3p	54131	Irf3	0	0	0	1	0	1
mmu-miR-223-3p	54135	Lsr	0	0	0	1	0	1
mmu-miR-223-3p	54137	Acrbp	0	0	0	1	0	1
mmu-miR-223-3p	54138	Atxn10	0	0	0	1	0	1
mmu-miR-223-3p	54140	Avpr1a	0	0	1	0	0	1
mmu-miR-223-3p	54150	Rdh7	0	0	0	1	0	1
mmu-miR-223-3p	54160	Copg2	0	0	0	1	0	1
mmu-miR-223-3p	54161	Copg1	0	0	0	1	0	1
mmu-miR-223-3p	54169	Kat6b	0	0	0	1	0	1
mmu-miR-223-3p	54195	Gucy1b3	0	0	0	1	0	1
mmu-miR-223-3p	54196	Pabpn1	1	0	0	0	0	1
mmu-miR-223-3p	54197	Rnf5	0	0	0	1	0	1
mmu-miR-223-3p	54198	Snx3	0	0	0	1	0	1
mmu-miR-223-3p	54218	B3galt4	0	0	0	1	0	1
mmu-miR-223-3p	54219	Cd320	0	0	0	1	0	1
mmu-miR-223-3p	54324	Arhgef5	0	0	0	1	0	1
mmu-miR-223-3p	54325	Elovl1	0	0	0	1	0	1
mmu-miR-223-3p	54343	Atf7ip	0	1	0	0	0	1

mmu-miR-223-3p	54351	Elp5	0	0	0	1	0	1
mmu-miR-223-3p	54352	Irx5	0	0	0	1	0	1
mmu-miR-223-3p	54364	Rpp30	0	0	0	1	0	1
mmu-miR-223-3p	54369	Nme6	0	0	0	1	0	1
mmu-miR-223-3p	54373	Prss16	0	0	1	0	0	1
mmu-miR-223-3p	54375	Azin1	0	0	0	1	0	1
mmu-miR-223-3p	54376	Cacng3	1	0	0	0	0	1
mmu-miR-223-3p	54377	Cacng4	0	1	0	0	0	1
mmu-miR-223-3p	54392	Ncapg	0	0	0	1	0	1
mmu-miR-223-3p	54394	Crlf3	0	0	0	1	0	1
mmu-miR-223-3p	54396	Irgm2	0	0	0	1	0	1
mmu-miR-223-3p	54411	Atp6ap1	0	0	0	1	0	1
mmu-miR-223-3p	54419	Cldn6	0	0	0	1	0	1
mmu-miR-223-3p	54420	Cldn8	0	0	0	1	0	1
mmu-miR-223-3p	54427	Dnmt3l	0	0	0	1	0	1
mmu-miR-223-3p	54445	Unc93b1	0	0	0	1	0	1
mmu-miR-223-3p	54483	Mefv	0	0	0	1	0	1
mmu-miR-223-3p	54485	Dll4	0	0	0	1	0	1
mmu-miR-223-3p	54525	Syt7	0	0	0	1	0	1
mmu-miR-223-3p	54561	Nap1l3	0	0	0	1	0	1
mmu-miR-223-3p	54562	Lrrc6	0	0	0	1	0	1
mmu-miR-223-3p	54609	Ubqln2	0	0	0	1	0	1
mmu-miR-223-3p	54611	Pde3a	0	0	0	1	0	1
mmu-miR-223-3p	54612	Sfrp5	0	0	0	1	0	1
mmu-miR-223-3p	54614	Prpf40b	0	1	0	0	0	1
mmu-miR-223-3p	54615	Npff	0	0	0	1	0	1
mmu-miR-223-3p	54637	Praf2	0	0	0	1	0	1
mmu-miR-223-3p	54644	Otud5	0	0	0	1	0	1
mmu-miR-223-3p	54645	Gripap1	0	0	0	1	0	1
mmu-miR-223-3p	54646	Ppp1r3f	0	0	0	1	0	1
mmu-miR-223-3p	54651	Usp27x	1	0	0	0	0	1
mmu-miR-223-3p	54710	Hs3st3b1	0	0	0	1	0	1
mmu-miR-223-3p	54721	Tyk2	0	0	0	1	0	1
mmu-miR-223-3p	54723	Tfip11	0	0	0	1	0	1
mmu-miR-223-3p	55932	Gbp3	0	0	0	1	0	1
mmu-miR-223-3p	55946	Ap3m1	0	0	0	1	0	1
mmu-miR-223-3p	55950	Bri3	0	0	0	1	0	1
mmu-miR-223-3p	55951	Mpc1	0	0	0	1	0	1
mmu-miR-223-3p	55980	Impa1	0	0	0	1	0	1
mmu-miR-223-3p	55981	Pigb	0	0	0	1	0	1
mmu-miR-223-3p	55987	Cpxm2	0	0	0	1	0	1
mmu-miR-223-3p	55988	Snx12	0	0	0	1	0	1
mmu-miR-223-3p	55989	Nop58	0	0	0	1	0	1
mmu-miR-223-3p	55990	Fmo2	0	0	0	1	0	1
mmu-miR-223-3p	56009	Alyref2	0	0	1	0	0	1
mmu-miR-223-3p	56016	Hebp2	0	0	0	1	0	1
mmu-miR-223-3p	56032	Nprl2	0	0	0	1	0	1
mmu-miR-223-3p	56040	Rplp1	0	0	0	1	0	1
mmu-miR-223-3p	56068	Ammecr1	0	0	0	1	0	1
mmu-miR-223-3p	56075	Pdss1	0	0	0	1	0	1
mmu-miR-223-3p	56079	Astn2	1	0	0	0	0	1
mmu-miR-223-3p	56086	Set	0	0	0	1	0	1
mmu-miR-223-3p	56087	Dnah10	0	0	0	1	0	1
mmu-miR-223-3p	56088	Psmg1	0	0	0	1	0	1
mmu-miR-223-3p	56092	Cts7	0	0	0	1	0	1

mmu-miR-223-3p	56094	Cts8	0	0	0	1	0	1
mmu-miR-223-3p	56096	Plac1	0	0	0	1	0	1
mmu-miR-223-3p	56173	Cldn14	0	0	0	1	0	1
mmu-miR-223-3p	56189	Prodh2	0	0	0	1	0	1
mmu-miR-223-3p	56190	Rbm38	0	0	0	1	0	1
mmu-miR-223-3p	56193	Plek	0	0	0	1	0	1
mmu-miR-223-3p	56199	Abcb10	0	0	1	0	0	1
mmu-miR-223-3p	56209	Gde1	0	0	0	1	0	1
mmu-miR-223-3p	56213	Htra1	0	0	0	1	0	1
mmu-miR-223-3p	56215	Acin1	0	0	0	1	0	1
mmu-miR-223-3p	56218	Patz1	0	0	0	1	0	1
mmu-miR-223-3p	56219	Extl1	0	0	0	1	0	1
mmu-miR-223-3p	56221	Ccl24	0	0	0	1	0	1
mmu-miR-223-3p	56222	Cited4	0	0	0	1	0	1
mmu-miR-223-3p	56223	Fscn3	1	0	0	0	0	1
mmu-miR-223-3p	56226	Espn	0	0	0	1	0	1
mmu-miR-223-3p	56258	Hnrnph2	0	0	0	1	0	1
mmu-miR-223-3p	56275	Rbm14	0	0	0	1	0	1
mmu-miR-223-3p	56277	Tmem45a	0	0	0	1	0	1
mmu-miR-223-3p	56279	Fam69b	0	0	0	1	0	1
mmu-miR-223-3p	56280	Mrpl37	0	0	0	1	0	1
mmu-miR-223-3p	56289	Rassf1	0	0	0	1	0	1
mmu-miR-223-3p	56293	Slc35g3	0	0	0	1	0	1
mmu-miR-223-3p	56296	Dmrtb1	0	0	0	1	0	1
mmu-miR-223-3p	56297	Arl6	0	0	0	1	0	1
mmu-miR-223-3p	56316	Ggcx	0	0	0	1	0	1
mmu-miR-223-3p	56317	Anapc7	0	0	0	1	0	1
mmu-miR-223-3p	56327	Arl2	0	0	0	1	0	1
mmu-miR-223-3p	56338	Txnip	0	0	0	1	0	1
mmu-miR-223-3p	56348	Hsd17b12	0	0	0	1	0	1
mmu-miR-223-3p	56349	Net1	0	0	0	1	0	1
mmu-miR-223-3p	56361	Pus1	0	0	0	1	0	1
mmu-miR-223-3p	56367	Scoc	0	0	0	1	0	1
mmu-miR-223-3p	56369	Apip	1	0	0	0	0	1
mmu-miR-223-3p	56371	Fzr1	0	0	0	1	0	1
mmu-miR-223-3p	56373	Cpb2	0	0	0	1	0	1
mmu-miR-223-3p	56380	Arid3b	0	0	0	1	0	1
mmu-miR-223-3p	56392	Shoc2	0	0	0	1	0	1
mmu-miR-223-3p	56397	Morf4l2	0	0	0	1	0	1
mmu-miR-223-3p	56401	Lepre1	0	0	0	1	0	1
mmu-miR-223-3p	56407	Trpc4ap	0	0	0	1	0	1
mmu-miR-223-3p	56417	Adar	0	0	0	1	0	1
mmu-miR-223-3p	56421	Pfkp	0	0	0	1	0	1
mmu-miR-223-3p	56422	Hbs1l	0	0	0	1	0	1
mmu-miR-223-3p	56424	Stub1	0	0	0	1	0	1
mmu-miR-223-3p	56427	Tubd1	1	0	0	0	0	1
mmu-miR-223-3p	56429	Dpt	0	0	0	1	0	1
mmu-miR-223-3p	56431	Dstn	0	0	0	1	0	1
mmu-miR-223-3p	56433	Vps29	0	0	0	1	0	1
mmu-miR-223-3p	56434	Tspan3	0	0	0	1	0	1
mmu-miR-223-3p	56436	Adrm1	0	0	0	1	0	1
mmu-miR-223-3p	56437	Rrad	0	0	0	1	0	1
mmu-miR-223-3p	56443	Arpc1a	0	0	0	1	0	1
mmu-miR-223-3p	56444	Actr10	0	0	0	1	0	1
mmu-miR-223-3p	56447	Copz1	0	0	0	1	0	1

mmu-miR-223-3p	56448	Cyp2d22	0	0	0	1	0	1
mmu-miR-223-3p	56449	Ybx3	0	0	0	1	0	1
mmu-miR-223-3p	56453	Mbtps1	0	0	0	1	0	1
mmu-miR-223-3p	56454	Aldh18a1	0	0	0	1	0	1
mmu-miR-223-3p	56455	Dynll1	0	0	0	1	0	1
mmu-miR-223-3p	56460	Pkp3	0	0	0	1	0	1
mmu-miR-223-3p	56462	Mtch1	0	0	0	1	0	1
mmu-miR-223-3p	56463	Snd1	0	0	0	1	0	1
mmu-miR-223-3p	56464	Ctsf	0	0	0	1	0	1
mmu-miR-223-3p	56469	Pias1	0	0	1	0	0	1
mmu-miR-223-3p	56470	Rgs19	0	0	0	1	0	1
mmu-miR-223-3p	56480	Tbk1	0	0	0	1	0	1
mmu-miR-223-3p	56486	Gabarap	0	0	0	1	0	1
mmu-miR-223-3p	56488	Nxt1	0	0	0	1	0	1
mmu-miR-223-3p	56505	Ruvbl1	1	0	0	0	0	1
mmu-miR-223-3p	56525	Zfp235	0	0	0	1	0	1
mmu-miR-223-3p	56529	Sec11a	0	0	0	1	0	1
mmu-miR-223-3p	56531	Ylpm1	0	0	0	1	0	1
mmu-miR-223-3p	56535	Pex3	0	0	0	1	0	1
mmu-miR-223-3p	56538	Klk11	0	0	0	1	0	1
mmu-miR-223-3p	56551	Txn2	0	0	0	1	0	1
mmu-miR-223-3p	56554	Raet1d	0	0	0	1	0	1
mmu-miR-223-3p	56615	Mgst1	0	0	0	1	0	1
mmu-miR-223-3p	56620	Clec4n	1	0	0	0	0	1
mmu-miR-223-3p	56632	Sphk2	0	0	0	1	0	1
mmu-miR-223-3p	56642	Ankrd2	0	0	0	1	0	1
mmu-miR-223-3p	56644	Clec7a	0	0	0	1	0	1
mmu-miR-223-3p	56691	Dnajb8	0	0	0	1	0	1
mmu-miR-223-3p	56693	Crtap	0	0	0	1	0	1
mmu-miR-223-3p	56697	Akap10	0	0	0	1	0	1
mmu-miR-223-3p	56698	Phax	0	0	0	1	0	1
mmu-miR-223-3p	56699	Cdc42ep4	0	0	0	1	0	1
mmu-miR-223-3p	56703	Pigo	0	0	0	1	0	1
mmu-miR-223-3p	56705	Ranbp9	0	0	0	1	0	1
mmu-miR-223-3p	56722	Litaf	0	0	0	1	0	1
mmu-miR-223-3p	56724	Cript	0	0	0	1	0	1
mmu-miR-223-3p	56737	Alg2	0	0	0	1	0	1
mmu-miR-223-3p	56742	Psrc1	0	0	0	1	0	1
mmu-miR-223-3p	56743	Lat2	0	0	0	1	0	1
mmu-miR-223-3p	56748	Nfu1	0	0	0	1	0	1
mmu-miR-223-3p	56753	Tacstd2	0	0	0	1	0	1
mmu-miR-223-3p	56772	Mllt11	0	0	0	1	0	1
mmu-miR-223-3p	56773	Chst5	0	0	0	1	0	1
mmu-miR-223-3p	56774	Slc6a14	0	0	0	1	0	1
mmu-miR-223-3p	56790	Supt20	0	0	0	1	0	1
mmu-miR-223-3p	56791	Ube2l6	0	0	0	1	0	1
mmu-miR-223-3p	56812	Dnajb2	0	0	0	1	0	1
mmu-miR-223-3p	56838	Ccl28	0	0	0	1	0	1
mmu-miR-223-3p	56843	Trpm5	0	0	1	0	0	1
mmu-miR-223-3p	56844	Tssc4	0	0	0	1	0	1
mmu-miR-223-3p	56846	Necab3	0	0	0	1	0	1
mmu-miR-223-3p	56856	Insm2	0	0	0	1	0	1
mmu-miR-223-3p	56863	Cldn9	0	0	0	1	0	1
mmu-miR-223-3p	56868	Psg23	0	0	0	1	0	1
mmu-miR-223-3p	56872	Pate4	0	0	0	1	0	1

mmu-miR-223-3p	56876	Nsmf	0	0	0	1	0	1
mmu-miR-223-3p	56878	Rbms1	0	0	0	1	0	1
mmu-miR-223-3p	57014	Htr3b	0	0	0	1	0	1
mmu-miR-223-3p	57028	Pdxp	0	0	0	1	0	1
mmu-miR-223-3p	57080	Gtf2ird1	0	0	0	1	0	1
mmu-miR-223-3p	57170	Dolpp1	0	1	0	0	0	1
mmu-miR-223-3p	57250	Olfr653	0	0	0	1	0	1
mmu-miR-223-3p	57258	Xpo4	0	1	0	0	0	1
mmu-miR-223-3p	57264	Retn	0	0	0	1	0	1
mmu-miR-223-3p	57265	Fzd2	0	0	0	1	0	1
mmu-miR-223-3p	57267	Apba3	0	0	0	1	0	1
mmu-miR-223-3p	57270	Olfr1508	0	0	0	1	0	1
mmu-miR-223-3p	57279	Slc25a20	0	0	0	1	0	1
mmu-miR-223-3p	57312	Mrps31	0	0	0	1	0	1
mmu-miR-223-3p	57319	Smpdl3a	0	0	0	1	0	1
mmu-miR-223-3p	57320	Park7	0	0	0	1	0	1
mmu-miR-223-3p	57330	Gigyf1	0	0	0	1	0	1
mmu-miR-223-3p	57339	Jph1	0	0	0	1	0	1
mmu-miR-223-3p	57349	Ppbp	1	0	0	0	0	1
mmu-miR-223-3p	57373	Akip1	0	0	0	1	0	1
mmu-miR-223-3p	57376	Smarce1	0	0	0	1	0	1
mmu-miR-223-3p	57429	Sult5a1	0	0	0	1	0	1
mmu-miR-223-3p	57431	Dnajc4	0	0	0	1	0	1
mmu-miR-223-3p	57432	Zc3h8	0	0	0	1	0	1
mmu-miR-223-3p	57435	Plin4	0	0	1	0	0	1
mmu-miR-223-3p	57436	Gabarapl1	0	0	0	1	0	1
mmu-miR-223-3p	57437	Golga7	0	0	0	1	0	1
mmu-miR-223-3p	57439	Tmem183a	0	0	0	1	0	1
mmu-miR-223-3p	57442	Kcne3	0	0	0	1	0	1
mmu-miR-223-3p	57741	Noc2l	0	0	0	1	0	1
mmu-miR-223-3p	57746	Piwil2	0	0	0	1	0	1
mmu-miR-223-3p	57752	Tacc2	0	0	0	1	0	1
mmu-miR-223-3p	57753	Noc3l	0	0	0	1	0	1
mmu-miR-223-3p	57757	Pglyrp2	0	0	0	1	0	1
mmu-miR-223-3p	57765	Tbx21	0	0	0	1	0	1
mmu-miR-223-3p	57776	Ttyh1	0	0	0	1	0	1
mmu-miR-223-3p	57782	Rbak	0	0	0	1	0	1
mmu-miR-223-3p	57869	Adck2	0	0	0	1	0	1
mmu-miR-223-3p	57895	Ccdc126	0	0	0	1	0	1
mmu-miR-223-3p	57905	Isy1	0	0	0	1	0	1
mmu-miR-223-3p	57911	Gsdma	0	0	0	1	0	1
mmu-miR-223-3p	58170	Asic5	0	0	0	1	0	1
mmu-miR-223-3p	58175	Rgs20	0	0	0	1	0	1
mmu-miR-223-3p	58184	Rqcd1	0	0	0	1	0	1
mmu-miR-223-3p	58186	Rad18	0	0	0	1	0	1
mmu-miR-223-3p	58198	Sall1	1	0	0	0	0	1
mmu-miR-223-3p	58203	Zbp1	0	0	0	1	0	1
mmu-miR-223-3p	58208	Bcl11b	0	0	0	1	0	1
mmu-miR-223-3p	58210	Sectm1b	0	0	0	1	0	1
mmu-miR-223-3p	58212	Srrm3	1	0	0	0	0	1
mmu-miR-223-3p	58214	Cst10	0	0	0	1	0	1
mmu-miR-223-3p	58222	Rab37	0	0	0	1	0	1
mmu-miR-223-3p	58229	Efcc1	0	0	0	1	0	1
mmu-miR-223-3p	58230	Rnf8	0	0	0	1	0	1
mmu-miR-223-3p	58233	Dnaja4	0	0	1	0	0	1

mmu-miR-223-3p	58234	Shank3	0	0	0	1	0	1
mmu-miR-223-3p	58237	Nkain4	0	0	0	1	0	1
mmu-miR-223-3p	58239	Dexi	0	0	0	1	0	1
mmu-miR-223-3p	58245	Gpr180	0	0	0	1	0	1
mmu-miR-223-3p	58246	Slc35b4	0	0	0	1	0	1
mmu-miR-223-3p	58248	1700123O20Rik	1	0	0	0	0	1
mmu-miR-223-3p	58523	Elp2	0	0	0	1	0	1
mmu-miR-223-3p	58800	Trpm7	0	0	0	1	0	1
mmu-miR-223-3p	58803	Pga5	0	0	0	1	0	1
mmu-miR-223-3p	58805	Mlxipl	0	0	0	1	0	1
mmu-miR-223-3p	58864	Tssk3	1	0	0	0	0	1
mmu-miR-223-3p	58865	Tdh	0	0	0	1	0	1
mmu-miR-223-3p	58866	Treh	1	0	0	0	0	1
mmu-miR-223-3p	58988	Rps6kb2	0	1	0	0	0	1
mmu-miR-223-3p	59002	Wrap73	0	0	0	1	0	1
mmu-miR-223-3p	59007	Ngly1	0	0	0	1	0	1
mmu-miR-223-3p	59016	Thap11	0	0	0	1	0	1
mmu-miR-223-3p	59020	Pdzk1	0	0	0	1	0	1
mmu-miR-223-3p	59022	Edf1	0	0	0	1	0	1
mmu-miR-223-3p	59030	Mkks	0	0	0	1	0	1
mmu-miR-223-3p	59036	Dact1	0	0	0	1	0	1
mmu-miR-223-3p	59038	Pxmp4	0	0	0	1	0	1
mmu-miR-223-3p	59043	Wsb2	0	0	0	1	0	1
mmu-miR-223-3p	59044	Rnf130	0	0	0	1	0	1
mmu-miR-223-3p	59045	Stard3	0	0	0	1	0	1
mmu-miR-223-3p	59052	Mettl9	0	0	0	1	0	1
mmu-miR-223-3p	59053	Fam203a	0	0	0	1	0	1
mmu-miR-223-3p	59054	Mrps30	0	0	0	1	0	1
mmu-miR-223-3p	59056	Evc	0	0	0	1	0	1
mmu-miR-223-3p	59090	Midn	0	0	0	1	0	1
mmu-miR-223-3p	59091	Jph2	0	0	0	1	0	1
mmu-miR-223-3p	59092	Pcbp4	0	0	0	1	0	1
mmu-miR-223-3p	59095	Fxyd6	0	0	0	1	0	1
mmu-miR-223-3p	59125	Nek7	0	0	0	1	0	1
mmu-miR-223-3p	59288	Dctn5	0	0	0	1	0	1
mmu-miR-223-3p	59289	Ackr2	0	0	0	1	0	1
mmu-miR-223-3p	60321	Wbp11	0	0	0	1	0	1
mmu-miR-223-3p	60322	Chst7	0	0	0	1	0	1
mmu-miR-223-3p	60345	Nrip2	0	0	0	1	0	1
mmu-miR-223-3p	60441	Mrpl38	0	0	0	1	0	1
mmu-miR-223-3p	60455	Tmem8	0	0	0	1	0	1
mmu-miR-223-3p	60527	Fads3	0	0	0	1	0	1
mmu-miR-223-3p	60534	Fancg	0	0	0	1	0	1
mmu-miR-223-3p	60594	Capn12	0	0	0	1	0	1
mmu-miR-223-3p	60595	Actn4	0	0	0	1	0	1
mmu-miR-223-3p	63856	Taf8	0	0	1	0	0	1
mmu-miR-223-3p	63873	Trpv4	0	0	0	1	0	1
mmu-miR-223-3p	63954	Rbp7	0	0	0	1	0	1
mmu-miR-223-3p	63986	Gmfg	0	0	0	1	0	1
mmu-miR-223-3p	63993	Slc5a7	0	0	0	1	0	1
mmu-miR-223-3p	64010	Sav1	0	0	0	1	0	1
mmu-miR-223-3p	64011	Nrgn	0	0	0	1	0	1
mmu-miR-223-3p	64050	Yeats4	0	0	0	1	0	1
mmu-miR-223-3p	64058	Perp	0	0	0	1	0	1
mmu-miR-223-3p	64059	Oxct2a	0	0	0	1	0	1

mmu-miR-223-3p	64074	Smoc2	0	0	0	1	0	1
mmu-miR-223-3p	64082	Popdc2	0	0	0	1	0	1
mmu-miR-223-3p	64113	Moap1	0	0	0	1	0	1
mmu-miR-223-3p	64139	Ctsm	0	0	0	1	0	1
mmu-miR-223-3p	64291	Osbpl1a	0	0	0	1	0	1
mmu-miR-223-3p	64293	Stk32b	0	0	1	0	0	1
mmu-miR-223-3p	64294	Itm2c	0	0	0	1	0	1
mmu-miR-223-3p	64295	Tmub1	0	0	0	1	0	1
mmu-miR-223-3p	64379	Irx6	1	0	0	0	0	1
mmu-miR-223-3p	64380	Ms4a4c	0	0	0	1	0	1
mmu-miR-223-3p	64383	Sirt2	0	0	0	1	0	1
mmu-miR-223-3p	64384	Sirt3	0	0	0	1	0	1
mmu-miR-223-3p	64424	Polr1e	0	0	0	1	0	1
mmu-miR-223-3p	64436	Inpp5e	0	0	0	1	0	1
mmu-miR-223-3p	64453	Zfp280b	0	0	0	1	0	1
mmu-miR-223-3p	64540	Tspan4	0	0	0	1	0	1
mmu-miR-223-3p	64580	Ndst4	0	0	0	1	0	1
mmu-miR-223-3p	64655	Mrps22	0	0	0	1	0	1
mmu-miR-223-3p	64657	Mrps10	0	0	0	1	0	1
mmu-miR-223-3p	64697	Keg1	0	0	0	1	0	1
mmu-miR-223-3p	64707	Suv39h2	0	0	0	1	0	1
mmu-miR-223-3p	64818	Krt81	0	0	0	1	0	1
mmu-miR-223-3p	64899	Lpin3	0	0	0	1	0	1
mmu-miR-223-3p	64918	Bhmt2	0	0	0	1	0	1
mmu-miR-223-3p	64931	Folr4	0	0	0	1	0	1
mmu-miR-223-3p	64933	Ap3m2	0	0	0	1	0	1
mmu-miR-223-3p	65098	Zfand6	0	0	0	1	0	1
mmu-miR-223-3p	65099	Irak1bp1	0	0	0	1	0	1
mmu-miR-223-3p	65103	Arl6ip6	0	0	0	1	0	1
mmu-miR-223-3p	65105	Arl6ip4	0	0	0	1	0	1
mmu-miR-223-3p	65111	Dap3	0	0	0	1	0	1
mmu-miR-223-3p	65114	Vps35	0	0	0	1	0	1
mmu-miR-223-3p	65116	Prrg2	0	0	0	1	0	1
mmu-miR-223-3p	65255	Asb4	0	0	1	0	0	1
mmu-miR-223-3p	65256	Asb2	0	0	0	1	0	1
mmu-miR-223-3p	65945	Clstn1	0	0	0	1	0	1
mmu-miR-223-3p	65962	Slc9a3r2	0	0	0	1	0	1
mmu-miR-223-3p	65969	Cubn	0	0	0	1	0	1
mmu-miR-223-3p	65971	Tbata	0	0	0	1	0	1
mmu-miR-223-3p	66011	Ranbp17	0	0	1	0	0	1
mmu-miR-223-3p	66039	Tmem254a	0	0	0	1	0	1
mmu-miR-223-3p	66042	Sostdc1	0	0	0	1	0	1
mmu-miR-223-3p	66043	Atp5d	1	0	0	0	0	1
mmu-miR-223-3p	66044	Dtd1	0	0	0	1	0	1
mmu-miR-223-3p	66046	Ndufb5	0	0	0	1	0	1
mmu-miR-223-3p	66048	Emc6	0	0	0	1	0	1
mmu-miR-223-3p	66049	Rogdi	0	0	0	1	0	1
mmu-miR-223-3p	66053	Ppil2	0	0	0	1	0	1
mmu-miR-223-3p	66056	Zfp524	0	0	0	1	0	1
mmu-miR-223-3p	66058	Tmem176a	0	0	0	1	0	1
mmu-miR-223-3p	66061	Tctex1d2	0	0	0	1	0	1
mmu-miR-223-3p	66065	Hsd17b14	0	0	0	1	0	1
mmu-miR-223-3p	66069	Snupn	0	0	0	1	0	1
mmu-miR-223-3p	66079	Tmem42	0	0	0	1	0	1
mmu-miR-223-3p	66083	Setd6	0	0	0	1	0	1

mmu-miR-223-3p	66084	Rmnd1	0	0	0	1	0	1
mmu-miR-223-3p	66085	Eif3f	0	0	0	1	0	1
mmu-miR-223-3p	66087	Emc3	0	0	0	1	0	1
mmu-miR-223-3p	66089	Rmnd5b	0	0	0	1	0	1
mmu-miR-223-3p	66090	Ypel3	0	0	0	1	0	1
mmu-miR-223-3p	66091	Ndufa3	0	0	0	1	0	1
mmu-miR-223-3p	66092	Ghitm	0	0	0	1	0	1
mmu-miR-223-3p	66098	Chchd6	0	0	0	1	0	1
mmu-miR-223-3p	66102	Cxcl16	0	0	0	1	0	1
mmu-miR-223-3p	66104	Tceal6	0	0	0	1	0	1
mmu-miR-223-3p	66106	Smpx	0	0	0	1	0	1
mmu-miR-223-3p	66108	Ndufa9	0	0	0	1	0	1
mmu-miR-223-3p	66109	Tspan13	0	0	0	1	0	1
mmu-miR-223-3p	66111	Tmed3	0	0	0	1	0	1
mmu-miR-223-3p	66114	Dnajc30	0	0	0	1	0	1
mmu-miR-223-3p	66116	Cml1	0	0	0	1	0	1
mmu-miR-223-3p	66119	Tomm6	0	0	0	1	0	1
mmu-miR-223-3p	66125	Sf3b5	0	0	0	1	0	1
mmu-miR-223-3p	66132	1110008L16Rik	0	0	0	1	0	1
mmu-miR-223-3p	66139	Tmem8c	0	0	0	1	0	1
mmu-miR-223-3p	66142	Cox7b	0	0	0	1	0	1
mmu-miR-223-3p	66143	Eef1e1	0	0	0	1	0	1
mmu-miR-223-3p	66144	Atp6v1f	0	0	0	1	0	1
mmu-miR-223-3p	66151	Prr13	0	0	0	1	0	1
mmu-miR-223-3p	66154	Tmem14c	0	0	0	1	0	1
mmu-miR-223-3p	66156	Anapc11	0	0	0	1	0	1
mmu-miR-223-3p	66168	Grina	0	0	0	1	0	1
mmu-miR-223-3p	66170	Chchd5	0	0	0	1	0	1
mmu-miR-223-3p	66172	Med11	0	0	0	1	0	1
mmu-miR-223-3p	66177	Ubl5	0	0	0	1	0	1
mmu-miR-223-3p	66179	Ogfod3	0	0	0	1	0	1
mmu-miR-223-3p	66181	Nop10	0	0	0	1	0	1
mmu-miR-223-3p	66194	Pycrl	0	0	0	1	0	1
mmu-miR-223-3p	66196	Myo19	0	0	0	1	0	1
mmu-miR-223-3p	66203	Lce1m	0	1	0	0	0	1
mmu-miR-223-3p	66206	1110059E24Rik	0	0	0	1	0	1
mmu-miR-223-3p	66213	Med7	0	0	0	1	0	1
mmu-miR-223-3p	66222	Serpinb1a	0	0	0	1	0	1
mmu-miR-223-3p	66223	Mrpl35	0	0	0	1	0	1
mmu-miR-223-3p	66231	Thoc7	0	0	0	1	0	1
mmu-miR-223-3p	66233	Dmap1	0	0	0	1	0	1
mmu-miR-223-3p	66234	Sc4mol	0	0	0	1	0	1
mmu-miR-223-3p	66245	Hspbp1	0	0	0	1	0	1
mmu-miR-223-3p	66246	Osgep	0	0	0	1	0	1
mmu-miR-223-3p	66248	Alg5	0	0	0	1	0	1
mmu-miR-223-3p	66249	Pno1	1	0	0	0	0	1
mmu-miR-223-3p	66254	Dimt1	0	0	0	1	0	1
mmu-miR-223-3p	66258	Mrps17	0	0	0	1	0	1
mmu-miR-223-3p	66260	Tmem54	0	0	0	1	0	1
mmu-miR-223-3p	66264	Ccdc28b	1	0	0	0	0	1
mmu-miR-223-3p	66266	Eapp	0	1	0	0	0	1
mmu-miR-223-3p	66273	Aamdc	0	0	0	1	0	1
mmu-miR-223-3p	66277	Klf15	0	0	0	1	0	1
mmu-miR-223-3p	66278	Smim20	0	0	0	1	0	1
mmu-miR-223-3p	66279	Tmem218	0	0	0	1	0	1

mmu-miR-223-3p	66282	Tma16	0	0	0	1	0	1
mmu-miR-223-3p	66283	Gkn1	1	0	0	0	0	1
mmu-miR-223-3p	66284	Gkn2	0	0	0	1	0	1
mmu-miR-223-3p	66290	Atp6v1g1	0	0	0	1	0	1
mmu-miR-223-3p	66296	Haus2	0	0	0	1	0	1
mmu-miR-223-3p	66298	Defa21	0	0	0	1	0	1
mmu-miR-223-3p	66300	Prr24	0	0	0	1	0	1
mmu-miR-223-3p	66302	Rmdn1	0	0	0	1	0	1
mmu-miR-223-3p	66308	Mplkip	0	0	0	1	0	1
mmu-miR-223-3p	66317	Wdr61	0	0	0	1	0	1
mmu-miR-223-3p	66326	Dnajc5b	0	0	0	1	0	1
mmu-miR-223-3p	66328	Scp2d1	0	0	0	1	0	1
mmu-miR-223-3p	66336	Cenpp	0	0	0	1	0	1
mmu-miR-223-3p	66337	Fam229b	0	0	0	1	0	1
mmu-miR-223-3p	66340	Psenen	0	0	0	1	0	1
mmu-miR-223-3p	66341	Eid3	1	0	0	0	0	1
mmu-miR-223-3p	66346	1700029P11Rik	0	0	0	1	0	1
mmu-miR-223-3p	66354	Snw1	0	0	0	1	0	1
mmu-miR-223-3p	66355	Gmpr	0	0	0	1	0	1
mmu-miR-223-3p	66362	Exosc3	0	0	0	1	0	1
mmu-miR-223-3p	66369	Dus2l	0	0	0	1	0	1
mmu-miR-223-3p	66374	2310011J03Rik	0	0	0	1	0	1
mmu-miR-223-3p	66380	Krtap3-3	0	0	0	1	0	1
mmu-miR-223-3p	66383	Iscu	0	0	0	1	0	1
mmu-miR-223-3p	66384	Srp19	1	0	0	0	0	1
mmu-miR-223-3p	66390	Slmo2	0	0	0	1	0	1
mmu-miR-223-3p	66394	Nosip	0	0	0	1	0	1
mmu-miR-223-3p	66395	Ahnak	1	0	0	0	0	1
mmu-miR-223-3p	66396	Ccdc82	0	0	0	1	0	1
mmu-miR-223-3p	66398	Commd5	0	0	0	1	0	1
mmu-miR-223-3p	66400	Alkbh7	0	0	0	1	0	1
mmu-miR-223-3p	66401	Nudt2	0	0	0	1	0	1
mmu-miR-223-3p	66403	Asf1a	0	0	0	1	0	1
mmu-miR-223-3p	66404	Rtfdc1	0	0	0	1	0	1
mmu-miR-223-3p	66409	Rsl1d1	1	0	0	0	0	1
mmu-miR-223-3p	66410	Mterfd1	0	1	0	0	0	1
mmu-miR-223-3p	66420	Polr2e	0	0	1	0	0	1
mmu-miR-223-3p	66432	Slc7a6os	0	0	0	1	0	1
mmu-miR-223-3p	66433	Chchd7	0	1	0	0	0	1
mmu-miR-223-3p	66446	Exosc7	0	0	0	1	0	1
mmu-miR-223-3p	66447	Mgst3	0	0	0	1	0	1
mmu-miR-223-3p	66448	Mrpl20	1	0	0	0	0	1
mmu-miR-223-3p	66454	Nmnat1	0	1	0	0	0	1
mmu-miR-223-3p	66455	Cnpy4	0	0	0	1	0	1
mmu-miR-223-3p	66461	Ptpmt1	0	0	0	1	0	1
mmu-miR-223-3p	66462	2810428I15Rik	0	0	0	1	0	1
mmu-miR-223-3p	66477	Usmg5	0	0	0	1	0	1
mmu-miR-223-3p	66489	Rpl35	0	0	0	1	0	1
mmu-miR-223-3p	66492	Zmat2	0	0	0	1	0	1
mmu-miR-223-3p	66498	Dda1	0	0	0	1	0	1
mmu-miR-223-3p	66501	1700029H14Rik	0	0	0	1	0	1
mmu-miR-223-3p	66505	Zmynd11	0	0	0	1	0	1
mmu-miR-223-3p	66508	Lamtor1	0	0	0	1	0	1
mmu-miR-223-3p	66513	Tab1	0	0	0	1	0	1
mmu-miR-223-3p	66530	Ubxn6	0	0	0	1	0	1

mmu-miR-223-3p	66531	Cmc2	0	0	0	1	0	1
mmu-miR-223-3p	66532	Rep15	0	0	0	1	0	1
mmu-miR-223-3p	66533	2310050C09Rik	1	0	0	0	0	1
mmu-miR-223-3p	66540	Fam107b	0	0	0	1	0	1
mmu-miR-223-3p	66548	Adamtsl5	0	0	0	1	0	1
mmu-miR-223-3p	66566	Ntpcr	0	0	0	1	0	1
mmu-miR-223-3p	66568	Rwdd3	1	0	0	0	0	1
mmu-miR-223-3p	66580	Esf1	0	0	0	1	0	1
mmu-miR-223-3p	66583	Exosc1	0	0	0	1	0	1
mmu-miR-223-3p	66590	Farsa	0	0	0	1	0	1
mmu-miR-223-3p	66591	Mad2l1bp	0	0	0	1	0	1
mmu-miR-223-3p	66592	Stoml2	0	0	0	1	0	1
mmu-miR-223-3p	66594	Uqcr11	0	0	0	1	0	1
mmu-miR-223-3p	66595	Aste1	0	0	0	1	0	1
mmu-miR-223-3p	66598	3110001I22Rik	0	0	0	1	0	1
mmu-miR-223-3p	66603	Gemin2	0	0	0	1	0	1
mmu-miR-223-3p	66606	Lrrc57	0	0	0	1	0	1
mmu-miR-223-3p	66609	Cryzl1	0	0	0	1	0	1
mmu-miR-223-3p	66610	Abi3	0	0	0	1	0	1
mmu-miR-223-3p	66612	Ormdl3	0	0	0	1	0	1
mmu-miR-223-3p	66614	Gpatch4	0	0	0	1	0	1
mmu-miR-223-3p	66617	Ntmt1	0	0	0	1	0	1
mmu-miR-223-3p	66618	Snrnp27	1	0	0	0	0	1
mmu-miR-223-3p	66628	Thg1l	1	0	0	0	0	1
mmu-miR-223-3p	66629	Golph3	0	0	0	1	0	1
mmu-miR-223-3p	66631	Hiatl1	0	0	0	1	0	1
mmu-miR-223-3p	66637	Tsen15	0	0	0	1	0	1
mmu-miR-223-3p	66641	Sike1	0	0	0	1	0	1
mmu-miR-223-3p	66656	Eef1d	0	0	0	1	0	1
mmu-miR-223-3p	66658	Ccdc51	0	0	0	1	0	1
mmu-miR-223-3p	66659	Acp6	0	0	0	1	0	1
mmu-miR-223-3p	66664	Tmem41a	0	0	0	1	0	1
mmu-miR-223-3p	66671	Ccnh	1	0	0	0	0	1
mmu-miR-223-3p	66679	Rae1	1	0	0	0	0	1
mmu-miR-223-3p	66680	Oser1	0	0	0	1	0	1
mmu-miR-223-3p	66681	Pgm1	1	0	0	0	0	1
mmu-miR-223-3p	66682	Trappc5	0	0	0	1	0	1
mmu-miR-223-3p	66686	Dcbld1	0	0	0	1	0	1
mmu-miR-223-3p	66687	Tbc1d15	0	0	0	1	0	1
mmu-miR-223-3p	66694	Uqcrfs1	0	0	0	1	0	1
mmu-miR-223-3p	66704	Rbm4b	0	0	0	1	0	1
mmu-miR-223-3p	66705	Dnase1l2	0	0	0	1	0	1
mmu-miR-223-3p	66708	Krtap3-2	0	0	0	1	0	1
mmu-miR-223-3p	66712	Spesp1	0	0	0	1	0	1
mmu-miR-223-3p	66715	Henmt1	0	0	0	1	0	1
mmu-miR-223-3p	66716	Ccer1	0	0	0	1	0	1
mmu-miR-223-3p	66722	Spag16	0	0	0	1	0	1
mmu-miR-223-3p	66732	4921530L21Rik	0	0	0	1	0	1
mmu-miR-223-3p	66734	Map1lc3a	0	0	0	1	0	1
mmu-miR-223-3p	66736	Emc2	0	0	0	1	0	1
mmu-miR-223-3p	66742	Cypt1	1	0	0	0	0	1
mmu-miR-223-3p	66745	Trpd52l3	0	0	0	1	0	1
mmu-miR-223-3p	66758	Zfp474	0	0	0	1	0	1
mmu-miR-223-3p	66761	4933417A18Rik	0	0	0	1	0	1
mmu-miR-223-3p	66763	4933425L06Rik	0	0	0	1	0	1

mmu-miR-223-3p	66766	Tmem239	0	0	0	1	0	1
mmu-miR-223-3p	66768	Pacrgl	0	0	0	1	0	1
mmu-miR-223-3p	66773	Gm17019	0	0	0	1	0	1
mmu-miR-223-3p	66789	Alg14	1	0	0	0	0	1
mmu-miR-223-3p	66790	Grtp1	1	0	0	0	0	1
mmu-miR-223-3p	66793	Efcab1	0	1	0	0	0	1
mmu-miR-223-3p	66808	9030624G23Rik	0	0	0	1	0	1
mmu-miR-223-3p	66813	Bcl2l14	0	0	0	1	0	1
mmu-miR-223-3p	66815	Ccdc109b	0	0	0	1	0	1
mmu-miR-223-3p	66824	Pycard	0	0	0	1	0	1
mmu-miR-223-3p	66825	Rnf186	0	0	0	1	0	1
mmu-miR-223-3p	66832	Rsph3a	1	0	0	0	0	1
mmu-miR-223-3p	66840	Wdr45b	0	0	0	1	0	1
mmu-miR-223-3p	66841	Etfdh	0	0	0	1	0	1
mmu-miR-223-3p	66845	Mrpl33	0	0	0	1	0	1
mmu-miR-223-3p	66853	Pnpla2	0	0	0	1	0	1
mmu-miR-223-3p	66859	Slc16a9	0	0	0	1	0	1
mmu-miR-223-3p	66865	Pmpca	1	0	0	0	0	1
mmu-miR-223-3p	66869	Zfp869	0	0	0	1	0	1
mmu-miR-223-3p	66871	Cpne8	0	0	0	1	0	1
mmu-miR-223-3p	66881	Pcyox1	0	0	0	1	0	1
mmu-miR-223-3p	66884	Appbp2	0	0	0	1	0	1
mmu-miR-223-3p	66887	Lonp2	0	0	0	1	0	1
mmu-miR-223-3p	66890	Lman2	0	0	0	1	0	1
mmu-miR-223-3p	66894	Wwp2	0	0	0	1	0	1
mmu-miR-223-3p	66895	Pxdc1	0	0	0	1	0	1
mmu-miR-223-3p	66898	Baiap2l1	0	0	0	1	0	1
mmu-miR-223-3p	66901	Proz	0	0	0	1	0	1
mmu-miR-223-3p	66904	Pccb	0	0	0	1	0	1
mmu-miR-223-3p	66905	Plin3	0	0	0	1	0	1
mmu-miR-223-3p	66910	Tmem107	0	0	0	1	0	1
mmu-miR-223-3p	66913	Kdelr2	0	0	0	1	0	1
mmu-miR-223-3p	66915	Myeov2	0	0	0	1	0	1
mmu-miR-223-3p	66921	Prpf38b	0	0	0	1	0	1
mmu-miR-223-3p	66928	Tmem261	0	0	0	1	0	1
mmu-miR-223-3p	66931	1700010I14Rik	0	0	0	1	0	1
mmu-miR-223-3p	66938	Sh3d21	1	0	0	0	0	1
mmu-miR-223-3p	66940	Shisa5	0	0	0	1	0	1
mmu-miR-223-3p	66945	Sdha	0	0	1	0	0	1
mmu-miR-223-3p	66957	Serpinb11	0	0	0	1	0	1
mmu-miR-223-3p	66958	Tmx2	0	0	0	1	0	1
mmu-miR-223-3p	66965	Ctu2	1	0	0	0	0	1
mmu-miR-223-3p	66966	Trit1	0	0	0	1	0	1
mmu-miR-223-3p	66968	Plin5	0	0	0	1	0	1
mmu-miR-223-3p	66975	Trappc13	0	0	0	1	0	1
mmu-miR-223-3p	66980	Zdhhc6	0	0	0	1	0	1
mmu-miR-223-3p	66989	Kctd20	1	0	0	0	0	1
mmu-miR-223-3p	66990	Tmem134	0	0	0	1	0	1
mmu-miR-223-3p	66994	Cep19	0	0	0	1	0	1
mmu-miR-223-3p	67003	Uqcrc2	0	0	0	1	0	1
mmu-miR-223-3p	67009	Ttc23	0	0	0	1	0	1
mmu-miR-223-3p	67013	Oma1	0	0	0	1	0	1
mmu-miR-223-3p	67015	Ccdc91	0	0	0	1	0	1
mmu-miR-223-3p	67019	Actr6	1	0	0	0	0	1
mmu-miR-223-3p	67026	Thap4	0	0	0	1	0	1

mmu-miR-223-3p	67027	Mkrn2	0	0	0	1	0	1
mmu-miR-223-3p	67031	Upf3a	0	0	0	1	0	1
mmu-miR-223-3p	67035	Dnajb4	0	0	0	1	0	1
mmu-miR-223-3p	67037	Pmf1	0	0	0	1	0	1
mmu-miR-223-3p	67039	Rbm25	0	0	0	1	0	1
mmu-miR-223-3p	67041	Oxct1	0	1	0	0	0	1
mmu-miR-223-3p	67043	Syap1	0	0	0	1	0	1
mmu-miR-223-3p	67046	Tbc1d7	0	0	0	1	0	1
mmu-miR-223-3p	67049	Pus3	0	1	0	0	0	1
mmu-miR-223-3p	67053	Rpp14	0	0	0	1	0	1
mmu-miR-223-3p	67054	Paics	0	0	0	1	0	1
mmu-miR-223-3p	67057	Yaf2	0	0	0	1	0	1
mmu-miR-223-3p	67063	Tmem246	0	0	0	1	0	1
mmu-miR-223-3p	67068	Dynlrb1	0	0	0	1	0	1
mmu-miR-223-3p	67070	Lsm14a	0	0	0	1	0	1
mmu-miR-223-3p	67073	Pi4k2b	0	0	0	1	0	1
mmu-miR-223-3p	67089	Psmc6	0	0	0	1	0	1
mmu-miR-223-3p	67092	Gatm	0	0	0	1	0	1
mmu-miR-223-3p	67095	Trak1	0	0	0	1	0	1
mmu-miR-223-3p	67096	Mmachc	0	0	0	1	0	1
mmu-miR-223-3p	67099	Mettl21a	0	0	0	1	0	1
mmu-miR-223-3p	67101	2310039H08Rik	1	0	0	0	0	1
mmu-miR-223-3p	67106	Zbtb8os	0	0	0	1	0	1
mmu-miR-223-3p	67109	Zfp787	0	0	0	1	0	1
mmu-miR-223-3p	67116	Cuedc2	0	0	0	1	0	1
mmu-miR-223-3p	67122	Nrarp	0	0	0	1	0	1
mmu-miR-223-3p	67123	Ubap1	0	0	0	1	0	1
mmu-miR-223-3p	67125	Tspan31	0	0	0	1	0	1
mmu-miR-223-3p	67127	Lce1a1	1	0	0	0	0	1
mmu-miR-223-3p	67133	Gp2	0	0	0	1	0	1
mmu-miR-223-3p	67138	Herc6	0	0	0	1	0	1
mmu-miR-223-3p	67148	Fam103a1	0	0	0	1	0	1
mmu-miR-223-3p	67153	Rnaseh2b	1	0	0	0	0	1
mmu-miR-223-3p	67158	Sft2d3	0	0	0	1	0	1
mmu-miR-223-3p	67180	Yipf5	0	0	0	1	0	1
mmu-miR-223-3p	67199	Pfdn1	0	0	0	1	0	1
mmu-miR-223-3p	67210	Gatad1	0	0	0	1	0	1
mmu-miR-223-3p	67211	Armc10	0	0	0	1	0	1
mmu-miR-223-3p	67216	Mboat2	0	0	0	1	0	1
mmu-miR-223-3p	67219	Med18	0	0	0	1	0	1
mmu-miR-223-3p	67224	Med29	0	0	0	1	0	1
mmu-miR-223-3p	67231	Tbc1d20	0	1	0	0	0	1
mmu-miR-223-3p	67236	Cinp	0	0	0	1	0	1
mmu-miR-223-3p	67238	Fam220a	0	0	0	1	0	1
mmu-miR-223-3p	67239	Rpf2	0	0	0	1	0	1
mmu-miR-223-3p	67242	Gemin6	0	0	0	1	0	1
mmu-miR-223-3p	67246	2810474O19Rik	0	0	0	1	0	1
mmu-miR-223-3p	67247	Marc2	0	0	0	1	0	1
mmu-miR-223-3p	67249	Tbc1d19	0	0	0	1	0	1
mmu-miR-223-3p	67255	Zfp422	0	0	0	1	0	1
mmu-miR-223-3p	67263	Zswim6	1	0	0	0	0	1
mmu-miR-223-3p	67266	Fam69a	0	0	0	1	0	1
mmu-miR-223-3p	67272	Cmtm5	0	0	0	1	0	1
mmu-miR-223-3p	67279	Med31	0	0	0	1	0	1
mmu-miR-223-3p	67281	Rpl37	0	0	0	1	0	1

mmu-miR-223-3p	67282	Ccdc53	1	0	0	0	0	1
mmu-miR-223-3p	67286	Rabl5	0	0	0	1	0	1
mmu-miR-223-3p	67292	Pigc	0	0	0	1	0	1
mmu-miR-223-3p	67298	Gprasp1	0	0	0	1	0	1
mmu-miR-223-3p	67300	Cltc	0	0	0	1	0	1
mmu-miR-223-3p	67305	Gpx7	0	0	0	1	0	1
mmu-miR-223-3p	67307	Pbld2	1	0	0	0	0	1
mmu-miR-223-3p	67308	Mrpl46	0	0	0	1	0	1
mmu-miR-223-3p	67311	Nanp	0	0	0	1	0	1
mmu-miR-223-3p	67313	5730559C18Rik	0	0	0	1	0	1
mmu-miR-223-3p	67315	Ceacam12	0	0	0	1	0	1
mmu-miR-223-3p	67317	1700022I11Rik	0	0	0	1	0	1
mmu-miR-223-3p	67323	1700042G07Rik	0	0	0	1	0	1
mmu-miR-223-3p	67326	1700037H04Rik	0	0	0	1	0	1
mmu-miR-223-3p	67337	Cstf1	0	0	0	1	0	1
mmu-miR-223-3p	67343	1700065I17Rik	0	1	0	0	0	1
mmu-miR-223-3p	67345	Herc4	0	0	0	1	0	1
mmu-miR-223-3p	67356	Tmco5	0	0	0	1	0	1
mmu-miR-223-3p	67358	1700093K21Rik	0	0	0	1	0	1
mmu-miR-223-3p	67369	Qpctl	0	0	0	1	0	1
mmu-miR-223-3p	67373	2210010C04Rik	0	0	0	1	0	1
mmu-miR-223-3p	67383	2410127L17Rik	1	0	0	0	0	1
mmu-miR-223-3p	67390	Rnmtl1	0	0	0	1	0	1
mmu-miR-223-3p	67391	Fundc2	0	0	0	1	0	1
mmu-miR-223-3p	67397	Erp29	0	0	0	1	0	1
mmu-miR-223-3p	67402	Txndc8	0	0	0	1	0	1
mmu-miR-223-3p	67405	Nts	0	0	0	1	0	1
mmu-miR-223-3p	67412	Soga3	0	0	0	1	0	1
mmu-miR-223-3p	67414	Mfn1	0	0	0	1	0	1
mmu-miR-223-3p	67426	Adck3	0	0	0	1	0	1
mmu-miR-223-3p	67429	Nudcd1	0	0	0	1	0	1
mmu-miR-223-3p	67430	4921536K21Rik	0	0	0	1	0	1
mmu-miR-223-3p	67437	Ssr3	0	0	0	1	0	1
mmu-miR-223-3p	67439	Xab2	0	1	0	0	0	1
mmu-miR-223-3p	67441	Isoc2b	0	0	0	1	0	1
mmu-miR-223-3p	67444	Ilkap	0	0	0	1	0	1
mmu-miR-223-3p	67446	Dusp28	0	0	0	1	0	1
mmu-miR-223-3p	67458	Ergic1	0	0	0	1	0	1
mmu-miR-223-3p	67464	Entpd4	0	0	0	1	0	1
mmu-miR-223-3p	67473	Slc47a1	0	0	0	1	0	1
mmu-miR-223-3p	67475	Ero1lb	0	0	0	1	0	1
mmu-miR-223-3p	67484	Eepd1	0	0	0	1	0	1
mmu-miR-223-3p	67489	Ap4b1	0	0	0	1	0	1
mmu-miR-223-3p	67509	Saysd1	0	0	0	1	0	1
mmu-miR-223-3p	67511	Tmed9	0	0	0	1	0	1
mmu-miR-223-3p	67512	Agpat2	0	0	0	1	0	1
mmu-miR-223-3p	67515	Ttc33	0	0	0	1	0	1
mmu-miR-223-3p	67526	Atg12	0	0	0	1	0	1
mmu-miR-223-3p	67528	Nudt7	0	0	0	1	0	1
mmu-miR-223-3p	67534	Ttll4	0	0	0	1	0	1
mmu-miR-223-3p	67537	Glipr1l2	0	0	0	1	0	1
mmu-miR-223-3p	67542	Cog6	0	0	0	1	0	1
mmu-miR-223-3p	67549	Gpr89	0	1	0	0	0	1
mmu-miR-223-3p	67553	Gstcd	0	0	0	1	0	1
mmu-miR-223-3p	67555	4933434I20Rik	0	0	0	1	0	1

mmu-miR-223-3p	67557	Larp6	1	0	0	0	0	1
mmu-miR-223-3p	67563	Narfl	0	0	0	1	0	1
mmu-miR-223-3p	67564	Tmem35	0	0	0	1	0	1
mmu-miR-223-3p	67575	4930430A15Rik	0	0	0	1	0	1
mmu-miR-223-3p	67578	Patl2	0	0	0	1	0	1
mmu-miR-223-3p	67579	Cpeb4	0	1	0	0	0	1
mmu-miR-223-3p	67580	Lrrc18	0	0	0	1	0	1
mmu-miR-223-3p	67596	Tespa1	0	0	0	1	0	1
mmu-miR-223-3p	67603	Dusp6	0	0	0	1	0	1
mmu-miR-223-3p	67604	Get4	0	0	0	1	0	1
mmu-miR-223-3p	67605	Akt1s1	0	0	0	1	0	1
mmu-miR-223-3p	67606	Fibin	0	0	0	1	0	1
mmu-miR-223-3p	67609	4930453N24Rik	0	0	1	0	0	1
mmu-miR-223-3p	67618	Aasdhppt	1	0	0	0	0	1
mmu-miR-223-3p	67619	Nob1	0	0	0	1	0	1
mmu-miR-223-3p	67622	Mxra7	0	0	0	1	0	1
mmu-miR-223-3p	67628	Anp32b	0	0	0	1	0	1
mmu-miR-223-3p	67629	Spc24	0	0	0	1	0	1
mmu-miR-223-3p	67646	4930522H14Rik	1	0	0	0	0	1
mmu-miR-223-3p	67647	4930523C07Rik	0	0	0	1	0	1
mmu-miR-223-3p	67653	4930544G11Rik	0	0	0	1	0	1
mmu-miR-223-3p	67655	Ctdp1	0	0	0	1	0	1
mmu-miR-223-3p	67657	Rabl3	0	0	0	1	0	1
mmu-miR-223-3p	67663	4930549C01Rik	0	0	0	1	0	1
mmu-miR-223-3p	67664	Rnf125	0	0	0	1	0	1
mmu-miR-223-3p	67673	Tceb2	0	0	0	1	0	1
mmu-miR-223-3p	67674	Trmt112	0	0	0	1	0	1
mmu-miR-223-3p	67678	Lsm3	0	0	0	1	0	1
mmu-miR-223-3p	67681	Mrpl18	0	0	0	1	0	1
mmu-miR-223-3p	67683	Pbdc1	0	0	0	1	0	1
mmu-miR-223-3p	67685	Dyx1c1	0	0	0	1	0	1
mmu-miR-223-3p	67687	1700011L22Rik	0	0	0	1	0	1
mmu-miR-223-3p	67689	Aldh3b1	0	0	0	1	0	1
mmu-miR-223-3p	67690	Prss37	0	0	0	1	0	1
mmu-miR-223-3p	67694	Ift74	1	0	0	0	0	1
mmu-miR-223-3p	67708	Pcnxl4	0	0	0	1	0	1
mmu-miR-223-3p	67711	Nsmce1	0	0	0	1	0	1
mmu-miR-223-3p	67717	Lipf	0	0	0	1	0	1
mmu-miR-223-3p	67722	Actl11	0	0	0	1	0	1
mmu-miR-223-3p	67726	Fam114a2	0	0	0	1	0	1
mmu-miR-223-3p	67728	Dph2	0	0	1	0	0	1
mmu-miR-223-3p	67729	Mansc1	0	0	0	1	0	1
mmu-miR-223-3p	67733	Itgb3bp	0	0	0	1	0	1
mmu-miR-223-3p	67736	Ccdc130	0	0	1	0	0	1
mmu-miR-223-3p	67738	Ppid	0	0	0	1	0	1
mmu-miR-223-3p	67750	4930578I06Rik	0	0	0	1	0	1
mmu-miR-223-3p	67755	Ddx47	0	0	0	1	0	1
mmu-miR-223-3p	67759	Plgrkt	0	0	0	1	0	1
mmu-miR-223-3p	67767	Jagn1	0	0	0	1	0	1
mmu-miR-223-3p	67773	Kat8	0	0	0	1	0	1
mmu-miR-223-3p	67775	Rtp4	0	0	0	1	0	1
mmu-miR-223-3p	67797	Snrnp48	0	0	0	1	0	1
mmu-miR-223-3p	67808	Tprgl	0	0	0	1	0	1
mmu-miR-223-3p	67809	Rmdn3	0	0	0	1	0	1
mmu-miR-223-3p	67826	Snap47	0	0	0	1	0	1

mmu-miR-223-3p	67838	Dnajb11	0	0	0	1	0	1
mmu-miR-223-3p	67841	Atg3	0	0	0	1	0	1
mmu-miR-223-3p	67842	Nop9	0	0	0	1	0	1
mmu-miR-223-3p	67843	Slc35a4	0	0	0	1	0	1
mmu-miR-223-3p	67844	Rab32	0	0	0	1	0	1
mmu-miR-223-3p	67845	Rnf115	0	0	0	1	0	1
mmu-miR-223-3p	67846	Tmem39a	0	0	0	1	0	1
mmu-miR-223-3p	67847	Sncaip	0	0	0	1	0	1
mmu-miR-223-3p	67848	Ddx55	0	0	0	1	0	1
mmu-miR-223-3p	67849	Cdca5	0	0	0	1	0	1
mmu-miR-223-3p	67855	Asprv1	0	0	0	1	0	1
mmu-miR-223-3p	67859	2310002J15Rik	0	0	0	1	0	1
mmu-miR-223-3p	67860	S100a16	0	0	0	1	0	1
mmu-miR-223-3p	67862	2310033P09Rik	0	0	0	1	0	1
mmu-miR-223-3p	67866	Wfdc1	0	0	0	1	0	1
mmu-miR-223-3p	67868	Cela3b	0	0	0	1	0	1
mmu-miR-223-3p	67872	Nsmce4a	0	1	0	0	0	1
mmu-miR-223-3p	67874	Rprm	0	0	0	1	0	1
mmu-miR-223-3p	67876	Coq10b	0	0	0	1	0	1
mmu-miR-223-3p	67877	Naa20	0	0	0	1	0	1
mmu-miR-223-3p	67884	1810043G02Rik	0	0	0	1	0	1
mmu-miR-223-3p	67891	Rpl4	1	0	0	0	0	1
mmu-miR-223-3p	67893	Tmem86a	0	0	0	1	0	1
mmu-miR-223-3p	67894	Fam45a	0	0	0	1	0	1
mmu-miR-223-3p	67895	Ppa1	0	0	0	1	0	1
mmu-miR-223-3p	67896	Ccdc80	0	0	0	1	0	1
mmu-miR-223-3p	67899	Cmc1	0	0	0	1	0	1
mmu-miR-223-3p	67900	Mtfp1	0	0	0	1	0	1
mmu-miR-223-3p	67902	Sumf2	0	0	0	1	0	1
mmu-miR-223-3p	67903	Gipc1	0	0	0	1	0	1
mmu-miR-223-3p	67916	Ppap2b	0	0	0	1	0	1
mmu-miR-223-3p	67917	Zcchc3	0	0	0	1	0	1
mmu-miR-223-3p	67921	Ube2f	0	0	0	1	0	1
mmu-miR-223-3p	67929	Ccdc70	0	0	0	1	0	1
mmu-miR-223-3p	67931	Serpini2	0	0	0	1	0	1
mmu-miR-223-3p	67932	1700129C05Rik	0	0	0	1	0	1
mmu-miR-223-3p	67935	Ces5a	0	0	0	1	0	1
mmu-miR-223-3p	67937	Tmem59l	0	0	0	1	0	1
mmu-miR-223-3p	67938	Myl12b	0	0	0	1	0	1
mmu-miR-223-3p	67945	Rpl41	1	0	0	0	0	1
mmu-miR-223-3p	67951	Tubb6	0	0	0	1	0	1
mmu-miR-223-3p	67955	Sugt1	1	0	0	0	0	1
mmu-miR-223-3p	67956	Setd8	0	0	0	1	0	1
mmu-miR-223-3p	67959	Puf60	0	0	0	1	0	1
mmu-miR-223-3p	67967	Pold3	0	0	0	1	0	1
mmu-miR-223-3p	67968	Ooep	0	0	0	1	0	1
mmu-miR-223-3p	67971	Tppp3	0	0	0	1	0	1
mmu-miR-223-3p	67976	Trabd	1	0	0	0	0	1
mmu-miR-223-3p	67978	Tctn2	0	0	0	1	0	1
mmu-miR-223-3p	67980	Gnpda2	0	0	0	1	0	1
mmu-miR-223-3p	67983	Pdzd9	0	0	0	1	0	1
mmu-miR-223-3p	67994	Mrps11	0	0	0	1	0	1
mmu-miR-223-3p	67998	Fam134c	0	0	0	1	0	1
mmu-miR-223-3p	68009	Defa20	0	0	0	1	0	1
mmu-miR-223-3p	68014	Zwilch	0	0	0	1	0	1

mmu-miR-223-3p	68015	Trap1	1	0	0	0	0	1
mmu-miR-223-3p	68016	Murc	0	0	0	1	0	1
mmu-miR-223-3p	68021	Bphl	0	0	0	1	0	1
mmu-miR-223-3p	68023	Pdf	0	0	0	1	0	1
mmu-miR-223-3p	68033	Cox19	0	0	0	1	0	1
mmu-miR-223-3p	68040	Zfp593	0	0	0	1	0	1
mmu-miR-223-3p	68045	2700060E02Rik	0	0	0	1	0	1
mmu-miR-223-3p	68055	Atp5s	0	0	1	0	0	1
mmu-miR-223-3p	68058	Chd1l	0	0	0	1	0	1
mmu-miR-223-3p	68059	Tm9sf2	0	0	0	1	0	1
mmu-miR-223-3p	68080	Gpn3	0	0	0	1	0	1
mmu-miR-223-3p	68087	Dcakd	0	0	0	1	0	1
mmu-miR-223-3p	68094	Smarcc2	0	0	0	1	0	1
mmu-miR-223-3p	68097	Dynll2	0	0	0	1	0	1
mmu-miR-223-3p	68098	Rchy1	0	0	0	1	0	1
mmu-miR-223-3p	68099	Fam92a	0	0	0	1	0	1
mmu-miR-223-3p	68112	Sdccag3	0	0	0	1	0	1
mmu-miR-223-3p	68115	9430016H08Rik	0	0	0	1	0	1
mmu-miR-223-3p	68126	Fahd2a	0	0	0	1	0	1
mmu-miR-223-3p	68135	Eif3h	0	0	0	1	0	1
mmu-miR-223-3p	68140	Tigd2	0	0	0	1	0	1
mmu-miR-223-3p	68147	Gar1	0	0	0	1	0	1
mmu-miR-223-3p	68153	Gtf2e2	0	0	0	1	0	1
mmu-miR-223-3p	68165	Fdx1l	1	0	0	0	0	1
mmu-miR-223-3p	68170	B230118H07Rik	0	0	1	0	0	1
mmu-miR-223-3p	68171	D730048I06Rik	0	0	0	1	0	1
mmu-miR-223-3p	68183	Bcas2	0	0	0	1	0	1
mmu-miR-223-3p	68187	Fam135a	0	0	0	1	0	1
mmu-miR-223-3p	68192	Leprotl1	0	0	0	1	0	1
mmu-miR-223-3p	68198	Ndufb2	1	0	0	0	0	1
mmu-miR-223-3p	68201	Ccdc34	0	0	0	1	0	1
mmu-miR-223-3p	68205	Urm1	0	0	1	0	0	1
mmu-miR-223-3p	68215	Fam98b	0	0	0	1	0	1
mmu-miR-223-3p	68221	Wfdc15a	0	0	0	1	0	1
mmu-miR-223-3p	68235	2410066E13Rik	0	0	0	1	0	1
mmu-miR-223-3p	68239	Krt42	0	0	0	1	0	1
mmu-miR-223-3p	68241	Fam195a	0	0	0	1	0	1
mmu-miR-223-3p	68250	Fam96a	0	0	0	1	0	1
mmu-miR-223-3p	68251	Babam1	0	0	0	1	0	1
mmu-miR-223-3p	68262	Agpat4	1	0	0	0	0	1
mmu-miR-223-3p	68265	Iqcf3	0	0	0	1	0	1
mmu-miR-223-3p	68267	Slc25a22	0	0	0	1	0	1
mmu-miR-223-3p	68268	Zdhhc21	0	0	0	1	0	1
mmu-miR-223-3p	68272	Rbm28	0	0	0	1	0	1
mmu-miR-223-3p	68275	Rpa1	0	0	0	1	0	1
mmu-miR-223-3p	68279	Mcoln2	0	0	0	1	0	1
mmu-miR-223-3p	68299	Vps53	0	1	0	0	0	1
mmu-miR-223-3p	68312	Gstm7	0	0	0	1	0	1
mmu-miR-223-3p	68327	Tsr3	0	0	0	1	0	1
mmu-miR-223-3p	68328	Rab13	0	0	0	1	0	1
mmu-miR-223-3p	68332	Sdhaf1	0	0	0	1	0	1
mmu-miR-223-3p	68337	Crip2	0	0	0	1	0	1
mmu-miR-223-3p	68339	Ccdc88c	0	0	0	1	0	1
mmu-miR-223-3p	68344	Tmem174	0	0	0	1	0	1
mmu-miR-223-3p	68371	Pbld1	0	0	0	1	0	1

mmu-miR-223-3p	68375	Ndufa8	0	0	0	1	0	1
mmu-miR-223-3p	68379	Ciz1	0	0	0	1	0	1
mmu-miR-223-3p	68385	Tlcd1	0	0	0	1	0	1
mmu-miR-223-3p	68404	Nrn1	0	0	0	1	0	1
mmu-miR-223-3p	68449	Tbc1d10b	0	0	0	1	0	1
mmu-miR-223-3p	68463	Mrpl14	0	0	0	1	0	1
mmu-miR-223-3p	68472	Tmem126b	0	0	0	1	0	1
mmu-miR-223-3p	68479	Phf5a	1	0	0	0	0	1
mmu-miR-223-3p	68490	Zfp579	0	0	0	1	0	1
mmu-miR-223-3p	68498	Tspan11	0	0	0	1	0	1
mmu-miR-223-3p	68501	Nsmce2	0	0	0	1	0	1
mmu-miR-223-3p	68507	Ppfia4	0	0	0	1	0	1
mmu-miR-223-3p	68510	Ints1	0	0	0	1	0	1
mmu-miR-223-3p	68511	Dcdc2c	0	0	0	1	0	1
mmu-miR-223-3p	68515	Myadml2	0	0	0	1	0	1
mmu-miR-223-3p	68520	Zfyve21	0	0	0	1	0	1
mmu-miR-223-3p	68521	Fam189b	0	0	0	1	0	1
mmu-miR-223-3p	68528	Smim6	0	0	0	1	0	1
mmu-miR-223-3p	68533	Mphosph6	0	0	0	1	0	1
mmu-miR-223-3p	68539	Tmem109	0	0	0	1	0	1
mmu-miR-223-3p	68544	2310036O22Rik	0	0	0	1	0	1
mmu-miR-223-3p	68566	Caly	0	0	0	1	0	1
mmu-miR-223-3p	68576	Lamtor5	0	0	0	1	0	1
mmu-miR-223-3p	68592	Syf2	0	0	0	1	0	1
mmu-miR-223-3p	68603	Pmvk	0	0	0	1	0	1
mmu-miR-223-3p	68611	Mrpl28	0	0	0	1	0	1
mmu-miR-223-3p	68612	Ube2c	0	0	0	1	0	1
mmu-miR-223-3p	68617	Soga2	0	0	0	1	0	1
mmu-miR-223-3p	68628	Fbxw9	0	0	0	1	0	1
mmu-miR-223-3p	68631	Cryl1	0	0	0	1	0	1
mmu-miR-223-3p	68642	Tmem216	0	0	0	1	0	1
mmu-miR-223-3p	68646	Nadk2	0	0	0	1	0	1
mmu-miR-223-3p	68655	Fndc1	0	0	0	1	0	1
mmu-miR-223-3p	68667	Trpm4	0	0	0	1	0	1
mmu-miR-223-3p	68682	Slc44a2	0	0	0	1	0	1
mmu-miR-223-3p	68693	Hnrnpul2	0	0	0	1	0	1
mmu-miR-223-3p	68701	Ppp1r27	0	0	0	1	0	1
mmu-miR-223-3p	68718	Rnf166	0	0	0	1	0	1
mmu-miR-223-3p	68721	1110032A03Rik	0	0	0	1	0	1
mmu-miR-223-3p	68723	Hrnr	0	0	0	1	0	1
mmu-miR-223-3p	68725	1110032F04Rik	0	0	0	1	0	1
mmu-miR-223-3p	68731	Rbfa	0	0	0	1	0	1
mmu-miR-223-3p	68732	Lrrc16a	0	0	0	1	0	1
mmu-miR-223-3p	68736	Tyw5	0	0	0	1	0	1
mmu-miR-223-3p	68737	Angel1	0	0	0	1	0	1
mmu-miR-223-3p	68740	Krtap22-2	0	0	0	1	0	1
mmu-miR-223-3p	68760	Synpo2l	0	0	0	1	0	1
mmu-miR-223-3p	68768	Krtap4-6	0	0	0	1	0	1
mmu-miR-223-3p	68774	Ms4a6d	0	0	1	0	0	1
mmu-miR-223-3p	68795	Ubr3	0	0	0	1	0	1
mmu-miR-223-3p	68799	Rgmb	0	0	0	1	0	1
mmu-miR-223-3p	68801	Elovl5	0	0	0	1	0	1
mmu-miR-223-3p	68802	Mypn	0	0	0	1	0	1
mmu-miR-223-3p	68815	Btbd10	1	0	0	0	0	1
mmu-miR-223-3p	68816	Ppil1	0	0	0	1	0	1

mmu-miR-223-3p	68818	Zfand2b	0	0	0	1	0	1
mmu-miR-223-3p	68828	Sync	0	0	0	1	0	1
mmu-miR-223-3p	68837	Foxk2	0	0	0	1	0	1
mmu-miR-223-3p	68842	Tulp4	0	0	0	1	0	1
mmu-miR-223-3p	68852	Lrrn4cl	0	0	0	1	0	1
mmu-miR-223-3p	68857	Dtwd2	0	0	0	1	0	1
mmu-miR-223-3p	68876	Xrcc6bp1	0	0	0	1	0	1
mmu-miR-223-3p	68880	1190003K10Rik	0	0	0	1	0	1
mmu-miR-223-3p	68888	Gkn3	0	0	0	1	0	1
mmu-miR-223-3p	68891	Cd177	0	0	0	1	0	1
mmu-miR-223-3p	68895	Rasl11a	0	0	0	1	0	1
mmu-miR-223-3p	68911	Pygo2	0	0	0	1	0	1
mmu-miR-223-3p	68917	Hint2	0	0	0	1	0	1
mmu-miR-223-3p	68918	1190005I06Rik	0	0	0	1	0	1
mmu-miR-223-3p	68920	1110065P20Rik	0	0	0	1	0	1
mmu-miR-223-3p	68929	Mospd3	0	0	0	1	0	1
mmu-miR-223-3p	68936	Smim11	0	0	0	1	0	1
mmu-miR-223-3p	68943	Pink1	0	0	0	1	0	1
mmu-miR-223-3p	68952	Fam57b	0	0	0	1	0	1
mmu-miR-223-3p	68961	Phkg2	0	1	0	0	0	1
mmu-miR-223-3p	68971	Tamm41	0	0	0	1	0	1
mmu-miR-223-3p	68972	Tatdn3	0	0	0	1	0	1
mmu-miR-223-3p	68975	Med27	0	0	0	1	0	1
mmu-miR-223-3p	68981	Snrpa1	0	0	0	1	0	1
mmu-miR-223-3p	68988	Prpf31	0	0	0	1	0	1
mmu-miR-223-3p	68991	Ssu72	0	0	0	1	0	1
mmu-miR-223-3p	69009	Thap7	0	0	0	1	0	1
mmu-miR-223-3p	69019	Spcs1	0	1	0	0	0	1
mmu-miR-223-3p	69029	Smdt1	0	0	0	1	0	1
mmu-miR-223-3p	69032	Lyzl4	0	0	0	1	0	1
mmu-miR-223-3p	69047	Atp2c2	0	0	0	1	0	1
mmu-miR-223-3p	69048	Slc30a5	0	0	0	1	0	1
mmu-miR-223-3p	69051	Pycr2	0	0	0	1	0	1
mmu-miR-223-3p	69053	1810013L24Rik	0	0	0	1	0	1
mmu-miR-223-3p	69064	Fuom	0	0	0	1	0	1
mmu-miR-223-3p	69065	Chac1	0	0	0	1	0	1
mmu-miR-223-3p	69073	1810019J16Rik	0	0	0	1	0	1
mmu-miR-223-3p	69076	Triap1	0	0	0	1	0	1
mmu-miR-223-3p	69077	Psmd11	0	0	0	1	0	1
mmu-miR-223-3p	69082	Zc3h15	0	0	0	1	0	1
mmu-miR-223-3p	69089	Oxa1l	0	0	0	1	0	1
mmu-miR-223-3p	69106	Stoml1	1	0	0	0	0	1
mmu-miR-223-3p	69109	Fam58b	0	0	0	1	0	1
mmu-miR-223-3p	69121	Chrdl2	0	0	0	1	0	1
mmu-miR-223-3p	69129	Pex11g	0	0	0	1	0	1
mmu-miR-223-3p	69136	Tusc1	0	0	0	1	0	1
mmu-miR-223-3p	69149	Kbtbd3	0	0	0	1	0	1
mmu-miR-223-3p	69150	Snx4	0	0	0	1	0	1
mmu-miR-223-3p	69151	Lzic	0	0	0	1	0	1
mmu-miR-223-3p	69162	Sec31a	0	0	0	1	0	1
mmu-miR-223-3p	69163	Mrpl44	0	0	0	1	0	1
mmu-miR-223-3p	69165	Cd209b	0	0	0	1	0	1
mmu-miR-223-3p	69168	Bola1	0	0	0	1	0	1
mmu-miR-223-3p	69169	Faim3	0	0	0	1	0	1
mmu-miR-223-3p	69189	1810033B17Rik	0	0	0	1	0	1

mmu-miR-223-3p	69195	Tmem121	0	0	0	1	0	1
mmu-miR-223-3p	69207	Srsf11	0	0	0	1	0	1
mmu-miR-223-3p	69216	Ccdc23	0	0	0	1	0	1
mmu-miR-223-3p	69225	Carkd	0	0	0	1	0	1
mmu-miR-223-3p	69232	Qrich1	0	0	0	1	0	1
mmu-miR-223-3p	69241	Polr2d	0	0	0	1	0	1
mmu-miR-223-3p	69257	Elf2	0	1	0	0	0	1
mmu-miR-223-3p	69260	Ing2	0	0	0	1	0	1
mmu-miR-223-3p	69270	Gins1	0	0	0	1	0	1
mmu-miR-223-3p	69277	3300002I08Rik	1	0	0	0	0	1
mmu-miR-223-3p	69281	Spata4	0	0	0	1	0	1
mmu-miR-223-3p	69282	1700001J03Rik	0	0	0	1	0	1
mmu-miR-223-3p	69287	Odf3	0	0	0	1	0	1
mmu-miR-223-3p	69305	Dcps	0	0	0	1	0	1
mmu-miR-223-3p	69310	Pacrg	1	0	0	0	0	1
mmu-miR-223-3p	69324	1700012B07Rik	0	0	0	1	0	1
mmu-miR-223-3p	69327	1700007K13Rik	0	0	0	1	0	1
mmu-miR-223-3p	69347	1700008P02Rik	0	0	0	1	0	1
mmu-miR-223-3p	69354	Slc38a4	0	0	0	1	0	1
mmu-miR-223-3p	69358	Lrrc51	0	0	0	1	0	1
mmu-miR-223-3p	69361	Cypt3	1	0	0	0	0	1
mmu-miR-223-3p	69372	Mocs3	1	0	0	0	0	1
mmu-miR-223-3p	69382	1700024P04Rik	0	0	0	1	0	1
mmu-miR-223-3p	69396	1700018F24Rik	0	0	0	1	0	1
mmu-miR-223-3p	69398	Cdhr4	0	1	0	0	0	1
mmu-miR-223-3p	69416	1700025F22Rik	0	0	0	1	0	1
mmu-miR-223-3p	69440	Dennd6b	0	0	0	1	0	1
mmu-miR-223-3p	69453	Prss56	0	0	0	1	0	1
mmu-miR-223-3p	69462	2300005B03Rik	0	0	0	1	0	1
mmu-miR-223-3p	69464	Krtap4-13	0	0	0	1	0	1
mmu-miR-223-3p	69478	2300009A05Rik	0	0	0	1	0	1
mmu-miR-223-3p	69482	Nup35	0	0	0	1	0	1
mmu-miR-223-3p	69487	Ndufaf5	0	0	0	1	0	1
mmu-miR-223-3p	69496	Dydc1	0	0	0	1	0	1
mmu-miR-223-3p	69514	Lce3e	0	0	0	1	0	1
mmu-miR-223-3p	69519	Rwdd2a	0	0	0	1	0	1
mmu-miR-223-3p	69524	Esam	0	0	0	1	0	1
mmu-miR-223-3p	69533	Krtap26-1	1	0	0	0	0	1
mmu-miR-223-3p	69534	Avpi1	0	0	0	1	0	1
mmu-miR-223-3p	69536	Hemk1	0	0	0	1	0	1
mmu-miR-223-3p	69541	Lyg1	0	0	0	1	0	1
mmu-miR-223-3p	69542	2300002M23Rik	0	0	0	1	0	1
mmu-miR-223-3p	69544	Wdr5b	0	0	0	1	0	1
mmu-miR-223-3p	69550	Bst2	0	0	0	1	0	1
mmu-miR-223-3p	69551	2310022B05Rik	0	0	0	1	0	1
mmu-miR-223-3p	69554	Klhdc2	0	0	0	1	0	1
mmu-miR-223-3p	69556	Bod1	0	0	0	1	0	1
mmu-miR-223-3p	69564	Nmrk2	1	0	0	0	0	1
mmu-miR-223-3p	69573	Hilpda	0	0	0	1	0	1
mmu-miR-223-3p	69574	Cmbl	0	0	0	1	0	1
mmu-miR-223-3p	69576	Smco1	0	0	0	1	0	1
mmu-miR-223-3p	69577	Fastkd3	0	0	0	1	0	1
mmu-miR-223-3p	69585	Hfe2	0	0	0	1	0	1
mmu-miR-223-3p	69587	Pcgf3	0	0	0	1	0	1
mmu-miR-223-3p	69596	Ap5s1	0	0	0	1	0	1

mmu-miR-223-3p	69602	Otop3	1	0	0	0	0	1
mmu-miR-223-3p	69612	Kansl2	1	0	0	0	0	1
mmu-miR-223-3p	69617	Pitrm1	0	0	0	1	0	1
mmu-miR-223-3p	69635	Dapk1	0	0	0	1	0	1
mmu-miR-223-3p	69638	Enho	0	0	0	1	0	1
mmu-miR-223-3p	69654	Dctn2	1	0	0	0	0	1
mmu-miR-223-3p	69656	Pir	0	0	0	1	0	1
mmu-miR-223-3p	69660	Tmbim1	0	0	0	1	0	1
mmu-miR-223-3p	69668	Ccdc115	0	0	0	1	0	1
mmu-miR-223-3p	69671	Tmem52	0	0	0	1	0	1
mmu-miR-223-3p	69675	Pxdn	0	0	0	1	0	1
mmu-miR-223-3p	69677	Il1f8	1	0	0	0	0	1
mmu-miR-223-3p	69683	Emc10	0	0	0	1	0	1
mmu-miR-223-3p	69693	Pof1b	0	0	0	1	0	1
mmu-miR-223-3p	69696	2310057N15Rik	1	0	0	0	0	1
mmu-miR-223-3p	69698	Slc52a3	0	0	0	1	0	1
mmu-miR-223-3p	69699	2310079G19Rik	0	0	0	1	0	1
mmu-miR-223-3p	69700	Col22a1	0	0	0	1	0	1
mmu-miR-223-3p	69710	Arap1	0	0	0	1	0	1
mmu-miR-223-3p	69713	Pin4	0	0	0	1	0	1
mmu-miR-223-3p	69716	Trip13	1	0	0	0	0	1
mmu-miR-223-3p	69724	Rnaseh2a	0	0	0	1	0	1
mmu-miR-223-3p	69731	Gemin7	1	0	0	0	0	1
mmu-miR-223-3p	69737	Ttl	0	0	0	1	0	1
mmu-miR-223-3p	69740	Dph5	0	0	1	0	0	1
mmu-miR-223-3p	69742	Tm2d2	0	0	0	1	0	1
mmu-miR-223-3p	69743	Casz1	0	0	0	1	0	1
mmu-miR-223-3p	69747	Zswim7	0	0	0	1	0	1
mmu-miR-223-3p	69748	Aldh16a1	1	0	0	0	0	1
mmu-miR-223-3p	69752	Zfp511	0	0	0	1	0	1
mmu-miR-223-3p	69754	Fbxo7	0	0	0	1	0	1
mmu-miR-223-3p	69757	Leng1	0	0	0	1	0	1
mmu-miR-223-3p	69770	1600002K03Rik	0	0	0	1	0	1
mmu-miR-223-3p	69772	Bdh2	0	0	0	1	0	1
mmu-miR-223-3p	69774	Ms4a6b	1	0	0	0	0	1
mmu-miR-223-3p	69784	1500009L16Rik	0	0	0	1	0	1
mmu-miR-223-3p	69787	Anxa13	0	0	0	1	0	1
mmu-miR-223-3p	69792	Med6	0	0	0	1	0	1
mmu-miR-223-3p	69804	Tmem147	0	0	0	1	0	1
mmu-miR-223-3p	69809	1810046K07Rik	0	0	0	1	0	1
mmu-miR-223-3p	69816	Mzb1	0	0	0	1	0	1
mmu-miR-223-3p	69823	Fyttd1	0	0	0	1	0	1
mmu-miR-223-3p	69826	Ms4a10	0	0	0	1	0	1
mmu-miR-223-3p	69847	Wnk4	0	0	0	1	0	1
mmu-miR-223-3p	69870	Polr3gl	0	0	0	1	0	1
mmu-miR-223-3p	69875	Ndufa11	0	0	0	1	0	1
mmu-miR-223-3p	69876	Thap3	0	0	0	1	0	1
mmu-miR-223-3p	69878	Snrpf	0	0	0	1	0	1
mmu-miR-223-3p	69890	Zfp219	0	0	0	1	0	1
mmu-miR-223-3p	69893	Selrc1	0	0	0	1	0	1
mmu-miR-223-3p	69894	2010107G23Rik	0	0	1	0	0	1
mmu-miR-223-3p	69903	Rasip1	0	0	0	1	0	1
mmu-miR-223-3p	69917	Nabp2	0	0	0	1	0	1
mmu-miR-223-3p	69922	Vrk2	0	0	0	1	0	1
mmu-miR-223-3p	69926	Dnah17	0	0	0	1	0	1

mmu-miR-223-3p	69928	Apitd1	0	0	0	1	0	1
mmu-miR-223-3p	69930	Zfp715	0	0	0	1	0	1
mmu-miR-223-3p	69934	Trmt10b	0	0	0	1	0	1
mmu-miR-223-3p	69956	Ptcd3	0	0	0	1	0	1
mmu-miR-223-3p	69961	Rpp25l	0	0	0	1	0	1
mmu-miR-223-3p	69983	Sis	0	0	0	1	0	1
mmu-miR-223-3p	69994	Rsc1a1	0	0	0	1	0	1
mmu-miR-223-3p	70021	Nt5dc2	0	0	0	1	0	1
mmu-miR-223-3p	70025	Acot7	0	0	0	1	0	1
mmu-miR-223-3p	70028	Dopey2	0	0	0	1	0	1
mmu-miR-223-3p	70045	2610528A11Rik	0	0	0	1	0	1
mmu-miR-223-3p	70047	Trnt1	0	0	0	1	0	1
mmu-miR-223-3p	70060	Spata3	1	0	0	0	0	1
mmu-miR-223-3p	70073	Zdhhc25	0	0	0	1	0	1
mmu-miR-223-3p	70080	Igsf23	0	0	0	1	0	1
mmu-miR-223-3p	70081	2210404O09Rik	0	0	0	1	0	1
mmu-miR-223-3p	70082	Lysmd2	0	0	0	1	0	1
mmu-miR-223-3p	70083	Metrn	0	0	0	1	0	1
mmu-miR-223-3p	70099	Smc4	0	1	0	0	0	1
mmu-miR-223-3p	70113	Odf3b	0	0	0	1	0	1
mmu-miR-223-3p	70134	2210011C24Rik	0	0	0	1	0	1
mmu-miR-223-3p	70144	Lrch3	0	0	0	1	0	1
mmu-miR-223-3p	70153	2210016F16Rik	0	0	0	1	0	1
mmu-miR-223-3p	70207	Taco1	0	0	0	1	0	1
mmu-miR-223-3p	70208	Med23	0	0	0	1	0	1
mmu-miR-223-3p	70218	Kif18b	0	0	0	1	0	1
mmu-miR-223-3p	70223	Nars	0	0	0	1	0	1
mmu-miR-223-3p	70225	Ppil3	0	0	0	1	0	1
mmu-miR-223-3p	70237	Bhlhb9	0	0	0	1	0	1
mmu-miR-223-3p	70240	Ufsp1	0	0	0	1	0	1
mmu-miR-223-3p	70247	Psmd1	0	0	0	1	0	1
mmu-miR-223-3p	70248	Dazap1	0	0	0	1	0	1
mmu-miR-223-3p	70266	Ccbl1	0	0	0	1	0	1
mmu-miR-223-3p	70274	Ly6g6e	0	0	0	1	0	1
mmu-miR-223-3p	70285	Rpf1	0	0	0	1	0	1
mmu-miR-223-3p	70294	Rnf126	1	0	0	0	0	1
mmu-miR-223-3p	70312	Cactin	0	0	0	1	0	1
mmu-miR-223-3p	70314	Rabep2	0	0	0	1	0	1
mmu-miR-223-3p	70315	Hdac8	0	0	0	1	0	1
mmu-miR-223-3p	70325	Pigw	0	0	0	1	0	1
mmu-miR-223-3p	70335	Reep6	0	0	0	1	0	1
mmu-miR-223-3p	70337	Iyd	0	0	0	1	0	1
mmu-miR-223-3p	70348	Ube2cbp	0	0	0	1	0	1
mmu-miR-223-3p	70349	Copb1	0	0	0	1	0	1
mmu-miR-223-3p	70351	Ppp4r1	0	0	0	1	0	1
mmu-miR-223-3p	70355	Gprc5c	0	0	0	1	0	1
mmu-miR-223-3p	70356	St13	1	0	0	0	0	1
mmu-miR-223-3p	70358	Steap1	0	0	0	1	0	1
mmu-miR-223-3p	70359	Gtpbp3	0	0	0	1	0	1
mmu-miR-223-3p	70369	Bag5	0	0	0	1	0	1
mmu-miR-223-3p	70370	Fbln7	0	0	0	1	0	1
mmu-miR-223-3p	70382	Kctd2	0	0	0	1	0	1
mmu-miR-223-3p	70394	Kptn	0	0	0	1	0	1
mmu-miR-223-3p	70397	Tmem70	0	0	0	1	0	1
mmu-miR-223-3p	70405	Calml3	0	0	0	1	0	1

mmu-miR-223-3p	70419	2810408A11Rik	0	1	0	0	0	1
mmu-miR-223-3p	70425	Csnk1g3	0	0	0	1	0	1
mmu-miR-223-3p	70430	Tbce	0	1	0	0	0	1
mmu-miR-223-3p	70450	Unc13d	0	0	0	1	0	1
mmu-miR-223-3p	70484	Slc35d2	0	0	0	1	0	1
mmu-miR-223-3p	70495	Atp6ap2	0	0	0	1	0	1
mmu-miR-223-3p	70497	Arhgap17	0	0	0	1	0	1
mmu-miR-223-3p	70510	Rnf167	1	0	0	0	0	1
mmu-miR-223-3p	70527	Stambp	0	0	0	1	0	1
mmu-miR-223-3p	70530	Lrfn2	0	0	0	1	0	1
mmu-miR-223-3p	70544	Tmem242	1	0	0	0	0	1
mmu-miR-223-3p	70552	Lrrc56	0	0	0	1	0	1
mmu-miR-223-3p	70564	Fam213a	0	0	0	1	0	1
mmu-miR-223-3p	70575	Gfod2	0	1	0	0	0	1
mmu-miR-223-3p	70584	Pak4	0	0	0	1	0	1
mmu-miR-223-3p	70601	Ecd	0	0	0	1	0	1
mmu-miR-223-3p	70603	Mutyh	0	1	0	0	0	1
mmu-miR-223-3p	70605	Zdhhc24	0	0	0	1	0	1
mmu-miR-223-3p	70612	Tmem230	0	0	0	1	0	1
mmu-miR-223-3p	70675	Vcpip1	0	1	0	0	0	1
mmu-miR-223-3p	70680	3021401N23Rik	0	0	0	1	0	1
mmu-miR-223-3p	70681	Fam175a	0	0	0	1	0	1
mmu-miR-223-3p	70683	Utp20	0	0	0	1	0	1
mmu-miR-223-3p	70686	Dusp16	0	0	0	1	0	1
mmu-miR-223-3p	70693	Gpr125	1	0	0	0	0	1
mmu-miR-223-3p	70696	3830417A13Rik	0	0	0	1	0	1
mmu-miR-223-3p	70713	Gpr137c	1	0	0	0	0	1
mmu-miR-223-3p	70726	Angptl6	0	0	0	1	0	1
mmu-miR-223-3p	70750	Kdsr	0	0	0	1	0	1
mmu-miR-223-3p	70769	Nolc1	0	0	0	1	0	1
mmu-miR-223-3p	70772	Ggnbp1	0	0	0	1	0	1
mmu-miR-223-3p	70779	Prdm5	0	0	0	1	0	1
mmu-miR-223-3p	70784	Rasl12	0	0	0	1	0	1
mmu-miR-223-3p	70790	Ubr5	0	0	0	1	0	1
mmu-miR-223-3p	70791	Hars2	0	0	0	1	0	1
mmu-miR-223-3p	70810	Krt25	0	1	0	0	0	1
mmu-miR-223-3p	70840	Slc22a16	0	0	0	1	0	1
mmu-miR-223-3p	70853	Vwa3b	0	0	0	1	0	1
mmu-miR-223-3p	70862	Spata16	0	0	0	1	0	1
mmu-miR-223-3p	70885	Ints10	0	0	0	1	0	1
mmu-miR-223-3p	70887	Dmrtc1a	0	0	0	1	0	1
mmu-miR-223-3p	70897	Fam71d	0	0	0	1	0	1
mmu-miR-223-3p	70900	4921517D22Rik	0	0	0	1	0	1
mmu-miR-223-3p	70920	4921511H03Rik	0	0	0	1	0	1
mmu-miR-223-3p	70935	Speer4f	0	0	0	1	0	1
mmu-miR-223-3p	70941	4921539E11Rik	0	0	0	1	0	1
mmu-miR-223-3p	70945	Mmrn1	0	0	0	1	0	1
mmu-miR-223-3p	70948	Wdr20b	0	0	0	1	0	1
mmu-miR-223-3p	70950	Ccdc178	0	0	0	1	0	1
mmu-miR-223-3p	70951	Spata1	0	0	0	1	0	1
mmu-miR-223-3p	70956	Tex19.2	0	0	0	1	0	1
mmu-miR-223-3p	70979	Fancd2os	0	0	0	1	0	1
mmu-miR-223-3p	70997	Spef1	0	0	0	1	0	1
mmu-miR-223-3p	70999	Naa40	0	0	0	1	0	1
mmu-miR-223-3p	71020	Spats1	0	0	0	1	0	1

mmu-miR-223-3p	71026	Speer3	0	0	0	1	0	1
mmu-miR-223-3p	71027	Tmem30c	0	0	0	1	0	1
mmu-miR-223-3p	71059	Hexim2	0	0	0	1	0	1
mmu-miR-223-3p	71066	Hsfy2	1	0	0	0	0	1
mmu-miR-223-3p	71083	Dmrtc1c1	0	0	0	1	0	1
mmu-miR-223-3p	71085	Arhgap19	0	0	0	1	0	1
mmu-miR-223-3p	71089	Sept12	0	0	0	1	0	1
mmu-miR-223-3p	71093	Atoh8	0	0	0	1	0	1
mmu-miR-223-3p	71111	Gpr39	0	0	0	1	0	1
mmu-miR-223-3p	71116	Stx18	0	0	0	1	0	1
mmu-miR-223-3p	71137	Rfx4	0	0	0	1	0	1
mmu-miR-223-3p	71145	Scara5	0	0	0	1	0	1
mmu-miR-223-3p	71149	4933413G19Rik	0	0	0	1	0	1
mmu-miR-223-3p	71156	Lrrc72	0	0	0	1	0	1
mmu-miR-223-3p	71162	4933421I07Rik	0	0	0	1	0	1
mmu-miR-223-3p	71177	Asun	0	0	0	1	0	1
mmu-miR-223-3p	71198	Otud1	0	0	0	1	0	1
mmu-miR-223-3p	71200	Dydc2	0	0	0	1	0	1
mmu-miR-223-3p	71223	Gpr15	0	0	0	1	0	1
mmu-miR-223-3p	71236	Rtdr1	0	1	0	0	0	1
mmu-miR-223-3p	71238	Acn9	0	0	0	1	0	1
mmu-miR-223-3p	71240	Osbpl7	0	0	0	1	0	1
mmu-miR-223-3p	71241	Dmrtc2	1	0	0	0	0	1
mmu-miR-223-3p	71242	Spata24	0	0	0	1	0	1
mmu-miR-223-3p	71254	Naif1	0	0	0	1	0	1
mmu-miR-223-3p	71268	Lrrfip2	0	0	0	1	0	1
mmu-miR-223-3p	71300	Rnf148	0	0	0	1	0	1
mmu-miR-223-3p	71310	Tbc1d9	0	0	0	1	0	1
mmu-miR-223-3p	71323	Rassf8	0	0	0	1	0	1
mmu-miR-223-3p	71325	Tchhl1	0	0	0	1	0	1
mmu-miR-223-3p	71345	Ano9	0	0	0	1	0	1
mmu-miR-223-3p	71351	5430402E10Rik	0	0	0	1	0	1
mmu-miR-223-3p	71354	Wdr31	0	0	0	1	0	1
mmu-miR-223-3p	71355	Col24a1	0	0	0	1	0	1
mmu-miR-223-3p	71365	Pdss2	0	0	0	1	0	1
mmu-miR-223-3p	71386	Krtap28-13	0	0	0	1	0	1
mmu-miR-223-3p	71393	Kctd6	0	0	0	1	0	1
mmu-miR-223-3p	71414	5430427G11Rik	0	0	0	1	0	1
mmu-miR-223-3p	71436	Flrt3	0	0	0	1	0	1
mmu-miR-223-3p	71461	Ptk7	0	0	0	1	0	1
mmu-miR-223-3p	71468	Obox1	0	0	0	1	0	1
mmu-miR-223-3p	71472	Usp19	0	0	0	1	0	1
mmu-miR-223-3p	71474	Ppp6r2	0	0	0	1	0	1
mmu-miR-223-3p	71508	Zfp935	0	0	0	1	0	1
mmu-miR-223-3p	71514	Sfpq	0	0	0	1	0	1
mmu-miR-223-3p	71519	Cyp2u1	0	0	0	1	0	1
mmu-miR-223-3p	71522	Ggt6	0	0	0	1	0	1
mmu-miR-223-3p	71538	Fbxo9	0	0	0	1	0	1
mmu-miR-223-3p	71562	Afmid	0	1	0	0	0	1
mmu-miR-223-3p	71564	Izumo4	1	0	0	0	0	1
mmu-miR-223-3p	71601	Ceacam20	1	0	0	0	0	1
mmu-miR-223-3p	71607	Snx20	0	0	0	1	0	1
mmu-miR-223-3p	71609	Tradd	0	0	0	1	0	1
mmu-miR-223-3p	71619	Arl14	0	0	0	1	0	1
mmu-miR-223-3p	71640	Zfp949	0	0	0	1	0	1

mmu-miR-223-3p	71648	Optn	0	0	0	1	0	1
mmu-miR-223-3p	71653	4930506M07Rik	0	0	0	1	0	1
mmu-miR-223-3p	71665	Fuca1	0	0	0	1	0	1
mmu-miR-223-3p	71675	0610010F05Rik	0	0	0	1	0	1
mmu-miR-223-3p	71683	Gypc	0	0	0	1	0	1
mmu-miR-223-3p	71685	Galnt14	0	0	1	0	0	1
mmu-miR-223-3p	71690	Esm1	0	0	0	1	0	1
mmu-miR-223-3p	71699	Slc41a3	0	0	0	1	0	1
mmu-miR-223-3p	71702	Cdc5l	0	0	0	1	0	1
mmu-miR-223-3p	71706	Slc46a3	0	0	0	1	0	1
mmu-miR-223-3p	71710	Lrrcc1	0	0	0	1	0	1
mmu-miR-223-3p	71713	Cdc40	0	0	0	1	0	1
mmu-miR-223-3p	71715	Dhx35	0	0	0	1	0	1
mmu-miR-223-3p	71718	Telo2	0	0	0	1	0	1
mmu-miR-223-3p	71722	Cic	0	0	0	1	0	1
mmu-miR-223-3p	71726	Smug1	0	0	0	1	0	1
mmu-miR-223-3p	71728	Stk11ip	0	0	1	0	0	1
mmu-miR-223-3p	71729	Rgs12	0	0	0	1	0	1
mmu-miR-223-3p	71732	Vps11	0	0	0	1	0	1
mmu-miR-223-3p	71738	Mamdc2	0	0	0	1	0	1
mmu-miR-223-3p	71742	Ulk3	0	0	0	1	0	1
mmu-miR-223-3p	71746	Rgl3	0	0	0	1	0	1
mmu-miR-223-3p	71753	Tmprss6	0	0	0	1	0	1
mmu-miR-223-3p	71754	Cyp2d40	0	0	0	1	0	1
mmu-miR-223-3p	71765	Klhdc3	0	0	0	1	0	1
mmu-miR-223-3p	71767	Tysnd1	0	0	0	1	0	1
mmu-miR-223-3p	71768	Vwce	0	0	0	1	0	1
mmu-miR-223-3p	71781	Slc16a14	0	0	0	1	0	1
mmu-miR-223-3p	71785	Pdgfd	0	1	0	0	0	1
mmu-miR-223-3p	71790	Anxa9	0	0	0	1	0	1
mmu-miR-223-3p	71799	Ptcd1	0	0	0	1	0	1
mmu-miR-223-3p	71803	Slc25a18	0	0	0	1	0	1
mmu-miR-223-3p	71817	Tmem50a	0	0	0	1	0	1
mmu-miR-223-3p	71824	1700006A11Rik	0	0	0	1	0	1
mmu-miR-223-3p	71826	1700001F09Rik	0	0	0	1	0	1
mmu-miR-223-3p	71827	Lrrc34	0	0	0	1	0	1
mmu-miR-223-3p	71828	Gtf2a1l	0	0	0	1	0	1
mmu-miR-223-3p	71829	Ddi1	0	0	0	1	0	1
mmu-miR-223-3p	71831	1700007B14Rik	0	0	0	1	0	1
mmu-miR-223-3p	71834	Zbtb43	0	0	0	1	0	1
mmu-miR-223-3p	71840	Tekt4	0	0	0	1	0	1
mmu-miR-223-3p	71841	1700008I05Rik	0	0	0	1	0	1
mmu-miR-223-3p	71843	R3hcc1	0	0	0	1	0	1
mmu-miR-223-3p	71844	Nupl1	0	0	0	1	0	1
mmu-miR-223-3p	71846	Syce2	0	0	0	1	0	1
mmu-miR-223-3p	71856	Wfdc3	0	0	0	1	0	1
mmu-miR-223-3p	71865	Fbxo30	0	0	0	1	0	1
mmu-miR-223-3p	71870	Ccdc19	1	0	0	0	0	1
mmu-miR-223-3p	71872	Aox4	0	0	0	1	0	1
mmu-miR-223-3p	71874	2310007B03Rik	0	0	0	1	0	1
mmu-miR-223-3p	71877	Efhc1	1	0	0	0	0	1
mmu-miR-223-3p	71881	Apmap	0	0	0	1	0	1
mmu-miR-223-3p	71883	Coq2	0	0	0	1	0	1
mmu-miR-223-3p	71885	2310003H01Rik	0	0	0	1	0	1
mmu-miR-223-3p	71886	2310002L09Rik	0	0	0	1	0	1

mmu-miR-223-3p	71888	Krt33a	0	0	0	1	0	1
mmu-miR-223-3p	71890	Mad2l2	0	0	0	1	0	1
mmu-miR-223-3p	71893	Noxo1	0	0	0	1	0	1
mmu-miR-223-3p	71897	Lypd6b	0	0	0	1	0	1
mmu-miR-223-3p	71901	Fam219a	0	0	0	1	0	1
mmu-miR-223-3p	71902	Cand1	0	0	0	1	0	1
mmu-miR-223-3p	71907	Serpina9	0	0	0	1	0	1
mmu-miR-223-3p	71909	Haus5	0	0	0	1	0	1
mmu-miR-223-3p	71913	Tmem79	0	0	0	1	0	1
mmu-miR-223-3p	71923	2310047M10Rik	0	0	0	1	0	1
mmu-miR-223-3p	71927	Itfg1	0	0	0	1	0	1
mmu-miR-223-3p	71928	2310047K21Rik	0	0	0	1	0	1
mmu-miR-223-3p	71943	Tom1l1	0	0	0	1	0	1
mmu-miR-223-3p	71946	Endod1	0	0	0	1	0	1
mmu-miR-223-3p	71949	Cers5	0	0	0	1	0	1
mmu-miR-223-3p	71952	2410016O06Rik	0	0	0	1	0	1
mmu-miR-223-3p	71954	Suds3	0	0	0	1	0	1
mmu-miR-223-3p	71960	Myh14	0	0	0	1	0	1
mmu-miR-223-3p	71963	Cdca4	0	0	0	1	0	1
mmu-miR-223-3p	71968	Wdr73	0	0	0	1	0	1
mmu-miR-223-3p	71970	2410018M08Rik	0	0	0	1	0	1
mmu-miR-223-3p	71971	Zswim1	0	0	0	1	0	1
mmu-miR-223-3p	71972	Dnmbp	0	0	0	1	0	1
mmu-miR-223-3p	71978	Ppp2r2a	0	0	0	1	0	1
mmu-miR-223-3p	71982	Snx10	0	0	0	1	0	1
mmu-miR-223-3p	71983	Tmco6	0	0	0	1	0	1
mmu-miR-223-3p	71984	Sars2	0	0	0	1	0	1
mmu-miR-223-3p	71986	Ddx28	0	0	0	1	0	1
mmu-miR-223-3p	71990	Ddx54	0	0	0	1	0	1
mmu-miR-223-3p	71998	Slc25a35	0	0	0	1	0	1
mmu-miR-223-3p	72008	Zfyve19	0	0	0	1	0	1
mmu-miR-223-3p	72014	Btbd17	0	0	0	1	0	1
mmu-miR-223-3p	72027	Slc39a4	0	0	0	1	0	1
mmu-miR-223-3p	72039	Mccc1	0	1	0	0	0	1
mmu-miR-223-3p	72041	Alkbh4	0	0	0	1	0	1
mmu-miR-223-3p	72042	Cotl1	0	0	0	1	0	1
mmu-miR-223-3p	72045	2010001E11Rik	0	0	0	1	0	1
mmu-miR-223-3p	72053	Tmub2	0	0	0	1	0	1
mmu-miR-223-3p	72054	Cyp4f18	0	0	0	1	0	1
mmu-miR-223-3p	72057	Phf10	0	1	0	0	0	1
mmu-miR-223-3p	72061	2010111I01Rik	0	0	0	1	0	1
mmu-miR-223-3p	72075	Ogfr	0	0	0	1	0	1
mmu-miR-223-3p	72080	Sapcd2	1	0	0	0	0	1
mmu-miR-223-3p	72088	Ush1c	0	0	0	1	0	1
mmu-miR-223-3p	72094	Ugt2a3	0	0	0	1	0	1
mmu-miR-223-3p	72096	Mettl10	0	0	0	1	0	1
mmu-miR-223-3p	72097	2010300C02Rik	0	0	0	1	0	1
mmu-miR-223-3p	72098	Tmem68	0	0	0	1	0	1
mmu-miR-223-3p	72108	Ddhd2	0	0	0	1	0	1
mmu-miR-223-3p	72125	Amer2	0	0	0	1	0	1
mmu-miR-223-3p	72135	Pygo1	0	1	0	0	0	1
mmu-miR-223-3p	72141	Adpgk	0	0	0	1	0	1
mmu-miR-223-3p	72148	Tdrp	0	0	0	1	0	1
mmu-miR-223-3p	72167	Thumpd2	0	0	0	1	0	1
mmu-miR-223-3p	72170	Chchd4	0	0	0	1	0	1

mmu-miR-223-3p	72179	Fbxl2	0	0	0	1	0	1
mmu-miR-223-3p	72180	Zfp661	0	0	0	1	0	1
mmu-miR-223-3p	72195	Supt7l	0	0	1	0	0	1
mmu-miR-223-3p	72205	Eml2	0	0	0	1	0	1
mmu-miR-223-3p	72221	1700021F07Rik	0	0	0	1	0	1
mmu-miR-223-3p	72230	Zfp558	0	0	0	1	0	1
mmu-miR-223-3p	72238	Tbc1d5	0	0	0	1	0	1
mmu-miR-223-3p	72242	Psg21	0	0	0	1	0	1
mmu-miR-223-3p	72273	2210404O07Rik	0	0	0	1	0	1
mmu-miR-223-3p	72281	Sh2d4a	0	0	0	1	0	1
mmu-miR-223-3p	72282	1810062G17Rik	0	0	0	1	0	1
mmu-miR-223-3p	72284	Oraov1	0	0	0	1	0	1
mmu-miR-223-3p	72296	Rusc1	0	0	0	1	0	1
mmu-miR-223-3p	72303	Cyp2c65	1	0	0	0	0	1
mmu-miR-223-3p	72307	2510002D24Rik	0	0	0	1	0	1
mmu-miR-223-3p	72310	Nkg7	0	0	0	1	0	1
mmu-miR-223-3p	72325	Vps9d1	0	0	0	1	0	1
mmu-miR-223-3p	72330	Klhl40	0	0	0	1	0	1
mmu-miR-223-3p	72338	Wdr89	0	0	0	1	0	1
mmu-miR-223-3p	72341	Elp6	0	0	0	1	0	1
mmu-miR-223-3p	72368	2310045N01Rik	0	0	0	1	0	1
mmu-miR-223-3p	72373	Psca	0	0	0	1	0	1
mmu-miR-223-3p	72401	Slc43a1	0	0	0	1	0	1
mmu-miR-223-3p	72431	Ceacam18	0	0	0	1	0	1
mmu-miR-223-3p	72433	Rab38	0	0	0	1	0	1
mmu-miR-223-3p	72434	Lypd3	0	0	0	1	0	1
mmu-miR-223-3p	72454	Ccdc71	0	0	1	0	0	1
mmu-miR-223-3p	72459	Htatsf1	0	0	0	1	0	1
mmu-miR-223-3p	72465	Zfp131	1	0	0	0	0	1
mmu-miR-223-3p	72469	Plcd3	0	0	0	1	0	1
mmu-miR-223-3p	72475	Ssbp3	0	0	0	1	0	1
mmu-miR-223-3p	72482	Acbd6	0	0	0	1	0	1
mmu-miR-223-3p	72500	Ier5l	0	0	0	1	0	1
mmu-miR-223-3p	72519	Tmem55a	0	0	0	1	0	1
mmu-miR-223-3p	72536	Tagap	0	0	0	1	0	1
mmu-miR-223-3p	72549	Reep4	0	0	0	1	0	1
mmu-miR-223-3p	72556	Zfp566	1	0	0	0	0	1
mmu-miR-223-3p	72562	Pcbd2	0	0	0	1	0	1
mmu-miR-223-3p	72565	Uaca	0	0	0	1	0	1
mmu-miR-223-3p	72567	Bclaf1	0	0	0	1	0	1
mmu-miR-223-3p	72569	Bbs5	0	0	0	1	0	1
mmu-miR-223-3p	72580	Zufsp	0	0	0	1	0	1
mmu-miR-223-3p	72585	Lypd1	0	1	0	0	0	1
mmu-miR-223-3p	72607	Usp13	0	0	0	1	0	1
mmu-miR-223-3p	72611	Zfp655	0	0	0	1	0	1
mmu-miR-223-3p	72614	Pih1d2	0	1	0	0	0	1
mmu-miR-223-3p	72615	Anks3	0	0	0	1	0	1
mmu-miR-223-3p	72634	Tdrkh	0	1	0	0	0	1
mmu-miR-223-3p	72661	Serp2	0	0	0	1	0	1
mmu-miR-223-3p	72662	Dis3	0	0	0	1	0	1
mmu-miR-223-3p	72668	Skida1	0	0	1	0	0	1
mmu-miR-223-3p	72672	Zfp518a	0	0	0	1	0	1
mmu-miR-223-3p	72691	Calhm2	0	0	0	1	0	1
mmu-miR-223-3p	72699	Lime1	0	0	0	1	0	1
mmu-miR-223-3p	72709	C1qtnf6	0	0	0	1	0	1

mmu-miR-223-3p	72726	Tbcc	0	0	0	1	0	1
mmu-miR-223-3p	72736	Tmx1	1	0	0	0	0	1
mmu-miR-223-3p	72754	Arhgef10l	0	1	0	0	0	1
mmu-miR-223-3p	72787	Ndc1	0	0	0	1	0	1
mmu-miR-223-3p	72807	Zfp429	0	0	0	1	0	1
mmu-miR-223-3p	72832	Crtac1	0	0	0	1	0	1
mmu-miR-223-3p	72843	Prdm4	0	0	0	1	0	1
mmu-miR-223-3p	72873	Ccdc176	0	0	0	1	0	1
mmu-miR-223-3p	72886	Ccdc94	0	0	0	1	0	1
mmu-miR-223-3p	72900	Ndufv2	0	1	0	0	0	1
mmu-miR-223-3p	72902	Spock3	0	0	0	1	0	1
mmu-miR-223-3p	72927	Hepacam	1	0	0	0	0	1
mmu-miR-223-3p	72930	Ppp2r2b	0	0	0	1	0	1
mmu-miR-223-3p	72935	Ddx41	0	0	0	1	0	1
mmu-miR-223-3p	72946	Lrrc47	0	0	0	1	0	1
mmu-miR-223-3p	72961	Slc17a7	0	0	0	1	0	1
mmu-miR-223-3p	72962	Tymp	0	0	0	1	0	1
mmu-miR-223-3p	72972	Ccser2	0	0	0	1	0	1
mmu-miR-223-3p	72982	Tmem138	0	0	0	1	0	1
mmu-miR-223-3p	73020	2900073C17Rik	0	0	0	1	0	1
mmu-miR-223-3p	73032	Ttc9b	0	0	0	1	0	1
mmu-miR-223-3p	73047	Camk2n2	0	0	0	1	0	1
mmu-miR-223-3p	73062	Ppp1r16a	0	0	0	1	0	1
mmu-miR-223-3p	73072	BC068157	0	0	0	1	0	1
mmu-miR-223-3p	73090	2900092C05Rik	1	0	0	0	0	1
mmu-miR-223-3p	73102	Slc22a23	0	0	0	1	0	1
mmu-miR-223-3p	73112	Abracl	1	0	0	0	0	1
mmu-miR-223-3p	73121	Fam101a	0	0	0	1	0	1
mmu-miR-223-3p	73149	Clec4a3	0	0	0	1	0	1
mmu-miR-223-3p	73172	Exo5	0	0	0	1	0	1
mmu-miR-223-3p	73174	Tbkbp1	0	0	0	1	0	1
mmu-miR-223-3p	73176	3110040M04Rik	0	0	0	1	0	1
mmu-miR-223-3p	73182	Pear1	0	0	0	1	0	1
mmu-miR-223-3p	73191	Fezf1	0	0	0	1	0	1
mmu-miR-223-3p	73205	3110043O21Rik	0	0	1	0	0	1
mmu-miR-223-3p	73212	3110082I17Rik	0	0	0	1	0	1
mmu-miR-223-3p	73218	Sppl2b	0	0	0	1	0	1
mmu-miR-223-3p	73244	Prl8a1	0	0	0	1	0	1
mmu-miR-223-3p	73247	Mrgbp	0	0	0	1	0	1
mmu-miR-223-3p	73250	Ceacam5	0	0	0	1	0	1
mmu-miR-223-3p	73259	Cib4	0	0	0	1	0	1
mmu-miR-223-3p	73284	Ddit4l	0	0	0	1	0	1
mmu-miR-223-3p	73314	Lrrc69	0	0	0	1	0	1
mmu-miR-223-3p	73316	Calr3	0	0	0	1	0	1
mmu-miR-223-3p	73324	1700034F02Rik	0	0	0	1	0	1
mmu-miR-223-3p	73327	Pradc1	0	0	0	1	0	1
mmu-miR-223-3p	73332	Ccdc30	0	0	0	1	0	1
mmu-miR-223-3p	73333	Slc25a31	0	0	0	1	0	1
mmu-miR-223-3p	73344	1700034J05Rik	0	0	0	1	0	1
mmu-miR-223-3p	73347	1700042B14Rik	0	0	0	1	0	1
mmu-miR-223-3p	73390	Msl3l2	0	0	0	1	0	1
mmu-miR-223-3p	73398	Nipsnap3a	0	0	0	1	0	1
mmu-miR-223-3p	73407	Tepp	0	0	0	1	0	1
mmu-miR-223-3p	73453	1700067K01Rik	0	1	0	0	0	1
mmu-miR-223-3p	73458	1700055N04Rik	0	0	0	1	0	1

mmu-miR-223-3p	73466	Ms4a13	0	0	0	1	0	1
mmu-miR-223-3p	73526	Speer4b	0	0	0	1	0	1
mmu-miR-223-3p	73530	1700074I03Rik	0	0	0	1	0	1
mmu-miR-223-3p	73610	Zfp433	0	0	0	1	0	1
mmu-miR-223-3p	73614	Rhox13	0	0	0	1	0	1
mmu-miR-223-3p	73647	Capn9	0	0	0	1	0	1
mmu-miR-223-3p	73656	Ms4a6c	0	0	0	1	0	1
mmu-miR-223-3p	73667	2410004P03Rik	0	0	0	1	0	1
mmu-miR-223-3p	73677	Psma8	0	0	0	1	0	1
mmu-miR-223-3p	73679	Tex19.1	0	0	0	1	0	1
mmu-miR-223-3p	73681	Trmt11	0	0	0	1	0	1
mmu-miR-223-3p	73689	Bloc1s2	0	0	0	1	0	1
mmu-miR-223-3p	73692	2410089E03Rik	0	0	0	1	0	1
mmu-miR-223-3p	73693	Dppa4	0	0	0	1	0	1
mmu-miR-223-3p	73694	Ndufaf7	0	0	0	1	0	1
mmu-miR-223-3p	73711	Mvb12a	0	0	0	1	0	1
mmu-miR-223-3p	73721	1110017D15Rik	0	0	0	1	0	1
mmu-miR-223-3p	73728	Psd	0	0	0	1	0	1
mmu-miR-223-3p	73729	Zfp383	0	0	0	1	0	1
mmu-miR-223-3p	73737	1110008P14Rik	0	0	0	1	0	1
mmu-miR-223-3p	73739	Cby1	0	0	0	1	0	1
mmu-miR-223-3p	73748	Gadl1	0	0	0	1	0	1
mmu-miR-223-3p	73754	Thap1	0	0	0	1	0	1
mmu-miR-223-3p	73804	Kif2c	0	0	0	1	0	1
mmu-miR-223-3p	73813	Fam83e	0	0	0	1	0	1
mmu-miR-223-3p	73833	Fam98c	0	0	0	1	0	1
mmu-miR-223-3p	73847	Fam110a	0	0	0	1	0	1
mmu-miR-223-3p	73852	D3Ertd751e	0	0	0	1	0	1
mmu-miR-223-3p	73873	Fam161a	0	1	0	0	0	1
mmu-miR-223-3p	73910	Arhgap18	0	0	1	0	0	1
mmu-miR-223-3p	73919	Lyrm1	0	0	0	1	0	1
mmu-miR-223-3p	73942	Fam151b	0	0	0	1	0	1
mmu-miR-223-3p	73991	Atl1	0	0	0	1	0	1
mmu-miR-223-3p	73998	Herc3	0	0	0	1	0	1
mmu-miR-223-3p	74006	Dnm1l	0	0	0	1	0	1
mmu-miR-223-3p	74011	Slc25a27	0	0	0	1	0	1
mmu-miR-223-3p	74012	Rap2b	0	0	0	1	0	1
mmu-miR-223-3p	74015	Fcho1	0	0	0	1	0	1
mmu-miR-223-3p	74018	Als2	0	0	0	1	0	1
mmu-miR-223-3p	74019	Traf3ip1	0	0	0	1	0	1
mmu-miR-223-3p	74022	Glyr1	0	0	0	1	0	1
mmu-miR-223-3p	74026	Msl1	0	0	0	1	0	1
mmu-miR-223-3p	74030	Rin2	1	0	0	0	0	1
mmu-miR-223-3p	74032	Sdr42e1	0	0	0	1	0	1
mmu-miR-223-3p	74035	Nol9	0	0	0	1	0	1
mmu-miR-223-3p	74053	Grip1	0	0	0	1	0	1
mmu-miR-223-3p	74054	4931406B18Rik	0	0	0	1	0	1
mmu-miR-223-3p	74069	Serpina3a	0	0	0	1	0	1
mmu-miR-223-3p	74071	Ifltd1	0	1	0	0	0	1
mmu-miR-223-3p	74087	Slc7a13	1	0	0	0	0	1
mmu-miR-223-3p	74088	0610012H03Rik	0	0	0	1	0	1
mmu-miR-223-3p	74094	Tjap1	0	0	0	1	0	1
mmu-miR-223-3p	74097	Pop7	0	0	0	1	0	1
mmu-miR-223-3p	74098	0610037L13Rik	0	0	0	1	0	1
mmu-miR-223-3p	74114	Crot	0	0	0	1	0	1

mmu-miR-223-3p	74116	Pi16	0	0	0	1	0	1
mmu-miR-223-3p	74120	Zfp263	0	0	0	1	0	1
mmu-miR-223-3p	74132	Rnf6	0	1	0	0	0	1
mmu-miR-223-3p	74133	Smg8	0	0	0	1	0	1
mmu-miR-223-3p	74137	Nuak2	0	0	0	1	0	1
mmu-miR-223-3p	74140	Tm9sf1	0	0	0	1	0	1
mmu-miR-223-3p	74143	Opa1	0	0	0	1	0	1
mmu-miR-223-3p	74145	F13a1	0	0	0	1	0	1
mmu-miR-223-3p	74147	Ehhadh	0	0	0	1	0	1
mmu-miR-223-3p	74148	Cluh	0	0	0	1	0	1
mmu-miR-223-3p	74157	Cmtr1	0	0	0	1	0	1
mmu-miR-223-3p	74159	Acbd5	0	0	0	1	0	1
mmu-miR-223-3p	74175	Crct1	0	0	0	1	0	1
mmu-miR-223-3p	74176	Tgm5	0	0	0	1	0	1
mmu-miR-223-3p	74182	Gpcpd1	0	0	0	1	0	1
mmu-miR-223-3p	74185	Gbe1	0	0	0	1	0	1
mmu-miR-223-3p	74190	Exoc3l4	0	0	1	0	0	1
mmu-miR-223-3p	74194	Rnd3	0	0	0	1	0	1
mmu-miR-223-3p	74195	Elp3	0	0	0	1	0	1
mmu-miR-223-3p	74196	Ttc27	0	0	0	1	0	1
mmu-miR-223-3p	74199	Vit	0	0	0	1	0	1
mmu-miR-223-3p	74203	Eif4enif1	0	0	0	1	0	1
mmu-miR-223-3p	74204	Xpo6	0	0	0	1	0	1
mmu-miR-223-3p	74213	Rbm26	0	0	0	1	0	1
mmu-miR-223-3p	74221	Tex37	0	0	0	1	0	1
mmu-miR-223-3p	74238	Mterfd3	0	0	0	1	0	1
mmu-miR-223-3p	74239	Iqce	0	0	0	1	0	1
mmu-miR-223-3p	74241	Chpf	0	0	0	1	0	1
mmu-miR-223-3p	74253	Klrg2	0	0	0	1	0	1
mmu-miR-223-3p	74256	Cyld	0	0	0	1	0	1
mmu-miR-223-3p	74268	Aven	0	0	0	1	0	1
mmu-miR-223-3p	74270	Usp20	0	0	0	1	0	1
mmu-miR-223-3p	74277	Chic2	0	0	0	1	0	1
mmu-miR-223-3p	74279	1700080O16Rik	0	0	0	1	0	1
mmu-miR-223-3p	74281	Spatc1	0	0	0	1	0	1
mmu-miR-223-3p	74309	Osbp2	0	0	0	1	0	1
mmu-miR-223-3p	74318	Hopx	0	0	0	1	0	1
mmu-miR-223-3p	74325	Cltb	0	0	0	1	0	1
mmu-miR-223-3p	74335	Xrcc3	0	0	1	0	0	1
mmu-miR-223-3p	74337	Palm3	0	0	0	1	0	1
mmu-miR-223-3p	74343	Crtc2	1	0	0	0	0	1
mmu-miR-223-3p	74355	Smchd1	0	0	0	1	0	1
mmu-miR-223-3p	74356	4931428F04Rik	0	0	0	1	0	1
mmu-miR-223-3p	74359	4931414P19Rik	0	0	0	1	0	1
mmu-miR-223-3p	74362	Spag17	0	0	0	1	0	1
mmu-miR-223-3p	74377	Hsf2bp	0	0	0	1	0	1
mmu-miR-223-3p	74383	Ubap2l	0	0	0	1	0	1
mmu-miR-223-3p	74387	4932438H23Rik	0	0	0	1	0	1
mmu-miR-223-3p	74392	Specc1l	0	0	0	1	0	1
mmu-miR-223-3p	74401	4933406J08Rik	0	0	0	1	0	1
mmu-miR-223-3p	74405	Efhc2	0	0	0	1	0	1
mmu-miR-223-3p	74407	Ttc25	0	0	0	1	0	1
mmu-miR-223-3p	74410	Ttll11	0	0	0	1	0	1
mmu-miR-223-3p	74413	Tc2n	0	0	0	1	0	1
mmu-miR-223-3p	74414	Polr3c	0	0	0	1	0	1

mmu-miR-223-3p	74419	Tktl2	0	0	0	1	0	1
mmu-miR-223-3p	74424	Tmc5	0	0	0	1	0	1
mmu-miR-223-3p	74426	4933402D24Rik	0	0	0	1	0	1
mmu-miR-223-3p	74434	Sohlh2	0	0	0	1	0	1
mmu-miR-223-3p	74440	Cmip	0	0	0	1	0	1
mmu-miR-223-3p	74467	Pus10	0	0	0	1	0	1
mmu-miR-223-3p	74472	4933433C11Rik	0	0	0	1	0	1
mmu-miR-223-3p	74477	4933427D14Rik	0	0	0	1	0	1
mmu-miR-223-3p	74478	Snx29	0	0	0	1	0	1
mmu-miR-223-3p	74479	Snx11	0	0	0	1	0	1
mmu-miR-223-3p	74482	Ifitm7	0	0	0	1	0	1
mmu-miR-223-3p	74485	Lrrc71	0	0	0	1	0	1
mmu-miR-223-3p	74490	Mamstr	1	0	0	0	0	1
mmu-miR-223-3p	74493	Tnks2	0	0	0	1	0	1
mmu-miR-223-3p	74498	Gorasp1	0	0	0	1	0	1
mmu-miR-223-3p	74511	Lrrc17	0	0	0	1	0	1
mmu-miR-223-3p	74519	Cyp2j9	1	0	0	0	0	1
mmu-miR-223-3p	74522	Morc2a	0	0	0	1	0	1
mmu-miR-223-3p	74525	8430419L09Rik	0	0	0	1	0	1
mmu-miR-223-3p	74528	Mgme1	0	0	1	0	0	1
mmu-miR-223-3p	74548	Gsdmc4	0	0	0	1	0	1
mmu-miR-223-3p	74558	Gvin1	0	0	0	1	0	1
mmu-miR-223-3p	74568	Mlkl	0	0	0	1	0	1
mmu-miR-223-3p	74596	Cds1	0	0	0	1	0	1
mmu-miR-223-3p	74600	Mrpl47	0	1	0	0	0	1
mmu-miR-223-3p	74610	Abcb8	0	0	0	1	0	1
mmu-miR-223-3p	74616	Scrn3	0	0	0	1	0	1
mmu-miR-223-3p	74617	Scpep1	0	0	0	1	0	1
mmu-miR-223-3p	74626	Tmem81	0	0	0	1	0	1
mmu-miR-223-3p	74646	Spsb1	0	0	0	1	0	1
mmu-miR-223-3p	74648	S100pbp	0	1	0	0	0	1
mmu-miR-223-3p	74653	Pomk	0	0	0	1	0	1
mmu-miR-223-3p	74665	Lrrc48	0	0	0	1	0	1
mmu-miR-223-3p	74675	Ptchd3	0	0	0	1	0	1
mmu-miR-223-3p	74682	Wdr35	0	0	0	1	0	1
mmu-miR-223-3p	74685	4930451C15Rik	0	0	0	1	0	1
mmu-miR-223-3p	74686	Slc25a54	1	0	0	0	0	1
mmu-miR-223-3p	74691	Tdrd9	0	0	0	1	0	1
mmu-miR-223-3p	74695	4930510E17Rik	0	0	0	1	0	1
mmu-miR-223-3p	74703	Ccdc7	0	0	0	1	0	1
mmu-miR-223-3p	74708	Pih1d3	0	0	0	1	0	1
mmu-miR-223-3p	74709	4930505O20Rik	0	0	0	1	0	1
mmu-miR-223-3p	74717	Spata17	0	0	0	1	0	1
mmu-miR-223-3p	74720	Cldn26	0	0	0	1	0	1
mmu-miR-223-3p	74732	Stx11	0	0	0	1	0	1
mmu-miR-223-3p	74741	C2cd5	0	0	0	1	0	1
mmu-miR-223-3p	74761	Mxra8	0	0	0	1	0	1
mmu-miR-223-3p	74766	Yipf2	0	0	0	1	0	1
mmu-miR-223-3p	74772	Atp13a2	0	0	0	1	0	1
mmu-miR-223-3p	74775	Lmbr1l	0	0	0	1	0	1
mmu-miR-223-3p	74840	Manf	0	1	0	0	0	1
mmu-miR-223-3p	74841	Usp38	0	0	0	1	0	1
mmu-miR-223-3p	74843	Mss51	0	0	0	1	0	1
mmu-miR-223-3p	74854	4930402F06Rik	0	0	0	1	0	1
mmu-miR-223-3p	74895	Ccdc181	0	0	0	1	0	1

mmu-miR-223-3p	74901	Kbtbd11	0	0	0	1	0	1
mmu-miR-223-3p	74918	Iqca	0	0	0	1	0	1
mmu-miR-223-3p	74954	4930503E14Rik	0	0	0	1	0	1
mmu-miR-223-3p	74972	4930500A05Rik	0	0	0	1	0	1
mmu-miR-223-3p	74987	4930468A15Rik	0	0	0	1	0	1
mmu-miR-223-3p	74996	Usp47	0	0	0	1	0	1
mmu-miR-223-3p	75007	Fam63a	0	0	0	1	0	1
mmu-miR-223-3p	75010	4930511M11Rik	0	0	0	1	0	1
mmu-miR-223-3p	75011	4930488N24Rik	0	0	0	1	0	1
mmu-miR-223-3p	75013	4930502E18Rik	0	0	0	1	0	1
mmu-miR-223-3p	75019	Rnase10	0	0	0	1	0	1
mmu-miR-223-3p	75039	4930505H01Rik	0	0	0	1	0	1
mmu-miR-223-3p	75050	Kif27	0	0	0	1	0	1
mmu-miR-223-3p	75071	4930524N10Rik	0	0	0	1	0	1
mmu-miR-223-3p	75089	Uhrf1bp1l	0	0	0	1	0	1
mmu-miR-223-3p	75106	4930519F16Rik	0	0	0	1	0	1
mmu-miR-223-3p	75122	Zc2hc1b	0	0	0	1	0	1
mmu-miR-223-3p	75125	4930513O06Rik	0	0	0	1	0	1
mmu-miR-223-3p	75141	Rasd2	0	0	0	1	0	1
mmu-miR-223-3p	75146	Tmem180	0	0	0	1	0	1
mmu-miR-223-3p	75172	Ccdc146	0	0	0	1	0	1
mmu-miR-223-3p	75180	4930538K18Rik	0	0	0	1	0	1
mmu-miR-223-3p	75284	Bcdin3d	0	0	0	1	0	1
mmu-miR-223-3p	75291	Zbtb3	0	0	0	1	0	1
mmu-miR-223-3p	75300	4930548F15Rik	0	0	0	1	0	1
mmu-miR-223-3p	75304	4930563E22Rik	0	0	0	1	0	1
mmu-miR-223-3p	75316	Taf1d	0	0	0	1	0	1
mmu-miR-223-3p	75317	Parpbp	0	0	0	1	0	1
mmu-miR-223-3p	75339	Mphosph8	0	0	0	1	0	1
mmu-miR-223-3p	75342	4930556J24Rik	0	0	0	1	0	1
mmu-miR-223-3p	75345	Slamf7	0	0	0	1	0	1
mmu-miR-223-3p	75352	4930550L24Rik	0	0	1	0	0	1
mmu-miR-223-3p	75373	4930597O21Rik	1	0	0	0	0	1
mmu-miR-223-3p	75388	Boll	0	1	0	0	0	1
mmu-miR-223-3p	75409	Slitrk5	0	0	0	1	0	1
mmu-miR-223-3p	75430	Anapc15	0	0	0	1	0	1
mmu-miR-223-3p	75434	1700001C02Rik	0	0	0	1	0	1
mmu-miR-223-3p	75439	Cypt12	0	0	0	1	0	1
mmu-miR-223-3p	75452	Ascc2	0	0	0	1	0	1
mmu-miR-223-3p	75454	Phpt1	0	0	0	1	0	1
mmu-miR-223-3p	75458	Cklf	0	0	0	1	0	1
mmu-miR-223-3p	75462	1700001C19Rik	0	0	0	1	0	1
mmu-miR-223-3p	75467	1700011E24Rik	0	0	0	1	0	1
mmu-miR-223-3p	75477	Pfn3	0	0	0	1	0	1
mmu-miR-223-3p	75507	Pou5f2	0	0	0	1	0	1
mmu-miR-223-3p	75512	Gpx6	0	0	0	1	0	1
mmu-miR-223-3p	75516	Ttc32	0	0	0	1	0	1
mmu-miR-223-3p	75524	1700018C11Rik	0	0	0	1	0	1
mmu-miR-223-3p	75526	Eppin	0	0	0	1	0	1
mmu-miR-223-3p	75547	Akap13	0	0	0	1	0	1
mmu-miR-223-3p	75556	1700026D08Rik	0	0	0	1	0	1
mmu-miR-223-3p	75560	Ep400	0	0	0	1	0	1
mmu-miR-223-3p	75563	Dnali1	0	0	0	1	0	1
mmu-miR-223-3p	75565	Ccdc101	0	0	0	1	0	1
mmu-miR-223-3p	75568	Capsl	0	0	0	1	0	1

mmu-miR-223-3p	75570	Nhej1	0	0	0	1	0	1
mmu-miR-223-3p	75572	Acyp2	1	0	0	0	0	1
mmu-miR-223-3p	75573	2310007L24Rik	0	0	0	1	0	1
mmu-miR-223-3p	75577	Dynap	0	0	0	1	0	1
mmu-miR-223-3p	75578	Fggy	0	0	0	1	0	1
mmu-miR-223-3p	75586	Krtap9-3	0	0	0	1	0	1
mmu-miR-223-3p	75593	Malsu1	0	1	0	0	0	1
mmu-miR-223-3p	75600	Calml4	0	0	0	1	0	1
mmu-miR-223-3p	75604	Tm4sf5	0	0	0	1	0	1
mmu-miR-223-3p	75607	Wnk2	0	1	0	0	0	1
mmu-miR-223-3p	75610	2010109A12Rik	0	0	0	1	0	1
mmu-miR-223-3p	75617	Rps25	0	0	0	1	0	1
mmu-miR-223-3p	75620	Kxd1	0	0	0	1	0	1
mmu-miR-223-3p	75622	Spaca3	0	0	0	1	0	1
mmu-miR-223-3p	75623	Tex30	0	0	0	1	0	1
mmu-miR-223-3p	75625	Mageh1	0	0	0	1	0	1
mmu-miR-223-3p	75627	Snapc1	0	0	0	1	0	1
mmu-miR-223-3p	75645	Ccdc172	0	0	0	1	0	1
mmu-miR-223-3p	75657	Speer4a	0	0	0	1	0	1
mmu-miR-223-3p	75660	Lin37	0	0	0	1	0	1
mmu-miR-223-3p	75668	Rasl10a	0	0	0	1	0	1
mmu-miR-223-3p	75677	Cldn22	0	0	0	1	0	1
mmu-miR-223-3p	75678	Ippk	0	0	0	1	0	1
mmu-miR-223-3p	75687	Fam65a	0	0	0	1	0	1
mmu-miR-223-3p	75691	Anks6	0	0	0	1	0	1
mmu-miR-223-3p	75692	Nr2c2ap	0	0	0	1	0	1
mmu-miR-223-3p	75704	Ces1h	0	0	0	1	0	1
mmu-miR-223-3p	75712	Tmem14a	0	0	0	1	0	1
mmu-miR-223-3p	75718	Vwa5b1	0	1	0	0	0	1
mmu-miR-223-3p	75721	4932414N04Rik	0	1	0	0	0	1
mmu-miR-223-3p	75729	Fam227a	0	0	0	1	0	1
mmu-miR-223-3p	75731	Idnk	0	0	0	1	0	1
mmu-miR-223-3p	75732	Iqcd	1	0	0	0	0	1
mmu-miR-223-3p	75734	Mff	0	0	0	1	0	1
mmu-miR-223-3p	75740	Egfem1	0	0	0	1	0	1
mmu-miR-223-3p	75746	Morc4	0	0	0	1	0	1
mmu-miR-223-3p	75747	Sesn3	0	0	0	1	0	1
mmu-miR-223-3p	75750	Slc10a6	0	0	0	1	0	1
mmu-miR-223-3p	75751	Ipo4	0	1	0	0	0	1
mmu-miR-223-3p	75777	Ttc23l	0	0	0	1	0	1
mmu-miR-223-3p	75788	Smurf1	0	0	0	1	0	1
mmu-miR-223-3p	75820	Wdr64	0	0	0	1	0	1
mmu-miR-223-3p	75861	4930571K23Rik	0	0	0	1	0	1
mmu-miR-223-3p	75870	Tcam1	1	0	0	0	0	1
mmu-miR-223-3p	75871	Zfp821	0	0	0	1	0	1
mmu-miR-223-3p	75873	4930568B11Rik	0	0	0	1	0	1
mmu-miR-223-3p	75894	Adal	0	0	0	1	0	1
mmu-miR-223-3p	75906	Fam184a	0	0	0	1	0	1
mmu-miR-223-3p	75939	4930579G24Rik	1	0	0	0	0	1
mmu-miR-223-3p	75956	Srrm2	0	0	0	1	0	1
mmu-miR-223-3p	75964	Trappc8	0	0	0	1	0	1
mmu-miR-223-3p	75986	Agmat	0	0	0	1	0	1
mmu-miR-223-3p	76002	Ms4a18	1	0	0	0	0	1
mmu-miR-223-3p	76014	Zc3h18	0	0	0	1	0	1
mmu-miR-223-3p	76022	Gon4l	0	0	0	1	0	1

mmu-miR-223-3p	76025	Cant1	0	0	0	1	0	1
mmu-miR-223-3p	76030	5830433I10Rik	0	0	0	1	0	1
mmu-miR-223-3p	76041	Ccdc125	0	0	0	1	0	1
mmu-miR-223-3p	76072	Rnf183	0	0	0	1	0	1
mmu-miR-223-3p	76074	Gbp8	0	0	0	1	0	1
mmu-miR-223-3p	76130	Las1l	0	0	0	1	0	1
mmu-miR-223-3p	76131	Depdc1a	0	0	0	1	0	1
mmu-miR-223-3p	76142	Ppp1r14c	0	0	0	1	0	1
mmu-miR-223-3p	76156	Fam131b	0	0	0	1	0	1
mmu-miR-223-3p	76161	Lamp5	0	0	0	1	0	1
mmu-miR-223-3p	76183	Celf6	1	0	0	0	0	1
mmu-miR-223-3p	76184	Abca6	1	0	0	0	0	1
mmu-miR-223-3p	76187	Adhfe1	0	0	0	1	0	1
mmu-miR-223-3p	76192	Abhd12	0	0	0	1	0	1
mmu-miR-223-3p	76223	Agbl3	0	1	0	0	0	1
mmu-miR-223-3p	76227	6530403G13Rik	0	0	0	1	0	1
mmu-miR-223-3p	76237	6430628N08Rik	0	0	0	1	0	1
mmu-miR-223-3p	76257	Slc38a3	0	0	0	1	0	1
mmu-miR-223-3p	76261	0610040J01Rik	0	0	0	1	0	1
mmu-miR-223-3p	76263	Gstk1	0	0	0	1	0	1
mmu-miR-223-3p	76273	Ndfip2	0	0	0	1	0	1
mmu-miR-223-3p	76279	Cyp2d26	0	0	0	1	0	1
mmu-miR-223-3p	76282	Gpt	1	0	0	0	0	1
mmu-miR-223-3p	76293	Mfap4	0	0	0	1	0	1
mmu-miR-223-3p	76294	Asb5	0	0	0	1	0	1
mmu-miR-223-3p	76308	Rab1b	0	0	0	1	0	1
mmu-miR-223-3p	76332	Cog2	0	0	0	1	0	1
mmu-miR-223-3p	76355	Tgds	0	0	0	1	0	1
mmu-miR-223-3p	76375	Det1	0	0	0	1	0	1
mmu-miR-223-3p	76380	Cep112	0	0	0	1	0	1
mmu-miR-223-3p	76390	Zfp735	0	0	0	1	0	1
mmu-miR-223-3p	76399	Il31	0	0	0	1	0	1
mmu-miR-223-3p	76405	1700018B08Rik	0	0	0	1	0	1
mmu-miR-223-3p	76408	Abcc3	0	0	0	1	0	1
mmu-miR-223-3p	76421	1700028K03Rik	0	0	0	1	0	1
mmu-miR-223-3p	76426	Fam209	0	0	0	1	0	1
mmu-miR-223-3p	76438	Rftn1	0	0	0	1	0	1
mmu-miR-223-3p	76454	Fbxo31	0	0	0	1	0	1
mmu-miR-223-3p	76469	Cmya5	0	0	0	1	0	1
mmu-miR-223-3p	76477	Pcolce2	0	0	0	1	0	1
mmu-miR-223-3p	76485	Glt8d1	0	0	0	1	0	1
mmu-miR-223-3p	76487	Ppp1r3g	0	0	0	1	0	1
mmu-miR-223-3p	76491	Abhd14b	0	0	0	1	0	1
mmu-miR-223-3p	76500	Ip6k2	0	0	0	1	0	1
mmu-miR-223-3p	76501	Commd9	0	0	0	1	0	1
mmu-miR-223-3p	76507	Aoc1	0	0	0	1	0	1
mmu-miR-223-3p	76522	Naa38	0	0	0	1	0	1
mmu-miR-223-3p	76539	D19Ertd737e	0	0	0	1	0	1
mmu-miR-223-3p	76547	Tmem101	0	0	0	1	0	1
mmu-miR-223-3p	76561	Snx7	0	0	0	1	0	1
mmu-miR-223-3p	76563	Qrsl1	0	0	0	1	0	1
mmu-miR-223-3p	76572	Rbmxl2	0	0	0	1	0	1
mmu-miR-223-3p	76580	Mib2	0	0	0	1	0	1
mmu-miR-223-3p	76589	Unc5cl	0	0	0	1	0	1
mmu-miR-223-3p	76612	Lrrc27	0	0	0	1	0	1

mmu-miR-223-3p	76630	Stambpl1	0	0	0	1	0	1
mmu-miR-223-3p	76640	1700113H08Rik	0	0	0	1	0	1
mmu-miR-223-3p	76652	Actrt3	0	0	0	1	0	1
mmu-miR-223-3p	76654	Upp2	0	0	0	1	0	1
mmu-miR-223-3p	76658	1700123K08Rik	0	0	0	1	0	1
mmu-miR-223-3p	76670	Ttc18	1	0	0	0	0	1
mmu-miR-223-3p	76686	Clip3	0	0	0	1	0	1
mmu-miR-223-3p	76703	Cpb1	0	1	0	0	0	1
mmu-miR-223-3p	76713	1700039E15Rik	0	0	0	1	0	1
mmu-miR-223-3p	76722	Ckmt2	0	0	0	1	0	1
mmu-miR-223-3p	76737	Creld2	0	0	0	1	0	1
mmu-miR-223-3p	76757	Trdn	0	0	0	1	0	1
mmu-miR-223-3p	76763	Mospd2	0	0	0	1	0	1
mmu-miR-223-3p	76779	Cluap1	0	1	0	0	0	1
mmu-miR-223-3p	76784	Mtif2	0	0	0	1	0	1
mmu-miR-223-3p	76787	Ppfia3	0	0	0	1	0	1
mmu-miR-223-3p	76795	Tbc1d9b	0	0	0	1	0	1
mmu-miR-223-3p	76799	Tmem234	0	0	0	1	0	1
mmu-miR-223-3p	76808	Rpl18a	0	0	0	1	0	1
mmu-miR-223-3p	76815	Calcoco2	0	0	0	1	0	1
mmu-miR-223-3p	76826	Nubpl	0	1	0	0	0	1
mmu-miR-223-3p	76832	Hyls1	0	0	0	1	0	1
mmu-miR-223-3p	76846	Rps9	0	0	0	1	0	1
mmu-miR-223-3p	76863	Dcun1d5	0	0	0	1	0	1
mmu-miR-223-3p	76867	Rhbdd1	0	0	0	1	0	1
mmu-miR-223-3p	76872	Ccdc116	0	0	0	1	0	1
mmu-miR-223-3p	76884	Cyfip2	0	0	0	1	0	1
mmu-miR-223-3p	76889	Adck4	0	0	0	1	0	1
mmu-miR-223-3p	76890	Memo1	0	0	0	1	0	1
mmu-miR-223-3p	76893	Cers2	0	0	1	0	0	1
mmu-miR-223-3p	76894	Mettl15	0	0	0	1	0	1
mmu-miR-223-3p	76901	Phf15	0	0	0	1	0	1
mmu-miR-223-3p	76915	Mnd1	0	0	0	1	0	1
mmu-miR-223-3p	76925	1700015E13Rik	0	0	0	1	0	1
mmu-miR-223-3p	76936	Hnrnpm	0	0	0	1	0	1
mmu-miR-223-3p	76938	Rbm17	0	0	0	1	0	1
mmu-miR-223-3p	76947	Ndufaf6	0	0	0	1	0	1
mmu-miR-223-3p	76952	Nt5c2	0	0	0	1	0	1
mmu-miR-223-3p	76954	St5	0	0	0	1	0	1
mmu-miR-223-3p	76959	Chmp5	0	0	0	1	0	1
mmu-miR-223-3p	76967	2700049A03Rik	0	0	0	1	0	1
mmu-miR-223-3p	76969	Chst1	0	0	0	1	0	1
mmu-miR-223-3p	76971	2810007J24Rik	0	0	0	1	0	1
mmu-miR-223-3p	76976	Arxes2	0	0	0	1	0	1
mmu-miR-223-3p	76998	Fbxw27	0	0	0	1	0	1
mmu-miR-223-3p	77006	Ddrgk1	0	1	0	0	0	1
mmu-miR-223-3p	77034	2510039O18Rik	0	0	0	1	0	1
mmu-miR-223-3p	77038	Arfgap2	0	0	0	1	0	1
mmu-miR-223-3p	77042	Hyal4	0	0	0	1	0	1
mmu-miR-223-3p	77049	4921528I07Rik	0	0	0	1	0	1
mmu-miR-223-3p	77056	Tmco4	0	0	0	1	0	1
mmu-miR-223-3p	77057	Ston1	0	0	0	1	0	1
mmu-miR-223-3p	77065	Ints7	0	0	0	1	0	1
mmu-miR-223-3p	77080	9230110F15Rik	0	0	0	1	0	1
mmu-miR-223-3p	77087	Ankrd11	0	0	0	1	0	1

mmu-miR-223-3p	77090	Ocel1	0	1	0	0	0	1
mmu-miR-223-3p	77110	Gpbp1l1	1	0	0	0	0	1
mmu-miR-223-3p	77117	Zfp934	0	0	0	1	0	1
mmu-miR-223-3p	77134	Hnrnpa0	1	0	0	0	0	1
mmu-miR-223-3p	77220	Tmem200a	0	0	0	1	0	1
mmu-miR-223-3p	77286	Nkrf	0	0	0	1	0	1
mmu-miR-223-3p	77358	9430069I07Rik	0	0	0	1	0	1
mmu-miR-223-3p	77411	Esrp2	0	0	0	1	0	1
mmu-miR-223-3p	77422	C330018D20Rik	0	0	0	1	0	1
mmu-miR-223-3p	77432	9530002B09Rik	1	0	0	0	0	1
mmu-miR-223-3p	77481	C030048H21Rik	0	0	0	1	0	1
mmu-miR-223-3p	77552	Shisa4	0	0	0	1	0	1
mmu-miR-223-3p	77573	Vps33a	0	0	0	1	0	1
mmu-miR-223-3p	77574	Fam115a	0	0	1	0	0	1
mmu-miR-223-3p	77577	Spns3	0	0	0	1	0	1
mmu-miR-223-3p	77578	Bcl9	0	0	0	1	0	1
mmu-miR-223-3p	77583	Notum	0	0	0	1	0	1
mmu-miR-223-3p	77596	Gpr110	1	0	0	0	0	1
mmu-miR-223-3p	77605	H2afv	0	0	0	1	0	1
mmu-miR-223-3p	77609	Ccdc151	0	0	0	1	0	1
mmu-miR-223-3p	77613	Prss36	0	0	0	1	0	1
mmu-miR-223-3p	77626	Smpd4	0	0	0	1	0	1
mmu-miR-223-3p	77627	Efcab6	0	0	0	1	0	1
mmu-miR-223-3p	77630	Prdm8	0	0	0	1	0	1
mmu-miR-223-3p	77634	Snapc3	0	1	0	0	0	1
mmu-miR-223-3p	77683	Ehmt1	0	0	0	1	0	1
mmu-miR-223-3p	77705	9230104L09Rik	0	0	0	1	0	1
mmu-miR-223-3p	77721	Mrps5	0	0	0	1	0	1
mmu-miR-223-3p	77727	6030468B19Rik	0	0	0	1	0	1
mmu-miR-223-3p	77733	Rnf170	0	0	0	1	0	1
mmu-miR-223-3p	77782	Polq	0	0	0	1	0	1
mmu-miR-223-3p	77798	A930009A15Rik	0	0	0	1	0	1
mmu-miR-223-3p	77803	Fam159b	1	0	0	0	0	1
mmu-miR-223-3p	77805	Esco1	0	0	0	1	0	1
mmu-miR-223-3p	77827	Krba1	0	0	0	1	0	1
mmu-miR-223-3p	77908	9230113P08Rik	0	0	0	1	0	1
mmu-miR-223-3p	77914	Krtap17-1	0	0	0	1	0	1
mmu-miR-223-3p	77917	A030014E15Rik	0	0	0	1	0	1
mmu-miR-223-3p	77922	A030005L19Rik	0	0	0	1	0	1
mmu-miR-223-3p	77945	Rpgrip1	0	0	0	1	0	1
mmu-miR-223-3p	77975	Tmem50b	0	0	0	1	0	1
mmu-miR-223-3p	77987	Ascc3	0	0	0	1	0	1
mmu-miR-223-3p	78004	Prr15	0	0	0	1	0	1
mmu-miR-223-3p	78016	Ccdc150	0	0	0	1	0	1
mmu-miR-223-3p	78070	Cpt1c	0	0	0	1	0	1
mmu-miR-223-3p	78100	Msantd4	0	0	0	1	0	1
mmu-miR-223-3p	78128	Spag11a	0	0	0	1	0	1
mmu-miR-223-3p	78134	Lpar4	0	0	0	1	0	1
mmu-miR-223-3p	78177	Ninl	0	0	0	1	0	1
mmu-miR-223-3p	78249	Gpr115	0	0	0	1	0	1
mmu-miR-223-3p	78251	Zfp712	0	0	0	1	0	1
mmu-miR-223-3p	78266	Zfp687	0	0	0	1	0	1
mmu-miR-223-3p	78283	Map7d2	0	0	0	1	0	1
mmu-miR-223-3p	78284	Creb3l4	0	0	0	1	0	1
mmu-miR-223-3p	78286	Nav2	0	0	0	1	0	1

mmu-miR-223-3p	78317	Ccdc88b	0	0	0	1	0	1
mmu-miR-223-3p	78323	Fam219b	0	0	0	1	0	1
mmu-miR-223-3p	78330	Ndufv3	0	0	0	1	0	1
mmu-miR-223-3p	78376	Sapcd1	0	0	0	1	0	1
mmu-miR-223-3p	78382	Lce6a	0	0	0	1	0	1
mmu-miR-223-3p	78390	Pla2g4d	0	0	0	1	0	1
mmu-miR-223-3p	78394	Ddx52	0	0	0	1	0	1
mmu-miR-223-3p	78405	Ntf5	0	0	0	1	0	1
mmu-miR-223-3p	78408	Fam131a	0	0	0	1	0	1
mmu-miR-223-3p	78412	3110062M04Rik	0	0	0	1	0	1
mmu-miR-223-3p	78428	Wibg	0	0	0	1	0	1
mmu-miR-223-3p	78459	1700057G04Rik	0	0	0	1	0	1
mmu-miR-223-3p	78465	1700084C01Rik	0	0	0	1	0	1
mmu-miR-223-3p	78478	Tmsb15a	0	0	0	1	0	1
mmu-miR-223-3p	78506	Micu3	0	0	0	1	0	1
mmu-miR-223-3p	78514	Arhgap10	0	0	0	1	0	1
mmu-miR-223-3p	78586	Srbd1	0	1	0	0	0	1
mmu-miR-223-3p	78600	Pde6h	0	1	0	0	0	1
mmu-miR-223-3p	78610	Uvrag	0	0	0	1	0	1
mmu-miR-223-3p	78617	Cstad	0	0	0	1	0	1
mmu-miR-223-3p	78619	Zfp449	0	0	0	1	0	1
mmu-miR-223-3p	78634	Spaca7	0	0	0	1	0	1
mmu-miR-223-3p	78670	Plekhj1	0	0	0	1	0	1
mmu-miR-223-3p	78697	Pus7	0	0	0	1	0	1
mmu-miR-223-3p	78733	Troap	0	0	0	1	0	1
mmu-miR-223-3p	78752	Csgalnact2	0	0	0	1	0	1
mmu-miR-223-3p	78767	Efcab11	1	0	0	0	0	1
mmu-miR-223-3p	78772	Hhipl2	0	0	0	1	0	1
mmu-miR-223-3p	78779	Spata2l	0	0	0	1	0	1
mmu-miR-223-3p	78781	Zc3hav1	0	0	0	1	0	1
mmu-miR-223-3p	78784	Celf3	0	0	0	1	0	1
mmu-miR-223-3p	78785	Clip4	0	0	0	1	0	1
mmu-miR-223-3p	78796	Zcchc4	0	1	0	0	0	1
mmu-miR-223-3p	78801	Ak7	1	0	0	0	0	1
mmu-miR-223-3p	78816	Gmip	0	0	0	1	0	1
mmu-miR-223-3p	78826	P2ry10	1	0	0	0	0	1
mmu-miR-223-3p	78829	Tsc22d4	0	0	0	1	0	1
mmu-miR-223-3p	78833	Gins3	1	0	0	0	0	1
mmu-miR-223-3p	78834	Zfp623	0	0	0	1	0	1
mmu-miR-223-3p	78890	Trmt44	0	0	0	1	0	1
mmu-miR-223-3p	78891	Scyl1	1	0	0	0	0	1
mmu-miR-223-3p	78892	Crispld2	0	0	0	1	0	1
mmu-miR-223-3p	78895	Pus7l	0	0	0	1	0	1
mmu-miR-223-3p	78903	Wrnip1	0	0	0	1	0	1
mmu-miR-223-3p	78906	Misp	0	0	0	1	0	1
mmu-miR-223-3p	78911	Trim42	0	1	0	0	0	1
mmu-miR-223-3p	78919	Fndc8	0	0	0	1	0	1
mmu-miR-223-3p	78923	Chsy3	1	0	0	0	0	1
mmu-miR-223-3p	78933	Agbl4	0	0	0	1	0	1
mmu-miR-223-3p	78937	Avl9	0	0	0	1	0	1
mmu-miR-223-3p	78938	Fbxo34	0	0	0	1	0	1
mmu-miR-223-3p	78977	Popdc3	0	0	0	1	0	1
mmu-miR-223-3p	79043	Spsb3	0	0	0	1	0	1
mmu-miR-223-3p	79044	Mrps34	0	0	0	1	0	1
mmu-miR-223-3p	79059	Nme3	0	0	0	1	0	1

mmu-miR-223-3p	79196	Osbpl5	0	0	0	1	0	1
mmu-miR-223-3p	79464	Lias	0	0	0	1	0	1
mmu-miR-223-3p	79560	Ublcp1	0	0	0	1	0	1
mmu-miR-223-3p	80280	Cdk5rap3	0	0	0	1	0	1
mmu-miR-223-3p	80284	Smim12	0	0	0	1	0	1
mmu-miR-223-3p	80294	Pofut2	0	0	1	0	0	1
mmu-miR-223-3p	80297	Sptbn4	0	0	0	1	0	1
mmu-miR-223-3p	80384	Tex21	0	0	0	1	0	1
mmu-miR-223-3p	80509	Med8	0	0	0	1	0	1
mmu-miR-223-3p	80517	Herpud2	0	0	0	1	0	1
mmu-miR-223-3p	80708	Pacsin3	0	0	0	1	0	1
mmu-miR-223-3p	80721	Slc19a3	0	0	0	1	0	1
mmu-miR-223-3p	80743	Vps16	0	0	0	1	0	1
mmu-miR-223-3p	80744	Cwc22	0	0	0	1	0	1
mmu-miR-223-3p	80750	N4bp1	0	0	0	1	0	1
mmu-miR-223-3p	80752	Fam20c	0	0	0	1	0	1
mmu-miR-223-3p	80795	Selk	0	0	0	1	0	1
mmu-miR-223-3p	80796	Calm4	0	0	0	1	0	1
mmu-miR-223-3p	80797	Clca2	0	0	0	1	0	1
mmu-miR-223-3p	80857	Fgf20	0	0	0	1	0	1
mmu-miR-223-3p	80859	Nfkbiz	0	0	0	1	0	1
mmu-miR-223-3p	80861	Dhx58	1	0	0	0	0	1
mmu-miR-223-3p	80884	Maged2	0	1	0	0	0	1
mmu-miR-223-3p	80885	Niacr1	0	0	0	1	0	1
mmu-miR-223-3p	80886	Senp3	0	0	0	1	0	1
mmu-miR-223-3p	80888	Hspb8	0	0	0	1	0	1
mmu-miR-223-3p	80893	Tmprss5	0	1	0	0	0	1
mmu-miR-223-3p	80898	Erap1	0	0	0	1	0	1
mmu-miR-223-3p	80901	Cxcr6	0	0	0	1	0	1
mmu-miR-223-3p	80905	Polh	0	0	0	1	0	1
mmu-miR-223-3p	80906	Kcnip2	0	0	0	1	0	1
mmu-miR-223-3p	80907	Lactb	0	0	0	1	0	1
mmu-miR-223-3p	80914	Uck2	1	0	0	0	0	1
mmu-miR-223-3p	80982	9930013L23Rik	0	0	0	1	0	1
mmu-miR-223-3p	80987	Nckipsd	0	0	0	1	0	1
mmu-miR-223-3p	81011	Vmn1r148	0	0	0	1	0	1
mmu-miR-223-3p	81012	Vmn1r171	0	0	0	1	0	1
mmu-miR-223-3p	81014	Vmn1r58	0	0	0	1	0	1
mmu-miR-223-3p	81016	Vmn1r62	0	0	0	1	0	1
mmu-miR-223-3p	81017	Vmn1r63	0	0	0	1	0	1
mmu-miR-223-3p	81018	Rnf114	0	0	0	1	0	1
mmu-miR-223-3p	81489	Dnajb1	0	0	0	1	0	1
mmu-miR-223-3p	81535	Sgpp1	0	0	0	1	0	1
mmu-miR-223-3p	81600	Chia	0	0	0	1	0	1
mmu-miR-223-3p	81601	Kat5	0	0	0	1	0	1
mmu-miR-223-3p	81630	Zbtb22	0	0	0	1	0	1
mmu-miR-223-3p	81845	Gpank1	0	0	0	1	0	1
mmu-miR-223-3p	81877	Tnxb	1	0	0	0	0	1
mmu-miR-223-3p	81897	Tlr9	0	0	0	1	0	1
mmu-miR-223-3p	81898	Sf3b1	0	0	0	1	0	1
mmu-miR-223-3p	81907	Tmem108	1	0	0	0	0	1
mmu-miR-223-3p	81909	Zfpl1	0	0	0	1	0	1
mmu-miR-223-3p	83382	Siglece	0	0	0	1	0	1
mmu-miR-223-3p	83395	Sp6	0	0	0	1	0	1
mmu-miR-223-3p	83396	Glis2	0	0	1	0	0	1

mmu-miR-223-3p	83431	Ndel1	0	0	0	1	0	1
mmu-miR-223-3p	83433	Trem2	0	0	0	1	0	1
mmu-miR-223-3p	83434	Rsph6a	0	0	0	1	0	1
mmu-miR-223-3p	83457	Fthl17	0	0	0	1	0	1
mmu-miR-223-3p	83485	Ngrn	0	0	0	1	0	1
mmu-miR-223-3p	83490	Pik3ap1	0	0	0	1	0	1
mmu-miR-223-3p	83492	Gsdmc	0	0	0	1	0	1
mmu-miR-223-3p	83553	Tktl1	0	0	0	1	0	1
mmu-miR-223-3p	83554	Fstl3	0	0	0	1	0	1
mmu-miR-223-3p	83557	Lin28a	0	0	0	1	0	1
mmu-miR-223-3p	83558	Tex11	0	0	0	1	0	1
mmu-miR-223-3p	83561	Tdrd1	0	0	0	1	0	1
mmu-miR-223-3p	83563	Usp26	0	0	0	1	0	1
mmu-miR-223-3p	83564	Nlrp4c	0	0	0	1	0	1
mmu-miR-223-3p	83669	Wdr6	0	0	0	1	0	1
mmu-miR-223-3p	83762	Otof	0	0	0	1	0	1
mmu-miR-223-3p	83768	Dpp7	1	0	0	0	0	1
mmu-miR-223-3p	83770	Tas1r2	0	0	0	1	0	1
mmu-miR-223-3p	83885	Slc25a2	0	0	0	1	0	1
mmu-miR-223-3p	83964	Jam3	0	0	0	1	0	1
mmu-miR-223-3p	83965	Enpp5	0	0	0	1	0	1
mmu-miR-223-3p	83993	Tbx19	0	0	0	1	0	1
mmu-miR-223-3p	83996	Mmp1b	0	0	0	1	0	1
mmu-miR-223-3p	84004	Mcam	0	0	0	1	0	1
mmu-miR-223-3p	84036	Kcnn1	0	0	0	1	0	1
mmu-miR-223-3p	84092	Usp8	0	0	0	1	0	1
mmu-miR-223-3p	84094	Plvap	0	0	0	1	0	1
mmu-miR-223-3p	84112	Sucnr1	0	0	0	1	0	1
mmu-miR-223-3p	84113	Ptov1	0	0	0	1	0	1
mmu-miR-223-3p	84585	Rnf123	0	0	0	1	0	1
mmu-miR-223-3p	84682	Cox4i2	1	0	0	0	0	1
mmu-miR-223-3p	84704	Snurf	0	0	0	1	0	1
mmu-miR-223-3p	85030	Tnfrsf25	0	0	0	1	0	1
mmu-miR-223-3p	85308	Emc9	0	0	0	1	0	1
mmu-miR-223-3p	93671	Cd163	0	0	0	1	0	1
mmu-miR-223-3p	93679	Trim8	0	0	0	1	0	1
mmu-miR-223-3p	93686	Rbfox2	0	0	0	1	0	1
mmu-miR-223-3p	93687	Csnk1a1	0	0	1	0	0	1
mmu-miR-223-3p	93689	Lmod1	0	0	0	1	0	1
mmu-miR-223-3p	93691	Klf7	0	1	0	0	0	1
mmu-miR-223-3p	93692	Glrx	0	0	0	1	0	1
mmu-miR-223-3p	93726	Ear11	0	0	0	1	0	1
mmu-miR-223-3p	93732	Acox2	0	0	0	1	0	1
mmu-miR-223-3p	93735	Wnt16	0	1	0	0	0	1
mmu-miR-223-3p	93747	Echs1	0	0	0	1	0	1
mmu-miR-223-3p	93759	Sirt1	0	0	0	1	0	1
mmu-miR-223-3p	93762	Smarca5	0	0	0	1	0	1
mmu-miR-223-3p	93806	Serpinb9g	1	0	0	0	0	1
mmu-miR-223-3p	93838	Dqx1	0	0	1	0	0	1
mmu-miR-223-3p	93873	Pcdhb2	0	0	0	1	0	1
mmu-miR-223-3p	93874	Pcdhb3	0	0	0	1	0	1
mmu-miR-223-3p	93875	Pcdhb4	0	0	0	1	0	1
mmu-miR-223-3p	93876	Pcdhb5	0	0	0	1	0	1
mmu-miR-223-3p	93878	Pcdhb7	0	0	0	1	0	1
mmu-miR-223-3p	93883	Pcdhb12	0	0	0	1	0	1

mmu-miR-223-3p	93884	Pcdhb13	0	0	0	1	0	1
mmu-miR-223-3p	93885	Pcdhb14	0	0	0	1	0	1
mmu-miR-223-3p	93893	Pcdhb22	0	0	0	1	0	1
mmu-miR-223-3p	93897	Fzd10	1	0	0	0	0	1
mmu-miR-223-3p	93968	Klra21	1	0	0	0	0	1
mmu-miR-223-3p	94043	Tm2d1	0	0	0	1	0	1
mmu-miR-223-3p	94063	Mrpl16	0	0	0	1	0	1
mmu-miR-223-3p	94066	Mrpl36	0	0	0	1	0	1
mmu-miR-223-3p	94178	Mcoln1	0	0	0	1	0	1
mmu-miR-223-3p	94181	Nans	0	0	0	1	0	1
mmu-miR-223-3p	94186	Strn3	0	0	0	1	0	1
mmu-miR-223-3p	94190	Ophn1	0	0	0	1	0	1
mmu-miR-223-3p	94192	C1galt1	1	0	0	0	0	1
mmu-miR-223-3p	94214	Spock2	0	0	0	1	0	1
mmu-miR-223-3p	94217	Lrp1b	0	1	0	0	0	1
mmu-miR-223-3p	94218	Cnnm3	0	0	0	1	0	1
mmu-miR-223-3p	94222	Olig3	1	0	0	0	0	1
mmu-miR-223-3p	94242	Tinagl1	0	0	0	1	0	1
mmu-miR-223-3p	94244	Fkbp6	0	0	0	1	0	1
mmu-miR-223-3p	94275	Maged1	0	0	0	1	0	1
mmu-miR-223-3p	94279	Sfxn2	0	0	0	1	0	1
mmu-miR-223-3p	94281	Sfxn4	0	0	0	1	0	1
mmu-miR-223-3p	94315	Prcc	0	0	0	1	0	1
mmu-miR-223-3p	94353	Hmgn3	0	0	0	1	0	1
mmu-miR-223-3p	96875	Prg4	0	0	0	1	0	1
mmu-miR-223-3p	96979	Ptges2	0	0	0	1	0	1
mmu-miR-223-3p	97031	Tprn	0	0	0	1	0	1
mmu-miR-223-3p	97086	Slc9b2	0	1	0	0	0	1
mmu-miR-223-3p	97112	Nmd3	0	0	0	1	0	1
mmu-miR-223-3p	97114	Hist2h3c2	0	0	0	1	0	1
mmu-miR-223-3p	97159	A430005L14Rik	0	0	0	1	0	1
mmu-miR-223-3p	97165	Hmgb2	0	0	0	1	0	1
mmu-miR-223-3p	97187	C87977	0	0	0	1	0	1
mmu-miR-223-3p	97476	C86695	0	0	0	1	0	1
mmu-miR-223-3p	97484	Cog8	0	1	0	0	0	1
mmu-miR-223-3p	97827	Exd2	0	0	0	1	0	1
mmu-miR-223-3p	97863	C78339	0	0	0	1	0	1
mmu-miR-223-3p	97961	Nol12	0	0	0	1	0	1
mmu-miR-223-3p	98170	Tmem132a	0	0	0	1	0	1
mmu-miR-223-3p	98238	Lrrc59	0	0	0	1	0	1
mmu-miR-223-3p	98256	Kmo	0	0	0	1	0	1
mmu-miR-223-3p	98363	Efhd1	0	0	0	1	0	1
mmu-miR-223-3p	98365	Slamf9	0	0	0	1	0	1
mmu-miR-223-3p	98366	Smap1	0	0	0	1	0	1
mmu-miR-223-3p	98376	Gorab	0	0	0	1	0	1
mmu-miR-223-3p	98403	Zfp451	0	1	0	0	0	1
mmu-miR-223-3p	98660	Atp1a2	0	0	0	1	0	1
mmu-miR-223-3p	98710	Rabif	0	0	0	1	0	1
mmu-miR-223-3p	98752	Fcrla	0	0	0	1	0	1
mmu-miR-223-3p	98758	Hnrnpf	0	0	0	1	0	1
mmu-miR-223-3p	98828	Cdc123	0	0	0	1	0	1
mmu-miR-223-3p	99011	Pomt1	0	0	0	1	0	1
mmu-miR-223-3p	99100	Cep152	0	0	0	1	0	1
mmu-miR-223-3p	99151	Cercam	0	0	0	1	0	1
mmu-miR-223-3p	99152	Anapc2	0	0	0	1	0	1

mmu-miR-223-3p	99167	Ssx2ip	0	0	0	1	0	1
mmu-miR-223-3p	99237	Tm9sf4	0	0	0	1	0	1
mmu-miR-223-3p	99296	Hrh3	0	0	0	1	0	1
mmu-miR-223-3p	99349	Dnajc24	1	0	0	0	0	1
mmu-miR-223-3p	99377	Sall4	0	0	0	1	0	1
mmu-miR-223-3p	99412	Golga2	0	0	0	1	0	1
mmu-miR-223-3p	99571	Fgg	0	0	0	1	0	1
mmu-miR-223-3p	99650	4933434E20Rik	0	0	0	1	0	1
mmu-miR-223-3p	99662	Eps8l3	0	0	0	1	0	1
mmu-miR-223-3p	99663	Clca6	0	0	0	1	0	1
mmu-miR-223-3p	99681	Tchh	0	0	0	1	0	1
mmu-miR-223-3p	99730	Taf13	0	0	0	1	0	1
mmu-miR-223-3p	99738	Kcnc4	0	0	0	1	0	1
mmu-miR-223-3p	99890	Prmt6	0	0	0	1	0	1
mmu-miR-223-3p	99982	Kdm1a	0	0	0	1	0	1
mmu-miR-223-3p	100012	Oog3	0	0	0	1	0	1
mmu-miR-223-3p	100090	Zbtb48	0	0	0	1	0	1
mmu-miR-223-3p	100121	Tdrd7	0	0	0	1	0	1
mmu-miR-223-3p	100210	Gpn2	0	0	0	1	0	1
mmu-miR-223-3p	100273	Osbpl9	0	0	0	1	0	1
mmu-miR-223-3p	100317	AU040320	1	0	0	0	0	1
mmu-miR-223-3p	100336	Ppp1r8	0	0	0	1	0	1
mmu-miR-223-3p	100383	Bsdc1	0	0	0	1	0	1
mmu-miR-223-3p	100434	Slc44a1	0	0	0	1	0	1
mmu-miR-223-3p	100559	Ugt2b38	0	0	0	1	0	1
mmu-miR-223-3p	100604	Lrrc8c	0	1	0	0	0	1
mmu-miR-223-3p	100608	Noc4l	0	0	0	1	0	1
mmu-miR-223-3p	100683	Trrap	0	0	0	1	0	1
mmu-miR-223-3p	100689	Spon2	0	0	0	1	0	1
mmu-miR-223-3p	100705	Acacb	1	0	0	0	0	1
mmu-miR-223-3p	100900	Hscb	0	1	0	0	0	1
mmu-miR-223-3p	100929	Tyw1	0	0	0	1	0	1
mmu-miR-223-3p	100972	Rab28	0	0	0	1	0	1
mmu-miR-223-3p	101023	Zfp513	0	0	0	1	0	1
mmu-miR-223-3p	101100	Ttll3	0	0	0	1	0	1
mmu-miR-223-3p	101122	Rpusd3	1	0	0	0	0	1
mmu-miR-223-3p	101148	B630005N14Rik	0	0	0	1	0	1
mmu-miR-223-3p	101202	Hepacam2	0	0	0	1	0	1
mmu-miR-223-3p	101214	Tra2a	0	0	0	1	0	1
mmu-miR-223-3p	101240	Wdr91	0	0	0	1	0	1
mmu-miR-223-3p	101314	Brk1	0	0	0	1	0	1
mmu-miR-223-3p	101488	Slco2b1	0	0	0	1	0	1
mmu-miR-223-3p	101513	Mob2	0	0	0	1	0	1
mmu-miR-223-3p	101533	Klk9	0	0	0	1	0	1
mmu-miR-223-3p	101543	Wtip	0	0	0	1	0	1
mmu-miR-223-3p	101544	Zfp575	0	0	0	1	0	1
mmu-miR-223-3p	101565	Ccp110	0	0	0	1	0	1
mmu-miR-223-3p	101568	Vrk3	0	0	0	1	0	1
mmu-miR-223-3p	101592	Eftud1	0	0	0	1	0	1
mmu-miR-223-3p	101604	E430018J23Rik	0	0	0	1	0	1
mmu-miR-223-3p	101631	Pwwp2b	0	0	0	1	0	1
mmu-miR-223-3p	101739	Psip1	0	0	0	1	0	1
mmu-miR-223-3p	101744	Papl	0	0	0	1	0	1
mmu-miR-223-3p	101831	C230052I12Rik	1	0	0	0	0	1
mmu-miR-223-3p	101943	Sf3b3	0	0	0	1	0	1

mmu-miR-223-3p	101966	D8Ertd738e	0	0	0	1	0	1
mmu-miR-223-3p	101985	Usb1	0	0	0	1	0	1
mmu-miR-223-3p	101994	Champ1	0	0	0	1	0	1
mmu-miR-223-3p	102075	Plekhg4	0	0	0	1	0	1
mmu-miR-223-3p	102093	Phkb	0	0	0	1	0	1
mmu-miR-223-3p	102162	Taf5l	0	0	0	1	0	1
mmu-miR-223-3p	102209	Snapc2	0	0	0	1	0	1
mmu-miR-223-3p	102371	Myzap	0	0	0	1	0	1
mmu-miR-223-3p	102402	AA414992	1	0	0	0	0	1
mmu-miR-223-3p	102414	Clk3	0	0	0	1	0	1
mmu-miR-223-3p	102423	Hinfp	0	0	0	1	0	1
mmu-miR-223-3p	102566	Ano10	0	0	0	1	0	1
mmu-miR-223-3p	102570	Slc22a13	1	0	0	0	0	1
mmu-miR-223-3p	102626	Mapkapk3	0	0	0	1	0	1
mmu-miR-223-3p	102632	Acad11	0	0	0	1	0	1
mmu-miR-223-3p	102644	Oaf	0	0	0	1	0	1
mmu-miR-223-3p	102657	Cd276	0	0	0	1	0	1
mmu-miR-223-3p	102857	Slc6a8	0	0	0	1	0	1
mmu-miR-223-3p	102871	D330045A20Rik	0	0	0	1	0	1
mmu-miR-223-3p	102920	Cenpi	0	0	0	1	0	1
mmu-miR-223-3p	102954	Nudt10	0	0	0	1	0	1
mmu-miR-223-3p	102991	AU022751	0	0	0	1	0	1
mmu-miR-223-3p	103098	Slc6a15	0	0	0	1	0	1
mmu-miR-223-3p	103142	Rdh9	0	0	0	1	0	1
mmu-miR-223-3p	103199	Fig4	0	0	0	1	0	1
mmu-miR-223-3p	103213	Traf3ip2	0	0	0	1	0	1
mmu-miR-223-3p	103220	BC030307	0	0	0	1	0	1
mmu-miR-223-3p	103266	AI597468	0	0	0	1	0	1
mmu-miR-223-3p	103406	Zfr2	0	0	0	1	0	1
mmu-miR-223-3p	103425	Ncln	0	0	0	1	0	1
mmu-miR-223-3p	103511	Fam26e	0	0	0	1	0	1
mmu-miR-223-3p	103534	Mgat4b	0	0	0	1	0	1
mmu-miR-223-3p	103551	E130012A19Rik	1	0	0	0	0	1
mmu-miR-223-3p	103554	Psme4	0	0	0	1	0	1
mmu-miR-223-3p	103573	Xpo1	0	0	0	1	0	1
mmu-miR-223-3p	103583	Fbxw11	0	0	0	1	0	1
mmu-miR-223-3p	103655	Sec14l4	0	0	1	0	0	1
mmu-miR-223-3p	103694	Tmed4	0	0	0	1	0	1
mmu-miR-223-3p	103711	Pnpo	0	0	0	1	0	1
mmu-miR-223-3p	103733	Tubg1	0	0	0	1	0	1
mmu-miR-223-3p	103765	Tmem17	0	0	0	1	0	1
mmu-miR-223-3p	103768	Tubg2	0	0	0	1	0	1
mmu-miR-223-3p	103844	Inca1	0	0	0	1	0	1
mmu-miR-223-3p	103850	Nt5m	0	0	0	1	0	1
mmu-miR-223-3p	103968	Plin1	0	0	0	1	0	1
mmu-miR-223-3p	103978	Gpc5	0	0	0	1	0	1
mmu-miR-223-3p	104001	Rtn1	0	0	0	1	0	1
mmu-miR-223-3p	104002	Ctsq	0	0	0	1	0	1
mmu-miR-223-3p	104079	Nxph3	0	0	0	1	0	1
mmu-miR-223-3p	104111	Adcy3	0	0	0	1	0	1
mmu-miR-223-3p	104112	Acly	0	0	0	1	0	1
mmu-miR-223-3p	104252	Cdc42ep2	0	0	0	1	0	1
mmu-miR-223-3p	104263	Kdm3a	0	0	0	1	0	1
mmu-miR-223-3p	104303	Arl1	0	1	0	0	0	1
mmu-miR-223-3p	104362	Meig1	0	0	0	1	0	1

mmu-miR-223-3p	104382	Barhl2	0	0	0	1	0	1
mmu-miR-223-3p	104383	Rcor2	0	0	0	1	0	1
mmu-miR-223-3p	104416	Bap1	0	0	0	1	0	1
mmu-miR-223-3p	104418	Dgkz	0	0	0	1	0	1
mmu-miR-223-3p	104444	Rexo2	0	0	0	1	0	1
mmu-miR-223-3p	104445	Cdc42ep1	0	0	0	1	0	1
mmu-miR-223-3p	104457	0610010K14Rik	0	0	0	1	0	1
mmu-miR-223-3p	104479	Ccdc117	0	0	0	1	0	1
mmu-miR-223-3p	104570	Smek2	0	0	0	1	0	1
mmu-miR-223-3p	104625	Cnot6	0	0	0	1	0	1
mmu-miR-223-3p	104662	Tsr1	0	0	0	1	0	1
mmu-miR-223-3p	104718	Ttc7b	0	0	0	1	0	1
mmu-miR-223-3p	104725	Sptssa	0	0	0	1	0	1
mmu-miR-223-3p	104771	Jkamp	0	0	0	1	0	1
mmu-miR-223-3p	104776	Aldh6a1	0	0	0	1	0	1
mmu-miR-223-3p	104923	Adi1	0	0	0	1	0	1
mmu-miR-223-3p	105083	Pelo	0	0	0	1	0	1
mmu-miR-223-3p	105203	BC016423	0	0	0	1	0	1
mmu-miR-223-3p	105239	Rnf44	0	0	0	1	0	1
mmu-miR-223-3p	105246	Brd9	0	0	0	1	0	1
mmu-miR-223-3p	105278	Cdk20	0	0	0	1	0	1
mmu-miR-223-3p	105351	AW209491	0	0	0	1	0	1
mmu-miR-223-3p	105352	Dusp22	0	0	0	1	0	1
mmu-miR-223-3p	105387	Akr1c14	0	0	0	1	0	1
mmu-miR-223-3p	105439	Slain1	0	0	0	1	0	1
mmu-miR-223-3p	105450	Mmrn2	0	0	0	1	0	1
mmu-miR-223-3p	105501	Abhd4	0	0	0	1	0	1
mmu-miR-223-3p	105518	A630023A22Rik	0	0	0	1	0	1
mmu-miR-223-3p	105590	Zfp957	1	0	0	0	0	1
mmu-miR-223-3p	105638	Dph3	0	0	0	1	0	1
mmu-miR-223-3p	105663	Thtpa	0	0	0	1	0	1
mmu-miR-223-3p	105675	Ppif	0	0	0	1	0	1
mmu-miR-223-3p	105732	Fam83h	0	0	0	1	0	1
mmu-miR-223-3p	105785	Kdelr3	0	0	0	1	0	1
mmu-miR-223-3p	105835	Sgsm3	0	0	0	1	0	1
mmu-miR-223-3p	105844	Card10	0	0	0	1	0	1
mmu-miR-223-3p	105855	Nckap1l	0	1	0	0	0	1
mmu-miR-223-3p	105887	Ugt3a1	0	0	0	1	0	1
mmu-miR-223-3p	106025	Sharpin	0	0	0	1	0	1
mmu-miR-223-3p	106042	Prickle1	0	0	0	1	0	1
mmu-miR-223-3p	106200	Txndc11	0	1	0	0	0	1
mmu-miR-223-3p	106248	Qtrtd1	0	0	0	1	0	1
mmu-miR-223-3p	106338	Nsun3	0	1	0	0	0	1
mmu-miR-223-3p	106389	Eaf2	0	0	0	1	0	1
mmu-miR-223-3p	106489	Sft2d1	0	0	0	1	0	1
mmu-miR-223-3p	106529	Tecr	0	0	0	1	0	1
mmu-miR-223-3p	106564	Ppcs	0	0	0	1	0	1
mmu-miR-223-3p	106581	Itfg3	0	0	0	1	0	1
mmu-miR-223-3p	106582	Nrm	0	0	0	1	0	1
mmu-miR-223-3p	106628	Trip10	0	0	0	1	0	1
mmu-miR-223-3p	106633	Ift140	0	0	0	1	0	1
mmu-miR-223-3p	106648	Cyp4f15	0	0	0	1	0	1
mmu-miR-223-3p	106672	AI413582	0	0	0	1	0	1
mmu-miR-223-3p	106707	Rpusd1	0	0	0	1	0	1
mmu-miR-223-3p	106759	Ticam1	0	0	0	1	0	1

mmu-miR-223-3p	106821	Oard1	0	0	0	1	0	1
mmu-miR-223-3p	106878	Smim3	0	0	0	1	0	1
mmu-miR-223-3p	106952	Arap3	0	0	0	1	0	1
mmu-miR-223-3p	106957	Slc39a6	0	0	0	1	0	1
mmu-miR-223-3p	107022	Gramd3	0	0	0	1	0	1
mmu-miR-223-3p	107035	Fbxo38	0	0	0	1	0	1
mmu-miR-223-3p	107045	Lars	0	0	0	1	0	1
mmu-miR-223-3p	107141	Cyp2c50	0	0	0	1	0	1
mmu-miR-223-3p	107146	Glyat	0	0	0	1	0	1
mmu-miR-223-3p	107239	Carns1	0	0	0	1	0	1
mmu-miR-223-3p	107305	Vps37c	0	0	0	1	0	1
mmu-miR-223-3p	107373	Fam111a	0	0	0	1	0	1
mmu-miR-223-3p	107375	Slc25a45	0	0	0	1	0	1
mmu-miR-223-3p	107392	Brms1	0	0	0	1	0	1
mmu-miR-223-3p	107435	Hat1	0	0	0	1	0	1
mmu-miR-223-3p	107448	Unc5a	0	0	0	1	0	1
mmu-miR-223-3p	107476	Acaca	0	0	1	0	0	1
mmu-miR-223-3p	107477	Guca1b	0	0	0	1	0	1
mmu-miR-223-3p	107503	Atf5	0	0	0	1	0	1
mmu-miR-223-3p	107508	Eprs	0	0	0	1	0	1
mmu-miR-223-3p	107522	Ece2	0	0	0	1	0	1
mmu-miR-223-3p	107526	Gimap4	0	0	0	1	0	1
mmu-miR-223-3p	107527	Il1rl2	0	0	0	1	0	1
mmu-miR-223-3p	107569	Nt5c3	0	0	0	1	0	1
mmu-miR-223-3p	107607	Nod1	0	0	0	1	0	1
mmu-miR-223-3p	107652	Uap1	0	0	0	1	0	1
mmu-miR-223-3p	107702	Rnh1	0	0	0	1	0	1
mmu-miR-223-3p	107732	Mrpl10	0	0	1	0	0	1
mmu-miR-223-3p	107746	Rapgef1	0	0	0	1	0	1
mmu-miR-223-3p	107751	Prrxl1	0	0	0	1	0	1
mmu-miR-223-3p	107766	Haao	0	0	0	1	0	1
mmu-miR-223-3p	107770	Tm6sf2	0	0	0	1	0	1
mmu-miR-223-3p	107869	Cth	0	0	0	1	0	1
mmu-miR-223-3p	107939	Pom121	0	0	0	1	0	1
mmu-miR-223-3p	107971	Frs3	0	0	0	1	0	1
mmu-miR-223-3p	107975	Pacs1	0	0	0	1	0	1
mmu-miR-223-3p	107986	Ddb2	0	0	0	1	0	1
mmu-miR-223-3p	108000	Cenpf	0	0	0	1	0	1
mmu-miR-223-3p	108017	Fxyd4	0	0	0	1	0	1
mmu-miR-223-3p	108078	Olr1	0	0	0	1	0	1
mmu-miR-223-3p	108086	Rnf216	0	0	0	1	0	1
mmu-miR-223-3p	108096	Slco1a5	0	0	0	1	0	1
mmu-miR-223-3p	108098	Med21	0	0	1	0	0	1
mmu-miR-223-3p	108099	Prkag2	0	0	0	1	0	1
mmu-miR-223-3p	108101	Fermt3	0	0	0	1	0	1
mmu-miR-223-3p	108114	Slc22a7	0	0	0	1	0	1
mmu-miR-223-3p	108121	U2af1	0	0	0	1	0	1
mmu-miR-223-3p	108147	Atic	0	0	0	1	0	1
mmu-miR-223-3p	108148	Galnt2	0	0	0	1	0	1
mmu-miR-223-3p	108153	Adamts7	0	0	1	0	0	1
mmu-miR-223-3p	108160	Fam50a	0	0	0	1	0	1
mmu-miR-223-3p	108645	Mat2b	0	0	0	1	0	1
mmu-miR-223-3p	108660	Rnf187	0	0	0	1	0	1
mmu-miR-223-3p	108664	Atp6v1h	0	0	0	1	0	1
mmu-miR-223-3p	108679	Cops8	0	0	0	1	0	1

mmu-miR-223-3p	108707	Fam207a	0	0	0	1	0	1
mmu-miR-223-3p	108723	Card11	0	0	0	1	0	1
mmu-miR-223-3p	108760	Galnt16	0	0	0	1	0	1
mmu-miR-223-3p	108767	Pnrc1	0	0	1	0	0	1
mmu-miR-223-3p	108800	Ston2	0	0	0	1	0	1
mmu-miR-223-3p	108811	Ccdc122	0	0	0	1	0	1
mmu-miR-223-3p	108832	Tmem74b	0	0	0	1	0	1
mmu-miR-223-3p	108841	Rdh13	0	0	0	1	0	1
mmu-miR-223-3p	108853	Mtrf1l	0	0	0	1	0	1
mmu-miR-223-3p	108857	Ankhd1	0	0	0	1	0	1
mmu-miR-223-3p	108888	Atad3a	0	0	0	1	0	1
mmu-miR-223-3p	108902	B3gnt1	0	0	0	1	0	1
mmu-miR-223-3p	108911	Rcc2	0	0	0	1	0	1
mmu-miR-223-3p	108912	Cdca2	0	0	0	1	0	1
mmu-miR-223-3p	108958	Fam73b	0	0	0	1	0	1
mmu-miR-223-3p	108989	Tpr	0	0	0	1	0	1
mmu-miR-223-3p	109019	Nabp1	1	0	0	0	0	1
mmu-miR-223-3p	109052	Krt75	0	1	0	0	0	1
mmu-miR-223-3p	109077	Ints5	1	0	0	0	0	1
mmu-miR-223-3p	109079	Sephs1	0	0	0	1	0	1
mmu-miR-223-3p	109082	Fbxw17	0	1	0	0	0	1
mmu-miR-223-3p	109129	Mmadhc	0	0	0	1	0	1
mmu-miR-223-3p	109135	Plekha5	0	0	0	1	0	1
mmu-miR-223-3p	109212	Fam64a	0	0	0	1	0	1
mmu-miR-223-3p	109218	Tmem139	0	0	0	1	0	1
mmu-miR-223-3p	109222	Rarres1	0	0	0	1	0	1
mmu-miR-223-3p	109232	Sccpdh	0	0	0	1	0	1
mmu-miR-223-3p	109254	Adtrp	0	0	0	1	0	1
mmu-miR-223-3p	109263	Rlf	0	0	0	1	0	1
mmu-miR-223-3p	109270	Prr5	0	0	0	1	0	1
mmu-miR-223-3p	109272	Mybpc1	0	0	0	1	0	1
mmu-miR-223-3p	109284	R3hdm4	0	0	0	1	0	1
mmu-miR-223-3p	109305	Orai1	0	0	0	1	0	1
mmu-miR-223-3p	109314	Prr9	0	0	0	1	0	1
mmu-miR-223-3p	109332	Cdcp1	0	0	0	1	0	1
mmu-miR-223-3p	109346	Ankrd39	0	0	0	1	0	1
mmu-miR-223-3p	109349	Fam163b	0	0	0	1	0	1
mmu-miR-223-3p	109359	Fam175b	0	1	0	0	0	1
mmu-miR-223-3p	109593	Lmo3	0	0	0	1	0	1
mmu-miR-223-3p	109594	Lmo1	0	0	0	1	0	1
mmu-miR-223-3p	109620	Dsp	0	0	0	1	0	1
mmu-miR-223-3p	109674	Ampd2	0	0	0	1	0	1
mmu-miR-223-3p	109711	Actn1	1	0	0	0	0	1
mmu-miR-223-3p	109731	Maob	0	0	0	1	0	1
mmu-miR-223-3p	109815	Vimp	0	0	0	1	0	1
mmu-miR-223-3p	109820	Pgc	1	0	0	0	0	1
mmu-miR-223-3p	109889	Mzf1	1	0	0	0	0	1
mmu-miR-223-3p	109901	Cela1	0	0	0	1	0	1
mmu-miR-223-3p	109929	Zbtb25	0	0	0	1	0	1
mmu-miR-223-3p	109934	Abr	0	0	0	1	0	1
mmu-miR-223-3p	109978	Art4	1	0	0	0	0	1
mmu-miR-223-3p	109979	Art3	0	0	0	1	0	1
mmu-miR-223-3p	110075	Bmp3	0	0	0	1	0	1
mmu-miR-223-3p	110082	Dnah5	0	0	0	1	0	1
mmu-miR-223-3p	110094	Phka2	0	0	0	1	0	1

mmu-miR-223-3p	110109	Nop2	0	0	0	1	0	1
mmu-miR-223-3p	110115	Cyp11b1	0	0	0	1	0	1
mmu-miR-223-3p	110147	Ehmt2	0	0	0	1	0	1
mmu-miR-223-3p	110168	Gpr18	1	0	0	0	0	1
mmu-miR-223-3p	110172	Slc35b1	0	1	0	0	0	1
mmu-miR-223-3p	110175	Ggct	0	0	0	1	0	1
mmu-miR-223-3p	110187	Scgb2b26	0	0	0	1	0	1
mmu-miR-223-3p	110208	Pgd	0	0	0	1	0	1
mmu-miR-223-3p	110253	Triobp	0	0	0	1	0	1
mmu-miR-223-3p	110265	Msra	0	0	0	1	0	1
mmu-miR-223-3p	110279	Bcr	0	0	0	1	0	1
mmu-miR-223-3p	110312	Pmch	0	0	0	1	0	1
mmu-miR-223-3p	110332	Pp2d1	0	0	0	1	0	1
mmu-miR-223-3p	110351	Rap1gap	0	0	0	1	0	1
mmu-miR-223-3p	110391	Qdpr	0	0	0	1	0	1
mmu-miR-223-3p	110446	Acat1	0	0	0	1	0	1
mmu-miR-223-3p	110637	Grik4	0	1	0	0	0	1
mmu-miR-223-3p	110749	Chaf1b	0	0	0	1	0	1
mmu-miR-223-3p	110789	Gpr98	0	1	0	0	0	1
mmu-miR-223-3p	110816	Pwp2	0	0	0	1	0	1
mmu-miR-223-3p	110829	Lims1	0	0	0	1	0	1
mmu-miR-223-3p	110834	Chrna3	1	0	0	0	0	1
mmu-miR-223-3p	110855	Pde6c	0	0	0	1	0	1
mmu-miR-223-3p	110862	Kcnq3	0	0	0	1	0	1
mmu-miR-223-3p	110891	Slc8a2	0	0	0	1	0	1
mmu-miR-223-3p	110895	Slc9a4	0	0	0	1	0	1
mmu-miR-223-3p	110902	Chrna2	0	0	0	1	0	1
mmu-miR-223-3p	110957	D1Pas1	0	0	0	1	0	1
mmu-miR-223-3p	110960	Tars	0	0	0	1	0	1
mmu-miR-223-3p	110962	Mbd6	0	0	0	1	0	1
mmu-miR-223-3p	112418	1700102P08Rik	0	0	0	1	0	1
mmu-miR-223-3p	113853	Vmn1r53	0	0	0	1	0	1
mmu-miR-223-3p	113868	Acaa1a	0	0	0	1	0	1
mmu-miR-223-3p	114229	Kiss1r	0	0	0	1	0	1
mmu-miR-223-3p	114304	Slc28a3	0	0	0	1	0	1
mmu-miR-223-3p	114332	Lyve1	0	0	1	0	0	1
mmu-miR-223-3p	114570	Crip3	0	0	0	1	0	1
mmu-miR-223-3p	114585	D17H6S53E	0	0	0	1	0	1
mmu-miR-223-3p	114606	Tle6	1	0	0	0	0	1
mmu-miR-223-3p	114644	Slc13a3	0	0	0	1	0	1
mmu-miR-223-3p	114662	Prss29	0	0	0	1	0	1
mmu-miR-223-3p	114663	Impa2	0	0	0	1	0	1
mmu-miR-223-3p	114664	Hsd17b11	0	0	0	1	0	1
mmu-miR-223-3p	114666	Krtap5-5	0	0	0	1	0	1
mmu-miR-223-3p	114712	Ferd3l	0	0	0	1	0	1
mmu-miR-223-3p	114713	Rasa2	0	0	0	1	0	1
mmu-miR-223-3p	114716	Spred2	0	1	0	0	0	1
mmu-miR-223-3p	114774	Pawr	0	0	0	1	0	1
mmu-miR-223-3p	114871	Psg28	0	0	0	1	0	1
mmu-miR-223-3p	114872	Psg29	0	0	0	1	0	1
mmu-miR-223-3p	116701	Fgfrl1	1	0	0	0	0	1
mmu-miR-223-3p	116748	Lsm10	0	0	0	1	0	1
mmu-miR-223-3p	116810	Foxn4	0	0	0	1	0	1
mmu-miR-223-3p	116838	Rims2	0	0	0	1	0	1
mmu-miR-223-3p	116852	Akr1c20	0	0	0	1	0	1

mmu-miR-223-3p	116870	Mta1	0	0	0	1	0	1
mmu-miR-223-3p	116872	Serpinb7	0	0	0	1	0	1
mmu-miR-223-3p	116904	Alpk3	0	0	0	1	0	1
mmu-miR-223-3p	116972	Fam57a	0	1	0	0	0	1
mmu-miR-223-3p	117109	Pop5	0	0	0	1	0	1
mmu-miR-223-3p	117148	Necab2	0	0	0	1	0	1
mmu-miR-223-3p	117150	Pip4k2c	1	0	0	0	0	1
mmu-miR-223-3p	117160	Ttyh2	0	0	0	1	0	1
mmu-miR-223-3p	117167	Steap4	0	0	0	1	0	1
mmu-miR-223-3p	117171	1110038F14Rik	0	0	0	1	0	1
mmu-miR-223-3p	117172	2310034C09Rik	0	0	0	1	0	1
mmu-miR-223-3p	117586	A1bg	0	0	0	1	0	1
mmu-miR-223-3p	117589	Asb7	0	0	0	1	0	1
mmu-miR-223-3p	117599	Helb	0	1	0	0	0	1
mmu-miR-223-3p	118445	Klf16	0	0	0	1	0	1
mmu-miR-223-3p	118453	Mmp28	0	0	0	1	0	1
mmu-miR-223-3p	118454	Gjc2	0	0	0	1	0	1
mmu-miR-223-3p	121021	Cspg4	0	0	0	1	0	1
mmu-miR-223-3p	121022	Mrps6	0	0	0	1	0	1
mmu-miR-223-3p	140474	Muc4	0	0	0	1	0	1
mmu-miR-223-3p	140476	Strc	0	1	0	0	0	1
mmu-miR-223-3p	140477	Dmbx1	0	0	0	1	0	1
mmu-miR-223-3p	140483	Hnmt	0	0	0	1	0	1
mmu-miR-223-3p	140492	Kcnn2	1	0	0	0	0	1
mmu-miR-223-3p	140497	AF251705	0	0	0	1	0	1
mmu-miR-223-3p	140498	Rxfp2	0	0	0	1	0	1
mmu-miR-223-3p	140499	Ube2j2	0	0	0	1	0	1
mmu-miR-223-3p	140557	Smc1b	0	0	0	1	0	1
mmu-miR-223-3p	140559	Igsf8	0	0	0	1	0	1
mmu-miR-223-3p	140577	Ankrd6	0	0	0	1	0	1
mmu-miR-223-3p	140709	Col26a1	0	0	0	1	0	1
mmu-miR-223-3p	140742	Sesn1	0	0	0	1	0	1
mmu-miR-223-3p	140795	P2ry14	0	0	0	1	0	1
mmu-miR-223-3p	140806	Il25	0	0	0	1	0	1
mmu-miR-223-3p	140859	Nek8	0	0	0	1	0	1
mmu-miR-223-3p	140904	Caln1	0	0	0	1	0	1
mmu-miR-223-3p	142688	Asb13	0	0	0	1	0	1
mmu-miR-223-3p	170472	Recql5	0	0	0	1	0	1
mmu-miR-223-3p	170571	Cntnap4	0	0	0	1	0	1
mmu-miR-223-3p	170574	Sp7	0	0	0	1	0	1
mmu-miR-223-3p	170677	Cdhr1	1	0	0	0	0	1
mmu-miR-223-3p	170706	Tmem37	0	0	0	1	0	1
mmu-miR-223-3p	170716	Cyp4f13	0	1	0	0	0	1
mmu-miR-223-3p	170718	Idh3b	0	0	0	1	0	1
mmu-miR-223-3p	170721	Papln	0	0	0	1	0	1
mmu-miR-223-3p	170729	Scrt1	0	0	0	1	0	1
mmu-miR-223-3p	170732	Trhr2	0	0	0	1	0	1
mmu-miR-223-3p	170734	Zscan5b	0	0	0	1	0	1
mmu-miR-223-3p	170735	Arr3	0	1	0	0	0	1
mmu-miR-223-3p	170738	Kcnh7	0	1	0	0	0	1
mmu-miR-223-3p	170743	Tlr7	0	0	0	1	0	1
mmu-miR-223-3p	170748	Smco4	0	0	0	1	0	1
mmu-miR-223-3p	170750	Xpnpep1	0	0	1	0	0	1
mmu-miR-223-3p	170752	Bco2	0	0	0	1	0	1
mmu-miR-223-3p	170758	Rac3	0	0	0	1	0	1

mmu-miR-223-3p	170759	Atp13a1	0	0	0	1	0	1
mmu-miR-223-3p	170760	Acbd3	0	0	0	1	0	1
mmu-miR-223-3p	170761	Pdzd3	0	0	0	1	0	1
mmu-miR-223-3p	170762	Nup155	0	0	0	1	0	1
mmu-miR-223-3p	170763	Zfp87	1	0	0	0	0	1
mmu-miR-223-3p	170768	Pfkfb3	0	0	0	1	0	1
mmu-miR-223-3p	170770	Bbc3	0	1	0	0	0	1
mmu-miR-223-3p	170780	Cd209e	0	0	0	1	0	1
mmu-miR-223-3p	170786	Cd209a	0	0	0	1	0	1
mmu-miR-223-3p	170788	Crb1	0	0	0	1	0	1
mmu-miR-223-3p	170791	Rbm39	0	0	0	1	0	1
mmu-miR-223-3p	170799	Rtkn2	0	0	0	1	0	1
mmu-miR-223-3p	170813	Ms4a3	0	0	0	1	0	1
mmu-miR-223-3p	170823	Glmn	0	1	0	0	0	1
mmu-miR-223-3p	170833	Hook2	1	0	0	0	0	1
mmu-miR-223-3p	170930	Sumo2	0	0	0	1	0	1
mmu-miR-223-3p	170939	Krtap19-9b	0	0	0	1	0	1
mmu-miR-223-3p	170947	Myoz3	0	0	0	1	0	1
mmu-miR-223-3p	170952	Prima1	0	0	1	0	0	1
mmu-miR-223-3p	171168	Acer1	0	0	0	1	0	1
mmu-miR-223-3p	171205	Vmn1r8	0	0	0	1	0	1
mmu-miR-223-3p	171207	Arhgap4	0	0	0	1	0	1
mmu-miR-223-3p	171227	Vmn1r232	0	0	0	1	0	1
mmu-miR-223-3p	171235	Vmn1r236	0	0	0	1	0	1
mmu-miR-223-3p	171264	Vmn1r66	0	0	0	1	0	1
mmu-miR-223-3p	171282	Acot4	0	0	0	1	0	1
mmu-miR-223-3p	171283	Havcr1	0	0	0	1	0	1
mmu-miR-223-3p	171284	Timd2	0	0	0	1	0	1
mmu-miR-223-3p	171286	Slc12a8	0	0	0	1	0	1
mmu-miR-223-3p	171382	Trpm8	0	0	0	1	0	1
mmu-miR-223-3p	171429	Slc26a6	0	0	0	1	0	1
mmu-miR-223-3p	171508	Creld1	0	0	0	1	0	1
mmu-miR-223-3p	171530	Ucn2	0	0	0	1	0	1
mmu-miR-223-3p	192113	Atp12a	0	0	0	1	0	1
mmu-miR-223-3p	192120	Bspry	1	0	0	0	0	1
mmu-miR-223-3p	192136	5033411D12Rik	0	0	0	1	0	1
mmu-miR-223-3p	192159	Prpf8	0	1	0	0	0	1
mmu-miR-223-3p	192170	Eif4a3	0	0	0	1	0	1
mmu-miR-223-3p	192173	Fam195b	0	0	0	1	0	1
mmu-miR-223-3p	192176	Flna	0	1	0	0	0	1
mmu-miR-223-3p	192194	Btnl10	0	0	1	0	0	1
mmu-miR-223-3p	192201	Wfdc15b	1	0	0	0	0	1
mmu-miR-223-3p	192232	Hps4	0	0	0	1	0	1
mmu-miR-223-3p	192285	Phf21a	0	0	0	1	0	1
mmu-miR-223-3p	192289	Tmlhe	1	0	0	0	0	1
mmu-miR-223-3p	192652	Wdr81	0	0	0	1	0	1
mmu-miR-223-3p	192656	Ripk2	0	0	0	1	0	1
mmu-miR-223-3p	192658	Rfpl4	0	0	0	1	0	1
mmu-miR-223-3p	192897	Itgb4	0	0	0	1	0	1
mmu-miR-223-3p	192976	Fam211a	0	0	0	1	0	1
mmu-miR-223-3p	193813	Mcfd2	0	0	0	1	0	1
mmu-miR-223-3p	193838	Eme2	0	1	0	0	0	1
mmu-miR-223-3p	194225	Gm13103	0	0	0	1	0	1
mmu-miR-223-3p	194231	Cnksr1	0	0	0	1	0	1
mmu-miR-223-3p	194588	Gm4745	0	0	1	0	0	1

mmu-miR-223-3p	194655	Klf11	0	0	0	1	0	1
mmu-miR-223-3p	194744	Slc25a43	0	0	0	1	0	1
mmu-miR-223-3p	194854	Gm9	0	0	0	1	0	1
mmu-miR-223-3p	195236	Pom121l2	0	0	0	1	0	1
mmu-miR-223-3p	195646	Hs3st2	1	0	0	0	0	1
mmu-miR-223-3p	195727	Nhs	0	0	0	1	0	1
mmu-miR-223-3p	195733	Grhl1	0	0	0	1	0	1
mmu-miR-223-3p	207209	Ccdc154	0	0	0	1	0	1
mmu-miR-223-3p	207213	Tdpoz1	1	0	0	0	0	1
mmu-miR-223-3p	207215	Fbxo40	0	1	0	0	0	1
mmu-miR-223-3p	207304	Hectd1	0	0	0	1	0	1
mmu-miR-223-3p	207352	Sec23ip	0	0	0	1	0	1
mmu-miR-223-3p	207521	Dtx4	0	0	0	1	0	1
mmu-miR-223-3p	207565	Camkk2	0	0	0	1	0	1
mmu-miR-223-3p	207686	A330021E22Rik	0	0	0	1	0	1
mmu-miR-223-3p	207728	Pde2a	0	0	0	1	0	1
mmu-miR-223-3p	207740	Ubald1	0	0	0	1	0	1
mmu-miR-223-3p	207742	Rnf43	0	0	0	1	0	1
mmu-miR-223-3p	207792	BC034090	0	0	0	1	0	1
mmu-miR-223-3p	207911	Mchr1	0	0	0	1	0	1
mmu-miR-223-3p	207920	Esrp1	0	0	0	1	0	1
mmu-miR-223-3p	207952	Klhl25	0	0	0	1	0	1
mmu-miR-223-3p	208076	Pknox2	0	0	0	1	0	1
mmu-miR-223-3p	208146	Yeats2	0	0	0	1	0	1
mmu-miR-223-3p	208177	Phldb2	0	0	0	1	0	1
mmu-miR-223-3p	208194	Exog	0	0	0	1	0	1
mmu-miR-223-3p	208211	Alg1	0	0	0	1	0	1
mmu-miR-223-3p	208213	Tmem132c	1	0	0	0	0	1
mmu-miR-223-3p	208266	Dot1l	0	1	0	0	0	1
mmu-miR-223-3p	208426	Iqcj	0	0	0	1	0	1
mmu-miR-223-3p	208439	Klhl29	0	0	0	1	0	1
mmu-miR-223-3p	208449	Sgms1	0	0	0	1	0	1
mmu-miR-223-3p	208595	Gm9897	0	0	0	1	0	1
mmu-miR-223-3p	208613	Tmem212	0	0	0	1	0	1
mmu-miR-223-3p	208628	Kntc1	0	0	0	1	0	1
mmu-miR-223-3p	208634	Tspan10	0	0	0	1	0	1
mmu-miR-223-3p	208638	Slc25a38	0	0	0	1	0	1
mmu-miR-223-3p	208643	Eif4g1	0	0	0	1	0	1
mmu-miR-223-3p	208665	Akr1d1	0	0	0	1	0	1
mmu-miR-223-3p	208666	Diras1	0	0	0	1	0	1
mmu-miR-223-3p	208718	Dis3l2	0	0	0	1	0	1
mmu-miR-223-3p	208795	Tmem63a	0	0	0	1	0	1
mmu-miR-223-3p	208884	Zdhhc9	0	0	1	0	0	1
mmu-miR-223-3p	208898	Unc13c	0	0	0	1	0	1
mmu-miR-223-3p	208936	Adamts18	0	1	0	0	0	1
mmu-miR-223-3p	208967	Thnsl1	0	0	0	1	0	1
mmu-miR-223-3p	209003	Rbmx2	0	0	0	1	0	1
mmu-miR-223-3p	209005	Gm595	0	0	0	1	0	1
mmu-miR-223-3p	209011	Sirt7	0	0	0	1	0	1
mmu-miR-223-3p	209018	Vps8	0	0	0	1	0	1
mmu-miR-223-3p	209027	Pycr1	0	0	0	1	0	1
mmu-miR-223-3p	209225	Zfp710	0	0	0	1	0	1
mmu-miR-223-3p	209268	Igsf1	0	0	0	1	0	1
mmu-miR-223-3p	209318	Gps1	0	0	0	1	0	1
mmu-miR-223-3p	209351	Wfdc6a	0	0	0	1	0	1

mmu-miR-223-3p	209357	Gtf2h3	0	0	0	1	0	1
mmu-miR-223-3p	209456	Trp53bp2	0	0	0	1	0	1
mmu-miR-223-3p	209488	Hsh2d	0	0	0	1	0	1
mmu-miR-223-3p	209586	Nudcd3	0	0	0	1	0	1
mmu-miR-223-3p	209590	Il23r	0	0	0	1	0	1
mmu-miR-223-3p	209601	4922501L14Rik	0	0	0	1	0	1
mmu-miR-223-3p	209837	Slc38a5	0	0	0	1	0	1
mmu-miR-223-3p	210004	B3gntl1	0	0	0	1	0	1
mmu-miR-223-3p	210009	Mtrr	0	0	0	1	0	1
mmu-miR-223-3p	210029	Metrnl	0	0	0	1	0	1
mmu-miR-223-3p	210045	Nlrp4b	0	0	0	1	0	1
mmu-miR-223-3p	210104	Zfp658	0	0	0	1	0	1
mmu-miR-223-3p	210106	Papd7	0	0	0	1	0	1
mmu-miR-223-3p	210148	Slc30a6	0	0	0	1	0	1
mmu-miR-223-3p	210155	Gm4763	0	0	0	1	0	1
mmu-miR-223-3p	210297	Lrch2	0	0	0	1	0	1
mmu-miR-223-3p	210356	Nckap5	0	0	0	1	0	1
mmu-miR-223-3p	210463	Slc22a22	0	0	0	1	0	1
mmu-miR-223-3p	210510	Tdrd6	0	0	0	1	0	1
mmu-miR-223-3p	210544	Tbc1d31	0	0	0	1	0	1
mmu-miR-223-3p	210622	Pamr1	0	0	0	1	0	1
mmu-miR-223-3p	210673	Prrt3	0	0	0	1	0	1
mmu-miR-223-3p	210710	Gab3	0	1	0	0	0	1
mmu-miR-223-3p	210762	Ppp1r36	0	0	0	1	0	1
mmu-miR-223-3p	210789	Tbc1d4	0	0	0	1	0	1
mmu-miR-223-3p	210925	Ints9	0	0	0	1	0	1
mmu-miR-223-3p	210962	Gm597	0	0	0	1	0	1
mmu-miR-223-3p	210982	Gltscr1l	0	0	0	1	0	1
mmu-miR-223-3p	211007	Trim41	0	0	0	1	0	1
mmu-miR-223-3p	211135	D130040H23Rik	1	0	0	0	0	1
mmu-miR-223-3p	211151	Churc1	0	0	0	1	0	1
mmu-miR-223-3p	211286	Cln5	1	0	0	0	0	1
mmu-miR-223-3p	211305	Fbxw13	1	0	0	0	0	1
mmu-miR-223-3p	211329	Ncoa7	0	0	0	1	0	1
mmu-miR-223-3p	211378	6720489N17Rik	0	0	0	1	0	1
mmu-miR-223-3p	211389	Suox	0	0	0	1	0	1
mmu-miR-223-3p	211499	Tmem87a	0	0	0	1	0	1
mmu-miR-223-3p	211535	Ccdc114	0	0	0	1	0	1
mmu-miR-223-3p	211548	Nomo1	0	0	0	1	0	1
mmu-miR-223-3p	211550	Tifa	0	0	0	1	0	1
mmu-miR-223-3p	211578	Mrgprd	0	0	0	1	0	1
mmu-miR-223-3p	211623	Plac9a	0	1	0	0	0	1
mmu-miR-223-3p	211651	Fancd2	0	0	0	1	0	1
mmu-miR-223-3p	211660	Cspp1	0	0	0	1	0	1
mmu-miR-223-3p	211673	Arfgef1	0	0	0	1	0	1
mmu-miR-223-3p	211948	Pde12	0	1	0	0	0	1
mmu-miR-223-3p	211949	Spsb4	0	0	0	1	0	1
mmu-miR-223-3p	211978	Zfyve26	1	0	0	0	0	1
mmu-miR-223-3p	212085	Trim52	1	0	0	0	0	1
mmu-miR-223-3p	212090	Tmem60	0	0	0	1	0	1
mmu-miR-223-3p	212111	Inpp5a	0	0	0	1	0	1
mmu-miR-223-3p	212114	Nhlrc3	0	0	0	1	0	1
mmu-miR-223-3p	212123	Dcaf15	0	0	0	1	0	1
mmu-miR-223-3p	212163	8030462N17Rik	0	0	0	1	0	1
mmu-miR-223-3p	212285	Arap2	1	0	0	0	0	1

mmu-miR-223-3p	212377	Mms22l	0	1	0	0	0	1
mmu-miR-223-3p	212398	Frat2	0	0	0	1	0	1
mmu-miR-223-3p	212427	A730008H23Rik	0	0	0	1	0	1
mmu-miR-223-3p	212442	Lactb2	0	0	0	1	0	1
mmu-miR-223-3p	212503	Paox	0	0	0	1	0	1
mmu-miR-223-3p	212508	Mtg1	0	0	0	1	0	1
mmu-miR-223-3p	212528	Trmt1	0	0	0	1	0	1
mmu-miR-223-3p	212547	BC027231	0	0	1	0	0	1
mmu-miR-223-3p	212555	Pqlc2	0	0	0	1	0	1
mmu-miR-223-3p	212627	Prpsap2	0	0	0	1	0	1
mmu-miR-223-3p	212679	Mars2	0	0	0	1	0	1
mmu-miR-223-3p	212706	N4bp3	0	0	0	1	0	1
mmu-miR-223-3p	212733	Ccdc64b	0	0	0	1	0	1
mmu-miR-223-3p	212772	Arl14ep	0	0	0	1	0	1
mmu-miR-223-3p	212892	Rsph4a	0	0	0	1	0	1
mmu-miR-223-3p	212898	Dse	0	0	0	1	0	1
mmu-miR-223-3p	212919	Kctd7	0	0	0	1	0	1
mmu-miR-223-3p	212933	Pm20d1	0	0	0	1	0	1
mmu-miR-223-3p	212996	Wbscr17	0	0	0	1	0	1
mmu-miR-223-3p	212999	Tnpo2	0	0	0	1	0	1
mmu-miR-223-3p	213012	Abhd10	0	0	0	1	0	1
mmu-miR-223-3p	213054	Gabpb2	0	0	0	1	0	1
mmu-miR-223-3p	213081	Wdr19	0	0	0	1	0	1
mmu-miR-223-3p	213233	Tapbpl	0	0	0	1	0	1
mmu-miR-223-3p	213311	Fbxl21	0	0	0	1	0	1
mmu-miR-223-3p	213326	Scyl2	0	0	0	1	0	1
mmu-miR-223-3p	213393	8430408G22Rik	0	0	0	1	0	1
mmu-miR-223-3p	213436	Zcchc5	0	0	0	1	0	1
mmu-miR-223-3p	213439	Gpr174	0	0	0	1	0	1
mmu-miR-223-3p	213484	Nudt18	0	0	0	1	0	1
mmu-miR-223-3p	213491	Szrd1	0	0	0	1	0	1
mmu-miR-223-3p	213527	Pth2r	0	0	0	1	0	1
mmu-miR-223-3p	213539	Bag2	0	0	0	1	0	1
mmu-miR-223-3p	213541	Ythdf2	0	0	0	1	0	1
mmu-miR-223-3p	213649	Arhgef19	0	0	0	1	0	1
mmu-miR-223-3p	213673	9530068E07Rik	0	0	0	1	0	1
mmu-miR-223-3p	213696	Duoxa1	0	0	0	1	0	1
mmu-miR-223-3p	213760	Prepl	0	0	0	1	0	1
mmu-miR-223-3p	213765	Nutm1	0	0	0	1	0	1
mmu-miR-223-3p	213783	Plekhg1	0	0	0	1	0	1
mmu-miR-223-3p	213895	Bms1	0	0	0	1	0	1
mmu-miR-223-3p	213948	Atg9b	0	0	0	1	0	1
mmu-miR-223-3p	213956	Fam83f	0	0	0	1	0	1
mmu-miR-223-3p	213980	Fbxw10	1	0	0	0	0	1
mmu-miR-223-3p	213993	A630007B06Rik	0	1	0	0	0	1
mmu-miR-223-3p	214111	Slc24a1	0	0	0	1	0	1
mmu-miR-223-3p	214137	Arhgap29	0	1	0	0	0	1
mmu-miR-223-3p	214230	Pak6	0	0	0	1	0	1
mmu-miR-223-3p	214254	Nudt15	0	0	0	1	0	1
mmu-miR-223-3p	214292	Syna	0	0	0	1	0	1
mmu-miR-223-3p	214345	Lrrc1	0	0	0	1	0	1
mmu-miR-223-3p	214359	Tmem51	0	0	0	1	0	1
mmu-miR-223-3p	214384	Myocd	0	0	0	1	0	1
mmu-miR-223-3p	214394	A530010F05Rik	0	0	0	1	0	1
mmu-miR-223-3p	214403	Gm4788	0	0	0	1	0	1

mmu-miR-223-3p	214424	Parp16	0	0	0	1	0	1
mmu-miR-223-3p	214425	Cilp	0	0	0	1	0	1
mmu-miR-223-3p	214444	Cdk5rap2	0	1	0	0	0	1
mmu-miR-223-3p	214498	Cdc73	0	1	0	0	0	1
mmu-miR-223-3p	214579	Aldh5a1	0	0	0	1	0	1
mmu-miR-223-3p	214585	Spg11	1	0	0	0	0	1
mmu-miR-223-3p	214597	Sidt2	0	0	0	1	0	1
mmu-miR-223-3p	214642	Cped1	0	0	0	1	0	1
mmu-miR-223-3p	214685	Chadl	0	0	0	1	0	1
mmu-miR-223-3p	214742	Rcor3	0	0	0	1	0	1
mmu-miR-223-3p	214779	Zfp879	0	0	0	1	0	1
mmu-miR-223-3p	214791	Sertad4	0	0	0	1	0	1
mmu-miR-223-3p	214804	Syde2	0	0	0	1	0	1
mmu-miR-223-3p	214931	Fbxl16	0	0	0	1	0	1
mmu-miR-223-3p	214932	Cecr5	0	0	0	1	0	1
mmu-miR-223-3p	214951	Rhbdl1	0	0	1	0	0	1
mmu-miR-223-3p	214987	Chtf8	0	0	0	1	0	1
mmu-miR-223-3p	215029	Prl3d3	0	0	0	1	0	1
mmu-miR-223-3p	215031	Vgll2	0	0	0	1	0	1
mmu-miR-223-3p	215051	Bud13	0	0	0	1	0	1
mmu-miR-223-3p	215090	Maneal	1	0	0	0	0	1
mmu-miR-223-3p	215095	Astl	0	0	0	1	0	1
mmu-miR-223-3p	215160	Rhbdd2	0	0	0	1	0	1
mmu-miR-223-3p	215201	Trmt2b	0	0	0	1	0	1
mmu-miR-223-3p	215351	Senp6	0	1	0	0	0	1
mmu-miR-223-3p	215384	Fcgbp	0	0	0	1	0	1
mmu-miR-223-3p	215445	Rab11fip3	0	0	0	1	0	1
mmu-miR-223-3p	215467	Gm4791	0	0	0	1	0	1
mmu-miR-223-3p	215494	Pomgnt2	0	0	0	1	0	1
mmu-miR-223-3p	215512	Fam117a	0	0	0	1	0	1
mmu-miR-223-3p	215641	Mageb18	0	0	0	1	0	1
mmu-miR-223-3p	215705	Arrdc1	0	1	0	0	0	1
mmu-miR-223-3p	215707	Ccdc92	0	0	0	1	0	1
mmu-miR-223-3p	215751	Ginm1	0	0	0	1	0	1
mmu-miR-223-3p	215866	LOC215866	0	0	0	1	0	1
mmu-miR-223-3p	215900	Fam26f	0	0	0	1	0	1
mmu-miR-223-3p	215929	AI317395	0	0	0	1	0	1
mmu-miR-223-3p	215951	Lace1	0	0	0	1	0	1
mmu-miR-223-3p	216019	Hkdc1	0	0	0	1	0	1
mmu-miR-223-3p	216033	Ctnna3	0	0	0	1	0	1
mmu-miR-223-3p	216144	Vmn2r81	0	0	0	1	0	1
mmu-miR-223-3p	216152	BC005764	0	0	0	1	0	1
mmu-miR-223-3p	216154	Med16	0	0	0	1	0	1
mmu-miR-223-3p	216157	Tmem259	0	0	0	1	0	1
mmu-miR-223-3p	216161	Sbno2	0	0	0	1	0	1
mmu-miR-223-3p	216166	Plk5	0	0	0	1	0	1
mmu-miR-223-3p	216177	AU041133	0	0	0	1	0	1
mmu-miR-223-3p	216197	Ckap4	0	0	0	1	0	1
mmu-miR-223-3p	216198	Tcp11l2	0	0	0	1	0	1
mmu-miR-223-3p	216344	Rab21	0	0	0	1	0	1
mmu-miR-223-3p	216350	Tspan8	0	0	0	1	0	1
mmu-miR-223-3p	216395	Tmem5	0	1	0	0	0	1
mmu-miR-223-3p	216438	March9	0	0	0	1	0	1
mmu-miR-223-3p	216443	Mars	0	0	0	1	0	1
mmu-miR-223-3p	216445	Arhgap9	0	0	0	1	0	1

mmu-miR-223-3p	216453	Rdh19	0	0	0	1	0	1
mmu-miR-223-3p	216454	BC089597	0	0	0	1	0	1
mmu-miR-223-3p	216516	Ccdc157	0	0	0	1	0	1
mmu-miR-223-3p	216527	Ccm2	0	0	0	1	0	1
mmu-miR-223-3p	216558	Ugp2	1	0	0	0	0	1
mmu-miR-223-3p	216560	Wdpcp	0	0	0	1	0	1
mmu-miR-223-3p	216565	Ehbp1	0	0	0	1	0	1
mmu-miR-223-3p	216643	Gabrp	0	0	0	1	0	1
mmu-miR-223-3p	216724	Rufy1	0	0	0	1	0	1
mmu-miR-223-3p	216781	Trim58	0	0	0	1	0	1
mmu-miR-223-3p	216792	Iba57	0	0	0	1	0	1
mmu-miR-223-3p	216820	Dhrs7b	0	0	0	1	0	1
mmu-miR-223-3p	216821	Tmem11	0	0	0	1	0	1
mmu-miR-223-3p	216825	Usp22	0	0	0	1	0	1
mmu-miR-223-3p	216835	Usp43	0	0	0	1	0	1
mmu-miR-223-3p	216846	Cntrob	0	0	0	1	0	1
mmu-miR-223-3p	216850	Kdm6b	0	0	0	1	0	1
mmu-miR-223-3p	216867	Slc16a11	0	0	0	1	0	1
mmu-miR-223-3p	216971	Fam222b	0	0	0	1	0	1
mmu-miR-223-3p	216991	Adap2	0	0	0	1	0	1
mmu-miR-223-3p	217011	Nle1	0	0	0	1	0	1
mmu-miR-223-3p	217012	Unc45b	0	0	0	1	0	1
mmu-miR-223-3p	217030	Synrg	0	0	0	1	0	1
mmu-miR-223-3p	217031	Tada2a	0	1	0	0	0	1
mmu-miR-223-3p	217039	Ggnbp2	0	0	0	1	0	1
mmu-miR-223-3p	217069	Trim25	0	0	0	1	0	1
mmu-miR-223-3p	217119	Xylt2	0	0	0	1	0	1
mmu-miR-223-3p	217138	Prr15l	1	0	0	0	0	1
mmu-miR-223-3p	217151	Arl5c	0	0	0	1	0	1
mmu-miR-223-3p	217154	Stac2	0	0	0	1	0	1
mmu-miR-223-3p	217216	BC030867	0	0	0	1	0	1
mmu-miR-223-3p	217217	Asb16	0	1	0	0	0	1
mmu-miR-223-3p	217305	Cd300ld	0	0	1	0	0	1
mmu-miR-223-3p	217306	Cd300e	0	0	0	1	0	1
mmu-miR-223-3p	217310	Hid1	0	0	0	1	0	1
mmu-miR-223-3p	217335	Fbf1	0	0	0	1	0	1
mmu-miR-223-3p	217337	Srp68	0	0	0	1	0	1
mmu-miR-223-3p	217342	Ube2o	0	0	0	1	0	1
mmu-miR-223-3p	217353	Tmc6	0	0	0	1	0	1
mmu-miR-223-3p	217369	Uts2r	0	0	0	1	0	1
mmu-miR-223-3p	217370	BC017643	0	0	0	1	0	1
mmu-miR-223-3p	217430	Pqlc3	0	0	0	1	0	1
mmu-miR-223-3p	217431	Nol10	0	0	0	1	0	1
mmu-miR-223-3p	217463	Snx13	0	0	0	1	0	1
mmu-miR-223-3p	217517	Stxbp6	0	0	0	1	0	1
mmu-miR-223-3p	217648	Gm527	0	0	0	1	0	1
mmu-miR-223-3p	217674	Gphb5	0	0	0	1	0	1
mmu-miR-223-3p	217695	Zfyve1	0	0	0	1	0	1
mmu-miR-223-3p	217698	Acot5	0	0	0	1	0	1
mmu-miR-223-3p	217700	Acot6	0	0	0	1	0	1
mmu-miR-223-3p	217715	Eif2b2	0	0	0	1	0	1
mmu-miR-223-3p	217716	Mlh3	0	0	0	1	0	1
mmu-miR-223-3p	217718	Nek9	0	0	0	1	0	1
mmu-miR-223-3p	217779	Lysmd1	0	0	0	1	0	1
mmu-miR-223-3p	217827	Nrde2	0	0	0	1	0	1

mmu-miR-223-3p	217835	Rin3	0	0	0	1	0	1
mmu-miR-223-3p	217843	Unc79	0	0	0	1	0	1
mmu-miR-223-3p	217845	Ifi27l2b	0	0	0	1	0	1
mmu-miR-223-3p	217847	Serpina10	0	0	0	1	0	1
mmu-miR-223-3p	217864	Rcor1	0	0	0	1	0	1
mmu-miR-223-3p	217887	BC022687	0	0	0	1	0	1
mmu-miR-223-3p	217944	Rapgef5	0	0	0	1	0	1
mmu-miR-223-3p	218121	Mboat1	0	0	0	1	0	1
mmu-miR-223-3p	218138	Gmds	0	0	0	1	0	1
mmu-miR-223-3p	218203	Mylip	0	0	1	0	0	1
mmu-miR-223-3p	218236	Fam120a	0	0	0	1	0	1
mmu-miR-223-3p	218268	Eif4e1b	0	0	0	1	0	1
mmu-miR-223-3p	218311	Zfp455	1	0	0	0	0	1
mmu-miR-223-3p	218341	Rfesd	0	0	0	1	0	1
mmu-miR-223-3p	218506	Mrps27	1	0	0	0	0	1
mmu-miR-223-3p	218518	Marveld2	0	0	0	1	0	1
mmu-miR-223-3p	218581	Depdc1b	0	0	0	1	0	1
mmu-miR-223-3p	218624	Il31ra	1	0	0	0	0	1
mmu-miR-223-3p	218693	Paip1	0	0	0	1	0	1
mmu-miR-223-3p	218699	Pxk	0	0	0	1	0	1
mmu-miR-223-3p	218734	3830406C13Rik	0	0	0	1	0	1
mmu-miR-223-3p	218763	Lrrc3b	1	0	0	0	0	1
mmu-miR-223-3p	218793	Ube2e2	0	0	0	1	0	1
mmu-miR-223-3p	218832	Polr3a	0	0	0	1	0	1
mmu-miR-223-3p	218850	D14Abb1e	0	0	0	1	0	1
mmu-miR-223-3p	218865	Chdh	0	0	0	1	0	1
mmu-miR-223-3p	219026	Eddm3b	0	0	0	1	0	1
mmu-miR-223-3p	219033	Ang4	0	0	0	1	0	1
mmu-miR-223-3p	219114	Ska3	0	0	1	0	0	1
mmu-miR-223-3p	219131	Phf11a	0	0	0	1	0	1
mmu-miR-223-3p	219132	Phf11d	0	0	0	1	0	1
mmu-miR-223-3p	219134	Shisa2	0	0	0	1	0	1
mmu-miR-223-3p	219150	Hmbox1	0	0	0	1	0	1
mmu-miR-223-3p	219158	Ccar2	0	0	0	1	0	1
mmu-miR-223-3p	219249	Tdrd3	0	0	1	0	0	1
mmu-miR-223-3p	223255	Stk24	0	0	0	1	0	1
mmu-miR-223-3p	223435	Trio	0	0	1	0	0	1
mmu-miR-223-3p	223642	Zc3h3	0	0	0	1	0	1
mmu-miR-223-3p	223650	Eppk1	0	0	0	1	0	1
mmu-miR-223-3p	223658	Mroh1	0	0	0	1	0	1
mmu-miR-223-3p	223664	Lrrc14	0	0	0	1	0	1
mmu-miR-223-3p	223665	C030006K11Rik	0	0	0	1	0	1
mmu-miR-223-3p	223666	Arhgap39	0	0	0	1	0	1
mmu-miR-223-3p	223669	Zfp7	0	0	0	1	0	1
mmu-miR-223-3p	223693	Tmem184b	0	0	0	1	0	1
mmu-miR-223-3p	223697	Sun2	0	0	0	1	0	1
mmu-miR-223-3p	223723	Ttll12	0	0	0	1	0	1
mmu-miR-223-3p	223774	Alg12	1	0	0	0	0	1
mmu-miR-223-3p	223775	Pim3	0	0	0	1	0	1
mmu-miR-223-3p	223809	Smgc	0	0	0	1	0	1
mmu-miR-223-3p	223827	Gxylt1	0	0	0	1	0	1
mmu-miR-223-3p	223828	Pphln1	0	0	0	1	0	1
mmu-miR-223-3p	223864	Rapgef3	0	0	0	1	0	1
mmu-miR-223-3p	223870	Senp1	0	0	0	1	0	1
mmu-miR-223-3p	223881	Rnd1	0	1	0	0	0	1

mmu-miR-223-3p	223915	Krt73	0	0	0	1	0	1
mmu-miR-223-3p	223917	Krt79	0	0	0	1	0	1
mmu-miR-223-3p	223989	Marf1	0	0	0	1	0	1
mmu-miR-223-3p	224019	Tmem191c	0	0	0	1	0	1
mmu-miR-223-3p	224024	Scarf2	0	0	0	1	0	1
mmu-miR-223-3p	224079	Atp13a4	0	0	0	1	0	1
mmu-miR-223-3p	224088	Atp13a3	0	0	0	1	0	1
mmu-miR-223-3p	224093	Fam43a	0	0	0	1	0	1
mmu-miR-223-3p	224109	Nrros	1	0	0	0	0	1
mmu-miR-223-3p	224143	Poglut1	0	0	0	1	0	1
mmu-miR-223-3p	224171	C330027C09Rik	0	0	0	1	0	1
mmu-miR-223-3p	224247	E330017A01Rik	0	1	0	0	0	1
mmu-miR-223-3p	224318	Speer2	0	0	0	1	0	1
mmu-miR-223-3p	224405	Cyyr1	0	0	0	1	0	1
mmu-miR-223-3p	224419	Map3k7cl	0	0	0	1	0	1
mmu-miR-223-3p	224432	Scaf4	0	0	0	1	0	1
mmu-miR-223-3p	224585	Zfp160	0	0	0	1	0	1
mmu-miR-223-3p	224613	Flywch1	0	0	0	1	0	1
mmu-miR-223-3p	224624	Rab40c	0	0	0	1	0	1
mmu-miR-223-3p	224647	D17Wsu92e	0	0	0	1	0	1
mmu-miR-223-3p	224650	Anks1	0	0	0	1	0	1
mmu-miR-223-3p	224656	Zfp523	0	0	1	0	0	1
mmu-miR-223-3p	224674	Slc37a1	0	0	0	1	0	1
mmu-miR-223-3p	224694	Zfp81	0	0	0	1	0	1
mmu-miR-223-3p	224697	Adamts10	0	0	0	1	0	1
mmu-miR-223-3p	224705	Vps52	0	0	1	0	0	1
mmu-miR-223-3p	224762	Trim31	0	0	0	1	0	1
mmu-miR-223-3p	224814	Abcc10	0	0	0	1	0	1
mmu-miR-223-3p	224824	Pex6	0	0	0	1	0	1
mmu-miR-223-3p	224826	Ubr2	0	0	0	1	0	1
mmu-miR-223-3p	224829	Trerf1	0	0	0	1	0	1
mmu-miR-223-3p	224833	AI661453	0	0	0	1	0	1
mmu-miR-223-3p	224860	Plcl2	0	0	0	1	0	1
mmu-miR-223-3p	224904	2410015M20Rik	0	0	0	1	0	1
mmu-miR-223-3p	224907	Dus3l	0	0	0	1	0	1
mmu-miR-223-3p	224912	Crb3	0	0	0	1	0	1
mmu-miR-223-3p	225030	Kcng3	0	0	0	1	0	1
mmu-miR-223-3p	225055	Fbxo11	0	0	0	1	0	1
mmu-miR-223-3p	225152	Gjd4	1	0	0	0	0	1
mmu-miR-223-3p	225164	Mib1	0	1	0	0	0	1
mmu-miR-223-3p	225182	Rbbp8	0	0	0	1	0	1
mmu-miR-223-3p	225280	Ino80c	0	0	1	0	0	1
mmu-miR-223-3p	225341	Lims2	1	0	0	0	0	1
mmu-miR-223-3p	225348	Wdr36	0	0	0	1	0	1
mmu-miR-223-3p	225358	Fam13b	0	0	0	1	0	1
mmu-miR-223-3p	225362	Reep2	0	0	0	1	0	1
mmu-miR-223-3p	225392	Rell2	0	0	0	1	0	1
mmu-miR-223-3p	225518	Prdm6	0	0	0	1	0	1
mmu-miR-223-3p	225579	Slc27a6	0	0	0	1	0	1
mmu-miR-223-3p	225594	Gm4841	0	0	0	1	0	1
mmu-miR-223-3p	225608	Sh3tc2	0	0	0	1	0	1
mmu-miR-223-3p	225642	Grp	0	0	0	1	0	1
mmu-miR-223-3p	225651	Mppe1	0	0	0	1	0	1
mmu-miR-223-3p	225655	Slmo1	0	0	0	1	0	1
mmu-miR-223-3p	225659	Cep76	0	0	0	1	0	1

mmu-miR-223-3p	225791	Zadh2	0	0	0	1	0	1
mmu-miR-223-3p	225825	Cd226	0	0	0	1	0	1
mmu-miR-223-3p	225870	Rin1	0	0	0	1	0	1
mmu-miR-223-3p	225887	Ndufs8	0	0	0	1	0	1
mmu-miR-223-3p	225898	Eml3	0	0	0	1	0	1
mmu-miR-223-3p	225908	Myrf	0	0	0	1	0	1
mmu-miR-223-3p	225923	Gm97	0	0	0	1	0	1
mmu-miR-223-3p	225994	Nmrk1	0	0	0	1	0	1
mmu-miR-223-3p	225995	D030056L22Rik	0	0	0	1	0	1
mmu-miR-223-3p	225997	Trpm6	0	0	0	1	0	1
mmu-miR-223-3p	226075	Glis3	0	1	0	0	0	1
mmu-miR-223-3p	226089	C030046E11Rik	0	0	0	1	0	1
mmu-miR-223-3p	226115	Opalin	0	0	0	1	0	1
mmu-miR-223-3p	226122	Ubtd1	0	0	0	1	0	1
mmu-miR-223-3p	226123	Morn4	0	0	0	1	0	1
mmu-miR-223-3p	226154	Lzts2	0	0	0	1	0	1
mmu-miR-223-3p	226243	Habp2	0	0	0	1	0	1
mmu-miR-223-3p	226245	Plekhs1	0	0	0	1	0	1
mmu-miR-223-3p	226255	Atrnl1	0	0	0	1	0	1
mmu-miR-223-3p	226359	C1ql2	0	1	0	0	0	1
mmu-miR-223-3p	226407	Rab3gap1	0	0	0	1	0	1
mmu-miR-223-3p	226412	R3hdm1	0	0	0	1	0	1
mmu-miR-223-3p	226413	Lct	0	0	0	1	0	1
mmu-miR-223-3p	226414	Dars	0	0	0	1	0	1
mmu-miR-223-3p	226422	Rab7l1	0	0	0	1	0	1
mmu-miR-223-3p	226442	Zfp281	0	0	0	1	0	1
mmu-miR-223-3p	226499	BC003331	0	0	0	1	0	1
mmu-miR-223-3p	226539	Dars2	0	0	0	1	0	1
mmu-miR-223-3p	226548	Aph1a	0	0	0	1	0	1
mmu-miR-223-3p	226551	Suco	0	0	0	1	0	1
mmu-miR-223-3p	226564	Fmo4	0	0	0	1	0	1
mmu-miR-223-3p	226594	Rcsd1	0	0	0	1	0	1
mmu-miR-223-3p	226601	Gm4846	0	0	0	1	0	1
mmu-miR-223-3p	226604	Gm4847	0	0	0	1	0	1
mmu-miR-223-3p	226695	Ifi205	0	0	0	1	0	1
mmu-miR-223-3p	226747	Ahctf1	0	1	0	0	0	1
mmu-miR-223-3p	226778	Mark1	0	0	0	1	0	1
mmu-miR-223-3p	226791	Lyplal1	0	1	0	0	0	1
mmu-miR-223-3p	226849	Ppp2r5a	0	0	1	0	0	1
mmu-miR-223-3p	226861	Hhat	0	1	0	0	0	1
mmu-miR-223-3p	226866	Sbspon	0	0	0	1	0	1
mmu-miR-223-3p	226970	Arhgef4	1	0	0	0	0	1
mmu-miR-223-3p	227059	Slc39a10	0	0	0	1	0	1
mmu-miR-223-3p	227157	Mpp4	1	0	0	0	0	1
mmu-miR-223-3p	227292	Ctdsp1	0	0	0	1	0	1
mmu-miR-223-3p	227298	Fam134a	0	0	0	1	0	1
mmu-miR-223-3p	227325	Dner	0	0	0	1	0	1
mmu-miR-223-3p	227326	Gpr55	0	0	0	1	0	1
mmu-miR-223-3p	227333	Dgkd	0	0	0	1	0	1
mmu-miR-223-3p	227358	Fam132b	0	0	1	0	0	1
mmu-miR-223-3p	227545	Proser2	0	0	0	1	0	1
mmu-miR-223-3p	227580	C1ql3	0	0	0	1	0	1
mmu-miR-223-3p	227615	Tmem203	0	0	0	1	0	1
mmu-miR-223-3p	227620	Uap1l1	0	1	0	0	0	1
mmu-miR-223-3p	227624	Rabl6	0	0	0	1	0	1

mmu-miR-223-3p	227630	Lcn11	0	0	0	1	0	1
mmu-miR-223-3p	227683	Coq4	0	0	0	1	0	1
mmu-miR-223-3p	227696	Phyhd1	0	0	0	1	0	1
mmu-miR-223-3p	227697	Dolk	0	0	0	1	0	1
mmu-miR-223-3p	227699	Nup188	0	0	0	1	0	1
mmu-miR-223-3p	227700	Sh3glb2	0	0	0	1	0	1
mmu-miR-223-3p	227721	Ppapdc3	0	0	0	1	0	1
mmu-miR-223-3p	227733	Pip5kl1	0	0	0	1	0	1
mmu-miR-223-3p	227736	1700019L03Rik	0	0	0	1	0	1
mmu-miR-223-3p	227738	Lrsam1	0	0	0	1	0	1
mmu-miR-223-3p	227743	Mapkap1	0	0	0	1	0	1
mmu-miR-223-3p	228003	Klhl41	0	0	0	1	0	1
mmu-miR-223-3p	228136	Zdhhc5	0	0	0	1	0	1
mmu-miR-223-3p	228151	4833423E24Rik	0	0	0	1	0	1
mmu-miR-223-3p	228228	Olfr1102	0	0	0	1	0	1
mmu-miR-223-3p	228355	Madd	0	0	0	1	0	1
mmu-miR-223-3p	228361	Ambra1	0	0	0	1	0	1
mmu-miR-223-3p	228413	Prrg4	0	0	0	1	0	1
mmu-miR-223-3p	228421	Kif18a	0	0	0	1	0	1
mmu-miR-223-3p	228432	Ano3	0	0	0	1	0	1
mmu-miR-223-3p	228491	Zfp770	0	0	0	1	0	1
mmu-miR-223-3p	228545	Vps18	0	0	0	1	0	1
mmu-miR-223-3p	228550	Itpka	0	0	0	1	0	1
mmu-miR-223-3p	228592	F830045P16Rik	0	0	0	1	0	1
mmu-miR-223-3p	228607	Mavs	0	0	0	1	0	1
mmu-miR-223-3p	228608	Smox	0	0	0	1	0	1
mmu-miR-223-3p	228756	Cstl1	0	0	0	1	0	1
mmu-miR-223-3p	228765	Sdcbp2	0	0	0	1	0	1
mmu-miR-223-3p	228775	Trib3	0	0	0	1	0	1
mmu-miR-223-3p	228778	6820408C15Rik	0	0	0	1	0	1
mmu-miR-223-3p	228807	Zfp341	0	0	0	1	0	1
mmu-miR-223-3p	228812	Pigu	0	0	0	1	0	1
mmu-miR-223-3p	228836	Dlgap4	0	0	0	1	0	1
mmu-miR-223-3p	228839	Tgif2	0	0	0	1	0	1
mmu-miR-223-3p	228846	D630003M21Rik	0	0	0	1	0	1
mmu-miR-223-3p	228859	Fitm2	0	0	0	1	0	1
mmu-miR-223-3p	228869	Ncoa5	0	0	0	1	0	1
mmu-miR-223-3p	228875	Slc35c2	0	0	0	1	0	1
mmu-miR-223-3p	228960	Stx16	0	0	0	1	0	1
mmu-miR-223-3p	228961	Npepl1	0	0	1	0	0	1
mmu-miR-223-3p	228993	Slc17a9	0	0	0	1	0	1
mmu-miR-223-3p	228994	Ythdf1	0	0	0	1	0	1
mmu-miR-223-3p	229003	Helz2	0	0	0	1	0	1
mmu-miR-223-3p	229011	Samd10	0	0	0	1	0	1
mmu-miR-223-3p	229096	Ythdf3	0	0	0	1	0	1
mmu-miR-223-3p	229227	4932438A13Rik	0	0	0	1	0	1
mmu-miR-223-3p	229323	Gpr171	0	0	0	1	0	1
mmu-miR-223-3p	229333	C130079G13Rik	0	0	0	1	0	1
mmu-miR-223-3p	229474	Fhdc1	0	0	0	1	0	1
mmu-miR-223-3p	229487	Pet112	0	1	0	0	0	1
mmu-miR-223-3p	229534	Pbxip1	0	0	0	1	0	1
mmu-miR-223-3p	229541	Dennd4b	0	1	0	0	0	1
mmu-miR-223-3p	229542	Gatad2b	0	0	0	1	0	1
mmu-miR-223-3p	229543	Ints3	0	0	0	1	0	1
mmu-miR-223-3p	229615	Pias3	0	0	0	1	0	1

mmu-miR-223-3p	229663	Csde1	0	0	0	1	0	1
mmu-miR-223-3p	229714	Gpr61	0	0	1	0	0	1
mmu-miR-223-3p	229715	Amigo1	0	0	0	1	0	1
mmu-miR-223-3p	229731	Slc25a24	0	0	0	1	0	1
mmu-miR-223-3p	229776	Cdc14a	0	0	0	1	0	1
mmu-miR-223-3p	229898	Gbp5	0	0	0	1	0	1
mmu-miR-223-3p	229906	Gtf2b	0	0	0	1	0	1
mmu-miR-223-3p	229937	Znhit6	0	0	0	1	0	1
mmu-miR-223-3p	229949	Ak5	0	0	0	1	0	1
mmu-miR-223-3p	230025	Prdm13	0	0	0	1	0	1
mmu-miR-223-3p	230027	Coq3	0	0	0	1	0	1
mmu-miR-223-3p	230082	Nol6	0	0	0	1	0	1
mmu-miR-223-3p	230085	N28178	0	0	0	1	0	1
mmu-miR-223-3p	230098	Arhgef39	0	0	0	1	0	1
mmu-miR-223-3p	230103	Npr2	0	0	0	1	0	1
mmu-miR-223-3p	230119	Zbtb5	0	0	0	1	0	1
mmu-miR-223-3p	230161	Acnat1	0	0	0	1	0	1
mmu-miR-223-3p	230163	Aldob	0	0	0	1	0	1
mmu-miR-223-3p	230396	Ifna13	1	0	0	0	0	1
mmu-miR-223-3p	230405	Ifne	0	0	0	1	0	1
mmu-miR-223-3p	230484	Usp1	1	0	0	0	0	1
mmu-miR-223-3p	230500	Efcab7	0	0	0	1	0	1
mmu-miR-223-3p	230558	C8a	0	0	0	1	0	1
mmu-miR-223-3p	230576	Ttc22	0	0	0	1	0	1
mmu-miR-223-3p	230577	Pars2	0	0	0	1	0	1
mmu-miR-223-3p	230587	Glis1	0	0	0	1	0	1
mmu-miR-223-3p	230590	Zyg11a	0	0	0	1	0	1
mmu-miR-223-3p	230594	Zcchc11	0	0	0	1	0	1
mmu-miR-223-3p	230612	Slc5a9	0	0	0	1	0	1
mmu-miR-223-3p	230654	Lrrc41	0	0	0	1	0	1
mmu-miR-223-3p	230657	Tmem69	0	0	0	1	0	1
mmu-miR-223-3p	230661	Tesk2	0	0	0	1	0	1
mmu-miR-223-3p	230674	Kdm4a	0	0	0	1	0	1
mmu-miR-223-3p	230676	Szt2	0	0	0	1	0	1
mmu-miR-223-3p	230709	Zmpste24	1	0	0	0	0	1
mmu-miR-223-3p	230721	Pabpc4	0	0	0	1	0	1
mmu-miR-223-3p	230726	Rhbdl2	1	0	0	0	0	1
mmu-miR-223-3p	230734	Yrdc	0	0	0	1	0	1
mmu-miR-223-3p	230738	Zc3h12a	0	0	0	1	0	1
mmu-miR-223-3p	230751	Oscp1	0	0	0	1	0	1
mmu-miR-223-3p	230752	Eva1b	1	0	0	0	0	1
mmu-miR-223-3p	230775	Bai2	0	0	0	1	0	1
mmu-miR-223-3p	230777	Hcrtr1	0	0	0	1	0	1
mmu-miR-223-3p	230789	Fam76a	0	0	0	1	0	1
mmu-miR-223-3p	230796	Wdtc1	0	0	0	1	0	1
mmu-miR-223-3p	230801	Pigv	0	0	0	1	0	1
mmu-miR-223-3p	230861	Eif4g3	0	0	0	1	0	1
mmu-miR-223-3p	230866	Emc1	0	0	0	1	0	1
mmu-miR-223-3p	230872	Crocc	0	0	0	1	0	1
mmu-miR-223-3p	230903	Fbxo44	0	0	0	1	0	1
mmu-miR-223-3p	230904	Fbxo2	0	0	0	1	0	1
mmu-miR-223-3p	230935	Dnajc11	0	0	1	0	0	1
mmu-miR-223-3p	230959	Ajap1	0	0	0	1	0	1
mmu-miR-223-3p	230967	Cep104	0	0	0	1	0	1
mmu-miR-223-3p	230971	Megf6	0	0	0	1	0	1

mmu-miR-223-3p	230972	Arhgef16	0	0	0	1	0	1
mmu-miR-223-3p	230991	B930041F14Rik	0	0	0	1	0	1
mmu-miR-223-3p	231003	Klhl17	0	0	0	1	0	1
mmu-miR-223-3p	231004	Samd11	1	0	0	0	0	1
mmu-miR-223-3p	231042	Nupl2	0	0	0	1	0	1
mmu-miR-223-3p	231050	Galnt11	0	0	0	1	0	1
mmu-miR-223-3p	231086	Hadhb	0	0	0	1	0	1
mmu-miR-223-3p	231093	Agbl5	0	0	0	1	0	1
mmu-miR-223-3p	231098	Dnajc5g	0	0	0	1	0	1
mmu-miR-223-3p	231103	Gckr	0	0	0	1	0	1
mmu-miR-223-3p	231125	Zfyve28	0	0	0	1	0	1
mmu-miR-223-3p	231128	Fam193a	0	0	0	1	0	1
mmu-miR-223-3p	231130	Tnip2	0	0	0	1	0	1
mmu-miR-223-3p	231147	Sh3tc1	0	1	0	0	0	1
mmu-miR-223-3p	231252	Chrna9	0	0	0	1	0	1
mmu-miR-223-3p	231290	Slc10a4	0	0	0	1	0	1
mmu-miR-223-3p	231296	Lrrc66	0	0	0	1	0	1
mmu-miR-223-3p	231386	Ythdc1	0	0	0	1	0	1
mmu-miR-223-3p	231396	Ugt2b36	0	0	0	1	0	1
mmu-miR-223-3p	231452	Sdad1	0	0	0	1	0	1
mmu-miR-223-3p	231506	Lin54	0	0	0	1	0	1
mmu-miR-223-3p	231532	Arhgap24	0	0	0	1	0	1
mmu-miR-223-3p	231570	A830010M20Rik	0	0	0	1	0	1
mmu-miR-223-3p	231571	Rpap2	1	0	0	0	0	1
mmu-miR-223-3p	231583	Slc26a1	0	0	0	1	0	1
mmu-miR-223-3p	231605	Galnt9	0	0	0	1	0	1
mmu-miR-223-3p	231630	Ficd	0	0	0	1	0	1
mmu-miR-223-3p	231655	Oasl1	0	0	0	1	0	1
mmu-miR-223-3p	231659	Gcn1l1	0	0	0	1	0	1
mmu-miR-223-3p	231699	Oas1e	0	0	0	1	0	1
mmu-miR-223-3p	231712	Trafd1	0	0	0	1	0	1
mmu-miR-223-3p	231713	Naa25	0	0	0	1	0	1
mmu-miR-223-3p	231724	Rad9b	0	0	0	1	0	1
mmu-miR-223-3p	231727	B3gnt4	0	0	0	1	0	1
mmu-miR-223-3p	231798	Lrch4	0	0	0	1	0	1
mmu-miR-223-3p	231801	Agfg2	0	0	0	1	0	1
mmu-miR-223-3p	231803	Mepce	0	0	0	1	0	1
mmu-miR-223-3p	231807	BC037034	0	0	0	1	0	1
mmu-miR-223-3p	231832	Tmem184a	0	0	0	1	0	1
mmu-miR-223-3p	231834	Snx8	0	0	0	1	0	1
mmu-miR-223-3p	231841	Brat1	0	0	0	1	0	1
mmu-miR-223-3p	231842	Amz1	0	0	0	1	0	1
mmu-miR-223-3p	231855	Ap5z1	0	0	0	1	0	1
mmu-miR-223-3p	231858	Radil	0	0	0	1	0	1
mmu-miR-223-3p	231863	Fbxl18	0	1	0	0	0	1
mmu-miR-223-3p	231871	Daglb	0	0	0	1	0	1
mmu-miR-223-3p	231874	Ccz1	0	0	0	1	0	1
mmu-miR-223-3p	231887	Pdap1	0	0	0	1	0	1
mmu-miR-223-3p	231931	Gimap6	0	0	0	1	0	1
mmu-miR-223-3p	231932	Gimap7	0	0	0	1	0	1
mmu-miR-223-3p	231946	Fam221a	0	0	0	1	0	1
mmu-miR-223-3p	232078	Thnsl2	0	0	0	1	0	1
mmu-miR-223-3p	232086	Tmem150a	0	0	0	1	0	1
mmu-miR-223-3p	232089	Elmod3	0	0	0	1	0	1
mmu-miR-223-3p	232196	C87436	0	0	0	1	0	1

mmu-miR-223-3p	232201	Arhgap25	0	0	0	1	0	1
mmu-miR-223-3p	232217	4933427D06Rik	0	0	0	1	0	1
mmu-miR-223-3p	232236	Ccdc174	0	0	0	1	0	1
mmu-miR-223-3p	232288	Frmd4b	0	0	1	0	0	1
mmu-miR-223-3p	232313	Gxylt2	0	0	0	1	0	1
mmu-miR-223-3p	232339	Ankrd26	0	0	0	1	0	1
mmu-miR-223-3p	232370	Clstn3	0	0	0	1	0	1
mmu-miR-223-3p	232371	C1rl	0	0	0	1	0	1
mmu-miR-223-3p	232406	BC035044	0	0	0	1	0	1
mmu-miR-223-3p	232430	Crebl2	0	0	0	1	0	1
mmu-miR-223-3p	232440	H2afj	0	0	0	1	0	1
mmu-miR-223-3p	232491	Pyroxd1	0	0	0	1	0	1
mmu-miR-223-3p	232566	Amn1	0	0	0	1	0	1
mmu-miR-223-3p	232664	Ccdc136	0	0	0	1	0	1
mmu-miR-223-3p	232670	Tspan33	0	0	0	1	0	1
mmu-miR-223-3p	232714	Mgam	0	0	0	1	0	1
mmu-miR-223-3p	232784	Zfp212	0	0	0	1	0	1
mmu-miR-223-3p	232790	Oscar	0	0	0	1	0	1
mmu-miR-223-3p	232791	Cnot3	0	0	0	1	0	1
mmu-miR-223-3p	232798	Leng8	0	0	0	1	0	1
mmu-miR-223-3p	232801	Lilra5	1	0	0	0	0	1
mmu-miR-223-3p	232807	Ppp1r12c	0	0	0	1	0	1
mmu-miR-223-3p	232811	Suv420h2	0	0	0	1	0	1
mmu-miR-223-3p	232813	Shisa7	0	0	0	1	0	1
mmu-miR-223-3p	232821	Ccdc106	0	0	0	1	0	1
mmu-miR-223-3p	232827	Nlrp2	0	0	0	1	0	1
mmu-miR-223-3p	232853	Zfp954	0	0	0	1	0	1
mmu-miR-223-3p	232854	Zfp418	1	0	0	0	0	1
mmu-miR-223-3p	232875	Zscan18	0	0	0	1	0	1
mmu-miR-223-3p	232889	Pla2g4c	0	0	0	1	0	1
mmu-miR-223-3p	232941	Ppm1n	0	1	0	0	0	1
mmu-miR-223-3p	232943	Klc3	0	0	0	1	0	1
mmu-miR-223-3p	232946	Bloc1s3	0	0	0	1	0	1
mmu-miR-223-3p	232947	Ppp1r37	0	0	0	1	0	1
mmu-miR-223-3p	232966	Zfp114	0	0	0	1	0	1
mmu-miR-223-3p	232969	Zfp428	0	0	0	1	0	1
mmu-miR-223-3p	232970	Phldb3	0	0	0	1	0	1
mmu-miR-223-3p	232972	BC049730	0	0	0	1	0	1
mmu-miR-223-3p	232975	Atp1a3	0	0	0	1	0	1
mmu-miR-223-3p	232976	Zfp574	0	0	0	1	0	1
mmu-miR-223-3p	232983	Cxcl17	0	0	0	1	0	1
mmu-miR-223-3p	232984	B3gnt8	0	0	0	1	0	1
mmu-miR-223-3p	233001	Nlrp9a	0	0	0	1	0	1
mmu-miR-223-3p	233005	Cyp2a22	1	0	0	0	0	1
mmu-miR-223-3p	233020	Hipk4	0	0	0	1	0	1
mmu-miR-223-3p	233040	Fbxo27	0	0	0	1	0	1
mmu-miR-223-3p	233058	Zfp420	0	0	0	1	0	1
mmu-miR-223-3p	233073	U2af1l4	0	1	0	0	0	1
mmu-miR-223-3p	233079	Ffar2	0	0	0	1	0	1
mmu-miR-223-3p	233080	Ffar3	0	0	0	1	0	1
mmu-miR-223-3p	233090	Scgb2b24	0	0	0	1	0	1
mmu-miR-223-3p	233099	Scgb2b27	0	0	0	1	0	1
mmu-miR-223-3p	233164	Gm4884	0	0	0	1	0	1
mmu-miR-223-3p	233186	Siglec5	0	0	0	1	0	1
mmu-miR-223-3p	233208	Scaf1	0	0	0	1	0	1

mmu-miR-223-3p	233276	Tubgcp5	0	0	0	1	0	1
mmu-miR-223-3p	233280	Nipa1	0	0	0	1	0	1
mmu-miR-223-3p	233335	Synm	0	0	0	1	0	1
mmu-miR-223-3p	233424	Tmc3	0	1	0	0	0	1
mmu-miR-223-3p	233571	P2ry6	0	0	0	1	0	1
mmu-miR-223-3p	233649	Cnga4	0	0	0	1	0	1
mmu-miR-223-3p	233765	Plekha7	0	0	0	1	0	1
mmu-miR-223-3p	233781	Xylt1	0	0	0	1	0	1
mmu-miR-223-3p	233813	Vwa3a	0	1	0	0	0	1
mmu-miR-223-3p	233824	Cog7	0	0	0	1	0	1
mmu-miR-223-3p	233863	Gtf3c1	0	0	0	1	0	1
mmu-miR-223-3p	233870	Tufm	0	0	0	1	0	1
mmu-miR-223-3p	233871	Atxn2l	0	1	0	0	0	1
mmu-miR-223-3p	233876	Hirip3	0	0	0	1	0	1
mmu-miR-223-3p	233877	Kctd13	0	0	0	1	0	1
mmu-miR-223-3p	233878	Sez6l2	0	0	0	1	0	1
mmu-miR-223-3p	233887	Zfp553	0	0	0	1	0	1
mmu-miR-223-3p	233890	Zfp768	0	0	0	1	0	1
mmu-miR-223-3p	233893	Zfp764	0	0	0	1	0	1
mmu-miR-223-3p	233902	Fbxl19	0	0	0	1	0	1
mmu-miR-223-3p	233905	Zfp646	0	0	0	1	0	1
mmu-miR-223-3p	233912	Armc5	0	0	0	1	0	1
mmu-miR-223-3p	233913	BC017158	0	0	0	1	0	1
mmu-miR-223-3p	233987	Zfp958	0	0	0	1	0	1
mmu-miR-223-3p	234069	Pcid2	0	0	0	1	0	1
mmu-miR-223-3p	234290	BC030500	0	0	0	1	0	1
mmu-miR-223-3p	234311	Ddx60	0	0	0	1	0	1
mmu-miR-223-3p	234344	Naf1	0	0	0	1	0	1
mmu-miR-223-3p	234356	Csgalnact1	0	0	0	1	0	1
mmu-miR-223-3p	234362	Zfp868	0	0	0	1	0	1
mmu-miR-223-3p	234374	Ddx49	0	0	0	1	0	1
mmu-miR-223-3p	234378	Klhl26	0	0	0	1	0	1
mmu-miR-223-3p	234395	Ushbp1	0	0	0	1	0	1
mmu-miR-223-3p	234396	Ankle1	0	0	0	1	0	1
mmu-miR-223-3p	234407	Glt25d1	0	0	1	0	0	1
mmu-miR-223-3p	234413	Zfp961	0	0	0	1	0	1
mmu-miR-223-3p	234463	Tmem184c	0	0	0	1	0	1
mmu-miR-223-3p	234549	Heatr3	0	0	0	1	0	1
mmu-miR-223-3p	234593	Ndrg4	0	0	0	1	0	1
mmu-miR-223-3p	234594	Cnot1	0	0	0	1	0	1
mmu-miR-223-3p	234595	Slc38a7	0	0	0	1	0	1
mmu-miR-223-3p	234677	Ces4a	0	0	0	1	0	1
mmu-miR-223-3p	234699	Edc4	0	0	0	1	0	1
mmu-miR-223-3p	234700	Nrn1l	0	0	0	1	0	1
mmu-miR-223-3p	234723	Txnl4b	0	0	0	1	0	1
mmu-miR-223-3p	234729	Vac14	0	0	0	1	0	1
mmu-miR-223-3p	234736	Rfwd3	0	0	0	1	0	1
mmu-miR-223-3p	234740	Tmem231	0	1	0	0	0	1
mmu-miR-223-3p	234776	Atmin	0	0	0	1	0	1
mmu-miR-223-3p	234825	Klhdc4	0	0	0	1	0	1
mmu-miR-223-3p	234839	Piezo1	0	0	0	1	0	1
mmu-miR-223-3p	234847	Spg7	0	0	0	1	0	1
mmu-miR-223-3p	234854	Cdk10	0	0	0	1	0	1
mmu-miR-223-3p	234865	Nup133	0	0	0	1	0	1
mmu-miR-223-3p	234964	Ccdc67	0	0	0	1	0	1

mmu-miR-223-3p	234967	Slc36a4	0	0	0	1	0	1
mmu-miR-223-3p	235072	Sept7	0	0	0	1	0	1
mmu-miR-223-3p	235130	Adamts15	0	0	0	1	0	1
mmu-miR-223-3p	235184	Msantd2	0	0	0	1	0	1
mmu-miR-223-3p	235281	Scn3b	0	0	0	1	0	1
mmu-miR-223-3p	235283	Gramd1b	0	1	0	0	0	1
mmu-miR-223-3p	235312	C1qtnf5	0	0	0	1	0	1
mmu-miR-223-3p	235406	Snx33	0	0	0	1	0	1
mmu-miR-223-3p	235439	Herc1	0	0	0	1	0	1
mmu-miR-223-3p	235505	Cd109	0	0	0	1	0	1
mmu-miR-223-3p	235527	Plscr4	0	0	0	1	0	1
mmu-miR-223-3p	235533	Gk5	0	0	0	1	0	1
mmu-miR-223-3p	235534	Acpl2	0	0	0	1	0	1
mmu-miR-223-3p	235582	Glyctk	0	0	0	1	0	1
mmu-miR-223-3p	235584	Dusp7	1	0	0	0	0	1
mmu-miR-223-3p	235587	Parp3	0	0	0	1	0	1
mmu-miR-223-3p	235599	6430571L13Rik	0	0	0	1	0	1
mmu-miR-223-3p	235604	Camkv	0	0	0	1	0	1
mmu-miR-223-3p	235612	Fbxw19	1	0	0	0	0	1
mmu-miR-223-3p	235623	Scap	0	0	0	1	0	1
mmu-miR-223-3p	235626	Setd2	0	0	0	1	0	1
mmu-miR-223-3p	235627	Nbeal2	0	0	0	1	0	1
mmu-miR-223-3p	235779	Nlrp4g	0	0	0	1	0	1
mmu-miR-223-3p	236082	Dhrsx	0	0	0	1	0	1
mmu-miR-223-3p	236149	Slc22a26	0	0	0	1	0	1
mmu-miR-223-3p	236193	Zfp709	0	0	0	1	0	1
mmu-miR-223-3p	236219	Tcstv3	0	0	0	1	0	1
mmu-miR-223-3p	236266	Alms1	1	0	0	0	0	1
mmu-miR-223-3p	236727	Slc9a7	0	0	0	1	0	1
mmu-miR-223-3p	236732	Rbm10	0	0	0	1	0	1
mmu-miR-223-3p	236733	Usp11	0	0	0	1	0	1
mmu-miR-223-3p	236848	BC023829	0	0	0	1	0	1
mmu-miR-223-3p	236874	Gm14743	0	0	0	1	0	1
mmu-miR-223-3p	236904	Klhl15	0	0	0	1	0	1
mmu-miR-223-3p	236930	Ercc6l	0	1	0	0	0	1
mmu-miR-223-3p	237029	4932411N23Rik	0	0	0	1	0	1
mmu-miR-223-3p	237038	Nox1	0	0	0	1	0	1
mmu-miR-223-3p	237091	Lhfpl1	0	0	0	1	0	1
mmu-miR-223-3p	237175	Gpr64	0	0	0	1	0	1
mmu-miR-223-3p	237222	Ofd1	0	0	0	1	0	1
mmu-miR-223-3p	237256	Zc3h12d	0	0	0	1	0	1
mmu-miR-223-3p	237310	Il22ra2	0	0	0	1	0	1
mmu-miR-223-3p	237313	Il20ra	0	0	0	1	0	1
mmu-miR-223-3p	237339	L3mbtl3	0	0	0	1	0	1
mmu-miR-223-3p	237360	Adamts14	0	0	1	0	0	1
mmu-miR-223-3p	237387	Lrrc3	0	0	0	1	0	1
mmu-miR-223-3p	237433	Gm4925	1	0	0	0	0	1
mmu-miR-223-3p	237500	Tmtc3	0	0	0	1	0	1
mmu-miR-223-3p	237560	Lrrc10	0	0	0	1	0	1
mmu-miR-223-3p	237611	Stac3	0	0	0	1	0	1
mmu-miR-223-3p	237636	Npc1l1	0	0	0	1	0	1
mmu-miR-223-3p	237716	Gpr75	0	0	0	1	0	1
mmu-miR-223-3p	237754	Btnl9	0	0	0	1	0	1
mmu-miR-223-3p	237758	Zfp454	0	0	0	1	0	1
mmu-miR-223-3p	237823	Pfas	0	0	0	1	0	1

mmu-miR-223-3p	237831	Slc13a5	0	0	1	0	0	1
mmu-miR-223-3p	237868	Sarm1	0	0	0	1	0	1
mmu-miR-223-3p	237890	Slfn14	0	0	0	1	0	1
mmu-miR-223-3p	237891	Gas2l2	0	1	0	0	0	1
mmu-miR-223-3p	237926	Rsad1	0	0	0	1	0	1
mmu-miR-223-3p	237930	Ttll6	0	0	0	1	0	1
mmu-miR-223-3p	237987	Otop2	0	0	0	1	0	1
mmu-miR-223-3p	237988	Cdr2l	0	0	0	1	0	1
mmu-miR-223-3p	238023	Hexdc	0	0	0	1	0	1
mmu-miR-223-3p	238024	Fn3krp	0	0	0	1	0	1
mmu-miR-223-3p	238037	BC068281	0	0	0	1	0	1
mmu-miR-223-3p	238055	Apob	0	0	0	1	0	1
mmu-miR-223-3p	238217	Rpl10l	0	0	0	1	0	1
mmu-miR-223-3p	238247	Arid4a	0	0	0	1	0	1
mmu-miR-223-3p	238257	Tmem30b	0	0	0	1	0	1
mmu-miR-223-3p	238271	Kcnh5	0	0	0	1	0	1
mmu-miR-223-3p	238323	Rps6kl1	1	0	0	0	0	1
mmu-miR-223-3p	238328	Vash1	0	0	0	1	0	1
mmu-miR-223-3p	238377	Gpr68	0	0	0	1	0	1
mmu-miR-223-3p	238393	Serpina3f	0	0	0	1	0	1
mmu-miR-223-3p	238395	Serpina3j	0	0	0	1	0	1
mmu-miR-223-3p	238463	Tubal3	0	0	0	1	0	1
mmu-miR-223-3p	238505	Mtr	0	0	0	1	0	1
mmu-miR-223-3p	238555	Btn2a2	0	0	0	1	0	1
mmu-miR-223-3p	238690	Zfp458	0	0	0	1	0	1
mmu-miR-223-3p	238693	Zfp58	0	0	0	1	0	1
mmu-miR-223-3p	238722	Zfp72	0	0	0	1	0	1
mmu-miR-223-3p	238726	Fam81b	0	0	0	1	0	1
mmu-miR-223-3p	238829	Gm4937	0	0	0	1	0	1
mmu-miR-223-3p	238875	Gapt	1	0	0	0	0	1
mmu-miR-223-3p	239027	Arhgap22	0	0	0	1	0	1
mmu-miR-223-3p	239083	Ccnb1ip1	0	0	0	1	0	1
mmu-miR-223-3p	239122	Setdb2	0	0	0	1	0	1
mmu-miR-223-3p	239126	C1qtnf9	0	0	0	1	0	1
mmu-miR-223-3p	239134	Gucy1b2	0	0	0	1	0	1
mmu-miR-223-3p	239167	Synb	0	1	0	0	0	1
mmu-miR-223-3p	239170	Fam160b2	0	0	0	1	0	1
mmu-miR-223-3p	239319	Card6	0	0	0	1	0	1
mmu-miR-223-3p	239393	Lrp12	0	0	0	1	0	1
mmu-miR-223-3p	239405	Rspo2	0	0	0	1	0	1
mmu-miR-223-3p	239435	Aard	0	0	0	1	0	1
mmu-miR-223-3p	239530	Gpr20	0	0	1	0	0	1
mmu-miR-223-3p	239546	Zfp647	0	0	0	1	0	1
mmu-miR-223-3p	239552	Apol8	0	0	0	1	0	1
mmu-miR-223-3p	239559	A4galt	0	0	0	1	0	1
mmu-miR-223-3p	239591	Ttll8	0	0	1	0	0	1
mmu-miR-223-3p	239618	Pdzrn4	0	0	0	1	0	1
mmu-miR-223-3p	239652	Zfp641	0	0	0	1	0	1
mmu-miR-223-3p	239706	Mettl22	1	0	0	0	0	1
mmu-miR-223-3p	239766	Rtp1	0	0	0	1	0	1
mmu-miR-223-3p	239790	Ostn	1	0	0	0	0	1
mmu-miR-223-3p	239849	Cd200r4	0	0	0	1	0	1
mmu-miR-223-3p	240064	Zfp799	0	0	0	1	0	1
mmu-miR-223-3p	240067	Zfp952	1	0	0	0	0	1
mmu-miR-223-3p	240087	Mdc1	0	0	0	1	0	1

mmu-miR-223-3p	240120	Zfp119b	0	0	0	1	0	1
mmu-miR-223-3p	240121	Fsd1	0	0	0	1	0	1
mmu-miR-223-3p	240186	Zfp438	1	0	0	0	0	1
mmu-miR-223-3p	240216	E230025N22Rik	0	0	0	1	0	1
mmu-miR-223-3p	240239	Gpr151	0	0	0	1	0	1
mmu-miR-223-3p	240255	Ythdc2	0	0	0	1	0	1
mmu-miR-223-3p	240261	Ccdc112	0	0	0	1	0	1
mmu-miR-223-3p	240263	Fem1c	0	0	0	1	0	1
mmu-miR-223-3p	240332	Slc6a7	0	0	0	1	0	1
mmu-miR-223-3p	240479	Fam69c	0	0	0	1	0	1
mmu-miR-223-3p	240505	Cdc42bpg	0	0	0	1	0	1
mmu-miR-223-3p	240549	Gm4952	0	0	0	1	0	1
mmu-miR-223-3p	240590	Dmrt3	1	0	0	0	0	1
mmu-miR-223-3p	240613	9930021J03Rik	1	0	0	0	0	1
mmu-miR-223-3p	240660	Slc35g1	0	0	0	1	0	1
mmu-miR-223-3p	240675	Vwa2	0	0	0	1	0	1
mmu-miR-223-3p	240753	Plekha6	0	0	0	1	0	1
mmu-miR-223-3p	240756	Klhl12	0	0	0	1	0	1
mmu-miR-223-3p	240776	Kcnt2	0	0	0	1	0	1
mmu-miR-223-3p	240816	Rgsl1	0	0	0	1	0	1
mmu-miR-223-3p	240817	5830403L16Rik	0	0	0	1	0	1
mmu-miR-223-3p	240888	Gpr161	0	0	0	1	0	1
mmu-miR-223-3p	240892	Dusp27	0	0	0	1	0	1
mmu-miR-223-3p	240913	Adamts4	0	0	0	1	0	1
mmu-miR-223-3p	240916	Vsig8	0	0	0	1	0	1
mmu-miR-223-3p	241113	Prkag3	0	0	0	1	0	1
mmu-miR-223-3p	241118	Asic4	0	0	0	1	0	1
mmu-miR-223-3p	241128	Fam124b	0	0	0	1	0	1
mmu-miR-223-3p	241197	Serpinb10	0	0	0	1	0	1
mmu-miR-223-3p	241274	Pnpla7	1	0	0	0	0	1
mmu-miR-223-3p	241308	Ralgps1	1	0	0	0	0	1
mmu-miR-223-3p	241391	Galnt5	0	0	0	1	0	1
mmu-miR-223-3p	241452	Dhrs9	0	1	0	0	0	1
mmu-miR-223-3p	241520	Fam171b	0	0	0	1	0	1
mmu-miR-223-3p	241850	Abhd16b	0	0	0	1	0	1
mmu-miR-223-3p	241950	Bbs12	0	0	0	1	0	1
mmu-miR-223-3p	241989	Pabpc4l	0	0	0	1	0	1
mmu-miR-223-3p	242037	Gm410	1	0	0	0	0	1
mmu-miR-223-3p	242125	Mab21l3	0	0	0	1	0	1
mmu-miR-223-3p	242286	Sdr16c6	0	0	0	1	0	1
mmu-miR-223-3p	242291	Impad1	0	0	0	1	0	1
mmu-miR-223-3p	242297	Fam110b	0	1	0	0	0	1
mmu-miR-223-3p	242384	Lingo2	0	0	0	1	0	1
mmu-miR-223-3p	242519	Ifna12	0	0	1	0	0	1
mmu-miR-223-3p	242594	1700024P16Rik	0	0	0	1	0	1
mmu-miR-223-3p	242602	BC055111	0	0	0	1	0	1
mmu-miR-223-3p	242603	Cdcp2	0	0	0	1	0	1
mmu-miR-223-3p	242607	Slc1a7	0	0	0	1	0	1
mmu-miR-223-3p	242662	Rims3	0	1	0	0	0	1
mmu-miR-223-3p	242711	Gm13011	0	0	0	1	0	1
mmu-miR-223-3p	242735	Lrrc38	0	0	0	1	0	1
mmu-miR-223-3p	242736	Pramef8	0	0	0	1	0	1
mmu-miR-223-3p	242737	Oog4	0	0	0	1	0	1
mmu-miR-223-3p	242915	Gareml	0	0	0	1	0	1
mmu-miR-223-3p	242960	Fbxl5	0	0	0	1	0	1

mmu-miR-223-3p	243078	Tecrl	0	0	0	1	0	1
mmu-miR-223-3p	243168	Hsd17b13	0	0	0	1	0	1
mmu-miR-223-3p	243197	Mfsd7a	0	0	0	1	0	1
mmu-miR-223-3p	243277	Gpr133	0	0	0	1	0	1
mmu-miR-223-3p	243369	Sspo	1	0	0	0	0	1
mmu-miR-223-3p	243529	H1fx	0	0	0	1	0	1
mmu-miR-223-3p	243538	Ccdc37	0	0	0	1	0	1
mmu-miR-223-3p	243547	Grip2	0	0	0	1	0	1
mmu-miR-223-3p	243659	Styk1	0	0	0	1	0	1
mmu-miR-223-3p	243771	Parp12	0	0	0	1	0	1
mmu-miR-223-3p	243816	Gp6	0	0	0	1	0	1
mmu-miR-223-3p	243819	Ppp6r1	0	0	0	1	0	1
mmu-miR-223-3p	243822	Fam71e2	0	0	0	1	0	1
mmu-miR-223-3p	243846	Ccdc9	0	0	0	1	0	1
mmu-miR-223-3p	243862	Psg22	0	0	0	1	0	1
mmu-miR-223-3p	243874	Nlrp9b	0	0	0	1	0	1
mmu-miR-223-3p	243905	Zfp568	0	0	0	1	0	1
mmu-miR-223-3p	243906	Zfp14	0	0	0	1	0	1
mmu-miR-223-3p	243910	Nfkbid	0	0	0	1	0	1
mmu-miR-223-3p	243978	Mrgprx2	0	0	0	1	0	1
mmu-miR-223-3p	243979	Mrgprb2	1	0	0	0	0	1
mmu-miR-223-3p	243983	Zdhhc13	0	0	0	1	0	1
mmu-miR-223-3p	243996	4933405O20Rik	0	0	0	1	0	1
mmu-miR-223-3p	244144	Usp35	1	0	0	0	0	1
mmu-miR-223-3p	244179	Ubqlnl	0	0	0	1	0	1
mmu-miR-223-3p	244183	Trim30b	0	0	0	1	0	1
mmu-miR-223-3p	244216	Zfp771	0	0	0	1	0	1
mmu-miR-223-3p	244218	Ctf2	0	0	0	1	0	1
mmu-miR-223-3p	244219	Zfp668	0	0	0	1	0	1
mmu-miR-223-3p	244233	Cd163l1	0	0	0	1	0	1
mmu-miR-223-3p	244234	5830411N06Rik	0	0	0	1	0	1
mmu-miR-223-3p	244418	D8Ertd82e	1	0	0	0	0	1
mmu-miR-223-3p	244421	Lonrf1	0	0	0	1	0	1
mmu-miR-223-3p	244448	Triml1	0	0	0	1	0	1
mmu-miR-223-3p	244486	Adam29	0	0	0	1	0	1
mmu-miR-223-3p	244551	Nanos3	0	0	0	1	0	1
mmu-miR-223-3p	244631	Pskh1	0	0	0	1	0	1
mmu-miR-223-3p	244666	Sprtn	0	0	1	0	0	1
mmu-miR-223-3p	244668	Sipa1l2	0	0	0	1	0	1
mmu-miR-223-3p	244694	Kdm4d	0	0	0	1	0	1
mmu-miR-223-3p	244721	Zfp846	0	0	0	1	0	1
mmu-miR-223-3p	244810	AW551984	0	0	0	1	0	1
mmu-miR-223-3p	244853	Nxpe4	0	0	0	1	0	1
mmu-miR-223-3p	244882	Tnfaip8l3	1	0	0	0	0	1
mmu-miR-223-3p	244886	AI118078	0	0	0	1	0	1
mmu-miR-223-3p	244954	Prss35	1	0	0	0	0	1
mmu-miR-223-3p	244958	Mrap2	0	0	0	1	0	1
mmu-miR-223-3p	244962	Snx14	0	1	0	0	0	1
mmu-miR-223-3p	245020	Slc35g2	0	0	0	1	0	1
mmu-miR-223-3p	245050	Fam198a	0	0	0	1	0	1
mmu-miR-223-3p	245109	Zscan4c	0	0	0	1	0	1
mmu-miR-223-3p	245240	9930111J21Rik2	0	0	0	1	0	1
mmu-miR-223-3p	245263	Gm4981	0	0	0	1	0	1
mmu-miR-223-3p	245308	Zdhhc19	0	0	0	1	0	1
mmu-miR-223-3p	245347	Gm4984	0	0	0	1	0	1

mmu-miR-223-3p	245368	Zfp300	0	0	0	1	0	1
mmu-miR-223-3p	245381	Sowahd	0	0	1	0	0	1
mmu-miR-223-3p	245404	Dcaf12l1	0	0	0	1	0	1
mmu-miR-223-3p	245423	Gm364	0	0	0	1	0	1
mmu-miR-223-3p	245440	Gm4988	0	0	0	1	0	1
mmu-miR-223-3p	245450	Slitrk2	0	0	0	1	0	1
mmu-miR-223-3p	245468	Pnma3	0	0	0	1	0	1
mmu-miR-223-3p	245469	Pdzd4	0	1	0	0	0	1
mmu-miR-223-3p	245474	Dkc1	0	0	0	1	0	1
mmu-miR-223-3p	245509	4932429P05Rik	1	0	0	0	0	1
mmu-miR-223-3p	245532	Awat2	0	0	1	0	0	1
mmu-miR-223-3p	245566	Cypt2	1	0	0	0	0	1
mmu-miR-223-3p	245595	Zfp711	0	0	0	1	0	1
mmu-miR-223-3p	245643	Frmpd3	0	1	0	0	0	1
mmu-miR-223-3p	245666	Iqsec2	0	0	0	1	0	1
mmu-miR-223-3p	245670	Rragb	0	0	0	1	0	1
mmu-miR-223-3p	245827	Fat2	0	0	1	0	0	1
mmu-miR-223-3p	245828	Trappc1	1	0	0	0	0	1
mmu-miR-223-3p	245847	Amdhd2	0	0	0	1	0	1
mmu-miR-223-3p	245860	Atg9a	0	0	0	1	0	1
mmu-miR-223-3p	245866	Ift52	0	0	0	1	0	1
mmu-miR-223-3p	245867	Pcmtd2	0	0	0	1	0	1
mmu-miR-223-3p	245877	Map7d1	0	0	0	1	0	1
mmu-miR-223-3p	245884	Fam71f2	0	0	0	1	0	1
mmu-miR-223-3p	246048	Chodl	1	0	0	0	0	1
mmu-miR-223-3p	246049	Slc36a2	0	0	0	1	0	1
mmu-miR-223-3p	246079	Defb9	0	0	0	1	0	1
mmu-miR-223-3p	246081	Defb11	0	0	0	1	0	1
mmu-miR-223-3p	246085	Defb10	0	0	0	1	0	1
mmu-miR-223-3p	246102	Rttn	1	0	0	0	0	1
mmu-miR-223-3p	246154	Vasn	0	0	0	1	0	1
mmu-miR-223-3p	246177	Myo1g	0	1	0	0	0	1
mmu-miR-223-3p	246179	Fktn	0	0	0	1	0	1
mmu-miR-223-3p	246190	Otoa	0	0	0	1	0	1
mmu-miR-223-3p	246196	Zfp277	1	0	0	0	0	1
mmu-miR-223-3p	246221	Mpst	0	0	0	1	0	1
mmu-miR-223-3p	246256	Fcgr4	0	0	0	1	0	1
mmu-miR-223-3p	246277	Csad	0	0	0	1	0	1
mmu-miR-223-3p	246278	Cd207	0	0	0	1	0	1
mmu-miR-223-3p	246696	Slc25a28	0	0	0	1	0	1
mmu-miR-223-3p	246707	Emilin2	0	0	0	1	0	1
mmu-miR-223-3p	246709	Rgs13	0	0	0	1	0	1
mmu-miR-223-3p	246729	Oas1h	0	0	0	1	0	1
mmu-miR-223-3p	246730	Oas1a	0	0	0	1	0	1
mmu-miR-223-3p	246746	Cd300lf	0	0	0	1	0	1
mmu-miR-223-3p	246747	Adig	0	0	0	1	0	1
mmu-miR-223-3p	246787	Slc5a2	0	0	0	1	0	1
mmu-miR-223-3p	246788	Trpv3	0	1	0	0	0	1
mmu-miR-223-3p	246791	Obox3	0	0	0	1	0	1
mmu-miR-223-3p	246792	Obox2	0	0	0	1	0	1
mmu-miR-223-3p	252830	Obox6	0	0	0	1	0	1
mmu-miR-223-3p	252910	Vmn1r71	1	0	0	0	0	1
mmu-miR-223-3p	252967	Ropn1l	0	0	0	1	0	1
mmu-miR-223-3p	252974	Tspear	0	0	0	1	0	1
mmu-miR-223-3p	257630	Il17f	0	0	0	1	0	1

mmu-miR-223-3p	257633	Acsf3	0	0	0	1	0	1
mmu-miR-223-3p	257883	Olfr1357	0	0	0	1	0	1
mmu-miR-223-3p	257908	Olfr115	0	0	0	1	0	1
mmu-miR-223-3p	258096	Olfr112	1	0	0	0	0	1
mmu-miR-223-3p	258261	Olfr325	0	0	0	1	0	1
mmu-miR-223-3p	258287	Olfr125	1	0	0	0	0	1
mmu-miR-223-3p	258311	Olfr601	0	0	0	1	0	1
mmu-miR-223-3p	258314	Olfr725	0	0	0	1	0	1
mmu-miR-223-3p	258462	Olfr1392	0	0	0	1	0	1
mmu-miR-223-3p	258494	Olfr318	0	0	0	1	0	1
mmu-miR-223-3p	258529	Olfr313	0	0	0	1	0	1
mmu-miR-223-3p	258605	Olfr968	0	0	0	1	0	1
mmu-miR-223-3p	258622	Olfr121	0	0	0	1	0	1
mmu-miR-223-3p	258638	Olfr1163	0	0	0	1	0	1
mmu-miR-223-3p	258644	Olfr1166	0	0	0	1	0	1
mmu-miR-223-3p	258692	Olfr1442	0	0	0	1	0	1
mmu-miR-223-3p	258706	Olfr43	0	0	0	1	0	1
mmu-miR-223-3p	258744	Olfr875	0	0	0	1	0	1
mmu-miR-223-3p	258750	Olfr551	0	0	0	1	0	1
mmu-miR-223-3p	258777	Olfr922	0	0	0	1	0	1
mmu-miR-223-3p	258805	Olfr1426	0	0	0	1	0	1
mmu-miR-223-3p	258809	Olfr651	0	0	0	1	0	1
mmu-miR-223-3p	258879	Olfr330	0	0	0	1	0	1
mmu-miR-223-3p	258881	Olfr1404	0	0	0	1	0	1
mmu-miR-223-3p	258883	Olfr876	0	0	0	1	0	1
mmu-miR-223-3p	258893	Olfr1225	0	0	0	1	0	1
mmu-miR-223-3p	258902	Olfr1220	0	0	0	1	0	1
mmu-miR-223-3p	258903	Olfr1217	0	0	0	1	0	1
mmu-miR-223-3p	258905	Olfr871	0	0	0	1	0	1
mmu-miR-223-3p	259001	Olfr181	0	0	0	1	0	1
mmu-miR-223-3p	259102	Olfr630	0	0	0	1	0	1
mmu-miR-223-3p	259112	Olfr979	0	0	0	1	0	1
mmu-miR-223-3p	259144	Olfr456	0	0	0	1	0	1
mmu-miR-223-3p	259148	Olfr329-ps	0	0	0	1	0	1
mmu-miR-223-3p	259163	Olfr1354	0	0	0	1	0	1
mmu-miR-223-3p	259300	Ehd2	0	0	0	1	0	1
mmu-miR-223-3p	259301	Leap2	0	0	0	1	0	1
mmu-miR-223-3p	260296	Trim61	0	0	0	1	0	1
mmu-miR-223-3p	260409	Cdc42ep3	0	0	0	1	0	1
mmu-miR-223-3p	264895	Acsf2	0	0	1	0	0	1
mmu-miR-223-3p	266614	Ly6g5b	0	0	0	1	0	1
mmu-miR-223-3p	266620	Defb36	0	0	0	1	0	1
mmu-miR-223-3p	266632	Irak4	0	0	0	1	0	1
mmu-miR-223-3p	266781	Snx17	0	0	0	1	0	1
mmu-miR-223-3p	268291	Rnf217	0	0	0	1	0	1
mmu-miR-223-3p	268448	Phf12	0	0	0	1	0	1
mmu-miR-223-3p	268449	Rpl23a	1	0	0	0	0	1
mmu-miR-223-3p	268451	Rab11fip4	0	0	0	1	0	1
mmu-miR-223-3p	268465	Eme1	0	0	0	1	0	1
mmu-miR-223-3p	268469	Zfp652	0	0	0	1	0	1
mmu-miR-223-3p	268470	Ube2z	0	0	0	1	0	1
mmu-miR-223-3p	268482	Krt12	0	0	0	1	0	1
mmu-miR-223-3p	268510	Mgat5b	0	0	0	1	0	1
mmu-miR-223-3p	268512	Slc26a11	0	0	0	1	0	1
mmu-miR-223-3p	268515	Bahcc1	0	0	0	1	0	1

mmu-miR-223-3p	268534	Sntg2	0	0	0	1	0	1
mmu-miR-223-3p	268567	Tmem229b	0	0	0	1	0	1
mmu-miR-223-3p	268591	Serpina5	0	0	1	0	0	1
mmu-miR-223-3p	268595	D430019H16Rik	0	0	0	1	0	1
mmu-miR-223-3p	268709	Fam107a	0	0	0	1	0	1
mmu-miR-223-3p	268721	Zswim8	0	0	0	1	0	1
mmu-miR-223-3p	268729	Gm626	0	0	0	1	0	1
mmu-miR-223-3p	268747	Lrrc16b	1	0	0	0	0	1
mmu-miR-223-3p	268752	Wdfy2	0	0	0	1	0	1
mmu-miR-223-3p	268756	Gulo	0	0	0	1	0	1
mmu-miR-223-3p	268759	9930012K11Rik	0	0	0	1	0	1
mmu-miR-223-3p	268780	Egflam	0	0	0	1	0	1
mmu-miR-223-3p	268807	Klhl38	1	0	0	0	0	1
mmu-miR-223-3p	268816	Mroh5	0	0	0	1	0	1
mmu-miR-223-3p	268857	Nlrc3	0	0	0	1	0	1
mmu-miR-223-3p	268890	Lsamp	0	0	0	1	0	1
mmu-miR-223-3p	268903	Nrip1	0	1	0	0	0	1
mmu-miR-223-3p	268905	Krtap13-1	0	0	0	1	0	1
mmu-miR-223-3p	268933	Wdr24	0	0	0	1	0	1
mmu-miR-223-3p	268935	Scube3	0	1	0	0	0	1
mmu-miR-223-3p	268958	Capn11	0	0	0	1	0	1
mmu-miR-223-3p	268970	Arhgap28	0	0	0	1	0	1
mmu-miR-223-3p	268977	Ltbp1	0	0	0	1	0	1
mmu-miR-223-3p	269061	Cpsf7	0	0	0	1	0	1
mmu-miR-223-3p	269113	Nup54	1	0	0	0	0	1
mmu-miR-223-3p	269116	Nfasc	0	0	0	1	0	1
mmu-miR-223-3p	269120	Optc	0	0	0	1	0	1
mmu-miR-223-3p	269152	Kif26b	0	0	0	1	0	1
mmu-miR-223-3p	269336	Ccdc32	0	0	0	1	0	1
mmu-miR-223-3p	269338	Vps39	0	0	0	1	0	1
mmu-miR-223-3p	269346	Slc28a2	0	0	0	1	0	1
mmu-miR-223-3p	269356	Slc4a11	0	0	0	1	0	1
mmu-miR-223-3p	269378	Ahcy	0	0	0	1	0	1
mmu-miR-223-3p	269400	Rtel1	0	0	0	1	0	1
mmu-miR-223-3p	269423	3110057O12Rik	1	0	0	0	0	1
mmu-miR-223-3p	269470	Wdr3	1	0	0	0	0	1
mmu-miR-223-3p	269513	Nkain3	0	0	0	1	0	1
mmu-miR-223-3p	269523	Vcp	0	0	0	1	0	1
mmu-miR-223-3p	269529	Fbxo10	0	0	0	1	0	1
mmu-miR-223-3p	269536	Tex10	0	1	0	0	0	1
mmu-miR-223-3p	269582	Clspn	0	0	0	1	0	1
mmu-miR-223-3p	269643	Ppp2r2c	0	0	0	1	0	1
mmu-miR-223-3p	269693	Ccdc60	0	0	0	1	0	1
mmu-miR-223-3p	269700	Gm15800	0	1	0	0	0	1
mmu-miR-223-3p	269701	Wdr66	0	0	0	1	0	1
mmu-miR-223-3p	269823	Pon3	0	0	0	1	0	1
mmu-miR-223-3p	269854	Nat14	0	0	0	1	0	1
mmu-miR-223-3p	269951	Idh2	0	1	0	0	0	1
mmu-miR-223-3p	269959	Adamtsl3	0	0	0	1	0	1
mmu-miR-223-3p	269966	Nup98	0	0	0	1	0	1
mmu-miR-223-3p	270004	Foxi2	0	0	0	1	0	1
mmu-miR-223-3p	270049	Galntl6	0	0	0	1	0	1
mmu-miR-223-3p	270076	Gcdh	0	0	0	1	0	1
mmu-miR-223-3p	270091	Lrrc36	0	0	0	1	0	1
mmu-miR-223-3p	270109	Pcnxl2	0	0	0	1	0	1

mmu-miR-223-3p	270151	Nlrx1	0	0	0	1	0	1
mmu-miR-223-3p	270162	Elmod1	1	0	0	0	0	1
mmu-miR-223-3p	270166	Clpx	0	0	0	1	0	1
mmu-miR-223-3p	270190	Ephb1	0	0	0	1	0	1
mmu-miR-223-3p	270599	Gm648	0	0	0	1	0	1
mmu-miR-223-3p	270669	Mbtps2	0	0	0	1	0	1
mmu-miR-223-3p	270672	Map3k15	1	0	0	0	0	1
mmu-miR-223-3p	270685	Mthfd1l	1	0	0	0	0	1
mmu-miR-223-3p	271127	Adamts16	0	0	0	1	0	1
mmu-miR-223-3p	271209	Rp1l1	0	0	0	1	0	1
mmu-miR-223-3p	271278	BC024139	0	0	0	1	0	1
mmu-miR-223-3p	271377	Zbtb11	0	0	0	1	0	1
mmu-miR-223-3p	271424	Ip6k3	0	0	0	1	0	1
mmu-miR-223-3p	271508	4933408B17Rik	0	0	0	1	0	1
mmu-miR-223-3p	271711	Tmem169	0	0	0	1	0	1
mmu-miR-223-3p	271813	Agbl2	0	0	1	0	0	1
mmu-miR-223-3p	271944	C2cd4d	0	0	0	1	0	1
mmu-miR-223-3p	271970	Arsj	0	0	0	1	0	1
mmu-miR-223-3p	271981	Tbck	0	0	0	1	0	1
mmu-miR-223-3p	272009	Srsf12	0	0	0	1	0	1
mmu-miR-223-3p	272322	Arntl2	0	1	0	0	0	1
mmu-miR-223-3p	272347	Zfp398	0	0	0	1	0	1
mmu-miR-223-3p	272411	B3gnt6	0	0	0	1	0	1
mmu-miR-223-3p	272538	Tango6	0	0	0	1	0	1
mmu-miR-223-3p	272551	Gins2	0	0	0	1	0	1
mmu-miR-223-3p	272589	Tbcel	0	0	0	1	0	1
mmu-miR-223-3p	272636	Esyt3	1	0	0	0	0	1
mmu-miR-223-3p	272643	Prss43	0	0	0	1	0	1
mmu-miR-223-3p	276770	Eif5a	1	0	0	0	0	1
mmu-miR-223-3p	276829	Smtnl2	0	0	0	1	0	1
mmu-miR-223-3p	276846	Pigs	0	0	0	1	0	1
mmu-miR-223-3p	276891	Timd4	0	0	0	1	0	1
mmu-miR-223-3p	276905	Armc7	0	0	0	1	0	1
mmu-miR-223-3p	276920	Ccdc42	0	0	0	1	0	1
mmu-miR-223-3p	277010	Marveld1	0	0	0	1	0	1
mmu-miR-223-3p	277203	Tm4sf19	0	0	0	1	0	1
mmu-miR-223-3p	277328	Trpa1	0	0	0	1	0	1
mmu-miR-223-3p	277343	Wfdc8	0	0	0	1	0	1
mmu-miR-223-3p	277345	Wfdc16	0	0	0	1	0	1
mmu-miR-223-3p	277353	Tcfl5	0	0	0	1	0	1
mmu-miR-223-3p	277396	Klhl23	0	0	0	1	0	1
mmu-miR-223-3p	277463	Gpr107	0	0	0	1	0	1
mmu-miR-223-3p	277668	Gm13088	0	0	0	1	0	1
mmu-miR-223-3p	277744	Gm694	0	0	0	1	0	1
mmu-miR-223-3p	277753	Cyp4a12a	0	0	0	1	0	1
mmu-miR-223-3p	277939	C2cd3	0	0	0	1	0	1
mmu-miR-223-3p	278672	Duxbl1	0	0	0	1	0	1
mmu-miR-223-3p	278795	Lrrc10b	0	0	0	1	0	1
mmu-miR-223-3p	279499	Kctd19	0	0	0	1	0	1
mmu-miR-223-3p	279766	Rhbdd3	0	0	0	1	0	1
mmu-miR-223-3p	280411	Lix1l	0	0	0	1	0	1
mmu-miR-223-3p	280667	Adam1b	0	0	0	1	0	1
mmu-miR-223-3p	286942	Kif19a	0	0	0	1	0	1
mmu-miR-223-3p	317677	Gm5077	0	0	0	1	0	1
mmu-miR-223-3p	317750	Slc24a5	1	0	0	0	0	1

mmu-miR-223-3p	319146	Ifnz	0	0	0	1	0	1
mmu-miR-223-3p	319158	Hist1h4i	0	0	0	1	0	1
mmu-miR-223-3p	319164	Hist1h2ac	0	0	0	1	0	1
mmu-miR-223-3p	319167	Hist1h2ag	0	0	0	1	0	1
mmu-miR-223-3p	319179	Hist1h2be	0	0	0	1	0	1
mmu-miR-223-3p	319189	Hist2h2bb	0	0	0	1	0	1
mmu-miR-223-3p	319197	Gpr4	0	0	0	1	0	1
mmu-miR-223-3p	319236	Trim12c	0	0	0	1	0	1
mmu-miR-223-3p	319262	Fchsd1	0	0	0	1	0	1
mmu-miR-223-3p	319266	A130010J15Rik	0	0	0	1	0	1
mmu-miR-223-3p	319277	A230046K03Rik	0	0	0	1	0	1
mmu-miR-223-3p	319352	Pianp	0	0	0	1	0	1
mmu-miR-223-3p	319370	Ubald2	0	0	0	1	0	1
mmu-miR-223-3p	319430	C5ar2	0	0	0	1	0	1
mmu-miR-223-3p	319448	Fndc3a	0	0	0	1	0	1
mmu-miR-223-3p	319493	A430078G23Rik	0	0	0	1	0	1
mmu-miR-223-3p	319506	7530428D23Rik	0	0	0	1	0	1
mmu-miR-223-3p	319518	Pdpr	0	1	0	0	0	1
mmu-miR-223-3p	319530	Zfp750	0	0	0	1	0	1
mmu-miR-223-3p	319554	Idi1	0	0	0	1	0	1
mmu-miR-223-3p	319581	Xkr5	0	0	0	1	0	1
mmu-miR-223-3p	319582	6430573F11Rik	0	0	0	1	0	1
mmu-miR-223-3p	319583	Lig4	0	0	0	1	0	1
mmu-miR-223-3p	319613	Sybu	0	0	0	1	0	1
mmu-miR-223-3p	319618	Dcp1b	0	0	0	1	0	1
mmu-miR-223-3p	319634	Efcab5	0	0	0	1	0	1
mmu-miR-223-3p	319660	Agmo	0	0	0	1	0	1
mmu-miR-223-3p	319675	5830418K08Rik	1	0	0	0	0	1
mmu-miR-223-3p	319701	Fbxo48	0	0	0	1	0	1
mmu-miR-223-3p	319740	Zfyve27	0	0	0	1	0	1
mmu-miR-223-3p	319742	Mpzl3	0	0	0	1	0	1
mmu-miR-223-3p	319757	Smo	0	0	0	1	0	1
mmu-miR-223-3p	319823	F630003A18Rik	0	0	0	1	0	1
mmu-miR-223-3p	319845	Bbs9	0	0	0	1	0	1
mmu-miR-223-3p	319848	Slc17a4	0	0	0	1	0	1
mmu-miR-223-3p	319875	Tmprss11bnl	0	0	0	1	0	1
mmu-miR-223-3p	319876	Cobll1	0	0	0	1	0	1
mmu-miR-223-3p	319887	E030030I06Rik	0	0	0	1	0	1
mmu-miR-223-3p	319899	Dock6	0	0	0	1	0	1
mmu-miR-223-3p	319934	Sbf2	1	0	0	0	0	1
mmu-miR-223-3p	319942	A530016L24Rik	1	0	0	0	0	1
mmu-miR-223-3p	319953	Ttll1	0	0	0	1	0	1
mmu-miR-223-3p	319984	Jph4	0	0	0	1	0	1
mmu-miR-223-3p	319998	Tmem198	0	0	0	1	0	1
mmu-miR-223-3p	320007	Sidt1	0	0	0	1	0	1
mmu-miR-223-3p	320022	Ccdc79	0	1	0	0	0	1
mmu-miR-223-3p	320051	Exph5	0	0	0	1	0	1
mmu-miR-223-3p	320078	Olfml2b	1	0	0	0	0	1
mmu-miR-223-3p	320106	Slc38a11	0	0	0	1	0	1
mmu-miR-223-3p	320118	Fbxl13	0	0	0	1	0	1
mmu-miR-223-3p	320165	Tacc1	0	0	0	1	0	1
mmu-miR-223-3p	320202	Lefty2	0	0	1	0	0	1
mmu-miR-223-3p	320225	Catsperg1	0	0	0	1	0	1
mmu-miR-223-3p	320234	Ccdc66	1	0	0	0	0	1
mmu-miR-223-3p	320253	Marhc3	0	0	0	1	0	1

mmu-miR-223-3p	320265	Fam19a1	0	0	0	1	0	1
mmu-miR-223-3p	320277	Spef2	1	0	0	0	0	1
mmu-miR-223-3p	320292	Rasgef1b	0	0	0	1	0	1
mmu-miR-223-3p	320299	Iqcb1	0	0	0	1	0	1
mmu-miR-223-3p	320302	Glt28d2	0	0	0	1	0	1
mmu-miR-223-3p	320376	Bcorl1	0	0	0	1	0	1
mmu-miR-223-3p	320405	Cadps2	0	0	0	1	0	1
mmu-miR-223-3p	320429	Trank1	0	0	0	1	0	1
mmu-miR-223-3p	320452	P4ha3	0	0	0	1	0	1
mmu-miR-223-3p	320454	Tmprss11g	0	0	0	1	0	1
mmu-miR-223-3p	320473	Heatr5b	1	0	0	0	0	1
mmu-miR-223-3p	320484	Rasal3	0	0	0	1	0	1
mmu-miR-223-3p	320506	Lmbrd2	0	0	0	1	0	1
mmu-miR-223-3p	320554	Tcp11l1	0	0	0	1	0	1
mmu-miR-223-3p	320557	Fam169a	0	0	0	1	0	1
mmu-miR-223-3p	320558	Sycp2	0	0	1	0	0	1
mmu-miR-223-3p	320581	Idi2	0	0	0	1	0	1
mmu-miR-223-3p	320587	Tmem88b	0	0	0	1	0	1
mmu-miR-223-3p	320590	Svopl	1	0	0	0	0	1
mmu-miR-223-3p	320604	Ccdc169	0	0	1	0	0	1
mmu-miR-223-3p	320631	Abca15	0	0	0	1	0	1
mmu-miR-223-3p	320685	Dctd	0	0	0	1	0	1
mmu-miR-223-3p	320701	Fam19a4	0	0	0	1	0	1
mmu-miR-223-3p	320705	Bend6	0	0	0	1	0	1
mmu-miR-223-3p	320712	Abi3bp	0	0	0	1	0	1
mmu-miR-223-3p	320720	Fastkd1	0	0	0	1	0	1
mmu-miR-223-3p	320752	Dpy19l2	0	0	0	1	0	1
mmu-miR-223-3p	320784	C230072F16Rik	0	0	0	1	0	1
mmu-miR-223-3p	320795	Pkn1	0	0	0	1	0	1
mmu-miR-223-3p	320816	Ankrd16	1	0	0	0	0	1
mmu-miR-223-3p	320858	L3mbtl4	0	0	0	1	0	1
mmu-miR-223-3p	320873	Cdh10	0	0	0	1	0	1
mmu-miR-223-3p	320923	Mtap7d3	0	0	0	1	0	1
mmu-miR-223-3p	320997	Cyp4f39	0	0	0	1	0	1
mmu-miR-223-3p	321019	Gpr183	0	0	0	1	0	1
mmu-miR-223-3p	326618	Tpm4	1	0	0	0	0	1
mmu-miR-223-3p	327799	Usp44	0	0	0	1	0	1
mmu-miR-223-3p	327900	Ubtd2	0	0	0	1	0	1
mmu-miR-223-3p	327954	Dnah2	0	0	0	1	0	1
mmu-miR-223-3p	327959	Xaf1	1	0	0	0	0	1
mmu-miR-223-3p	328162	Trmt61a	0	0	0	1	0	1
mmu-miR-223-3p	328231	Gm5082	0	0	0	1	0	1
mmu-miR-223-3p	328234	Rnf182	0	0	0	1	0	1
mmu-miR-223-3p	328258	Slc25a48	0	0	0	1	0	1
mmu-miR-223-3p	328329	Mast4	0	0	0	1	0	1
mmu-miR-223-3p	328381	Sh2d4b	0	1	0	0	0	1
mmu-miR-223-3p	328417	Parp4	0	0	0	1	0	1
mmu-miR-223-3p	328424	Kcnrg	0	0	0	1	0	1
mmu-miR-223-3p	328531	9330182O14Rik	0	0	0	1	0	1
mmu-miR-223-3p	328563	Apol11b	0	0	0	1	0	1
mmu-miR-223-3p	328572	Ep300	0	0	0	1	0	1
mmu-miR-223-3p	328573	4930407I10Rik	0	0	0	1	0	1
mmu-miR-223-3p	328580	Tubgcp6	0	0	0	1	0	1
mmu-miR-223-3p	328695	Gm813	0	0	0	1	0	1
mmu-miR-223-3p	328699	Gabrr3	0	0	0	1	0	1

mmu-miR-223-3p	328778	Rab26	0	0	0	1	0	1
mmu-miR-223-3p	328779	Hs3st6	0	0	0	1	0	1
mmu-miR-223-3p	328783	Mslnl	0	0	0	1	0	1
mmu-miR-223-3p	328789	Lhfpl5	0	0	0	1	0	1
mmu-miR-223-3p	328795	Ubash3a	0	0	0	1	0	1
mmu-miR-223-3p	328830	A530064D06Rik	0	0	0	1	0	1
mmu-miR-223-3p	328971	Spink10	0	0	0	1	0	1
mmu-miR-223-3p	329055	Gm5097	0	0	0	1	0	1
mmu-miR-223-3p	329064	Pkd2l1	0	0	0	1	0	1
mmu-miR-223-3p	329065	Scd4	0	0	0	1	0	1
mmu-miR-223-3p	329251	Ppp1r12b	0	1	0	0	0	1
mmu-miR-223-3p	329375	1700101E01Rik	0	0	0	1	0	1
mmu-miR-223-3p	329436	Gm14461	0	0	0	1	0	1
mmu-miR-223-3p	329502	Pla2g4e	0	0	0	1	0	1
mmu-miR-223-3p	329504	Lcmt2	0	0	0	1	0	1
mmu-miR-223-3p	329554	Gm826	0	0	0	1	0	1
mmu-miR-223-3p	329557	Svs3b	0	0	0	1	0	1
mmu-miR-223-3p	329559	Zfp335	0	0	0	1	0	1
mmu-miR-223-3p	329575	Gm14325	0	0	0	1	0	1
mmu-miR-223-3p	329650	Med12l	0	0	0	1	0	1
mmu-miR-223-3p	329716	BC107364	0	0	0	1	0	1
mmu-miR-223-3p	329777	Pigk	0	0	0	1	0	1
mmu-miR-223-3p	329795	Tmem67	0	1	0	0	0	1
mmu-miR-223-3p	329831	Fam166b	0	0	0	1	0	1
mmu-miR-223-3p	329877	Dennd4c	1	0	0	0	0	1
mmu-miR-223-3p	329908	Usp24	0	0	0	1	0	1
mmu-miR-223-3p	329909	Tmem61	0	0	0	1	0	1
mmu-miR-223-3p	329919	Skint2	0	0	0	1	0	1
mmu-miR-223-3p	329934	Foxo6	0	0	0	1	0	1
mmu-miR-223-3p	329984	Gm13109	0	0	0	1	0	1
mmu-miR-223-3p	329986	BC080695	0	0	0	1	0	1
mmu-miR-223-3p	330050	Fam185a	0	0	0	1	0	1
mmu-miR-223-3p	330122	Cxcl3	1	0	0	0	0	1
mmu-miR-223-3p	330149	Hfm1	0	0	0	1	0	1
mmu-miR-223-3p	330171	Kctd10	0	0	0	1	0	1
mmu-miR-223-3p	330189	Tmem120b	0	0	0	1	0	1
mmu-miR-223-3p	330192	Vps37b	0	0	0	1	0	1
mmu-miR-223-3p	330216	Mblac1	0	0	0	1	0	1
mmu-miR-223-3p	330305	Gm5111	0	0	0	1	0	1
mmu-miR-223-3p	330440	Gm766	0	0	0	1	0	1
mmu-miR-223-3p	330485	Tmem145	0	0	0	1	0	1
mmu-miR-223-3p	330503	Gm5113	0	0	0	1	0	1
mmu-miR-223-3p	330513	Gm5114	0	0	0	1	0	1
mmu-miR-223-3p	330657	Prss53	1	0	0	0	0	1
mmu-miR-223-3p	330660	Btbd16	0	0	0	1	0	1
mmu-miR-223-3p	330695	Ctxn1	0	0	0	1	0	1
mmu-miR-223-3p	330938	Dixdc1	0	0	0	1	0	1
mmu-miR-223-3p	331004	Slc9a9	0	0	0	1	0	1
mmu-miR-223-3p	331026	Gmppb	0	0	0	1	0	1
mmu-miR-223-3p	331046	Tgm4	0	0	0	1	0	1
mmu-miR-223-3p	331063	Gsdmc2	0	0	0	1	0	1
mmu-miR-223-3p	331195	A430089I19Rik	0	1	0	0	0	1
mmu-miR-223-3p	331461	Il1rapl1	0	1	0	0	0	1
mmu-miR-223-3p	331524	Xkrx	0	0	0	1	0	1
mmu-miR-223-3p	331535	Serpina7	0	0	0	1	0	1

mmu-miR-223-3p	332309	Grxcr2	0	0	0	1	0	1
mmu-miR-223-3p	332359	Tigd3	0	0	0	1	0	1
mmu-miR-223-3p	332396	Kcnk18	0	0	0	1	0	1
mmu-miR-223-3p	332713	BC051628	0	0	0	1	0	1
mmu-miR-223-3p	332923	Gm12794	0	0	0	1	0	1
mmu-miR-223-3p	332934	Zmynd12	0	0	0	1	0	1
mmu-miR-223-3p	332942	Gm853	0	0	0	1	0	1
mmu-miR-223-3p	333050	Ksr2	0	0	0	1	0	1
mmu-miR-223-3p	333193	BC053749	0	0	0	1	0	1
mmu-miR-223-3p	333307	Trim75	0	0	0	1	0	1
mmu-miR-223-3p	333424	A4gnt	1	0	0	0	0	1
mmu-miR-223-3p	333605	Frmpd4	0	0	0	1	0	1
mmu-miR-223-3p	333639	Mamld1	0	0	0	1	0	1
mmu-miR-223-3p	333654	Ppp1r13l	0	0	0	1	0	1
mmu-miR-223-3p	337924	Cyp3a44	0	0	0	1	0	1
mmu-miR-223-3p	338346	Gpr21	0	0	0	1	0	1
mmu-miR-223-3p	338350	Acad12	0	0	0	1	0	1
mmu-miR-223-3p	338355	Fkbp15	1	0	0	0	0	1
mmu-miR-223-3p	338365	Slc41a2	0	0	0	1	0	1
mmu-miR-223-3p	353169	Slc2a12	0	0	0	1	0	1
mmu-miR-223-3p	353187	Nr1d2	0	0	0	1	0	1
mmu-miR-223-3p	353208	Zfp931	0	0	0	1	0	1
mmu-miR-223-3p	353282	Sfmbt2	0	0	0	1	0	1
mmu-miR-223-3p	353310	Zfp703	0	0	0	1	0	1
mmu-miR-223-3p	353326	Rtl1	0	1	0	0	0	1
mmu-miR-223-3p	353344	Opn5	0	0	0	1	0	1
mmu-miR-223-3p	353346	Gpr141	0	0	0	1	0	1
mmu-miR-223-3p	360013	Myo18a	0	0	0	1	0	1
mmu-miR-223-3p	360220	Speer4d	0	0	0	1	0	1
mmu-miR-223-3p	379043	Raet1e	0	0	0	1	0	1
mmu-miR-223-3p	380656	A230066D03Rik	0	0	0	1	0	1
mmu-miR-223-3p	380660	Acss3	0	0	0	1	0	1
mmu-miR-223-3p	380664	Lemd3	0	0	0	1	0	1
mmu-miR-223-3p	380698	Obscn	0	0	0	1	0	1
mmu-miR-223-3p	380709	Spata22	0	1	0	0	0	1
mmu-miR-223-3p	380713	Scarf1	0	0	0	1	0	1
mmu-miR-223-3p	380714	Rph3al	0	0	0	1	0	1
mmu-miR-223-3p	380728	Kcnh4	0	0	0	1	0	1
mmu-miR-223-3p	380753	Atxn7l1	0	0	0	1	0	1
mmu-miR-223-3p	380755	Lsmem1	0	0	0	1	0	1
mmu-miR-223-3p	380768	Ccdc177	0	0	0	1	0	1
mmu-miR-223-3p	380785	Begain	1	0	0	0	0	1
mmu-miR-223-3p	380787	A230065H16Rik	0	0	0	1	0	1
mmu-miR-223-3p	380836	Mrs2	0	0	0	1	0	1
mmu-miR-223-3p	380842	Stmnd1	1	0	0	0	0	1
mmu-miR-223-3p	380845	Gm904	0	0	0	1	0	1
mmu-miR-223-3p	380850	Gm5141	0	0	0	1	0	1
mmu-miR-223-3p	380855	Rsl1	0	0	0	1	0	1
mmu-miR-223-3p	380920	Gm1587	0	0	0	1	0	1
mmu-miR-223-3p	380921	Dgkh	0	0	0	1	0	1
mmu-miR-223-3p	380928	Lmo7	0	0	0	1	0	1
mmu-miR-223-3p	380967	Tmem106c	0	0	0	1	0	1
mmu-miR-223-3p	380969	Nckap5l	0	0	0	1	0	1
mmu-miR-223-3p	380993	Zfat	0	0	0	1	0	1
mmu-miR-223-3p	380994	Gm20736	0	0	0	1	0	1

mmu-miR-223-3p	381043	Gm933	0	0	0	1	0	1
mmu-miR-223-3p	381045	Ccdc58	0	0	0	1	0	1
mmu-miR-223-3p	381058	Unc93a	0	0	0	1	0	1
mmu-miR-223-3p	381062	Ermard	0	0	0	1	0	1
mmu-miR-223-3p	381085	Tbc1d22b	0	0	0	1	0	1
mmu-miR-223-3p	381091	H2-Eb2	0	0	0	1	0	1
mmu-miR-223-3p	381107	Tmem232	0	0	0	1	0	1
mmu-miR-223-3p	381122	Capn13	0	0	0	1	0	1
mmu-miR-223-3p	381217	Fam189a2	0	0	0	1	0	1
mmu-miR-223-3p	381229	Ccdc147	0	0	0	1	0	1
mmu-miR-223-3p	381246	Xkr9	0	0	0	1	0	1
mmu-miR-223-3p	381260	Gm973	0	0	0	1	0	1
mmu-miR-223-3p	381284	E030010N08Rik	0	0	0	1	0	1
mmu-miR-223-3p	381308	Mnda	0	0	0	1	0	1
mmu-miR-223-3p	381314	Iars2	0	0	1	0	0	1
mmu-miR-223-3p	381337	Fam178b	0	0	0	1	0	1
mmu-miR-223-3p	381356	Cacfd1	0	0	0	1	0	1
mmu-miR-223-3p	381359	Prdm12	0	0	0	1	0	1
mmu-miR-223-3p	381393	4921509C19Rik	0	0	0	1	0	1
mmu-miR-223-3p	381404	Pabpc1l	0	0	0	1	0	1
mmu-miR-223-3p	381405	Zfp663	0	0	0	1	0	1
mmu-miR-223-3p	381409	Cdh26	0	0	0	1	0	1
mmu-miR-223-3p	381413	Gpr176	0	0	0	1	0	1
mmu-miR-223-3p	381418	Ctxn2	0	0	0	1	0	1
mmu-miR-223-3p	381463	Nr1h5	0	0	0	1	0	1
mmu-miR-223-3p	381476	Stpg2	0	0	0	1	0	1
mmu-miR-223-3p	381531	Mup21	0	0	0	1	0	1
mmu-miR-223-3p	381534	Ube2u	0	0	0	1	0	1
mmu-miR-223-3p	381544	Gm1661	1	0	0	0	0	1
mmu-miR-223-3p	381549	Zfp69	0	0	0	1	0	1
mmu-miR-223-3p	381570	Oog2	0	0	0	1	0	1
mmu-miR-223-3p	381580	Ccdc27	0	0	0	1	0	1
mmu-miR-223-3p	381590	C87499	0	0	0	1	0	1
mmu-miR-223-3p	381605	Tbc1d2	0	0	0	1	0	1
mmu-miR-223-3p	381629	Atraid	0	0	0	1	0	1
mmu-miR-223-3p	381654	C87414	0	0	0	1	0	1
mmu-miR-223-3p	381667	Lrcol1	1	0	0	0	0	1
mmu-miR-223-3p	381668	Fbrsl1	0	0	0	1	0	1
mmu-miR-223-3p	381673	A330070K13Rik	0	0	0	1	0	1
mmu-miR-223-3p	381678	Zcwpw1	0	0	0	1	0	1
mmu-miR-223-3p	381724	BC061212	0	1	0	0	0	1
mmu-miR-223-3p	381760	Ssbp1	0	0	0	1	0	1
mmu-miR-223-3p	381801	Tatdn2	0	0	0	1	0	1
mmu-miR-223-3p	381802	Tsen2	0	0	0	1	0	1
mmu-miR-223-3p	381809	Clec4b2	0	0	0	1	0	1
mmu-miR-223-3p	381832	Prpmp5	0	0	0	1	0	1
mmu-miR-223-3p	381852	Gm5155	0	0	0	1	0	1
mmu-miR-223-3p	381884	Slc6a16	0	0	0	1	0	1
mmu-miR-223-3p	381921	Taok2	0	0	0	1	0	1
mmu-miR-223-3p	381924	Itgad	0	0	0	1	0	1
mmu-miR-223-3p	381933	6430531B16Rik	0	0	0	1	0	1
mmu-miR-223-3p	381959	Gm1096	0	0	0	1	0	1
mmu-miR-223-3p	381994	E030018B13Rik	0	0	0	1	0	1
mmu-miR-223-3p	382014	Ano8	0	0	0	1	0	1
mmu-miR-223-3p	382038	Urb2	0	0	0	1	0	1

mmu-miR-223-3p	382062	AB124611	0	0	0	1	0	1
mmu-miR-223-3p	382074	Foxr1	0	0	0	1	0	1
mmu-miR-223-3p	382075	Odf3l1	0	0	0	1	0	1
mmu-miR-223-3p	382089	Ripply2	0	0	0	1	0	1
mmu-miR-223-3p	382097	Gm1123	0	0	0	1	0	1
mmu-miR-223-3p	382105	Fbxw15	1	0	0	0	0	1
mmu-miR-223-3p	382106	Fbxw24	1	0	0	0	0	1
mmu-miR-223-3p	382113	Slc22a14	0	0	0	1	0	1
mmu-miR-223-3p	382131	Gm20737	0	0	0	1	0	1
mmu-miR-223-3p	382156	Fbxw22	0	0	0	1	0	1
mmu-miR-223-3p	382207	Phf16	0	1	0	0	0	1
mmu-miR-223-3p	382217	Gm1140	0	0	0	1	0	1
mmu-miR-223-3p	382243	Gm10439	0	0	0	1	0	1
mmu-miR-223-3p	382244	Gm15091	0	0	0	1	0	1
mmu-miR-223-3p	382252	A830080D01Rik	0	0	0	1	0	1
mmu-miR-223-3p	382275	Gm5168	0	0	0	1	0	1
mmu-miR-223-3p	382277	Gm5169	0	0	0	1	0	1
mmu-miR-223-3p	382301	Sly	0	0	0	1	0	1
mmu-miR-223-3p	382639	Zbtb42	0	0	0	1	0	1
mmu-miR-223-3p	383103	Tvp23a	0	0	0	1	0	1
mmu-miR-223-3p	383295	Ypel5	0	0	0	1	0	1
mmu-miR-223-3p	383491	Prdm14	0	0	0	1	0	1
mmu-miR-223-3p	383563	Gpr25	0	0	0	1	0	1
mmu-miR-223-3p	383678	Obp2b	0	0	0	1	0	1
mmu-miR-223-3p	384061	Fndc5	0	0	0	1	0	1
mmu-miR-223-3p	384071	Slc25a34	1	0	0	0	0	1
mmu-miR-223-3p	384077	Pramel5	1	0	0	0	0	1
mmu-miR-223-3p	384281	Gatc	0	0	0	1	0	1
mmu-miR-223-3p	384452	Noto	0	0	0	1	0	1
mmu-miR-223-3p	384605	Wdr88	0	0	0	1	0	1
mmu-miR-223-3p	384763	Zfp667	0	0	0	1	0	1
mmu-miR-223-3p	385138	BC061237	0	0	0	1	0	1
mmu-miR-223-3p	385317	4930557A04Rik	0	0	0	1	0	1
mmu-miR-223-3p	385643	Kng2	0	0	0	1	0	1
mmu-miR-223-3p	386612	Thoc6	0	0	0	1	0	1
mmu-miR-223-3p	386655	Eid2	0	0	0	1	0	1
mmu-miR-223-3p	386750	Slitrk3	0	0	0	1	0	1
mmu-miR-223-3p	386753	Dbpht2	0	0	0	1	0	1
mmu-miR-223-3p	387132	Ssxb2	0	0	0	1	0	1
mmu-miR-223-3p	387524	Znrf2	0	0	0	1	0	1
mmu-miR-223-3p	393082	Mettl7a2	0	0	0	1	0	1
mmu-miR-223-3p	394430	Ugt1a10	0	0	0	1	0	1
mmu-miR-223-3p	394432	Ugt1a7c	0	0	0	1	0	1
mmu-miR-223-3p	394433	Ugt1a5	0	0	0	1	0	1
mmu-miR-223-3p	394434	Ugt1a9	0	0	0	1	0	1
mmu-miR-223-3p	394435	Ugt1a6b	0	0	0	1	0	1
mmu-miR-223-3p	394436	Ugt1a1	0	0	0	1	0	1
mmu-miR-223-3p	399566	Btbd6	0	0	0	1	0	1
mmu-miR-223-3p	399591	Tmsb15l	0	0	0	1	0	1
mmu-miR-223-3p	399599	Ccdc87	0	0	0	1	0	1
mmu-miR-223-3p	403175	Tigd4	0	0	0	1	0	1
mmu-miR-223-3p	403183	Mettl21e	0	0	0	1	0	1
mmu-miR-223-3p	406176	Olfr151	0	0	0	1	0	1
mmu-miR-223-3p	406219	Krt83	0	0	0	1	0	1
mmu-miR-223-3p	407243	Tmem189	0	0	0	1	0	1

mmu-miR-223-3p	407790	Ndufa4l2	0	0	0	1	0	1
mmu-miR-223-3p	407819	BC031181	0	0	0	1	0	1
mmu-miR-223-3p	408059	BC049352	0	0	0	1	0	1
mmu-miR-223-3p	408191	Gm5415	0	0	0	1	0	1
mmu-miR-223-3p	414077	BC056474	0	0	0	1	0	1
mmu-miR-223-3p	432442	Akap7	0	0	0	1	0	1
mmu-miR-223-3p	432478	Tmprss9	0	0	0	1	0	1
mmu-miR-223-3p	432480	Gm1553	0	0	0	1	0	1
mmu-miR-223-3p	432555	Gm5431	0	0	0	1	0	1
mmu-miR-223-3p	432591	Gm11544	0	0	0	1	0	1
mmu-miR-223-3p	432602	Krtap31-2	0	0	0	1	0	1
mmu-miR-223-3p	432611	Dnaic2	0	0	0	1	0	1
mmu-miR-223-3p	432677	Vrtn	0	0	0	1	0	1
mmu-miR-223-3p	432770	Rslcan18	0	0	0	1	0	1
mmu-miR-223-3p	432867	Defb48	0	0	0	1	0	1
mmu-miR-223-3p	432940	Fam105b	0	0	0	1	0	1
mmu-miR-223-3p	433091	Pnpla1	0	0	0	1	0	1
mmu-miR-223-3p	433107	Gm5494	0	0	0	1	0	1
mmu-miR-223-3p	433178	Spink14	0	0	0	1	0	1
mmu-miR-223-3p	433180	Spink6	0	0	0	1	0	1
mmu-miR-223-3p	433181	Spink11	0	0	0	1	0	1
mmu-miR-223-3p	433182	Gm5506	0	0	0	1	0	1
mmu-miR-223-3p	433208	D030046N08Rik	0	0	0	1	0	1
mmu-miR-223-3p	433287	Gm15455	0	0	0	1	0	1
mmu-miR-223-3p	433292	Nms	0	0	0	1	0	1
mmu-miR-223-3p	433294	Mettl21c	0	0	0	1	0	1
mmu-miR-223-3p	433386	4922505E12Rik	0	0	0	1	0	1
mmu-miR-223-3p	433424	Gm13476	0	0	0	1	0	1
mmu-miR-223-3p	433481	Gm5535	0	0	0	1	0	1
mmu-miR-223-3p	433483	Gm14135	0	0	0	1	0	1
mmu-miR-223-3p	433485	Syndig1	0	0	0	1	0	1
mmu-miR-223-3p	433486	Gm14151	0	0	0	1	0	1
mmu-miR-223-3p	433490	Defb45	0	0	0	1	0	1
mmu-miR-223-3p	433502	Wfdc6b	0	0	0	1	0	1
mmu-miR-223-3p	433619	Kprp	0	0	0	1	0	1
mmu-miR-223-3p	433638	I830077J02Rik	0	0	0	1	0	1
mmu-miR-223-3p	433693	Akirin2	0	0	0	1	0	1
mmu-miR-223-3p	433702	Ncbp1	0	0	0	1	0	1
mmu-miR-223-3p	433752	AA415398	0	0	0	1	0	1
mmu-miR-223-3p	433759	Hdac1	0	0	0	1	0	1
mmu-miR-223-3p	433766	Trim63	0	0	0	1	0	1
mmu-miR-223-3p	433864	Nom1	0	0	0	1	0	1
mmu-miR-223-3p	433882	Gm16223	0	0	0	1	0	1
mmu-miR-223-3p	433931	Pigg	0	0	0	1	0	1
mmu-miR-223-3p	433956	Heatr2	0	0	0	1	0	1
mmu-miR-223-3p	434121	Sult2a4	0	0	0	1	0	1
mmu-miR-223-3p	434128	Pnmal2	0	0	0	1	0	1
mmu-miR-223-3p	434156	Eid2b	0	0	0	1	0	1
mmu-miR-223-3p	434171	Gm5591	0	0	0	1	0	1
mmu-miR-223-3p	434172	Gm5592	0	0	0	1	0	1
mmu-miR-223-3p	434204	Whamm	0	0	0	1	0	1
mmu-miR-223-3p	434234	2610020H08Rik	0	0	0	1	0	1
mmu-miR-223-3p	434246	Trim72	0	0	0	1	0	1
mmu-miR-223-3p	434264	Sult2a5	0	0	0	1	0	1
mmu-miR-223-3p	434325	Tmem221	0	0	0	1	0	1

mmu-miR-223-3p	434341	Nlrc5	0	0	0	1	0	1
mmu-miR-223-3p	434377	Zfp560	0	0	0	1	0	1
mmu-miR-223-3p	434378	7030419G21Rik	1	0	0	0	0	1
mmu-miR-223-3p	434459	Gm5622	0	0	0	1	0	1
mmu-miR-223-3p	434484	Sp140	0	0	0	1	0	1
mmu-miR-223-3p	434689	Gm10220	0	0	0	1	0	1
mmu-miR-223-3p	434726	Gm5634	0	0	0	1	0	1
mmu-miR-223-3p	434727	Gm14511	0	0	0	1	0	1
mmu-miR-223-3p	434729	Gm5635	0	0	0	1	0	1
mmu-miR-223-3p	434784	Ldoc1	0	0	0	1	0	1
mmu-miR-223-3p	434797	Gm5640	1	0	0	0	0	1
mmu-miR-223-3p	434863	Gm15128	0	0	0	1	0	1
mmu-miR-223-3p	434864	Gm15107	0	0	0	1	0	1
mmu-miR-223-3p	434865	Luzp4	0	0	0	1	0	1
mmu-miR-223-3p	434866	Gm15127	0	0	0	1	0	1
mmu-miR-223-3p	434869	Gm15097	0	0	0	1	0	1
mmu-miR-223-3p	434903	Mageb4	0	0	0	1	0	1
mmu-miR-223-3p	435285	Krtap4-16	0	0	0	1	0	1
mmu-miR-223-3p	435350	Serpinb6e	0	0	0	1	0	1
mmu-miR-223-3p	435529	Gpr111	0	0	0	1	0	1
mmu-miR-223-3p	435565	Iigp1b	0	0	0	1	0	1
mmu-miR-223-3p	435626	Rufy4	0	0	0	1	0	1
mmu-miR-223-3p	435811	Ldlrad2	0	0	0	1	0	1
mmu-miR-223-3p	435845	Tmprss11c	0	1	0	0	0	1
mmu-miR-223-3p	436022	Dnaaf3	0	0	0	1	0	1
mmu-miR-223-3p	436062	Fam92b	0	0	0	1	0	1
mmu-miR-223-3p	436522	Try10	1	0	0	0	0	1
mmu-miR-223-3p	442801	Arhgef15	0	0	0	1	0	1
mmu-miR-223-3p	442809	4932416K20Rik	0	0	0	1	0	1
mmu-miR-223-3p	442836	A530053M12Rik	0	0	0	1	0	1
mmu-miR-223-3p	446099	Nlrp4e	0	0	0	1	0	1
mmu-miR-223-3p	446101	Xrra1	0	0	0	1	0	1
mmu-miR-223-3p	448850	Znhit3	0	0	0	1	0	1
mmu-miR-223-3p	474145	Clec4a4	0	0	0	1	0	1
mmu-miR-223-3p	493583	Itlnb	0	0	0	1	0	1
mmu-miR-223-3p	494448	Cbx6	0	0	0	1	0	1
mmu-miR-223-3p	494519	Scgb2b20	0	0	0	1	0	1
mmu-miR-223-3p	497114	Defa23	0	0	0	1	0	1
mmu-miR-223-3p	497652	Acd	0	0	0	1	0	1
mmu-miR-223-3p	503491	Defa24	0	0	0	1	0	1
mmu-miR-223-3p	503844	Ang5	0	0	0	1	0	1
mmu-miR-223-3p	504186	Chrna10	0	0	0	1	0	1
mmu-miR-223-3p	544763	Hbq1b	0	0	0	1	0	1
mmu-miR-223-3p	544864	Gm5785	0	0	0	1	0	1
mmu-miR-223-3p	544881	BB287469	1	0	0	0	0	1
mmu-miR-223-3p	544922	Zkscan4	0	0	0	1	0	1
mmu-miR-223-3p	544973	Gm10257	0	0	0	1	0	1
mmu-miR-223-3p	545007	Gm5796	0	0	0	1	0	1
mmu-miR-223-3p	545047	Gm5800	0	0	0	1	0	1
mmu-miR-223-3p	545136	Fam186b	0	0	0	1	0	1
mmu-miR-223-3p	545140	Olfr288	0	0	0	1	0	1
mmu-miR-223-3p	545253	Gm5820	0	0	0	1	0	1
mmu-miR-223-3p	545276	Gal3st3	0	0	0	1	0	1
mmu-miR-223-3p	545279	Ms4a15	0	0	0	1	0	1
mmu-miR-223-3p	545391	Gm16432	1	0	0	0	0	1

mmu-miR-223-3p	545475	Defb28	0	0	0	1	0	1
mmu-miR-223-3p	545477	Bpifa6	0	0	0	1	0	1
mmu-miR-223-3p	545486	Tubb1	0	0	0	1	0	1
mmu-miR-223-3p	545548	Lce3a	0	0	0	1	0	1
mmu-miR-223-3p	545554	Ankrd34a	0	0	0	1	0	1
mmu-miR-223-3p	545622	Ptpn3	0	0	0	1	0	1
mmu-miR-223-3p	545645	Gm13283	0	0	0	1	0	1
mmu-miR-223-3p	545646	Gm13290	0	0	0	1	0	1
mmu-miR-223-3p	545647	Gm13289	0	0	0	1	0	1
mmu-miR-223-3p	545648	Gm13272	0	0	0	1	0	1
mmu-miR-223-3p	545649	Gm13276	0	0	0	1	0	1
mmu-miR-223-3p	545650	Gm13277	0	0	0	1	0	1
mmu-miR-223-3p	545651	Gm13278	0	0	0	1	0	1
mmu-miR-223-3p	545652	Gm13275	0	0	0	1	0	1
mmu-miR-223-3p	545653	Gm13279	0	0	0	1	0	1
mmu-miR-223-3p	545693	Gm13043	0	0	0	1	0	1
mmu-miR-223-3p	545725	Mterf	0	0	0	1	0	1
mmu-miR-223-3p	545732	4933402N22Rik	0	0	0	1	0	1
mmu-miR-223-3p	545798	Tmem233	0	0	0	1	0	1
mmu-miR-223-3p	545861	Gm5878	0	0	0	1	0	1
mmu-miR-223-3p	545886	Gm5886	0	0	0	1	0	1
mmu-miR-223-3p	545913	Zscan4d	0	0	0	1	0	1
mmu-miR-223-3p	545925	Psg27	0	0	0	1	0	1
mmu-miR-223-3p	545929	Gm5891	0	0	0	1	0	1
mmu-miR-223-3p	545947	Scgb2b19	0	0	0	1	0	1
mmu-miR-223-3p	546024	Crxos1	0	0	0	1	0	1
mmu-miR-223-3p	546038	Spag11b	0	0	0	1	0	1
mmu-miR-223-3p	546272	Gm5934	0	0	0	1	0	1
mmu-miR-223-3p	546282	Gm5935	0	0	0	1	0	1
mmu-miR-223-3p	546325	Gm5936	1	0	0	0	0	1
mmu-miR-223-3p	546519	Tmem235	0	0	0	1	0	1
mmu-miR-223-3p	546643	I830127L07Rik	0	0	0	1	0	1
mmu-miR-223-3p	547109	Trim43a	0	0	0	1	0	1
mmu-miR-223-3p	547334	LOC547334	0	0	0	1	0	1
mmu-miR-223-3p	550619	Arid3c	0	0	0	1	0	1
mmu-miR-223-3p	552899	Ugt2a2	0	0	0	1	0	1
mmu-miR-223-3p	553127	Cxx1b	0	0	0	1	0	1
mmu-miR-223-3p	554292	AB099516	0	0	0	1	0	1
mmu-miR-223-3p	574404	Gm14685	1	0	0	0	0	1
mmu-miR-223-3p	574405	DXBay18	1	0	0	0	0	1
mmu-miR-223-3p	574428	Zmynd15	0	0	0	1	0	1
mmu-miR-223-3p	574438	Xlr5a	0	0	0	1	0	1
mmu-miR-223-3p	594844	Tceal3	0	0	0	1	0	1
mmu-miR-223-3p	606496	Gsk3a	0	0	0	1	0	1
mmu-miR-223-3p	619289	Rfx8	0	0	0	1	0	1
mmu-miR-223-3p	619294	4930428D18Rik	0	0	0	1	0	1
mmu-miR-223-3p	619331	Zfp551	0	0	0	1	0	1
mmu-miR-223-3p	619332	4933416C03Rik	0	0	0	1	0	1
mmu-miR-223-3p	619548	Defb42	0	0	0	1	0	1
mmu-miR-223-3p	619937	Gm6115	0	0	0	1	0	1
mmu-miR-223-3p	619991	Gm6121	0	0	0	1	0	1
mmu-miR-223-3p	620078	C130026I21Rik	1	0	0	0	0	1
mmu-miR-223-3p	620253	Dcpp3	1	0	0	0	0	1
mmu-miR-223-3p	620480	Gm6155	1	0	0	0	0	1
mmu-miR-223-3p	620592	Tmem28	0	1	0	0	0	1

mmu-miR-223-3p	620709	Lcn6	0	0	0	1	0	1
mmu-miR-223-3p	620913	Gm12185	0	0	0	1	0	1
mmu-miR-223-3p	621080	AI429214	0	0	0	1	0	1
mmu-miR-223-3p	621239	Nhlrc4	0	0	0	1	0	1
mmu-miR-223-3p	621495	Krtap28-10	0	0	0	1	0	1
mmu-miR-223-3p	621603	Aldh3b2	0	0	0	1	0	1
mmu-miR-223-3p	622307	5830473C10Rik	0	0	0	1	0	1
mmu-miR-223-3p	622408	Mcidas	0	0	0	1	0	1
mmu-miR-223-3p	622474	Smok3b	0	0	0	1	0	1
mmu-miR-223-3p	622531	Gm6330	0	0	0	1	0	1
mmu-miR-223-3p	622554	1700123I01Rik	0	0	0	1	0	1
mmu-miR-223-3p	622629	Gm10318	0	0	0	1	0	1
mmu-miR-223-3p	622645	Tmem200c	0	0	0	1	0	1
mmu-miR-223-3p	623121	Pydc4	0	0	0	1	0	1
mmu-miR-223-3p	623166	Prr23a	0	0	0	1	0	1
mmu-miR-223-3p	623230	Tmem200b	0	0	0	1	0	1
mmu-miR-223-3p	623459	Gm6432	0	0	0	1	0	1
mmu-miR-223-3p	623474	Rad54b	0	0	0	1	0	1
mmu-miR-223-3p	624219	Gm6484	0	0	0	1	0	1
mmu-miR-223-3p	624245	Speer4e	0	0	0	1	0	1
mmu-miR-223-3p	624439	Scgb2b15	0	0	0	1	0	1
mmu-miR-223-3p	625098	Slc38a6	0	0	0	1	0	1
mmu-miR-223-3p	625249	Gpx4	0	0	0	1	0	1
mmu-miR-223-3p	625286	Tmem236	0	0	0	1	0	1
mmu-miR-223-3p	625424	Gm6583	0	0	0	1	0	1
mmu-miR-223-3p	625464	Gm6588	0	0	0	1	0	1
mmu-miR-223-3p	625530	Usp17le	0	0	0	1	0	1
mmu-miR-223-3p	626359	Wdr93	0	0	0	1	0	1
mmu-miR-223-3p	626415	4930467E23Rik	0	0	0	1	0	1
mmu-miR-223-3p	626578	Gbp10	0	0	0	1	0	1
mmu-miR-223-3p	626708	Defa26	0	0	0	1	0	1
mmu-miR-223-3p	626943	Pramef17	0	0	0	1	0	1
mmu-miR-223-3p	626995	Gm13128	0	0	0	1	0	1
mmu-miR-223-3p	627081	Xlr5b	0	0	0	1	0	1
mmu-miR-223-3p	627191	Syndig1l	0	0	0	1	0	1
mmu-miR-223-3p	627214	Fam196a	0	0	0	1	0	1
mmu-miR-223-3p	627280	Vmn1r90	0	1	0	0	0	1
mmu-miR-223-3p	627488	Gm6763	0	0	0	1	0	1
mmu-miR-223-3p	627914	Gm14430	0	0	0	1	0	1
mmu-miR-223-3p	628100	Fbxo39	0	0	0	1	0	1
mmu-miR-223-3p	628308	Gm14420	0	0	0	1	0	1
mmu-miR-223-3p	628664	Gm6902	0	0	0	1	0	1
mmu-miR-223-3p	628693	Gm6904	0	0	0	1	0	1
mmu-miR-223-3p	628705	Phf11c	0	0	0	1	0	1
mmu-miR-223-3p	628779	Hs3st4	0	0	0	1	0	1
mmu-miR-223-3p	628900	Serpina3i	0	0	0	1	0	1
mmu-miR-223-3p	628923	Gm14744	0	0	0	1	0	1
mmu-miR-223-3p	628991	Obp1b	0	0	0	1	0	1
mmu-miR-223-3p	629147	Ctxn3	0	0	0	1	0	1
mmu-miR-223-3p	629219	Sult2a6	0	0	0	1	0	1
mmu-miR-223-3p	629242	Gm6958	0	0	0	1	0	1
mmu-miR-223-3p	629378	Dact3	0	0	0	1	0	1
mmu-miR-223-3p	629754	Wfdc9	0	0	0	1	0	1
mmu-miR-223-3p	629761	Wfdc11	0	0	0	1	0	1
mmu-miR-223-3p	630537	Dcpp2	1	0	0	0	0	1

mmu-miR-223-3p	630579	Zfp808	0	0	0	1	0	1
mmu-miR-223-3p	630663	Gm7040	0	0	0	1	0	1
mmu-miR-223-3p	630836	2010315B03Rik	0	0	0	1	0	1
mmu-miR-223-3p	630952	Ang6	0	0	0	1	0	1
mmu-miR-223-3p	631101	Lce1k	0	0	0	1	0	1
mmu-miR-223-3p	631323	Gm12250	0	0	0	1	0	1
mmu-miR-223-3p	631784	Gm7073	0	0	0	1	0	1
mmu-miR-223-3p	631990	Cdr1	0	0	0	1	0	1
mmu-miR-223-3p	632778	Gm7092	0	0	0	1	0	1
mmu-miR-223-3p	632971	Rergl	0	0	0	1	0	1
mmu-miR-223-3p	633057	Gm7102	0	0	0	1	0	1
mmu-miR-223-3p	633417	LOC633417	1	0	0	0	0	1
mmu-miR-223-3p	634650	Gbp11	0	0	0	1	0	1
mmu-miR-223-3p	634731	Susd1	0	0	0	1	0	1
mmu-miR-223-3p	635169	CK137956	0	0	0	1	0	1
mmu-miR-223-3p	635668	Fbxw23	1	0	0	0	0	1
mmu-miR-223-3p	636808	Cntnap5a	0	1	0	0	0	1
mmu-miR-223-3p	636931	Trim71	0	0	0	1	0	1
mmu-miR-223-3p	637578	Gm20765	0	0	0	1	0	1
mmu-miR-223-3p	638580	Gm7244	0	0	0	1	0	1
mmu-miR-223-3p	639025	Gm7257	0	0	0	1	0	1
mmu-miR-223-3p	639606	Gm10420	0	0	0	1	0	1
mmu-miR-223-3p	639634	Aadacl2	0	0	0	1	0	1
mmu-miR-223-3p	639774	Skint8	0	0	0	1	0	1
mmu-miR-223-3p	639931	LOC639931	0	0	0	1	0	1
mmu-miR-223-3p	640370	Gm7292	0	0	0	1	0	1
mmu-miR-223-3p	641240	LOC641240	0	0	0	1	0	1
mmu-miR-223-3p	641340	Nrbf2	0	0	0	1	0	1
mmu-miR-223-3p	652925	Tmem243	1	0	0	0	0	1
mmu-miR-223-3p	654309	Nrp	0	0	0	1	0	1
mmu-miR-223-3p	654362	Dear1	0	0	0	1	0	1
mmu-miR-223-3p	654453	Defb33	0	0	0	1	0	1
mmu-miR-223-3p	654459	Defb25	0	0	0	1	0	1
mmu-miR-223-3p	654462	Kncn	0	0	0	1	0	1
mmu-miR-223-3p	654470	Tctn1	0	0	0	1	0	1
mmu-miR-223-3p	654498	Hhla1	0	0	0	1	0	1
mmu-miR-223-3p	654795	Sdr39u1	0	0	0	1	0	1
mmu-miR-223-3p	654801	Zfp784	0	0	0	1	0	1
mmu-miR-223-3p	654812	Angptl7	0	0	0	1	0	1
mmu-miR-223-3p	654818	Smco3	0	0	0	1	0	1
mmu-miR-223-3p	664829	Slx	0	0	0	1	0	1
mmu-miR-223-3p	664994	Isoc2a	0	0	0	1	0	1
mmu-miR-223-3p	665001	Gm14391	0	0	0	1	0	1
mmu-miR-223-3p	665033	Col6a5	0	0	0	1	0	1
mmu-miR-223-3p	665211	Gm14326	0	0	0	1	0	1
mmu-miR-223-3p	665255	Vmn2r28	0	0	0	1	0	1
mmu-miR-223-3p	665301	Gm20773	0	0	0	1	0	1
mmu-miR-223-3p	665413	Gm7628	0	0	0	1	0	1
mmu-miR-223-3p	665574	Gm7694	0	0	0	1	0	1
mmu-miR-223-3p	665596	Hist1h2bq	1	0	0	0	0	1
mmu-miR-223-3p	665615	Gm7714	0	0	0	1	0	1
mmu-miR-223-3p	665700	Hmcn2	0	0	0	1	0	1
mmu-miR-223-3p	665775	Bod1l	0	0	0	1	0	1
mmu-miR-223-3p	665780	Zscan4b	0	0	0	1	0	1
mmu-miR-223-3p	665848	Zscan4e	0	0	0	1	0	1

mmu-miR-223-3p	665891	Krtap4-1	0	0	0	1	0	1
mmu-miR-223-3p	665902	Zscan4f	0	0	0	1	0	1
mmu-miR-223-3p	665998	Krtap4-9	0	0	0	1	0	1
mmu-miR-223-3p	666168	Cyp4a31	0	0	0	1	0	1
mmu-miR-223-3p	666184	Gm15080	0	0	0	1	0	1
mmu-miR-223-3p	666186	Gm7970	0	1	0	0	0	1
mmu-miR-223-3p	666207	Gm7980	0	1	0	0	0	1
mmu-miR-223-3p	666253	Gm8005	0	1	0	0	0	1
mmu-miR-223-3p	666279	Dspp	0	0	0	1	0	1
mmu-miR-223-3p	666311	Zscan25	0	0	0	1	0	1
mmu-miR-223-3p	666329	Gm3317	0	0	0	1	0	1
mmu-miR-223-3p	666376	Gm8068	0	1	0	0	0	1
mmu-miR-223-3p	666420	Gm8094	0	1	0	0	0	1
mmu-miR-223-3p	666485	Gm8127	0	1	0	0	0	1
mmu-miR-223-3p	666502	Gm8138	0	1	0	0	0	1
mmu-miR-223-3p	666532	Gm13139	0	0	0	1	0	1
mmu-miR-223-3p	666647	Gm8212	0	1	0	0	0	1
mmu-miR-223-3p	666660	Gm8220	0	1	0	0	0	1
mmu-miR-223-3p	666675	Gm8229	0	1	0	0	0	1
mmu-miR-223-3p	666678	Gm8232	0	1	0	0	0	1
mmu-miR-223-3p	666704	Samd1	0	0	0	1	0	1
mmu-miR-223-3p	666731	Trim43c	0	0	0	1	0	1
mmu-miR-223-3p	666744	Gm8267	0	0	0	1	0	1
mmu-miR-223-3p	666747	Trim43b	0	0	0	1	0	1
mmu-miR-223-3p	666790	Gm8290	1	0	0	0	0	1
mmu-miR-223-3p	667118	Zbed6	0	0	0	1	0	1
mmu-miR-223-3p	667214	9930111J21Rik1	0	0	0	1	0	1
mmu-miR-223-3p	667250	Gm12657	0	0	0	1	0	1
mmu-miR-223-3p	667253	Gm8539	0	0	0	1	0	1
mmu-miR-223-3p	667370	I830012O16Rik	0	0	0	1	0	1
mmu-miR-223-3p	667373	Gm14446	0	0	0	1	0	1
mmu-miR-223-3p	667631	Gm8738	0	0	0	1	0	1
mmu-miR-223-3p	667692	Gm8764	0	0	0	1	0	1
mmu-miR-223-3p	667772	Myh15	0	0	0	1	0	1
mmu-miR-223-3p	667794	Gm8817	0	0	0	1	0	1
mmu-miR-223-3p	667823	Trim5	0	0	0	1	0	1
mmu-miR-223-3p	667962	Gm8898	0	0	0	1	0	1
mmu-miR-223-3p	668039	Gm14434	0	0	0	1	0	1
mmu-miR-223-3p	668137	Gm8994	0	0	0	1	0	1
mmu-miR-223-3p	668178	Mettl7a3	0	0	0	1	0	1
mmu-miR-223-3p	668200	Gm9041	0	0	0	1	0	1
mmu-miR-223-3p	668339	Gm9112	0	0	0	1	0	1
mmu-miR-223-3p	668357	Dmrtc1c2	0	0	0	1	0	1
mmu-miR-223-3p	668501	Zfp507	0	0	0	1	0	1
mmu-miR-223-3p	668539	Gm9229	0	0	0	1	0	1
mmu-miR-223-3p	668620	Zfp936	0	0	0	1	0	1
mmu-miR-223-3p	668661	2410002F23Rik	0	0	0	1	0	1
mmu-miR-223-3p	668727	Mrgpra2a	0	0	0	1	0	1
mmu-miR-223-3p	668758	Fbxw28	0	0	0	1	0	1
mmu-miR-223-3p	668829	Gm9385	0	0	0	1	0	1
mmu-miR-223-3p	668936	Gm14217	0	0	0	1	0	1
mmu-miR-223-3p	670358	Gm9484	0	0	0	1	0	1
mmu-miR-223-3p	670472	Gm11569	0	0	0	1	0	1
mmu-miR-223-3p	670482	Gm11554	0	0	0	1	0	1
mmu-miR-223-3p	670550	Gm11565	0	0	0	1	0	1

mmu-miR-223-3p	671878	Gm14680	0	0	0	1	0	1
mmu-miR-223-3p	672511	Rnf213	0	0	0	1	0	1
mmu-miR-223-3p	672682	Gm9573	0	1	0	0	0	1
mmu-miR-223-3p	673132	Gm16506	0	1	0	0	0	1
mmu-miR-223-3p	675812	Zfp605	0	0	0	1	0	1
mmu-miR-223-3p	676676	F530104D19Rik	0	0	0	1	0	1
mmu-miR-223-3p	677156	Gm9705	0	0	0	1	0	1
mmu-miR-223-3p	677296	Fcrl6	0	0	0	1	0	1
mmu-miR-223-3p	692132	Trappc3l	0	0	0	1	0	1
mmu-miR-223-3p	751865	Sap25	0	0	0	1	0	1
mmu-miR-223-3p	791260	Tomt	0	0	0	1	0	1
mmu-miR-223-3p	791386	Gm10113	0	0	0	1	0	1
mmu-miR-223-3p	791387	Gm9869	0	0	0	1	0	1
mmu-miR-223-3p	1E+08	Wfdc17	0	0	0	1	0	1
mmu-miR-223-3p	1E+08	Mfap1b	0	0	0	1	0	1
mmu-miR-223-3p	1E+08	Tmsb15b2	0	0	0	1	0	1
mmu-miR-223-3p	1E+08	BC100530	0	1	0	0	0	1
mmu-miR-223-3p	1E+08	Gm15114	0	0	0	1	0	1
mmu-miR-223-3p	1E+08	Gm11837	0	0	0	1	0	1
mmu-miR-223-3p	1E+08	Gm11744	0	0	0	1	0	1
mmu-miR-223-3p	1E+08	Isg15	0	0	0	1	0	1
mmu-miR-223-3p	1E+08	Gm14548	0	0	0	1	0	1
mmu-miR-223-3p	1E+08	Gm15284	0	0	0	1	0	1
mmu-miR-223-3p	1E+08	Gm1979	0	0	0	1	0	1
mmu-miR-223-3p	1E+08	Gm1993	0	0	0	1	0	1
mmu-miR-223-3p	1E+08	Gm13552	0	0	0	1	0	1
mmu-miR-223-3p	1E+08	Gm20793	0	0	0	1	0	1
mmu-miR-223-3p	1E+08	Gm2012	0	0	0	1	0	1
mmu-miR-223-3p	1E+08	Gm20795	0	0	0	1	0	1
mmu-miR-223-3p	1E+08	Gm10471	0	0	0	1	0	1
mmu-miR-223-3p	1E+08	Gm2022	1	0	0	0	0	1
mmu-miR-223-3p	1E+08	0610010B08Rik	0	0	0	1	0	1
mmu-miR-223-3p	1E+08	Gm14525	0	0	0	1	0	1
mmu-miR-223-3p	1E+08	Gm14295	0	0	0	1	0	1
mmu-miR-223-3p	1E+08	Tmem254c	0	0	0	1	0	1
mmu-miR-223-3p	1E+08	Duxbl2	0	0	0	1	0	1
mmu-miR-223-3p	1E+08	Gm10058	0	0	0	1	0	1
mmu-miR-223-3p	1E+08	Tmem254b	0	0	0	1	0	1
mmu-miR-223-3p	1E+08	Gm2136	0	0	0	1	0	1
mmu-miR-223-3p	1E+08	Duxbl3	0	0	0	1	0	1
mmu-miR-223-3p	1E+08	Gm10147	0	0	0	1	0	1
mmu-miR-223-3p	1E+08	Gm10096	0	0	0	1	0	1
mmu-miR-223-3p	1E+08	Gm2260	0	0	0	1	0	1
mmu-miR-223-3p	1E+08	Gm2274	0	0	0	1	0	1
mmu-miR-223-3p	1E+08	Gm10486	0	0	0	1	0	1
mmu-miR-223-3p	1E+08	Gm14819	0	0	0	1	0	1
mmu-miR-223-3p	1E+08	Msmp	0	0	0	1	0	1
mmu-miR-223-3p	1E+08	Gm2373	0	0	0	1	0	1
mmu-miR-223-3p	1E+08	Gm10051	0	0	0	1	0	1
mmu-miR-223-3p	1E+08	Hrct1	0	0	0	1	0	1
mmu-miR-223-3p	1E+08	Gm2423	0	0	0	1	0	1
mmu-miR-223-3p	1E+08	Gm2427	0	0	0	1	0	1
mmu-miR-223-3p	1E+08	Gm15093	0	0	0	1	0	1
mmu-miR-223-3p	1E+08	Gm15085	0	0	0	1	0	1
mmu-miR-223-3p	1E+08	Gm10229	0	0	0	1	0	1

mmu-miR-223-3p	1E+08	Gm10228	0	0	0	1	0	1
mmu-miR-223-3p	1E+08	Gm20831	0	0	0	1	0	1
mmu-miR-223-3p	1E+08	Gm11595	0	0	0	1	0	1
mmu-miR-223-3p	1E+08	Dynlt1f	1	0	0	0	0	1
mmu-miR-223-3p	1E+08	Dynlt1c	0	0	0	1	0	1
mmu-miR-223-3p	1E+08	Kcnj13	0	0	0	1	0	1
mmu-miR-223-3p	1E+08	Gm15319	0	0	0	1	0	1
mmu-miR-223-3p	1E+08	Gm2897	0	0	0	1	0	1
mmu-miR-223-3p	1E+08	Gm20851	0	0	0	1	0	1
mmu-miR-223-3p	1E+08	Cyp4a32	0	0	0	1	0	1
mmu-miR-223-3p	1E+08	Gm13040	0	0	0	1	0	1
mmu-miR-223-3p	1E+08	Gm3004	0	0	0	1	0	1
mmu-miR-223-3p	1E+08	Gm13057	0	0	0	1	0	1
mmu-miR-223-3p	1E+08	Gm16390	0	0	0	1	0	1
mmu-miR-223-3p	1E+08	Gm10145	0	0	0	1	0	1
mmu-miR-223-3p	1E+08	G630090E17Rik	0	0	0	1	0	1
mmu-miR-223-3p	1E+08	Ahnak2	0	0	0	1	0	1
mmu-miR-223-3p	1E+08	AU018829	0	0	0	1	0	1
mmu-miR-223-3p	1E+08	Gm3259	0	0	0	1	0	1
mmu-miR-223-3p	1E+08	Tcp10c	0	0	0	1	0	1
mmu-miR-223-3p	1E+08	Gm20873	0	0	0	1	0	1
mmu-miR-223-3p	1E+08	Cyp3a41b	0	0	0	1	0	1
mmu-miR-223-3p	1E+08	Gm3325	0	0	0	1	0	1
mmu-miR-223-3p	1E+08	Gm3362	1	0	0	0	0	1
mmu-miR-223-3p	1E+08	Gm3363	1	0	0	0	0	1
mmu-miR-223-3p	1E+08	Gm3364	0	0	0	1	0	1
mmu-miR-223-3p	1E+08	Gm3376	0	0	0	1	0	1
mmu-miR-223-3p	1E+08	Zkscan16	0	0	0	1	0	1
mmu-miR-223-3p	1E+08	Gm13157	1	0	0	0	0	1
mmu-miR-223-3p	1E+08	Gm3500	0	0	0	1	0	1
mmu-miR-223-3p	1E+08	Gm3470	0	0	0	1	0	1
mmu-miR-223-3p	1E+08	Gm10104	0	0	0	1	0	1
mmu-miR-223-3p	1E+08	Gm3494	0	0	0	1	0	1
mmu-miR-223-3p	1E+08	Gm20885	0	0	0	1	0	1
mmu-miR-223-3p	1E+08	Gm10408	0	0	0	1	0	1
mmu-miR-223-3p	1E+08	Gm3558	0	0	0	1	0	1
mmu-miR-223-3p	1E+08	Gm15308	0	0	0	1	0	1
mmu-miR-223-3p	1E+08	Gm14850	0	0	0	1	0	1
mmu-miR-223-3p	1E+08	Gm15315	0	0	0	1	0	1
mmu-miR-223-3p	1E+08	Gm10094	0	0	0	1	0	1
mmu-miR-223-3p	1E+08	9230110K08Rik	0	0	0	1	0	1
mmu-miR-223-3p	1E+08	Gm10377	0	0	0	1	0	1
mmu-miR-223-3p	1E+08	Gm20901	0	0	0	1	0	1
mmu-miR-223-3p	1E+08	Gm3667	0	0	0	1	0	1
mmu-miR-223-3p	1E+08	Gm10488	0	0	0	1	0	1
mmu-miR-223-3p	1E+08	Gm14632	0	0	0	1	0	1
mmu-miR-223-3p	1E+08	Gm3696	0	0	0	1	0	1
mmu-miR-223-3p	1E+08	Gm10230	0	0	0	1	0	1
mmu-miR-223-3p	1E+08	Gm3776	0	0	0	1	0	1
mmu-miR-223-3p	1E+08	Gm10639	0	0	0	1	0	1
mmu-miR-223-3p	1E+08	Gm20910	0	0	0	1	0	1
mmu-miR-223-3p	1E+08	Gm10375	0	0	0	1	0	1
mmu-miR-223-3p	1E+08	Gm10705	0	0	0	1	0	1
mmu-miR-223-3p	1E+08	Gm16532	0	0	0	1	0	1
mmu-miR-223-3p	1E+08	Gm20923	0	0	0	1	0	1

mmu-miR-223-3p	1E+08	Gm3896	0	0	0	1	0	1
mmu-miR-223-3p	1E+08	Gm20928	0	0	0	1	0	1
mmu-miR-223-3p	1E+08	Gm20931	0	0	0	1	0	1
mmu-miR-223-3p	1E+08	Gm3985	0	0	0	1	0	1
mmu-miR-223-3p	1E+08	Gm4024	0	0	0	1	0	1
mmu-miR-223-3p	1E+08	Gm4027	0	0	0	1	0	1
mmu-miR-223-3p	1E+08	Vmn2r40	0	1	0	0	0	1
mmu-miR-223-3p	1E+08	Gm14458	0	0	0	1	0	1
mmu-miR-223-3p	1E+08	Prdm11	0	1	0	0	0	1
mmu-miR-223-3p	1E+08	Gm4055	0	0	0	1	0	1
mmu-miR-223-3p	1E+08	Vmn2r41	0	1	0	0	0	1
mmu-miR-223-3p	1E+08	Gm4064	0	0	0	1	0	1
mmu-miR-223-3p	1E+08	Gm4070	0	0	0	1	0	1
mmu-miR-223-3p	1E+08	Gm10256	0	0	0	1	0	1
mmu-miR-223-3p	1E+08	Gm10352	0	0	0	1	0	1
mmu-miR-223-3p	1E+08	Llph-ps2	0	0	0	1	0	1
mmu-miR-223-3p	1E+08	Tmem207	0	0	0	1	0	1
mmu-miR-223-3p	1E+08	9130023H24Rik	0	0	0	1	0	1
mmu-miR-223-3p	1E+08	Gm10696	0	0	0	1	0	1
mmu-miR-223-3p	1E+08	Sult2a2	0	0	0	1	0	1
mmu-miR-223-3p	1E+08	Gm4297	0	0	0	1	0	1
mmu-miR-223-3p	1E+08	Gm15583	0	0	0	1	0	1
mmu-miR-223-3p	1E+08	Nps	0	0	0	1	0	1
mmu-miR-223-3p	1E+08	Gm15453	0	0	0	1	0	1
mmu-miR-223-3p	1E+08	Gm4340	0	0	0	1	0	1
mmu-miR-223-3p	1E+08	Gm4345	0	0	0	1	0	1
mmu-miR-223-3p	1E+08	Ankrd66	0	0	0	1	0	1
mmu-miR-223-3p	1E+08	Gm4392	0	0	0	1	0	1
mmu-miR-223-3p	1E+08	Gm14308	0	0	0	1	0	1
mmu-miR-223-3p	1E+08	Gm4425	0	0	0	1	0	1
mmu-miR-223-3p	1E+08	Gm4450	0	0	0	1	0	1
mmu-miR-223-3p	1E+08	Zfp955b	0	0	0	1	0	1
mmu-miR-223-3p	1E+08	Gm15776	0	0	0	1	0	1
mmu-miR-223-3p	1E+08	Srcap	0	0	0	1	0	1
mmu-miR-223-3p	1E+08	Vmn1r132	0	0	0	1	0	1
mmu-miR-223-3p	1E+08	Gm4567	0	0	0	1	0	1
mmu-miR-223-3p	1E+08	Gm10662	0	0	0	1	0	1
mmu-miR-223-3p	1E+08	Scgb2b7	0	0	0	1	0	1
mmu-miR-223-3p	1E+08	Scgb1b30	0	1	0	0	0	1
mmu-miR-223-3p	1E+08	R3hdml	0	0	0	1	0	1
mmu-miR-223-3p	1E+08	Gm4724	0	0	0	1	0	1
mmu-miR-223-3p	1E+08	Gm14378	0	0	0	1	0	1
mmu-miR-223-3p	1E+08	LOC100044656	0	0	0	1	0	1
mmu-miR-223-3p	1E+08	LOC100044742	0	0	0	1	0	1
mmu-miR-223-3p	1E+08	LOC100044900	0	0	0	1	0	1
mmu-miR-223-3p	1E+08	LOC100045026	0	0	0	1	0	1
mmu-miR-223-3p	1E+08	LOC100045688	0	0	0	1	0	1
mmu-miR-223-3p	1E+08	Rnf223	0	0	0	1	0	1
mmu-miR-223-3p	1E+08	LOC100045924	0	0	0	1	0	1
mmu-miR-223-3p	1E+08	LOC100046079	0	0	0	1	0	1
mmu-miR-223-3p	1E+08	LOC100046151	0	0	0	1	0	1
mmu-miR-223-3p	1E+08	LOC100046223	0	0	0	1	0	1
mmu-miR-223-3p	1E+08	LOC100046899	0	0	0	1	0	1
mmu-miR-223-3p	1E+08	LOC100047146	0	0	0	1	0	1
mmu-miR-223-3p	1E+08	LOC100047577	0	0	0	1	0	1

mmu-miR-223-3p	1E+08	LOC100048119	0	0	0	1	0	1
mmu-miR-223-3p	1E+08	LOC100048410	0	0	0	1	0	1
mmu-miR-223-3p	1E+08	LOC100048483	0	0	0	1	0	1
mmu-miR-223-3p	1E+08	LOC100048499	0	0	0	1	0	1
mmu-miR-223-3p	1E+08	Wdr96	0	0	0	1	0	1
mmu-miR-223-3p	1E+08	LOC100048557	0	0	0	1	0	1
mmu-miR-223-3p	1E+08	Ddx43	0	0	0	1	0	1
mmu-miR-223-3p	1E+08	Adat3	0	0	0	1	0	1
mmu-miR-223-3p	1E+08	5430421N21Rik	0	0	0	1	0	1
mmu-miR-223-3p	1E+08	Gm20408	0	0	0	1	0	1
mmu-miR-223-3p	1E+08	Gm20579	0	0	0	1	0	1
mmu-miR-223-3p	1E+08	Gm20581	0	0	0	1	0	1
mmu-miR-223-3p	1E+08	Gm12689	0	0	0	1	0	1
mmu-miR-223-3p	1E+08	Gm10910	0	0	0	1	0	1
mmu-miR-223-3p	1E+08	EU599041	0	0	0	1	0	1
mmu-miR-223-3p	1E+08	Krtap10-4	0	0	0	1	0	1
mmu-miR-223-3p	1E+08	Gm14124	0	0	0	1	0	1
mmu-miR-223-3p	1E+08	AK010878	0	0	0	1	0	1
mmu-miR-223-3p	1E+08	Fam150b	0	0	0	1	0	1
mmu-miR-223-3p	1E+08	Defa2	0	0	0	1	0	1
mmu-miR-223-3p	1E+08	Gm14431	0	0	0	1	0	1
mmu-miR-223-3p	1E+08	D930001M23Rik	0	0	0	1	0	1
mmu-miR-223-3p	1E+08	Gm11559	0	0	0	1	0	1
mmu-miR-223-3p	1E+08	Adh6b	0	0	0	1	0	1
mmu-miR-223-3p	1.01E+08	4930469G21Rik	1	0	0	0	0	1
mmu-miR-223-3p	1.01E+08	Tsga8	1	0	0	0	0	1
mmu-miR-223-3p	1.01E+08	Gm19345	0	0	0	1	0	1
mmu-miR-223-3p	1.01E+08	Kifc1	0	0	0	1	0	1
mmu-miR-223-3p	1.01E+08	LOC100502818	0	0	0	1	0	1
mmu-miR-223-3p	1.01E+08	Epg5	0	0	0	1	0	1
mmu-miR-223-3p	1.01E+08	Ccdc13	0	0	0	1	0	1
mmu-miR-223-3p	1.01E+08	Gm12854	0	0	0	1	0	1
mmu-miR-223-3p	1.01E+08	Gm684	1	0	0	0	0	1
mmu-miR-223-3p	1.01E+08	Gm19497	0	0	0	1	0	1
mmu-miR-223-3p	1.01E+08	Armcx4	0	0	0	1	0	1
mmu-miR-223-3p	1.01E+08	Gm19549	0	0	0	1	0	1
mmu-miR-223-3p	1.01E+08	LOC100503217	0	0	0	1	0	1
mmu-miR-223-3p	1.01E+08	LOC100503235	0	0	0	1	0	1
mmu-miR-223-3p	1.01E+08	LOC100503254	0	0	0	1	0	1
mmu-miR-223-3p	1.01E+08	Pifo	0	0	0	1	0	1
mmu-miR-223-3p	1.01E+08	Gm4980	0	1	0	0	0	1
mmu-miR-223-3p	1.01E+08	Gm15433	0	0	0	1	0	1
mmu-miR-223-3p	1.01E+08	Bbip1	0	0	0	1	0	1
mmu-miR-223-3p	1.01E+08	Fsbp	0	0	0	1	0	1
mmu-miR-223-3p	1.01E+08	Rpl5	0	0	0	1	0	1
mmu-miR-223-3p	1.01E+08	Gm7931	0	0	0	1	0	1
mmu-miR-223-3p	1.01E+08	Pet100	0	0	0	1	0	1
mmu-miR-223-3p	1.01E+08	Smpd5	0	0	0	1	0	1
mmu-miR-223-3p	1.01E+08	Gm6040	0	0	0	1	0	1
mmu-miR-223-3p	1.01E+08	Gm20033	0	0	0	1	0	1
mmu-miR-223-3p	1.01E+08	Gm6537	0	0	0	1	0	1
mmu-miR-223-3p	1.01E+08	Gm10591	0	0	0	1	0	1
mmu-miR-223-3p	1.01E+08	Gm16340	0	0	0	1	0	1
mmu-miR-223-3p	1.01E+08	Gm13304	0	0	0	1	0	1
mmu-miR-223-3p	1.01E+08	H2-Ea-ps	1	0	0	0	0	1

mmu-miR-223-3p	1.01E+08	Gm12367	0	0	0	1	0	1
mmu-miR-223-3p	1.01E+08	Smlr1	0	0	0	1	0	1
mmu-miR-223-3p	1.01E+08	LOC100504500	0	0	0	1	0	1
mmu-miR-223-3p	1.01E+08	3425401B19Rik	0	0	0	1	0	1
mmu-miR-223-3p	1.01E+08	Gm21996	0	0	0	1	0	1
mmu-miR-223-3p	1.01E+08	Tmppe	0	0	0	1	0	1
mmu-miR-223-3p	1.01E+08	LOC100504863	0	0	0	1	0	1
mmu-miR-223-3p	1.01E+08	LOC100504912	0	0	0	1	0	1
mmu-miR-223-3p	1.01E+08	LOC100504922	0	0	0	1	0	1
mmu-miR-223-3p	1.01E+08	LOC100504934	0	0	0	1	0	1
mmu-miR-223-3p	1.01E+08	LOC100504965	0	0	0	1	0	1
mmu-miR-223-3p	1.01E+08	Gm21002	0	0	0	1	0	1
mmu-miR-223-3p	1.01E+08	Gm21949	0	0	0	1	0	1
mmu-miR-223-3p	1.01E+08	A430107P09Rik	0	0	0	1	0	1
mmu-miR-223-3p	1.01E+08	Gm20604	0	0	0	1	0	1
mmu-miR-223-3p	1.01E+08	Gm21071	0	0	0	1	0	1
mmu-miR-223-3p	1.01E+08	LOC100861615	0	0	0	1	0	1
mmu-miR-223-3p	1.01E+08	Gm21083	0	0	0	1	0	1
mmu-miR-223-3p	1.01E+08	Gm21098	0	0	0	1	0	1
mmu-miR-223-3p	1.01E+08	LOC100861657	0	0	0	1	0	1
mmu-miR-223-3p	1.01E+08	Gm21117	0	0	0	1	0	1
mmu-miR-223-3p	1.01E+08	Gm21119	0	0	0	1	0	1
mmu-miR-223-3p	1.01E+08	Gm21149	0	0	0	1	0	1
mmu-miR-223-3p	1.01E+08	LOC100861722	0	0	0	1	0	1
mmu-miR-223-3p	1.01E+08	Gm21170	0	0	0	1	0	1
mmu-miR-223-3p	1.01E+08	Gm21171	0	0	0	1	0	1
mmu-miR-223-3p	1.01E+08	Gm21190	0	0	0	1	0	1
mmu-miR-223-3p	1.01E+08	Gm21191	0	0	0	1	0	1
mmu-miR-223-3p	1.01E+08	Gm21209	0	0	0	1	0	1
mmu-miR-223-3p	1.01E+08	Gm21244	0	0	0	1	0	1
mmu-miR-223-3p	1.01E+08	Gm21293	0	0	0	1	0	1
mmu-miR-223-3p	1.01E+08	Gm21304	0	0	0	1	0	1
mmu-miR-223-3p	1.01E+08	Gm21312	0	0	0	1	0	1
mmu-miR-223-3p	1.01E+08	Gm21319	0	0	0	1	0	1
mmu-miR-223-3p	1.01E+08	LOC100861934	0	0	0	1	0	1
mmu-miR-223-3p	1.01E+08	Gm13123	0	0	0	1	0	1
mmu-miR-223-3p	1.01E+08	LOC100861952	0	0	0	1	0	1
mmu-miR-223-3p	1.01E+08	Gm21399	0	0	0	1	0	1
mmu-miR-223-3p	1.01E+08	LOC100862035	0	0	0	1	0	1
mmu-miR-223-3p	1.01E+08	Gm21440	0	0	0	1	0	1
mmu-miR-223-3p	1.01E+08	Gm21451	0	0	0	1	0	1
mmu-miR-223-3p	1.01E+08	Gm21454	0	0	0	1	0	1
mmu-miR-223-3p	1.01E+08	Gm21498	0	0	0	1	0	1
mmu-miR-223-3p	1.01E+08	Gm21518	0	0	0	1	0	1
mmu-miR-223-3p	1.01E+08	Gm21540	0	0	0	1	0	1
mmu-miR-223-3p	1.01E+08	Gm21541	0	0	0	1	0	1
mmu-miR-223-3p	1.01E+08	Gm21596	0	0	0	1	0	1
mmu-miR-223-3p	1.01E+08	Gm21647	0	0	0	1	0	1
mmu-miR-223-3p	1.01E+08	Gm21677	0	0	0	1	0	1
mmu-miR-223-3p	1.01E+08	Gm21678	0	0	0	1	0	1
mmu-miR-223-3p	1.01E+08	Gm21693	0	0	0	1	0	1
mmu-miR-223-3p	1.01E+08	Gm21704	0	0	0	1	0	1
mmu-miR-223-3p	1.01E+08	Gm21708	0	0	0	1	0	1
mmu-miR-223-3p	1.01E+08	LOC100862401	0	0	0	1	0	1
mmu-miR-223-3p	1.01E+08	LOC100862531	0	0	0	1	0	1

mmu-miR-223-3p	1.01E+08	LOC100862548	0	0	0	1	0	1
mmu-miR-223-3p	1.01E+08	LOC100862578	1	0	0	0	0	1
mmu-miR-223-3p	1.01E+08	LOC100862594	0	0	0	1	0	1
mmu-miR-223-3p	1.01E+08	LOC100862627	0	0	0	1	0	1
mmu-miR-223-3p	1.01E+08	LOC101055631	0	0	0	1	0	1
mmu-miR-223-3p	1.01E+08	LOC101055633	0	0	0	1	0	1
mmu-miR-223-3p	1.01E+08	LOC101055642	0	0	0	1	0	1
mmu-miR-223-3p	1.01E+08	LOC101055666	0	0	0	1	0	1
mmu-miR-223-3p	1.01E+08	LOC101055670	0	0	0	1	0	1
mmu-miR-223-3p	1.01E+08	LOC101055680	0	0	0	1	0	1
mmu-miR-223-3p	1.01E+08	LOC101055686	1	0	0	0	0	1
mmu-miR-223-3p	1.01E+08	LOC101055698	0	0	0	1	0	1
mmu-miR-223-3p	1.01E+08	LOC101055714	0	0	0	1	0	1
mmu-miR-223-3p	1.01E+08	LOC101055715	0	0	0	1	0	1
mmu-miR-223-3p	1.01E+08	LOC101055726	0	0	0	1	0	1
mmu-miR-223-3p	1.01E+08	LOC101055733	0	0	0	1	0	1
mmu-miR-223-3p	1.01E+08	LOC101055739	0	0	0	1	0	1
mmu-miR-223-3p	1.01E+08	LOC101055743	0	0	0	1	0	1
mmu-miR-223-3p	1.01E+08	LOC101055747	0	0	0	1	0	1
mmu-miR-223-3p	1.01E+08	LOC101055751	0	0	0	1	0	1
mmu-miR-223-3p	1.01E+08	LOC101055755	0	0	0	1	0	1
mmu-miR-223-3p	1.01E+08	LOC101055759	0	0	0	1	0	1
mmu-miR-223-3p	1.01E+08	LOC101055764	0	0	0	1	0	1
mmu-miR-223-3p	1.01E+08	LOC101055773	0	0	0	1	0	1
mmu-miR-223-3p	1.01E+08	LOC101055785	0	0	0	1	0	1
mmu-miR-223-3p	1.01E+08	LOC101055794	0	0	0	1	0	1
mmu-miR-223-3p	1.01E+08	LOC101055798	0	0	0	1	0	1
mmu-miR-223-3p	1.01E+08	LOC101055801	0	0	0	1	0	1
mmu-miR-223-3p	1.01E+08	LOC101055810	0	0	0	1	0	1
mmu-miR-223-3p	1.01E+08	LOC101055814	0	0	0	1	0	1
mmu-miR-223-3p	1.01E+08	LOC101055815	1	0	0	0	0	1
mmu-miR-223-3p	1.01E+08	LOC101055816	0	0	0	1	0	1
mmu-miR-223-3p	1.01E+08	LOC101055819	0	0	0	1	0	1
mmu-miR-223-3p	1.01E+08	LOC101055820	0	0	0	1	0	1
mmu-miR-223-3p	1.01E+08	LOC101055822	0	0	0	1	0	1
mmu-miR-223-3p	1.01E+08	LOC101055828	0	0	0	1	0	1
mmu-miR-223-3p	1.01E+08	LOC101055835	0	0	0	1	0	1
mmu-miR-223-3p	1.01E+08	LOC101055843	0	0	0	1	0	1
mmu-miR-223-3p	1.01E+08	LOC101055850	0	0	0	1	0	1
mmu-miR-223-3p	1.01E+08	LOC101055854	0	0	0	1	0	1
mmu-miR-223-3p	1.01E+08	LOC101055860	0	0	0	1	0	1
mmu-miR-223-3p	1.01E+08	LOC101055869	0	0	0	1	0	1
mmu-miR-223-3p	1.01E+08	LOC101055883	0	0	0	1	0	1
mmu-miR-223-3p	1.01E+08	LOC101055909	0	0	0	1	0	1
mmu-miR-223-3p	1.01E+08	LOC101055924	0	0	0	1	0	1
mmu-miR-223-3p	1.01E+08	LOC101055928	0	0	0	1	0	1
mmu-miR-223-3p	1.01E+08	LOC101055929	0	0	0	1	0	1
mmu-miR-223-3p	1.01E+08	LOC101055930	0	0	0	1	0	1
mmu-miR-223-3p	1.01E+08	LOC101055940	0	0	0	1	0	1
mmu-miR-223-3p	1.01E+08	LOC101055946	0	0	0	1	0	1
mmu-miR-223-3p	1.01E+08	LOC101055948	0	0	0	1	0	1
mmu-miR-223-3p	1.01E+08	LOC101055953	0	0	0	1	0	1
mmu-miR-223-3p	1.01E+08	LOC101055955	0	0	0	1	0	1
mmu-miR-223-3p	1.01E+08	LOC101055969	0	0	0	1	0	1
mmu-miR-223-3p	1.01E+08	LOC101055975	0	0	0	1	0	1

mmu-miR-223-3p	1.01E+08	LOC101055983	0	1	0	0	0	1
mmu-miR-223-3p	1.01E+08	LOC101056001	0	0	0	1	0	1
mmu-miR-223-3p	1.01E+08	LOC101056011	0	0	0	1	0	1
mmu-miR-223-3p	1.01E+08	LOC101056015	0	0	0	1	0	1
mmu-miR-223-3p	1.01E+08	LOC101056021	0	0	0	1	0	1
mmu-miR-223-3p	1.01E+08	LOC101056032	0	0	0	1	0	1
mmu-miR-223-3p	1.01E+08	LOC101056054	0	0	0	1	0	1
mmu-miR-223-3p	1.01E+08	LOC101056069	0	0	0	1	0	1
mmu-miR-223-3p	1.01E+08	LOC101056071	0	0	0	1	0	1
mmu-miR-223-3p	1.01E+08	LOC101056076	0	0	0	1	0	1
mmu-miR-223-3p	1.01E+08	LOC101056080	0	0	0	1	0	1
mmu-miR-223-3p	1.01E+08	LOC101056089	0	0	0	1	0	1
mmu-miR-223-3p	1.01E+08	LOC101056094	0	0	0	1	0	1
mmu-miR-223-3p	1.01E+08	LOC101056101	0	0	0	1	0	1
mmu-miR-223-3p	1.01E+08	LOC101056131	0	0	0	1	0	1
mmu-miR-223-3p	1.01E+08	LOC101056168	0	0	0	1	0	1
mmu-miR-223-3p	1.01E+08	LOC101056186	0	0	0	1	0	1
mmu-miR-223-3p	1.01E+08	LOC101056187	0	0	0	1	0	1
mmu-miR-223-3p	1.01E+08	LOC101056204	0	0	0	1	0	1
mmu-miR-223-3p	1.01E+08	LOC101056214	0	0	0	1	0	1
mmu-miR-223-3p	1.01E+08	LOC101056333	0	0	0	1	0	1
mmu-miR-223-3p	1.01E+08	LOC101056334	0	0	0	1	0	1
mmu-miR-223-3p	1.01E+08	LOC101056352	0	0	0	1	0	1
mmu-miR-223-3p	1.01E+08	LOC101056375	0	0	0	1	0	1
mmu-miR-223-3p	1.01E+08	LOC101056423	1	0	0	0	0	1
mmu-miR-223-3p	1.01E+08	LOC101056429	0	0	0	1	0	1
mmu-miR-223-3p	1.01E+08	LOC101056443	0	0	0	1	0	1
mmu-miR-223-3p	1.01E+08	LOC101056446	0	0	0	1	0	1
mmu-miR-223-3p	1.01E+08	LOC101056447	0	0	0	1	0	1
mmu-miR-223-3p	1.01E+08	LOC101056457	0	0	0	1	0	1
mmu-miR-223-3p	1.01E+08	LOC101056473	0	0	0	1	0	1
mmu-miR-223-3p	1.01E+08	LOC101056491	0	0	0	1	0	1
mmu-miR-223-3p	1.01E+08	LOC101056497	0	0	0	1	0	1
mmu-miR-223-3p	1.01E+08	LOC101056505	0	0	0	1	0	1
mmu-miR-223-3p	1.01E+08	LOC101056512	0	0	0	1	0	1
mmu-miR-223-3p	1.01E+08	LOC101056514	0	0	0	1	0	1
mmu-miR-223-3p	1.01E+08	LOC101056541	0	0	0	1	0	1
mmu-miR-223-3p	1.01E+08	LOC101056551	0	0	0	1	0	1
mmu-miR-223-3p	1.01E+08	LOC101056555	0	0	0	1	0	1
mmu-miR-223-3p	1.01E+08	LOC101056558	0	0	0	1	0	1
mmu-miR-223-3p	1.01E+08	LOC101056559	0	0	0	1	0	1
mmu-miR-223-3p	1.01E+08	LOC101056567	0	0	0	1	0	1
mmu-miR-223-3p	1.01E+08	LOC101056589	1	0	0	0	0	1
mmu-miR-223-3p	1.01E+08	LOC101056619	0	0	0	1	0	1
mmu-miR-223-3p	1.01E+08	LOC101056632	0	0	0	1	0	1
mmu-miR-223-3p	1.01E+08	LOC101056649	0	0	0	1	0	1
mmu-miR-223-3p	1.01E+08	LOC101056662	0	0	0	1	0	1
mmu-miR-223-3p	1.01E+08	LOC101056668	0	0	0	1	0	1
mmu-miR-223-3p	1.01E+08	LOC101056669	0	0	0	1	0	1
mmu-miR-223-3p	1.01E+08	LOC101056679	0	0	0	1	0	1
mmu-miR-223-3p	1.01E+08	LOC101056680	0	0	0	1	0	1
mmu-miR-223-3p	1.01E+08	LOC101056691	0	0	0	1	0	1
